# Genomic landscape of high-grade meningiomas

**DOI:** 10.1038/s41525-017-0014-7

**Published:** 2017-04-26

**Authors:** Wenya Linda Bi, Noah F. Greenwald, Malak Abedalthagafi, Jeremiah Wala, Will J. Gibson, Pankaj K. Agarwalla, Peleg Horowitz, Steven E. Schumacher, Ekaterina Esaulova, Yu Mei, Aaron Chevalier, Matthew A. Ducar, Aaron R. Thorner, Paul van Hummelen, Anat O. Stemmer-Rachamimov, Maksym Artyomov, Ossama Al-Mefty, Gavin P. Dunn, Sandro Santagata, Ian F. Dunn, Rameen Beroukhim

**Affiliations:** 1 Center for Skull Base and Pituitary Surgery, Department of Neurosurgery, Brigham and Women’s Hospital, Harvard Medical School, Boston, MA USA; 2 Department of Cancer Biology, Dana-Farber Cancer Institute, Boston, MA USA; 3 Broad Institute of MIT and Harvard, Cambridge, MA USA; 4 Division of Neuropathology, Department of Pathology, Brigham and Women’s Hospital, Boston, MA USA; 5 Research Center, King Fahad Medical City, Riyadh, Saudi Arabia; 6 The Saudi Human Genome Project Lab, King Abdulaziz City for Science and Technology, Riyadh, Saudi Arabia; 7 Department of Neurosurgery, Massachusetts General Hospital, Boston, MA USA; 8 Department of Surgery, The University of Chicago, Chicago, IL USA; 9 Department of Pathology and Immunology, Washington University School of Medicine, St. Louis, MO USA; 10 Computer Technologies Department, ITMO University, Saint Petersburg, Russia; 11 Center for Cancer Genome Discovery, Dana-Farber Cancer Institute, Boston, MA USA; 12 Department of Pathology, Massachusetts General Hospital, Boston, MA USA; 13 Department of Neurosurgery, Washington University School of Medicine, St. Louis, MO USA; 14 Center for Human Immunology and Immunotherapy Programs, Washington University School of Medicine, St. Louis, MO USA; 15 Department of Medical Oncology, Dana-Farber Cancer Institute, Boston, MA USA

## Abstract

High-grade meningiomas frequently recur and are associated with high rates of morbidity and mortality. To determine the factors that promote the development and evolution of these tumors, we analyzed the genomes of 134 high-grade meningiomas and compared this information with data from 595 previously published meningiomas. High-grade meningiomas had a higher mutation burden than low-grade meningiomas but did not harbor any significantly mutated genes aside from *NF2*. High-grade meningiomas also possessed significantly elevated rates of chromosomal gains and losses, especially among tumors with monosomy 22. Meningiomas previously treated with adjuvant radiation had significantly more copy number alterations than radiation-induced or radiation-naïve meningiomas. Across serial recurrences, genomic disruption preceded the emergence of nearly all mutations, remained largely uniform across time, and when present in low-grade meningiomas correlated with subsequent progression to a higher grade. In contrast to the largely stable copy number alterations, mutations were strikingly heterogeneous across tumor recurrences, likely due to extensive geographic heterogeneity in the primary tumor. While high-grade meningiomas harbored significantly fewer overtly targetable alterations than low-grade meningiomas, they contained numerous mutations that are predicted to be neoantigens, suggesting that immunologic targeting may be of therapeutic value.

## Brain tumors: uncovering genomic disruption in meningiomas

Meningiomas, which arise from the tissue surrounding the brain and spinal cord, are the most common primary brain tumor in adults. The majority of these are slow-growing and amenable to surgical resection, if treatment is indicated. However, a subset of aggressive meningiomas are considered high-grade, producing significantly worse mortality. In a first study of its kind, Drs. Wenya Linda Bi, Ian Dunn, Sandro Santagata, Rameen Beroukhim, and colleagues at Harvard Medical School sequenced the genomes of 134 high-grade meningiomas and compared their makeup with lower-grade meningiomas. They found that aggressive tumors were more likely to harbor mutations in the *NF2* gene and exhibit widespread genomic disruption. They also harbored an elevated rate of predicted immunogenic mutations, with implications for the use of immuno-modulatory therapies.

## Introduction

Meningiomas, tumors arising from the arachnoid cap cells that surround the brain, are the most common primary tumor of the central nervous system.^1^ The majority of meningiomas are low-grade (grade I) neoplasms that may be effectively managed by surgical resection. A significant subset of meningiomas, however, have aggressive features and are associated with insidious growth, frequent recurrence, and poor progression-free survival (grade II–III).^2^ Despite the greater recurrence rates and mortality that results from high-grade meningiomas, our understanding of the genomic aberrations that drive these tumors remains incomplete. Numerous clinical trials have failed to identify systemic medical therapies that can effectively control the relentless growth of these tumors.^3^

The discovery that mutation of the *NF2* gene was responsible for Neurofibromatosis 2, an inherited genetic disorder characterized by the development of schwannomas and meningiomas, was a substantial step forward in the characterization of the pathobiology of meningioma and marked these tumors as one of the first to be associated with a genomic driver.^4^ Subsequent analyses of sporadic meningiomas identified inactivating mutations and copy loss of the region on 22q harboring the *NF2* gene in approximately 40–60% of cases.^5–7^ Recently, our group and others used next-generation sequencing methods to identify recurrent mutations in several additional genes besides *NF2*, including *v-Akt murine thymoma viral oncogene homolog 1* (*AKT1*) and *3* (*AKT3*), *Phosphatidylinositol 3-kinase* (*PIK3CA*), *Smoothened* (*SMO*), *SUFU negative regulator of hedgehog signaling* (*SUFU*), *TNF receptor-associated factor 7* (*TRAF7*), *Kruppel-like factor 4* (*KLF4*), *SWI/SNF-related, matrix-associated, actin-dependent regulator of chromatin, subfamily b, member 1* (*SMARCB1*), *RNA polymerase II subunit A* (*POLR2A*) and *BRCA1-associated protein 1* (*BAP1*), in addition to alterations in the promoter region of the *Telomerase reverse transcriptase* (*TERT*) gene.^6–11^ Notably, mutations in *AKT1*, *PIK3CA*, and *SMO* are associated with downstream activation of proto-oncogenic pathways, making these proteins logical targets for attempts at pharmacologic inhibition.

However, these alterations have been observed predominantly in low-grade meningiomas, while the genomic landscape of high-grade meningiomas remains largely unexplored. Little is known about the differences in driver alterations between low-grade and high-grade tumors, whether there are specific genetic events that are correlated with progression to higher grade, or how these tumors respond to treatment, specifically surgical resection followed by radiation. To address these questions, we genomically characterized a large cohort of sporadic high-grade meningiomas in order to identify mutations, copy number alterations, and rearrangements, as well to chart their evolution over time, with the goal of furthering our understanding of the most aggressive forms of meningioma.

## Results

We performed genome-level sequencing using either whole genome sequencing (WGS) or whole exome sequencing (WES) on 66 samples from 39 individuals with high-grade meningiomas. Tumors from nine of these individuals were analyzed with WGS (mean coverage of 37×). Three of these individuals also had tumors that were analyzed with WES and an additional 30 patients had tumors analyzed with WES. In all, a total of 57 meningiomas were characterized with WES (mean coverage of 91×). Eleven of the patients had multiple recurrences that were sequenced. We also sequenced selected genes in 76 additional high-grade meningiomas for a total of 134 high-grade meningiomas (Supplementary Table 1 and Supplementary Fig. 1). We compared the data derived from these 134 samples to previously published data from 595 sporadic meningiomas profiled with whole genome or WES (108 low-grade and 22 high-grade tumors), and targeted capture sequencing (348 low-grade and 117 high-grade tumors).^6, 7, 9–12^ We used these data to characterize somatic mutations (including insertions and deletions), somatic copy number alterations (SCNAs), and rearrangements genome-wide.

### High-grade meningiomas exhibit a relatively high somatic mutation burden with few recurrent events

**Fig. 1:**
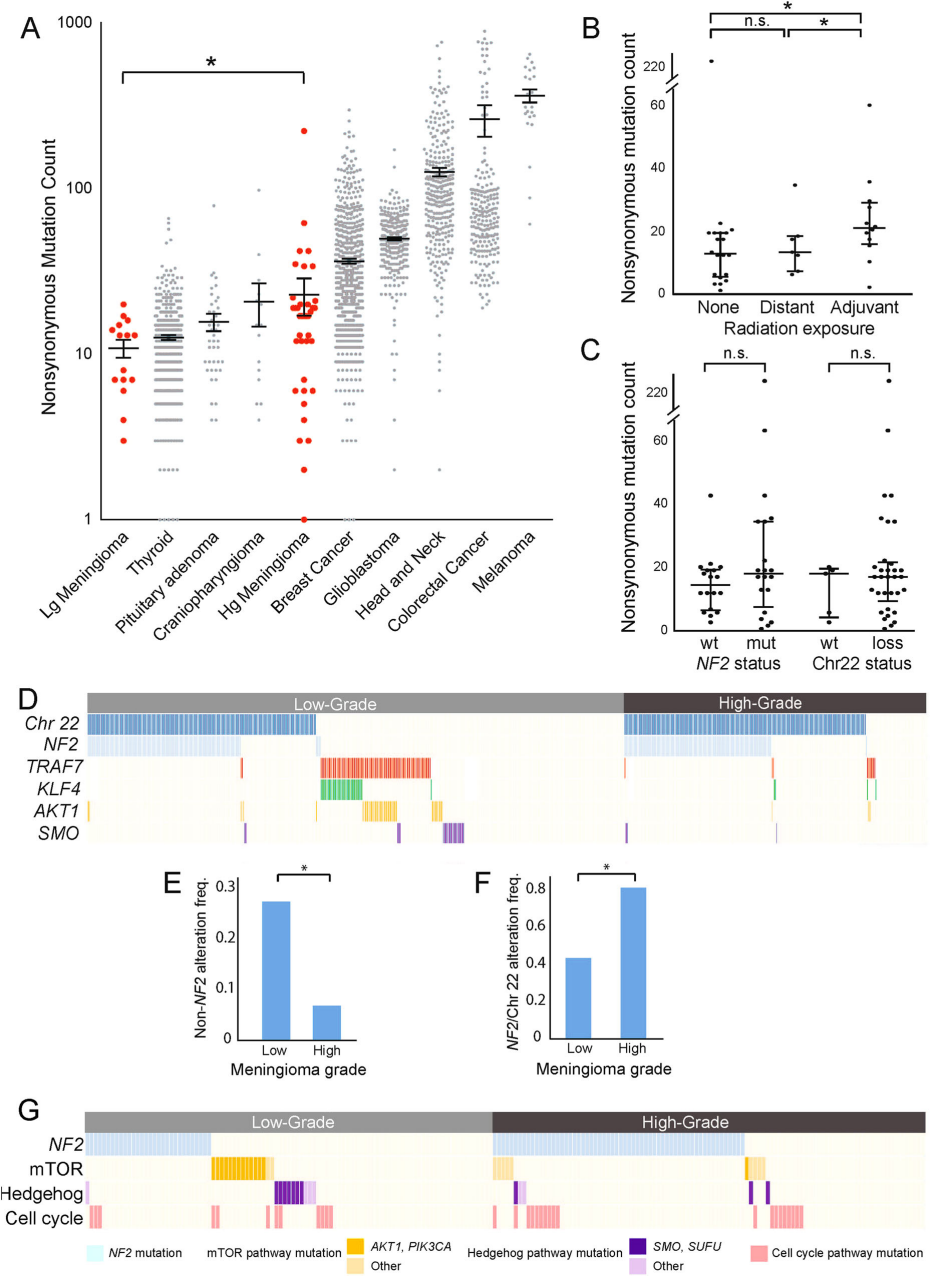
Mutational characteristics of high-grade meningioma. **a** Nonsynonymous mutation counts (*y*-axis) per sample for a selection of tumor types (*x*-axis). **b** Nonsynonymous mutation counts (*y*-axis) for meningiomas stratified by prior radiation exposure (*x*-axis). **c** Nonsynonymous mutation counts (*y*-axis) for meningiomas with or without *NF2* mutations or chromosome 22 loss (*x*-axis). **d** Chromosome 22 loss or canonical meningioma gene mutation (*y*-axis) across 702 aggregated samples (*x*-axis); each column represents one sample and *white space* indicates lack of coverage. **e** Percentage of samples (*y*-axis) with *NF2* mutation or chromosome 22 loss stratified by grade (*x*-axis). **f** Percentage of samples (*y*-axis) with non-*NF2* driver mutations stratified by grade (*x*-axis). **g** Presence of mutations in meningioma-associated pathways (*y*-axis) across 200 samples with genomic characterization (*x*-axis). *Dark colors* correspond to canonical alterations; *lighter hues* represent non-canonical alterations in the pathway. *Lg meningioma* low-grade meningioma, *Hg meningioma* high-grade meningioma, *wt* wild-type, *mut* mutant, *n.s*. not significant. *Error bars* and *central values* represent mean with s.e.m. (**a**) or median with i.q.r. (**b**, **c**)

Across 39 high-grade meningiomas from unique individuals, we observed an average of 23 (range 1–223) nonsynonymous coding alterations. This is a significantly higher rate than in low-grade meningiomas and comparable to that of thyroid cancer and craniopharyngioma, but lower than that seen in head and neck tumors, colorectal carcinoma, and melanoma (Fig. 1a).^6, 13^ The mutations tended to be C>T transitions (Supplementary Fig. 2A), a change shown to result from the increased rate of deamination of cytosine relative to other bases, and which represents the most common mutational signature found in an analysis of over 30 different cancer types.^14^

We observed one sample with a mutation load that was significantly greater than the rest of the cohort. This hypermutator phenotype did not correspond to a change in the overall mutational signature of this tumor compared with the rest of the cohort (Supplementary Fig. 2B). We also did not identify any mismatch repair pathway alterations in this sample, which have been shown to produce elevated rates of mutations in other cancer types.^15^

Meningiomas exposed to adjuvant radiation exhibited a higher mutation burden (mean of 23) than both radiation-naïve meningiomas and tumors that arose in patients who had received prior radiation treatment to the brain, commonly referred to as “radiation-induced meningiomas” (mean of 14, *p* = 0.001; Fig. 1b). We did not detect significant differences in mutational signatures between irradiated and non-irradiated samples (Supplementary Fig. 2C).

The most frequently mutated gene among these 39 tumors was *NF2*, which was mutated in 46% of samples. Of the 18 *NF2* alterations, 89% were inactivating frame-shift, splice site, or nonsense alterations, consistent with the known tumor suppressor role of *NF2*, compared with only 16% of non-*NF2* mutations (*p* = 6 × 10^−10^). The average allelic fraction of *NF2* alterations was 57% (range 19–84%), in comparison with the cohort-wide average of 25% among all other mutations (*p* = 3.7 × 10^−6^), suggesting that *NF2* mutations tend to be shared by a larger fraction of cells in the tumor relative to other mutations. There was no significant difference in mutation burden between samples with and without *NF2* mutations or chromosome 22 loss (*p* = 0.34, Fig. 1c).

In addition to *NF2*, 54 genes exhibited recurrent mutations (range 2–4, Supplementary Table 2). However, none of these genes reached a significant level of recurrence after accounting for gene-specific mutation rates and multiple hypothesis correction (see “Methods”).

Next, we performed targeted sequencing (mean coverage of 185x) of 34 of the recurrently mutated genes that we had identified in the discovery cohort in an additional cohort of 76 independent high-grade meningioma samples (72 grade II, 4 grade III; Supplementary Tables 1 and 3). Aside from *NF2*, none of these 34 genes harbored significantly recurrent mutations in the combined discovery and extension cohorts. *NF2* was mutated in 53% (62/115) of all samples at an average allelic fraction of 55%, compared with allelic fractions of 26% among all other genes (*p* < 1 × 10^−16^). Other than *NF2*, the most frequently mutated genes were *CDC27* and *LRP1B*, mutated in 10 (9%) and 9 (8%) samples, respectively, of the total set of 115 meningiomas (Supplementary Table 4). Both of these genes undergo high rates of mutation across cancer types, likely due to a high background rate of mutation.^16^

### High-grade and low-grade meningiomas differ in genomic profile

The finding that no genes other than *NF2* were significantly mutated in high-grade meningiomas suggests that high-grade meningiomas are much less likely to harbor mutations in known drivers of low-grade meningioma, including *TRAF7*, *KLF4*, *AKT1*, or *SMO*. To evaluate this possibility, we sequenced these genes, which are the four most common non-*NF2* driver alterations, in our extension cohort of 76 samples. We then integrated the data with previously published sequencing data from 595 additional meningiomas.^6, 7, 9, 10, 12^ For each gene, we compared rates of mutation among both high-grade and low-grade meningiomas (Fig. 1d).^6, 7^ Mutations in each of these genes occurred significantly less frequently in high-grade meningiomas (*p* = 0.03 for each, Fig. 1e). Among these genes, *TRAF7* was mutated most frequently among the high-grade meningiomas, but only in 10 of the 254 samples (4%).

Conversely, alterations in *NF2* or chromosome 22 occurred significantly more frequently in high-grade (80%) than low-grade meningiomas (43%, *p* < 1 × 10^−16^; Fig. 1f). Such elevated rates of *NF2* driver events may partly explain why non-*NF2* driver genes are mutated significantly less frequently in high-grade than low-grade tumors: the non-*NF2* drivers are mutated most often in *NF2* wild-type tumors (Fig. 1d), and there are fewer such tumors among high-grade cases (Supplementary Fig. 3A).^6, 7^ However, even among high-grade tumors without *NF2* alterations, the difference in rates of non-*NF2* driver mutations remained statistically significant (*p* < 3 × 10^−5^).

Across the cohort of 702 high-grade and low-grade meningiomas from unique patients, loss of chromosome 22 and mutations in *NF2* tended to co-occur (*p* < 2 × 10^−16^). The presence of a mutation in any of the four non-*NF2* drivers was anti-correlated with the presence of an *NF2* mutation (*p* < 2 × 10^−16^) and with loss of chromosome 22 (*p* < 2 × 10^−16^). Even in the 309 meningiomas with chromosome 22 loss, these canonical non-*NF2* mutations were mutually exclusive with *NF2* mutations (*p* = 0.02), suggesting that loss of chromosome 22 may not be driving bi-allelic inactivation of *NF2* in tumors with non-*NF2* driver alterations.

We further evaluated an additional 143 genes that have been reported to be altered in low-grade or high-grade meningioma, or were in the same pathways as known meningioma drivers, in 201 meningiomas (Supplementary Table 3). We detected 25 alterations in the mTOR pathway (including mutations in *AKT1*, *PIK3CA*, *MTOR*, *TSC2*, and *RICTOR*), 16 alterations in the Hedgehog pathway (including mutations in *SMO* and *SUFU*), and two mutations in *TP53*, which is downstream of *KLF4*. However, mutations in these pathways were not significantly enriched above the background rate (Fig. 1g).

The lack of significantly recurrent mutations, other than in *NF2*, among high-grade tumors indicates that it is unlikely that any individual gene is mutated in more than 20% of high-grade tumors. We did not find any genes other than *NF2* mutated in more than 10 patients, which would represent a mutation rate of 13%. We had 95% power to detect genes mutated in at least 19% of patients, and 50% power to detect genes mutated in 13% of patients (Supplementary Fig. 3B).

### High-grade meningiomas demonstrate frequent copy number alterations

**Fig. 2:**
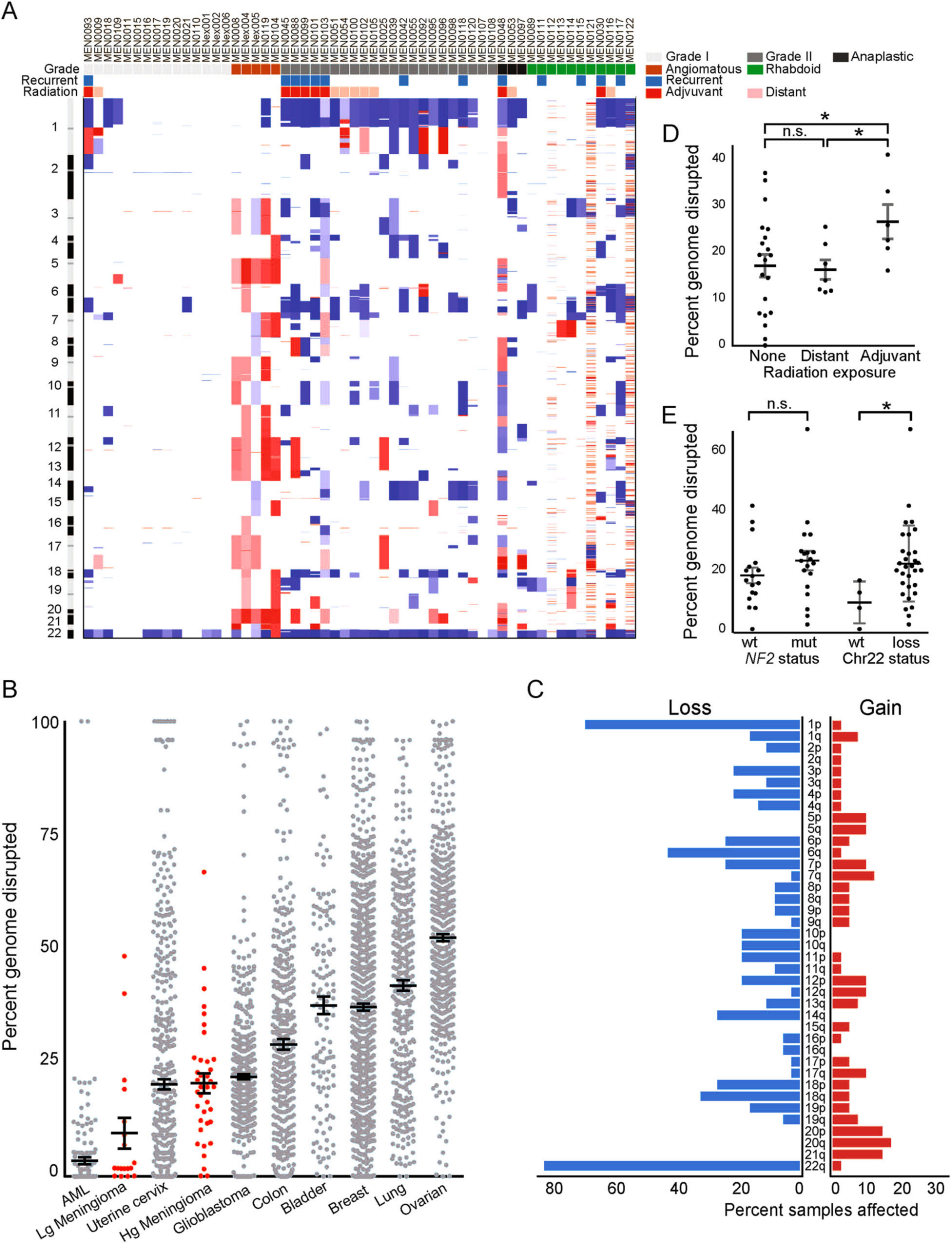
Landscape of copy number alterations in meningioma. **a** Heatmap of gains (*red*) and losses (*blue*) across the genome (*y*-axis) for 56 samples (*x*-axis) with whole exome or WGS. Pathological features, primary or recurrent status, and exposure to distant (radiation-induced) or recent adjuvant radiation are annotated. **b** Percent genome disrupted (*y*-axis) for low-grade and high-grade meningiomas, as compared with eight other cancers (*x*-axis). **c** Percent incidence (*x*-axis) of chromosome arm-level gains and losses (*y*-axis). **d** Percent of genome disrupted (*y*-axis) for meningiomas stratified by radiation exposure (*x*-axis). **e** Percent of genome disrupted (*y*-axis) for meningiomas stratified by *NF2* mutation or chromosome 22 loss (*x*-axis); angiomatous meningiomas were excluded due to their markedly different genomic profile. *AML* acute myeloid leukemia, *Lg meningioma* low-grade meningioma, *Hg meningioma* high-grade meningioma, *GBM* glioblastoma, *n.s*. not significant. *Error bars* and *central values* represent mean with s.e.m

We next investigated chromosomal instability across meningiomas (Fig. 2a). Similar to mutation burden, high-grade meningiomas have significantly higher levels of genomic disruption than low-grade meningiomas (3% vs. 19%, *p* < 1 × 10^−8^), and disruption rates comparable to other aggressive systemic and CNS cancers such as glioblastoma (Fig. 2b).^17^

Loss of chromosome 22 was the most frequent arm-level SCNA, observed in 56% of 702 sequenced meningiomas (Fig. 1d). Losses of chromosomes 1p, 6q, 10q, and 18q, along with gains of 17q and 20q, were also recurrent across high-grade meningiomas (Fig. 2c), consistent with previous cytogenetic studies.^18^ High-grade meningiomas harbored loss of chromosome 22 more frequently (87%) than low-grade meningiomas (58%, *p* = 0.03), with chromosome 1p loss being the second most common SCNA. We did not detect any significantly recurrent focal copy number alterations.

Within our high-grade meningioma cohort, 7 tumors were considered radiation-induced, as they originated following distant exposure to therapeutic radiation for other reasons, and 11 tumors were treated with radiation following an initial diagnosis of meningioma. Meningiomas treated with adjuvant radiation exhibited a significantly higher burden of copy number alterations than radiation-induced or non-irradiated meningiomas (*p* = 0.02, Fig. 2d). While there was no significant difference in genomic disruption between *NF2-*mutant and *NF2*-wild-type samples, meningiomas with chromosome 22 loss demonstrated increased rates of genomic disruption, even after excluding chromosome 22 (*p* = 0.05, Fig. 2e).

### Complex rearrangements are prevalent in both low-grade and high-grade meningiomas

**Fig. 3:**
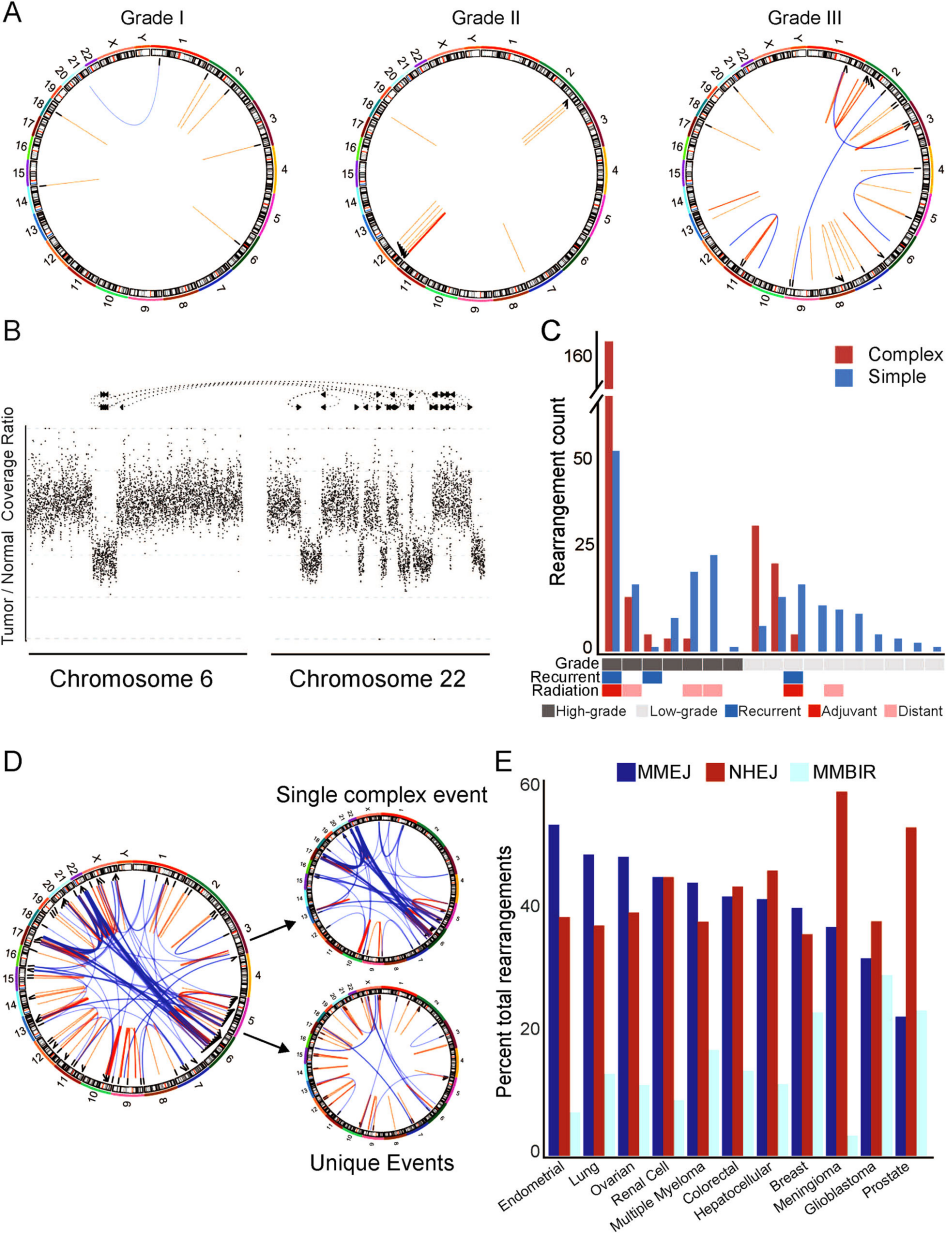
Characteristics of meningioma rearrangements. **a** Representative Circos plots for three samples (MEN0042, MEN0011, MEN0053), with lines between genomic coordinates representing intrachromosomal (*orange*) or interchromosomal (*blue*) rearrangements. **b** Example of a complex event involving multiple genomic positions (*x*-axis) with associated changes in read coverage (*y*-axis). **c** Number of rearrangements (*y*-axis) per sample (*x*-axis) broken down by complex (*orange*) or simple (*blue*) event type. **d** Circos plot of a hyper-rearranged grade III meningioma and the deconstruction of complex and simple rearrangements to the overall makeup of this sample. **e** Percent incidence of different mechanisms driving rearrangement formation (*y*-axis) across multiple cancer types (*x*-axis). *MMEJ* micro-homology-mediated end joining, *NHEJ* non-homologous end joining, *MMBIR* micro-homology-mediated break-induced repair

We next sought to determine the burden of rearrangements in 19 meningiomas (8 high-grade, 11 low-grade) with WGS data (Fig. 3a). We detected a median of 21.5 rearrangements per high-grade tumor (range 0–217, SD = 72), compared with five rearrangements per low-grade tumor (range 0–39, SD = 12; *p* = 0.15), for a total of 446 distinct rearrangements across our cohort (Supplementary Table 5A). Translocations (interchromosomal rearrangements) represented 35% (157 of 446) of these events. Among the intrachromosomal rearrangements, deletions were the most common (26%), followed by inversions and duplications (20 and 11%, respectively; Supplementary Fig. 4A–B).

*NF2* was the most frequently rearranged gene across our cohort (*n* = 4). These rearrangements all occurred in samples with chromosome 22 loss and never in conjunction with mutations or indels in *NF2*, indicating that they likely represented a second hit resulting in bi-allelic *NF2* inactivation.

We also detected recurrent rearrangements in 11 additional genes. These included three patients with rearrangements in the microtubule-associated GTPase *DNM3*, which is likely a fragile site. The remaining 10 genes were each rearranged in two patients, including the serine/threonine kinase *CDK14*, the neuronal growth regulator *NEGR1*, and the Rho GTPase family guanine nucleotide exchange factor *VAV3* (Supplementary Table 5B).

Prior work has demonstrated that rearrangements can sometimes occur as part of a single, catastrophic event. These phenomena include chromothripsis, where focal regions of one or two chromosomes are highly rearranged, and chromoplexy, which affects multiple chromosomes.^19, 20^ We classified groups of rearrangements with at least four linked alterations as part of a complex event (Fig. 3b). More than half (227 of 399; 57%) of all detected rearrangements were the result of a relatively small number of unique complex events (Fig. 3c). We detected 8 such events across our cohort, with a mean of 43 (median 24) individual rearrangements in each. The median number of rearrangements in meningiomas with complex events was significantly greater than in tumors without complex events (24 vs. 8, *p* = 0.03), and complex events were responsible for an average of 55% (range 22–100%) of the rearrangements in the samples in which they occurred.

One sample had 212 rearrangements and contributed more than half of the total observed rearrangements across all samples. It also had a highly disrupted copy number profile (Supplementary Fig. 4D). This sample harbored a *TP53* ^G187S^ mutation, which has been shown to be a recurrent *TP53* alteration across cancers.^21^ Alterations in p53 have not been found to be significantly recurrent in meningioma, but this alteration may explain the significant rates of disruption found in this sample. Nearly 75% of all rearrangements in this sample were due to a complex cluster that spanned seven different chromosomes (Fig. 3d).

Indeed, across the cohort, complex events often linked together geographically distinct regions of the genome, and on average included events from four chromosomes. We found that 53% of complex events were translocations, compared with only 21% of isolated events (*p* = 5 × 10^−11^; Supplementary Fig. 4C), largely as a result of the hyper-rearranged sample, in which 65% of complex rearrangements were translocations.

We next sought to categorize the mechanistic basis of detected rearrangements. Briefly, we classified the genomic context of each rearrangement according to the degree of sequence homology, overlap with repetitive elements, and size of junction insertion in order to characterize the biological double-strand break repair mechanism most likely to have generated it, according to previously described methods.^22^ Previous work analyzing 10 cancer types has suggested that micro-homology-mediated end joining (MMEJ) and non-homologous end joining (NHEJ) are the most frequent drivers of somatic rearrangements (41 and 39%, respectively). Using our own rearrangement detection pipeline, we likewise found that the majority (>90%) of observed rearrangements in meningioma have signatures consistent with MMEJ and NHEJ repair processes (Fig. 3e). Low-grade and high-grade meningiomas did not significantly differ in the composition (inversion, deletion, duplication, or translocation) or inferred mechanistic basis (MMEJ, NHEJ) of the detected rearrangements.

### Meningiomas exhibit substantial heterogeneity across space and time.

**Fig. 4:**
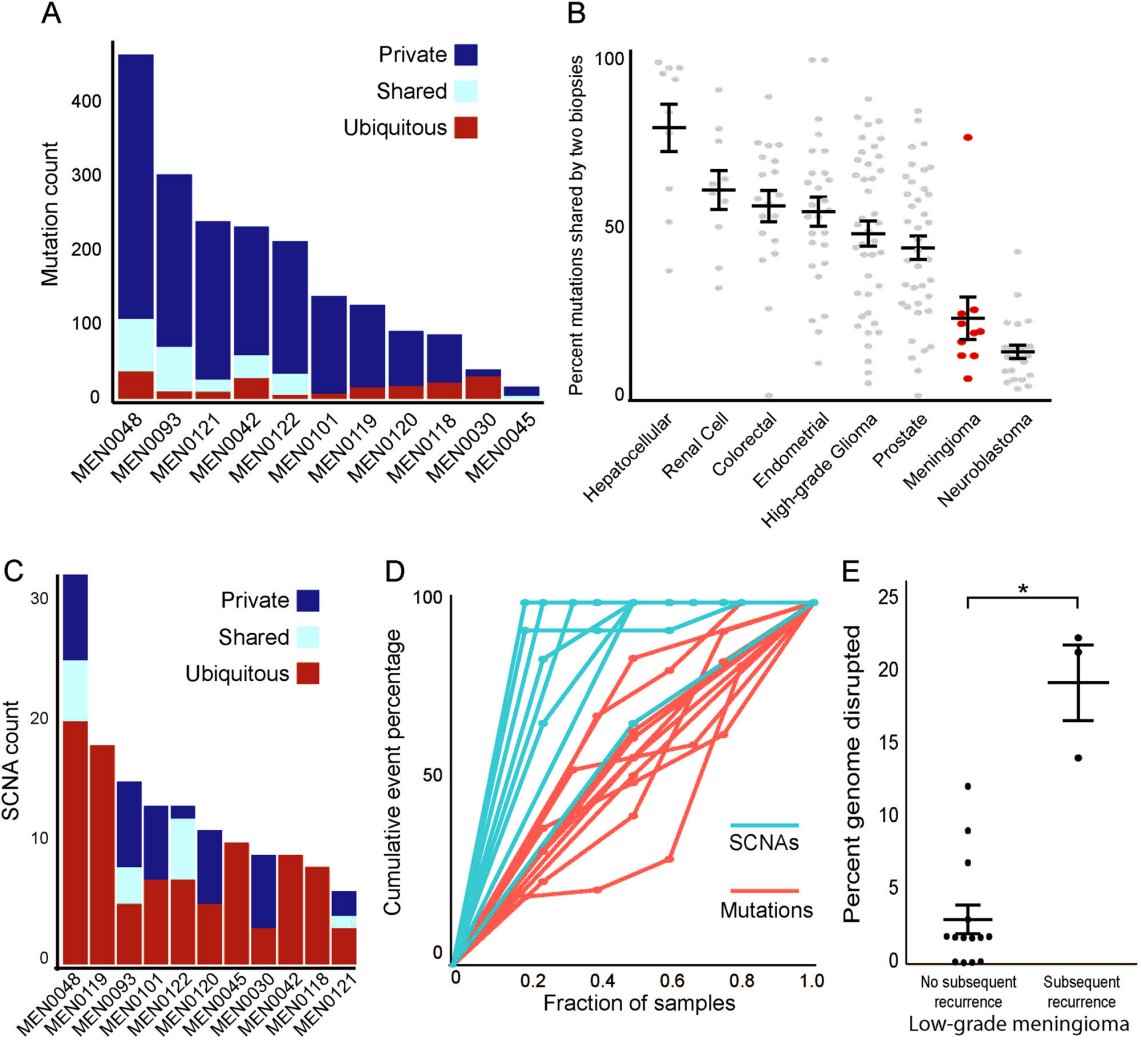
Intra-patient heterogeneity in meningioma. **a** Mutation count (*y*-axis) across 11 recurrent samples (*x*-axis) for mutations that are present in all biopsies (ubiquitous, *red*), some biopsies (shared, *teal*), or only a single biopsy (private, *blue*). **b** Percent of mutations shared from pairs of samples from the same patient (*y*-axis) across a variety of diverse cancer types (*x*-axis).^15–17, 19, 20, 22, 23^ **c** SCNA count (*y*-axis) across 11 recurrent samples (*x*-axis) for SCNAs that are present in all biopsies (ubiquitous, *red*), some biopsies (shared, *teal*), or only a single biopsy (private, *blue*). **d** Cumulative percentage of events per patient (*y*-axis) as a function of the percentage of samples examined (*x*-axis) for mutations (*red*) and SCNAs (*teal*). **e** Percent of genome disrupted (*y*-axis) for low-grade meningiomas, stratified by whether or not they went on to recur (*x*-axis). *Error bars* and *central values* represent mean with s.e.m

We next focused on 11 patients in our cohort with two or more serial meningioma resections (Supplementary Table 6). On average, 23% of specific mutations (range 5–77%) were shared across any pair of samples from the same patient (Fig. 4a). This heterogeneity is not an artifact of tumor impurity (see Supplementary Note 1), and represents a much higher level of heterogeneity than seen in many other cancer types (Fig. 4b).^23–29^

This heterogeneity could represent the appearance of new mutations over time, or it could represent geographic heterogeneity in the initial tumor. If the appearance of mutations over time were driving the observed heterogeneity, we would expect later tumors to exhibit a greater mutation burden than earlier tumors. However, we did not observe a statistically significant increase in the number of mutations between subsequent samples (Supplementary Fig. 5).

These results suggest that the observed heterogeneity is primarily spatial. To evaluate the contribution of spatial heterogeneity, we assessed four distinct areas from the same resection of a single patient and found that only 9% of the mutations were shared between any two of the regions. To validate this finding, we aggregated all samples in our cohort that had previously been characterized by next-generation sequencing of a custom cancer gene panel. This prior sequencing was done with DNA obtained from a different region of the same formalin-fixed paraffin embedded (FFPE) core, thus lending insight into the spatial heterogeneity of these tumors. We observed a striking dichotomy in rates of overlap between driver and passenger mutations. In particular, we found that 21 of 22 (95%) driver mutations were shared across cores from the same sample, compared with only 18/61 (30%) of putative passenger alterations that were covered by the targeted sequencing panel (*p* = 0.0001, Supplementary Table 7).

The finding that *NF2* is the most frequently altered gene in low-grade and high-grade meningiomas, combined with the finding that these mutations tend to have high allelic fractions and nearly always co-occur with loss of the wild-type copy of chromosome 22, has led to the presumption that *NF2* mutations are an initiating event in meningioma tumorigenesis. However, we observed three distinct inactivating *NF2* alterations across five recurrences in one patient (MEN0045). Conversely, all copy number alterations were shared by all recurrences in this patient. For this patient, the data suggest that *NF2* mutation, and thus bi-allelic inactivation, was not the initiating event, but was instead preceded by widespread genomic disruption.

Recurrent tumors demonstrated little variation in copy number profiles, with any two samples from the same patient sharing an average 75% of arm-level SCNAs. This represents a significantly elevated rate of overlap compared with mutations (Fig. 4a vs. 4c), where only 23% were shared across paired samples on average (*p* < 2 × 10^−5^; Fig. 4d), and suggests that SCNAs precede most non-driver mutations during the development of high-grade meningiomas.^30^

The homogeneity in copy number profile was observed even in samples that recurred at higher grade. Most low-grade meningioma tended to have few arm-level events other than loss of chromosome 22. However, those low-grade samples that later recurred as higher-grade meningiomas had significantly elevated rates of copy number alterations compared with tumors which did not (*p* < 0.0001; Fig. 4e).

### Temporal relationships indicate survival of multiple lineages across resections

**Fig. 5:**
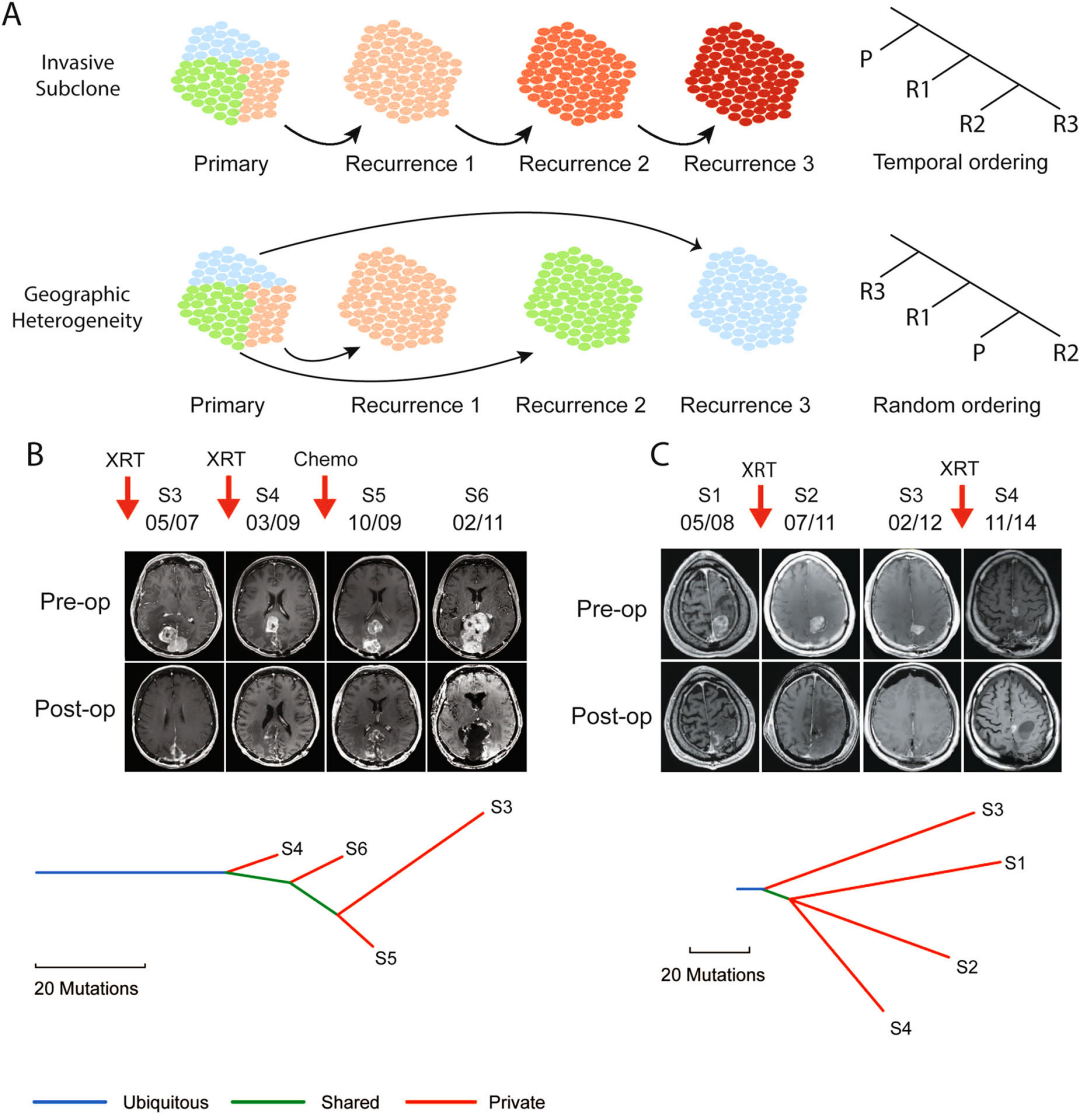
Phylogenetic analysis of recurrent meningioma. **a** Schematic illustrating the expected phylogenetic relationship across successive recurrences if a tumor evolves through progressive dominance of an invasive subclone (*top*) compared with outgrowth of subclones from a geographically heterogeneous primary (*bottom*). **b** Patient with a multiply recurrent parasagittal anaplastic meningioma that underwent serial resections as well as interval radiation (XRT) and sunitinib (chemo). Pre-operative and post-operative MR imaging (*top*) from the third (S3), fourth (S4), fifth (S5), and sixth (S6) resections, spanning a 4-year interval, demonstrates a heterogeneous pattern and location of tumor regrowth despite excellent resections. Phylogenetic tree (*bottom*) demonstrates a branched evolution of the mutations associated with each tumor resection (S3–S6). **c** Pre-operative and post-operative MRIs (*top*) and phylogenetic tree (*bottom*) of four serial resections (S1–S4) over 6 years in a patient with recurrent rhabdoid meningioma

Successive recurrences of the same meningioma may comprise either a single invasive subclone that develops a growth advantage compared with other initially co-existing subclones, or different subclones that are present in geographically distinct regions of the primary meningioma which can each subsequently emerge. In the former case, one would expect successive resections to be more closely related to each other than to the primary resection, whereas the latter case would imply a random ordering to the relationships between successive resections (Fig. 5a).

To distinguish between these possibilities, we constructed phylogenetic trees to assess the evolution of tumors over time among six patients for whom we had sequenced more than two recurrences. In five of the six cases, we found that samples that did not immediately precede one another were the most closely related (Supplementary Fig. 6). For example, in one case, the fifth and third resection were more similar to one another than to any of the other sequenced samples (Fig. 5b). In another, the primary tumor and fourth resection were more similar to one another than to the third resection (Fig. 5c). These results suggest that subsequent recurrences reflect geographically distinct unresected regions rather than a dominant invasive subclone that originates from the primary tumor.

### Genomic features associate with high-grade meningioma subtype and location

It is increasingly appreciated that the driver mutations of meningiomas are associated with particular histopathologic subtypes and with anatomic locations in the cranium.^6, 7, 9, 18^ Recent work has shown that a subset of rhabdoid meningiomas, but not meningiomas of other histological subtypes, harbor mutations in the tumor suppressor gene *BAP1*.^11^ Across high-grade meningiomas, we found that the rhabdoid subtype was associated with a significantly lower incidence of chromosome 1p losses compared with other high-grade meningiomas (*p* = 0.002, Fig. 2a), consistent with a distinct pathogenesis. Angiomatous meningiomas also demonstrated a markedly different copy number profile from other meningiomas, with frequent arm-level gains, consistent with previous cytogenetics observations.^31^ These spanned both classic grade I angiomatous meningiomas as well as meningiomas with angiomatous features that fulfilled histologic criteria for grade II.

High-grade meningiomas in our cohort were also significantly more likely to exhibit a paravenous origin (including parasagittal, falcine, torcula, and intraventricular locations), and significantly less likely to originate in the anterior skull base (including olfactory groove, clinoid, planum, tuberculum), than low-grade meningioma (*p* < 3 × 10^7^).

Across the 702 aggregated samples, low-grade meningiomas were significantly more likely to occur in females compared with males, in line with previous reports. We also validated the association between *AKT1/PIK3CA* mutations and meningothelial subtype (*p* < 0.001), *NF2* mutations with fibroblastic subtype (*p* < 0.001), and mutations in *TRAF7/KLF4* and secretory subtype (*p* < 0.001). We did not detect additional associations between age or gender and chromosomal disruption, mutation burden, location, or specific driver alterations.

Furthermore, we did not detect a significant difference in recurrence rates between high-grade meningiomas with canonical low-grade driver alterations and those without, or between *NF2*-mutant and *NF2*-wild-type low-grade meningiomas. However, we had limited power to assess such relationships as a result of the relatively low recurrence rate observed in our cohort, in part due to our limited follow-up time.

### Genomic basis for therapeutic options in high-grade meningiomas

We next assessed the percentage of high-grade tumors with potentially targetable alterations. We first considered the significantly recurrent genetic alterations that are targets of existing clinical therapeutics (*AKT1*, *SMO*, or *PIK3CA*). Relative to low-grade meningiomas, fewer high-grade meningiomas had mutations in these genes (17 vs. 5%; *p* < 8 × 10^−5^). We did observe isolated mutations in other members of these pathways in our high-grade extension cohort (Fig. 2e). However, their rates of mutation were also low (8.5% at most) and did not reach statistical significance.

**Fig. 6:**
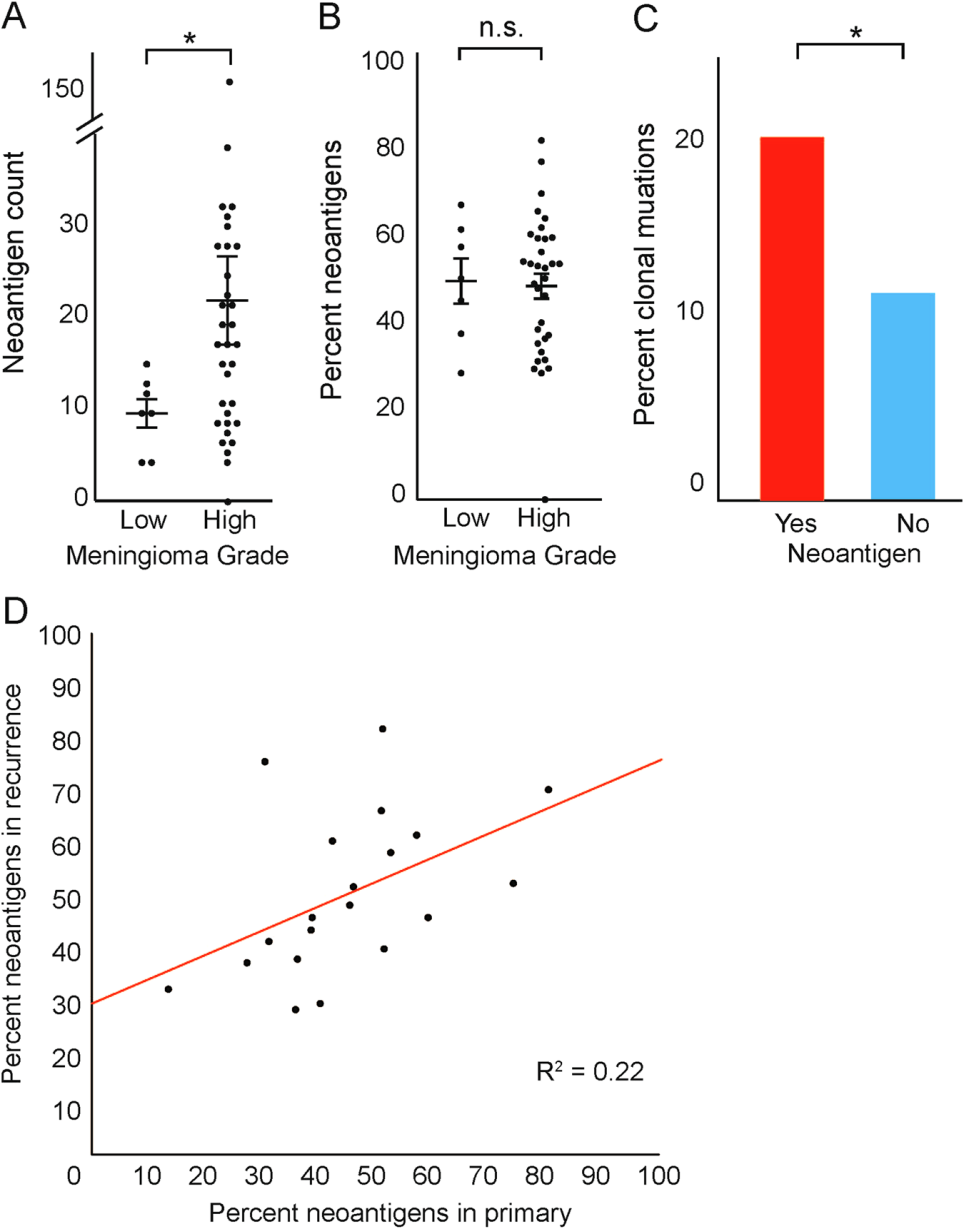
Analysis of predicted neoantigen load in meningioma. **a** Number of predicted neoantigens (*y*-axis) in low-grade and high-grade meningioma (*x*-axis). **b** Percentage of identified neoantigens (*y*-axis) in low-grade and high-grade meningioma (*x*-axis). **c** Percentage of mutations which are present in all tumor cells (*y*-axis) stratified by whether they are predicted to be immunogenic (*x*-axis). **d** Percentage of mutations in the primary (*x*-axis) which are predicted to be neoantigens vs. percentage of mutations in matched recurrence (*y*-axis) which are predicted to be neoantigens. *Error bars* and *central values* represent mean with s.e.m

We then characterized the putative neoantigen burden of high-grade meningiomas using a cancer immunogenomics approach, which has been shown to predict response to immunotherapy in melanoma,^32^ colorectal cancer,^33^ lung cancer,^34^ and in small subsets of brain tumor patients.^35, 36^ We used WES data to impute the human leukocyte antigen (HLA) type of each patient to predict which mutations could represent candidate neoantigens (Supplementary Table 8; see “Methods”). Across our high-grade cohort, 66% of mutations were predicted to be neoantigenic, and each sample had on average 87 such mutations (range 2–587). This represents a significant increase in putative neoantigen load compared with low-grade meningioma (mean = 8.4, *p* = 0.03; Fig. 6a) and was due primarily to the increased mutation burden of high-grade tumors, rather than to specific mutational processes that produced an increased rate of potentially immunogenic alterations. The fraction of mutations that could serve as neoantigens did not differ between low-grade and high-grade meningiomas (Fig. 6b).

To determine the relevance of these proposed neoantigens, we examined previously published expression data from a cohort of meningiomas.^37^ We calculated the overlap between genes with putative neoantigens in our cohort with those that were expressed in this prior cohort. We found that 76% of the genes with proposed neoantigens were expressed.

We next sought to determine whether these predicted neo-epitopes could represent robust substrates for immune targeting. Recent work has demonstrated that the presence of clonal neoantigens is correlated with overall survival.^38^ Interestingly, mutations predicted to be neoantigenic were more likely to be clonal than other mutations (20 vs. 11%, *p* < 0.001; Fig. 6c). Indeed, each sample had an average of 10 clonal mutations (range 1–71), suggesting that faithful markers that distinguish tumor cells from normal cells may exist for the majority of meningioma.

To further elucidate the potential for immune targeting of these neoantigens, we examined our cohort of serial recurrences in which we had shown that the specific mutations vary significantly from recurrence to recurrence in meningioma. Consistent with the lack of significantly recurrent mutations across patients, we found that relatively few of these putative neoantigens (2%) were identified in samples from more than one patient. However, the overall predicted neoantigen burden was relatively constant across samples from the same patient (Fig. 6d), demonstrating that even with variation of specific immunogenic alterations, the relative incidence of targetable alterations remains stable from sample to sample.

## Discussion

We performed next-generation sequencing on nearly 140 high-grade meningiomas, and found that they harbored elevated rates of mutations and copy number alterations compared with low-grade samples. Furthermore, the relative importance of specific driver alterations identified were markedly different across grades. Our data and previous publications support a model of meningioma formation in which PI3-kinase and Hedgehog pathway alterations are mostly restricted to low-grade tumors, *NF2* mutations and genomic disruption are enriched among high-grade tumors, and mutations of chromatin modifiers are observed across grades.^6, 7, 9, 10^

The major consistent genetic distinction between high-grade (grade II–III) and low-grade (grade I) meningiomas is the presence of widespread genomic disruption in aggressive meningiomas. Moreover, low-grade meningiomas which progressed to a higher grade on recurrence harbored disrupted copy number profiles that closely resembled their high-grade counterparts. Molecular classification of tumors has shown great promise for risk stratification across a variety of cancer types. We have previously shown that the degree of genomic disruption predicts subsequent recurrence in atypical meningioma.^39^ In our present study, we extend this analysis to low-grade tumors. The ability to differentiate the recurrence risk of meningioma by their copy number profiles may help augment traditional histopathological recurrence risk assessment, with consequent impact on management decisions.

When present, *NF2* alterations are believed to be the initiating event in meningiomagenesis, in part because germline alterations in *NF2* cause Neurofibromatosis 2, characterized by frequent meningiomas and schwannomas. However, genomic disruption tends to occur earlier in tumor evolution than do most mutations, and in one case, we observed three distinct *NF2* alterations across multiple recurrences in the setting of an identical copy number profile (Supplementary Fig. 6C). These findings suggest a model wherein high-grade meningiomas are initiated by widespread genomic disruption, followed by expansion of cells that then acquire *NF2* and other mutations. Further investigation will be necessary to determine whether widespread copy number alterations, or *NF2* mutations, tend to be earlier events in sporadic high-grade meningiomas.

Of the widespread genomic disruption observed in high-grade meningioma, the single most frequent event is arm-level loss of chromosome 22, which is postulated to drive bi-allelic inactivation of *NF2*. In line with this hypothesis, we found that mutations in *NF2* occur in 75% of meningiomas with loss of chromosome 22, but in less than 1% of samples without such loss. It is possible that the 25% of meningiomas with chromosome 22 loss that do not exhibit *NF2* mutations nevertheless contain cryptic *NF2*-inactivating events. Indeed, among meningiomas that had undergone WGS, every meningioma with loss of chromosome 22 exhibited inactivation of *NF2*. In four cases (27%), this inactivation was due to truncating rearrangements that may not be detectable by targeted sequencing.

However, it is also possible that chromosome 22 loss targets additional tumor suppressors. In principle, if inactivation of the second copy of *NF2* was the sole driver of chromosome 22 loss, then this could be accomplished by either arm-level losses, or through focal loss of the area surrounding *NF2*—but we observed no focal losses of *NF2* in any of the 56 tumors for which we had copy number profiling data. The possibility that chromosome 22 loss provides selective benefit in addition to loss of *NF2* is also supported by patterns of non-*NF2* driver mutations. Among tumors with chromosome 22 loss, we detected non-*NF2* driver mutations at a higher rate in meningiomas without *NF2* mutations than in meningiomas with *NF2* mutations, suggesting that *NF2* was not equivalently inactivated in the two groups. Several meningioma tumor suppressors on chromosome 22 have been proposed, including *SMARCB1*, *CHEK2*, and *CLH22*.^40, 41^ *CHEK2* halts cell-cycle progression in the presence of DNA damage, so loss of *CHEK2* would likely be associated with increased genomic disruption. Indeed, we observed increased genomic disruption among meningiomas with loss of chromosome 22.

The relative paucity of mutations in the non-*NF2* driver genes among high-grade tumors has implications for clinical care. mTOR pathway inhibitors are under clinical trial in recurrent and progressive meningiomas with *AKT1* or *PI3K* pathway alterations. The low incidence of such alterations in high-grade tumors means that enrollment in such trials on the basis of these mutations will require large multicenter trials. We identified mutations in additional, previously unreported pathway members of these genes, but they did not reach statistical significance. Larger cohorts which are powered for detection of low-frequency events will be necessary to determine whether these mutations are also drivers of meningiomagenesis and therefore may represent alternative avenues of therapeutic potential.

Both angiomatous and rhabdoid meningiomas present distinct copy number profiles compared with other histologic subtypes. Angiomatous meningiomas are typically considered to be grade I, although emerging data suggest the existence of grade II meningiomas with angiomatous features based on the presence of copy number changes such as monosomy 14 that are traditionally found in higher grade meningiomas. Independent of histologic grade, we observe frequent gains across the genome in multiple chromosomes in angiomatous meningiomas. Rhabdoid meningiomas are considered to be grade III tumors by WHO guidelines,^42^ but recent reports have proposed that the histolopathologic finding of rhabdoid features may be found in meningiomas of each of the three WHO grades. In our cohort, rhabdoid meningiomas harbored loss of chromosome 1p significantly less frequently than grade II–III meningiomas of other histologic subtypes. These observations support a role for the incorporation of genetic criteria in meningioma classification.

Recent work has also demonstrated that the molecular classification of meningioma extends beyond the genome.^43^ An integrated genomic analysis of grade II atypical meningiomas found that these tumors harbored distinct patterns of both DNA methylation and RNA expression compared with grade I tumors. This may contribute to the variable clinical outcomes that result from tumors with similar genomic profiles. Interestingly, copy number alterations appear to drive some, but not all, of these changes, implying that the mechanistic trigger for these differential cell states remains to be elucidated.

The appropriate classification, biological course, and adjuvant treatment options for radiation-induced meningiomas remains a challenge for clinicians. We found that the copy number burden of radiation-induced meningiomas is similar to radiation-naïve meningiomas, and is significantly lower than tumors previously exposed to therapeutic adjuvant radiation. This suggests that the effects of radiation on the molecular makeup of meningioma may be context-dependent and dose-dependent. Longitudinal follow-up will be necessary to determine if the levels of genomic disruption correspond to recurrence risk in radiation-induced meningiomas.

We observed no clear statistical evidence for clonal outgrowth between recurrences. In other cancer types, resistance to targeted inhibitors often result from pre-existing clones harboring resistance mutations in a background of additional mutations, and these clones rise to prominence in the population following treatment. The random phylogenetic ordering of multiple recurrences implies that the surgery and radiation applied to these tumors do not exert similarly strong bottlenecks on the tumor population. In time, as targeted and other systemic therapies become available for high-grade meningiomas, this pattern of clonal outgrowth may be altered.

Finally, we present the first analysis of predicted neoantigen load in meningioma. We identified a number of mutations in each meningioma that were predicted to be immunogenic. Although true credentialing of these neoantigens will require paired expression data and further follow-up, our work provides a proof-of-concept that a cancer immunogenomics approach to these tumors may be promising. Previous work has demonstrated that high-grade meningiomas express PD-L1 and harbor exuberant immune infiltrates.^44^ Clinical trials are currently enrolling for patients with recurrent high-grade meningioma to test the applicability of immunotherapy. Our finding that a significant fraction of mutations observed in high-grade meningioma is likely to result in neo-epitope presentation provides additional evidence for this line of therapeutic investigation. In particular, even if the cumulative neoantigen burden does not predict a high response to treatments such as checkpoint blockade immunotherapies, clonal neoantigens may be robust vaccine or adoptive cellular therapy targets. As we did not detect recurrent immunogenic targets, these data would likely inform the design of personalized cancer vaccines, rather than vaccines targeting shared tumor antigens. These particular efforts are currently being developed across a number of cancer types, and therefore this approach is conceivable for challenging meningiomas as well.^45^ Ongoing work to determine the applicability of such immunotherapy approaches will hopefully expand our therapeutic options for patients with these challenging tumors who currently have no reliable alternatives to surgery and radiation.

## Methods

### Sample identification

This study was reviewed and approved by the institutional review boards of the Dana-Farber/Harvard Cancer Center, the Brigham and Women’s Hospital, and the Broad Institute. Histopathologic diagnosis based on WHO 2007 criteria and tumor purity >80% was confirmed in all samples selected for study by two board-certified neuropathologists (S.S., M.A.). DNA was extracted from fresh-frozen tissue shavings or FFPE cores for tumor and paired blood buffy coat preparations or saliva for normal control DNA using standard protocols (Qiagen, Valencia, CA) and quantified using the PicoGreen system (Invitrogen). The tumor-normal pairs were confirmed by mass spectrometric genotypying with an established 48-SNP panel (Sequenom, San Diego, CA).

### Next-generation sequencing

A total of nine meningiomas underwent WGS, and 57 meningiomas WES, along with DNA from matched blood or saliva, as previously described.^6^ DNA was sonicated to 250 bp fragments, size selected with Agencourt AMPure XP beads, and ligated to specific barcoded adapters (Illumina TruSeq; Illumina Inc., San Diego, CA) for multiplexed analysis. Exome hybrid capture was performed on 76 meningiomas using the Agilent SureSelect hybrid capture kit (Whole Exome_v4; Agilent Technologies, Santa Clara, CA) and sequenced on a HiSeq 2500 system (Illumina Inc., San Diego, CA). All samples achieved at least 80× coverage in exons (mean coverage = 108×).

Sequence data were aligned to the hg19 (b37) reference sequence using the Burrows-Wheeler Aligner. Sample reads were sorted, duplicate-marked, and indexed using SAMtools and Picard. Bias in base quality score assignments due to flowcell, lane, dinucleotide context, and machine cycle were analyzed and recalibrated, and local realignment around insertions or deletions (indels) was achieved using the Genome Analysis Toolkit. All paired samples underwent quality control testing to ensure accuracy of tumor-normal pairs.

### Mutation analyses

Somatic mutations, insertions, and deletions were detected using MuTect and IndelLocator.^46^ These were annotated to genes and compared with events in the Catalogue of Somatic Mutations in Cancer (COSMIC) using Oncotator and also verified through visualization in Integrated Genome Viewer. Mutations with allelic fractions less than 0.1 were discarded for the purposes of driver mutation analysis. Significance of identified genetic alterations was assayed using MutSig2CV, which uses patient and gene-specific mutation rates to estimate a background model of predicted mutation incidence across the genome.^16^ It then factors in biological co-variates such as replication timing and gene-expression level on a gene-by-gene basis to account for the increased mutational rate of certain classes of genes.

To reduce the false positive rate of mutations in our validation cohort, samples without normal DNA were passed through a panel of normals filter. We removed all mutations present in either the ExAC or ESP databases.^47, 48^ To determine our power to detect driver mutations, we used a binomial distribution to calculate the true mutation rate which would produce either a 95 or 50% likelihood of success for seeing at least 15 mutations.

To calculate cohort-wide statistics, we excluded samples derived from the same patient. In the cases in which both whole genome and WES samples were available for the same patient, we included the whole genome sequenced sample; otherwise, we included the earliest available sample in our analysis (Supplementary Table 1).

### Copy number analyses

To analyze SCNAs from whole exome data, we used ReCapseg, which assesses homolog-specific copy ratios from segmental estimates of multipoint allelic copy ratios at heterozygous loci incorporating the statistical phasing software (BEAGLE) and population haplotype panels (HAPMAP3).^49, 50^ Allele-specific SCNAs and tumor ploidy status were assessed with ABSOLUTE.^51^ For copy number alteration significance analysis, segmented copy number data were analyzed by GISTIC 2.0, which separately assesses the significance of recurrent focal and arm-level SCNAs by comparing their rates of alteration to the overall genome-wide alteration rate. In the case of arm-level events, it controls for the tendency for short arms to undergo more frequent alterations.^52^

Calculations which compared degree of chromosomal disruption across groups of samples did not include angiomatous meningioma, due to their unique genomic profile characterized largely by gains.

### Rearrangements analyses

Rearrangement detection was performed using Snowman. Snowman performs genome-wide unbiased local assembly with SGA and realigns contigs to the reference using BWA-MEM.^53^ Aligned contigs with multi-part or gapped alignments indicate candidate structural variants. Reads are re-aligned to the contigs to score candidate variants and to classify events as somatic or germline. Microhomology at the breakpoints was determined by recording the number of bases at the junction that could be aligned to either side of the breakpoint. Event types were determined based on interpretation of read coverage plots paired with called rearrangements. Classification of the mechanistic basis of rearrangements was adapted from Yang et al.^22^

### Phylogenetic analyses

To determine the percentage of mutations shared by a typical pair of samples from the same patient, we calculated the overlap of shared mutations from all possible pairs of samples for each patient, then took the per-patient average. CCFs were determined using ABSOLUTE, which combines copy number data with mutations to estimate purity and ploidy of samples. Phylogenetic trees were constructed by looking at mutations with 95% power of detection across all samples from each patient, then determining the phylogenetic relationship based on shared overlap. Copy number alterations were assessed separately.

Spatial heterogeneity in the validation cohort was calculated as the percentage of mutations identified in our current cohort that were also identified in the earlier targeted sequencing. We excluded genes that were not covered in the earlier gene lists. Genomic position was inferred from protein change using TransVar (http://bioinformatics.mdanderson.org/transvarweb).^54^

### Mutational immunogenicity prediction

HLA alleles were called with PHLAT from exome sequencing data.^55^ For each tumor, epitope predictions were made by considering interaction between confidently called HLA alleles and single-residue missense alterations (SNV) or protein-altering indel alterations. Separate lists were generated consisting of wild-type and mutant peptides of 8, 9, 10, and 11 amino acids in length for MHC Class I and 15 amino acids in length for MHC Class II, as these are known to be the possible lengths for peptides presented by human MHC.

We then predicted MHC binding affinity for each of the peptides as described previously.^56^ We used the NetMHC, NetMHCpan, SMM, SMMPMBEC, and NetMHCIIpan prediction methods to predict MHC binding affinity values for each peptide and used the median value across all algorithms as a composite measure of binding strength.^57–61^ We also defined the neoepitope ratio for each mutant and wild-type peptide pair as the median affinity value for the mutant peptide divided by the median affinity value for the wild-type peptide. This value was found to be a reliable comparator of the relative immunogenicities of the mutant vs. wild-type peptide sequences.^56^ Because epitope binding is HLA dependent, the previous steps were performed for each of the called MHC I proteins. After this, only peptides predicted to be the best epitopes for each mutation were considered.

### Gene expression analysis

Published data were downloaded from GEO (https://www.ncbi.nlm.nih.gov/geo/) from accession GSE77259.^37^ Data were imported into GenePattern,^62^ then normalized using the AffySTExpressionFileCreator module, which implements the oligo package.^63^ The mean for each gene was calculated across samples, and the bottom 25% expressing genes were removed. We then calculated the percent overlap between genes with putative neoantigens, and genes that were expressed.

### Statistical analyses

All statistical analyses were performed using version 3.1 of the open source R software package.^64^ The Student’s *t*-test and Wilcox log-rank tests were used to assess differences in means. The Fisher’s exact test was used to determine differences in count data. A binomial null distribution was used to determine statistical power. All tests were two-sided. Non-sparse data without significant outliers were assumed to be normal; sparse data or those with significant outliers were evaluated using non-parametric tests.

### Availability of data and material

All sequencing data have been deposited in the EGA repository at https://www.ebi.ac.uk/ega/studies/EGAS00001002294. All code used for analysis is available at https://github.com/ngreenwald/Publications/tree/master/High_Grade_Meningioma.

## Electronic supplementary material

Supplementary Figures and Legends

Supplementary Table 1

Supplementary Table 2

Supplementary Table 3

Supplementary Table 4

Supplementary Table 5

Supplementary Table 6 and 7

Supplementary Table 8

## References

[1] Ostrom QT (2015). CBTRUS statistical report: primary brain and central nervous system tumors diagnosed in the United States in 2008-2012. Neuro. Oncol..

[2] Louis, D., Ohgaki, H., Wiestler, O. D. & Cavenee, W. K. *WHO Classification of Tumours of the Central Nervous System*, 408 (International Agency for Research on Cancer, 2016).

[3] Wen PY (2010). Medical therapies for meningiomas. J. Neurooncol..

[4] Rouleau GA (1993). Alteration in a new gene encoding a putative membrane-organizing protein causes neuro-fibromatosis type 2. Nature.

[5] Ruttledge MH (1994). Evidence for the complete inactivation of the NF2 gene in the majority of sporadic meningiomas. Nat. Genet..

[6] Brastianos PK (2013). Genomic sequencing of meningiomas identifies oncogenic SMO and AKT1 mutations. Nat. Genet..

[7] Clark VE (2013). Genomic analysis of non-NF2 meningiomas reveals mutations in TRAF7, KLF4, AKT1, and SMO. Science.

[8] Goutagny S (2014). High incidence of activating TERT promoter mutations in meningiomas undergoing malignant progression. Brain Pathol..

[9] Abedalthagafi M (2016). Oncogenic PI3K mutations are as common as AKT1 and SMO mutations in meningioma. Neuro. Oncol..

[10] Clark VE (2016). Recurrent somatic mutations in POLR2A define a distinct subset of meningiomas. Nat. Genet..

[11] Shankar, G. M. et al. Germline and somatic BAP1 mutations in high-grade rhabdoid meningiomas. *Neuro. Oncol*. (2016) [Epub ahead of print]. 10.1093/neuonc/now235 PMC5464371 28170043

[12] Abedalthagafi, M. B. et al. ARID1A and TERT promoter mutations in dedifferentiated meningioma. *Cancer Genet* **208**, 345–350 (2015). 10.1016/j.cancergen.2015.03.005 PMC4882906 25963524

[13] Brastianos PK (2014). Exome sequencing identifies BRAF mutations in papillary craniopharyngiomas. Nat. Genet..

[14] Alexandrov LB (2013). Signatures of mutational processes in human cancer. Nature.

[15] Haradhvala NJ (2016). Mutational strand asymmetries in cancer genomes reveal mechanisms of DNA damage and repair. Cell.

[16] Lawrence MS (2013). Mutational heterogeneity in cancer and the search for new cancer-associated genes. Nature.

[17] Zack TI (2013). Pan-cancer patterns of somatic copy number alteration. Nat. Genet..

[18] Bi WL (2016). Genomic landscape of intracranial meningiomas. J. Neurosurg..

[19] Forment JV (2012). Chromothripsis and cancer: causes and consequences of chromosome shattering. Nat. Rev. Cancer.

[20] Baca SC (2013). Punctuated evolution of prostate cancer genomes. Cell.

[21] Forbes SA (2015). COSMIC: exploring the world’s knowledge of somatic mutations in human cancer. Nucleic Acids Res..

[22] Yang L (2013). Diverse mechanisms of somatic structural variations in human cancer genomes. Cell.

[23] Gerlinger M (2014). Genomic architecture and evolution of clear cell renal cell carcinomas defined by multiregion sequencing. Nat. Genet..

[24] Eleveld TF (2015). Relapsed neuroblastomas show frequent RAS-MAPK pathway mutations. Nat. Genet..

[25] Lim B (2015). Genome-wide mutation profiles of colorectal tumors and associated liver metastases at the exome and transcriptome levels. Oncotarget.

[26] Bai H (2016). Integrated genomic characterization of IDH1-mutant glioma malignant progression. Nat. Genet..

[27] Gibson WJ (2016). The genomic landscape and evolution of endometrial carcinoma progression and abdominopelvic metastasis. Nat. Genet..

[28] Kumar A (2016). Substantial interindividual and limited intraindividual genomic diversity among tumors from men with metastatic prostate cancer. Nat. Med..

[29] Xue R (2016). Variable intra-tumor genomic heterogeneity of multiple lesions in patients with hepatocellular carcinoma. Gastroenterology.

[30] Simon M (1996). Role of genomic instability in meningioma progression. Genes Chromosomes Cancer.

[31] Abedalthagafi, M. S. et al. Angiomatous meningiomas have a distinct genetic profile with multiple chromosomal polysomies including polysomy of chromosome 5. *Oncotarget* **5**, 10596–10606 (2014). 10.18632/oncotarget.2517 PMC4279396 25347344

[32] Van Allen EM (2015). Genomic correlates of response to CTLA-4 blockade in metastatic melanoma. Science.

[33] Le DT (2015). PD-1 blockade in tumors with mismatch-repair deficiency. N. Engl. J. Med..

[34] Rizvi NA (2015). Cancer immunology. Mutational landscape determines sensitivity to PD-1 blockade in non-small cell lung cancer. Science.

[35] Bouffet E (2016). Immune checkpoint inhibition for hypermutant glioblastoma multiforme resulting from germline biallelic mismatch repair deficiency. J. Clin. Oncol..

[36] Johanns TM (2016). Immunogenomics of hypermutated glioblastoma: a patient with germline POLE deficiency treated with checkpoint blockade immunotherapy. Cancer Discov..

[37] Schulten HJ (2016). Microarray expression data identify DCC as a candidate gene for early meningioma progression. PLoS ONE.

[38] McGranahan N (2016). Clonal neoantigens elicit T cell immunoreactivity and sensitivity to immune checkpoint blockade. Science.

[39] Aizer AA (2016). A prognostic cytogenetic scoring system to guide the adjuvant management of patients with atypical meningioma. Neuro. Oncol..

[40] Schmitz U (2001). INI1 mutations in meningiomas at a potential hotspot in exon 9. Br. J. Cancer.

[41] Yang HW (2012). Alternative splicing of CHEK2 and codeletion with NF2 promote chromosomal instability in meningioma. Neoplasia.

[42] Vaubel RA (2016). Meningiomas with rhabdoid features lacking other histologic features of malignancy: a study of 44 cases and review of the literature. J. Neuropathol. Exp. Neurol..

[43] Harmanci AS (2017). Integrated genomic analyses of de novo pathways underlying atypical meningiomas. Nat. Commun..

[44] Du, Z. et al. Increased expression of the immune modulatory molecule PD-L1 (CD274) in anaplastic meningioma. *Oncotarget* **6**, 4704–4716 (2014). 10.18632/oncotarget.3082 PMC4467109 25609200

[45] Johanns, T. M. & Dunn, G. P. Applied cancer immunogenomics: leveraging neoantigen discovery in glioblastoma. *Cancer J*. **23**, 125–130 (2017). 10.1097/PPO.0000000000000247 PMC5605294 28410300

[46] Cibulskis K (2013). Sensitive detection of somatic point mutations in impure and heterogeneous cancer samples. Nat. Biotechnol..

[47] Exome Variant Server, NHLBI GO Exome Sequencing Project (ESP), Seattle, WA http://evs.gs.washington.edu/EVS/. (September 2015)

[48] Lek M (2016). Analysis of protein-coding genetic variation in 60,706 humans. Nature.

[49] Browning BL, Yu Z (2009). Simultaneous genotype calling and haplotype phasing improves genotype accuracy and reduces false-positive associations for genome-wide association studies. Am. J. Hum. Genet..

[50] International HapMap C (2010). Integrating common and rare genetic variation in diverse human populations. Nature.

[51] Carter SL (2012). Absolute quantification of somatic DNA alterations in human cancer. Nat. Biotechnol..

[52] Beroukhim R (2010). The landscape of somatic copy-number alteration across human cancers. Nature.

[53] Simpson JT, Durbin R (2012). Efficient de novo assembly of large genomes using compressed data structures. Genome Res..

[54] Zhou W (2015). TransVar: a multilevel variant annotator for precision genomics. Nat. Methods.

[55] Bai Y, Ni M, Cooper B, Wei Y, Fury W (2014). Inference of high resolution HLA types using genome-wide RNA or DNA sequencing reads. BMC Genomics.

[56] Gubin MM (2014). Checkpoint blockade cancer immunotherapy targets tumour-specific mutant antigens. Nature.

[57] Nielsen M (2003). Reliable prediction of T-cell epitopes using neural networks with novel sequence representations. Protein Sci..

[58] Peters B, Sette A (2005). Generating quantitative models describing the sequence specificity of biological processes with the stabilized matrix method. BMC Bioinformatics.

[59] Hoof I (2009). NetMHCpan, a method for MHC class I binding prediction beyond humans. Immunogenetics.

[60] Kim Y, Sidney J, Pinilla C, Sette A, Peters B (2009). Derivation of an amino acid similarity matrix for peptide: MHC binding and its application as a Bayesian prior. BMC Bioinformatics.

[61] Karosiene E (2013). NetMHCIIpan-3.0, a common pan-specific MHC class II prediction method including all three human MHC class II isotypes, HLA-DR, HLA-DP and HLA-DQ. Immunogenetics.

[62] Reich M (2006). GenePattern 2.0. Nat. Genet..

[63] Carvalho BS, Irizarry RA (2010). A framework for oligonucleotide microarray preprocessing. Bioinformatics.

[64] R Development Core Team. *R: A Language and Environment for Statistical Computing*. Vienna, Austria: the R Foundation for Statistical Computing (2016).

