# Erratum: Genomic landscape of high-grade meningiomas

**DOI:** 10.1038/s41525-017-0023-6

**Published:** 2017-09-04

**Authors:** Wenya Linda Bi, Noah F. Greenwald, Malak Abedalthagafi, Jeremiah Wala, Will J. Gibson, Pankaj K. Agarwalla, Peleg Horowitz, Steven E. Schumacher, Ekaterina Esaulova, Yu Mei, Aaron Chevalier, Matthew A. Ducar, Aaron R. Thorner, Paul van Hummelen, Anat O. Stemmer-Rachamimov, Maksym Artyomov, Ossama Al-Mefty, Gavin P. Dunn, Sandro Santagata, Ian F. Dunn, Rameen Beroukhim

**Affiliations:** 1 Center for Skull Base and Pituitary Surgery, Department of Neurosurgery, Brigham and Women’s Hospital, Harvard Medical School, Boston, MA USA; 2 Department of Cancer Biology, Dana-Farber Cancer Institute, Boston, MA USA; 3 Broad Institute of MIT and Harvard, Cambridge, MA USA; 4 Division of Neuropathology, Department of Pathology, Brigham and Women’s Hospital, Boston, MA USA; 5 Research Center, King Fahad Medical City, Riyadh, Saudi Arabia; 6 The Saudi Human Genome Project Lab, King Abdulaziz City for Science and Technology, Riyadh, Saudi Arabia; 7 Department of Neurosurgery, Massachusetts General Hospital, Boston, MA USA; 8 Department of Surgery, The University of Chicago, Chicago, IL USA; 9 Department of Pathology and Immunology, Washington University School of Medicine, St. Louis, MO USA; 10 Computer Technologies Department, ITMO University, Saint Petersburg, Russia; 11 Center for Cancer Genome Discovery, Dana-Farber Cancer Institute, Boston, MA USA; 12 Department of Pathology, Massachusetts General Hospital, Boston, MA USA; 13 Department of Neurosurgery, Washington University School of Medicine, St. Louis, MO USA; 14 Center for Human Immunology and Immunotherapy Programs, Washington University School of Medicine, St. Louis, MO USA; 15 Department of Medical Oncology, Dana-Farber Cancer Institute, Boston, MA USA

**Erratum to:** *npj Genomic Medicine* (2017); doi:10.1038/s41525-017-0014-7; Published 26 April 2017

This article contained typographical errors within the Availability of data and material section:

All code used for analysis is available at https://github.com/ngreenwald/publications/Lab_Stuff/High_Grade_Meningioma.

Now reads:

All code used for analysis is available at https://github.com/ngreenwald/Publications/tree/master/High_Grade_Meningioma.

This error has now been corrected in the HTML and PDF versions of this Article.

