# Supplementary material for: Genomic landscape of high-grade meningiomas
Source: NPJ Genom Med. 2017 Apr 26;2:15. doi: 10.1038/s41525-017-0014-7 (PMC5506858; doi:10.1038/s41525-017-0014-7)
Supplement: Supplementary file 1 — Supplementary Figures and Legends [file 41525_2017_14_MOESM1_ESM.docx]

Supplementary Information

**Supplementary Note**

**Supplementary Note 1**

To assess tumor purity, we first examined the allelic fraction of *NF2* alterations, as mutations in this gene are thought to be an early driver event in a majority of meningiomas. The allelic fraction of a given mutation represents the percentage of sequenced reads from that location which contain the alteration; large amounts of contaminating normal DNA which do not contain the alteration would reduce the fraction of reads with the mutation. Among these samples, the average allelic fraction of *NF2* mutations was 60%, comparable to the cohort wide average for *NF2* of 59%, and well within detection limits. Analysis of both copy-number data and the allelic fractions of all mutations genome-wide indicates the multi-sampled meningiomas had an average purity of 70%.

**Supplementary Figures**


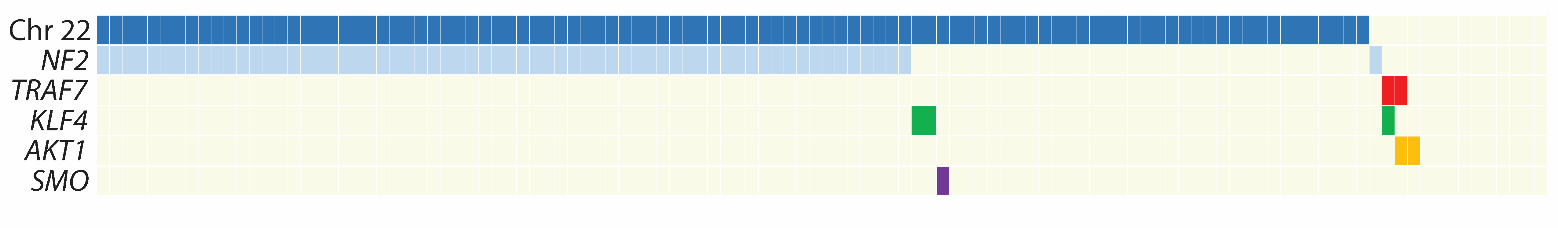


**Supplementary Figure 1.** Heatmap of driver mutations in newly-sequenced high-grade meningiomas.

**
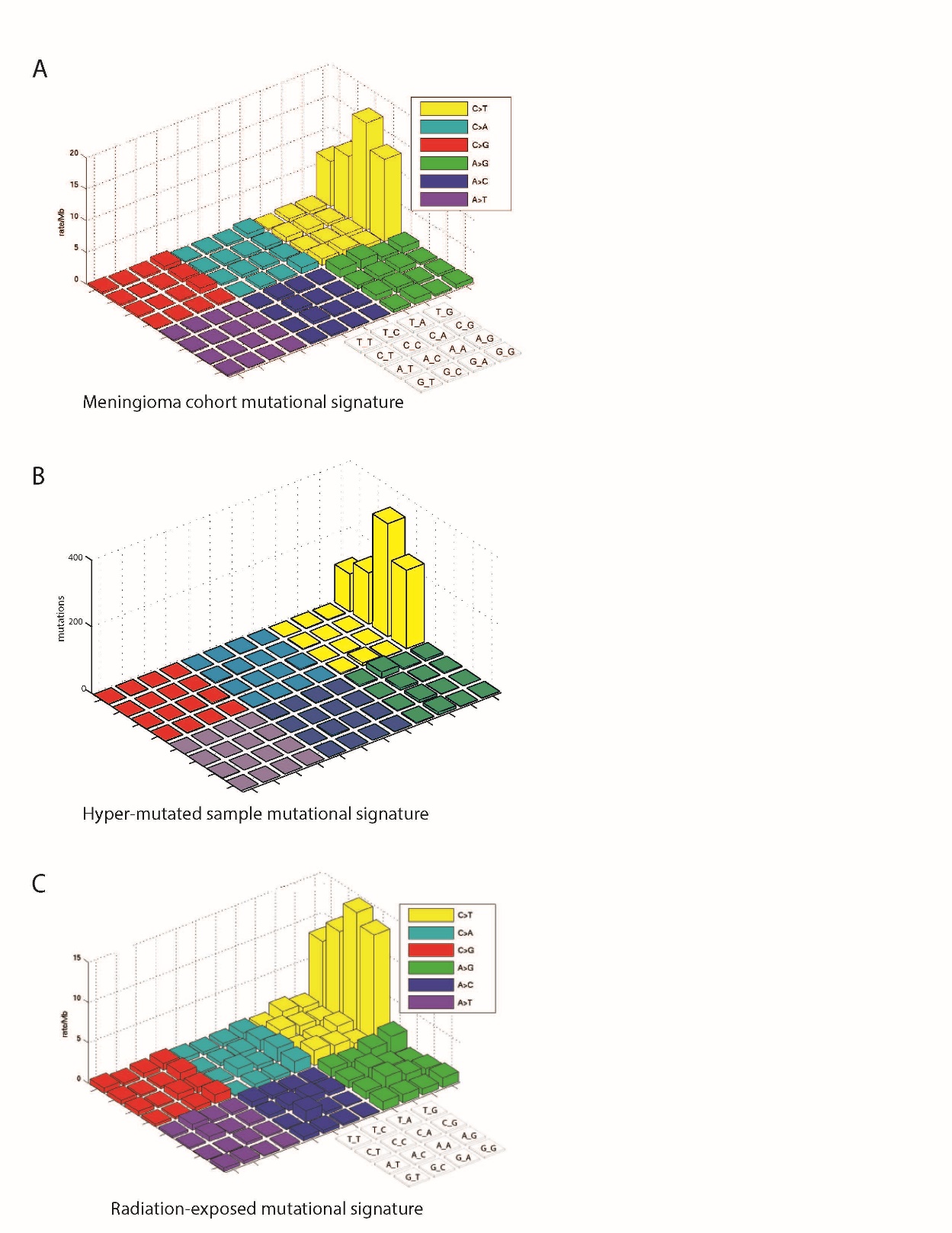
**

**Supplementary Figure 2.** Mutational patterns of meningioma. (A) Lego plot demonstrating the magnitude (z-axis) of different mutational signatures (x- and y-axis) in the high-grade meningioma cohort. (B) Lego plot demonstrating the magnitude (z-axis) of different mutational signatures (x- and y-axis) in a hyper-mutated meningioma sample (MEN0092). (C) Lego plot demonstrating the magnitude (z-axis) of different mutational signatures (x- and y-axis) across meningiomas exposed to prior radiation.

**
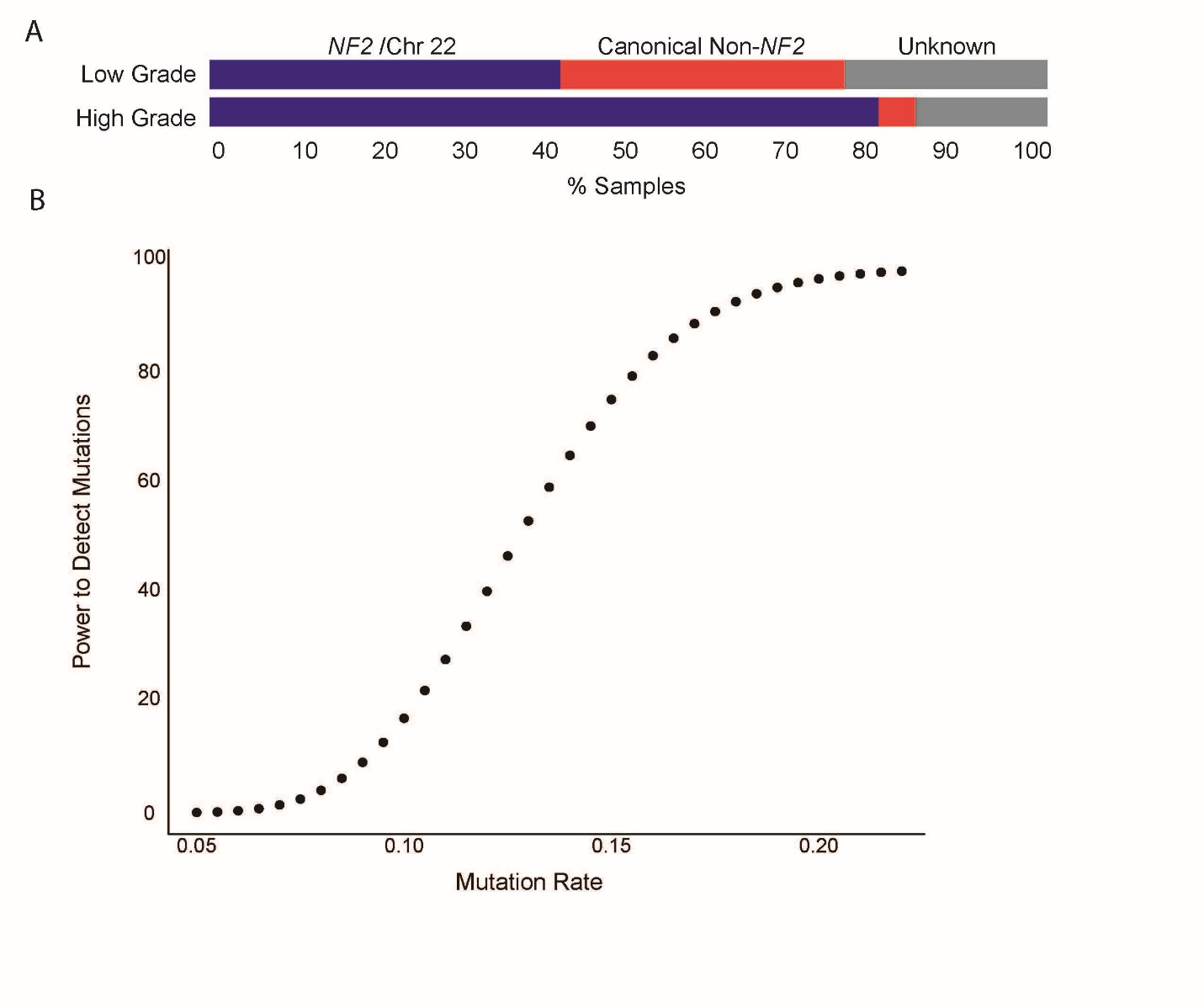
Supplementary Figure 3.** Mutational patterns in low- and high-grade meningioma. (A) Distribution of mutational drivers across low- and high-grade tumors. (B) Simulated data estimating the power to detect mutations (y-axis) as a function of the true mutation rate (x-axis).


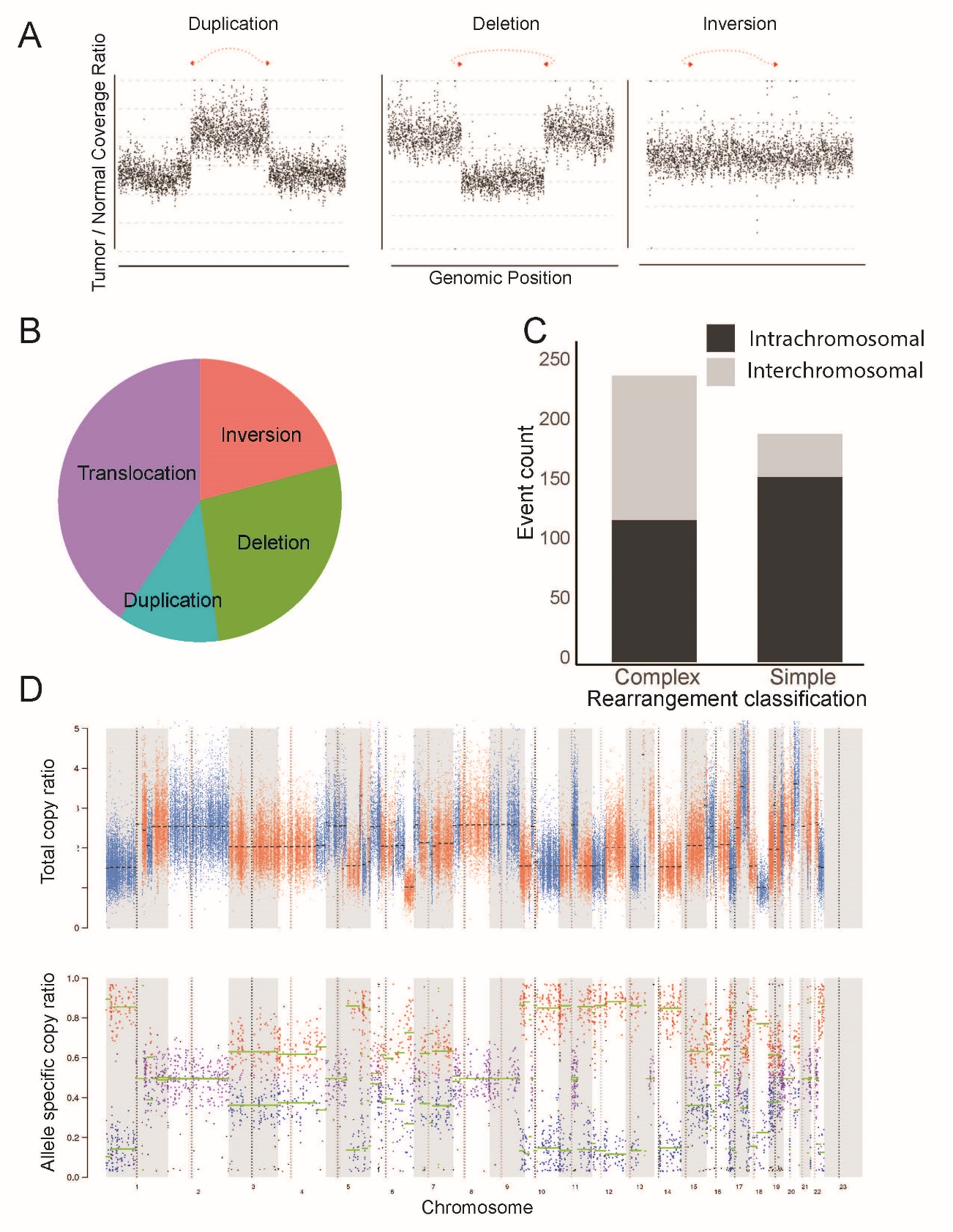


**Supplementary Figure 4.** Rearrangements in meningioma. (A) Sample plots demonstrating change in read coverage (y-axis) across the genome (x-axis) for duplications (left), deletions (center), or inversion (right). (B) Relative frequencies of event type (pie sectors) across the whole-genome sequenced meningioma cohort. (C) Comparison of number of rearrangements (y-axis) classified as complex or simple (x-axis) events, and stratified by intrachromosomal (black) versus interchromosomal (gray) status. (D) Copy number ratio (y-axis) across the genome (x-axis) for total copy ratio (top) and allele-specific copy ratio (bottom).

**
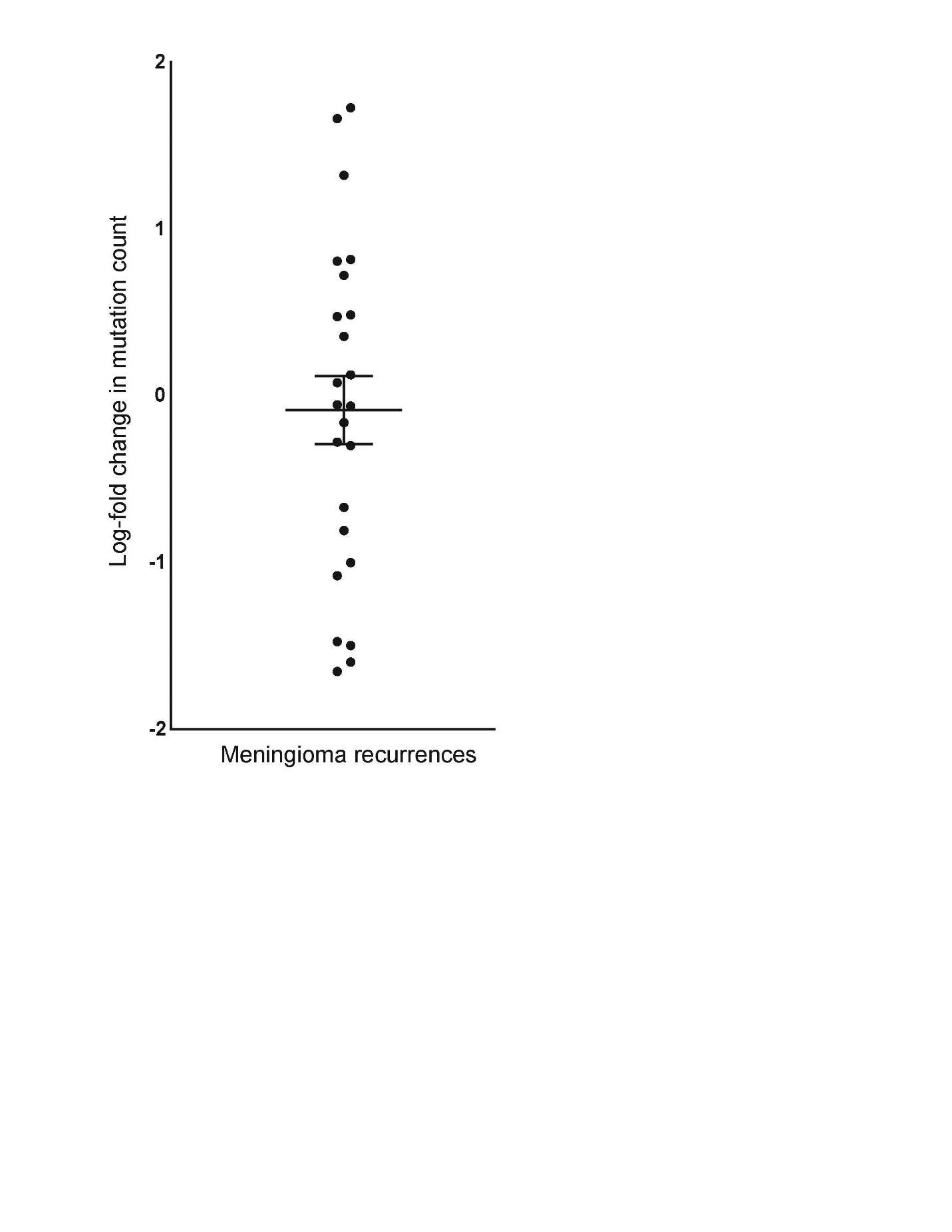
**

**Supplementary Figure 5.** Heterogeneity in meningioma recurrences. Log_2_ fold change in the total number of mutations (y-axis) from subsequent recurrences of the same patient (x-axis).

**
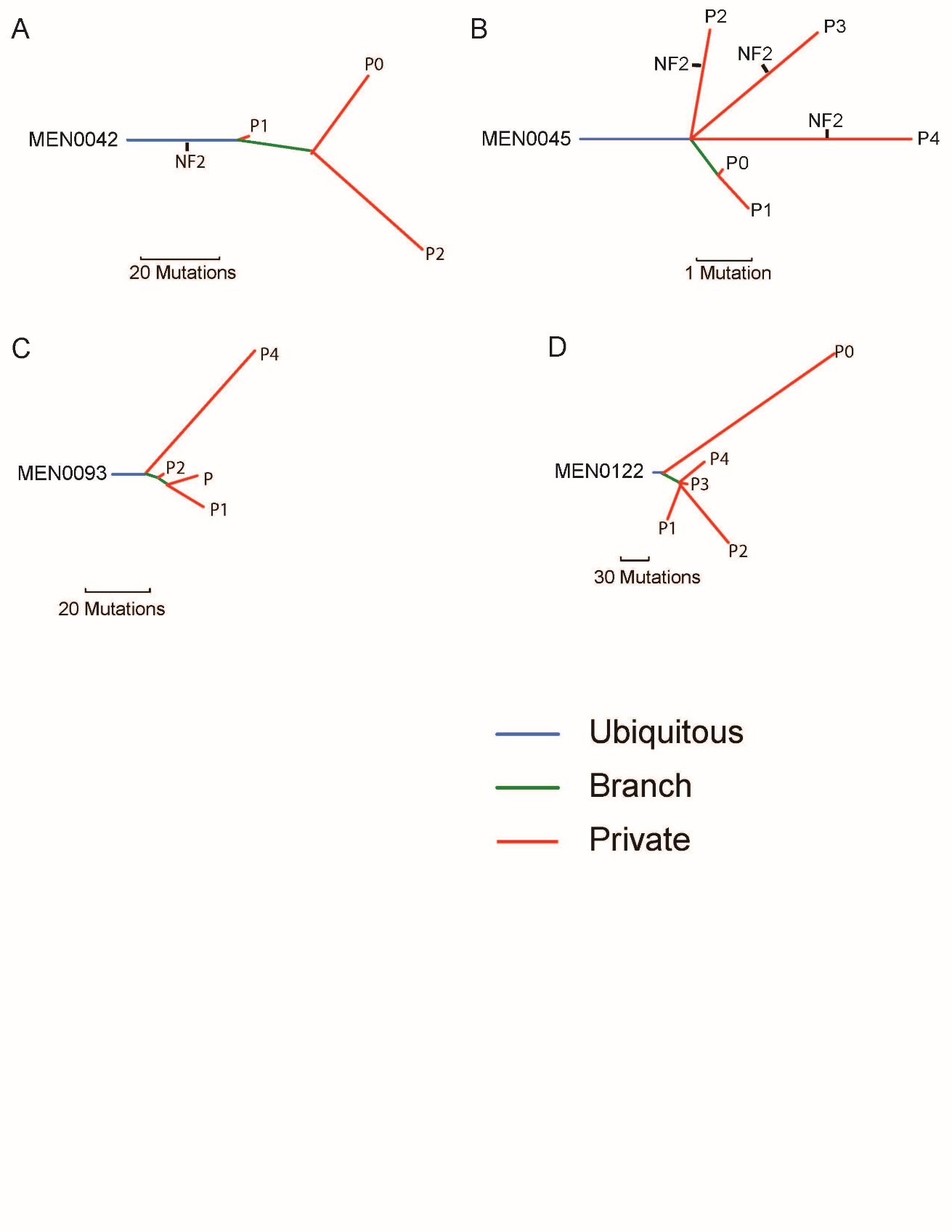
Supplementary Figure 6.** Temporal relationship between mutation status across meningioma recurrences. (A–D) Phylogenetic trees for patients with serial recurrences.

**Supplementary Table Legends**

**Supplementary Table 1**. Sample information of sequenced meningiomas. -, not available

**Supplementary Table 2**. Mutations identified from whole-exome or whole-genome sequencing.

**Supplementary Table 3**. Genes sequenced in targeted sequencing extension cohort.

**Supplementary Table 4**. Mutations identified from targeted capture sequencing.

**Supplementary Table 5**. (**A**) Rearrangements identified from whole-genome or whole-exome sequencing. (**B**) Genes with rearrangements in at least two samples.

**Supplementary Table 6.** Descriptive information for patients with multiple sequenced samples.

**Supplementary Table 7**. Analysis of spatial heterogeneity for samples with two separate FFPE cores of the same resection sequenced.

**Supplementary Table 8**. Predicted immunogenicity for mutations from whole-exome sequenced meningiomas.
