# Supplementary material for: Genomic landscape of high-grade meningiomas
Source: NPJ Genom Med. 2017 Apr 26;2:15. doi: 10.1038/s41525-017-0014-7 (PMC5506858; doi:10.1038/s41525-017-0014-7)
Supplement: Supplementary file 8 — Supplementary Table 8 [file 41525_2017_14_MOESM8_ESM.pdf]

Sample	Gene symbol	Variant	chr	start	end	ref	alt
MEN0030-P0	BRD7	indel	16	50368678	50368679	CT	-
MEN0030-P0	CACNA1E	indel	1	181680102	181680103	AG	-
MEN0030-P0	SRP14	indel	15	40328597	40328599	TGC	-
MEN0030-P0	TMPRSS6	indel	22	37466653	37466655	CCA	-
MEN0030-P0	SALL1	indel	16	51175655	51175655	-	GCT
MEN0030-P0	SCAF4	indel	21	33044256	33044256	-	GCTGCT
MEN0030-P0	SNAPC4	indel	9	139277995	139277997	GCT	-
MEN0030-P0	SRP14	indel	15	40328597	40328599	TGC	-
MEN0030-P0	CACNA1E	indel	1	181680102	181680103	AG	-
MEN0030-P0	BRD7	indel	16	50368678	50368679	CT	-
MEN0030-P0	TMPRSS6	indel	22	37466653	37466655	CCA	-
MEN0030-P0	SNAPC4	indel	9	139277995	139277997	GCT	-
MEN0030-P0	SALL1	indel	16	51175655	51175655	-	GCT
MEN0030-P0	SCAF4	indel	21	33044256	33044256	-	GCTGCT
MEN0030-P1	BRD7	indel	16	50368678	50368679	CT	-
MEN0030-P1	CACNA1E	indel	1	181680102	181680103	AG	-
MEN0030-P1	SRP14	indel	15	40328597	40328599	TGC	-
MEN0030-P1	TMPRSS6	indel	22	37466653	37466655	CCA	-
MEN0030-P1	SALL1	indel	16	51175655	51175655	-	GCT
MEN0030-P1	SCAF4	indel	21	33044256	33044256	-	GCTGCT
MEN0030-P1	SRP14	indel	15	40328597	40328599	TGC	-
MEN0030-P1	CACNA1E	indel	1	181680102	181680103	AG	-
MEN0030-P1	BRD7	indel	16	50368678	50368679	CT	-
MEN0030-P1	TMPRSS6	indel	22	37466653	37466655	CCA	-
MEN0030-P1	SALL1	indel	16	51175655	51175655	-	GCT
MEN0030-P1	SCAF4	indel	21	33044256	33044256	-	GCTGCT
MEN0042-P0	OTOP1	indel	4	4228274	4228282	CCACAGCA	-
MEN0042-P0	KMT2C	indel	7	151874148	151874148	T	-
MEN0042-P0	PLAT	indel	8	42038123	42038123	A	-
MEN0042-P0	CRIPAK	indel	4	1388498	1388501	CACA	-
MEN0042-P0	MED24	indel	17	38187423	38187423	A	-
MEN0042-P0	TPTE	indel	21	10944697	10944697	A	-
MEN0042-P0	SLAMF1	indel	1	160589601	160589601	T	-
MEN0042-P0	TGFBR2	indel	3	30691872	30691872	A	-
MEN0042-P0	EP400	indel	12	132547093	132547093	-	CAG
MEN0042-P0	BEGAIN	indel	14	101005271	101005273	CCT	-
MEN0042-P0	CDKN2C	indel	1	51439798	51439798	C	-
MEN0042-P0	KDM6A	indel	X	44922688	44922691	CAGG	-
MEN0042-P0	GOLGA8R	indel	15	30700168	30700168	-	TTG
MEN0042-P0	TPCN1	indel	12	113704096	113704098	CTG	-
MEN0042-P0	TNFRSF4	indel	1	1148074	1148074	G	-
MEN0042-P0	CEBPZ	indel	2	37454908	37454908	T	-
MEN0042-P0	SACS	indel	13	23914687	23914687	T	-
MEN0042-P0	BRD7	indel	16	50368678	50368679	CT	-
MEN0042-P0	AMT	indel	3	49459653	49459653	-	C
MEN0042-P0	CHD2	indel	15	93545433	93545433	-	A

MEN0042-P0	FSCB	indel	14	44974154	44974154	-	GGGGCCT(
MEN0042-P0	PLAC4	indel	21	42551272	42551272	C	-
MEN0042-P0	POM121	indel	7	72413723	72413723	-	CTC
MEN0042-P0	ABL1	indel	9	133759490	133759492	AAG	-
MEN0042-P0	PDILT	indel	16	20370700	20370702	CCA	-
MEN0042-P0	SLC25A5	indel	X	118603706	118603706	-	G
MEN0042-P0	INA	indel	10	105048271	105048273	GAG	-
MEN0042-P0	KRTAP4-1	indel	17	39340796	39340852	CGGCAGC/	-
MEN0042-P0	KANK1	indel	9	732475	732477	GAG	-
MEN0042-P0	TMEM63A	indel	1	226034840	226034842	CTG	-
MEN0042-P0	GAGE2D	indel	X	49208295	49208295	-	TAT
MEN0042-P0	ADAMTS7	indel	15	79058183	79058183	-	TGGGTCC
MEN0042-P0	CDC27	indel	17	45219612	45219612	A	-
MEN0042-P0	TERF2IP	indel	16	75690204	75690206	GAA	-
MEN0042-P0	ATXN1	indel	6	16327913	16327915	TGA	-
MEN0042-P0	SLC51A	indel	3	195955100	195955102	CTG	-
MEN0042-P0	CDC42EP1	indel	22	37964409	37964429	CAGCGCCT	-
MEN0042-P0	KRTAP4-5	indel	17	39305775	39305775	-	GGCAGCA(
MEN0042-P0	UBXN11	indel	1	26608878	26608883	CCGGGA	-
MEN0042-P0	CCNT1	indel	12	49087434	49087436	ATG	-
MEN0042-P0	KRTAP5-5	indel	11	1651199	1651199	-	GGCTGTG(
MEN0042-P0	FAM155A	indel	13	108518687	108518689	CTG	-
MEN0042-P0	CCAR1	indel	10	70509285	70509286	GA	-
MEN0042-P0	CD3EAP	indel	19	45911859	45911861	GAA	-
MEN0042-P0	CTNND2	indel	5	11110989	11110991	TTC	-
MEN0042-P0	CEBPZ	indel	2	37454908	37454908	T	-
MEN0042-P0	ABL1	indel	9	133759490	133759492	AAG	-
MEN0042-P0	SACS	indel	13	23914687	23914687	T	-
MEN0042-P0	CRIPAK	indel	4	1388498	1388501	CACA	-
MEN0042-P0	OTOP1	indel	4	4228274	4228282	CCACAGCA	-
MEN0042-P0	POM121	indel	7	72413723	72413723	-	CTC
MEN0042-P0	KMT2C	indel	7	151874148	151874148	T	-
MEN0042-P0	SLAMF1	indel	1	160589601	160589601	T	-
MEN0042-P0	TGFBR2	indel	3	30691872	30691872	A	-
MEN0042-P0	PLAT	indel	8	42038123	42038123	A	-
MEN0042-P0	EP400	indel	12	132445252	132445252	-	C
MEN0042-P0	GAGE2D	indel	X	49208295	49208295	-	TAT
MEN0042-P0	BRD7	indel	16	50368678	50368679	CT	-
MEN0042-P0	AMT	indel	3	49459653	49459653	-	C
MEN0042-P0	BEGAIN	indel	14	101005271	101005273	CCT	-
MEN0042-P0	TPCN1	indel	12	113704096	113704098	CTG	-
MEN0042-P0	MED24	indel	17	38187423	38187423	A	-
MEN0042-P0	PLAC4	indel	21	42551272	42551272	C	-
MEN0042-P0	CDC27	indel	17	45219612	45219612	A	-
MEN0042-P0	TNFRSF4	indel	1	1148074	1148074	G	-
MEN0042-P0	PDILT	indel	16	20370700	20370702	CCA	-
MEN0042-P0	KDM6A	indel	X	44922688	44922691	CAGG	-

MEN0042-P0	PHF2	indel	9	96439004	96439004	-	CCTGCCTC
MEN0042-P0	CDC42EP1	indel	22	37964409	37964429	CAGCGCCT	-
MEN0042-P0	SLC25A5	indel	X	118603706	118603706	-	G
MEN0042-P0	TPTE	indel	21	10944697	10944697	A	-
MEN0042-P0	GOLGA8R	indel	15	30700168	30700168	-	TTG
MEN0042-P0	KANK1	indel	9	732475	732477	GAG	-
MEN0042-P0	CD3EAP	indel	19	45911859	45911861	GAA	-
MEN0042-P0	CDKN2C	indel	1	51439798	51439798	C	-
MEN0042-P0	TMEM63A	indel	1	226034840	226034842	CTG	-
MEN0042-P0	FSCB	indel	14	44974154	44974154	-	GGGGCCT(
MEN0042-P0	CTNND2	indel	5	11110989	11110991	TTC	-
MEN0042-P0	SLC51A	indel	3	195955100	195955102	CTG	-
MEN0042-P0	KRTAP4-1	indel	17	39340796	39340852	CGGCAGC/	-
MEN0042-P0	INA	indel	10	105048271	105048273	GAG	-
MEN0042-P0	ADAMTS7	indel	15	79058183	79058183	-	TGGGTCC
MEN0042-P0	KRTAP5-5	indel	11	1651199	1651199	-	GGCTGTG(
MEN0042-P0	CADM1	indel	11	115080312	115080314	TGG	-
MEN0042-P0	CHD2	indel	15	93545433	93545433	-	A
MEN0042-P0	CCNT1	indel	12	49087434	49087436	ATG	-
MEN0042-P0	TERF2IP	indel	16	75690204	75690206	GAA	-
MEN0042-P0	KRTAP4-5	indel	17	39305775	39305775	-	GGCAGCA(
MEN0042-P0	UBXN11	indel	1	26608878	26608883	CCGGGA	-
MEN0042-P0	TSC22D1	indel	13	45148706	45148708	TGC	-
MEN0042-P0	FAM155A	indel	13	108518687	108518689	CTG	-
MEN0042-P0	CCAR1	indel	10	70509285	70509286	GA	-
MEN0042-P0	ATXN1	indel	6	16327913	16327915	TGA	-
MEN0042-P2	APAF1	indel	12	99117445	99117445	A	-
MEN0042-P2	PLAT	indel	8	42038123	42038123	A	-
MEN0042-P2	SLC24A1	indel	15	65918177	65918179	CTG	-
MEN0042-P2	PABPC3	indel	13	25671273	25671273	G	-
MEN0042-P2	OR7C1	indel	19	14910638	14910638	A	-
MEN0042-P2	KCND3	indel	1	112524444	112524444	-	G
MEN0042-P2	CDKN2C	indel	1	51439798	51439798	C	-
MEN0042-P2	COL5A3	indel	19	10071225	10071225	T	-
MEN0042-P2	SLC5A11	indel	16	24921737	24921739	CAG	-
MEN0042-P2	MAGI1	indel	3	65372850	65372851	TC	-
MEN0042-P2	KCNN3	indel	1	154680586	154680588	GCT	-
MEN0042-P2	KDM6A	indel	X	44922688	44922691	CAGG	-
MEN0042-P2	GOLGA8R	indel	15	30700168	30700168	-	TTG
MEN0042-P2	CHEK1	indel	11	125505377	125505377	-	A
MEN0042-P2	CCDC30	indel	1	43002200	43002201	AG	-
MEN0042-P2	BRD7	indel	16	50368678	50368679	CT	-
MEN0042-P2	SLC9B1	indel	4	103822483	103822484	AC	-
MEN0042-P2	TRPV1	indel	17	3491527	3491527	-	T
MEN0042-P2	PEX1	indel	7	92146721	92146721	T	-
MEN0042-P2	FSCB	indel	14	44974154	44974154	-	GGGGCCT(
MEN0042-P2	HAX1	indel	1	154245864	154245866	GAA	-

MEN0042-P2	ALS2CR11	indel	2	202352382	202352387	AATCTG	-
MEN0042-P2	TMEM255B	indel	13	114503876	114503957	CCTGCGG(	-
MEN0042-P2	GBP3	indel	1	89473441	89473441	-	T
MEN0042-P2	MNS1	indel	15	56736723	56736723	T	-
MEN0042-P2	SLC25A5	indel	X	118603706	118603706	-	G
MEN0042-P2	WNK1	indel	12	970297	970297	A	-
MEN0042-P2	PRRC2C	indel	1	171511148	171511148	-	A
MEN0042-P2	ANGPT2	indel	8	6378797	6378797	-	T
MEN0042-P2	KRTAP4-1	indel	17	39340796	39340852	CGGCAGC/	-
MEN0042-P2	MBD4	indel	3	129155548	129155548	T	-
MEN0042-P2	GAGE2D	indel	X	49208295	49208295	-	TAT
MEN0042-P2	PITX1	indel	5	134369504	134369506	GGC	-
MEN0042-P2	SLTM	indel	15	59182564	59182565	CT	-
MEN0042-P2	CWC27	indel	5	64082402	64082402	A	-
MEN0042-P2	ZFR	indel	5	32404161	32404161	T	-
MEN0042-P2	FIP1L1	indel	4	54319248	54319249	AG	-
MEN0042-P2	CDC42EP1	indel	22	37964409	37964429	CAGCGCCT	-
MEN0042-P2	KRTAP4-5	indel	17	39305775	39305775	-	GGCAGCA(
MEN0042-P2	UBXN11	indel	1	26608878	26608883	CCGGGA	-
MEN0042-P2	CCNT1	indel	12	49087434	49087436	ATG	-
MEN0042-P2	ZNF395	indel	8	28209226	28209228	GCA	-
MEN0042-P2	ERC2	indel	3	55733470	55733472	TGG	-
MEN0042-P2	CTNND2	indel	5	11110989	11110991	TTC	-
MEN0042-P2	ZNF395	indel	8	28209226	28209228	GCA	-
MEN0042-P2	PEX1	indel	7	92146721	92146721	T	-
MEN0042-P2	COL5A3	indel	19	10071225	10071225	T	-
MEN0042-P2	KCND3	indel	1	112524444	112524444	-	G
MEN0042-P2	TMEM255B	indel	13	114503876	114503957	CCTGCGG(	-
MEN0042-P2	APAF1	indel	12	99117445	99117445	A	-
MEN0042-P2	PABPC3	indel	13	25671273	25671273	G	-
MEN0042-P2	ALS2CR11	indel	2	202352382	202352387	AATCTG	-
MEN0042-P2	CWC27	indel	5	64082402	64082402	A	-
MEN0042-P2	PLAT	indel	8	42038123	42038123	A	-
MEN0042-P2	GAGE2D	indel	X	49208295	49208295	-	TAT
MEN0042-P2	GBP3	indel	1	89473441	89473441	-	T
MEN0042-P2	BRD7	indel	16	50368678	50368679	CT	-
MEN0042-P2	MAGI1	indel	3	65372850	65372851	TC	-
MEN0042-P2	SLC9B1	indel	4	103822483	103822484	AC	-
MEN0042-P2	KDM6A	indel	X	44922688	44922691	CAGG	-
MEN0042-P2	CDC42EP1	indel	22	37964409	37964429	CAGCGCCT	-
MEN0042-P2	KCNN3	indel	1	154680586	154680588	GCT	-
MEN0042-P2	SLC5A11	indel	16	24921737	24921739	CAG	-
MEN0042-P2	OR7C1	indel	19	14910638	14910638	A	-
MEN0042-P2	WNK1	indel	12	970297	970297	A	-
MEN0042-P2	SLC25A5	indel	X	118603706	118603706	-	G
MEN0042-P2	GOLGA8R	indel	15	30700168	30700168	-	TTG
MEN0042-P2	ZFR	indel	5	32404161	32404161	T	-

MEN0042-P2	MNS1	indel	15	56736723	56736723	T	-
MEN0042-P2	SLC24A1	indel	15	65918177	65918179	CTG	-
MEN0042-P2	CHEK1	indel	11	125505377	125505377	-	A
MEN0042-P2	ERC2	indel	3	55733470	55733472	TGG	-
MEN0042-P2	CDKN2C	indel	1	51439798	51439798	C	-
MEN0042-P2	MBD4	indel	3	129155548	129155548	T	-
MEN0042-P2	TRPV1	indel	17	3491527	3491527	-	T
MEN0042-P2	SLTM	indel	15	59182564	59182565	CT	-
MEN0042-P2	FSCB	indel	14	44974154	44974154	-	GGGGCCT(
MEN0042-P2	CTNND2	indel	5	11110989	11110991	TTC	-
MEN0042-P2	KRTAP4-1	indel	17	39340796	39340852	CGGCAGC/	-
MEN0042-P2	ANGPT2	indel	8	6378797	6378797	-	T
MEN0042-P2	HAX1	indel	1	154245864	154245866	GAA	-
MEN0042-P2	CCNT1	indel	12	49087434	49087436	ATG	-
MEN0042-P2	CCDC30	indel	1	43002200	43002201	AG	-
MEN0042-P2	PITX1	indel	5	134369504	134369506	GGC	-
MEN0042-P2	KRTAP4-5	indel	17	39305775	39305775	-	GGCAGCA(
MEN0042-P2	UBXN11	indel	1	26608878	26608883	CCGGGA	-
MEN0042-P2	PRRC2C	indel	1	171511148	171511148	-	A
MEN0042-P2	FIP1L1	indel	4	54319248	54319249	AG	-
MEN0042G-P1	PLAT	indel	8	42038123	42038123	A	-
MEN0042G-P1	CDKN2C	indel	1	51439798	51439798	C	-
MEN0042G-P1	KDM6A	indel	X	44922688	44922691	CAGG	-
MEN0042G-P1	PLAT	indel	8	42038123	42038123	A	-
MEN0042G-P1	KDM6A	indel	X	44922688	44922691	CAGG	-
MEN0042G-P1	CDKN2C	indel	1	51439798	51439798	C	-
MEN0045-P3	NF2	indel	22	30067836	30067836	C	-
MEN0045-P3	ABL1	indel	9	133759490	133759492	AAG	-
MEN0045-P3	ABL1	indel	9	133759490	133759492	AAG	-
MEN0045-P3	NF2	indel	22	30067836	30067836	C	-
MEN0048-P0	TPTE	indel	21	10944697	10944697	A	-
MEN0048-P0	CRIPAK	indel	4	1388441	1388441	-	CG
MEN0048-P0	SLC9B1	indel	4	103822483	103822484	AC	-
MEN0048-P0	BRD7	indel	16	50368678	50368679	CT	-
MEN0048-P0	ADAMTSL4	indel	1	150530506	150530506	G	-
MEN0048-P0	INPP5E	indel	9	139333416	139333416	C	-
MEN0048-P0	TSKU	indel	11	76506673	76506675	CTG	-
MEN0048-P0	SLC35D2	indel	9	99126780	99126783	TGAA	-
MEN0048-P0	HLA-F	indel	6	29694802	29694802	-	T
MEN0048-P0	PTPN4	indel	2	120643407	120643408	AA	-
MEN0048-P0	PASD1	indel	X	150817142	150817144	GCT	-
MEN0048-P0	POM121	indel	7	72413723	72413723	-	CTC
MEN0048-P0	SLC25A5	indel	X	118603706	118603706	-	G
MEN0048-P0	NOVA1	indel	14	27066575	27066576	GG	-
MEN0048-P0	ALPP	indel	2	233243529	233243531	TGC	-
MEN0048-P0	CACNA1E	indel	1	181680102	181680103	AG	-
MEN0048-P0	LRRIQ3	indel	1	74575212	74575212	-	T

MEN0048-P0	KRTAP4-1	indel	17	39340796	39340852	CGGCAGC/	-
MEN0048-P0	ECM2	indel	9	95277146	95277148	TCC	-
MEN0048-P0	TPCN1	indel	12	113704096	113704098	CTG	-
MEN0048-P0	XPC	indel	3	14219966	14219968	CCT	-
MEN0048-P0	AP3S1	indel	5	115202418	115202421	AAGA	-
MEN0048-P0	TMCC1	indel	3	129546680	129546682	GCA	-
MEN0048-P0	NPIPA5	indel	16	15457565	15457566	AC	-
MEN0048-P0	GOLGA8R	indel	15	30700168	30700168	-	TTG
MEN0048-P0	CLOCK	indel	4	56304530	56304532	CTG	-
MEN0048-P0	PRG4	indel	1	186276143	186276145	CTC	-
MEN0048-P0	LRP10	indel	14	23341527	23341529	CCT	-
MEN0048-P0	MKI67	indel	10	129905112	129905113	TG	-
MEN0048-P0	TMEM63A	indel	1	226034840	226034842	CTG	-
MEN0048-P0	TNN	indel	1	175086297	175086301	AGGGG	-
MEN0048-P0	EMC7	indel	15	34393991	34393993	AGC	-
MEN0048-P0	SLC8A2	indel	19	47935681	47935683	TCC	-
MEN0048-P0	TCHH	indel	1	152082957	152082959	CTC	-
MEN0048-P0	CTNND2	indel	5	11110989	11110991	TTC	-
MEN0048-P0	BHLHB9	indel	X	102004419	102004421	GAG	-
MEN0048-P0	KLHL41	indel	2	170367102	170367103	AA	-
MEN0048-P0	ZFR	indel	5	32419951	32419953	GGT	-
MEN0048-P0	PTPN14	indel	1	214557049	214557051	CCT	-
MEN0048-P0	HAX1	indel	1	154245864	154245866	GAA	-
MEN0048-P0	SLC51A	indel	3	195955100	195955102	CTG	-
MEN0048-P0	RSPH6A	indel	19	46299147	46299149	CCT	-
MEN0048-P0	CTCFL	indel	20	56099187	56099187	T	-
MEN0048-P0	SCAF4	indel	21	33044257	33044259	GCT	-
MEN0048-P0	MAMLD1	indel	X	149639633	149639635	GCA	-
MEN0048-P0	PASD1	indel	X	150817142	150817144	GCT	-
MEN0048-P0	TMCC1	indel	3	129370576	129370578	CTG	-
MEN0048-P0	POM121	indel	7	72413723	72413723	-	CTC
MEN0048-P0	CACNA1E	indel	1	181680102	181680103	AG	-
MEN0048-P0	ADAMTSL4	indel	1	150530506	150530506	G	-
MEN0048-P0	LRP10	indel	14	23341527	23341529	CCT	-
MEN0048-P0	TSKU	indel	11	76506673	76506675	CTG	-
MEN0048-P0	SLC35D2	indel	9	99126780	99126783	TGAA	-
MEN0048-P0	CRIPAK	indel	4	1389382	1389382	C	-
MEN0048-P0	PTPN4	indel	2	120643407	120643408	AA	-
MEN0048-P0	INPP5E	indel	9	139333416	139333416	C	-
MEN0048-P0	NOVA1	indel	14	27066575	27066576	GG	-
MEN0048-P0	LRRIQ3	indel	1	74575212	74575212	-	T
MEN0048-P0	HLA-F	indel	6	29694802	29694802	-	T
MEN0048-P0	AP3S1	indel	5	115202418	115202421	AAGA	-
MEN0048-P0	SLC9B1	indel	4	103822483	103822484	AC	-
MEN0048-P0	TPCN1	indel	12	113704096	113704098	CTG	-
MEN0048-P0	SLC25A5	indel	X	118603706	118603706	-	G
MEN0048-P0	GOLGA8R	indel	15	30700168	30700168	-	TTG

MEN0048-P0	EMC7	indel	15	34393991	34393993	AGC	-
MEN0048-P0	ALPP	indel	2	233243529	233243531	TGC	-
MEN0048-P0	BRD7	indel	16	50368678	50368679	CT	-
MEN0048-P0	KLHL41	indel	2	170367102	170367103	AA	-
MEN0048-P0	CTNND2	indel	5	11110989	11110991	TTC	-
MEN0048-P0	TMEM63A	indel	1	226034840	226034842	CTG	-
MEN0048-P0	KRTAP4-1	indel	17	39340796	39340852	CGGCAGC/	-
MEN0048-P0	SLC8A2	indel	19	47935681	47935683	TCC	-
MEN0048-P0	TPTE	indel	21	10944697	10944697	A	-
MEN0048-P0	TNN	indel	1	175086297	175086301	AGGGG	-
MEN0048-P0	PRG4	indel	1	186276143	186276145	CTC	-
MEN0048-P0	NPIPA5	indel	16	15457565	15457566	AC	-
MEN0048-P0	MKI67	indel	10	129905112	129905113	TG	-
MEN0048-P0	ZFR	indel	5	32419951	32419953	GGT	-
MEN0048-P0	CTCFL	indel	20	56099187	56099187	T	-
MEN0048-P0	TCHH	indel	1	152082957	152082959	CTC	-
MEN0048-P0	PTPN14	indel	1	214557049	214557051	CCT	-
MEN0048-P0	SLC51A	indel	3	195955100	195955102	CTG	-
MEN0048-P0	CLOCK	indel	4	56304530	56304532	CTG	-
MEN0048-P0	HAX1	indel	1	154245864	154245866	GAA	-
MEN0048-P0	XPC	indel	3	14219966	14219968	CCT	-
MEN0048-P0	BHLHB9	indel	X	102004419	102004421	GAG	-
MEN0048-P0	ECM2	indel	9	95277146	95277148	TCC	-
MEN0048-P0	MAMLD1	indel	X	149639633	149639635	GCA	-
MEN0048-P0	SCAF4	indel	21	33044257	33044259	GCT	-
MEN0048-P0	RSPH6A	indel	19	46299147	46299149	CCT	-
MEN0048-P0	HMGB3	indel	X	150156358	150156360	GAG	-
MEN0048-P2	CACNA1B	indel	9	140968025	140968027	TCC	-
MEN0048-P2	CRIPAK	indel	4	1389382	1389382	C	-
MEN0048-P2	KIF7	indel	15	90174936	90174936	G	-
MEN0048-P2	SLC9B1	indel	4	103822483	103822484	AC	-
MEN0048-P2	BRD7	indel	16	50368678	50368679	CT	-
MEN0048-P2	FCER2	indel	19	7763700	7763700	C	-
MEN0048-P2	LOXL3	indel	2	74763923	74763923	-	C
MEN0048-P2	GAGE2D	indel	X	49208295	49208295	-	TAT
MEN0048-P2	WNK1	indel	12	970297	970297	A	-
MEN0048-P2	OPA1	indel	3	193332768	193332769	CT	-
MEN0048-P2	HLA-F	indel	6	29694802	29694802	-	T
MEN0048-P2	PASD1	indel	X	150817142	150817144	GCT	-
MEN0048-P2	LONRF3	indel	X	118151623	118151627	CCTGG	-
MEN0048-P2	SLC25A5	indel	X	118603706	118603706	-	G
MEN0048-P2	LRRIQ3	indel	1	74575212	74575212	-	T
MEN0048-P2	KRTAP4-1	indel	17	39340796	39340852	CGGCAGC/	-
MEN0048-P2	AP3S1	indel	5	115202418	115202421	AAGA	-
MEN0048-P2	COG6	indel	13	40268847	40268857	ATCACCAT	-
MEN0048-P2	GOLGA8R	indel	15	30700168	30700168	-	TTG
MEN0048-P2	VCP	indel	9	35059646	35059646	-	T

MEN0048-P2	MCC	indel	5	112824033	112824033	-	GCTGCC
MEN0048-P2	RANGAP1	indel	22	41642641	41642641	C	-
MEN0048-P2	MKI67	indel	10	129905112	129905113	TG	-
MEN0048-P2	TNN	indel	1	175086297	175086301	AGGGG	-
MEN0048-P2	CTNND2	indel	5	11110989	11110991	TTC	-
MEN0048-P2	KLHL41	indel	2	170367102	170367103	AA	-
MEN0048-P2	PRG4	indel	1	186276284	186276286	CTC	-
MEN0048-P2	ADAM12	indel	10	127753380	127753381	CC	-
MEN0048-P2	SCAF4	indel	21	33044257	33044259	GCT	-
MEN0048-P2	PASD1	indel	X	150817142	150817144	GCT	-
MEN0048-P2	LONRF3	indel	X	118151623	118151627	CCTGG	-
MEN0048-P2	CACNA1B	indel	9	140968025	140968027	TCC	-
MEN0048-P2	COG6	indel	13	40268847	40268857	ATCACCAT	-
MEN0048-P2	CRIPAK	indel	4	1389382	1389382	C	-
MEN0048-P2	KIF7	indel	15	90174936	90174936	G	-
MEN0048-P2	OPA1	indel	3	193332768	193332769	CT	-
MEN0048-P2	GAGE2D	indel	X	49208295	49208295	-	TAT
MEN0048-P2	GAGE2D	indel	X	49208295	49208295	-	TAT
MEN0048-P2	LRRIQ3	indel	1	74575212	74575212	-	T
MEN0048-P2	MCC	indel	5	112824033	112824033	-	GCTGCC
MEN0048-P2	HLA-F	indel	6	29694802	29694802	-	T
MEN0048-P2	AP3S1	indel	5	115202418	115202421	AAGA	-
MEN0048-P2	LOXL3	indel	2	74763923	74763923	-	C
MEN0048-P2	SLC9B1	indel	4	103822483	103822484	AC	-
MEN0048-P2	SLC25A5	indel	X	118603706	118603706	-	G
MEN0048-P2	GOLGA8R	indel	15	30700168	30700168	-	TTG
MEN0048-P2	BRD7	indel	16	50368678	50368679	CT	-
MEN0048-P2	KLHL41	indel	2	170367102	170367103	AA	-
MEN0048-P2	FCER2	indel	19	7763700	7763700	C	-
MEN0048-P2	CTNND2	indel	5	11110989	11110991	TTC	-
MEN0048-P2	KRTAP4-1	indel	17	39340796	39340852	CGGCAGCA	-
MEN0048-P2	TNN	indel	1	175086297	175086301	AGGGG	-
MEN0048-P2	RANGAP1	indel	22	41642641	41642641	C	-
MEN0048-P2	VCP	indel	9	35059646	35059646	-	T
MEN0048-P2	WNK1	indel	12	970297	970297	A	-
MEN0048-P2	PRG4	indel	1	186276284	186276286	CTC	-
MEN0048-P2	MKI67	indel	10	129905112	129905113	TG	-
MEN0048-P2	ADAM12	indel	10	127753380	127753381	CC	-
MEN0048-P2	SCAF4	indel	21	33044257	33044259	GCT	-
MEN0048-P3	CACNA1B	indel	9	140968025	140968027	TCC	-
MEN0048-P3	PRDM2	indel	1	14108749	14108749	A	-
MEN0048-P3	CRIPAK	indel	4	1389456	1389457	CA	-
MEN0048-P3	HOXD1	indel	2	177053855	177053855	-	G
MEN0048-P3	OR7C1	indel	19	14910637	14910637	-	A
MEN0048-P3	SLC9B1	indel	4	103822483	103822484	AC	-
MEN0048-P3	BRD7	indel	16	50368678	50368679	CT	-
MEN0048-P3	ABL1	indel	9	133759490	133759492	AAG	-

MEN0048-P3	GAGE2D	indel	X	49208295	49208295	-	TAT
MEN0048-P3	SEC63	indel	6	108214755	108214755	T	-
MEN0048-P3	OPA1	indel	3	193332768	193332769	CT	-
MEN0048-P3	MYO7A	indel	11	76915137	76915137	G	-
MEN0048-P3	LONRF3	indel	X	118151623	118151627	CCTGG	-
MEN0048-P3	BEX3	indel	X	102632402	102632402	-	A
MEN0048-P3	SLC25A5	indel	X	118603706	118603706	-	G
MEN0048-P3	MIS18BP1	indel	14	45693722	45693722	T	-
MEN0048-P3	KRTAP4-1	indel	17	39340796	39340852	CGGCAGC/	-
MEN0048-P3	COG6	indel	13	40268847	40268857	ATCACCAT	-
MEN0048-P3	GOLGA8R	indel	15	30700168	30700168	-	TTG
MEN0048-P3	MCC	indel	5	112824033	112824033	-	GCTGCC
MEN0048-P3	KRTAP4-5	indel	17	39305775	39305775	-	GGCAGCA(
MEN0048-P3	KATNB1	indel	16	57790780	57790780	C	-
MEN0048-P3	TNN	indel	1	175086297	175086301	AGGGG	-
MEN0048-P3	PLAC4	indel	21	42551272	42551272	C	-
MEN0048-P3	CTNND2	indel	5	11110989	11110991	TTC	-
MEN0048-P3	KLHL41	indel	2	170367102	170367103	AA	-
MEN0048-P3	PRG4	indel	1	186276284	186276286	CTC	-
MEN0048-P3	ZFHX3	indel	16	72822564	72822566	TGC	-
MEN0048-P3	KRTAP5-5	indel	11	1651199	1651199	-	GGCTGTG(
MEN0048-P3	NAP1L2	indel	X	72433664	72433666	TCC	-
MEN0048-P3	ABL1	indel	9	133759490	133759492	AAG	-
MEN0048-P3	HOXD1	indel	2	177053855	177053855	-	G
MEN0048-P3	MYO7A	indel	11	76915137	76915137	G	-
MEN0048-P3	LONRF3	indel	X	118151623	118151627	CCTGG	-
MEN0048-P3	PRDM2	indel	1	14108749	14108749	A	-
MEN0048-P3	CACNA1B	indel	9	140968025	140968027	TCC	-
MEN0048-P3	COG6	indel	13	40268847	40268857	ATCACCAT	-
MEN0048-P3	CRIPAK	indel	4	1389456	1389457	CA	-
MEN0048-P3	KATNB1	indel	16	57790780	57790780	C	-
MEN0048-P3	OPA1	indel	3	193332768	193332769	CT	-
MEN0048-P3	GAGE2D	indel	X	49208295	49208295	-	TAT
MEN0048-P3	GAGE2D	indel	X	49208295	49208295	-	TAT
MEN0048-P3	MCC	indel	5	112824033	112824033	-	GCTGCC
MEN0048-P3	OR7C1	indel	19	14910637	14910637	-	A
MEN0048-P3	SLC9B1	indel	4	103822483	103822484	AC	-
MEN0048-P3	SEC63	indel	6	108214755	108214755	T	-
MEN0048-P3	PLAC4	indel	21	42551272	42551272	C	-
MEN0048-P3	SLC25A5	indel	X	118603706	118603706	-	G
MEN0048-P3	GOLGA8R	indel	15	30700168	30700168	-	TTG
MEN0048-P3	BRD7	indel	16	50368678	50368679	CT	-
MEN0048-P3	BEX3	indel	X	102632402	102632402	-	A
MEN0048-P3	KRTAP5-5	indel	11	1651199	1651199	-	GGCTGTG(
MEN0048-P3	KLHL41	indel	2	170367102	170367103	AA	-
MEN0048-P3	CTNND2	indel	5	11110989	11110991	TTC	-
MEN0048-P3	KRTAP4-1	indel	17	39340796	39340852	CGGCAGC/	-

MEN0048-P3	TNN	indel	1	175086297	175086301	AGGGG	-
MEN0048-P3	PRG4	indel	1	186276284	186276286	CTC	-
MEN0048-P3	MIS18BP1	indel	14	45693722	45693722	T	-
MEN0048-P3	NAP1L2	indel	X	72433664	72433666	TCC	-
MEN0048-P3	MAMLD1	indel	X	149639325	149639327	CAG	-
MEN0048-P3	KRTAP4-5	indel	17	39305775	39305775	-	GGCAGCA(
MEN0048-P3	ZFHX3	indel	16	72822564	72822566	TGC	-
MEN0048G-P1	MMP2	indel	16	55519638	55519638	A	-
MEN0048G-P1	ITGA5	indel	12	54803327	54803327	-	C
MEN0048G-P1	F8	indel	X	154157726	154157726	C	-
MEN0048G-P1	OPA1	indel	3	193332768	193332769	CT	-
MEN0048G-P1	DLG3	indel	X	69672596	69672599	GGGA	-
MEN0048G-P1	COG6	indel	13	40268847	40268857	ATCACCAT	-
MEN0048G-P1	FANCG	indel	9	35078290	35078290	C	-
MEN0048G-P1	LONRF3	indel	X	118151623	118151627	CCTGG	-
MEN0048G-P1	UNC13D	indel	17	73826552	73826554	GGT	-
MEN0048G-P1	DYRK4	indel	12	4708930	4708930	T	-
MEN0048G-P1	TNN	indel	1	175086297	175086301	AGGGG	-
MEN0048G-P1	CCT4	indel	2	62100237	62100254	GTTCCAAT	-
MEN0048G-P1	UHRF1BP1L	indel	12	100452139	100452142	TAAA	-
MEN0048G-P1	KLHL41	indel	2	170367102	170367103	AA	-
MEN0048G-P1	MMP2	indel	16	55519638	55519638	A	-
MEN0048G-P1	LONRF3	indel	X	118151623	118151627	CCTGG	-
MEN0048G-P1	COG6	indel	13	40268847	40268857	ATCACCAT	-
MEN0048G-P1	OPA1	indel	3	193332768	193332769	CT	-
MEN0048G-P1	UNC13D	indel	17	73826552	73826554	GGT	-
MEN0048G-P1	DYRK4	indel	12	4708930	4708930	T	-
MEN0048G-P1	ITGA5	indel	12	54803327	54803327	-	C
MEN0048G-P1	DLG3	indel	X	69672596	69672599	GGGA	-
MEN0048G-P1	F8	indel	X	154157726	154157726	C	-
MEN0048G-P1	KLHL41	indel	2	170367102	170367103	AA	-
MEN0048G-P1	CCT4	indel	2	62100237	62100254	GTTCCAAT	-
MEN0048G-P1	CCT4	indel	2	62100237	62100254	GTTCCAAT	-
MEN0048G-P1	FANCG	indel	9	35078290	35078290	C	-
MEN0048G-P1	TNN	indel	1	175086297	175086301	AGGGG	-
MEN0048G-P1	UHRF1BP1L	indel	12	100452139	100452142	TAAA	-
MEN0088-P	RICTOR	indel	5	38967446	38967458	TGGCACG/	-
MEN0088-P	CREBBP	indel	16	3779136	3779138	TGC	-
MEN0088-P	FLNA	indel	X	153581266	153581266	C	-
MEN0088-P	FCER1A	indel	1	159277635	159277635	G	-
MEN0088-P	TLR2	indel	4	154625390	154625390	C	-
MEN0088-P	ITGB6	indel	2	160994169	160994182	CACACCCC	-
MEN0088-P	UNC13D	indel	17	73829062	73829062	T	-
MEN0088-P	FLNA	indel	X	153581266	153581266	C	-
MEN0088-P	TLR2	indel	4	154625390	154625390	C	-
MEN0088-P	UNC13D	indel	17	73829062	73829062	T	-
MEN0088-P	RICTOR	indel	5	38967446	38967458	TGGCACG/	-

MEN0088-P	CREBBP	indel	16	3779136	3779138	TGC	-
MEN0088-P	FCER1A	indel	1	159277635	159277635	G	-
MEN0088-P	ITGB6	indel	2	160994169	160994182	CACACCCC	-
MEN0089-P	KANK3	indel	19	8399855	8399855	C	-
MEN0089-P	RBM43	indel	2	152108087	152108087	-	T
MEN0089-P	COIL	indel	17	55028117	55028117	-	T
MEN0089-P	RBM43	indel	2	152108087	152108087	-	T
MEN0089-P	KANK3	indel	19	8399855	8399855	C	-
MEN0089-P	COIL	indel	17	55028117	55028117	-	T
MEN0092-P	ADAMTS2	indel	5	178608147	178608147	T	-
MEN0092-P	QSOX1	indel	1	180147959	180147959	A	-
MEN0092-P	LDLR	indel	19	11224276	11224276	C	-
MEN0092-P	PRRT1	indel	6	32118302	32118302	G	-
MEN0092-P	TMEM178A	indel	2	39893463	39893463	A	-
MEN0092-P	KCNMA1	indel	10	78729786	78729786	T	-
MEN0092-P	SKIDA1	indel	10	21805466	21805466	-	CCTCCT
MEN0092-P	RARG	indel	12	53606917	53606923	GGAACAT	-
MEN0092-P	RARG	indel	12	53606917	53606923	GGAACAT	-
MEN0092-P	LDLR	indel	19	11224276	11224276	C	-
MEN0092-P	QSOX1	indel	1	180147959	180147959	A	-
MEN0092-P	TMEM178A	indel	2	39893463	39893463	A	-
MEN0092-P	PRRT1	indel	6	32118302	32118302	G	-
MEN0092-P	ADAMTS2	indel	5	178608147	178608147	T	-
MEN0092-P	KCNMA1	indel	10	78729786	78729786	T	-
MEN0092-P	SKIDA1	indel	10	21805466	21805466	-	CCTCCT
MEN0093-P0	MARCKS	indel	6	114181210	114181210	A	-
MEN0093-P0	STAU2	indel	8	74507471	74507471	T	-
MEN0093-P0	RBM43	indel	2	152108087	152108087	-	T
MEN0093-P0	CRIPAK	indel	4	1389382	1389382	C	-
MEN0093-P0	BRD7	indel	16	50368678	50368679	CT	-
MEN0093-P0	OR7C1	indel	19	14910637	14910637	-	A
MEN0093-P0	CASP5	indel	11	104879687	104879687	T	-
MEN0093-P0	TPTE	indel	21	10944697	10944697	A	-
MEN0093-P0	CDC27	indel	17	45219612	45219612	A	-
MEN0093-P0	POM121	indel	7	72413723	72413723	-	CTC
MEN0093-P0	SLTM	indel	15	59182564	59182565	CT	-
MEN0093-P0	PABPC1	indel	8	101721933	101721933	T	-
MEN0093-P0	DMKN	indel	19	36002362	36002412	CTGCTGCC	-
MEN0093-P0	KRTAP4-1	indel	17	39340796	39340852	CGGCAGC/	-
MEN0093-P0	KRTAP4-5	indel	17	39305775	39305775	-	GGCAGCA(
MEN0093-P0	KRT1	indel	12	53069236	53069256	TAGCTGCT	-
MEN0093-P0	FSCB	indel	14	44974154	44974154	-	GGGGCCT(
MEN0093-P0	KRTAP4-3	indel	17	39324229	39324229	-	GCAGCAG(
MEN0093-P0	KRTAP5-5	indel	11	1651199	1651199	-	GGCTGTG(
MEN0093-P0	STAU2	indel	8	74507471	74507471	T	-
MEN0093-P0	RBM43	indel	2	152108087	152108087	-	T
MEN0093-P0	CASP5	indel	11	104879687	104879687	T	-

MEN0093-P0	CRIPAK	indel	4	1389382	1389382	C	-
MEN0093-P0	POM121	indel	7	72413723	72413723	-	CTC
MEN0093-P0	BRD7	indel	16	50368678	50368679	CT	-
MEN0093-P0	PABPC1	indel	8	101721933	101721933	T	-
MEN0093-P0	OR7C1	indel	19	14910637	14910637	-	A
MEN0093-P0	TPTE	indel	21	10944697	10944697	A	-
MEN0093-P0	CADM1	indel	11	115080312	115080314	TGG	-
MEN0093-P0	MARCKS	indel	6	114181210	114181210	A	-
MEN0093-P0	CDC27	indel	17	45219612	45219612	A	-
MEN0093-P0	FSCB	indel	14	44974154	44974154	-	GGGGCCT
MEN0093-P0	KRTAP4-1	indel	17	39340796	39340852	CGGCAGC	-
MEN0093-P0	KRT1	indel	12	53069236	53069256	TAGCTGCT	-
MEN0093-P0	KRTAP4-3	indel	17	39324229	39324229	-	GCAGCAG
MEN0093-P0	SLTM	indel	15	59182564	59182565	CT	-
MEN0093-P0	DMKN	indel	19	36002362	36002412	CTGCTGCC	-
MEN0093-P0	KRTAP4-5	indel	17	39305775	39305775	-	GGCAGCA
MEN0093-P0	KRTAP5-5	indel	11	1651199	1651199	-	GGCTGTG
MEN0093-P1	PRRC2A	indel	6	31601735	31601735	G	-
MEN0093-P1	BCL10	indel	1	85736511	85736511	T	-
MEN0093-P1	PRDM2	indel	1	14108749	14108749	A	-
MEN0093-P1	GAGE2D	indel	X	49208295	49208295	-	TAT
MEN0093-P1	PLCH1	indel	3	155199981	155199987	TTTGGTT	-
MEN0093-P1	MST1L	indel	1	17086085	17086085	-	C
MEN0093-P1	KIAA0907	indel	1	155886422	155886423	CT	-
MEN0093-P1	ACVR2A	indel	2	148683685	148683685	-	A
MEN0093-P1	CASP5	indel	11	104879687	104879687	T	-
MEN0093-P1	BRD7	indel	16	50368678	50368679	CT	-
MEN0093-P1	GPRIN2	indel	10	46999608	46999608	C	-
MEN0093-P1	CDC27	indel	17	45219612	45219612	A	-
MEN0093-P1	KCNN3	indel	1	154680586	154680588	GCT	-
MEN0093-P1	TPTE	indel	21	10944697	10944697	A	-
MEN0093-P1	PABPC1	indel	8	101721933	101721933	T	-
MEN0093-P1	CHD2	indel	15	93545433	93545433	-	A
MEN0093-P1	CCNT1	indel	12	49087434	49087436	ATG	-
MEN0093-P1	GOLGA8R	indel	15	30700168	30700168	-	TTG
MEN0093-P1	POM121	indel	7	72413723	72413723	-	CTC
MEN0093-P1	LRRIQ3	indel	1	74575212	74575212	-	T
MEN0093-P1	ABLIM1	indel	10	116200799	116200799	-	A
MEN0093-P1	SLTM	indel	15	59182564	59182565	CT	-
MEN0093-P1	KRTAP4-1	indel	17	39340796	39340852	CGGCAGC	-
MEN0093-P1	CD300C	indel	17	72541045	72541045	-	G
MEN0093-P1	KRT1	indel	12	53069236	53069256	TAGCTGCT	-
MEN0093-P1	DMKN	indel	19	36002362	36002412	CTGCTGCC	-
MEN0093-P1	KRTAP4-5	indel	17	39305775	39305775	-	GGCAGCA
MEN0093-P1	SLC51A	indel	3	195955100	195955102	CTG	-
MEN0093-P1	FSCB	indel	14	44974154	44974154	-	GGGGCCT
MEN0093-P1	KRTAP4-3	indel	17	39324229	39324229	-	GCAGCAG

MEN0093-P1	COIL	indel	17	55028117	55028117	-	T
MEN0093-P1	AATK	indel	17	79096114	79096115	GC	-
MEN0093-P1	LCE1F	indel	1	152749003	152749008	TGGCTC	-
MEN0093-P1	ZMYM5	indel	13	20426143	20426145	CAT	-
MEN0093-P1	PTPN14	indel	1	214557049	214557051	CCT	-
MEN0093-P1	PCDH10	indel	4	134073569	134073571	CTG	-
MEN0093-P1	RSPH6A	indel	19	46299147	46299149	CCT	-
MEN0093-P1	TERF2IP	indel	16	75690204	75690206	GAA	-
MEN0093-P1	CASP5	indel	11	104879687	104879687	T	-
MEN0093-P1	GPRIN2	indel	10	46999608	46999608	C	-
MEN0093-P1	POM121	indel	7	72413723	72413723	-	CTC
MEN0093-P1	PRRC2A	indel	6	31601735	31601735	G	-
MEN0093-P1	BRD7	indel	16	50368678	50368679	CT	-
MEN0093-P1	PABPC1	indel	8	101721933	101721933	T	-
MEN0093-P1	BCL10	indel	1	85736511	85736511	T	-
MEN0093-P1	PRDM2	indel	1	14108749	14108749	A	-
MEN0093-P1	KCNN3	indel	1	154680586	154680588	GCT	-
MEN0093-P1	TPTE	indel	21	10944697	10944697	A	-
MEN0093-P1	ABLIM1	indel	10	116200799	116200799	-	A
MEN0093-P1	AATK	indel	17	79096114	79096115	GC	-
MEN0093-P1	CD300C	indel	17	72541045	72541045	-	G
MEN0093-P1	GAGE2D	indel	X	49208295	49208295	-	TAT
MEN0093-P1	KIAA0907	indel	1	155886422	155886423	CT	-
MEN0093-P1	CDC27	indel	17	45219612	45219612	A	-
MEN0093-P1	MST1L	indel	1	17086085	17086085	-	C
MEN0093-P1	PLCH1	indel	3	155199981	155199987	TTTGGTT	-
MEN0093-P1	LRRIQ3	indel	1	74575212	74575212	-	T
MEN0093-P1	ZMYM5	indel	13	20426143	20426145	CAT	-
MEN0093-P1	GOLGA8R	indel	15	30700168	30700168	-	TTG
MEN0093-P1	ACVR2A	indel	2	148683685	148683685	-	A
MEN0093-P1	PTPN14	indel	1	214557049	214557051	CCT	-
MEN0093-P1	FSCB	indel	14	44974154	44974154	-	GGGGCCT(
MEN0093-P1	KRTAP4-1	indel	17	39340796	39340852	CGGCAGC/	-
MEN0093-P1	CCNT1	indel	12	49087434	49087436	ATG	-
MEN0093-P1	LCE1F	indel	1	152749003	152749008	TGGCTC	-
MEN0093-P1	COIL	indel	17	55028117	55028117	-	T
MEN0093-P1	PCDH10	indel	4	134073569	134073571	CTG	-
MEN0093-P1	SLC51A	indel	3	195955100	195955102	CTG	-
MEN0093-P1	KRT1	indel	12	53069236	53069256	TAGCTGCT	-
MEN0093-P1	KRTAP4-3	indel	17	39324229	39324229	-	GCAGCAG(
MEN0093-P1	SLTM	indel	15	59182564	59182565	CT	-
MEN0093-P1	DMKN	indel	19	36002362	36002412	CTGCTGCC	-
MEN0093-P1	KRTAP4-5	indel	17	39305775	39305775	-	GGCAGCA(
MEN0093-P1	TERF2IP	indel	16	75690204	75690206	GAA	-
MEN0093-P1	TSC22D1	indel	13	45148706	45148708	TGC	-
MEN0093-P1	CHD2	indel	15	93545433	93545433	-	A
MEN0093-P1	RSPH6A	indel	19	46299147	46299149	CCT	-

MEN0093-P4	TDRD6	indel	6	46660414	46660414	-	A
MEN0093-P4	PCNT	indel	21	47855966	47855966	C	-
MEN0093-P4	PEX1	indel	7	92146721	92146721	T	-
MEN0093-P4	SACS	indel	13	23914687	23914687	T	-
MEN0093-P4	CRIPAK	indel	4	1389382	1389382	C	-
MEN0093-P4	KIAA0907	indel	1	155886422	155886423	CT	-
MEN0093-P4	FLG2	indel	1	152324558	152324559	TG	-
MEN0093-P4	BRD7	indel	16	50368678	50368679	CT	-
MEN0093-P4	ZNF233	indel	19	44777422	44777422	A	-
MEN0093-P4	FAR2	indel	12	29450110	29450110	A	-
MEN0093-P4	MOK	indel	14	102718302	102718302	-	T
MEN0093-P4	RBM45	indel	2	178988920	178988920	A	-
MEN0093-P4	TPTE	indel	21	10944697	10944697	A	-
MEN0093-P4	RTN4	indel	2	55253745	55253745	-	T
MEN0093-P4	CCDC30	indel	1	43002200	43002201	AG	-
MEN0093-P4	POM121	indel	7	72413723	72413723	-	CTC
MEN0093-P4	SLTM	indel	15	59182564	59182565	CT	-
MEN0093-P4	PABPC1	indel	8	101721933	101721933	T	-
MEN0093-P4	KCNN3	indel	1	154680586	154680588	GCT	-
MEN0093-P4	CACNA1E	indel	1	181680102	181680103	AG	-
MEN0093-P4	FETUB	indel	3	186362544	186362544	A	-
MEN0093-P4	ABLIM1	indel	10	116200799	116200799	-	A
MEN0093-P4	MLH3	indel	14	75514604	75514604	T	-
MEN0093-P4	TMBIM4	indel	12	66531936	66531936	-	A
MEN0093-P4	ZC3H11A	indel	1	203786224	203786224	-	T
MEN0093-P4	AP3S1	indel	5	115202418	115202421	AAGA	-
MEN0093-P4	SEMG2	indel	20	43851983	43851985	ACA	-
MEN0093-P4	CHD2	indel	15	93545433	93545433	-	A
MEN0093-P4	NDUFC2-KCTD14	indel	11	77784147	77784147	A	-
MEN0093-P4	DMKN	indel	19	36002362	36002412	CTGCTGCC	-
MEN0093-P4	KRTAP4-1	indel	17	39340796	39340852	CGGCAGCA	-
MEN0093-P4	ZNF644	indel	1	91406039	91406039	-	T
MEN0093-P4	PHC3	indel	3	169896635	169896637	TGG	-
MEN0093-P4	KRTAP4-5	indel	17	39305775	39305775	-	GGCAGCA
MEN0093-P4	KRT1	indel	12	53069236	53069256	TAGCTGCT	-
MEN0093-P4	UBQLN3	indel	11	5529918	5529920	TGG	-
MEN0093-P4	TMEM63A	indel	1	226034840	226034842	CTG	-
MEN0093-P4	SCN7A	indel	2	167273429	167273430	TC	-
MEN0093-P4	LCE1F	indel	1	152749003	152749008	TGGCTC	-
MEN0093-P4	SLC51A	indel	3	195955100	195955102	CTG	-
MEN0093-P4	MTR	indel	1	237060393	237060393	-	TGTG
MEN0093-P4	SLC8A2	indel	19	47935681	47935683	TCC	-
MEN0093-P4	PLAC4	indel	21	42551272	42551272	C	-
MEN0093-P4	TCHH	indel	1	152082957	152082959	CTC	-
MEN0093-P4	CCNT1	indel	12	49087434	49087436	ATG	-
MEN0093-P4	DBR1	indel	3	137880741	137880743	TCG	-
MEN0093-P4	TOX	indel	8	59750796	59750798	CTT	-

MEN0093-P4	POLE	indel	12	133220099	133220100	CA	-
MEN0093-P4	PCDHA9	indel	5	140230508	140230508	-	T
MEN0093-P4	HMMR	indel	5	162917425	162917425	-	A
MEN0093-P4	ICE2	indel	15	60745782	60745782	A	-
MEN0093-P4	NEFH	indel	22	29885567	29885567	-	AAGTCCCC
MEN0093-P4	GRK4	indel	4	3015470	3015470	A	-
MEN0093-P4	GPALPP1	indel	13	45580365	45580367	GAT	-
MEN0093-P4	PAK1	indel	11	77069990	77069992	CAT	-
MEN0093-P4	RSPH6A	indel	19	46299147	46299149	CCT	-
MEN0093-P4	KRTAP5-5	indel	11	1651199	1651199	-	GGCTGTGC
MEN0093-P4	SACS	indel	13	23914687	23914687	T	-
MEN0093-P4	SACS	indel	13	23914687	23914687	T	-
MEN0093-P4	SACS	indel	13	23914687	23914687	T	-
MEN0093-P4	MTR	indel	1	237060393	237060393	-	TGTG
MEN0093-P4	ZNF233	indel	19	44777422	44777422	A	-
MEN0093-P4	CRIPAK	indel	4	1388622	1388622	-	CA
MEN0093-P4	POM121	indel	7	72413723	72413723	-	CTC
MEN0093-P4	BRD7	indel	16	50368678	50368679	CT	-
MEN0093-P4	PABPC1	indel	8	101721933	101721933	T	-
MEN0093-P4	NDUFC2-KCTD14	indel	11	77784147	77784147	A	-
MEN0093-P4	PEX1	indel	7	92146721	92146721	T	-
MEN0093-P4	AP3S1	indel	5	115202418	115202421	AAGA	-
MEN0093-P4	PHC3	indel	3	169896635	169896637	TGG	-
MEN0093-P4	CACNA1E	indel	1	181680102	181680103	AG	-
MEN0093-P4	KCNN3	indel	1	154680586	154680588	GCT	-
MEN0093-P4	TMBIM4	indel	12	66531936	66531936	-	A
MEN0093-P4	TPTE	indel	21	10944697	10944697	A	-
MEN0093-P4	CADM1	indel	11	115080312	115080314	TGG	-
MEN0093-P4	SLC8A2	indel	19	47935681	47935683	TCC	-
MEN0093-P4	ABLIM1	indel	10	116200799	116200799	-	A
MEN0093-P4	DBR1	indel	3	137880741	137880743	TCG	-
MEN0093-P4	FETUB	indel	3	186362544	186362544	A	-
MEN0093-P4	KIAA0907	indel	1	155886422	155886423	CT	-
MEN0093-P4	FLG2	indel	1	152324558	152324559	TG	-
MEN0093-P4	MLH3	indel	14	75514604	75514604	T	-
MEN0093-P4	ZC3H11A	indel	1	203786224	203786224	-	T
MEN0093-P4	MOK	indel	14	102718302	102718302	-	T
MEN0093-P4	RBM45	indel	2	178988920	178988920	A	-
MEN0093-P4	SCN7A	indel	2	167273429	167273430	TC	-
MEN0093-P4	POLE	indel	12	133220099	133220100	CA	-
MEN0093-P4	TMEM63A	indel	1	226034840	226034842	CTG	-
MEN0093-P4	PCDHA9	indel	5	140230508	140230508	-	T
MEN0093-P4	UBQLN3	indel	11	5529918	5529920	TGG	-
MEN0093-P4	ICE2	indel	15	60745782	60745782	A	-
MEN0093-P4	PCNT	indel	21	47855966	47855966	C	-
MEN0093-P4	TDRD6	indel	6	46660414	46660414	-	A
MEN0093-P4	HMMR	indel	5	162917425	162917425	-	A

MEN0093-P4	PLAC4	indel	21	42551272	42551272	C	-
MEN0093-P4	KRTAP4-1	indel	17	39340796	39340852	CGGCAGC/	-
MEN0093-P4	CCNT1	indel	12	49087434	49087436	ATG	-
MEN0093-P4	LCE1F	indel	1	152749003	152749008	TGGCTC	-
MEN0093-P4	SEMG2	indel	20	43851983	43851985	ACA	-
MEN0093-P4	CCDC30	indel	1	43002200	43002201	AG	-
MEN0093-P4	GRK4	indel	4	3015470	3015470	A	-
MEN0093-P4	FAR2	indel	12	29450110	29450110	A	-
MEN0093-P4	TCHH	indel	1	152082957	152082959	CTC	-
MEN0093-P4	PAK1	indel	11	77069990	77069992	CAT	-
MEN0093-P4	ZNF644	indel	1	91406039	91406039	-	T
MEN0093-P4	SLC51A	indel	3	195955100	195955102	CTG	-
MEN0093-P4	KRT1	indel	12	53069236	53069256	TAGCTGCT	-
MEN0093-P4	SLTM	indel	15	59182564	59182565	CT	-
MEN0093-P4	DMKN	indel	19	36002362	36002412	CTGCTGCC	-
MEN0093-P4	KRTAP4-5	indel	17	39305775	39305775	-	GGCAGCA(
MEN0093-P4	GPALPP1	indel	13	45580365	45580367	GAT	-
MEN0093-P4	KRTAP5-5	indel	11	1651199	1651199	-	GGCTGTG(
MEN0093-P4	CHD2	indel	15	93545433	93545433	-	A
MEN0093-P4	RSPH6A	indel	19	46299147	46299149	CCT	-
MEN0093-P4	NEFH	indel	22	29885567	29885567	-	AAGTCCCC
MEN0093-P4	RTN4	indel	2	55253745	55253745	-	T
MEN0093-P4	TOX	indel	8	59750796	59750798	CTT	-
MEN0093G-P2	ABLIM1	indel	10	116200799	116200799	-	A
MEN0093G-P2	ABLIM1	indel	10	116200799	116200799	-	A
MEN0095-P	RRP1	indel	21	45223401	45223402	GA	-
MEN0095-P	SOX11	indel	2	5833526	5833528	GAC	-
MEN0095-P	RRP1	indel	21	45223401	45223402	GA	-
MEN0095-P	SOX11	indel	2	5833526	5833528	GAC	-
MEN0096-P	EPS15L1	indel	19	16514552	16514552	T	-
MEN0096-P	GTF2H3	indel	12	124144361	124144361	A	-
MEN0096-P	CNPY3	indel	6	42897358	42897360	TGC	-
MEN0096-P	EPS15L1	indel	19	16514552	16514552	T	-
MEN0096-P	CNPY3	indel	6	42897358	42897360	TGC	-
MEN0096-P	GTF2H3	indel	12	124144361	124144361	A	-
MEN0097-P	ZNF805	indel	19	57764455	57764455	C	-
MEN0097-P	SFRP1	indel	8	41166638	41166640	GCT	-
MEN0097-P	RAD51AP2	indel	2	17699325	17699325	-	G
MEN0097-P	CDR1	indel	X	139866058	139866063	TTTGGG	-
MEN0097-P	USP19	indel	3	49154334	49154334	G	-
MEN0097-P	INO80E	indel	16	30007665	30007665	A	-
MEN0097-P	SETBP1	indel	18	42281462	42281484	GGCGGAG	-
MEN0097-P	SFRP1	indel	8	41166638	41166640	GCT	-
MEN0097-P	CDR1	indel	X	139866058	139866063	TTTGGG	-
MEN0097-P	RAD51AP2	indel	2	17699325	17699325	-	G
MEN0097-P	USP19	indel	3	49154334	49154334	G	-
MEN0097-P	ZNF805	indel	19	57764455	57764455	C	-

MEN0097-P	SETBP1	indel	18	42281462	42281484	GGCGGAG	-
MEN0097-P	INO80E	indel	16	30007665	30007665	A	-
MEN0097-P1	SOX17	indel	8	55371874	55371875	CC	-
MEN0097-P1	KIF26A	indel	14	104643204	104643204	C	-
MEN0097-P1	KIF26A	indel	14	104643204	104643204	C	-
MEN0097-P1	SGK223	indel	8	8234868	8234868	-	GCCGCT
MEN0097-P1	VWF	indel	12	6078487	6078489	ACC	-
MEN0097-P1	SETBP1	indel	18	42281462	42281484	GGCGGAG	-
MEN0097-P1	SOX17	indel	8	55371874	55371875	CC	-
MEN0097-P1	VWF	indel	12	6078487	6078489	ACC	-
MEN0097-P1	KIF26A	indel	14	104643204	104643204	C	-
MEN0097-P1	SGK223	indel	8	8234868	8234868	-	GCCGCT
MEN0097-P1	SETBP1	indel	18	42281462	42281484	GGCGGAG	-
MEN0097-P2	CPXM2	indel	10	125528165	125528167	CAG	-
MEN0097-P2	ARID1A	indel	1	27106440	27106446	GCACCAC	-
MEN0097-P2	VWF	indel	12	6078487	6078489	ACC	-
MEN0097-P2	PRB2	indel	12	11546506	11546508	TTG	-
MEN0097-P2	SETBP1	indel	18	42281462	42281484	GGCGGAG	-
MEN0097-P2	GOLGA6A	indel	15	74373009	74373009	-	T
MEN0097-P2	GZF1	indel	20	23345921	23345923	GAG	-
MEN0097-P2	ARID1A	indel	1	27106440	27106446	GCACCAC	-
MEN0097-P2	VWF	indel	12	6078487	6078489	ACC	-
MEN0097-P2	CPXM2	indel	10	125528165	125528167	CAG	-
MEN0097-P2	PRB2	indel	12	11546506	11546508	TTG	-
MEN0097-P2	GOLGA6A	indel	15	74373009	74373009	-	T
MEN0097-P2	SETBP1	indel	18	42281462	42281484	GGCGGAG	-
MEN0097-P2	GZF1	indel	20	23345921	23345923	GAG	-
MEN0097-P3	CELSR1	indel	22	46793616	46793616	G	-
MEN0097-P3	TMCC1	indel	3	129370576	129370578	CTG	-
MEN0097-P3	ARID1A	indel	1	27106440	27106446	GCACCAC	-
MEN0097-P3	VWF	indel	12	6078487	6078489	ACC	-
MEN0097-P3	GOLGA6A	indel	15	74373009	74373009	-	T
MEN0097-P3	FOXJ2	indel	12	8200558	8200560	CAG	-
MEN0097-P3	SKIDA1	indel	10	21805466	21805466	-	CCTCCT
MEN0097-P3	ARID1A	indel	1	27106440	27106446	GCACCAC	-
MEN0097-P3	VWF	indel	12	6078487	6078489	ACC	-
MEN0097-P3	CELSR1	indel	22	46793616	46793616	G	-
MEN0097-P3	TMCC1	indel	3	129370576	129370578	CTG	-
MEN0097-P3	GOLGA6A	indel	15	74373009	74373009	-	T
MEN0097-P3	FOXJ2	indel	12	8200558	8200560	CAG	-
MEN0097-P3	SKIDA1	indel	10	21805466	21805466	-	CCTCCT
MEN0098-P	SEMA3A	indel	7	83640525	83640525	C	-
MEN0098-P	APEX1	indel	14	20924856	20924856	-	T
MEN0098-P	CDH15	indel	16	89246676	89246676	G	-
MEN0098-P	SEMA3A	indel	7	83640525	83640525	C	-
MEN0098-P	CDH15	indel	16	89246676	89246676	G	-
MEN0098-P	APEX1	indel	14	20924856	20924856	-	T

MEN0099-P	NF2	indel	22	30054189	30054189	A	-
MEN0099-P	SLX4IP	indel	20	10603486	10603486	C	-
MEN0099-P	INO80E	indel	16	30016653	30016653	C	-
MEN0099-P	SLX4IP	indel	20	10603486	10603486	C	-
MEN0099-P	INO80E	indel	16	30016653	30016653	C	-
MEN0099-P	NF2	indel	22	30054189	30054189	A	-
MEN0100-P	TTC37	indel	5	94860173	94860191	AGAAAGT(	-
MEN0100-P	SNTB1	indel	8	121823722	121823728	CCCAGCT	-
MEN0100-P	UBQLN3	indel	11	5529918	5529920	TGG	-
MEN0100-P	ACPT	indel	19	51293714	51293716	CTG	-
MEN0100-P	USP24	indel	1	55624643	55624647	GATCC	-
MEN0100-P	TTC37	indel	5	94860173	94860191	AGAAAGT(	-
MEN0100-P	ACPT	indel	19	51293714	51293716	CTG	-
MEN0100-P	USP24	indel	1	55624643	55624647	GATCC	-
MEN0100-P	SNTB1	indel	8	121823722	121823728	CCCAGCT	-
MEN0100-P	UBQLN3	indel	11	5529918	5529920	TGG	-
MEN0101-P0	FGFR4	indel	5	176522693	176522693	C	-
MEN0101-P0	PHRF1	indel	11	609661	609661	G	-
MEN0101-P0	PCBP3	indel	21	47333903	47333903	G	-
MEN0101-P0	PXDC1	indel	6	3751538	3751538	G	-
MEN0101-P0	PRB2	indel	12	11546506	11546508	TTG	-
MEN0101-P0	PHRF1	indel	11	609661	609661	G	-
MEN0101-P0	PCBP3	indel	21	47333903	47333903	G	-
MEN0101-P0	FGFR4	indel	5	176522693	176522693	C	-
MEN0101-P0	PXDC1	indel	6	3751538	3751538	G	-
MEN0101-P0	PRB2	indel	12	11546506	11546508	TTG	-
MEN0101-P1	SCUBE2	indel	11	9055186	9055186	C	-
MEN0101-P1	WDCP	indel	2	24260552	24260552	A	-
MEN0101-P1	ACACA	indel	17	35614787	35614787	C	-
MEN0101-P1	TSC2	indel	16	2136245	2136250	ACGGAG	-
MEN0101-P1	DGAT1	indel	8	145541209	145541215	CCAGCTT	-
MEN0101-P1	OTULIN	indel	5	14664996	14665016	CGGCGCG(	-
MEN0101-P1	TRPM3	indel	9	73167858	73167858	G	-
MEN0101-P1	DGAT1	indel	8	145541209	145541215	CCAGCTT	-
MEN0101-P1	WDCP	indel	2	24260552	24260552	A	-
MEN0101-P1	SCUBE2	indel	11	9055186	9055186	C	-
MEN0101-P1	TSC2	indel	16	2136245	2136250	ACGGAG	-
MEN0101-P1	OTULIN	indel	5	14664996	14665016	CGGCGCG(	-
MEN0101-P1	TRPM3	indel	9	73167858	73167858	G	-
MEN0101-P1	ACACA	indel	17	35614787	35614787	C	-
MEN0102-P	GAGE2D	indel	X	49208295	49208295	-	TAT
MEN0102-P	BRD7	indel	16	50368678	50368679	CT	-
MEN0102-P	NF2	indel	22	30067844	30067844	G	-
MEN0102-P	C9orf43	indel	9	116187645	116187645	-	GCA
MEN0102-P	BHLHE22	indel	8	65494007	65494007	-	AGCGGC
MEN0102-P	BRD7	indel	16	50368678	50368679	CT	-
MEN0102-P	GAGE2D	indel	X	49208295	49208295	-	TAT

MEN0102-P	BHLHE22	indel	8	65494007	65494007	-	AGCGGC
MEN0102-P	NF2	indel	22	30067844	30067844	G	-
MEN0102-P	C9orf43	indel	9	116187645	116187645	-	GCA
MEN0103-P	ADCK5	indel	8	145617535	145617535	G	-
MEN0103-P	ITGAL	indel	16	30510513	30510513	-	C
MEN0103-P	DHX57	indel	2	39029982	39029982	T	-
MEN0103-P	PRB2	indel	12	11546506	11546508	TTG	-
MEN0103-P	HGFAC	indel	4	3443798	3443800	CTG	-
MEN0103-P	FMN2	indel	1	240255569	240255571	GGC	-
MEN0103-P	ADCK5	indel	8	145617535	145617535	G	-
MEN0103-P	DHX57	indel	2	39029982	39029982	T	-
MEN0103-P	ITGAL	indel	16	30510513	30510513	-	C
MEN0103-P	HGFAC	indel	4	3443798	3443800	CTG	-
MEN0103-P	FMN2	indel	1	240255569	240255571	GGC	-
MEN0103-P	PRB2	indel	12	11546506	11546508	TTG	-
MEN0104-P	BRAP	indel	12	112082016	112082016	T	-
MEN0104-P	FAM155A	indel	13	108518687	108518689	CTG	-
MEN0104-P	FAM155A	indel	13	108518687	108518689	CTG	-
MEN0104-P	BRAP	indel	12	112082016	112082016	T	-
MEN0106-P	SYNE1	indel	6	152644769	152644769	T	-
MEN0106-P	GAGE2D	indel	X	49208295	49208295	-	TAT
MEN0106-P	GAGE2D	indel	X	49208295	49208295	-	TAT
MEN0106-P	SYNE1	indel	6	152644769	152644769	T	-
MEN0107-P	TPTE	indel	21	10944697	10944697	A	-
MEN0107-P	GAGE2D	indel	X	49208295	49208295	-	TAT
MEN0107-P	TPTE	indel	21	10944697	10944697	A	-
MEN0107-P	GAGE2D	indel	X	49208295	49208295	-	TAT
MEN0108-P	MRGPRF	indel	11	68772804	68772804	C	-
MEN0108-P	SREBF2	indel	22	42262949	42262951	GCA	-
MEN0108-P	MRGPRF	indel	11	68772804	68772804	C	-
MEN0108-P	SREBF2	indel	22	42262949	42262951	GCA	-
MEN0109-P	KATNAL2	indel	18	44627332	44627333	GC	-
MEN0109-P	UNC119	indel	17	26879420	26879420	C	-
MEN0109-P	GOLGB1	indel	3	121417226	121417226	T	-
MEN0109-P	KATNAL2	indel	18	44627332	44627333	GC	-
MEN0109-P	UNC119	indel	17	26879420	26879420	C	-
MEN0109-P	GOLGB1	indel	3	121417226	121417226	T	-
MEN0110-P	IRF5	indel	7	128587491	128587491	TRUE	-
MEN0110-P	IRF5	indel	7	128587491	128587491	T	-
MEN0110-P	KCNN3	indel	1	154842199	154842199	-	GCTGCT
MEN0111-P	EVX2	indel	2	176945241	176945241	C	-
MEN0111-P	LMTK3	indel	19	49001891	49001891	-	C
MEN0111-P	MUM1	indel	19	1360803	1360804	GG	-
MEN0111-P	SPANXN3	indel	X	142605149	142605149	T	-
MEN0111-P	PASD1	indel	X	150817142	150817144	GCT	-
MEN0111-P	EFNA5	indel	5	106723407	106723409	TTG	-
MEN0111-P	EVX2	indel	2	176945241	176945241	C	-

MEN0111-P	PASD1	indel	X	150817142	150817144	GCT	-
MEN0111-P	LMTK3	indel	19	49001891	49001891	-	C
MEN0111-P	EFNA5	indel	5	106723407	106723409	TTG	-
MEN0111-P	MUM1	indel	19	1360803	1360804	GG	-
MEN0111-P	SPANXN3	indel	X	142605149	142605149	T	-
MEN0112-P	ATAD2	indel	8	124368685	124368685	A	-
MEN0112-P	EDEM1	indel	3	5248941	5248941	T	-
MEN0112-P	SLC27A5	indel	19	59023208	59023208	C	-
MEN0112-P	PPARGC1B	indel	5	149216400	149216402	CAG	-
MEN0112-P	GLTSCR1	indel	19	48185262	48185262	G	-
MEN0112-P	FPGT-TNNI3K	indel	1	74957824	74957826	CTT	-
MEN0112-P	CD99L2	indel	X	149984524	149984526	GTG	-
MEN0112-P	TOX	indel	8	59750796	59750798	CTT	-
MEN0112-P	FPGT-TNNI3K	indel	1	74957824	74957826	CTT	-
MEN0112-P	ATAD2	indel	8	124368685	124368685	A	-
MEN0112-P	GLTSCR1	indel	19	48185262	48185262	G	-
MEN0112-P	SLC27A5	indel	19	59023208	59023208	C	-
MEN0112-P	EDEM1	indel	3	5248941	5248941	T	-
MEN0112-P	PPARGC1B	indel	5	149216400	149216402	CAG	-
MEN0112-P	CD99L2	indel	X	149984524	149984526	GTG	-
MEN0112-P	TOX	indel	8	59750796	59750798	CTT	-
MEN0113-P	NF2	indel	22	30067873	30067873	G	-
MEN0113-P	SEC31A	indel	4	83785565	83785565	T	-
MEN0113-P	ABTB2	indel	11	34219085	34219085	C	-
MEN0113-P	CLEC16A	indel	16	11219957	11219966	CCCCACAG	-
MEN0113-P	ZDHHC12	indel	9	131483486	131483487	CC	-
MEN0113-P	MAST3	indel	19	18254768	18254768	C	-
MEN0113-P	ZFR	indel	5	32419951	32419953	GGT	-
MEN0113-P	ZDHHC12	indel	9	131483486	131483487	CC	-
MEN0113-P	SEC31A	indel	4	83785565	83785565	T	-
MEN0113-P	CLEC16A	indel	16	11219957	11219966	CCCCACAG	-
MEN0113-P	MAST3	indel	19	18254768	18254768	C	-
MEN0113-P	ABTB2	indel	11	34219085	34219085	C	-
MEN0113-P	NF2	indel	22	30067873	30067873	G	-
MEN0113-P	ZFR	indel	5	32419951	32419953	GGT	-
MEN0114-P	FBXO2	indel	1	11710779	11710779	-	GCG
MEN0114-P	ZFP36L2	indel	2	43452888	43452888	-	T
MEN0114-P	TAF1	indel	X	70603000	70603000	A	-
MEN0114-P	PIEZO1	indel	16	88800373	88800375	TCC	-
MEN0114-P	FBXO2	indel	1	11710779	11710779	-	GCG
MEN0114-P	ZFP36L2	indel	2	43452888	43452888	-	T
MEN0114-P	TAF1	indel	X	70603000	70603000	A	-
MEN0114-P	NR4A3	indel	9	102590616	102590618	CAC	-
MEN0114-P	PIEZO1	indel	16	88800373	88800375	TCC	-
MEN0116-P	TMCC1	indel	3	129370576	129370578	CTG	-
MEN0116-P	BRD7	indel	16	50368678	50368679	CT	-
MEN0116-P	GIGYF2	indel	2	233712228	233712230	CAG	-

MEN0116-P	ATXN1	indel	6	16327913	16327915	TGA	-
MEN0116-P	SCAF4	indel	21	33044257	33044259	GCT	-
MEN0116-P	BRD7	indel	16	50368678	50368679	CT	-
MEN0116-P	TMCC1	indel	3	129370576	129370578	CTG	-
MEN0116-P	GIGYF2	indel	2	233712228	233712230	CAG	-
MEN0116-P	SCAF4	indel	21	33044257	33044259	GCT	-
MEN0116-P	ATXN1	indel	6	16327913	16327915	TGA	-
MEN0117-P	KRT76	indel	12	53162772	53162772	-	GCT
MEN0117-P	BRD7	indel	16	50368678	50368679	CT	-
MEN0117-P	CHEK2	indel	22	29091227	29091227	A	-
MEN0117-P	ATXN1	indel	6	16327913	16327915	TGA	-
MEN0117-P	FAM155A	indel	13	108518687	108518689	CTG	-
MEN0117-P	SCAF4	indel	21	33044257	33044259	GCT	-
MEN0117-P	PRG4	indel	1	186275716	186275718	ACT	-
MEN0117-P	ZFHX3	indel	16	72822564	72822566	TGC	-
MEN0117-P	KRT76	indel	12	53162772	53162772	-	GCT
MEN0117-P	BRD7	indel	16	50368678	50368679	CT	-
MEN0117-P	CHEK2	indel	22	29091227	29091227	A	-
MEN0117-P	SCAF4	indel	21	33044257	33044259	GCT	-
MEN0117-P	ZFHX3	indel	16	72822564	72822566	TGC	-
MEN0117-P	PRG4	indel	1	186275716	186275718	ACT	-
MEN0117-P	FAM155A	indel	13	108518687	108518689	CTG	-
MEN0117-P	TMEM229A	indel	7	123672457	123672462	GCTGCT	-
MEN0117-P	ATXN1	indel	6	16327913	16327915	TGA	-
MEN0118-P0	CBLL1	indel	7	107395909	107395909	A	-
MEN0118-P0	SLC1A4	indel	2	65217006	65217006	T	-
MEN0118-P0	NAP1L5	indel	4	89618484	89618486	TCC	-
MEN0118-P0	CBLL1	indel	7	107395909	107395909	A	-
MEN0118-P0	SLC1A4	indel	2	65217006	65217006	T	-
MEN0118-P0	NAP1L5	indel	4	89618484	89618486	TCC	-
MEN0118-P0	TSC22D1	indel	13	45148706	45148708	TGC	-
MEN0118-P1	CEP192	indel	18	13092454	13092480	GAGAATT(	-
MEN0118-P1	KCTD1	indel	18	24128262	24128264	TCC	-
MEN0118-P1	CEP192	indel	18	13092454	13092480	GAGAATT(	-
MEN0118-P1	KCTD1	indel	18	24128262	24128264	TCC	-
MEN0118-P1	TSC22D1	indel	13	45148706	45148708	TGC	-
MEN0119-P0	RAPGEF4	indel	2	173898665	173898665	G	-
MEN0119-P0	PSMA1	indel	11	14529252	14529254	CAT	-
MEN0119-P0	RARRES3	indel	11	63313709	63313709	A	-
MEN0119-P0	KCNN3	indel	1	154680586	154680588	GCT	-
MEN0119-P0	GLCCI1	indel	7	8126097	8126099	CAG	-
MEN0119-P0	KCNN2	indel	5	113698631	113698631	-	GCC
MEN0119-P0	PRB2	indel	12	11546506	11546508	TTG	-
MEN0119-P0	POU6F2	indel	7	39379288	39379290	CAG	-
MEN0119-P0	KCNN2	indel	5	113698631	113698631	-	GCC
MEN0119-P0	RARRES3	indel	11	63313709	63313709	A	-
MEN0119-P0	RAPGEF4	indel	2	173898665	173898665	G	-

MEN0119-P0	KCNN3	indel	1	154680586	154680588	GCT	-
MEN0119-P0	PSMA1	indel	11	14529252	14529254	CAT	-
MEN0119-P0	GLCCI1	indel	7	8126097	8126099	CAG	-
MEN0119-P0	POU6F2	indel	7	39379288	39379290	CAG	-
MEN0119-P0	PRB2	indel	12	11546506	11546508	TTG	-
MEN0119-P1	ZMYND15	indel	17	4647712	4647712	G	-
MEN0119-P1	ZNF318	indel	6	43323502	43323502	T	-
MEN0119-P1	RAPGEF4	indel	2	173898665	173898665	G	-
MEN0119-P1	LRCH1	indel	13	47243183	47243186	CCTG	-
MEN0119-P1	GATA5	indel	20	61050249	61050249	C	-
MEN0119-P1	OTOF	indel	2	26705424	26705424	G	-
MEN0119-P1	PAXIP1	indel	7	154760670	154760672	TGC	-
MEN0119-P1	ZNF608	indel	5	124079813	124079815	CTC	-
MEN0119-P1	PRB2	indel	12	11546506	11546508	TTG	-
MEN0119-P1	ZNF318	indel	6	43323502	43323502	T	-
MEN0119-P1	RAPGEF4	indel	2	173898665	173898665	G	-
MEN0119-P1	ZMYND15	indel	17	4647712	4647712	G	-
MEN0119-P1	LRCH1	indel	13	47243183	47243186	CCTG	-
MEN0119-P1	GATA5	indel	20	61050249	61050249	C	-
MEN0119-P1	OTOF	indel	2	26705424	26705424	G	-
MEN0119-P1	ZNF608	indel	5	124079813	124079815	CTC	-
MEN0119-P1	PAXIP1	indel	7	154760670	154760672	TGC	-
MEN0119-P1	PRB2	indel	12	11546506	11546508	TTG	-
MEN0119-P1	TMEM229A	indel	7	123672457	123672459	GCT	-
MEN0120-P	KLK8	indel	19	51504365	51504365	C	-
MEN0120-P	ATM	indel	11	108172464	108172464	-	AACAG
MEN0120-P	AGAP4	indel	10	46342668	46342688	GCTCCTGC	-
MEN0120-P	GAGE2D	indel	X	49208295	49208295	-	TAT
MEN0120-P	ZNF729	indel	19	22497476	22497476	-	A
MEN0120-P	SKIDA1	indel	10	21805466	21805466	-	CCTCCT
MEN0120-P	AGAP4	indel	10	46342668	46342688	GCTCCTGC	-
MEN0120-P	KLK8	indel	19	51504365	51504365	C	-
MEN0120-P	ZNF729	indel	19	22497476	22497476	-	A
MEN0120-P	ATM	indel	11	108172464	108172464	-	AACAG
MEN0120-P	GAGE2D	indel	X	49208295	49208295	-	TAT
MEN0120-P	SKIDA1	indel	10	21805466	21805466	-	CCTCCT
MEN0120-P1	MPRIP	indel	17	17061918	17061918	C	-
MEN0120-P1	ATM	indel	11	108172464	108172464	-	AACAG
MEN0120-P1	SAPCD2	indel	9	139959609	139959609	G	-
MEN0120-P1	FASN	indel	17	80039118	80039118	C	-
MEN0120-P1	STX12	indel	1	28099861	28099861	T	-
MEN0120-P1	WFIKKN1	indel	16	684051	684051	G	-
MEN0120-P1	ZNF729	indel	19	22497476	22497476	-	A
MEN0120-P1	MPRIP	indel	17	17061918	17061918	C	-
MEN0120-P1	STX12	indel	1	28099861	28099861	T	-
MEN0120-P1	SAPCD2	indel	9	139959609	139959609	G	-
MEN0120-P1	ZNF729	indel	19	22497476	22497476	-	A

MEN0120-P1	ATM	indel	11	108172464	108172464	-	AACAG
MEN0120-P1	FASN	indel	17	80039118	80039118	C	-
MEN0120-P1	CADM1	indel	11	115080312	115080314	TGG	-
MEN0120-P1	WFIKKN1	indel	16	684051	684051	G	-
MEN0121-P1	NAE1	indel	16	66844289	66844289	-	T
MEN0121-P1	ASB16	indel	17	42248186	42248186	C	-
MEN0121-P1	RAB3B	indel	1	52403072	52403072	G	-
MEN0121-P1	ZNF728	indel	19	23158289	23158289	C	-
MEN0121-P1	ASB16	indel	17	42248186	42248186	C	-
MEN0121-P1	NAE1	indel	16	66844289	66844289	-	T
MEN0121-P1	ZNF728	indel	19	23158289	23158289	C	-
MEN0121-P1	RAB3B	indel	1	52403072	52403072	G	-
MEN0121-P2	ADGRD1	indel	12	131439031	131439031	-	C
MEN0121-P2	NAE1	indel	16	66844289	66844289	-	T
MEN0121-P2	ZNF544	indel	19	58758777	58758777	-	A
MEN0121-P2	MYL6B	indel	12	56547773	56547775	CAG	-
MEN0121-P2	ADGRD1	indel	12	131439031	131439031	-	C
MEN0121-P2	MYL6B	indel	12	56547773	56547775	CAG	-
MEN0121-P2	NAE1	indel	16	66844289	66844289	-	T
MEN0121-P2	ZNF544	indel	19	58758777	58758777	-	A
MEN0121-P3	NAE1	indel	16	66844289	66844289	-	T
MEN0121-P3	SYCP1	indel	1	115438125	115438125	G	-
MEN0121-P3	ANK2	indel	4	114214679	114214681	CAC	-
MEN0121-P3	PCDHA11	indel	5	140249857	140249864	CCCACGTT	-
MEN0121-P3	SCN3A	indel	2	166003398	166003398	-	T
MEN0121-P3	SYCP1	indel	1	115438125	115438125	G	-
MEN0121-P3	NAE1	indel	16	66844289	66844289	-	T
MEN0121-P3	PCDHA11	indel	5	140249857	140249864	CCCACGTT	-
MEN0121-P3	ANK2	indel	4	114214679	114214681	CAC	-
MEN0121-P3	SCN3A	indel	2	166003398	166003398	-	T
MEN0121-P4	NAE1	indel	16	66844289	66844289	-	T
MEN0121-P4	SPEF2	indel	5	35800137	35800137	T	-
MEN0121-P4	ULK1	indel	12	132401547	132401547	G	-
MEN0121-P4	SLC25A3	indel	12	98993827	98993828	AA	-
MEN0121-P4	PERM1	indel	1	915137	915137	C	-
MEN0121-P4	RNF213	indel	17	78313185	78313194	TGGCAGC(	-
MEN0121-P4	ANK2	indel	4	114214679	114214681	CAC	-
MEN0121-P4	WASF3	indel	13	27255386	27255386	-	C
MEN0121-P4	TRPC6	indel	11	101341944	101341944	T	-
MEN0121-P4	GPANK1	indel	6	31630207	31630207	G	-
MEN0121-P4	SLAIN1	indel	13	78335213	78335230	GCCTCGAA	-
MEN0121-P4	PERM1	indel	1	915137	915137	C	-
MEN0121-P4	SPEF2	indel	5	35800137	35800137	T	-
MEN0121-P4	ULK1	indel	12	132401547	132401547	G	-
MEN0121-P4	NAE1	indel	16	66844289	66844289	-	T
MEN0121-P4	SLAIN1	indel	13	78335213	78335230	GCCTCGAA	-
MEN0121-P4	TRPC6	indel	11	101341944	101341944	T	-

MEN0121-P4	SLC25A3	indel	12	98993827	98993828	AA	-
MEN0121-P4	GPANK1	indel	6	31630207	31630207	G	-
MEN0121-P4	RNF213	indel	17	78313185	78313194	TGGCAGC(	-
MEN0121-P4	ANK2	indel	4	114214679	114214681	CAC	-
MEN0121-P4	WASF3	indel	13	27255386	27255386	-	C
MEN0122-RBM-P1	PTPN23	indel	3	47452580	47452580	C	-
MEN0122-RBM-P1	NF2	indel	22	30032804	30032804	G	-
MEN0122-RBM-P1	DISP3	indel	1	11595128	11595128	C	-
MEN0122-RBM-P1	GUCY2F	indel	X	108718643	108718643	C	-
MEN0122-RBM-P1	KCNH8	indel	3	19554728	19554728	C	-
MEN0122-RBM-P1	NF2	indel	22	30032804	30032804	G	-
MEN0122-RBM-P1	PTPN23	indel	3	47452580	47452580	C	-
MEN0122-RBM-P1	DISP3	indel	1	11595128	11595128	C	-
MEN0122-RBM-P1	GUCY2F	indel	X	108718643	108718643	C	-
MEN0122-RBM-P1	KCNH8	indel	3	19554728	19554728	C	-
MEN0122-RBM-P2	FAM181B	indel	11	82444173	82444173	C	-
MEN0122-RBM-P2	NF2	indel	22	30032804	30032804	G	-
MEN0122-RBM-P2	SBNO2	indel	19	1122154	1122154	T	-
MEN0122-RBM-P2	BAP1	indel	3	52437302	52437311	CCCTTCCC/	-
MEN0122-RBM-P2	GUCY2F	indel	X	108718643	108718643	C	-
MEN0122-RBM-P2	BOD1L1	indel	4	13605020	13605020	-	TG
MEN0122-RBM-P2	EXTL1	indel	1	26349533	26349535	CCT	-
MEN0122-RBM-P2	BOD1L1	indel	4	13605020	13605020	-	TG
MEN0122-RBM-P2	NF2	indel	22	30032804	30032804	G	-
MEN0122-RBM-P2	EXTL1	indel	1	26349533	26349535	CCT	-
MEN0122-RBM-P2	GUCY2F	indel	X	108718643	108718643	C	-
MEN0122-RBM-P2	BAP1	indel	3	52437302	52437311	CCCTTCCC/	-
MEN0122-RBM-P2	FAM181B	indel	11	82444173	82444173	C	-
MEN0122-RBM-P2	SBNO2	indel	19	1122154	1122154	T	-
MEN0122-RBM-P3	JAKMIP3	indel	10	133955541	133955541	C	-
MEN0122-RBM-P3	NF2	indel	22	30032804	30032804	G	-
MEN0122-RBM-P3	BAP1	indel	3	52437302	52437311	CCCTTCCC/	-
MEN0122-RBM-P3	GUCY2F	indel	X	108718643	108718643	C	-
MEN0122-RBM-P3	BOD1L1	indel	4	13605020	13605020	-	TG
MEN0122-RBM-P3	RCOR2	indel	11	63679589	63679589	A	-
MEN0122-RBM-P3	OLIG1	indel	21	34442677	34442679	CCT	-
MEN0122-RBM-P3	HBS1L	indel	6	135357714	135357714	T	-
MEN0122-RBM-P3	TAF15	indel	17	34171570	34171572	AGT	-
MEN0122-RBM-P3	BOD1L1	indel	4	13605020	13605020	-	TG
MEN0122-RBM-P3	NF2	indel	22	30032804	30032804	G	-
MEN0122-RBM-P3	GUCY2F	indel	X	108718643	108718643	C	-
MEN0122-RBM-P3	BAP1	indel	3	52437302	52437311	CCCTTCCC/	-
MEN0122-RBM-P3	HBS1L	indel	6	135357714	135357714	T	-
MEN0122-RBM-P3	JAKMIP3	indel	10	133955541	133955541	C	-
MEN0122-RBM-P3	OLIG1	indel	21	34442677	34442679	CCT	-
MEN0122-RBM-P3	RCOR2	indel	11	63679589	63679589	A	-
MEN0122-RBM-P3	TAF15	indel	17	34171570	34171572	AGT	-

MEN0122-RBM-P4	NF2	indel	22	30032804	30032804	G	-
MEN0122-RBM-P4	GUCY2F	indel	X	108718643	108718643	C	-
MEN0122-RBM-P4	BOD1L1	indel	4	13605020	13605020	-	TG
MEN0122-RBM-P4	BOD1L1	indel	4	13605020	13605020	-	TG
MEN0122-RBM-P4	NF2	indel	22	30032804	30032804	G	-
MEN0122-RBM-P4	GUCY2F	indel	X	108718643	108718643	C	-
MEN0122-RBM-P5	NFAM1	indel	22	42783070	42783070	G	-
MEN0122-RBM-P5	PLD4	indel	14	105398216	105398216	C	-
MEN0122-RBM-P5	NF2	indel	22	30032804	30032804	G	-
MEN0122-RBM-P5	KRTAP9-9	indel	17	39411670	39411670	-	ACCTGCTG
MEN0122-RBM-P5	BAP1	indel	3	52437302	52437311	CCCTTCCCA	-
MEN0122-RBM-P5	HRCT1	indel	9	35906605	35906607	CCG	-
MEN0122-RBM-P5	GUCY2F	indel	X	108718643	108718643	C	-
MEN0122-RBM-P5	BOD1L1	indel	4	13605020	13605020	-	TG
MEN0122-RBM-P5	BOD1	indel	5	173043417	173043419	CCG	-
MEN0122-RBM-P5	BOD1L1	indel	4	13605020	13605020	-	TG
MEN0122-RBM-P5	NF2	indel	22	30032804	30032804	G	-
MEN0122-RBM-P5	NFAM1	indel	22	42783070	42783070	G	-
MEN0122-RBM-P5	GUCY2F	indel	X	108718643	108718643	C	-
MEN0122-RBM-P5	PLD4	indel	14	105398216	105398216	C	-
MEN0122-RBM-P5	BAP1	indel	3	52437302	52437311	CCCTTCCCA	-
MEN0122-RBM-P5	HRCT1	indel	9	35906605	35906607	CCG	-
MEN0122-RBM-P5	KRTAP9-9	indel	17	39411670	39411670	-	ACCTGCTG
MEN0122-RBM-P5	BOD1	indel	5	173043417	173043419	CCG	-
MENex001	AMER1	indel	X	63412973	63412973	C	-
MENex001	JPH3	indel	16	87637894	87637914	CTGCTGCT	-
MENex001	DICER1	indel	14	95562885	95562885	T	-
MENex001	CD300A	indel	17	72469708	72469708	-	G
MENex001	LOC729159	indel	16	60392816	60392816	G	-
MENex001	JPH3	indel	16	87637894	87637914	CTGCTGCT	-
MENex001	AMER1	indel	X	63412973	63412973	C	-
MENex001	CD300A	indel	17	72469708	72469708	-	G
MENex001	DICER1	indel	14	95562885	95562885	T	-
MENex001	LOC729159	indel	16	60392816	60392816	G	-
MENex002	FBXO2	indel	1	11710779	11710779	-	GCG
MENex002	FBXO2	indel	1	11710779	11710779	-	GCG
MENex004	RABEP1	indel	17	5266247	5266248	TG	-
MENex004	RABEP1	indel	17	5266247	5266248	TG	-
MENex005	RGPD8	indel	2	113147266	113147268	TTT	-
MENex005	CD300C	indel	17	72541049	72541049	-	C
MENex005	MRC2	indel	17	60757561	60757561	-	ACGACG
MENex005	TAS2R7	indel	12	10954224	10954225	TA	-
MENex005	NEFH	indel	22	29885859	29885876	GCTAAGTC	-
MENex005	RGPD4	indel	2	108487715	108487717	AAA	-
MENex005	CD300C	indel	17	72541049	72541049	-	C
MENex005	TAS2R7	indel	12	10954224	10954225	TA	-
MENex005	RGPD8	indel	2	113147266	113147268	TTT	-

MENex005	RGPD4	indel	2	108487715	108487717	AAA	-
MENex005	MRC2	indel	17	60757561	60757561	-	ACGACG
MENex005	NEFH	indel	22	29885859	29885876	GCTAAGT(	-
MENex006	MSH3	indel	5	79950742	79950750	CCCCCAGC	-
MENex006	MSH3	indel	5	79950742	79950750	CCCCCAGC	-
MENex006	CHERP	indel	19	16640581	16640583	TGC	-
MENex006	GOLGA6L2	indel	15	23685826	23685828	TCT	-
MEN0030-P0	MT-ND6	snp	MT	14178	14178	T	C
MEN0030-P0	FAT1	snp	4	187630235	187630235	C	A
MEN0030-P0	MT-ND5	snp	MT	13105	13105	A	G
MEN0030-P0	AL592183.1	snp	GL000219.1	78903	78903	C	T
MEN0030-P0	FRG1B	snp	20	29624081	29624081	A	T
MEN0030-P0	KMT2C	snp	7	151962222	151962222	C	A
MEN0030-P0	ZAN	snp	7	100345257	100345257	G	A
MEN0030-P0	MT-CYB	snp	MT	14769	14769	A	G
MEN0030-P0	MT-ND5	snp	MT	13880	13880	C	A
MEN0030-P0	MT-ND5	snp	MT	13789	13789	T	C
MEN0030-P0	RAG1	snp	11	36595156	36595156	C	T
MEN0030-P0	PRG4	snp	1	186275510	186275510	A	C
MEN0030-P0	MT-ND5	snp	MT	14053	14053	A	G
MEN0030-P0	PRG4	snp	1	186278008	186278008	C	T
MEN0030-P0	LRP1B	snp	2	141081479	141081479	C	T
MEN0030-P0	POTEE	snp	2	131976126	131976126	G	A
MEN0030-P0	PRG4	snp	1	186277568	186277568	C	T
MEN0030-P0	CTNNA1	snp	5	138147906	138147906	G	A
MEN0030-P0	POTEF	snp	2	130877871	130877871	C	G
MEN0030-P0	KMT2C	snp	7	151859495	151859495	C	G
MEN0030-P0	RAG1	snp	11	36595156	36595156	C	T
MEN0030-P0	MT-ND5	snp	MT	13880	13880	C	A
MEN0030-P0	MT-CYB	snp	MT	14769	14769	A	G
MEN0030-P0	POTEE	snp	2	131976126	131976126	G	A
MEN0030-P0	MT-ND5	snp	MT	13105	13105	A	G
MEN0030-P0	MT-ND5	snp	MT	13789	13789	T	C
MEN0030-P0	LRP1B	snp	2	141081479	141081479	C	T
MEN0030-P0	FAT1	snp	4	187630235	187630235	C	A
MEN0030-P0	MT-ND5	snp	MT	14053	14053	A	G
MEN0030-P0	FRG1B	snp	20	29624081	29624081	A	T
MEN0030-P0	MT-ND6	snp	MT	14178	14178	T	C
MEN0030-P0	ZAN	snp	7	100345257	100345257	G	A
MEN0030-P0	PRG4	snp	1	186275510	186275510	A	C
MEN0030-P0	AL592183.1	snp	GL000219.1	78903	78903	C	T
MEN0030-P0	CTNNA1	snp	5	138147906	138147906	G	A
MEN0030-P0	KMT2C	snp	7	151962222	151962222	C	A
MEN0030-P0	POTEF	snp	2	130877871	130877871	C	G
MEN0030-P0	PRG4	snp	1	186278008	186278008	C	T
MEN0030-P0	PRG4	snp	1	186277568	186277568	C	T
MEN0030-P0	KMT2C	snp	7	151859495	151859495	C	G

MEN0030-P1	MT-ND6	snp	MT	14178	14178	T	C
MEN0030-P1	FAT1	snp	4	187630235	187630235	C	A
MEN0030-P1	POTEF	snp	2	130877910	130877910	T	A
MEN0030-P1	MT-CYB	snp	MT	14861	14861	G	A
MEN0030-P1	MT-ND5	snp	MT	13105	13105	A	G
MEN0030-P1	AL592183.1	snp	GL000219.1	78903	78903	C	T
MEN0030-P1	FRG1B	snp	20	29624081	29624081	A	T
MEN0030-P1	MT-ATP6	snp	MT	8701	8701	A	G
MEN0030-P1	KMT2C	snp	7	151962222	151962222	C	A
MEN0030-P1	ZAN	snp	7	100345257	100345257	G	A
MEN0030-P1	MT-CYB	snp	MT	14769	14769	A	G
MEN0030-P1	MT-ND5	snp	MT	13880	13880	C	A
MEN0030-P1	MT-ND5	snp	MT	13789	13789	T	C
MEN0030-P1	RAG1	snp	11	36595156	36595156	C	T
MEN0030-P1	AKT1	snp	14	105246551	105246551	C	T
MEN0030-P1	PRG4	snp	1	186275510	186275510	A	C
MEN0030-P1	MT-ND5	snp	MT	14053	14053	A	G
MEN0030-P1	PRG4	snp	1	186278008	186278008	C	T
MEN0030-P1	LRP1B	snp	2	141081479	141081479	C	T
MEN0030-P1	MT-CO1	snp	MT	7389	7389	T	C
MEN0030-P1	POTEE	snp	2	131976126	131976126	G	A
MEN0030-P1	PRG4	snp	1	186277568	186277568	C	T
MEN0030-P1	CTNNA1	snp	5	138147906	138147906	G	A
MEN0030-P1	POTEF	snp	2	130877871	130877871	C	G
MEN0030-P1	KMT2C	snp	7	151859495	151859495	C	G
MEN0030-P1	RAG1	snp	11	36595156	36595156	C	T
MEN0030-P1	MT-ATP6	snp	MT	8701	8701	A	G
MEN0030-P1	AKT1	snp	14	105246551	105246551	C	T
MEN0030-P1	MT-ND5	snp	MT	13880	13880	C	A
MEN0030-P1	MT-CYB	snp	MT	14769	14769	A	G
MEN0030-P1	POTEE	snp	2	131976126	131976126	G	A
MEN0030-P1	POTEF	snp	2	130877938	130877938	C	T
MEN0030-P1	MT-ND5	snp	MT	13105	13105	A	G
MEN0030-P1	MT-ND5	snp	MT	13789	13789	T	C
MEN0030-P1	LRP1B	snp	2	141081479	141081479	C	T
MEN0030-P1	MT-CYB	snp	MT	14861	14861	G	A
MEN0030-P1	FAT1	snp	4	187630235	187630235	C	A
MEN0030-P1	MT-ND5	snp	MT	14053	14053	A	G
MEN0030-P1	FRG1B	snp	20	29624081	29624081	A	T
MEN0030-P1	MT-ND6	snp	MT	14178	14178	T	C
MEN0030-P1	ZAN	snp	7	100345257	100345257	G	A
MEN0030-P1	PRG4	snp	1	186275510	186275510	A	C
MEN0030-P1	AL592183.1	snp	GL000219.1	78903	78903	C	T
MEN0030-P1	CTNNA1	snp	5	138147906	138147906	G	A
MEN0030-P1	KMT2C	snp	7	151962222	151962222	C	A
MEN0030-P1	POTEF	snp	2	130877871	130877871	C	G
MEN0030-P1	POTEF	snp	2	130877910	130877910	T	A

MEN0030-P1	MT-CO1	snp	MT	7389	7389	T	C
MEN0030-P1	PRG4	snp	1	186278008	186278008	C	T
MEN0030-P1	PRG4	snp	1	186277568	186277568	C	T
MEN0030-P1	KMT2C	snp	7	151859495	151859495	C	G
MEN0042-P0	NBPF9	snp	1	144828707	144828707	G	T
MEN0042-P0	NBPF10	snp	1	145304613	145304613	T	C
MEN0042-P0	LPAR2	snp	19	19737520	19737520	C	T
MEN0042-P0	SRGAP1	snp	12	64383808	64383808	C	T
MEN0042-P0	FRG1B	snp	20	29623231	29623231	C	T
MEN0042-P0	NBPF10	snp	1	145296448	145296448	T	A
MEN0042-P0	AL592183.1	snp	GL000219.1	83290	83290	C	T
MEN0042-P0	ANKRD36	snp	2	97869931	97869931	A	T
MEN0042-P0	NBPF9	snp	1	144828683	144828683	C	G
MEN0042-P0	SLC27A5	snp	19	59011977	59011977	G	A
MEN0042-P0	AC109135.1	snp	GL000192.1	311344	311344	C	T
MEN0042-P0	FRG1B	snp	20	29625934	29625934	C	T
MEN0042-P0	ANKRD30B	snp	18	14779986	14779986	G	A
MEN0042-P0	PCSK5	snp	9	78790187	78790187	G	C
MEN0042-P0	IGKV1-17	snp	2	89416981	89416981	C	T
MEN0042-P0	UBR5	snp	8	103338877	103338877	A	C
MEN0042-P0	MAPK10	snp	4	87080596	87080596	A	G
MEN0042-P0	ZNF844	snp	19	12187394	12187394	T	C
MEN0042-P0	STAT6	snp	12	57493645	57493645	A	C
MEN0042-P0	DHRS3	snp	1	12640591	12640591	C	T
MEN0042-P0	SWT1	snp	1	185153955	185153955	C	T
MEN0042-P0	SLC9A5	snp	16	67289037	67289037	C	G
MEN0042-P0	POTEC	snp	18	14513675	14513675	T	C
MEN0042-P0	TMIGD1	snp	17	28651971	28651971	C	T
MEN0042-P0	RREB1	snp	6	7229893	7229893	A	C
MEN0042-P0	TCP10L2	snp	6	167591954	167591954	G	A
MEN0042-P0	FLG	snp	1	152281113	152281113	G	C
MEN0042-P0	SVIL	snp	10	29770552	29770552	T	G
MEN0042-P0	NBPF9	snp	1	144828599	144828599	G	A
MEN0042-P0	LYST	snp	1	235922642	235922642	C	T
MEN0042-P0	FOXP2	snp	7	114293993	114293993	C	T
MEN0042-P0	TMEM14E	snp	3	152058521	152058521	C	T
MEN0042-P0	POTEC	snp	18	14542978	14542978	C	T
MEN0042-P0	FLG	snp	1	152275960	152275960	T	C
MEN0042-P0	IGF2BP3	snp	7	23352422	23352422	G	A
MEN0042-P0	NBPF10	snp	1	145323690	145323690	A	G
MEN0042-P0	SCNN1G	snp	16	23226732	23226732	G	A
MEN0042-P0	FSCB	snp	14	44975827	44975827	G	T
MEN0042-P0	MDK	snp	11	46404331	46404331	C	G
MEN0042-P0	EPN3	snp	17	48615501	48615501	G	A
MEN0042-P0	SEMA4A	snp	1	156146507	156146507	A	G
MEN0042-P0	LUZP2	snp	11	24753676	24753676	G	A
MEN0042-P0	SYNE1	snp	6	152615187	152615187	C	T

MEN0042-P0	ZNF71	snp	19	57133555	57133555	C	G
MEN0042-P0	PRB2	snp	12	11546645	11546645	G	C
MEN0042-P0	KRTAP4-11	snp	17	39274087	39274087	G	C
MEN0042-P0	ATXN1	snp	6	16327897	16327897	C	A
MEN0042-P0	PCLO	snp	7	82784471	82784471	A	G
MEN0042-P0	SEMA4A	snp	1	156146507	156146507	A	G
MEN0042-P0	POTEC	snp	18	14542978	14542978	C	T
MEN0042-P0	FOXP2	snp	7	114293993	114293993	C	T
MEN0042-P0	IGKV1-17	snp	2	89416981	89416981	C	T
MEN0042-P0	NBPF9	snp	1	144828707	144828707	G	T
MEN0042-P0	EPN3	snp	17	48615501	48615501	G	A
MEN0042-P0	MDK	snp	11	46404331	46404331	C	G
MEN0042-P0	STAT6	snp	12	57493645	57493645	A	C
MEN0042-P0	SRGAP1	snp	12	64383808	64383808	C	T
MEN0042-P0	LPAR2	snp	19	19737520	19737520	C	T
MEN0042-P0	SWT1	snp	1	185153955	185153955	C	T
MEN0042-P0	FRG1B	snp	20	29623231	29623231	C	T
MEN0042-P0	UBR5	snp	8	103338877	103338877	A	C
MEN0042-P0	MAPK10	snp	4	87080596	87080596	A	G
MEN0042-P0	AC109135.1	snp	GL000192.1	311344	311344	C	T
MEN0042-P0	SCNN1G	snp	16	23226732	23226732	G	A
MEN0042-P0	AL592183.1	snp	GL000219.1	83290	83290	C	T
MEN0042-P0	DHRS3	snp	1	12640591	12640591	C	T
MEN0042-P0	TMEM14E	snp	3	152058521	152058521	C	T
MEN0042-P0	LUZP2	snp	11	24753676	24753676	G	A
MEN0042-P0	FRG1B	snp	20	29625934	29625934	C	T
MEN0042-P0	NBPF9	snp	1	144828683	144828683	C	G
MEN0042-P0	ZNF844	snp	19	12187394	12187394	T	C
MEN0042-P0	POTEC	snp	18	14513675	14513675	T	C
MEN0042-P0	POTEC	snp	18	14513675	14513675	T	C
MEN0042-P0	IGF2BP3	snp	7	23352422	23352422	G	A
MEN0042-P0	LYST	snp	1	235922642	235922642	C	T
MEN0042-P0	SLC9A5	snp	16	67289037	67289037	C	G
MEN0042-P0	SYNE1	snp	6	152615187	152615187	C	T
MEN0042-P0	PCSK5	snp	9	78790187	78790187	G	C
MEN0042-P0	FSCB	snp	14	44975827	44975827	G	T
MEN0042-P0	RREB1	snp	6	7229893	7229893	A	C
MEN0042-P0	NBPF10	snp	1	145304613	145304613	T	C
MEN0042-P0	NBPF9	snp	1	144828599	144828599	G	A
MEN0042-P0	ANKRD30B	snp	18	14779986	14779986	G	A
MEN0042-P0	FLG	snp	1	152275960	152275960	T	C
MEN0042-P0	FLG	snp	1	152281113	152281113	G	C
MEN0042-P0	NBPF10	snp	1	145296448	145296448	T	A
MEN0042-P0	KRTAP4-11	snp	17	39274087	39274087	G	C
MEN0042-P0	TMIGD1	snp	17	28651971	28651971	C	T
MEN0042-P0	SVIL	snp	10	29770552	29770552	T	G
MEN0042-P0	ZNF71	snp	19	57133555	57133555	C	G

MEN0042-P0	ANKRD36	snp	2	97869931	97869931	A	T
MEN0042-P0	SLC27A5	snp	19	59011977	59011977	G	A
MEN0042-P0	NBPF10	snp	1	145323690	145323690	A	G
MEN0042-P0	PRB2	snp	12	11546645	11546645	G	C
MEN0042-P0	PCLO	snp	7	82784471	82784471	A	G
MEN0042-P0	TCP10L2	snp	6	167591954	167591954	G	A
MEN0042-P0	ATXN1	snp	6	16327897	16327897	C	A
MEN0042-P2	NBPF9	snp	1	144828707	144828707	G	T
MEN0042-P2	PLP2	snp	X	49029787	49029787	T	C
MEN0042-P2	LPAR2	snp	19	19737520	19737520	C	T
MEN0042-P2	CTSW	snp	11	65649985	65649985	G	A
MEN0042-P2	CRB2	snp	9	126133531	126133531	C	T
MEN0042-P2	RANBP2	snp	2	109371685	109371685	A	T
MEN0042-P2	FRG1B	snp	20	29623231	29623231	C	T
MEN0042-P2	NBPF10	snp	1	145296448	145296448	T	A
MEN0042-P2	AL592183.1	snp	GL000219.1	83290	83290	C	T
MEN0042-P2	MRPL45	snp	17	36453192	36453192	T	G
MEN0042-P2	PRKDC	snp	8	48794481	48794481	T	C
MEN0042-P2	NBPF9	snp	1	144828683	144828683	C	G
MEN0042-P2	RANBP2	snp	2	109352660	109352660	C	T
MEN0042-P2	SLC27A5	snp	19	59011977	59011977	G	A
MEN0042-P2	PDE1B	snp	12	54966512	54966512	G	A
MEN0042-P2	SUMF2	snp	7	56146128	56146128	C	T
MEN0042-P2	PARG	snp	10	51093329	51093329	C	T
MEN0042-P2	BARD1	snp	2	215645445	215645445	C	T
MEN0042-P2	ANKRD30B	snp	18	14779986	14779986	G	A
MEN0042-P2	ADCY4	snp	14	24799146	24799146	G	A
MEN0042-P2	CILP	snp	15	65490150	65490150	A	C
MEN0042-P2	PCSK5	snp	9	78790187	78790187	G	C
MEN0042-P2	CBWD1	snp	9	121528	121528	G	A
MEN0042-P2	LINGO1	snp	15	77907606	77907606	G	T
MEN0042-P2	IGKV1-17	snp	2	89416981	89416981	C	T
MEN0042-P2	KLK15	snp	19	51331044	51331044	T	G
MEN0042-P2	UBR5	snp	8	103338877	103338877	A	C
MEN0042-P2	MAPK10	snp	4	87080596	87080596	A	G
MEN0042-P2	CYP4V2	snp	4	187126450	187126450	G	A
MEN0042-P2	GON4L	snp	1	155720514	155720514	C	T
MEN0042-P2	STAT6	snp	12	57493645	57493645	A	C
MEN0042-P2	CNOT1	snp	16	58577512	58577512	C	T
MEN0042-P2	DHRS3	snp	1	12640591	12640591	C	T
MEN0042-P2	SWT1	snp	1	185153955	185153955	C	T
MEN0042-P2	SLC9A5	snp	16	67289037	67289037	C	G
MEN0042-P2	TMIGD1	snp	17	28651971	28651971	C	T
MEN0042-P2	PCDH1	snp	5	141248193	141248193	G	A
MEN0042-P2	FRG1B	snp	20	29625947	29625947	T	C
MEN0042-P2	ADAMTS7	snp	15	79059041	79059041	T	C
MEN0042-P2	RBAK	snp	7	5103628	5103628	A	T

MEN0042-P2	DHX33	snp	17	5358584	5358584	C	T
MEN0042-P2	TAOK1	snp	17	27837991	27837991	G	A
MEN0042-P2	SLC1A7	snp	1	53554566	53554566	G	A
MEN0042-P2	SVIL	snp	10	29770552	29770552	T	G
MEN0042-P2	BOC	snp	3	112997000	112997000	G	C
MEN0042-P2	OR1B1	snp	9	125390908	125390908	C	A
MEN0042-P2	FOXP2	snp	7	114293993	114293993	C	T
MEN0042-P2	PI4KA	snp	22	21098962	21098962	G	T
MEN0042-P2	TMEM14E	snp	3	152058521	152058521	C	T
MEN0042-P2	IRF2	snp	4	185340717	185340717	C	T
MEN0042-P2	SCNN1G	snp	16	23226732	23226732	G	A
MEN0042-P2	FSCB	snp	14	44975827	44975827	G	T
MEN0042-P2	CUBN	snp	10	16870953	16870953	A	C
MEN0042-P2	KMT2D	snp	12	49446827	49446827	G	A
MEN0042-P2	MT-ND4	snp	MT	12038	12038	A	G
MEN0042-P2	KMT2C	snp	7	151970940	151970940	A	G
MEN0042-P2	SEMA4A	snp	1	156146507	156146507	A	G
MEN0042-P2	LUZP2	snp	11	24753676	24753676	G	A
MEN0042-P2	ZNF71	snp	19	57133555	57133555	C	G
MEN0042-P2	PRB2	snp	12	11546645	11546645	G	C
MEN0042-P2	KRTAP4-11	snp	17	39274087	39274087	G	C
MEN0042-P2	ZNF99	snp	19	22939101	22939101	C	T
MEN0042-P2	ZNF429	snp	19	21720452	21720452	G	A
MEN0042-P2	KRTAP5-10	snp	11	71276851	71276851	A	G
MEN0042-P2	TTN	snp	2	179424506	179424506	T	G
MEN0042-P2	PCLO	snp	7	82784471	82784471	A	G
MEN0042-P2	LINGO1	snp	15	77907606	77907606	G	T
MEN0042-P2	SEMA4A	snp	1	156146507	156146507	A	G
MEN0042-P2	PI4KA	snp	22	21098962	21098962	G	T
MEN0042-P2	FOXP2	snp	7	114293993	114293993	C	T
MEN0042-P2	IGKV1-17	snp	2	89416981	89416981	C	T
MEN0042-P2	NBPF9	snp	1	144828707	144828707	G	T
MEN0042-P2	ADCY4	snp	14	24799146	24799146	G	A
MEN0042-P2	KMT2C	snp	7	151970940	151970940	A	G
MEN0042-P2	STAT6	snp	12	57493645	57493645	A	C
MEN0042-P2	ZNF429	snp	19	21720452	21720452	G	A
MEN0042-P2	CNOT1	snp	16	58577512	58577512	C	T
MEN0042-P2	CILP	snp	15	65490150	65490150	A	C
MEN0042-P2	LPAR2	snp	19	19737520	19737520	C	T
MEN0042-P2	SWT1	snp	1	185153955	185153955	C	T
MEN0042-P2	FRG1B	snp	20	29623231	29623231	C	T
MEN0042-P2	UBR5	snp	8	103338877	103338877	A	C
MEN0042-P2	MT-ND4	snp	MT	12038	12038	A	G
MEN0042-P2	CRB2	snp	9	126133531	126133531	C	T
MEN0042-P2	DHX33	snp	17	5358584	5358584	C	T
MEN0042-P2	PRKDC	snp	8	48794481	48794481	T	C
MEN0042-P2	TTN	snp	2	179424506	179424506	T	G

MEN0042-P2	CTSW	snp	11	65649985	65649985	G	A
MEN0042-P2	CBWD1	snp	9	121528	121528	G	A
MEN0042-P2	RANBP2	snp	2	109352660	109352660	C	T
MEN0042-P2	MAPK10	snp	4	87080596	87080596	A	G
MEN0042-P2	SCNN1G	snp	16	23226732	23226732	G	A
MEN0042-P2	AL592183.1	snp	GL000219.1	83290	83290	C	T
MEN0042-P2	OR1B1	snp	9	125390908	125390908	C	A
MEN0042-P2	CUBN	snp	10	16870953	16870953	A	C
MEN0042-P2	BARD1	snp	2	215645445	215645445	C	T
MEN0042-P2	DHRS3	snp	1	12640591	12640591	C	T
MEN0042-P2	BOC	snp	3	112997000	112997000	G	C
MEN0042-P2	MRPL45	snp	17	36453192	36453192	T	G
MEN0042-P2	TMEM14E	snp	3	152058521	152058521	C	T
MEN0042-P2	ADAMTS7	snp	15	79059041	79059041	T	C
MEN0042-P2	PLP2	snp	X	49029787	49029787	T	C
MEN0042-P2	KMT2D	snp	12	49446827	49446827	G	A
MEN0042-P2	GON4L	snp	1	155720514	155720514	C	T
MEN0042-P2	SLC1A7	snp	1	53554566	53554566	G	A
MEN0042-P2	LUZP2	snp	11	24753676	24753676	G	A
MEN0042-P2	NBPF9	snp	1	144828683	144828683	C	G
MEN0042-P2	PARG	snp	10	51093329	51093329	C	T
MEN0042-P2	RANBP2	snp	2	109371685	109371685	A	T
MEN0042-P2	KLK15	snp	19	51331044	51331044	T	G
MEN0042-P2	PDE1B	snp	12	54966512	54966512	G	A
MEN0042-P2	SLC9A5	snp	16	67289037	67289037	C	G
MEN0042-P2	SUMF2	snp	7	56146128	56146128	C	T
MEN0042-P2	IRF2	snp	4	185340717	185340717	C	T
MEN0042-P2	PCSK5	snp	9	78790187	78790187	G	C
MEN0042-P2	FSCB	snp	14	44975827	44975827	G	T
MEN0042-P2	RBAK	snp	7	5103628	5103628	A	T
MEN0042-P2	ANKRD30B	snp	18	14779986	14779986	G	A
MEN0042-P2	NBPF10	snp	1	145296448	145296448	T	A
MEN0042-P2	TAOK1	snp	17	27837991	27837991	G	A
MEN0042-P2	ZNF99	snp	19	22939101	22939101	C	T
MEN0042-P2	KRTAP4-11	snp	17	39274087	39274087	G	C
MEN0042-P2	TMIGD1	snp	17	28651971	28651971	C	T
MEN0042-P2	PCDH1	snp	5	141248193	141248193	G	A
MEN0042-P2	SVIL	snp	10	29770552	29770552	T	G
MEN0042-P2	ZNF71	snp	19	57133555	57133555	C	G
MEN0042-P2	SLC27A5	snp	19	59011977	59011977	G	A
MEN0042-P2	FRG1B	snp	20	29625947	29625947	T	C
MEN0042-P2	CYP4V2	snp	4	187126450	187126450	G	A
MEN0042-P2	PRB2	snp	12	11546645	11546645	G	C
MEN0042-P2	PCLO	snp	7	82784471	82784471	A	G
MEN0042-P2	KRTAP5-10	snp	11	71276851	71276851	A	G
MEN0042G-P1	OR2T34	snp	1	248737419	248737419	T	C
MEN0042G-P1	LPAR2	snp	19	19737520	19737520	C	T

MEN0042G-P1	PDXDC1	snp	16	15100351	15100351	G	A
MEN0042G-P1	ANKRD18A	snp	9	38588630	38588630	G	C
MEN0042G-P1	SLC27A5	snp	19	59011977	59011977	G	A
MEN0042G-P1	ZNF582	snp	19	56896215	56896215	C	T
MEN0042G-P1	IGKV1-17	snp	2	89416981	89416981	C	T
MEN0042G-P1	UBR5	snp	8	103338877	103338877	A	C
MEN0042G-P1	MAPK10	snp	4	87080596	87080596	A	G
MEN0042G-P1	STAT6	snp	12	57493645	57493645	A	C
MEN0042G-P1	DHRS3	snp	1	12640591	12640591	C	T
MEN0042G-P1	SWT1	snp	1	185153955	185153955	C	T
MEN0042G-P1	SLC9A5	snp	16	67289037	67289037	C	G
MEN0042G-P1	NBPF24	snp	1	147580791	147580791	T	C
MEN0042G-P1	TMIGD1	snp	17	28651971	28651971	C	T
MEN0042G-P1	SVIL	snp	10	29770552	29770552	T	G
MEN0042G-P1	FOXP2	snp	7	114293993	114293993	C	T
MEN0042G-P1	TMEM14E	snp	3	152058521	152058521	C	T
MEN0042G-P1	NPIPB6	snp	16	28354418	28354418	C	T
MEN0042G-P1	SCNN1G	snp	16	23226732	23226732	G	A
MEN0042G-P1	FSCB	snp	14	44975827	44975827	G	T
MEN0042G-P1	ZAN	snp	7	100350126	100350126	G	A
MEN0042G-P1	AL356585.1	snp	GL000212.1	64910	64910	A	C
MEN0042G-P1	LUZP2	snp	11	24753676	24753676	G	A
MEN0042G-P1	ZNF71	snp	19	57133555	57133555	C	G
MEN0042G-P1	PRG4	snp	1	186276040	186276040	G	T
MEN0042G-P1	FOXP2	snp	7	114293993	114293993	C	T
MEN0042G-P1	IGKV1-17	snp	2	89416981	89416981	C	T
MEN0042G-P1	ANKRD18A	snp	9	38588630	38588630	G	C
MEN0042G-P1	ANKRD18A	snp	9	38588630	38588630	G	C
MEN0042G-P1	NPIPB6	snp	16	28354418	28354418	C	T
MEN0042G-P1	PDXDC1	snp	16	15100351	15100351	G	A
MEN0042G-P1	STAT6	snp	12	57493645	57493645	A	C
MEN0042G-P1	OR2T34	snp	1	248737419	248737419	T	C
MEN0042G-P1	LPAR2	snp	19	19737520	19737520	C	T
MEN0042G-P1	SWT1	snp	1	185153955	185153955	C	T
MEN0042G-P1	UBR5	snp	8	103338877	103338877	A	C
MEN0042G-P1	MAPK10	snp	4	87080596	87080596	A	G
MEN0042G-P1	AL356585.1	snp	GL000212.1	64910	64910	A	C
MEN0042G-P1	SCNN1G	snp	16	23226732	23226732	G	A
MEN0042G-P1	DHRS3	snp	1	12640591	12640591	C	T
MEN0042G-P1	NBPF24	snp	1	147580791	147580791	T	C
MEN0042G-P1	ZNF582	snp	19	56896215	56896215	C	T
MEN0042G-P1	TMEM14E	snp	3	152058521	152058521	C	T
MEN0042G-P1	LUZP2	snp	11	24753676	24753676	G	A
MEN0042G-P1	SLC9A5	snp	16	67289037	67289037	C	G
MEN0042G-P1	FSCB	snp	14	44975827	44975827	G	T
MEN0042G-P1	TMIGD1	snp	17	28651971	28651971	C	T
MEN0042G-P1	SVIL	snp	10	29770552	29770552	T	G

MEN0042G-P1	ZNF71	snp	19	57133555	57133555	C	G
MEN0042G-P1	SLC27A5	snp	19	59011977	59011977	G	A
MEN0042G-P1	ZAN	snp	7	100350126	100350126	G	A
MEN0042G-P1	PRG4	snp	1	186276040	186276040	G	T
MEN0045-P0	RGPD3	snp	2	107049681	107049681	TRUE	C
MEN0045-P0	RGPD3	snp	2	107049681	107049681	TRUE	C
MEN0045-P1	MYH1	snp	17	10412854	10412854	G	A
MEN0045-P1	RGPD3	snp	2	107049681	107049681	T	C
MEN0045-P1	MYH1	snp	17	10412854	10412854	G	A
MEN0045-P1	RGPD3	snp	2	107049681	107049681	T	C
MEN0045-P2	RGPD3	snp	2	107049681	107049681	T	C
MEN0045-P2	PTCH1	snp	9	98268727	98268727	G	A
MEN0045-P2	PTCH1	snp	9	98268727	98268727	G	A
MEN0045-P2	RGPD3	snp	2	107049681	107049681	T	C
MEN0045-P3	LRP2	snp	2	170092519	170092519	A	C
MEN0045-P3	KMT2C	snp	7	151875009	151875009	A	C
MEN0045-P3	KMT2C	snp	7	151875009	151875009	A	C
MEN0045-P3	LRP2	snp	2	170092519	170092519	A	C
MEN0045-P4	FLNB	snp	3	58124189	58124189	C	G
MEN0045-P4	FLNB	snp	3	58124189	58124189	C	G
MEN0048-P0	MAL	snp	2	95719159	95719159	G	T
MEN0048-P0	BMS1	snp	10	43318566	43318566	G	A
MEN0048-P0	DAXX	snp	6	33289272	33289272	G	A
MEN0048-P0	FAM47A	snp	X	34149266	34149266	G	A
MEN0048-P0	RAB33A	snp	X	129318346	129318346	G	A
MEN0048-P0	RHPN2	snp	19	33493188	33493188	T	A
MEN0048-P0	TEX101	snp	19	43922422	43922422	C	T
MEN0048-P0	NBPF9	snp	1	144828707	144828707	G	T
MEN0048-P0	CHP2	snp	16	23767187	23767187	A	T
MEN0048-P0	ATP6V1H	snp	8	54708292	54708292	C	T
MEN0048-P0	RHPN2	snp	19	33493201	33493201	C	T
MEN0048-P0	ELTD1	snp	1	79357261	79357261	T	C
MEN0048-P0	NBPF10	snp	1	145302745	145302745	A	T
MEN0048-P0	NBPF10	snp	1	145296448	145296448	T	A
MEN0048-P0	MUC16	snp	19	9056757	9056757	G	T
MEN0048-P0	BTK	snp	X	100609669	100609669	C	G
MEN0048-P0	EVPL	snp	17	74003956	74003956	G	A
MEN0048-P0	STON2	snp	14	81744954	81744954	G	T
MEN0048-P0	NRSN1	snp	6	24146146	24146146	A	G
MEN0048-P0	ZNF208	snp	19	22155114	22155114	C	T
MEN0048-P0	MPP1	snp	X	154020552	154020552	C	A
MEN0048-P0	SOWAHD	snp	X	118893556	118893556	T	G
MEN0048-P0	TBC1D14	snp	4	6925486	6925486	G	A
MEN0048-P0	CRAMP1L	snp	16	1705221	1705221	A	G
MEN0048-P0	EGR1	snp	5	137803490	137803490	C	T
MEN0048-P0	GPR113	snp	2	26536256	26536256	T	A
MEN0048-P0	PRPF6	snp	20	62630479	62630479	G	A

MEN0048-P0	SLC26A11	snp	17	78210753	78210753	G	A
MEN0048-P0	FZD8	snp	10	35930289	35930289	G	C
MEN0048-P0	AGL	snp	1	100356880	100356880	C	T
MEN0048-P0	MMADHC	snp	2	150438740	150438740	A	C
MEN0048-P0	NLRP3	snp	1	247597517	247597517	G	A
MEN0048-P0	ZNF91	snp	19	23544783	23544783	C	T
MEN0048-P0	F5	snp	1	169510354	169510354	A	G
MEN0048-P0	KCNQ3	snp	8	133196524	133196524	G	C
MEN0048-P0	CD93	snp	20	23065005	23065005	G	A
MEN0048-P0	PHYKPL	snp	5	177651695	177651695	A	C
MEN0048-P0	NCR3	snp	6	31557386	31557386	G	A
MEN0048-P0	PRDM15	snp	21	43222949	43222949	C	T
MEN0048-P0	TNNI3K	snp	1	75009602	75009602	A	T
MEN0048-P0	MUC17	snp	7	100682505	100682505	T	C
MEN0048-P0	PPP1R32	snp	11	61254481	61254481	G	A
MEN0048-P0	LRRTM3	snp	10	68687485	68687485	C	T
MEN0048-P0	PAPPA2	snp	1	176681002	176681002	A	C
MEN0048-P0	TP53	snp	17	7578371	7578371	C	T
MEN0048-P0	NBPF9	snp	1	144828683	144828683	C	G
MEN0048-P0	MUC17	snp	7	100682244	100682244	T	C
MEN0048-P0	ZNF347	snp	19	53643812	53643812	C	T
MEN0048-P0	DHX9	snp	1	182845645	182845645	T	A
MEN0048-P0	MUC17	snp	7	100682680	100682680	G	C
MEN0048-P0	KRT37	snp	17	39580519	39580519	G	A
MEN0048-P0	KLC2	snp	11	66034372	66034372	G	A
MEN0048-P0	UNC79	snp	14	94110009	94110009	C	A
MEN0048-P0	KMT2C	snp	7	151874763	151874763	T	C
MEN0048-P0	SHROOM4	snp	X	50377185	50377185	G	A
MEN0048-P0	HAO2	snp	1	119936413	119936413	C	T
MEN0048-P0	BRWD1	snp	21	40559073	40559073	A	C
MEN0048-P0	LTBP1	snp	2	33500964	33500964	G	A
MEN0048-P0	ZNF708	snp	19	21477046	21477046	C	T
MEN0048-P0	ZNF117	snp	7	64439383	64439383	T	C
MEN0048-P0	ZBTB20	snp	3	114069919	114069919	C	T
MEN0048-P0	ZNF808	snp	19	53058687	53058687	A	C
MEN0048-P0	NBPF10	snp	1	145299906	145299906	T	G
MEN0048-P0	FAM50A	snp	X	153678412	153678412	C	T
MEN0048-P0	SKIV2L	snp	6	31937383	31937383	G	A
MEN0048-P0	NOS2	snp	17	26109120	26109120	G	C
MEN0048-P0	STAB1	snp	3	52556589	52556589	T	C
MEN0048-P0	WDR33	snp	2	128525825	128525825	C	G
MEN0048-P0	MDGA2	snp	14	47342740	47342740	C	T
MEN0048-P0	STARD10	snp	11	72466113	72466113	C	T
MEN0048-P0	ANKRD36C	snp	2	96593016	96593016	C	A
MEN0048-P0	ZNF548	snp	19	57911082	57911082	C	T
MEN0048-P0	FKBP4	snp	12	2910467	2910467	G	A
MEN0048-P0	KIAA1009	snp	6	84896154	84896154	C	T

MEN0048-P0	ZNF721	snp	4	437235	437235	T	C
MEN0048-P0	ATP9A	snp	20	50286571	50286571	C	T
MEN0048-P0	AGBL5	snp	2	27292478	27292478	G	A
MEN0048-P0	RPTN	snp	1	152127897	152127897	C	T
MEN0048-P0	TAP1	snp	6	32816469	32816469	G	A
MEN0048-P0	DNAJC13	snp	3	132244556	132244556	A	G
MEN0048-P0	TUBB6	snp	18	12325395	12325395	G	A
MEN0048-P0	CNTNAP3B	snp	9	43915893	43915893	G	C
MEN0048-P0	IL3	snp	5	131398080	131398080	G	C
MEN0048-P0	ZNF267	snp	16	31927702	31927702	G	C
MEN0048-P0	UNC79	snp	14	94109916	94109916	G	T
MEN0048-P0	GLP2R	snp	17	9764491	9764491	G	A
MEN0048-P0	ZNF43	snp	19	21990830	21990830	A	C
MEN0048-P0	IL3RA	snp	X	1471267	1471267	C	T
MEN0048-P0	CACNA1B	snp	9	140991050	140991050	G	A
MEN0048-P0	ZNF799	snp	19	12501446	12501446	T	C
MEN0048-P0	ZNF658	snp	9	40773116	40773116	T	C
MEN0048-P0	CPNE4	snp	3	131261507	131261507	C	T
MEN0048-P0	C11orf49	snp	11	47176747	47176747	C	T
MEN0048-P0	BRINP3	snp	1	190067216	190067216	C	A
MEN0048-P0	C3	snp	19	6713269	6713269	G	T
MEN0048-P0	ACSL6	snp	5	131308476	131308476	G	A
MEN0048-P0	AGRN	snp	1	981113	981113	C	A
MEN0048-P0	ZNF267	snp	16	31927690	31927690	G	A
MEN0048-P0	MAGEA4	snp	X	151092594	151092594	G	A
MEN0048-P0	DSP	snp	6	7585620	7585620	G	A
MEN0048-P0	ZNF776	snp	19	58265928	58265928	G	A
MEN0048-P0	ZFHX3	snp	16	72845884	72845884	G	A
MEN0048-P0	KRTAP4-11	snp	17	39274206	39274206	C	T
MEN0048-P0	ZNF431	snp	19	21366698	21366698	G	A
MEN0048-P0	ZNF85	snp	19	21132292	21132292	G	C
MEN0048-P0	CREBZF	snp	11	85375769	85375769	G	A
MEN0048-P0	ZNF616	snp	19	52618845	52618845	T	A
MEN0048-P0	INO80	snp	15	41348900	41348900	G	A
MEN0048-P0	KCNJ4	snp	22	38824062	38824062	C	T
MEN0048-P0	WDR90	snp	16	708627	708627	G	A
MEN0048-P0	RHOU	snp	1	228871647	228871647	T	C
MEN0048-P0	ZNF85	snp	19	21132615	21132615	A	G
MEN0048-P0	ZNF585B	snp	19	37676173	37676173	C	T
MEN0048-P0	REL	snp	2	61149606	61149606	G	A
MEN0048-P0	PCLO	snp	7	82784366	82784366	A	G
MEN0048-P0	LOXL2	snp	8	23225710	23225710	T	C
MEN0048-P0	ESX1	snp	X	103499160	103499160	C	T
MEN0048-P0	CAPN7	snp	3	15282019	15282019	T	G
MEN0048-P0	MRPL45	snp	17	36453192	36453192	T	G
MEN0048-P0	POLR2A	snp	17	7404938	7404938	G	A
MEN0048-P0	TUBGCP5	snp	15	22846940	22846940	G	A

MEN0048-P0	KIAA0556	snp	16	27761387	27761387	G	A
MEN0048-P0	ZNF780B	snp	19	40541934	40541934	G	C
MEN0048-P0	ACTG1	snp	17	79477737	79477737	G	C
MEN0048-P0	AHNAK	snp	11	62295870	62295870	T	C
MEN0048-P0	ZNF724P	snp	19	23405462	23405462	C	T
MEN0048-P0	THOC2	snp	X	122774430	122774430	C	A
MEN0048-P0	ZNF431	snp	19	21366366	21366366	G	A
MEN0048-P0	ZNF138	snp	7	64292444	64292444	A	G
MEN0048-P0	ZNF91	snp	19	23544222	23544222	A	C
MEN0048-P0	KLHL6	snp	3	183226244	183226244	G	T
MEN0048-P0	ALB	snp	4	74270854	74270854	G	A
MEN0048-P0	DNAH1	snp	3	52383403	52383403	C	G
MEN0048-P0	ZAN	snp	7	100350052	100350052	T	C
MEN0048-P0	SIGLEC12	snp	19	52000192	52000192	A	G
MEN0048-P0	ANKRD36C	snp	2	96593025	96593025	C	T
MEN0048-P0	ZNF619	snp	3	40529017	40529017	A	C
MEN0048-P0	EFHC1	snp	6	52303267	52303267	C	T
MEN0048-P0	ZNF721	snp	4	436421	436421	A	G
MEN0048-P0	ZNF681	snp	19	23927195	23927195	A	C
MEN0048-P0	PCSK5	snp	9	78790192	78790192	C	G
MEN0048-P0	ENAM	snp	4	71509091	71509091	C	T
MEN0048-P0	LIG3	snp	17	33310377	33310377	G	A
MEN0048-P0	ZAN	snp	7	100350361	100350361	T	C
MEN0048-P0	SRF	snp	6	43146157	43146157	G	A
MEN0048-P0	KRTAP4-11	snp	17	39274150	39274150	T	A
MEN0048-P0	ANKLE1	snp	19	17397493	17397493	G	T
MEN0048-P0	ZNF678	snp	1	227843257	227843257	A	G
MEN0048-P0	ZNF117	snp	7	64439050	64439050	C	G
MEN0048-P0	ZNF678	snp	1	227842562	227842562	G	C
MEN0048-P0	SLC16A10	snp	6	111543299	111543299	C	T
MEN0048-P0	POM121	snp	7	72400489	72400489	T	C
MEN0048-P0	AHNAK	snp	11	62293095	62293095	C	T
MEN0048-P0	WDR86	snp	7	151093196	151093196	C	T
MEN0048-P0	HLA-C	snp	6	31239513	31239513	C	T
MEN0048-P0	FAM86C1	snp	11	71507261	71507261	A	G
MEN0048-P0	6-Sep	snp	X	118767441	118767441	T	C
MEN0048-P0	PLD5	snp	1	242253187	242253187	T	A
MEN0048-P0	ZNF708	snp	19	21476519	21476519	T	C
MEN0048-P0	ZNF107	snp	7	64168392	64168392	A	T
MEN0048-P0	ZNF592	snp	15	85326684	85326684	C	G
MEN0048-P0	ZNF107	snp	7	64168681	64168681	A	G
MEN0048-P0	ZNF708	snp	19	21476689	21476689	T	C
MEN0048-P0	ZNF91	snp	19	23543296	23543296	T	C
MEN0048-P0	ZNF85	snp	19	21132356	21132356	A	G
MEN0048-P0	ZNF100	snp	19	21909734	21909734	G	C
MEN0048-P0	ZNF91	snp	19	23543800	23543800	T	C
MEN0048-P0	ZNF208	snp	19	22154249	22154249	A	G

MEN0048-P0	FZD8	snp	10	35930289	35930289	G	C
MEN0048-P0	MMADHC	snp	2	150438740	150438740	A	C
MEN0048-P0	MMADHC	snp	2	150438740	150438740	A	C
MEN0048-P0	MMADHC	snp	2	150438740	150438740	A	C
MEN0048-P0	ZNF208	snp	19	22155114	22155114	C	T
MEN0048-P0	MAL	snp	2	95719159	95719159	G	T
MEN0048-P0	GPR113	snp	2	26536256	26536256	T	A
MEN0048-P0	ZNF267	snp	16	31927690	31927690	G	A
MEN0048-P0	CNTNAP3B	snp	9	43915893	43915893	G	C
MEN0048-P0	CD93	snp	20	23065005	23065005	G	A
MEN0048-P0	SLC16A10	snp	6	111543299	111543299	C	T
MEN0048-P0	CRAMP1L	snp	16	1705221	1705221	A	G
MEN0048-P0	ZNF708	snp	19	21476689	21476689	T	C
MEN0048-P0	BTK	snp	X	100609669	100609669	C	G
MEN0048-P0	CAPN7	snp	3	15282019	15282019	T	G
MEN0048-P0	RHOU	snp	1	228871647	228871647	T	C
MEN0048-P0	UNC79	snp	14	94110009	94110009	C	A
MEN0048-P0	WDR33	snp	2	128525825	128525825	C	G
MEN0048-P0	BMS1	snp	10	43318566	43318566	G	A
MEN0048-P0	MRPL45	snp	17	36453192	36453192	T	G
MEN0048-P0	MRPL45	snp	17	36453192	36453192	T	G
MEN0048-P0	MRPL45	snp	17	36453192	36453192	T	G
MEN0048-P0	KCNQ3	snp	8	133196524	133196524	G	C
MEN0048-P0	ZNF117	snp	7	64439383	64439383	T	C
MEN0048-P0	CREBZF	snp	11	85375769	85375769	G	A
MEN0048-P0	LOXL2	snp	8	23225710	23225710	T	C
MEN0048-P0	FAM86C1	snp	11	71507261	71507261	A	G
MEN0048-P0	NCR3	snp	6	31557386	31557386	G	A
MEN0048-P0	BRWD1	snp	21	40559073	40559073	A	C
MEN0048-P0	AGL	snp	1	100356880	100356880	C	T
MEN0048-P0	IL3	snp	5	131398080	131398080	G	C
MEN0048-P0	ZNF721	snp	4	437235	437235	T	C
MEN0048-P0	EFHC1	snp	6	52303267	52303267	C	T
MEN0048-P0	SRF	snp	6	43146157	43146157	G	A
MEN0048-P0	NBPF9	snp	1	144828707	144828707	G	T
MEN0048-P0	ZNF347	snp	19	53643812	53643812	C	T
MEN0048-P0	CHP2	snp	16	23767187	23767187	A	T
MEN0048-P0	SOWAHD	snp	X	118893556	118893556	T	G
MEN0048-P0	SIGLEC12	snp	19	52000192	52000192	A	G
MEN0048-P0	ZNF658	snp	9	40773116	40773116	T	C
MEN0048-P0	ZNF43	snp	19	21990830	21990830	A	C
MEN0048-P0	SLC26A11	snp	17	78210753	78210753	G	A
MEN0048-P0	TUBGCP5	snp	15	22846940	22846940	G	A
MEN0048-P0	AGBL5	snp	2	27292478	27292478	G	A
MEN0048-P0	ZNF85	snp	19	21132292	21132292	G	C
MEN0048-P0	ZNF681	snp	19	23927195	23927195	A	C
MEN0048-P0	ZNF107	snp	7	64168392	64168392	A	T

MEN0048-P0	PRPF6	snp	20	62630479	62630479	G	A
MEN0048-P0	ACSL6	snp	5	131308476	131308476	G	A
MEN0048-P0	KCNJ4	snp	22	38824062	38824062	C	T
MEN0048-P0	ZNF91	snp	19	23544783	23544783	C	T
MEN0048-P0	SKIV2L	snp	6	31937383	31937383	G	A
MEN0048-P0	EGR1	snp	5	137803490	137803490	C	T
MEN0048-P0	MUC16	snp	19	9056757	9056757	G	T
MEN0048-P0	NOS2	snp	17	26109120	26109120	G	C
MEN0048-P0	TNNI3K	snp	1	75009602	75009602	A	T
MEN0048-P0	FKBP4	snp	12	2910467	2910467	G	A
MEN0048-P0	RHPN2	snp	19	33493201	33493201	C	T
MEN0048-P0	ZNF107	snp	7	64168681	64168681	A	G
MEN0048-P0	TEX101	snp	19	43922422	43922422	C	T
MEN0048-P0	BRINP3	snp	1	190067216	190067216	C	A
MEN0048-P0	INO80	snp	15	41348900	41348900	G	A
MEN0048-P0	RAB33A	snp	X	129318346	129318346	G	A
MEN0048-P0	MUC17	snp	7	100682244	100682244	T	C
MEN0048-P0	ZNF708	snp	19	21477046	21477046	C	T
MEN0048-P0	ELTD1	snp	1	79357261	79357261	T	C
MEN0048-P0	CACNA1B	snp	9	140991050	140991050	G	A
MEN0048-P0	PHYKPL	snp	5	177651695	177651695	A	C
MEN0048-P0	ZNF678	snp	1	227842562	227842562	G	C
MEN0048-P0	ZNF431	snp	19	21366698	21366698	G	A
MEN0048-P0	C3	snp	19	6713269	6713269	G	T
MEN0048-P0	ZNF799	snp	19	12501446	12501446	T	C
MEN0048-P0	STARD10	snp	11	72466113	72466113	C	T
MEN0048-P0	PLD5	snp	1	242253187	242253187	T	A
MEN0048-P0	LRRTM3	snp	10	68687485	68687485	C	T
MEN0048-P0	PCLO	snp	7	82784366	82784366	A	G
MEN0048-P0	WDR90	snp	16	708627	708627	G	A
MEN0048-P0	EVPL	snp	17	74003956	74003956	G	A
MEN0048-P0	NBPF10	snp	1	145302745	145302745	A	T
MEN0048-P0	IL3RA	snp	X	1471267	1471267	C	T
MEN0048-P0	STAB1	snp	3	52556589	52556589	T	C
MEN0048-P0	ZNF91	snp	19	23544222	23544222	A	C
MEN0048-P0	ZNF592	snp	15	85326684	85326684	C	G
MEN0048-P0	POM121	snp	7	72400489	72400489	T	C
MEN0048-P0	HLA-C	snp	6	31239513	31239513	C	T
MEN0048-P0	CPNE4	snp	3	131261507	131261507	C	T
MEN0048-P0	ZNF708	snp	19	21476249	21476249	T	C
MEN0048-P0	ATP9A	snp	20	50286571	50286571	C	T
MEN0048-P0	HAO2	snp	1	119936413	119936413	C	T
MEN0048-P0	DNAJC13	snp	3	132244556	132244556	A	G
MEN0048-P0	ZNF431	snp	19	21366366	21366366	G	A
MEN0048-P0	DSP	snp	6	7585620	7585620	G	A
MEN0048-P0	THOC2	snp	X	122774430	122774430	C	A
MEN0048-P0	ZNF681	snp	19	23926839	23926839	A	G

MEN0048-P0	ZNF91	snp	19	23543800	23543800	T	C
MEN0048-P0	POLR2A	snp	17	7404938	7404938	G	A
MEN0048-P0	ZNF138	snp	7	64292444	64292444	A	G
MEN0048-P0	ZNF808	snp	19	53058687	53058687	A	C
MEN0048-P0	ZNF91	snp	19	23543296	23543296	T	C
MEN0048-P0	ACTG1	snp	17	79477737	79477737	G	C
MEN0048-P0	ZNF616	snp	19	52618845	52618845	T	A
MEN0048-P0	TUBB6	snp	18	12325395	12325395	G	A
MEN0048-P0	KRT37	snp	17	39580519	39580519	G	A
MEN0048-P0	NLRP3	snp	1	247597517	247597517	G	A
MEN0048-P0	NBPF10	snp	1	145299906	145299906	T	G
MEN0048-P0	MUC17	snp	7	100682680	100682680	G	C
MEN0048-P0	F5	snp	1	169510354	169510354	A	G
MEN0048-P0	KMT2C	snp	7	151874763	151874763	T	C
MEN0048-P0	ZNF117	snp	7	64439050	64439050	C	G
MEN0048-P0	KLC2	snp	11	66034372	66034372	G	A
MEN0048-P0	DAXX	snp	6	33289272	33289272	G	A
MEN0048-P0	ZFHX3	snp	16	72845884	72845884	G	A
MEN0048-P0	NRSN1	snp	6	24146146	24146146	A	G
MEN0048-P0	DNAH1	snp	3	52383403	52383403	C	G
MEN0048-P0	FAM47A	snp	X	34149266	34149266	G	A
MEN0048-P0	DHX9	snp	1	182845645	182845645	T	A
MEN0048-P0	ZNF585B	snp	19	37676173	37676173	C	T
MEN0048-P0	ZNF85	snp	19	21132615	21132615	A	G
MEN0048-P0	MDGA2	snp	14	47342740	47342740	C	T
MEN0048-P0	ESX1	snp	X	103499160	103499160	C	T
MEN0048-P0	ZNF678	snp	1	227843257	227843257	A	G
MEN0048-P0	NBPF9	snp	1	144828683	144828683	C	G
MEN0048-P0	SHROOM4	snp	X	50377185	50377185	G	A
MEN0048-P0	ZNF724P	snp	19	23405462	23405462	C	T
MEN0048-P0	ATP6V1H	snp	8	54708292	54708292	C	T
MEN0048-P0	PPP1R32	snp	11	61254481	61254481	G	A
MEN0048-P0	ZBTB20	snp	3	114069919	114069919	C	T
MEN0048-P0	ANKRD36C	snp	2	96593025	96593025	C	T
MEN0048-P0	TAP1	snp	6	32816469	32816469	G	A
MEN0048-P0	MAGEA4	snp	X	151092594	151092594	G	A
MEN0048-P0	PRDM15	snp	21	43222949	43222949	C	T
MEN0048-P0	KLHL6	snp	3	183226244	183226244	G	T
MEN0048-P0	ZNF708	snp	19	21476519	21476519	T	C
MEN0048-P0	C11orf49	snp	11	47176747	47176747	C	T
MEN0048-P0	ZNF776	snp	19	58265928	58265928	G	A
MEN0048-P0	LTBP1	snp	2	33500964	33500964	G	A
MEN0048-P0	GLP2R	snp	17	9764491	9764491	G	A
MEN0048-P0	ZNF85	snp	19	21132356	21132356	A	G
MEN0048-P0	ZNF780B	snp	19	40541934	40541934	G	C
MEN0048-P0	ZNF100	snp	19	21909734	21909734	G	C
MEN0048-P0	ZNF721	snp	4	436421	436421	A	G

MEN0048-P0	ZNF208	snp	19	22154249	22154249	A	G
MEN0048-P0	ZNF676	snp	19	22363008	22363008	C	G
MEN0048-P0	ZNF619	snp	3	40529017	40529017	A	C
MEN0048-P0	ALB	snp	4	74270854	74270854	G	A
MEN0048-P0	FAM50A	snp	X	153678412	153678412	C	T
MEN0048-P0	LIG3	snp	17	33310377	33310377	G	A
MEN0048-P0	PAPPA2	snp	1	176681002	176681002	A	C
MEN0048-P0	AGRN	snp	1	981113	981113	C	A
MEN0048-P0	MUC17	snp	7	100682505	100682505	T	C
MEN0048-P0	ZNF548	snp	19	57911082	57911082	C	T
MEN0048-P0	KIAA0556	snp	16	27761387	27761387	G	A
MEN0048-P0	AHNAK	snp	11	62293095	62293095	C	T
MEN0048-P0	REL	snp	2	61149606	61149606	G	A
MEN0048-P0	PCSK5	snp	9	78790192	78790192	C	G
MEN0048-P0	TBC1D14	snp	4	6925486	6925486	G	A
MEN0048-P0	AHNAK	snp	11	62295870	62295870	T	C
MEN0048-P0	WDR86	snp	7	151093196	151093196	C	T
MEN0048-P0	TP53	snp	17	7578371	7578371	C	T
MEN0048-P0	STON2	snp	14	81744954	81744954	G	T
MEN0048-P0	NBPF10	snp	1	145296448	145296448	T	A
MEN0048-P0	MPP1	snp	X	154020552	154020552	C	A
MEN0048-P0	RPTN	snp	1	152127897	152127897	C	T
MEN0048-P0	KIAA1009	snp	6	84896154	84896154	C	T
MEN0048-P0	ZNF714	snp	19	21300738	21300738	T	C
MEN0048-P0	6-Sep	snp	X	118767441	118767441	T	C
MEN0048-P0	KRTAP4-11	snp	17	39274150	39274150	T	A
MEN0048-P0	UNC79	snp	14	94109916	94109916	G	T
MEN0048-P0	ENAM	snp	4	71509091	71509091	C	T
MEN0048-P0	ZAN	snp	7	100350052	100350052	T	C
MEN0048-P0	KRTAP4-11	snp	17	39274206	39274206	C	T
MEN0048-P0	ZAN	snp	7	100350361	100350361	T	C
MEN0048-P0	ANKLE1	snp	19	17397493	17397493	G	T
MEN0048-P2	MAL	snp	2	95719159	95719159	G	T
MEN0048-P2	NBPF9	snp	1	144828624	144828624	A	T
MEN0048-P2	NBPF9	snp	1	144828707	144828707	G	T
MEN0048-P2	NBPF10	snp	1	145296448	145296448	T	A
MEN0048-P2	RUNX2	snp	6	45514792	45514792	C	G
MEN0048-P2	WNK2	snp	9	96062396	96062396	C	G
MEN0048-P2	CENPE	snp	4	104029980	104029980	G	A
MEN0048-P2	BTK	snp	X	100609669	100609669	C	G
MEN0048-P2	YIPF7	snp	4	44631547	44631547	T	C
MEN0048-P2	STON2	snp	14	81744954	81744954	G	T
MEN0048-P2	MPP1	snp	X	154020552	154020552	C	A
MEN0048-P2	OR8I2	snp	11	55861616	55861616	C	T
MEN0048-P2	ELOVL5	snp	6	53156637	53156637	G	C
MEN0048-P2	CRAMP1L	snp	16	1705221	1705221	A	G
MEN0048-P2	FZD8	snp	10	35930289	35930289	G	C

MEN0048-P2	AGL	snp	1	100356880	100356880	C	T
MEN0048-P2	IRS4	snp	X	107976735	107976735	A	C
MEN0048-P2	ZNF7	snp	8	146067855	146067855	C	T
MEN0048-P2	PHYKPL	snp	5	177651695	177651695	A	C
MEN0048-P2	COL6A6	snp	3	130285695	130285695	G	A
MEN0048-P2	PAPPA2	snp	1	176681002	176681002	A	C
MEN0048-P2	TP53	snp	17	7578371	7578371	C	T
MEN0048-P2	NBPF9	snp	1	144828683	144828683	C	G
MEN0048-P2	SLC2A9	snp	4	9998448	9998448	C	T
MEN0048-P2	SLC17A2	snp	6	25924070	25924070	C	T
MEN0048-P2	ZNF254	snp	19	24310294	24310294	T	G
MEN0048-P2	KMT2C	snp	7	151874763	151874763	T	C
MEN0048-P2	SHROOM4	snp	X	50377185	50377185	G	A
MEN0048-P2	HAO2	snp	1	119936413	119936413	C	T
MEN0048-P2	BRWD1	snp	21	40559073	40559073	A	C
MEN0048-P2	TACR3	snp	4	104579451	104579451	G	A
MEN0048-P2	BCAS3	snp	17	59093261	59093261	G	A
MEN0048-P2	SETD2	snp	3	47088054	47088054	G	T
MEN0048-P2	STAB1	snp	3	52556589	52556589	T	C
MEN0048-P2	PXDNL	snp	8	52321350	52321350	G	A
MEN0048-P2	CHRNA3	snp	15	78894452	78894452	C	T
MEN0048-P2	UBR5	snp	8	103372850	103372850	C	A
MEN0048-P2	DLG1	snp	3	196803489	196803489	T	G
MEN0048-P2	ZNF548	snp	19	57911082	57911082	C	T
MEN0048-P2	CMTM1	snp	16	66600543	66600543	A	C
MEN0048-P2	GLP2R	snp	17	9764491	9764491	G	A
MEN0048-P2	BRINP3	snp	1	190067216	190067216	C	A
MEN0048-P2	ZNF808	snp	19	53056864	53056864	C	A
MEN0048-P2	CCR7	snp	17	38711074	38711074	G	A
MEN0048-P2	ACSL6	snp	5	131308476	131308476	G	A
MEN0048-P2	CREBZF	snp	11	85375769	85375769	G	A
MEN0048-P2	THSD4	snp	15	72037537	72037537	G	A
MEN0048-P2	ABCA10	snp	17	67149465	67149465	C	T
MEN0048-P2	WDR90	snp	16	708627	708627	G	A
MEN0048-P2	LOXL2	snp	8	23225710	23225710	T	C
MEN0048-P2	THOC2	snp	X	122774430	122774430	C	A
MEN0048-P2	RGPD3	snp	2	107049681	107049681	T	C
MEN0048-P2	ZNF431	snp	19	21366366	21366366	G	A
MEN0048-P2	ZNF91	snp	19	23544724	23544724	T	C
MEN0048-P2	UTP15	snp	5	72875765	72875765	G	A
MEN0048-P2	SIGLEC12	snp	19	52000192	52000192	A	G
MEN0048-P2	KRTAP4-11	snp	17	39274161	39274161	T	C
MEN0048-P2	NBPF20	snp	1	148252752	148252752	C	T
MEN0048-P2	MBD3	snp	19	1584563	1584563	C	A
MEN0048-P2	FAM86C1	snp	11	71507261	71507261	A	G
MEN0048-P2	TBK1	snp	12	64889350	64889350	G	A
MEN0048-P2	ANKRD36C	snp	2	96521815	96521815	C	A

MEN0048-P2	PLD5	snp	1	242253187	242253187	T	A
MEN0048-P2	MYH13	snp	17	10214514	10214514	G	C
MEN0048-P2	KNG1	snp	3	186459531	186459531	G	A
MEN0048-P2	FZD8	snp	10	35930289	35930289	G	C
MEN0048-P2	SETD2	snp	3	47088054	47088054	G	T
MEN0048-P2	MAL	snp	2	95719159	95719159	G	T
MEN0048-P2	WNK2	snp	9	96062396	96062396	C	G
MEN0048-P2	CRAMP1L	snp	16	1705221	1705221	A	G
MEN0048-P2	BTK	snp	X	100609669	100609669	C	G
MEN0048-P2	SLC2A9	snp	4	9998448	9998448	C	T
MEN0048-P2	SLC17A2	snp	6	25924070	25924070	C	T
MEN0048-P2	SLC17A2	snp	6	25924070	25924070	C	T
MEN0048-P2	CREBZF	snp	11	85375769	85375769	G	A
MEN0048-P2	LOXL2	snp	8	23225710	23225710	T	C
MEN0048-P2	OR8I2	snp	11	55861616	55861616	C	T
MEN0048-P2	FAM86C1	snp	11	71507261	71507261	A	G
MEN0048-P2	BRWD1	snp	21	40559073	40559073	A	C
MEN0048-P2	AGL	snp	1	100356880	100356880	C	T
MEN0048-P2	NBPF9	snp	1	144828707	144828707	G	T
MEN0048-P2	CCR7	snp	17	38711074	38711074	G	A
MEN0048-P2	SIGLEC12	snp	19	52000192	52000192	A	G
MEN0048-P2	CMTM1	snp	16	66600543	66600543	A	C
MEN0048-P2	COL6A6	snp	3	130285695	130285695	G	A
MEN0048-P2	RUNX2	snp	6	45514792	45514792	C	G
MEN0048-P2	TACR3	snp	4	104579451	104579451	G	A
MEN0048-P2	TBK1	snp	12	64889350	64889350	G	A
MEN0048-P2	ZNF254	snp	19	24310294	24310294	T	G
MEN0048-P2	ANKRD36C	snp	2	96521815	96521815	C	A
MEN0048-P2	ACSL6	snp	5	131308476	131308476	G	A
MEN0048-P2	BCAS3	snp	17	59093261	59093261	G	A
MEN0048-P2	DLG1	snp	3	196803489	196803489	T	G
MEN0048-P2	BRINP3	snp	1	190067216	190067216	C	A
MEN0048-P2	PHYKPL	snp	5	177651695	177651695	A	C
MEN0048-P2	PLD5	snp	1	242253187	242253187	T	A
MEN0048-P2	ZNF7	snp	8	146067855	146067855	C	T
MEN0048-P2	WDR90	snp	16	708627	708627	G	A
MEN0048-P2	STAB1	snp	3	52556589	52556589	T	C
MEN0048-P2	YIPF7	snp	4	44631547	44631547	T	C
MEN0048-P2	HAO2	snp	1	119936413	119936413	C	T
MEN0048-P2	ZNF431	snp	19	21366366	21366366	G	A
MEN0048-P2	THOC2	snp	X	122774430	122774430	C	A
MEN0048-P2	ZNF91	snp	19	23544724	23544724	T	C
MEN0048-P2	ELOVL5	snp	6	53156637	53156637	G	C
MEN0048-P2	KMT2C	snp	7	151874763	151874763	T	C
MEN0048-P2	NBPF9	snp	1	144828683	144828683	C	G
MEN0048-P2	SHROOM4	snp	X	50377185	50377185	G	A
MEN0048-P2	IRS4	snp	X	107976735	107976735	A	C

MEN0048-P2	PXDNL	snp	8	52321350	52321350	G	A
MEN0048-P2	CENPE	snp	4	104029980	104029980	G	A
MEN0048-P2	GLP2R	snp	17	9764491	9764491	G	A
MEN0048-P2	ABCA10	snp	17	67149465	67149465	C	T
MEN0048-P2	NBPF9	snp	1	144828624	144828624	A	T
MEN0048-P2	RGPD3	snp	2	107049681	107049681	T	C
MEN0048-P2	ZNF808	snp	19	53056864	53056864	C	A
MEN0048-P2	UTP15	snp	5	72875765	72875765	G	A
MEN0048-P2	PAPPA2	snp	1	176681002	176681002	A	C
MEN0048-P2	UBR5	snp	8	103372850	103372850	C	A
MEN0048-P2	ZNF548	snp	19	57911082	57911082	C	T
MEN0048-P2	CHRNA3	snp	15	78894452	78894452	C	T
MEN0048-P2	TP53	snp	17	7578371	7578371	C	T
MEN0048-P2	STON2	snp	14	81744954	81744954	G	T
MEN0048-P2	NBPF10	snp	1	145296448	145296448	T	A
MEN0048-P2	MPP1	snp	X	154020552	154020552	C	A
MEN0048-P2	KRTAP4-11	snp	17	39274161	39274161	T	C
MEN0048-P2	MBD3	snp	19	1584563	1584563	C	A
MEN0048-P2	MYH13	snp	17	10214514	10214514	G	C
MEN0048-P2	THSD4	snp	15	72037537	72037537	G	A
MEN0048-P2	KNG1	snp	3	186459531	186459531	G	A
MEN0048-P2	NBPF20	snp	1	148252752	148252752	C	T
MEN0048-P3	MAL	snp	2	95719159	95719159	G	T
MEN0048-P3	NBPF9	snp	1	144828624	144828624	A	T
MEN0048-P3	NBPF9	snp	1	144828707	144828707	G	T
MEN0048-P3	CHP2	snp	16	23767187	23767187	A	T
MEN0048-P3	NBPF10	snp	1	145296448	145296448	T	A
MEN0048-P3	RUNX2	snp	6	45514792	45514792	C	G
MEN0048-P3	WNK2	snp	9	96062396	96062396	C	G
MEN0048-P3	CENPE	snp	4	104029980	104029980	G	A
MEN0048-P3	BTK	snp	X	100609669	100609669	C	G
MEN0048-P3	STON2	snp	14	81744954	81744954	G	T
MEN0048-P3	MPP1	snp	X	154020552	154020552	C	A
MEN0048-P3	SOWAHD	snp	X	118893556	118893556	T	G
MEN0048-P3	ELOVL5	snp	6	53156637	53156637	G	C
MEN0048-P3	CRAMP1L	snp	16	1705221	1705221	A	G
MEN0048-P3	FZD8	snp	10	35930289	35930289	G	C
MEN0048-P3	AGL	snp	1	100356880	100356880	C	T
MEN0048-P3	IRS4	snp	X	107976735	107976735	A	C
MEN0048-P3	PHYKPL	snp	5	177651695	177651695	A	C
MEN0048-P3	PAPPA2	snp	1	176681002	176681002	A	C
MEN0048-P3	TP53	snp	17	7578371	7578371	C	T
MEN0048-P3	NBPF9	snp	1	144828683	144828683	C	G
MEN0048-P3	AQP7	snp	9	33385614	33385614	A	T
MEN0048-P3	SLC17A2	snp	6	25924070	25924070	C	T
MEN0048-P3	KMT2C	snp	7	151874763	151874763	T	C
MEN0048-P3	SHROOM4	snp	X	50377185	50377185	G	A

MEN0048-P3	HAO2	snp	1	119936413	119936413	C	T
MEN0048-P3	BRWD1	snp	21	40559073	40559073	A	C
MEN0048-P3	TACR3	snp	4	104579451	104579451	G	A
MEN0048-P3	FAM86C1	snp	11	71507232	71507232	C	G
MEN0048-P3	SETD2	snp	3	47088054	47088054	G	T
MEN0048-P3	NBPF9	snp	1	144828704	144828704	T	G
MEN0048-P3	STAB1	snp	3	52556589	52556589	T	C
MEN0048-P3	PXDNL	snp	8	52321350	52321350	G	A
MEN0048-P3	CHRNA3	snp	15	78894452	78894452	C	T
MEN0048-P3	DLG1	snp	3	196803489	196803489	T	G
MEN0048-P3	ZNF548	snp	19	57911082	57911082	C	T
MEN0048-P3	CMTM1	snp	16	66600543	66600543	A	C
MEN0048-P3	GLP2R	snp	17	9764491	9764491	G	A
MEN0048-P3	BRINP3	snp	1	190067216	190067216	C	A
MEN0048-P3	CCR7	snp	17	38711074	38711074	G	A
MEN0048-P3	ACSL6	snp	5	131308476	131308476	G	A
MEN0048-P3	FAM133A	snp	X	92964673	92964673	G	T
MEN0048-P3	CREBZF	snp	11	85375769	85375769	G	A
MEN0048-P3	WDR90	snp	16	708627	708627	G	A
MEN0048-P3	LOXL2	snp	8	23225710	23225710	T	C
MEN0048-P3	THOC2	snp	X	122774430	122774430	C	A
MEN0048-P3	RGPD3	snp	2	107049681	107049681	T	C
MEN0048-P3	UTP15	snp	5	72875765	72875765	G	A
MEN0048-P3	SIGLEC12	snp	19	52000192	52000192	A	G
MEN0048-P3	PCSK5	snp	9	78790192	78790192	C	G
MEN0048-P3	DNAAF2	snp	14	50100271	50100271	T	C
MEN0048-P3	MCC	snp	5	112824054	112824054	C	T
MEN0048-P3	PRG4	snp	1	186276794	186276794	T	C
MEN0048-P3	FAM86C1	snp	11	71507261	71507261	A	G
MEN0048-P3	PLD5	snp	1	242253187	242253187	T	A
MEN0048-P3	MYH13	snp	17	10214514	10214514	G	C
MEN0048-P3	TCEAL3	snp	X	102864204	102864204	G	A
MEN0048-P3	MDK	snp	11	46404331	46404331	C	G
MEN0048-P3	FZD8	snp	10	35930289	35930289	G	C
MEN0048-P3	MDK	snp	11	46404331	46404331	C	G
MEN0048-P3	SETD2	snp	3	47088054	47088054	G	T
MEN0048-P3	MCC	snp	5	112824054	112824054	C	T
MEN0048-P3	MAL	snp	2	95719159	95719159	G	T
MEN0048-P3	WNK2	snp	9	96062396	96062396	C	G
MEN0048-P3	CRAMP1L	snp	16	1705221	1705221	A	G
MEN0048-P3	BTK	snp	X	100609669	100609669	C	G
MEN0048-P3	SLC17A2	snp	6	25924070	25924070	C	T
MEN0048-P3	SLC17A2	snp	6	25924070	25924070	C	T
MEN0048-P3	CREBZF	snp	11	85375769	85375769	G	A
MEN0048-P3	LOXL2	snp	8	23225710	23225710	T	C
MEN0048-P3	FAM86C1	snp	11	71507232	71507232	C	G
MEN0048-P3	BRWD1	snp	21	40559073	40559073	A	C

MEN0048-P3	AGL	snp	1	100356880	100356880	C	T
MEN0048-P3	NBPF9	snp	1	144828707	144828707	G	T
MEN0048-P3	CCR7	snp	17	38711074	38711074	G	A
MEN0048-P3	CHP2	snp	16	23767187	23767187	A	T
MEN0048-P3	SOWAHD	snp	X	118893556	118893556	T	G
MEN0048-P3	SIGLEC12	snp	19	52000192	52000192	A	G
MEN0048-P3	CMTM1	snp	16	66600543	66600543	A	C
MEN0048-P3	RUNX2	snp	6	45514792	45514792	C	G
MEN0048-P3	TACR3	snp	4	104579451	104579451	G	A
MEN0048-P3	ACSL6	snp	5	131308476	131308476	G	A
MEN0048-P3	DLG1	snp	3	196803489	196803489	T	G
MEN0048-P3	BRINP3	snp	1	190067216	190067216	C	A
MEN0048-P3	PHYKPL	snp	5	177651695	177651695	A	C
MEN0048-P3	PLD5	snp	1	242253187	242253187	T	A
MEN0048-P3	WDR90	snp	16	708627	708627	G	A
MEN0048-P3	STAB1	snp	3	52556589	52556589	T	C
MEN0048-P3	HAO2	snp	1	119936413	119936413	C	T
MEN0048-P3	THOC2	snp	X	122774430	122774430	C	A
MEN0048-P3	ELOVL5	snp	6	53156637	53156637	G	C
MEN0048-P3	DNAAF2	snp	14	50100271	50100271	T	C
MEN0048-P3	KMT2C	snp	7	151874763	151874763	T	C
MEN0048-P3	AQP7	snp	9	33385614	33385614	A	T
MEN0048-P3	NBPF9	snp	1	144828683	144828683	C	G
MEN0048-P3	SHROOM4	snp	X	50377185	50377185	G	A
MEN0048-P3	IRS4	snp	X	107976735	107976735	A	C
MEN0048-P3	PXDNL	snp	8	52321350	52321350	G	A
MEN0048-P3	CENPE	snp	4	104029980	104029980	G	A
MEN0048-P3	GLP2R	snp	17	9764491	9764491	G	A
MEN0048-P3	NBPF9	snp	1	144828624	144828624	A	T
MEN0048-P3	RGPD3	snp	2	107049681	107049681	T	C
MEN0048-P3	FAM133A	snp	X	92964673	92964673	G	T
MEN0048-P3	UTP15	snp	5	72875765	72875765	G	A
MEN0048-P3	PAPPA2	snp	1	176681002	176681002	A	C
MEN0048-P3	ZNF548	snp	19	57911082	57911082	C	T
MEN0048-P3	CHRNA3	snp	15	78894452	78894452	C	T
MEN0048-P3	PCSK5	snp	9	78790192	78790192	C	G
MEN0048-P3	TP53	snp	17	7578371	7578371	C	T
MEN0048-P3	STON2	snp	14	81744954	81744954	G	T
MEN0048-P3	NBPF10	snp	1	145296448	145296448	T	A
MEN0048-P3	MPP1	snp	X	154020552	154020552	C	A
MEN0048-P3	MYH13	snp	17	10214514	10214514	G	C
MEN0048-P3	PRG4	snp	1	186276794	186276794	T	C
MEN0048-P3	TCEAL3	snp	X	102864204	102864204	G	A
MEN0048G-P1	MAL	snp	2	95719159	95719159	G	T
MEN0048G-P1	UTS2R	snp	17	80332919	80332919	G	A
MEN0048G-P1	CHP2	snp	16	23767187	23767187	A	T
MEN0048G-P1	RUNX2	snp	6	45514792	45514792	C	G

MEN0048G-P1	BTK	snp	X	100609669	100609669	C	G
MEN0048G-P1	YIPF7	snp	4	44631547	44631547	T	C
MEN0048G-P1	BEST3	snp	12	70049318	70049318	C	A
MEN0048G-P1	STON2	snp	14	81744954	81744954	G	T
MEN0048G-P1	EYS	snp	6	66005780	66005780	G	A
MEN0048G-P1	MPP1	snp	X	154020552	154020552	C	A
MEN0048G-P1	SOWAHD	snp	X	118893556	118893556	T	G
MEN0048G-P1	ELOVL5	snp	6	53156637	53156637	G	C
MEN0048G-P1	CRAMP1L	snp	16	1705221	1705221	A	G
MEN0048G-P1	RAB6C	snp	2	130738031	130738031	A	G
MEN0048G-P1	KDM5C	snp	X	53228235	53228235	C	A
MEN0048G-P1	CAMSAP3	snp	19	7671226	7671226	G	A
MEN0048G-P1	FZD8	snp	10	35930289	35930289	G	C
MEN0048G-P1	AGL	snp	1	100356880	100356880	C	T
MEN0048G-P1	CACNA1B	snp	9	140919411	140919411	C	A
MEN0048G-P1	CCDC132	snp	7	92985368	92985368	G	A
MEN0048G-P1	PHYKPL	snp	5	177651695	177651695	A	C
MEN0048G-P1	PAPPA2	snp	1	176681002	176681002	A	C
MEN0048G-P1	TP53	snp	17	7578371	7578371	C	T
MEN0048G-P1	ARHGEF5	snp	7	144061047	144061047	A	G
MEN0048G-P1	LRTM2	snp	12	1940319	1940319	C	T
MEN0048G-P1	KMT2C	snp	7	151874763	151874763	T	C
MEN0048G-P1	SHROOM4	snp	X	50377185	50377185	G	A
MEN0048G-P1	HAO2	snp	1	119936413	119936413	C	T
MEN0048G-P1	BRWD1	snp	21	40559073	40559073	A	C
MEN0048G-P1	HELZ2	snp	20	62195972	62195972	C	A
MEN0048G-P1	FANCD2	snp	3	10114557	10114557	A	G
MEN0048G-P1	ELN	snp	7	73450900	73450900	C	A
MEN0048G-P1	NBAS	snp	2	15326889	15326889	T	C
MEN0048G-P1	PPIP5K1	snp	15	43873489	43873489	C	T
MEN0048G-P1	STAB1	snp	3	52556589	52556589	T	C
MEN0048G-P1	PXDNL	snp	8	52321350	52321350	G	A
MEN0048G-P1	TMCO4	snp	1	20066409	20066409	C	T
MEN0048G-P1	STARD10	snp	11	72466113	72466113	C	T
MEN0048G-P1	RP11-830F9.6	snp	16	89017349	89017349	G	C
MEN0048G-P1	DLG1	snp	3	196803489	196803489	T	G
MEN0048G-P1	ZNF548	snp	19	57911082	57911082	C	T
MEN0048G-P1	PLXNC1	snp	12	94543046	94543046	G	T
MEN0048G-P1	ALDOA	snp	16	30080970	30080970	C	T
MEN0048G-P1	DDX11	snp	12	31242911	31242911	C	T
MEN0048G-P1	GLP2R	snp	17	9764491	9764491	G	A
MEN0048G-P1	SLC25A6	snp	X	1506303	1506303	G	T
MEN0048G-P1	AL133481.1	snp	10	81266819	81266819	G	A
MEN0048G-P1	BRINP3	snp	1	190067216	190067216	C	A
MEN0048G-P1	SMARCA2	snp	9	2123887	2123887	C	T
MEN0048G-P1	SWT1	snp	1	185173912	185173912	G	C
MEN0048G-P1	ACSL6	snp	5	131308476	131308476	G	A

MEN0048G-P1	CREBZF	snp	11	85375769	85375769	G	A
MEN0048G-P1	S100P	snp	4	6695670	6695670	T	G
MEN0048G-P1	WDR90	snp	16	708627	708627	G	A
MEN0048G-P1	LOXL2	snp	8	23225710	23225710	T	C
MEN0048G-P1	COBL	snp	7	51150806	51150806	T	A
MEN0048G-P1	THOC2	snp	X	122774430	122774430	C	A
MEN0048G-P1	ANKRD30B	snp	18	14850328	14850328	A	G
MEN0048G-P1	NCKAP5	snp	2	133540236	133540236	G	C
MEN0048G-P1	CCDC150	snp	2	197586376	197586376	A	T
MEN0048G-P1	PLD5	snp	1	242253187	242253187	T	A
MEN0048G-P1	MYH13	snp	17	10214514	10214514	G	C
MEN0048G-P1	MYOF	snp	10	95169490	95169490	C	A
MEN0048G-P1	FZD8	snp	10	35930289	35930289	G	C
MEN0048G-P1	UTS2R	snp	17	80332919	80332919	G	A
MEN0048G-P1	MAL	snp	2	95719159	95719159	G	T
MEN0048G-P1	CRAMP1L	snp	16	1705221	1705221	A	G
MEN0048G-P1	BTK	snp	X	100609669	100609669	C	G
MEN0048G-P1	COBL	snp	7	51150806	51150806	T	A
MEN0048G-P1	CREBZF	snp	11	85375769	85375769	G	A
MEN0048G-P1	ELN	snp	7	73450900	73450900	C	A
MEN0048G-P1	LOXL2	snp	8	23225710	23225710	T	C
MEN0048G-P1	NBAS	snp	2	15326889	15326889	T	C
MEN0048G-P1	BRWD1	snp	21	40559073	40559073	A	C
MEN0048G-P1	AGL	snp	1	100356880	100356880	C	T
MEN0048G-P1	ALDOA	snp	16	30080970	30080970	C	T
MEN0048G-P1	CHP2	snp	16	23767187	23767187	A	T
MEN0048G-P1	SOWAHD	snp	X	118893556	118893556	T	G
MEN0048G-P1	TMCO4	snp	1	20066409	20066409	C	T
MEN0048G-P1	RUNX2	snp	6	45514792	45514792	C	G
MEN0048G-P1	HELZ2	snp	20	62195972	62195972	C	A
MEN0048G-P1	LRTM2	snp	12	1940319	1940319	C	T
MEN0048G-P1	ACSL6	snp	5	131308476	131308476	G	A
MEN0048G-P1	PLXNC1	snp	12	94543046	94543046	G	T
MEN0048G-P1	DLG1	snp	3	196803489	196803489	T	G
MEN0048G-P1	BRINP3	snp	1	190067216	190067216	C	A
MEN0048G-P1	PHYKPL	snp	5	177651695	177651695	A	C
MEN0048G-P1	STARD10	snp	11	72466113	72466113	C	T
MEN0048G-P1	PLD5	snp	1	242253187	242253187	T	A
MEN0048G-P1	SWT1	snp	1	185173912	185173912	G	C
MEN0048G-P1	CAMSAP3	snp	19	7671226	7671226	G	A
MEN0048G-P1	DDX11	snp	12	31242911	31242911	C	T
MEN0048G-P1	ARHGEF5	snp	7	144061047	144061047	A	G
MEN0048G-P1	RP11-830F9.6	snp	16	89017349	89017349	G	C
MEN0048G-P1	WDR90	snp	16	708627	708627	G	A
MEN0048G-P1	STAB1	snp	3	52556589	52556589	T	C
MEN0048G-P1	EYS	snp	6	66005780	66005780	G	A
MEN0048G-P1	AL133481.1	snp	10	81266819	81266819	G	A

MEN0048G-P1	YIPF7	snp	4	44631547	44631547	T	C
MEN0048G-P1	MYOF	snp	10	95169490	95169490	C	A
MEN0048G-P1	HAO2	snp	1	119936413	119936413	C	T
MEN0048G-P1	THOC2	snp	X	122774430	122774430	C	A
MEN0048G-P1	BEST3	snp	12	70049318	70049318	C	A
MEN0048G-P1	ELOVL5	snp	6	53156637	53156637	G	C
MEN0048G-P1	SLC25A6	snp	X	1506303	1506303	G	T
MEN0048G-P1	RAB6C	snp	2	130738031	130738031	A	G
MEN0048G-P1	KMT2C	snp	7	151874763	151874763	T	C
MEN0048G-P1	PPIP5K1	snp	15	43873489	43873489	C	T
MEN0048G-P1	FANCD2	snp	3	10114557	10114557	A	G
MEN0048G-P1	SHROOM4	snp	X	50377185	50377185	G	A
MEN0048G-P1	CCDC132	snp	7	92985368	92985368	G	A
MEN0048G-P1	PXDNL	snp	8	52321350	52321350	G	A
MEN0048G-P1	CACNA1B	snp	9	140919411	140919411	C	A
MEN0048G-P1	GLP2R	snp	17	9764491	9764491	G	A
MEN0048G-P1	KDM5C	snp	X	53228235	53228235	C	A
MEN0048G-P1	S100P	snp	4	6695670	6695670	T	G
MEN0048G-P1	PAPPA2	snp	1	176681002	176681002	A	C
MEN0048G-P1	ZNF548	snp	19	57911082	57911082	C	T
MEN0048G-P1	SMARCA2	snp	9	2123887	2123887	C	T
MEN0048G-P1	TP53	snp	17	7578371	7578371	C	T
MEN0048G-P1	STON2	snp	14	81744954	81744954	G	T
MEN0048G-P1	MPP1	snp	X	154020552	154020552	C	A
MEN0048G-P1	ANKRD30B	snp	18	14850328	14850328	A	G
MEN0048G-P1	NCKAP5	snp	2	133540236	133540236	G	C
MEN0048G-P1	MYH13	snp	17	10214514	10214514	G	C
MEN0048G-P1	CCDC150	snp	2	197586376	197586376	A	T
MEN0088-P	RGCC	snp	13	42032497	42032497	C	A
MEN0088-P	FAT1	snp	4	187628667	187628667	C	T
MEN0088-P	TSPAN8	snp	12	71531816	71531816	G	C
MEN0088-P	KIAA0586	snp	14	58896146	58896146	G	A
MEN0088-P	CNOT1	snp	16	58615279	58615279	T	C
MEN0088-P	BRMS1L	snp	14	36333076	36333076	A	T
MEN0088-P	PAPOLA	snp	14	97022210	97022210	C	G
MEN0088-P	ZFPM2	snp	8	106813476	106813476	G	A
MEN0088-P	PIK3C2G	snp	12	18719987	18719987	C	A
MEN0088-P	LRP5	snp	11	68133075	68133075	C	A
MEN0088-P	ITM2B	snp	13	48828029	48828029	T	C
MEN0088-P	ZMYM2	snp	13	20580650	20580650	C	T
MEN0088-P	KIF13B	snp	8	28956724	28956724	G	T
MEN0088-P	SCARB1	snp	12	125284768	125284768	G	T
MEN0088-P	TSPAN8	snp	12	71531804	71531804	T	C
MEN0088-P	DNAH8	snp	6	38866058	38866058	G	C
MEN0088-P	MYH3	snp	17	10542890	10542890	G	A
MEN0088-P	SDK2	snp	17	71334898	71334898	G	T
MEN0088-P	NCOA2	snp	8	71057051	71057051	T	A

MEN0088-P	SPINK14	snp	5	147553857	147553857	C	T
MEN0088-P	FAM126A	snp	7	23017031	23017031	C	T
MEN0088-P	KIF13B	snp	8	28956724	28956724	G	T
MEN0088-P	ITM2B	snp	13	48828029	48828029	T	C
MEN0088-P	SCARB1	snp	12	125284768	125284768	G	T
MEN0088-P	PAPOLA	snp	14	97022210	97022210	C	G
MEN0088-P	FAT1	snp	4	187628667	187628667	C	T
MEN0088-P	CNOT1	snp	16	58615279	58615279	T	C
MEN0088-P	DNAH8	snp	6	38866058	38866058	G	C
MEN0088-P	FAM126A	snp	7	23017031	23017031	C	T
MEN0088-P	KIAA0586	snp	14	58896146	58896146	G	A
MEN0088-P	RGCC	snp	13	42032497	42032497	C	A
MEN0088-P	PIK3C2G	snp	12	18719987	18719987	C	A
MEN0088-P	LRP5	snp	11	68133075	68133075	C	A
MEN0088-P	TSPAN8	snp	12	71531816	71531816	G	C
MEN0088-P	NCOA2	snp	8	71057051	71057051	T	A
MEN0088-P	SPINK14	snp	5	147553857	147553857	C	T
MEN0088-P	MYH3	snp	17	10542890	10542890	G	A
MEN0088-P	ZMYM2	snp	13	20580650	20580650	C	T
MEN0088-P	BRMS1L	snp	14	36333076	36333076	A	T
MEN0088-P	ZFPM2	snp	8	106813476	106813476	G	A
MEN0088-P	SDK2	snp	17	71334898	71334898	G	T
MEN0089-P	GABRR1	snp	6	89891668	89891668	G	T
MEN0089-P	CRLF2	snp	X	1331472	1331472	A	T
MEN0089-P	NDST3	snp	4	119064828	119064828	G	A
MEN0089-P	TOP1MT	snp	8	144406252	144406252	T	C
MEN0089-P	MOGAT3	snp	7	100839217	100839217	G	A
MEN0089-P	TBC1D7	snp	6	13306650	13306650	T	C
MEN0089-P	MAP3K4	snp	6	161519378	161519378	C	T
MEN0089-P	MCRS1	snp	12	49953267	49953267	C	T
MEN0089-P	MRM1	snp	17	34958469	34958469	G	A
MEN0089-P	ANKRD36C	snp	2	96593016	96593016	C	A
MEN0089-P	ZBTB8A	snp	1	33058651	33058651	A	T
MEN0089-P	ANKRD36C	snp	2	96593025	96593025	C	T
MEN0089-P	CACNA1E	snp	1	181702087	181702087	A	T
MEN0089-P	AASDH	snp	4	57209798	57209798	G	A
MEN0089-P	PLEKHH1	snp	14	68029583	68029583	C	T
MEN0089-P	AKIRIN1	snp	1	39469053	39469053	T	G
MEN0089-P	CNTN6	snp	3	1415294	1415294	C	A
MEN0089-P	ANKRD36C	snp	2	96593000	96593000	A	G
MEN0089-P	TP53BP2	snp	1	223992037	223992037	T	G
MEN0089-P	PRB1	snp	12	11506852	11506852	G	T
MEN0089-P	ZNF737	snp	19	20728095	20728095	A	C
MEN0089-P	PCDHGA11	snp	5	140802401	140802401	G	A
MEN0089-P	MCRS1	snp	12	49953267	49953267	C	T
MEN0089-P	MAP3K4	snp	6	161519378	161519378	C	T
MEN0089-P	ZBTB8A	snp	1	33058651	33058651	A	T

MEN0089-P	GABRR1	snp	6	89891668	89891668	G	T
MEN0089-P	AASDH	snp	4	57209798	57209798	G	A
MEN0089-P	MRM1	snp	17	34958469	34958469	G	A
MEN0089-P	TBC1D7	snp	6	13306650	13306650	T	C
MEN0089-P	NDST3	snp	4	119064828	119064828	G	A
MEN0089-P	ANKRD36C	snp	2	96593025	96593025	C	T
MEN0089-P	PLEKHH1	snp	14	68029583	68029583	C	T
MEN0089-P	MOGAT3	snp	7	100839217	100839217	G	A
MEN0089-P	CRLF2	snp	X	1331472	1331472	A	T
MEN0089-P	AKIRIN1	snp	1	39469053	39469053	T	G
MEN0089-P	ANKRD36C	snp	2	96593016	96593016	C	A
MEN0089-P	ZNF737	snp	19	20728095	20728095	A	C
MEN0089-P	CNTN6	snp	3	1415294	1415294	C	A
MEN0089-P	TOP1MT	snp	8	144406252	144406252	T	C
MEN0089-P	PCDHGA11	snp	5	140802401	140802401	G	A
MEN0089-P	TP53BP2	snp	1	223992037	223992037	T	G
MEN0089-P	CACNA1E	snp	1	181702087	181702087	A	T
MEN0089-P	ANKRD36C	snp	2	96593000	96593000	A	G
MEN0089-P	PRB1	snp	12	11506852	11506852	G	T
MEN0092-P	TTLL9	snp	20	30510805	30510805	G	A
MEN0092-P	PTPRF	snp	1	44069494	44069494	C	T
MEN0092-P	PKMYT1	snp	16	3029649	3029649	G	A
MEN0092-P	ERVFRD-1	snp	6	11104921	11104921	C	T
MEN0092-P	SPEG	snp	2	220353850	220353850	G	A
MEN0092-P	HERC4	snp	10	69684875	69684875	C	T
MEN0092-P	SLC35G2	snp	3	136573405	136573405	G	A
MEN0092-P	TMEM108	snp	3	133098626	133098626	C	T
MEN0092-P	PSMB11	snp	14	23512003	23512003	G	A
MEN0092-P	TMOD1	snp	9	100328233	100328233	C	T
MEN0092-P	SCMH1	snp	1	41617308	41617308	G	A
MEN0092-P	MAST1	snp	19	12981886	12981886	G	A
MEN0092-P	USP35	snp	11	77907340	77907340	G	A
MEN0092-P	EPPK1	snp	8	144946755	144946755	C	T
MEN0092-P	PLXNA1	snp	3	126708188	126708188	G	A
MEN0092-P	KAT2A	snp	17	40265767	40265767	C	T
MEN0092-P	TRAP1	snp	16	3727532	3727532	G	A
MEN0092-P	PTPN14	snp	1	214549666	214549666	C	T
MEN0092-P	AOC2	snp	17	41001304	41001304	G	A
MEN0092-P	NPBWR2	snp	20	62737901	62737901	G	A
MEN0092-P	PAXBP1	snp	21	34123491	34123491	G	A
MEN0092-P	TBC1D9B	snp	5	179305326	179305326	T	C
MEN0092-P	POM121	snp	7	72398987	72398987	G	A
MEN0092-P	SDPR	snp	2	192701100	192701100	G	A
MEN0092-P	LNPEP	snp	5	96315595	96315595	C	T
MEN0092-P	SEC16B	snp	1	177911150	177911150	C	T
MEN0092-P	C1orf222	snp	1	1854450	1854450	G	A
MEN0092-P	CENPI	snp	X	100403130	100403130	G	A

MEN0092-P	KCNJ11	snp	11	17408540	17408540	G	A
MEN0092-P	ZNFX1	snp	20	47879939	47879939	G	A
MEN0092-P	INTS3	snp	1	153742266	153742266	G	A
MEN0092-P	PPP2R2B	snp	5	145972609	145972609	C	T
MEN0092-P	GLYR1	snp	16	4861768	4861768	C	T
MEN0092-P	NOD2	snp	16	50746064	50746064	G	A
MEN0092-P	NOS1AP	snp	1	162336911	162336911	C	T
MEN0092-P	ERN1	snp	17	62121527	62121527	G	A
MEN0092-P	DYRK2	snp	12	68051937	68051937	C	T
MEN0092-P	NLRX1	snp	11	119045909	119045909	G	A
MEN0092-P	RAPGEF6	snp	5	130840366	130840366	G	A
MEN0092-P	MYH1	snp	17	10419567	10419567	C	A
MEN0092-P	UNC5B	snp	10	73050713	73050713	G	A
MEN0092-P	SMARCA4	snp	19	11132465	11132465	C	T
MEN0092-P	NFIC	snp	19	3382127	3382127	C	T
MEN0092-P	NEB	snp	2	152381087	152381087	G	C
MEN0092-P	TRIM46	snp	1	155152396	155152396	C	T
MEN0092-P	MARCO	snp	2	119750856	119750856	C	T
MEN0092-P	RAPGEF4	snp	2	173901401	173901401	T	C
MEN0092-P	SLIT2	snp	4	20533609	20533609	G	A
MEN0092-P	ARNTL	snp	11	13375965	13375965	G	A
MEN0092-P	TEP1	snp	14	20869673	20869673	A	G
MEN0092-P	ZNF77	snp	19	2933957	2933957	C	T
MEN0092-P	ZNF716	snp	7	57510026	57510026	G	A
MEN0092-P	TNXB	snp	6	32037369	32037369	C	T
MEN0092-P	LHFPL5	snp	6	35773661	35773661	G	A
MEN0092-P	FRK	snp	6	116264287	116264287	G	A
MEN0092-P	THOC6	snp	16	3077247	3077247	C	T
MEN0092-P	TCTN2	snp	12	124175110	124175110	G	A
MEN0092-P	RGAG1	snp	X	109697237	109697237	C	T
MEN0092-P	PSG4	snp	19	43708038	43708038	G	A
MEN0092-P	TBKBP1	snp	17	45785806	45785806	C	T
MEN0092-P	ZFHX4	snp	8	77616634	77616634	G	A
MEN0092-P	VPS13A	snp	9	79981670	79981670	G	A
MEN0092-P	RBBP6	snp	16	24578601	24578601	C	T
MEN0092-P	COPB2	snp	3	139078167	139078167	G	A
MEN0092-P	GRIA3	snp	X	122538739	122538739	G	A
MEN0092-P	FLG	snp	1	152277116	152277116	C	T
MEN0092-P	NID1	snp	1	236176855	236176855	C	T
MEN0092-P	HRH3	snp	20	60791370	60791370	A	C
MEN0092-P	WDR11	snp	10	122668104	122668104	G	A
MEN0092-P	POLQ	snp	3	121208882	121208882	T	C
MEN0092-P	LYRM5	snp	12	25357101	25357101	A	C
MEN0092-P	RYR3	snp	15	33926811	33926811	C	T
MEN0092-P	SAMD4A	snp	14	55243178	55243178	G	A
MEN0092-P	OR5B21	snp	11	58275329	58275329	G	A
MEN0092-P	SON	snp	21	34931986	34931986	G	A

MEN0092-P	PRC1	snp	15	91517428	91517428	C	T
MEN0092-P	DLGAP1	snp	18	3879539	3879539	C	T
MEN0092-P	EML1	snp	14	100377846	100377846	C	T
MEN0092-P	IQSEC3	snp	12	247481	247481	G	A
MEN0092-P	SLC32A1	snp	20	37356192	37356192	G	A
MEN0092-P	HERC1	snp	15	63933709	63933709	C	T
MEN0092-P	ESR2	snp	14	64701701	64701701	C	T
MEN0092-P	HTR2B	snp	2	231974117	231974117	G	T
MEN0092-P	LPAR6	snp	13	48986021	48986021	T	G
MEN0092-P	CYTH4	snp	22	37709494	37709494	G	A
MEN0092-P	TNXB	snp	6	32010287	32010287	C	T
MEN0092-P	KIAA1683	snp	19	18376965	18376965	G	T
MEN0092-P	RNF38	snp	9	36352824	36352824	G	A
MEN0092-P	KLC2	snp	11	66030380	66030380	G	A
MEN0092-P	ATP2A1	snp	16	28911947	28911947	C	T
MEN0092-P	CEP63	snp	3	134268018	134268018	G	T
MEN0092-P	NCAPH2	snp	22	50956007	50956007	C	T
MEN0092-P	POM121C	snp	7	75050828	75050828	C	T
MEN0092-P	RTN2	snp	19	45998201	45998201	C	T
MEN0092-P	MRPS10	snp	6	42185554	42185554	G	A
MEN0092-P	AIF1	snp	6	31583916	31583916	G	A
MEN0092-P	POLR3E	snp	16	22335966	22335966	C	T
MEN0092-P	RICTOR	snp	5	38950324	38950324	G	A
MEN0092-P	TTN	snp	2	179464170	179464170	G	A
MEN0092-P	HIVEP1	snp	6	12122908	12122908	G	C
MEN0092-P	MRGPRX1	snp	11	18955875	18955875	G	A
MEN0092-P	POLR2A	snp	17	7406782	7406782	G	A
MEN0092-P	TMC1	snp	9	75406943	75406943	G	A
MEN0092-P	GPR52	snp	1	174417941	174417941	G	A
MEN0092-P	ZNF830	snp	17	33289525	33289525	T	C
MEN0092-P	B9D1	snp	17	19263657	19263657	G	A
MEN0092-P	PARD3B	snp	2	206037007	206037007	G	A
MEN0092-P	FCGBP	snp	19	40364333	40364333	C	T
MEN0092-P	PALLD	snp	4	169433059	169433059	G	A
MEN0092-P	KLF7	snp	2	207988551	207988551	C	T
MEN0092-P	SLC47A1	snp	17	19480670	19480670	C	T
MEN0092-P	P2RX4	snp	12	121666668	121666668	A	C
MEN0092-P	SLC6A16	snp	19	49812145	49812145	C	T
MEN0092-P	KIAA1683	snp	19	18376959	18376959	G	C
MEN0092-P	POLE	snp	12	133225558	133225558	T	C
MEN0092-P	NOS1AP	snp	1	162313643	162313643	G	A
MEN0092-P	MSTO1	snp	1	155717640	155717640	C	T
MEN0092-P	GBAS	snp	7	56065092	56065092	G	A
MEN0092-P	LIMK1	snp	7	73523341	73523341	C	T
MEN0092-P	FBXL3	snp	13	77595741	77595741	C	G
MEN0092-P	HEBP1	snp	12	13128363	13128363	C	T
MEN0092-P	FGD2	snp	6	36982782	36982782	C	T

MEN0092-P	PAM	snp	5	102364747	102364747	C	T
MEN0092-P	NOS1AP	snp	1	162335254	162335254	C	T
MEN0092-P	CEACAM5	snp	19	42213763	42213763	C	T
MEN0092-P	MYOG	snp	1	203054890	203054890	G	A
MEN0092-P	KALRN	snp	3	124193577	124193577	T	C
MEN0092-P	PACS1	snp	11	66000525	66000525	G	A
MEN0092-P	TRIM48	snp	11	55035844	55035844	T	C
MEN0092-P	MRPL54	snp	19	3767338	3767338	C	T
MEN0092-P	ZNF213	snp	16	3189049	3189049	G	A
MEN0092-P	CA14	snp	1	150233929	150233929	A	G
MEN0092-P	FOPNL	snp	16	15967450	15967450	G	A
MEN0092-P	ME3	snp	11	86209022	86209022	C	A
MEN0092-P	TCHH	snp	1	152080468	152080468	C	T
MEN0092-P	MTSS1L	snp	16	70712220	70712220	G	A
MEN0092-P	EGR2	snp	10	64573434	64573434	T	C
MEN0092-P	IL2RG	snp	X	70330402	70330402	G	A
MEN0092-P	MAPK7	snp	17	19285573	19285573	G	A
MEN0092-P	ATP2B2	snp	3	10417322	10417322	A	G
MEN0092-P	AP2M1	snp	3	183899704	183899704	C	T
MEN0092-P	LHFPL5	snp	6	35773526	35773526	G	A
MEN0092-P	HS3ST4	snp	16	26147180	26147180	T	A
MEN0092-P	MACF1	snp	1	39900201	39900201	A	G
MEN0092-P	PCDHA8	snp	5	140222585	140222585	A	G
MEN0092-P	RNF213	snp	17	78350306	78350306	C	A
MEN0092-P	LRRC37B	snp	17	30349744	30349744	G	A
MEN0092-P	SLC16A7	snp	12	60168915	60168915	A	G
MEN0092-P	SIRPD	snp	20	1532445	1532445	G	A
MEN0092-P	SULT1C2	snp	2	108910769	108910769	G	A
MEN0092-P	CSNK1G2	snp	19	1979048	1979048	C	T
MEN0092-P	ARHGAP5	snp	14	32561286	32561286	A	G
MEN0092-P	DHRS4L2	snp	14	24464325	24464325	G	A
MEN0092-P	HPD	snp	12	122281735	122281735	G	A
MEN0092-P	PRADC1	snp	2	73456041	73456041	G	A
MEN0092-P	HS3ST2	snp	16	22926383	22926383	G	A
MEN0092-P	LRRIQ1	snp	12	85492270	85492270	G	A
MEN0092-P	OR4N5	snp	14	20612564	20612564	C	T
MEN0092-P	UHMK1	snp	1	162492234	162492234	C	T
MEN0092-P	ADAMTS6	snp	5	64587158	64587158	C	A
MEN0092-P	PHF20L1	snp	8	133790127	133790127	G	C
MEN0092-P	CTCFL	snp	20	56089711	56089711	C	T
MEN0092-P	RPUSD3	snp	3	9883915	9883915	G	A
MEN0092-P	VARS2	snp	6	30889969	30889969	G	A
MEN0092-P	BCL9	snp	1	147095868	147095868	T	C
MEN0092-P	ARHGEF28	snp	5	73069856	73069856	G	A
MEN0092-P	AGFG2	snp	7	100159983	100159983	G	A
MEN0092-P	DEAF1	snp	11	654033	654033	C	T
MEN0092-P	UHRF1BP1	snp	6	34826040	34826040	G	A

MEN0092-P	MELK	snp	9	36651813	36651813	G	A
MEN0092-P	EPHB3	snp	3	184294803	184294803	G	A
MEN0092-P	MTFR1L	snp	1	26158495	26158495	A	T
MEN0092-P	RMND5B	snp	5	177569902	177569902	C	T
MEN0092-P	ZNF124	snp	1	247320487	247320487	C	T
MEN0092-P	FBXW7	snp	4	153244137	153244137	G	A
MEN0092-P	FAM110A	snp	20	826309	826309	C	T
MEN0092-P	CYP4F22	snp	19	15659011	15659011	C	T
MEN0092-P	ANAPC2	snp	9	140082048	140082048	G	A
MEN0092-P	SMG8	snp	17	57290754	57290754	G	A
MEN0092-P	BMP8B	snp	1	40229458	40229458	C	T
MEN0092-P	TUBGCP5	snp	15	22855271	22855271	A	G
MEN0092-P	ZC3H12B	snp	X	64722658	64722658	C	T
MEN0092-P	ZNF814	snp	19	58370321	58370321	C	T
MEN0092-P	ZNF711	snp	X	84510549	84510549	G	A
MEN0092-P	SVEP1	snp	9	113173903	113173903	C	T
MEN0092-P	ANKRD11	snp	16	89349513	89349513	G	A
MEN0092-P	ZNF197	snp	3	44672663	44672663	C	T
MEN0092-P	SLC17A5	snp	6	74354233	74354233	G	A
MEN0092-P	NCAPG2	snp	7	158485620	158485620	A	G
MEN0092-P	ABCA13	snp	7	48619942	48619942	G	A
MEN0092-P	SCN9A	snp	2	167138287	167138287	C	T
MEN0092-P	SELENBP1	snp	1	151341629	151341629	C	T
MEN0092-P	TAS1R1	snp	1	6638842	6638842	C	T
MEN0092-P	PSEN2	snp	1	227071448	227071448	C	T
MEN0092-P	POLE	snp	12	133242014	133242014	G	A
MEN0092-P	PLXNB1	snp	3	48455169	48455169	G	A
MEN0092-P	FAM92B	snp	16	85143959	85143959	C	T
MEN0092-P	ASTN2	snp	9	120053712	120053712	C	T
MEN0092-P	PPFIA2	snp	12	81661851	81661851	C	T
MEN0092-P	TRAF7	snp	16	2215894	2215894	G	A
MEN0092-P	GPR124	snp	8	37690604	37690604	G	A
MEN0092-P	CHRFAM7A	snp	15	30665189	30665189	T	C
MEN0092-P	CNTROB	snp	17	7843487	7843487	G	A
MEN0092-P	MEGF8	snp	19	42866331	42866331	G	A
MEN0092-P	LMNA	snp	1	156106150	156106150	C	T
MEN0092-P	GLB1L3	snp	11	134182264	134182264	G	A
MEN0092-P	MAML3	snp	4	140640790	140640790	G	A
MEN0092-P	AMBN	snp	4	71472298	71472298	G	A
MEN0092-P	EIF4G1	snp	3	184045188	184045188	C	T
MEN0092-P	APBA1	snp	9	72131392	72131392	C	A
MEN0092-P	APOBEC1	snp	12	7802175	7802175	C	A
MEN0092-P	CGN	snp	1	151497279	151497279	C	T
MEN0092-P	PAQR8	snp	6	52268016	52268016	C	T
MEN0092-P	ZBTB3	snp	11	62520685	62520685	C	T
MEN0092-P	ARHGEF15	snp	17	8215674	8215674	G	A
MEN0092-P	SPNS1	snp	16	28993745	28993745	G	A

MEN0092-P	STAB1	snp	3	52548772	52548772	C	T
MEN0092-P	ACTN2	snp	1	236902792	236902792	A	G
MEN0092-P	MUC16	snp	19	9088981	9088981	G	A
MEN0092-P	MON1B	snp	16	77228709	77228709	C	T
MEN0092-P	IGLV1-47	snp	22	22712149	22712149	G	A
MEN0092-P	ANKRD27	snp	19	33096776	33096776	C	T
MEN0092-P	RSPH6A	snp	19	46299168	46299168	C	T
MEN0092-P	SUSD1	snp	9	114904657	114904657	T	C
MEN0092-P	RDX	snp	11	110102620	110102620	G	A
MEN0092-P	SKIV2L	snp	6	31928014	31928014	C	T
MEN0092-P	OR51E1	snp	11	4674445	4674445	G	A
MEN0092-P	RRP36	snp	6	42995168	42995168	G	A
MEN0092-P	RTP2	snp	3	187416659	187416659	G	A
MEN0092-P	SATB1	snp	3	18462365	18462365	C	T
MEN0092-P	UBE3B	snp	12	109962297	109962297	G	A
MEN0092-P	RYR3	snp	15	33905485	33905485	C	T
MEN0092-P	ATP13A3	snp	3	194152573	194152573	A	G
MEN0092-P	PCDHGA3	snp	5	140725053	140725053	G	A
MEN0092-P	CDH22	snp	20	44869727	44869727	C	T
MEN0092-P	SMYD1	snp	2	88390585	88390585	G	A
MEN0092-P	ANKS1A	snp	6	34937855	34937855	C	A
MEN0092-P	KPNA2	snp	17	66040120	66040120	G	A
MEN0092-P	ATG16L1	snp	2	234191397	234191397	C	T
MEN0092-P	PRKCD	snp	3	53223986	53223986	G	A
MEN0092-P	MRPL12	snp	17	79671425	79671425	C	G
MEN0092-P	AGAP3	snp	7	150817156	150817156	G	A
MEN0092-P	POTEC	snp	18	14513764	14513764	C	T
MEN0092-P	SIPA1L2	snp	1	232551324	232551324	C	T
MEN0092-P	AC011841.1	snp	GL000205.1	117670	117670	G	A
MEN0092-P	PROSER1	snp	13	39587833	39587833	T	C
MEN0092-P	SLC35B3	snp	6	8420990	8420990	C	T
MEN0092-P	CEP164	snp	11	117265848	117265848	G	A
MEN0092-P	CDKN2C	snp	1	51436090	51436090	A	G
MEN0092-P	ATOH8	snp	2	85991214	85991214	A	G
MEN0092-P	ABCA7	snp	19	1051944	1051944	G	A
MEN0092-P	ARID4B	snp	1	235345598	235345598	T	C
MEN0092-P	ERVW-1	snp	7	92099356	92099356	T	C
MEN0092-P	KRT83	snp	12	52714897	52714897	C	T
MEN0092-P	STXBP1	snp	9	130438979	130438979	G	C
MEN0092-P	OR2T8	snp	1	248084431	248084431	T	C
MEN0092-P	TBCEL	snp	11	120918273	120918273	G	A
MEN0092-P	CHRM4	snp	11	46407272	46407272	G	A
MEN0092-P	CKAP5	snp	11	46780906	46780906	T	C
MEN0092-P	FAT4	snp	4	126370710	126370710	G	A
MEN0092-P	MDC1	snp	6	30680233	30680233	C	T
MEN0092-P	CD163L1	snp	12	7526002	7526002	G	A
MEN0092-P	PAF1	snp	19	39880295	39880295	G	A

MEN0092-P	C9orf91	snp	9	117400932	117400932	G	A
MEN0092-P	ITM2A	snp	X	78618576	78618576	G	A
MEN0092-P	CYP7B1	snp	8	65537091	65537091	G	T
MEN0092-P	L1CAM	snp	X	153135330	153135330	G	A
MEN0092-P	TCHH	snp	1	152084615	152084615	G	A
MEN0092-P	KCNH6	snp	17	61613276	61613276	G	A
MEN0092-P	ETFB	snp	19	51857433	51857433	C	T
MEN0092-P	SLC17A2	snp	6	25913553	25913553	G	A
MEN0092-P	EXOC6	snp	10	94816757	94816757	G	A
MEN0092-P	FLG	snp	1	152283466	152283466	C	T
MEN0092-P	ADAMTS2	snp	5	178555047	178555047	C	T
MEN0092-P	QSOX1	snp	1	180163518	180163518	C	T
MEN0092-P	FGD1	snp	X	54481902	54481902	C	T
MEN0092-P	MYO5A	snp	15	52620191	52620191	C	T
MEN0092-P	DSP	snp	6	7584886	7584886	G	A
MEN0092-P	COLQ	snp	3	15529744	15529744	G	A
MEN0092-P	RNF8	snp	6	37336499	37336499	A	T
MEN0092-P	PTCH1	snp	9	98222052	98222052	C	T
MEN0092-P	BRD4	snp	19	15349771	15349771	G	A
MEN0092-P	EIF1AX	snp	X	20148638	20148638	T	C
MEN0092-P	ESR2	snp	14	64701701	64701701	C	T
MEN0092-P	HS3ST2	snp	16	22926383	22926383	G	A
MEN0092-P	FBXL3	snp	13	77595741	77595741	C	G
MEN0092-P	FBXW7	snp	4	153244137	153244137	G	A
MEN0092-P	PRKCD	snp	3	53223986	53223986	G	A
MEN0092-P	LPAR6	snp	13	48986021	48986021	T	G
MEN0092-P	NCAPG2	snp	7	158485620	158485620	A	G
MEN0092-P	CENPI	snp	X	100403130	100403130	G	A
MEN0092-P	PSG4	snp	19	43708038	43708038	G	A
MEN0092-P	TEP1	snp	14	20869673	20869673	A	G
MEN0092-P	SEC16B	snp	1	177911150	177911150	C	T
MEN0092-P	TRAF7	snp	16	2215894	2215894	G	A
MEN0092-P	TBC1D9B	snp	5	179305326	179305326	T	C
MEN0092-P	B9D1	snp	17	19263657	19263657	G	A
MEN0092-P	ANAPC2	snp	9	140082048	140082048	G	A
MEN0092-P	KLF7	snp	2	207988551	207988551	C	T
MEN0092-P	ASTN2	snp	9	120053712	120053712	C	T
MEN0092-P	FAM92B	snp	16	85143959	85143959	C	T
MEN0092-P	CYP7B1	snp	8	65537091	65537091	G	T
MEN0092-P	POLR2A	snp	17	7406782	7406782	G	A
MEN0092-P	OR2T8	snp	1	248084431	248084431	T	C
MEN0092-P	TAS1R1	snp	1	6638842	6638842	C	T
MEN0092-P	NOD2	snp	16	50746064	50746064	G	A
MEN0092-P	POLE	snp	12	133225558	133225558	T	C
MEN0092-P	ACTN2	snp	1	236902792	236902792	A	G
MEN0092-P	SLC17A5	snp	6	74354233	74354233	G	A
MEN0092-P	SLC17A5	snp	6	74354233	74354233	G	A

MEN0092-P	LYRM5	snp	12	25357101	25357101	A	C
MEN0092-P	MELK	snp	9	36651813	36651813	G	A
MEN0092-P	TRIM48	snp	11	55035844	55035844	T	C
MEN0092-P	MRPL12	snp	17	79671425	79671425	C	G
MEN0092-P	HEBP1	snp	12	13128363	13128363	C	T
MEN0092-P	AOC2	snp	17	41001304	41001304	G	A
MEN0092-P	ZNF77	snp	19	2933957	2933957	C	T
MEN0092-P	PTPRF	snp	1	44069494	44069494	C	T
MEN0092-P	FAM110A	snp	20	826309	826309	C	T
MEN0092-P	CYTH4	snp	22	37709494	37709494	G	A
MEN0092-P	KLC2	snp	11	66030380	66030380	G	A
MEN0092-P	HRH3	snp	20	60791370	60791370	A	C
MEN0092-P	POLE	snp	12	133242014	133242014	G	A
MEN0092-P	CDH22	snp	20	44869727	44869727	C	T
MEN0092-P	PACS1	snp	11	66000525	66000525	G	A
MEN0092-P	ATP2B2	snp	3	10417322	10417322	A	G
MEN0092-P	MARCO	snp	2	119750856	119750856	C	T
MEN0092-P	VPS13A	snp	9	79981670	79981670	G	A
MEN0092-P	MON1B	snp	16	77228709	77228709	C	T
MEN0092-P	THOC6	snp	16	3077247	3077247	C	T
MEN0092-P	QSOX1	snp	1	180163518	180163518	C	T
MEN0092-P	GPR52	snp	1	174417941	174417941	G	A
MEN0092-P	PPFIA2	snp	12	81661851	81661851	C	T
MEN0092-P	MTFR1L	snp	1	26158495	26158495	A	T
MEN0092-P	SAMD4A	snp	14	55243178	55243178	G	A
MEN0092-P	RNF213	snp	17	78350306	78350306	C	A
MEN0092-P	PLXNA1	snp	3	126708188	126708188	G	A
MEN0092-P	TNXB	snp	6	32037369	32037369	C	T
MEN0092-P	SMARCA4	snp	19	11132465	11132465	C	T
MEN0092-P	KCNJ11	snp	11	17408540	17408540	G	A
MEN0092-P	PLXNB1	snp	3	48455169	48455169	G	A
MEN0092-P	CTCFL	snp	20	56089711	56089711	C	T
MEN0092-P	TMEM108	snp	3	133098626	133098626	C	T
MEN0092-P	SELENBP1	snp	1	151341629	151341629	C	T
MEN0092-P	AIF1	snp	6	31583916	31583916	G	A
MEN0092-P	SPEG	snp	2	220353850	220353850	G	A
MEN0092-P	CDKN2C	snp	1	51436090	51436090	A	G
MEN0092-P	MRPL54	snp	19	3767338	3767338	C	T
MEN0092-P	LHFPL5	snp	6	35773526	35773526	G	A
MEN0092-P	NLRX1	snp	11	119045909	119045909	G	A
MEN0092-P	PALLD	snp	4	169433059	169433059	G	A
MEN0092-P	FRK	snp	6	116264287	116264287	G	A
MEN0092-P	RAPGEF4	snp	2	173901401	173901401	T	C
MEN0092-P	PKMYT1	snp	16	3029649	3029649	G	A
MEN0092-P	ABCA7	snp	19	1051944	1051944	G	A
MEN0092-P	RNF38	snp	9	36352824	36352824	G	A
MEN0092-P	USP35	snp	11	77907340	77907340	G	A

MEN0092-P	ZNF830	snp	17	33289525	33289525	T	C
MEN0092-P	UHMK1	snp	1	162492234	162492234	C	T
MEN0092-P	NOS1AP	snp	1	162313643	162313643	G	A
MEN0092-P	HERC4	snp	10	69684875	69684875	C	T
MEN0092-P	NOS1AP	snp	1	162335254	162335254	C	T
MEN0092-P	ATOH8	snp	2	85991214	85991214	A	G
MEN0092-P	HTR2B	snp	2	231974117	231974117	G	T
MEN0092-P	APOBEC1	snp	12	7802175	7802175	C	A
MEN0092-P	SKIV2L	snp	6	31928014	31928014	C	T
MEN0092-P	SDPR	snp	2	192701100	192701100	G	A
MEN0092-P	DSP	snp	6	7584886	7584886	G	A
MEN0092-P	NCAPH2	snp	22	50956007	50956007	C	T
MEN0092-P	LHFPL5	snp	6	35773661	35773661	G	A
MEN0092-P	MYOG	snp	1	203054890	203054890	G	A
MEN0092-P	TRAP1	snp	16	3727532	3727532	G	A
MEN0092-P	CSNK1G2	snp	19	1979048	1979048	C	T
MEN0092-P	PARD3B	snp	2	206037007	206037007	G	A
MEN0092-P	RPUSD3	snp	3	9883915	9883915	G	A
MEN0092-P	EGR2	snp	10	64573434	64573434	T	C
MEN0092-P	TBKBP1	snp	17	45785806	45785806	C	T
MEN0092-P	MAST1	snp	19	12981886	12981886	G	A
MEN0092-P	UHRF1BP1	snp	6	34826040	34826040	G	A
MEN0092-P	NPBWR2	snp	20	62737901	62737901	G	A
MEN0092-P	ZBTB3	snp	11	62520685	62520685	C	T
MEN0092-P	ZBTB3	snp	11	62520685	62520685	C	T
MEN0092-P	HPD	snp	12	122281735	122281735	G	A
MEN0092-P	MUC16	snp	19	9088981	9088981	G	A
MEN0092-P	IQSEC3	snp	12	247481	247481	G	A
MEN0092-P	EPPK1	snp	8	144946755	144946755	C	T
MEN0092-P	MYH1	snp	17	10419567	10419567	C	A
MEN0092-P	UNC5B	snp	10	73050713	73050713	G	A
MEN0092-P	PHF20L1	snp	8	133790127	133790127	G	C
MEN0092-P	MYO5A	snp	15	52620191	52620191	C	T
MEN0092-P	SULT1C2	snp	2	108910769	108910769	G	A
MEN0092-P	ABCA13	snp	7	48619942	48619942	G	A
MEN0092-P	PPP2R2B	snp	5	145972609	145972609	C	T
MEN0092-P	RNF8	snp	6	37336499	37336499	A	T
MEN0092-P	ANKRD27	snp	19	33096776	33096776	C	T
MEN0092-P	MRGPRX1	snp	11	18955875	18955875	G	A
MEN0092-P	SPNS1	snp	16	28993745	28993745	G	A
MEN0092-P	P2RX4	snp	12	121666668	121666668	A	C
MEN0092-P	MAPK7	snp	17	19285573	19285573	G	A
MEN0092-P	ATG16L1	snp	2	234191397	234191397	C	T
MEN0092-P	SLC35G2	snp	3	136573405	136573405	G	A
MEN0092-P	INTS3	snp	1	153742266	153742266	G	A
MEN0092-P	SCMH1	snp	1	41617308	41617308	G	A
MEN0092-P	SCMH1	snp	1	41617308	41617308	G	A

MEN0092-P	SCMH1	snp	1	41617308	41617308	G	A
MEN0092-P	SCMH1	snp	1	41617308	41617308	G	A
MEN0092-P	SCMH1	snp	1	41617308	41617308	G	A
MEN0092-P	PAQR8	snp	6	52268016	52268016	C	T
MEN0092-P	PTPN14	snp	1	214549666	214549666	C	T
MEN0092-P	ANKS1A	snp	6	34937855	34937855	C	A
MEN0092-P	AP2M1	snp	3	183899704	183899704	C	T
MEN0092-P	TMOD1	snp	9	100328233	100328233	C	T
MEN0092-P	EXOC6	snp	10	94816757	94816757	G	A
MEN0092-P	SCN9A	snp	2	167138287	167138287	C	T
MEN0092-P	OR5B21	snp	11	58275329	58275329	G	A
MEN0092-P	FGD1	snp	X	54481902	54481902	C	T
MEN0092-P	PAXBP1	snp	21	34123491	34123491	G	A
MEN0092-P	OR4N5	snp	14	20612564	20612564	C	T
MEN0092-P	TCHH	snp	1	152080468	152080468	C	T
MEN0092-P	HERC1	snp	15	63933709	63933709	C	T
MEN0092-P	RICTOR	snp	5	38950324	38950324	G	A
MEN0092-P	KPNA2	snp	17	66040120	66040120	G	A
MEN0092-P	OR51E1	snp	11	4674445	4674445	G	A
MEN0092-P	GLYR1	snp	16	4861768	4861768	C	T
MEN0092-P	AGAP3	snp	7	150817156	150817156	G	A
MEN0092-P	VARS2	snp	6	30889969	30889969	G	A
MEN0092-P	SLC6A16	snp	19	49812145	49812145	C	T
MEN0092-P	KALRN	snp	3	124193577	124193577	T	C
MEN0092-P	SLC16A7	snp	12	60168915	60168915	A	G
MEN0092-P	FAT4	snp	4	126370710	126370710	G	A
MEN0092-P	TUBGCP5	snp	15	22855271	22855271	A	G
MEN0092-P	LNPEP	snp	5	96315595	96315595	C	T
MEN0092-P	TMC1	snp	9	75406943	75406943	G	A
MEN0092-P	SLIT2	snp	4	20533609	20533609	G	A
MEN0092-P	PRADC1	snp	2	73456041	73456041	G	A
MEN0092-P	ETFB	snp	19	51857433	51857433	C	T
MEN0092-P	FGD2	snp	6	36982782	36982782	C	T
MEN0092-P	AGFG2	snp	7	100159983	100159983	G	A
MEN0092-P	SLC35B3	snp	6	8420990	8420990	C	T
MEN0092-P	COPB2	snp	3	139078167	139078167	G	A
MEN0092-P	RRP36	snp	6	42995168	42995168	G	A
MEN0092-P	KAT2A	snp	17	40265767	40265767	C	T
MEN0092-P	HS3ST4	snp	16	26147180	26147180	T	A
MEN0092-P	PCDHA8	snp	5	140222585	140222585	A	G
MEN0092-P	POLR3E	snp	16	22335966	22335966	C	T
MEN0092-P	LIMK1	snp	7	73523341	73523341	C	T
MEN0092-P	IL2RG	snp	X	70330402	70330402	G	A
MEN0092-P	FOPNL	snp	16	15967450	15967450	G	A
MEN0092-P	ZNFX1	snp	20	47879939	47879939	G	A
MEN0092-P	GRIA3	snp	X	122538739	122538739	G	A
MEN0092-P	NOS1AP	snp	1	162336911	162336911	C	T

MEN0092-P	MACF1	snp	1	39900201	39900201	A	G
MEN0092-P	WDR11	snp	10	122668104	122668104	G	A
MEN0092-P	ZNF124	snp	1	247320487	247320487	C	T
MEN0092-P	AC011841.1	snp	GL000205.1	117670	117670	G	A
MEN0092-P	PAM	snp	5	102364747	102364747	C	T
MEN0092-P	PTCH1	snp	9	98222052	98222052	C	T
MEN0092-P	ZNF197	snp	3	44672663	44672663	C	T
MEN0092-P	CHRFAM7A	snp	15	30665189	30665189	T	C
MEN0092-P	CEA	snp	19	42213763	42213763	C	T
MEN0092-P	ZC3H12B	snp	X	64722658	64722658	C	T
MEN0092-P	STXBP1	snp	9	130438979	130438979	G	C
MEN0092-P	POM121	snp	7	72398987	72398987	G	A
MEN0092-P	MAML3	snp	4	140640790	140640790	G	A
MEN0092-P	CEP63	snp	3	134268018	134268018	G	T
MEN0092-P	EPHB3	snp	3	184294803	184294803	G	A
MEN0092-P	STAB1	snp	3	52548772	52548772	C	T
MEN0092-P	RDX	snp	11	110102620	110102620	G	A
MEN0092-P	RTP2	snp	3	187416659	187416659	G	A
MEN0092-P	ERVW-1	snp	7	92099356	92099356	T	C
MEN0092-P	CGN	snp	1	151497279	151497279	C	T
MEN0092-P	DLGAP1	snp	18	3879539	3879539	C	T
MEN0092-P	PSMB11	snp	14	23512003	23512003	G	A
MEN0092-P	CNTROB	snp	17	7843487	7843487	G	A
MEN0092-P	LMNA	snp	1	156106150	156106150	C	T
MEN0092-P	ATP13A3	snp	3	194152573	194152573	A	G
MEN0092-P	CKAP5	snp	11	46780906	46780906	T	C
MEN0092-P	MRPS10	snp	6	42185554	42185554	G	A
MEN0092-P	KCNH6	snp	17	61613276	61613276	G	A
MEN0092-P	SMG8	snp	17	57290754	57290754	G	A
MEN0092-P	ARHGEF28	snp	5	73069856	73069856	G	A
MEN0092-P	TCTN2	snp	12	124175110	124175110	G	A
MEN0092-P	LRRIQ1	snp	12	85492270	85492270	G	A
MEN0092-P	TRIM46	snp	1	155152396	155152396	C	T
MEN0092-P	CD163L1	snp	12	7526002	7526002	G	A
MEN0092-P	TBCEL	snp	11	120918273	120918273	G	A
MEN0092-P	GPR124	snp	8	37690604	37690604	G	A
MEN0092-P	EML1	snp	14	100377846	100377846	C	T
MEN0092-P	IGLV1-47	snp	22	22712149	22712149	G	A
MEN0092-P	ADAMTS6	snp	5	64587158	64587158	C	A
MEN0092-P	ANKRD11	snp	16	89349513	89349513	G	A
MEN0092-P	ZNF716	snp	7	57510026	57510026	G	A
MEN0092-P	NID1	snp	1	236176855	236176855	C	T
MEN0092-P	CEP164	snp	11	117265848	117265848	G	A
MEN0092-P	HIVEP1	snp	6	12122908	12122908	G	C
MEN0092-P	TTLL9	snp	20	30510805	30510805	G	A
MEN0092-P	TNXB	snp	6	32010287	32010287	C	T
MEN0092-P	ADAMTS2	snp	5	178555047	178555047	C	T

MEN0092-P	BMP8B	snp	1	40229458	40229458	C	T
MEN0092-P	CYP4F22	snp	19	15659011	15659011	C	T
MEN0092-P	PROSER1	snp	13	39587833	39587833	T	C
MEN0092-P	MEGF8	snp	19	42866331	42866331	G	A
MEN0092-P	SLC32A1	snp	20	37356192	37356192	G	A
MEN0092-P	SIRPD	snp	20	1532445	1532445	G	A
MEN0092-P	MTSS1L	snp	16	70712220	70712220	G	A
MEN0092-P	NEB	snp	2	152381087	152381087	G	C
MEN0092-P	RYR3	snp	15	33905485	33905485	C	T
MEN0092-P	POM121C	snp	7	75050828	75050828	C	T
MEN0092-P	SMYD1	snp	2	88390585	88390585	G	A
MEN0092-P	RGAG1	snp	X	109697237	109697237	C	T
MEN0092-P	PRC1	snp	15	91517428	91517428	C	T
MEN0092-P	ZNF587	snp	19	58370321	58370321	C	T
MEN0092-P	DYRK2	snp	12	68051937	68051937	C	T
MEN0092-P	GLB1L3	snp	11	134182264	134182264	G	A
MEN0092-P	SUSD1	snp	9	114904657	114904657	T	C
MEN0092-P	AMBN	snp	4	71472298	71472298	G	A
MEN0092-P	PAF1	snp	19	39880295	39880295	G	A
MEN0092-P	L1CAM	snp	X	153135330	153135330	G	A
MEN0092-P	RMND5B	snp	5	177569902	177569902	C	T
MEN0092-P	C1orf222	snp	1	1854450	1854450	G	A
MEN0092-P	PCDHGA3	snp	5	140725053	140725053	G	A
MEN0092-P	ARHGEF15	snp	17	8215674	8215674	G	A
MEN0092-P	BRD4	snp	19	15349771	15349771	G	A
MEN0092-P	ARNTL	snp	11	13375965	13375965	G	A
MEN0092-P	MSTO1	snp	1	155717640	155717640	C	T
MEN0092-P	LRRC37B	snp	17	30349744	30349744	G	A
MEN0092-P	KIAA1683	snp	19	18376959	18376959	G	C
MEN0092-P	ATP2A1	snp	16	28911947	28911947	C	T
MEN0092-P	POLQ	snp	3	121208882	121208882	T	C
MEN0092-P	ERN1	snp	17	62121527	62121527	G	A
MEN0092-P	RYR3	snp	15	33926811	33926811	C	T
MEN0092-P	RSPH6A	snp	19	46299168	46299168	C	T
MEN0092-P	GBAS	snp	7	56065092	56065092	G	A
MEN0092-P	EIF4G1	snp	3	184045188	184045188	C	T
MEN0092-P	ZNF711	snp	X	84510549	84510549	G	A
MEN0092-P	DEAF1	snp	11	654033	654033	C	T
MEN0092-P	SLC47A1	snp	17	19480670	19480670	C	T
MEN0092-P	TTN	snp	2	179464170	179464170	G	A
MEN0092-P	ARHGAP5	snp	14	32561286	32561286	A	G
MEN0092-P	ZNF213	snp	16	3189049	3189049	G	A
MEN0092-P	DHRS4L2	snp	14	24464325	24464325	G	A
MEN0092-P	UBE3B	snp	12	109962297	109962297	G	A
MEN0092-P	NFIC	snp	19	3382127	3382127	C	T
MEN0092-P	ERVFRD-1	snp	6	11104921	11104921	C	T
MEN0092-P	KRT83	snp	12	52714897	52714897	C	T

MEN0092-P	COLQ	snp	3	15529744	15529744	G	A
MEN0092-P	BCL9	snp	1	147095868	147095868	T	C
MEN0092-P	ARID4B	snp	1	235345598	235345598	T	C
MEN0092-P	CTC-432M15.3	snp	5	130840366	130840366	G	A
MEN0092-P	FCGBP	snp	19	40364333	40364333	C	T
MEN0092-P	CA14	snp	1	150233929	150233929	A	G
MEN0092-P	SIPA1L2	snp	1	232551324	232551324	C	T
MEN0092-P	PSEN2	snp	1	227071448	227071448	C	T
MEN0092-P	ZFHX4	snp	8	77616634	77616634	G	A
MEN0092-P	ITM2A	snp	X	78618576	78618576	G	A
MEN0092-P	RBBP6	snp	16	24578601	24578601	C	T
MEN0092-P	CHRM4	snp	11	46407272	46407272	G	A
MEN0092-P	SATB1	snp	3	18462365	18462365	C	T
MEN0092-P	SLC17A2	snp	6	25913553	25913553	G	A
MEN0092-P	SON	snp	21	34931986	34931986	G	A
MEN0092-P	FLG	snp	1	152283466	152283466	C	T
MEN0092-P	SVEP1	snp	9	113173903	113173903	C	T
MEN0092-P	MDC1	snp	6	30680233	30680233	C	T
MEN0092-P	C9orf91	snp	9	117400932	117400932	G	A
MEN0092-P	ME3	snp	11	86209022	86209022	C	A
MEN0092-P	RTN2	snp	19	45998201	45998201	C	T
MEN0092-P	FLG	snp	1	152277116	152277116	C	T
MEN0092-P	EIF1AX	snp	X	20148638	20148638	T	C
MEN0092-P	APBA1	snp	9	72131392	72131392	C	A
MEN0092-P	POTEC	snp	18	14513764	14513764	C	T
MEN0092-P	TCHH	snp	1	152084615	152084615	G	A
MEN0093-P0	CHRDL2	snp	11	74421931	74421931	G	A
MEN0093-P0	NBPF9	snp	1	144828707	144828707	G	T
MEN0093-P0	NBPF10	snp	1	145296448	145296448	T	A
MEN0093-P0	KLHL26	snp	19	18779922	18779922	A	C
MEN0093-P0	KCNK9	snp	8	140630518	140630518	G	A
MEN0093-P0	NBPF9	snp	1	144828554	144828554	C	G
MEN0093-P0	TRBV6-7	snp	7	142143907	142143907	T	C
MEN0093-P0	PARG	snp	10	51093329	51093329	C	T
MEN0093-P0	INSR	snp	19	7142913	7142913	C	T
MEN0093-P0	NBPF9	snp	1	144828624	144828624	A	T
MEN0093-P0	KRT6B	snp	12	52845601	52845601	C	T
MEN0093-P0	MUC6	snp	11	1017546	1017546	T	C
MEN0093-P0	TRBV6-7	snp	7	142143839	142143839	G	A
MEN0093-P0	ANKRD36C	snp	2	96521862	96521862	T	A
MEN0093-P0	MAN2A1	snp	5	109178148	109178148	C	G
MEN0093-P0	FRG1B	snp	20	29625875	29625875	T	C
MEN0093-P0	ANAPC2	snp	9	140082133	140082133	G	C
MEN0093-P0	ACTR3C	snp	7	149983565	149983565	T	C
MEN0093-P0	RGPD3	snp	2	107049681	107049681	T	C
MEN0093-P0	TRAPPC8	snp	18	29432467	29432467	G	A
MEN0093-P0	TRIM48	snp	11	55032684	55032684	T	C

MEN0093-P0	FRG1B	snp	20	29625892	29625892	T	C
MEN0093-P0	MBD6	snp	12	57922477	57922477	G	C
MEN0093-P0	FAT4	snp	4	126371885	126371885	T	G
MEN0093-P0	THBS1	snp	15	39883745	39883745	A	G
MEN0093-P0	ANKRD36	snp	2	97869931	97869931	A	T
MEN0093-P0	RFPL3	snp	22	32756703	32756703	C	T
MEN0093-P0	KRTAP4-7	snp	17	39240627	39240627	T	C
MEN0093-P0	PRMT10	snp	4	148560188	148560188	G	A
MEN0093-P0	FRG1B	snp	20	29623218	29623218	A	G
MEN0093-P0	ADAMTS6	snp	5	64569214	64569214	G	A
MEN0093-P0	IGHV4OR15-8	snp	15	22473167	22473167	T	C
MEN0093-P0	DFNA5	snp	7	24745946	24745946	T	C
MEN0093-P0	AL592183.1	snp	GL000219.1	83307	83307	G	T
MEN0093-P0	TMEM91	snp	19	41889651	41889651	A	G
MEN0093-P0	PRMT8	snp	12	3649787	3649787	T	C
MEN0093-P0	RAG1	snp	11	36595333	36595333	G	A
MEN0093-P0	NBPF10	snp	1	145368524	145368524	A	T
MEN0093-P0	ZAN	snp	7	100350474	100350474	T	C
MEN0093-P0	KRTAP5-4	snp	11	1642989	1642989	C	A
MEN0093-P0	ZAN	snp	7	100350126	100350126	G	A
MEN0093-P0	ARHGEF12	snp	11	120352011	120352011	G	A
MEN0093-P0	TFAP4	snp	16	4310225	4310225	T	G
MEN0093-P0	PCLO	snp	7	82784471	82784471	A	G
MEN0093-P0	MDK	snp	11	46404331	46404331	C	G
MEN0093-P0	NBPF9	snp	1	144828707	144828707	G	T
MEN0093-P0	INSR	snp	19	7142913	7142913	C	T
MEN0093-P0	PRMT10	snp	4	148560188	148560188	G	A
MEN0093-P0	TRBV6-7	snp	7	142143907	142143907	T	C
MEN0093-P0	KLHL26	snp	19	18779922	18779922	A	C
MEN0093-P0	CHRDL2	snp	11	74421931	74421931	G	A
MEN0093-P0	ACTR3C	snp	7	149983565	149983565	T	C
MEN0093-P0	ANAPC2	snp	9	140082133	140082133	G	C
MEN0093-P0	PARG	snp	10	51093329	51093329	C	T
MEN0093-P0	ADAMTS6	snp	5	64569214	64569214	G	A
MEN0093-P0	TMEM91	snp	19	41889651	41889651	A	G
MEN0093-P0	NBPF10	snp	1	145368524	145368524	A	T
MEN0093-P0	FAT4	snp	4	126371885	126371885	T	G
MEN0093-P0	MAN2A1	snp	5	109178148	109178148	C	G
MEN0093-P0	FRG1B	snp	20	29625875	29625875	T	C
MEN0093-P0	NBPF9	snp	1	144828554	144828554	C	G
MEN0093-P0	IGHV4OR15-8	snp	15	22473167	22473167	T	C
MEN0093-P0	RFPL3	snp	22	32756703	32756703	C	T
MEN0093-P0	MUC6	snp	11	1017546	1017546	T	C
MEN0093-P0	NBPF9	snp	1	144828624	144828624	A	T
MEN0093-P0	DFNA5	snp	7	24745946	24745946	T	C
MEN0093-P0	TRIM48	snp	11	55032684	55032684	T	C
MEN0093-P0	ARHGEF12	snp	11	120352011	120352011	G	A

MEN0093-P0	NBPF10	snp	1	145296448	145296448	T	A
MEN0093-P0	AL592183.1	snp	GL000219.1	83307	83307	G	T
MEN0093-P0	RAG1	snp	11	36595333	36595333	G	A
MEN0093-P0	RGPD3	snp	2	107049681	107049681	T	C
MEN0093-P0	FRG1B	snp	20	29623218	29623218	A	G
MEN0093-P0	ANKRD36C	snp	2	96521862	96521862	T	A
MEN0093-P0	MDK	snp	11	46404331	46404331	C	G
MEN0093-P0	PRMT8	snp	12	3649787	3649787	T	C
MEN0093-P0	TRBV6-7	snp	7	142143839	142143839	G	A
MEN0093-P0	FRG1B	snp	20	29625892	29625892	T	C
MEN0093-P0	TFAP4	snp	16	4310225	4310225	T	G
MEN0093-P0	TRAPPC8	snp	18	29432467	29432467	G	A
MEN0093-P0	KRT6B	snp	12	52845601	52845601	C	T
MEN0093-P0	ANKRD36	snp	2	97869931	97869931	A	T
MEN0093-P0	KCNK9	snp	8	140630518	140630518	G	A
MEN0093-P0	ZAN	snp	7	100350126	100350126	G	A
MEN0093-P0	THBS1	snp	15	39883745	39883745	A	G
MEN0093-P0	MBD6	snp	12	57922477	57922477	G	C
MEN0093-P0	KRTAP5-4	snp	11	1642989	1642989	C	A
MEN0093-P0	KRTAP4-7	snp	17	39240627	39240627	T	C
MEN0093-P0	ZAN	snp	7	100350474	100350474	T	C
MEN0093-P0	PCLO	snp	7	82784471	82784471	A	G
MEN0093-P1	CHRDL2	snp	11	74421931	74421931	G	A
MEN0093-P1	FRG1B	snp	20	29625941	29625941	A	T
MEN0093-P1	NBPF9	snp	1	144828707	144828707	G	T
MEN0093-P1	FANCD2	snp	3	10136015	10136015	C	T
MEN0093-P1	OR10A4	snp	11	6898510	6898510	C	A
MEN0093-P1	NBPF10	snp	1	145296448	145296448	T	A
MEN0093-P1	BEST3	snp	12	70049492	70049492	G	A
MEN0093-P1	KLHL26	snp	19	18779922	18779922	A	C
MEN0093-P1	TRBV6-7	snp	7	142143907	142143907	T	C
MEN0093-P1	AHNAK2	snp	14	105414838	105414838	T	C
MEN0093-P1	KCNA5	snp	12	5154148	5154148	C	T
MEN0093-P1	NBPF9	snp	1	144828624	144828624	A	T
MEN0093-P1	ANKRD36C	snp	2	96521777	96521777	T	C
MEN0093-P1	MUC6	snp	11	1017546	1017546	T	C
MEN0093-P1	XPOT	snp	12	64815015	64815015	G	A
MEN0093-P1	HRH1	snp	3	11301963	11301963	G	A
MEN0093-P1	ANKRD36C	snp	2	96521862	96521862	T	A
MEN0093-P1	ANKRD36C	snp	2	96521834	96521834	T	C
MEN0093-P1	FRG1B	snp	20	29625875	29625875	T	C
MEN0093-P1	ISY1	snp	3	128878018	128878018	G	A
MEN0093-P1	POM121	snp	7	72398976	72398976	A	G
MEN0093-P1	ANAPC2	snp	9	140082133	140082133	G	C
MEN0093-P1	SPAG8	snp	9	35811816	35811816	G	A
MEN0093-P1	FRG1B	snp	20	29625885	29625885	A	T
MEN0093-P1	RGPD3	snp	2	107049681	107049681	T	C

MEN0093-P1	FAT4	snp	4	126371885	126371885	T	G
MEN0093-P1	STXBP5	snp	6	147704087	147704087	G	C
MEN0093-P1	FARP1	snp	13	98896867	98896867	T	G
MEN0093-P1	POTEC	snp	18	14543063	14543063	A	C
MEN0093-P1	ANKRD36	snp	2	97869931	97869931	A	T
MEN0093-P1	RFPL3	snp	22	32756703	32756703	C	T
MEN0093-P1	KRTAP4-7	snp	17	39240627	39240627	T	C
MEN0093-P1	NPIPB6	snp	16	28354353	28354353	T	G
MEN0093-P1	FRG1B	snp	20	29623218	29623218	A	G
MEN0093-P1	PIK3CB	snp	3	138478172	138478172	A	G
MEN0093-P1	IGHV4OR15-8	snp	15	22473167	22473167	T	C
MEN0093-P1	MUC5B	snp	11	1264394	1264394	T	C
MEN0093-P1	PRMT8	snp	12	3649787	3649787	T	C
MEN0093-P1	RAG1	snp	11	36595333	36595333	G	A
MEN0093-P1	ANKRD36C	snp	2	96593025	96593025	C	T
MEN0093-P1	KRTAP5-4	snp	11	1642989	1642989	C	A
MEN0093-P1	ZAN	snp	7	100349865	100349865	T	C
MEN0093-P1	MUC21	snp	6	30954806	30954806	T	C
MEN0093-P1	MUC21	snp	6	30955025	30955025	G	A
MEN0093-P1	NBPF9	snp	1	144825519	144825519	T	G
MEN0093-P1	ARHGEF12	snp	11	120352011	120352011	G	A
MEN0093-P1	OR10A4	snp	11	6898510	6898510	C	A
MEN0093-P1	NBPF9	snp	1	144828707	144828707	G	T
MEN0093-P1	SPAG8	snp	9	35811816	35811816	G	A
MEN0093-P1	BEST3	snp	12	70049492	70049492	G	A
MEN0093-P1	TRBV6-7	snp	7	142143907	142143907	T	C
MEN0093-P1	KLHL26	snp	19	18779922	18779922	A	C
MEN0093-P1	CHRDL2	snp	11	74421931	74421931	G	A
MEN0093-P1	HRH1	snp	3	11301963	11301963	G	A
MEN0093-P1	ANAPC2	snp	9	140082133	140082133	G	C
MEN0093-P1	MUC5B	snp	11	1264394	1264394	T	C
MEN0093-P1	POM121	snp	7	72398976	72398976	A	G
MEN0093-P1	XPOT	snp	12	64815015	64815015	G	A
MEN0093-P1	ISY1	snp	3	128878018	128878018	G	A
MEN0093-P1	AHNAK2	snp	14	105414838	105414838	T	C
MEN0093-P1	FRG1B	snp	20	29625875	29625875	T	C
MEN0093-P1	FARP1	snp	13	98896867	98896867	T	G
MEN0093-P1	PIK3CB	snp	3	138478172	138478172	A	G
MEN0093-P1	FANCD2	snp	3	10136015	10136015	C	T
MEN0093-P1	FAT4	snp	4	126371885	126371885	T	G
MEN0093-P1	STXBP5	snp	6	147704087	147704087	G	C
MEN0093-P1	IGHV4OR15-8	snp	15	22473167	22473167	T	C
MEN0093-P1	RFPL3	snp	22	32756703	32756703	C	T
MEN0093-P1	MUC6	snp	11	1017546	1017546	T	C
MEN0093-P1	NBPF9	snp	1	144828624	144828624	A	T
MEN0093-P1	MUC21	snp	6	30954806	30954806	T	C
MEN0093-P1	FRG1B	snp	20	29625941	29625941	A	T

MEN0093-P1	ARHGEF12	snp	11	120352011	120352011	G	A
MEN0093-P1	NBPF10	snp	1	145296448	145296448	T	A
MEN0093-P1	ANKRD36C	snp	2	96593025	96593025	C	T
MEN0093-P1	RAG1	snp	11	36595333	36595333	G	A
MEN0093-P1	NBPF9	snp	1	144825519	144825519	T	G
MEN0093-P1	RGPD3	snp	2	107049681	107049681	T	C
MEN0093-P1	FRG1B	snp	20	29623218	29623218	A	G
MEN0093-P1	ANKRD36C	snp	2	96521834	96521834	T	C
MEN0093-P1	MUC21	snp	6	30955025	30955025	G	A
MEN0093-P1	ANKRD36C	snp	2	96521777	96521777	T	C
MEN0093-P1	ANKRD36C	snp	2	96521862	96521862	T	A
MEN0093-P1	PRMT8	snp	12	3649787	3649787	T	C
MEN0093-P1	POTEC	snp	18	14543063	14543063	A	C
MEN0093-P1	KCNA5	snp	12	5154148	5154148	C	T
MEN0093-P1	ANKRD36	snp	2	97869931	97869931	A	T
MEN0093-P1	ZAN	snp	7	100349865	100349865	T	C
MEN0093-P1	NPIPB6	snp	16	28354353	28354353	T	G
MEN0093-P1	KRTAP5-4	snp	11	1642989	1642989	C	A
MEN0093-P1	KRTAP4-7	snp	17	39240627	39240627	T	C
MEN0093-P4	ZNF214	snp	11	7022096	7022096	T	A
MEN0093-P4	CHRDL2	snp	11	74421931	74421931	G	A
MEN0093-P4	HYDIN	snp	16	71163638	71163638	G	T
MEN0093-P4	ALAS1	snp	3	52245436	52245436	C	T
MEN0093-P4	FRG1B	snp	20	29625941	29625941	A	T
MEN0093-P4	NBPF9	snp	1	144828707	144828707	G	T
MEN0093-P4	RBM39	snp	20	34309742	34309742	G	A
MEN0093-P4	HK3	snp	5	176314566	176314566	G	C
MEN0093-P4	NEU1	snp	6	31827548	31827548	T	C
MEN0093-P4	NBPF10	snp	1	145296448	145296448	T	A
MEN0093-P4	SLC6A16	snp	19	49793546	49793546	C	T
MEN0093-P4	GPR125	snp	4	22389626	22389626	G	C
MEN0093-P4	KLHL26	snp	19	18779922	18779922	A	C
MEN0093-P4	BRMS1	snp	11	66105270	66105270	T	G
MEN0093-P4	DBF4	snp	7	87516713	87516713	G	T
MEN0093-P4	PPAP2A	snp	5	54721815	54721815	G	A
MEN0093-P4	PGM2	snp	4	37839156	37839156	C	T
MEN0093-P4	KCNA5	snp	12	5154148	5154148	C	T
MEN0093-P4	NBPF9	snp	1	144828624	144828624	A	T
MEN0093-P4	MUC6	snp	11	1017546	1017546	T	C
MEN0093-P4	MANEA	snp	6	96054057	96054057	G	A
MEN0093-P4	FRG1B	snp	20	29625875	29625875	T	C
MEN0093-P4	RAB3GAP2	snp	1	220330670	220330670	T	A
MEN0093-P4	ANAPC2	snp	9	140082133	140082133	G	C
MEN0093-P4	PCDHB7	snp	5	140553891	140553891	C	G
MEN0093-P4	STS	snp	X	7268006	7268006	G	A
MEN0093-P4	DEFB113	snp	6	49936566	49936566	T	C
MEN0093-P4	IPO4	snp	14	24653085	24653085	C	T

MEN0093-P4	RGPD3	snp	2	107049681	107049681	T	C
MEN0093-P4	FRG1B	snp	20	29625892	29625892	T	C
MEN0093-P4	TTN	snp	2	179582052	179582052	C	T
MEN0093-P4	NRF1	snp	7	129350395	129350395	C	A
MEN0093-P4	FAT4	snp	4	126371885	126371885	T	G
MEN0093-P4	S100Z	snp	5	76171202	76171202	G	T
MEN0093-P4	ZC3H13	snp	13	46544548	46544548	G	A
MEN0093-P4	ANKRD36	snp	2	97869931	97869931	A	T
MEN0093-P4	RFPL3	snp	22	32756703	32756703	C	T
MEN0093-P4	KIAA2013	snp	1	11985396	11985396	A	G
MEN0093-P4	KRTAP4-7	snp	17	39240627	39240627	T	C
MEN0093-P4	RELA	snp	11	65423158	65423158	C	T
MEN0093-P4	ECM1	snp	1	150483520	150483520	G	A
MEN0093-P4	ZNF90	snp	19	20229970	20229970	C	A
MEN0093-P4	KRT37	snp	17	39578646	39578646	C	T
MEN0093-P4	FRG1B	snp	20	29623218	29623218	A	G
MEN0093-P4	GIF	snp	11	59603378	59603378	C	T
MEN0093-P4	IGHV4OR15-8	snp	15	22473167	22473167	T	C
MEN0093-P4	IL10RB	snp	21	34652075	34652075	G	C
MEN0093-P4	SLX4	snp	16	3633345	3633345	G	C
MEN0093-P4	AL592183.1	snp	GL000219.1	83307	83307	G	T
MEN0093-P4	PRMT8	snp	12	3649787	3649787	T	C
MEN0093-P4	RAG1	snp	11	36595333	36595333	G	A
MEN0093-P4	EGFL6	snp	X	13626473	13626473	C	T
MEN0093-P4	NOBOX	snp	7	144098459	144098459	C	T
MEN0093-P4	KRTAP5-4	snp	11	1642989	1642989	C	A
MEN0093-P4	ESAM	snp	11	124628279	124628279	A	G
MEN0093-P4	SPACA7	snp	13	113088857	113088857	T	G
MEN0093-P4	FAM183B	snp	7	38725208	38725208	C	T
MEN0093-P4	SMNDC1	snp	10	112058542	112058542	T	G
MEN0093-P4	ARHGEF12	snp	11	120352011	120352011	G	A
MEN0093-P4	HOXD8	snp	2	176995618	176995618	A	C
MEN0093-P4	HNRNPC	snp	14	21679596	21679596	T	C
MEN0093-P4	RGPD8	snp	2	113127775	113127775	G	C
MEN0093-P4	PGM2	snp	4	37839156	37839156	C	T
MEN0093-P4	SLC6A16	snp	19	49793546	49793546	C	T
MEN0093-P4	NBPF9	snp	1	144828707	144828707	G	T
MEN0093-P4	GIF	snp	11	59603378	59603378	C	T
MEN0093-P4	MANEA	snp	6	96054057	96054057	G	A
MEN0093-P4	BRMS1	snp	11	66105270	66105270	T	G
MEN0093-P4	ZNF90	snp	19	20229970	20229970	C	A
MEN0093-P4	HK3	snp	5	176314566	176314566	G	C
MEN0093-P4	IPO4	snp	14	24653085	24653085	C	T
MEN0093-P4	KLHL26	snp	19	18779922	18779922	A	C
MEN0093-P4	NEU1	snp	6	31827548	31827548	T	C
MEN0093-P4	CHRDL2	snp	11	74421931	74421931	G	A
MEN0093-P4	RAB3GAP2	snp	1	220330670	220330670	T	A

MEN0093-P4	DBF4	snp	7	87516713	87516713	G	T
MEN0093-P4	RBM39	snp	20	34309742	34309742	G	A
MEN0093-P4	ALAS1	snp	3	52245436	52245436	C	T
MEN0093-P4	ANAPC2	snp	9	140082133	140082133	G	C
MEN0093-P4	PCDHB7	snp	5	140553891	140553891	C	G
MEN0093-P4	S100Z	snp	5	76171202	76171202	G	T
MEN0093-P4	HYDIN	snp	16	71163638	71163638	G	T
MEN0093-P4	PPAP2A	snp	5	54721815	54721815	G	A
MEN0093-P4	SLX4	snp	16	3633345	3633345	G	C
MEN0093-P4	FAT4	snp	4	126371885	126371885	T	G
MEN0093-P4	NRF1	snp	7	129350395	129350395	C	A
MEN0093-P4	KIAA2013	snp	1	11985396	11985396	A	G
MEN0093-P4	RGPD8	snp	2	113127775	113127775	G	C
MEN0093-P4	FRG1B	snp	20	29625875	29625875	T	C
MEN0093-P4	KRT37	snp	17	39578646	39578646	C	T
MEN0093-P4	IL10RB	snp	21	34652075	34652075	G	C
MEN0093-P4	IGHV4OR15-8	snp	15	22473167	22473167	T	C
MEN0093-P4	RFPL3	snp	22	32756703	32756703	C	T
MEN0093-P4	TTN	snp	2	179582052	179582052	C	T
MEN0093-P4	MUC6	snp	11	1017546	1017546	T	C
MEN0093-P4	NBPF9	snp	1	144828624	144828624	A	T
MEN0093-P4	FAM183B	snp	7	38725208	38725208	C	T
MEN0093-P4	DEFB113	snp	6	49936566	49936566	T	C
MEN0093-P4	FRG1B	snp	20	29625941	29625941	A	T
MEN0093-P4	ARHGEF12	snp	11	120352011	120352011	G	A
MEN0093-P4	NBPF10	snp	1	145296448	145296448	T	A
MEN0093-P4	AL592183.1	snp	GL000219.1	83307	83307	G	T
MEN0093-P4	STS	snp	X	7268006	7268006	G	A
MEN0093-P4	RAG1	snp	11	36595333	36595333	G	A
MEN0093-P4	NOBOX	snp	7	144098459	144098459	C	T
MEN0093-P4	RGPD3	snp	2	107049681	107049681	T	C
MEN0093-P4	FRG1B	snp	20	29623218	29623218	A	G
MEN0093-P4	GPR125	snp	4	22389626	22389626	G	C
MEN0093-P4	RELA	snp	11	65423158	65423158	C	T
MEN0093-P4	ZNF214	snp	11	7022096	7022096	T	A
MEN0093-P4	ESAM	snp	11	124628279	124628279	A	G
MEN0093-P4	EGFL6	snp	X	13626473	13626473	C	T
MEN0093-P4	PRMT8	snp	12	3649787	3649787	T	C
MEN0093-P4	ECM1	snp	1	150483520	150483520	G	A
MEN0093-P4	SMNDC1	snp	10	112058542	112058542	T	G
MEN0093-P4	FRG1B	snp	20	29625892	29625892	T	C
MEN0093-P4	HOXD8	snp	2	176995618	176995618	A	C
MEN0093-P4	KCNA5	snp	12	5154148	5154148	C	T
MEN0093-P4	ANKRD36	snp	2	97869931	97869931	A	T
MEN0093-P4	SPACA7	snp	13	113088857	113088857	T	G
MEN0093-P4	KRTAP5-4	snp	11	1642989	1642989	C	A
MEN0093-P4	HNRNPC	snp	14	21679596	21679596	T	C

MEN0093-P4	KRTAP4-7	snp	17	39240627	39240627	T	C
MEN0093-P4	ZC3H13	snp	13	46544548	46544548	G	A
MEN0093G-P2	CHRDL2	snp	11	74421931	74421931	G	A
MEN0093G-P2	NLRP9	snp	19	56244023	56244023	G	T
MEN0093G-P2	TCHH	snp	1	152082484	152082484	G	T
MEN0093G-P2	KLHL26	snp	19	18779922	18779922	A	C
MEN0093G-P2	BAZ2A	snp	12	56994060	56994060	G	A
MEN0093G-P2	ZNF417	snp	19	58420058	58420058	C	A
MEN0093G-P2	KCNA5	snp	12	5154148	5154148	C	T
MEN0093G-P2	KAT6A	snp	8	41832313	41832313	T	C
MEN0093G-P2	MUC6	snp	11	1017546	1017546	T	C
MEN0093G-P2	POLR2J3	snp	7	102207089	102207089	T	C
MEN0093G-P2	ANAPC2	snp	9	140082133	140082133	G	C
MEN0093G-P2	MUC17	snp	7	100676784	100676784	A	G
MEN0093G-P2	AC024580.1	snp	19	56662733	56662733	G	A
MEN0093G-P2	OR4M1	snp	14	20249164	20249164	A	T
MEN0093G-P2	SPAG8	snp	9	35811816	35811816	G	A
MEN0093G-P2	EZH1	snp	17	40876331	40876331	T	G
MEN0093G-P2	TNRC6A	snp	16	24815575	24815575	G	C
MEN0093G-P2	MUC4	snp	3	195508940	195508940	C	G
MEN0093G-P2	FAT4	snp	4	126371885	126371885	T	G
MEN0093G-P2	AC024580.1	snp	19	56662712	56662712	G	A
MEN0093G-P2	ZAN	snp	7	100349947	100349947	C	T
MEN0093G-P2	UBALD1	snp	16	4659758	4659758	T	G
MEN0093G-P2	FAM186A	snp	12	50746270	50746270	C	G
MEN0093G-P2	LRRC37B	snp	17	30372764	30372764	T	A
MEN0093G-P2	ZAN	snp	7	100350036	100350036	C	A
MEN0093G-P2	TRIOBP	snp	22	38120503	38120503	G	A
MEN0093G-P2	FAM186A	snp	12	50746092	50746092	A	G
MEN0093G-P2	IVL	snp	1	152883164	152883164	T	G
MEN0093G-P2	FAM186A	snp	12	50746699	50746699	T	G
MEN0093G-P2	MUC17	snp	7	100680117	100680117	T	C
MEN0093G-P2	RAG1	snp	11	36595333	36595333	G	A
MEN0093G-P2	DNAJC9	snp	10	75006934	75006934	T	C
MEN0093G-P2	FRG1B	snp	20	29631566	29631566	A	C
MEN0093G-P2	ITSN1	snp	21	35258689	35258689	C	G
MEN0093G-P2	MUC4	snp	3	195514297	195514297	A	G
MEN0093G-P2	MUC4	snp	3	195508630	195508630	T	G
MEN0093G-P2	MUC17	snp	7	100679214	100679214	T	C
MEN0093G-P2	ARHGEF12	snp	11	120352011	120352011	G	A
MEN0093G-P2	MUC2	snp	11	1093232	1093232	T	G
MEN0093G-P2	ZAN	snp	7	100350520	100350520	C	T
MEN0093G-P2	RP1L1	snp	8	10466031	10466031	A	C
MEN0093G-P2	ZAN	snp	7	100350010	100350010	C	T
MEN0093G-P2	SPAG8	snp	9	35811816	35811816	G	A
MEN0093G-P2	NLRP9	snp	19	56244023	56244023	G	T
MEN0093G-P2	KLHL26	snp	19	18779922	18779922	A	C

MEN0093G-P2	CHRDL2	snp	11	74421931	74421931	G	A
MEN0093G-P2	MUC17	snp	7	100676784	100676784	A	G
MEN0093G-P2	OR4M1	snp	14	20249164	20249164	A	T
MEN0093G-P2	ANAPC2	snp	9	140082133	140082133	G	C
MEN0093G-P2	AC024580.1	snp	19	56662712	56662712	G	A
MEN0093G-P2	EZH1	snp	17	40876331	40876331	T	G
MEN0093G-P2	ZNF417	snp	19	58420058	58420058	C	A
MEN0093G-P2	FAT4	snp	4	126371885	126371885	T	G
MEN0093G-P2	MUC4	snp	3	195508940	195508940	C	G
MEN0093G-P2	FAM186A	snp	12	50746092	50746092	A	G
MEN0093G-P2	MUC17	snp	7	100680117	100680117	T	C
MEN0093G-P2	MUC17	snp	7	100679214	100679214	T	C
MEN0093G-P2	BAZ2A	snp	12	56994060	56994060	G	A
MEN0093G-P2	MUC6	snp	11	1017546	1017546	T	C
MEN0093G-P2	FAM186A	snp	12	50746699	50746699	T	G
MEN0093G-P2	ARHGEF12	snp	11	120352011	120352011	G	A
MEN0093G-P2	FAM186A	snp	12	50746270	50746270	C	G
MEN0093G-P2	RAG1	snp	11	36595333	36595333	G	A
MEN0093G-P2	RP1L1	snp	8	10466031	10466031	A	C
MEN0093G-P2	POLR2J3	snp	7	102207089	102207089	T	C
MEN0093G-P2	LRRC37B	snp	17	30372764	30372764	T	A
MEN0093G-P2	ZAN	snp	7	100350520	100350520	C	T
MEN0093G-P2	DNAJC9	snp	10	75006934	75006934	T	C
MEN0093G-P2	MUC4	snp	3	195508630	195508630	T	G
MEN0093G-P2	ZAN	snp	7	100350010	100350010	C	T
MEN0093G-P2	KCNA5	snp	12	5154148	5154148	C	T
MEN0093G-P2	ITSN1	snp	21	35258689	35258689	C	G
MEN0093G-P2	KAT6A	snp	8	41832313	41832313	T	C
MEN0093G-P2	MUC4	snp	3	195514297	195514297	A	G
MEN0093G-P2	TNRC6A	snp	16	24815575	24815575	G	C
MEN0093G-P2	MUC2	snp	11	1093232	1093232	T	G
MEN0093G-P2	FRG1B	snp	20	29631566	29631566	A	C
MEN0093G-P2	ZAN	snp	7	100349947	100349947	C	T
MEN0093G-P2	UBALD1	snp	16	4659758	4659758	T	G
MEN0093G-P2	ZAN	snp	7	100350036	100350036	C	A
MEN0093G-P2	IVL	snp	1	152883164	152883164	T	G
MEN0093G-P2	TCHH	snp	1	152082484	152082484	G	T
MEN0093G-P2	TRIOBP	snp	22	38120503	38120503	G	A
MEN0093G-P2	AC024580.1	snp	19	56662733	56662733	G	A
MEN0095-P	SLC25A31	snp	4	128685483	128685483	C	T
MEN0095-P	MMRN1	snp	4	90857872	90857872	T	G
MEN0095-P	MICU3	snp	8	16971612	16971612	T	A
MEN0095-P	OR5D18	snp	11	55587868	55587868	A	T
MEN0095-P	GRB14	snp	2	165476198	165476198	T	C
MEN0095-P	ADAMTS4	snp	1	161166610	161166610	C	T
MEN0095-P	MYO15A	snp	17	18047232	18047232	G	A
MEN0095-P	FAM47B	snp	X	34962036	34962036	G	A

MEN0095-P	PCDHGB3	snp	5	140750929	140750929	A	T
MEN0095-P	KIR3DX1	snp	19	55048103	55048103	C	A
MEN0095-P	DDAH1	snp	1	85816100	85816100	T	G
MEN0095-P	MICU3	snp	8	16971612	16971612	T	A
MEN0095-P	OR5D18	snp	11	55587868	55587868	A	T
MEN0095-P	DDAH1	snp	1	85816100	85816100	T	G
MEN0095-P	KIR3DX1	snp	19	55048103	55048103	C	A
MEN0095-P	ADAMTS4	snp	1	161166610	161166610	C	T
MEN0095-P	SLC25A31	snp	4	128685483	128685483	C	T
MEN0095-P	MYO15A	snp	17	18047232	18047232	G	A
MEN0095-P	MMRN1	snp	4	90857872	90857872	T	G
MEN0095-P	PCDHGB3	snp	5	140750929	140750929	A	T
MEN0095-P	GRB14	snp	2	165476198	165476198	T	C
MEN0095-P	FAM47B	snp	X	34962036	34962036	G	A
MEN0096-P	XPO7	snp	8	21851929	21851929	C	T
MEN0096-P	PRKCQ	snp	10	6470255	6470255	C	G
MEN0096-P	REV3L	snp	6	111737490	111737490	C	T
MEN0096-P	USP2	snp	11	119230007	119230007	T	A
MEN0096-P	SNX13	snp	7	17861172	17861172	T	C
MEN0096-P	P4HA3	snp	11	74013532	74013532	T	C
MEN0096-P	FLG2	snp	1	152323673	152323673	A	G
MEN0096-P	TRERF1	snp	6	42236685	42236685	C	T
MEN0096-P	TANC2	snp	17	61278216	61278216	A	T
MEN0096-P	KIAA0907	snp	1	155887393	155887393	T	G
MEN0096-P	MAGEC3	snp	X	140984779	140984779	C	A
MEN0096-P	MTA1	snp	14	105926724	105926724	A	G
MEN0096-P	OR5F1	snp	11	55761510	55761510	T	C
MEN0096-P	LDLRAD3	snp	11	36057774	36057774	C	A
MEN0096-P	P4HA3	snp	11	74013532	74013532	T	C
MEN0096-P	REV3L	snp	6	111737490	111737490	C	T
MEN0096-P	PRKCQ	snp	10	6470255	6470255	C	G
MEN0096-P	XPO7	snp	8	21851929	21851929	C	T
MEN0096-P	TANC2	snp	17	61278216	61278216	A	T
MEN0096-P	MTA1	snp	14	105926724	105926724	A	G
MEN0096-P	OR5F1	snp	11	55761510	55761510	T	C
MEN0096-P	USP2	snp	11	119230007	119230007	T	A
MEN0096-P	KIAA0907	snp	1	155887393	155887393	T	G
MEN0096-P	SNX13	snp	7	17861172	17861172	T	C
MEN0096-P	TRERF1	snp	6	42236685	42236685	C	T
MEN0096-P	FLG2	snp	1	152323673	152323673	A	G
MEN0096-P	MAGEC3	snp	X	140984779	140984779	C	A
MEN0096-P	LDLRAD3	snp	11	36057774	36057774	C	A
MEN0097-P	KIAA1407	snp	3	113724559	113724559	C	A
MEN0097-P	RP11-1407O15.2	snp	17	36357265	36357265	C	T
MEN0097-P	ZBTB25	snp	14	64954148	64954148	G	T
MEN0097-P	MED16	snp	19	876997	876997	G	A
MEN0097-P	CPNE5	snp	6	36730719	36730719	C	A

MEN0097-P	DSCAM	snp	21	42080449	42080449	T	G
MEN0097-P	KIF4A	snp	X	69510629	69510629	C	T
MEN0097-P	RC3H1	snp	1	173933296	173933296	G	A
MEN0097-P	KAT6B	snp	10	76780927	76780927	G	C
MEN0097-P	NFE2L3	snp	7	26225102	26225102	G	A
MEN0097-P	SLC17A2	snp	6	25923988	25923988	T	C
MEN0097-P	DCDC2	snp	6	24178791	24178791	C	T
MEN0097-P	SDK2	snp	17	71344826	71344826	G	C
MEN0097-P	SPRED1	snp	15	38643662	38643662	C	G
MEN0097-P	ROCK1	snp	18	18567057	18567057	G	C
MEN0097-P	RIMS1	snp	6	72968723	72968723	C	T
MEN0097-P	CSTF3	snp	11	33118462	33118462	T	C
MEN0097-P	AR	snp	X	66765161	66765161	A	T
MEN0097-P	ATRAID	snp	2	27439404	27439404	G	C
MEN0097-P	SPRED1	snp	15	38643248	38643248	C	G
MEN0097-P	CHRNB4	snp	15	78917370	78917370	A	G
MEN0097-P	ZC3H13	snp	13	46543999	46543999	G	T
MEN0097-P	GAGE2A	snp	X	49355893	49355893	C	G
MEN0097-P	MED16	snp	19	876997	876997	G	A
MEN0097-P	KIF4A	snp	X	69510629	69510629	C	T
MEN0097-P	KIAA1407	snp	3	113724559	113724559	C	A
MEN0097-P	KAT6B	snp	10	76780927	76780927	G	C
MEN0097-P	RC3H1	snp	1	173933296	173933296	G	A
MEN0097-P	CHRNB4	snp	15	78917370	78917370	A	G
MEN0097-P	ZBTB25	snp	14	64954148	64954148	G	T
MEN0097-P	AR	snp	X	66765161	66765161	A	T
MEN0097-P	SPRED1	snp	15	38643662	38643662	C	G
MEN0097-P	CPNE5	snp	6	36730719	36730719	C	A
MEN0097-P	DSCAM	snp	21	42080449	42080449	T	G
MEN0097-P	SDK2	snp	17	71344826	71344826	G	C
MEN0097-P	SPRED1	snp	15	38643248	38643248	C	G
MEN0097-P	RP11-1407O15.2	snp	17	36357265	36357265	C	T
MEN0097-P	CSTF3	snp	11	33118462	33118462	T	C
MEN0097-P	ROCK1	snp	18	18567057	18567057	G	C
MEN0097-P	SLC17A2	snp	6	25923988	25923988	T	C
MEN0097-P	DCDC2	snp	6	24178791	24178791	C	T
MEN0097-P	NFE2L3	snp	7	26225102	26225102	G	A
MEN0097-P	RIMS1	snp	6	72968723	72968723	C	T
MEN0097-P	GAGE2A	snp	X	49355893	49355893	C	G
MEN0097-P	ATRAID	snp	2	27439404	27439404	G	C
MEN0097-P	ZC3H13	snp	13	46543999	46543999	G	T
MEN0097-P1	DNAH2	snp	17	7660519	7660519	G	A
MEN0097-P1	MUC17	snp	7	100684076	100684076	A	G
MEN0097-P1	RPTN	snp	1	152127891	152127891	T	G
MEN0097-P1	ZNF254	snp	19	24310294	24310294	T	G
MEN0097-P1	RP11-1407O15.2	snp	17	36357265	36357265	C	T
MEN0097-P1	ZNF844	snp	19	12187394	12187394	T	C

MEN0097-P1	HEATR5B	snp	2	37215951	37215951	G	A
MEN0097-P1	ZNF721	snp	4	436628	436628	A	T
MEN0097-P1	GPR182	snp	12	57389222	57389222	G	A
MEN0097-P1	LGR4	snp	11	27393221	27393221	G	C
MEN0097-P1	MUC7	snp	4	71346978	71346978	T	C
MEN0097-P1	ZNF681	snp	19	23926943	23926943	C	A
MEN0097-P1	NPIPB5	snp	16	22545865	22545865	G	C
MEN0097-P1	TGOLN2	snp	2	85554530	85554530	T	G
MEN0097-P1	ING4	snp	12	6761832	6761832	G	A
MEN0097-P1	ZNF727	snp	7	63538806	63538806	C	G
MEN0097-P1	ZAN	snp	7	100350052	100350052	T	C
MEN0097-P1	ZNF649	snp	19	52394736	52394736	T	C
MEN0097-P1	MDC1	snp	6	30672941	30672941	T	C
MEN0097-P1	ZNF675	snp	19	23836390	23836390	T	C
MEN0097-P1	NBPF10	snp	1	145323695	145323695	C	T
MEN0097-P1	ZAN	snp	7	100349878	100349878	C	T
MEN0097-P1	SDK2	snp	17	71344826	71344826	G	C
MEN0097-P1	ZNF208	snp	19	22154342	22154342	A	C
MEN0097-P1	ROCK1	snp	18	18567057	18567057	G	C
MEN0097-P1	P2RX7	snp	12	121603964	121603964	G	A
MEN0097-P1	ATP6V1D	snp	14	67814150	67814150	C	T
MEN0097-P1	ZNF708	snp	19	21477037	21477037	A	C
MEN0097-P1	ZNF649	snp	19	52394769	52394769	A	C
MEN0097-P1	ATRAID	snp	2	27439404	27439404	G	C
MEN0097-P1	ZNF736	snp	7	63809113	63809113	A	G
MEN0097-P1	CHRNB4	snp	15	78917370	78917370	A	G
MEN0097-P1	ZNF479	snp	7	57187725	57187725	G	T
MEN0097-P1	ZNF736	snp	7	63809112	63809112	A	G
MEN0097-P1	TGOLN2	snp	2	85554535	85554535	C	T
MEN0097-P1	ZNF208	snp	19	22156983	22156983	T	A
MEN0097-P1	ZNF678	snp	1	227842669	227842669	A	G
MEN0097-P1	FAN1	snp	15	31197473	31197473	G	A
MEN0097-P1	ZNF43	snp	19	21990711	21990711	T	C
MEN0097-P1	ZNF708	snp	19	21476249	21476249	T	C
MEN0097-P1	ZNF844	snp	19	12187394	12187394	T	C
MEN0097-P1	ZNF681	snp	19	23926943	23926943	C	A
MEN0097-P1	HEATR5B	snp	2	37215951	37215951	G	A
MEN0097-P1	MUC17	snp	7	100684076	100684076	A	G
MEN0097-P1	ZNF721	snp	4	436628	436628	A	T
MEN0097-P1	ZNF254	snp	19	24310294	24310294	T	G
MEN0097-P1	DNAH2	snp	17	7660519	7660519	G	A
MEN0097-P1	ING4	snp	12	6761832	6761832	G	A
MEN0097-P1	GPR182	snp	12	57389222	57389222	G	A
MEN0097-P1	ZNF649	snp	19	52394736	52394736	T	C
MEN0097-P1	CHRNB4	snp	15	78917370	78917370	A	G
MEN0097-P1	P2RX7	snp	12	121603964	121603964	G	A
MEN0097-P1	ZNF708	snp	19	21477037	21477037	A	C

MEN0097-P1	ZNF727	snp	7	63538806	63538806	C	G
MEN0097-P1	NPIPB5	snp	16	22545865	22545865	G	C
MEN0097-P1	ATP6V1D	snp	14	67814150	67814150	C	T
MEN0097-P1	ZNF736	snp	7	63809112	63809112	A	G
MEN0097-P1	ZNF708	snp	19	21476249	21476249	T	C
MEN0097-P1	SDK2	snp	17	71344826	71344826	G	C
MEN0097-P1	ZNF678	snp	1	227842669	227842669	A	G
MEN0097-P1	ZNF43	snp	19	21990711	21990711	T	C
MEN0097-P1	RP11-1407O15.2	snp	17	36357265	36357265	C	T
MEN0097-P1	ROCK1	snp	18	18567057	18567057	G	C
MEN0097-P1	NBPF10	snp	1	145323695	145323695	C	T
MEN0097-P1	MDC1	snp	6	30672941	30672941	T	C
MEN0097-P1	LGR4	snp	11	27393221	27393221	G	C
MEN0097-P1	ZNF208	snp	19	22154342	22154342	A	C
MEN0097-P1	ZNF208	snp	19	22156983	22156983	T	A
MEN0097-P1	ZAN	snp	7	100349878	100349878	C	T
MEN0097-P1	FAN1	snp	15	31197473	31197473	G	A
MEN0097-P1	MUC7	snp	4	71346978	71346978	T	C
MEN0097-P1	TGOLN2	snp	2	85554530	85554530	T	G
MEN0097-P1	ZNF479	snp	7	57187725	57187725	G	T
MEN0097-P1	ATRAID	snp	2	27439404	27439404	G	C
MEN0097-P1	ZNF675	snp	19	23836390	23836390	T	C
MEN0097-P1	ZNF649	snp	19	52394769	52394769	A	C
MEN0097-P1	RPTN	snp	1	152127891	152127891	T	G
MEN0097-P1	ZAN	snp	7	100350052	100350052	T	C
MEN0097-P2	DISP1	snp	1	223176649	223176649	C	T
MEN0097-P2	DHX38	snp	16	72132559	72132559	C	T
MEN0097-P2	SLC26A11	snp	17	78215607	78215607	G	T
MEN0097-P2	PPP4R1	snp	18	9588723	9588723	C	A
MEN0097-P2	ZNF443	snp	19	12542283	12542283	A	G
MEN0097-P2	FAM73A	snp	1	78279422	78279422	T	C
MEN0097-P2	ZNF512B	snp	20	62597771	62597771	G	A
MEN0097-P2	AC011841.1	snp	GL000205.1	117670	117670	G	A
MEN0097-P2	CNTROB	snp	17	7838372	7838372	G	A
MEN0097-P2	ITGAE	snp	17	3665222	3665222	T	G
MEN0097-P2	PLA2G6	snp	22	38539198	38539198	A	C
MEN0097-P2	BHLHE40	snp	3	5022077	5022077	A	G
MEN0097-P2	NPIPB5	snp	16	22545865	22545865	G	C
MEN0097-P2	SYNE2	snp	14	64490997	64490997	G	T
MEN0097-P2	ITPA	snp	20	3199240	3199240	C	G
MEN0097-P2	TBR1	snp	2	162274270	162274270	C	T
MEN0097-P2	ARPP21	snp	3	35833943	35833943	G	T
MEN0097-P2	ACAN	snp	15	89398371	89398371	C	A
MEN0097-P2	SLC41A3	snp	3	125786889	125786889	G	C
MEN0097-P2	TRPM1	snp	15	31359366	31359366	G	A
MEN0097-P2	CSF2	snp	5	131411485	131411485	G	C
MEN0097-P2	MED13L	snp	12	116424160	116424160	C	T

MEN0097-P2	COL4A3	snp	2	228153956	228153956	G	A
MEN0097-P2	DHRS7	snp	14	60616232	60616232	G	A
MEN0097-P2	PTPRS	snp	19	5218496	5218496	G	A
MEN0097-P2	FCRL4	snp	1	157545388	157545388	C	T
MEN0097-P2	SDK2	snp	17	71344826	71344826	G	C
MEN0097-P2	ROCK1	snp	18	18567057	18567057	G	C
MEN0097-P2	PPP6R3	snp	11	68337333	68337333	G	C
MEN0097-P2	ANO3	snp	11	26681967	26681967	G	T
MEN0097-P2	MUC6	snp	11	1017526	1017526	G	C
MEN0097-P2	TCHH	snp	1	152082220	152082220	G	C
MEN0097-P2	NR1H4	snp	12	100926317	100926317	G	A
MEN0097-P2	ATRAID	snp	2	27439404	27439404	G	C
MEN0097-P2	TMEM31	snp	X	102967248	102967248	C	A
MEN0097-P2	CHRNB4	snp	15	78917370	78917370	A	G
MEN0097-P2	MUC21	snp	6	30954947	30954947	A	C
MEN0097-P2	SYNE2	snp	14	64490997	64490997	G	T
MEN0097-P2	SLC26A11	snp	17	78215607	78215607	G	T
MEN0097-P2	DHRS7	snp	14	60616232	60616232	G	A
MEN0097-P2	PPP4R1	snp	18	9588723	9588723	C	A
MEN0097-P2	COL4A3	snp	2	228153956	228153956	G	A
MEN0097-P2	BHLHE40	snp	3	5022077	5022077	A	G
MEN0097-P2	DISP1	snp	1	223176649	223176649	C	T
MEN0097-P2	FAM73A	snp	1	78279422	78279422	T	C
MEN0097-P2	ITGAE	snp	17	3665222	3665222	T	G
MEN0097-P2	ITPA	snp	20	3199240	3199240	C	G
MEN0097-P2	ARPP21	snp	3	35833943	35833943	G	T
MEN0097-P2	CNTROB	snp	17	7838372	7838372	G	A
MEN0097-P2	ZNF512B	snp	20	62597771	62597771	G	A
MEN0097-P2	CHRNB4	snp	15	78917370	78917370	A	G
MEN0097-P2	TBR1	snp	2	162274270	162274270	C	T
MEN0097-P2	AC011841.1	snp	GL000205.1	117670	117670	G	A
MEN0097-P2	SLC41A3	snp	3	125786889	125786889	G	C
MEN0097-P2	NPIPB5	snp	16	22545865	22545865	G	C
MEN0097-P2	SDK2	snp	17	71344826	71344826	G	C
MEN0097-P2	MED13L	snp	12	116424160	116424160	C	T
MEN0097-P2	ACAN	snp	15	89398371	89398371	C	A
MEN0097-P2	ZNF443	snp	19	12542283	12542283	A	G
MEN0097-P2	PTPRS	snp	19	5218496	5218496	G	A
MEN0097-P2	NR1H4	snp	12	100926317	100926317	G	A
MEN0097-P2	PLA2G6	snp	22	38539198	38539198	A	C
MEN0097-P2	FCRL4	snp	1	157545388	157545388	C	T
MEN0097-P2	CSF2	snp	5	131411485	131411485	G	C
MEN0097-P2	TRPM1	snp	15	31359366	31359366	G	A
MEN0097-P2	ROCK1	snp	18	18567057	18567057	G	C
MEN0097-P2	MUC21	snp	6	30954947	30954947	A	C
MEN0097-P2	MUC6	snp	11	1017526	1017526	G	C
MEN0097-P2	PPP6R3	snp	11	68337333	68337333	G	C

MEN0097-P2	ANO3	snp	11	26681967	26681967	G	T
MEN0097-P2	DHX38	snp	16	72132559	72132559	C	T
MEN0097-P2	TMEM31	snp	X	102967248	102967248	C	A
MEN0097-P2	ATRAID	snp	2	27439404	27439404	G	C
MEN0097-P2	TCHH	snp	1	152082220	152082220	G	C
MEN0097-P3	KLK8	snp	19	51504401	51504401	G	A
MEN0097-P3	GABRA2	snp	4	46252555	46252555	G	A
MEN0097-P3	DHX38	snp	16	72132559	72132559	C	T
MEN0097-P3	PPP4R1	snp	18	9588723	9588723	C	A
MEN0097-P3	PDE4DIP	snp	1	145075648	145075648	G	A
MEN0097-P3	UNC13D	snp	17	73832481	73832481	G	A
MEN0097-P3	TLK2	snp	17	60673986	60673986	C	T
MEN0097-P3	ITGAE	snp	17	3665222	3665222	T	G
MEN0097-P3	BHLHE40	snp	3	5022077	5022077	A	G
MEN0097-P3	SYNE2	snp	14	64490997	64490997	G	T
MEN0097-P3	ITPA	snp	20	3199240	3199240	C	G
MEN0097-P3	PLCB3	snp	11	64029447	64029447	G	A
MEN0097-P3	ARPP21	snp	3	35833943	35833943	G	T
MEN0097-P3	ACAN	snp	15	89398371	89398371	C	A
MEN0097-P3	SLC41A3	snp	3	125786889	125786889	G	C
MEN0097-P3	TRPM1	snp	15	31359366	31359366	G	A
MEN0097-P3	CSF2	snp	5	131411485	131411485	G	C
MEN0097-P3	MED13L	snp	12	116424160	116424160	C	T
MEN0097-P3	COL4A3	snp	2	228153956	228153956	G	A
MEN0097-P3	FAM47C	snp	X	37028425	37028425	A	G
MEN0097-P3	NACA	snp	12	57111554	57111554	C	T
MEN0097-P3	PRG4	snp	1	186276169	186276169	C	A
MEN0097-P3	COL15A1	snp	9	101816914	101816914	G	T
MEN0097-P3	FCRL4	snp	1	157545388	157545388	C	T
MEN0097-P3	SDK2	snp	17	71344826	71344826	G	C
MEN0097-P3	ZAN	snp	7	100350466	100350466	C	T
MEN0097-P3	ADD2	snp	2	70922923	70922923	C	T
MEN0097-P3	ROCK1	snp	18	18567057	18567057	G	C
MEN0097-P3	PPP6R3	snp	11	68337333	68337333	G	C
MEN0097-P3	TCHH	snp	1	152082484	152082484	G	T
MEN0097-P3	SLC27A6	snp	5	128362942	128362942	G	A
MEN0097-P3	NR1H4	snp	12	100926317	100926317	G	A
MEN0097-P3	ATRAID	snp	2	27439404	27439404	G	C
MEN0097-P3	TMEM31	snp	X	102967248	102967248	C	A
MEN0097-P3	CHRNB4	snp	15	78917370	78917370	A	G
MEN0097-P3	ZNF623	snp	8	144733541	144733541	C	A
MEN0097-P3	SYNE2	snp	14	64490997	64490997	G	T
MEN0097-P3	GABRA2	snp	4	46252555	46252555	G	A
MEN0097-P3	PPP4R1	snp	18	9588723	9588723	C	A
MEN0097-P3	COL4A3	snp	2	228153956	228153956	G	A
MEN0097-P3	BHLHE40	snp	3	5022077	5022077	A	G
MEN0097-P3	KLK8	snp	19	51504401	51504401	G	A

MEN0097-P3	ITGAE	snp	17	3665222	3665222	T	G
MEN0097-P3	ITPA	snp	20	3199240	3199240	C	G
MEN0097-P3	ADD2	snp	2	70922923	70922923	C	T
MEN0097-P3	SLC27A6	snp	5	128362942	128362942	G	A
MEN0097-P3	ARPP21	snp	3	35833943	35833943	G	T
MEN0097-P3	UNC13D	snp	17	73832481	73832481	G	A
MEN0097-P3	PDE4DIP	snp	1	145075648	145075648	G	A
MEN0097-P3	CHRNB4	snp	15	78917370	78917370	A	G
MEN0097-P3	SLC41A3	snp	3	125786889	125786889	G	C
MEN0097-P3	TLK2	snp	17	60673986	60673986	C	T
MEN0097-P3	SDK2	snp	17	71344826	71344826	G	C
MEN0097-P3	PLCB3	snp	11	64029447	64029447	G	A
MEN0097-P3	MED13L	snp	12	116424160	116424160	C	T
MEN0097-P3	COL15A1	snp	9	101816914	101816914	G	T
MEN0097-P3	ACAN	snp	15	89398371	89398371	C	A
MEN0097-P3	ZNF623	snp	8	144733541	144733541	C	A
MEN0097-P3	FAM47C	snp	X	37028425	37028425	A	G
MEN0097-P3	NR1H4	snp	12	100926317	100926317	G	A
MEN0097-P3	FCRL4	snp	1	157545388	157545388	C	T
MEN0097-P3	CSF2	snp	5	131411485	131411485	G	C
MEN0097-P3	TRPM1	snp	15	31359366	31359366	G	A
MEN0097-P3	ROCK1	snp	18	18567057	18567057	G	C
MEN0097-P3	PPP6R3	snp	11	68337333	68337333	G	C
MEN0097-P3	DHX38	snp	16	72132559	72132559	C	T
MEN0097-P3	ZAN	snp	7	100350466	100350466	C	T
MEN0097-P3	PRG4	snp	1	186276169	186276169	C	A
MEN0097-P3	TMEM31	snp	X	102967248	102967248	C	A
MEN0097-P3	ATRAID	snp	2	27439404	27439404	G	C
MEN0097-P3	TCHH	snp	1	152082484	152082484	G	T
MEN0097-P3	NACA	snp	12	57111554	57111554	C	T
MEN0098-P	SRPK2	snp	7	104782599	104782599	A	C
MEN0098-P	ANO8	snp	19	17441024	17441024	C	T
MEN0098-P	RANBP2	snp	2	109371685	109371685	A	T
MEN0098-P	ANAPC4	snp	4	25384970	25384970	C	T
MEN0098-P	RNF31	snp	14	24629560	24629560	C	T
MEN0098-P	TMEM207	snp	3	190167524	190167524	C	A
MEN0098-P	EYA1	snp	8	72127722	72127722	T	G
MEN0098-P	C14orf105	snp	14	57960259	57960259	C	A
MEN0098-P	CCDC80	snp	3	112357180	112357180	G	C
MEN0098-P	LEPREL2	snp	12	6939141	6939141	A	T
MEN0098-P	PLCH1	snp	3	155200438	155200438	T	A
MEN0098-P	KLHL41	snp	2	170377448	170377448	T	G
MEN0098-P	SERAC1	snp	6	158535990	158535990	T	G
MEN0098-P	EVA1A	snp	2	75745256	75745256	G	C
MEN0098-P	SLC35F4	snp	14	58036652	58036652	G	A
MEN0098-P	NRBF2	snp	10	64905988	64905988	G	C
MEN0098-P	MED12	snp	X	70357484	70357484	A	G

MEN0098-P	RFX3	snp	9	3293165	3293165	G	A
MEN0098-P	DICER1	snp	14	95579502	95579502	C	G
MEN0098-P	DZIP1	snp	13	96293573	96293573	G	C
MEN0098-P	KIAA0141	snp	5	141314074	141314074	G	A
MEN0098-P	NAALADL2	snp	3	175184770	175184770	G	C
MEN0098-P	MYCBP2	snp	13	77661726	77661726	G	A
MEN0098-P	PCDHGA2	snp	5	140719008	140719008	C	T
MEN0098-P	ZNF454	snp	5	178391777	178391777	C	G
MEN0098-P	TATDN2	snp	3	10312328	10312328	G	C
MEN0098-P	CHEK2	snp	22	29121242	29121242	G	A
MEN0098-P	PANK2	snp	20	3897664	3897664	T	G
MEN0098-P	PTPN1	snp	20	49196458	49196458	C	G
MEN0098-P	ZNF443	snp	19	12542006	12542006	T	C
MEN0098-P	ARHGDIA	snp	17	79826815	79826815	G	A
MEN0098-P	GTF2H2	snp	5	70338020	70338020	A	G
MEN0098-P	PRIM1	snp	12	57135531	57135531	T	C
MEN0098-P	RPH3AL	snp	17	69510	69510	C	A
MEN0098-P	RARA	snp	17	38508601	38508601	C	G
MEN0098-P	EXO1	snp	1	242015703	242015703	T	C
MEN0098-P	HSPH1	snp	13	31725120	31725120	A	C
MEN0098-P	FAM135B	snp	8	139278031	139278031	A	T
MEN0098-P	USP9X	snp	X	41029361	41029361	G	A
MEN0098-P	COPB2	snp	3	139085426	139085426	T	G
MEN0098-P	CAPN5	snp	11	76823801	76823801	G	A
MEN0098-P	BACH1	snp	21	30714858	30714858	G	C
MEN0098-P	MAN2A2	snp	15	91459428	91459428	A	T
MEN0098-P	VPS13A	snp	9	79934489	79934489	T	A
MEN0098-P	KIT	snp	4	55573422	55573422	T	C
MEN0098-P	ZNF607	snp	19	38190605	38190605	G	C
MEN0098-P	ADAMTS9	snp	3	64601713	64601713	G	T
MEN0098-P	KRTAP21-1	snp	21	32127683	32127683	T	C
MEN0098-P	EYA1	snp	8	72127722	72127722	T	G
MEN0098-P	PLCH1	snp	3	155200438	155200438	T	A
MEN0098-P	PLCH1	snp	3	155200438	155200438	T	A
MEN0098-P	PLCH1	snp	3	155200438	155200438	T	A
MEN0098-P	ANO8	snp	19	17441024	17441024	C	T
MEN0098-P	MAN2A2	snp	15	91459428	91459428	A	T
MEN0098-P	PANK2	snp	20	3897664	3897664	T	G
MEN0098-P	USP9X	snp	X	41029361	41029361	G	A
MEN0098-P	KIAA0141	snp	5	141314074	141314074	G	A
MEN0098-P	SERAC1	snp	6	158535990	158535990	T	G
MEN0098-P	MYCBP2	snp	13	77661726	77661726	G	A
MEN0098-P	BBS5	snp	2	170377448	170377448	T	G
MEN0098-P	FAM135B	snp	8	139278031	139278031	A	T
MEN0098-P	FAM135B	snp	8	139278031	139278031	A	T
MEN0098-P	FAM135B	snp	8	139278031	139278031	A	T
MEN0098-P	LEPREL2	snp	12	6939141	6939141	A	T

MEN0098-P	C14orf105	snp	14	57960259	57960259	C	A
MEN0098-P	PRIM1	snp	12	57135531	57135531	T	C
MEN0098-P	ZNF607	snp	19	38190605	38190605	G	C
MEN0098-P	BACH1	snp	21	30714858	30714858	G	C
MEN0098-P	DZIP1	snp	13	96293573	96293573	G	C
MEN0098-P	RFX3	snp	9	3293165	3293165	G	A
MEN0098-P	CHEK2	snp	22	29121242	29121242	G	A
MEN0098-P	VPS13A	snp	9	79934489	79934489	T	A
MEN0098-P	TMEM207	snp	3	190167524	190167524	C	A
MEN0098-P	ANAPC4	snp	4	25384970	25384970	C	T
MEN0098-P	GTF2H2	snp	5	70338020	70338020	A	G
MEN0098-P	NAALADL2	snp	3	175184770	175184770	G	C
MEN0098-P	SRPK2	snp	7	104782599	104782599	A	C
MEN0098-P	RANBP2	snp	2	109371685	109371685	A	T
MEN0098-P	SLC35F4	snp	14	58036652	58036652	G	A
MEN0098-P	CAPN5	snp	11	76823801	76823801	G	A
MEN0098-P	RPH3AL	snp	17	69510	69510	C	A
MEN0098-P	RNF31	snp	14	24629560	24629560	C	T
MEN0098-P	ZNF443	snp	19	12542006	12542006	T	C
MEN0098-P	RARA	snp	17	38508601	38508601	C	G
MEN0098-P	ARHGDIA	snp	17	79826815	79826815	G	A
MEN0098-P	ZNF454	snp	5	178391777	178391777	C	G
MEN0098-P	EVA1A	snp	2	75745256	75745256	G	C
MEN0098-P	MED12	snp	X	70357484	70357484	A	G
MEN0098-P	COPB2	snp	3	139085426	139085426	T	G
MEN0098-P	CCDC80	snp	3	112357180	112357180	G	C
MEN0098-P	PTPN1	snp	20	49196458	49196458	C	G
MEN0098-P	RP11-438J1.1	snp	3	10312328	10312328	G	C
MEN0098-P	PCDHGA2	snp	5	140719008	140719008	C	T
MEN0098-P	NRBF2	snp	10	64905988	64905988	G	C
MEN0098-P	DICER1	snp	14	95579502	95579502	C	G
MEN0098-P	ADAMTS9	snp	3	64601713	64601713	G	T
MEN0098-P	KIT	snp	4	55573422	55573422	T	C
MEN0098-P	HSPH1	snp	13	31725120	31725120	A	C
MEN0098-P	EXO1	snp	1	242015703	242015703	T	C
MEN0098-P	KRTAP21-1	snp	21	32127683	32127683	T	C
MEN0099-P	CEP68	snp	2	65299689	65299689	G	A
MEN0099-P	CRISP3	snp	6	49700981	49700981	C	T
MEN0099-P	TBRG4	snp	7	45143798	45143798	G	A
MEN0099-P	EP400NL	snp	12	132589753	132589753	G	T
MEN0099-P	FBXO42	snp	1	16578872	16578872	T	A
MEN0099-P	FLNB	snp	3	58124201	58124201	T	C
MEN0099-P	FRG1B	snp	20	29628243	29628243	T	C
MEN0099-P	BDP1	snp	5	70791213	70791213	G	T
MEN0099-P	TRIM51	snp	11	55658957	55658957	C	T
MEN0099-P	NWD1	snp	19	16874756	16874756	G	A
MEN0099-P	HNRNPK	snp	9	86589456	86589456	C	G

MEN0099-P	HSPA1L	snp	6	31778516	31778516	T	G
MEN0099-P	PTCHD4	snp	6	47846682	47846682	G	C
MEN0099-P	USP47	snp	11	11962008	11962008	A	G
MEN0099-P	FCER1A	snp	1	159277630	159277630	C	A
MEN0099-P	SIRPB1	snp	20	1585397	1585397	T	C
MEN0099-P	DPYSL5	snp	2	27164854	27164854	G	A
MEN0099-P	PRR5L	snp	11	36467889	36467889	T	C
MEN0099-P	KLK13	snp	19	51563084	51563084	T	C
MEN0099-P	CPNE7	snp	16	89645279	89645279	G	T
MEN0099-P	ATP2A3	snp	17	3844317	3844317	T	A
MEN0099-P	VASN	snp	16	4431578	4431578	G	T
MEN0099-P	TSPYL2	snp	X	53112391	53112391	C	G
MEN0099-P	PSD3	snp	8	18430064	18430064	G	C
MEN0099-P	GCC2	snp	2	109087980	109087980	A	T
MEN0099-P	NYX	snp	X	41333781	41333781	G	A
MEN0099-P	COL7A1	snp	3	48625813	48625813	C	T
MEN0099-P	ACAT1	snp	11	108005005	108005005	A	G
MEN0099-P	CBLB	snp	3	105400400	105400400	G	C
MEN0099-P	VASN	snp	16	4431578	4431578	G	T
MEN0099-P	TBRG4	snp	7	45143798	45143798	G	A
MEN0099-P	DPYSL5	snp	2	27164854	27164854	G	A
MEN0099-P	TSPYL2	snp	X	53112391	53112391	C	G
MEN0099-P	CEP68	snp	2	65299689	65299689	G	A
MEN0099-P	PSD3	snp	8	18430064	18430064	G	C
MEN0099-P	ATP2A3	snp	17	3844317	3844317	T	A
MEN0099-P	PRR5L	snp	11	36467889	36467889	T	C
MEN0099-P	PTCHD4	snp	6	47846682	47846682	G	C
MEN0099-P	FCER1A	snp	1	159277630	159277630	C	A
MEN0099-P	TRIM51	snp	11	55658957	55658957	C	T
MEN0099-P	USP47	snp	11	11962008	11962008	A	G
MEN0099-P	EP400NL	snp	12	132589753	132589753	G	T
MEN0099-P	FLNB	snp	3	58124201	58124201	T	C
MEN0099-P	GCC2	snp	2	109087980	109087980	A	T
MEN0099-P	CPNE7	snp	16	89645279	89645279	G	T
MEN0099-P	HSPA1L	snp	6	31778516	31778516	T	G
MEN0099-P	FBXO42	snp	1	16578872	16578872	T	A
MEN0099-P	HNRNPK	snp	9	86589456	86589456	C	G
MEN0099-P	FRG1B	snp	20	29628243	29628243	T	C
MEN0099-P	CRISP3	snp	6	49700981	49700981	C	T
MEN0099-P	COL7A1	snp	3	48625813	48625813	C	T
MEN0099-P	NWD1	snp	19	16874756	16874756	G	A
MEN0099-P	SIRPB1	snp	20	1585397	1585397	T	C
MEN0099-P	BDP1	snp	5	70791213	70791213	G	T
MEN0099-P	KLK13	snp	19	51563084	51563084	T	C
MEN0099-P	ACAT1	snp	11	108005005	108005005	A	G
MEN0099-P	NYX	snp	X	41333781	41333781	G	A
MEN0099-P	CBLB	snp	3	105400400	105400400	G	C

MEN0100-P	BARX2	snp	11	129306661	129306661	G	A
MEN0100-P	MGLL	snp	3	127441353	127441353	C	T
MEN0100-P	ATP2A2	snp	12	110783126	110783126	T	G
MEN0100-P	POTEC	snp	18	14513675	14513675	T	C
MEN0100-P	MCM2	snp	3	127327280	127327280	C	T
MEN0100-P	UBN2	snp	7	138969055	138969055	C	T
MEN0100-P	PHLDB2	snp	3	111603964	111603964	C	T
MEN0100-P	SON	snp	21	34924415	34924415	C	A
MEN0100-P	WDR90	snp	16	715992	715992	C	G
MEN0100-P	CAMTA1	snp	1	7723547	7723547	G	A
MEN0100-P	MGLL	snp	3	127441353	127441353	C	T
MEN0100-P	ATP2A2	snp	12	110783126	110783126	T	G
MEN0100-P	BARX2	snp	11	129306661	129306661	G	A
MEN0100-P	PHLDB2	snp	3	111603964	111603964	C	T
MEN0100-P	UBN2	snp	7	138969055	138969055	C	T
MEN0100-P	MCM2	snp	3	127327280	127327280	C	T
MEN0100-P	SON	snp	21	34924415	34924415	C	A
MEN0100-P	WDR90	snp	16	715992	715992	C	G
MEN0100-P	POTEC	snp	18	14513675	14513675	T	C
MEN0100-P	CAMTA1	snp	1	7723547	7723547	G	A
MEN0101-P0	NINL	snp	20	25460868	25460868	T	A
MEN0101-P0	VOPP1	snp	7	55560037	55560037	G	A
MEN0101-P0	ZNF419	snp	19	58005146	58005146	C	G
MEN0101-P0	SCN9A	snp	2	167133716	167133716	C	T
MEN0101-P0	KIFC3	snp	16	57805243	57805243	C	T
MEN0101-P0	MST1R	snp	3	49941029	49941029	G	A
MEN0101-P0	SCARB1	snp	12	125298833	125298833	C	T
MEN0101-P0	SLAMF6	snp	1	160466156	160466156	G	A
MEN0101-P0	TRAPPC8	snp	18	29437582	29437582	G	A
MEN0101-P0	SLC9A3	snp	5	482812	482812	G	A
MEN0101-P0	TTC14	snp	3	180327775	180327775	A	G
MEN0101-P0	LGI3	snp	8	22011505	22011505	C	T
MEN0101-P0	NECAB2	snp	16	84012099	84012099	G	A
MEN0101-P0	ZNF678	snp	1	227842427	227842427	G	A
MEN0101-P0	ZNF91	snp	19	23544867	23544867	T	C
MEN0101-P0	GEMIN4	snp	17	650244	650244	G	A
MEN0101-P0	CD209	snp	19	7810766	7810766	C	T
MEN0101-P0	PGM2L1	snp	11	74057793	74057793	C	T
MEN0101-P0	RHOT1	snp	17	30535250	30535250	C	T
MEN0101-P0	TBC1D3F	snp	17	36288207	36288207	C	T
MEN0101-P0	KCNB1	snp	20	47990392	47990392	C	T
MEN0101-P0	TFG	snp	3	100447576	100447576	C	T
MEN0101-P0	SNX13	snp	7	17838675	17838675	G	T
MEN0101-P0	NBPF14	snp	1	148012533	148012533	T	G
MEN0101-P0	ZNF708	snp	19	21476898	21476898	T	G
MEN0101-P0	ZNF844	snp	19	12187275	12187275	G	C
MEN0101-P0	ZNF208	snp	19	22155896	22155896	A	C

MEN0101-P0	ZNF737	snp	19	20728254	20728254	C	G
MEN0101-P0	ZNF708	snp	19	21476519	21476519	T	C
MEN0101-P0	CAPRIN2	snp	12	30881677	30881677	A	T
MEN0101-P0	TLE1	snp	9	84249141	84249141	G	T
MEN0101-P0	NAF1	snp	4	164066964	164066964	A	C
MEN0101-P0	MOV10	snp	1	113232135	113232135	G	A
MEN0101-P0	ZNF433	snp	19	12125945	12125945	C	A
MEN0101-P0	CDH9	snp	5	26889966	26889966	A	C
MEN0101-P0	ZNF99	snp	19	22940775	22940775	C	T
MEN0101-P0	ZNF676	snp	19	22363039	22363039	T	C
MEN0101-P0	ZNF749	snp	19	57955885	57955885	C	G
MEN0101-P0	CEP85L	snp	6	118801571	118801571	C	A
MEN0101-P0	CAPRIN2	snp	12	30881678	30881678	C	G
MEN0101-P0	ZNF708	snp	19	21476249	21476249	T	C
MEN0101-P0	ATP6AP2	snp	X	40450514	40450514	G	A
MEN0101-P0	SPRY1	snp	4	124323120	124323120	G	A
MEN0101-P0	CGREF1	snp	2	27324405	27324405	T	C
MEN0101-P0	KRTAP1-3	snp	17	39190779	39190779	T	C
MEN0101-P0	KCNH8	snp	3	19190281	19190281	G	A
MEN0101-P0	ZNF730	snp	19	23328831	23328831	A	G
MEN0101-P0	TGOLN2	snp	2	85554367	85554367	T	C
MEN0101-P0	KRTAP4-11	snp	17	39274416	39274416	C	T
MEN0101-P0	GTF2F1	snp	19	6381016	6381016	G	A
MEN0101-P0	C2orf16	snp	2	27804477	27804477	G	A
MEN0101-P0	VOPP1	snp	7	55560037	55560037	G	A
MEN0101-P0	ZNF737	snp	19	20728254	20728254	C	G
MEN0101-P0	SNX13	snp	7	17838675	17838675	G	T
MEN0101-P0	MOV10	snp	1	113232135	113232135	G	A
MEN0101-P0	SLC9A3	snp	5	482812	482812	G	A
MEN0101-P0	ZNF749	snp	19	57955885	57955885	C	G
MEN0101-P0	TFG	snp	3	100447576	100447576	C	T
MEN0101-P0	KRTAP1-3	snp	17	39190779	39190779	T	C
MEN0101-P0	SPRY1	snp	4	124323120	124323120	G	A
MEN0101-P0	NINL	snp	20	25460868	25460868	T	A
MEN0101-P0	ATP6AP2	snp	X	40450514	40450514	G	A
MEN0101-P0	ZNF676	snp	19	22363039	22363039	T	C
MEN0101-P0	ZNF208	snp	19	22155896	22155896	A	C
MEN0101-P0	ZNF844	snp	19	12187275	12187275	G	C
MEN0101-P0	KCNH8	snp	3	19190281	19190281	G	A
MEN0101-P0	CD209	snp	19	7810766	7810766	C	T
MEN0101-P0	ZNF91	snp	19	23544867	23544867	T	C
MEN0101-P0	TBC1D3F	snp	17	36288207	36288207	C	T
MEN0101-P0	ZNF99	snp	19	22940775	22940775	C	T
MEN0101-P0	SCARB1	snp	12	125298833	125298833	C	T
MEN0101-P0	CAPRIN2	snp	12	30881677	30881677	A	T
MEN0101-P0	MST1R	snp	3	49941029	49941029	G	A
MEN0101-P0	SLAMF6	snp	1	160466156	160466156	G	A

MEN0101-P0	ZNF433	snp	19	12125945	12125945	C	A
MEN0101-P0	ZNF419	snp	19	58005146	58005146	C	G
MEN0101-P0	SCN9A	snp	2	167133716	167133716	C	T
MEN0101-P0	NAF1	snp	4	164066964	164066964	A	C
MEN0101-P0	ZNF708	snp	19	21476519	21476519	T	C
MEN0101-P0	TTC14	snp	3	180327775	180327775	A	G
MEN0101-P0	NECAB2	snp	16	84012099	84012099	G	A
MEN0101-P0	RHOT1	snp	17	30535250	30535250	C	T
MEN0101-P0	ZNF708	snp	19	21476249	21476249	T	C
MEN0101-P0	ZNF678	snp	1	227842427	227842427	G	A
MEN0101-P0	CDH9	snp	5	26889966	26889966	A	C
MEN0101-P0	NBPF14	snp	1	148012533	148012533	T	G
MEN0101-P0	TRAPPC8	snp	18	29437582	29437582	G	A
MEN0101-P0	ZNF730	snp	19	23328831	23328831	A	G
MEN0101-P0	TGOLN2	snp	2	85554367	85554367	T	C
MEN0101-P0	TLE1	snp	9	84249141	84249141	G	T
MEN0101-P0	KCNB1	snp	20	47990392	47990392	C	T
MEN0101-P0	ZNF708	snp	19	21476898	21476898	T	G
MEN0101-P0	LGI3	snp	8	22011505	22011505	C	T
MEN0101-P0	GEMIN4	snp	17	650244	650244	G	A
MEN0101-P0	GTF2F1	snp	19	6381016	6381016	G	A
MEN0101-P0	CGREF1	snp	2	27324405	27324405	T	C
MEN0101-P0	KIFC3	snp	16	57805243	57805243	C	T
MEN0101-P0	PGM2L1	snp	11	74057793	74057793	C	T
MEN0101-P0	CEP85L	snp	6	118801571	118801571	C	A
MEN0101-P0	C2orf16	snp	2	27804477	27804477	G	A
MEN0101-P0	KRTAP4-11	snp	17	39274416	39274416	C	T
MEN0101-P1	ALG10	snp	12	34179619	34179619	A	T
MEN0101-P1	SVEP1	snp	9	113170636	113170636	C	A
MEN0101-P1	VOPP1	snp	7	55560037	55560037	G	A
MEN0101-P1	OR52E6	snp	11	5863012	5863012	A	G
MEN0101-P1	TTC37	snp	5	94856484	94856484	G	C
MEN0101-P1	ZNF831	snp	20	57767937	57767937	G	C
MEN0101-P1	DONSON	snp	21	34955892	34955892	G	C
MEN0101-P1	GRIA1	snp	5	153085383	153085383	G	T
MEN0101-P1	NLRP10	snp	11	7981447	7981447	T	G
MEN0101-P1	RNF135	snp	17	29326126	29326126	G	C
MEN0101-P1	ARID4B	snp	1	235384900	235384900	A	T
MEN0101-P1	USH1C	snp	11	17523521	17523521	G	A
MEN0101-P1	LGI3	snp	8	22011505	22011505	C	T
MEN0101-P1	ADAM22	snp	7	87810838	87810838	T	A
MEN0101-P1	ZNF687	snp	1	151259363	151259363	T	A
MEN0101-P1	ABCA8	snp	17	66898860	66898860	T	C
MEN0101-P1	TFG	snp	3	100447576	100447576	C	T
MEN0101-P1	ZNF442	snp	19	12461974	12461974	G	A
MEN0101-P1	HELZ2	snp	20	62195703	62195703	T	C
MEN0101-P1	GNG2	snp	14	52433364	52433364	A	G

MEN0101-P1	ENTPD8	snp	9	140330478	140330478	C	T
MEN0101-P1	LRBA	snp	4	151207126	151207126	G	A
MEN0101-P1	UBE2S	snp	19	55918233	55918233	A	G
MEN0101-P1	ZNF442	snp	19	12461977	12461977	T	A
MEN0101-P1	SCN10A	snp	3	38743475	38743475	C	G
MEN0101-P1	MMP28	snp	17	34097303	34097303	G	C
MEN0101-P1	MAGED1	snp	X	51639619	51639619	G	T
MEN0101-P1	EGLN2	snp	19	41306722	41306722	G	A
MEN0101-P1	PIK3C2B	snp	1	204435934	204435934	G	A
MEN0101-P1	STXBP5L	snp	3	121134750	121134750	A	G
MEN0101-P1	SDCBP	snp	8	59490691	59490691	G	A
MEN0101-P1	SYVN1	snp	11	64896082	64896082	G	C
MEN0101-P1	ZNF814	snp	19	58385546	58385546	G	T
MEN0101-P1	KRTAP4-11	snp	17	39274416	39274416	C	T
MEN0101-P1	NEK6	snp	9	127074797	127074797	A	G
MEN0101-P1	SCN9A	snp	2	167168161	167168161	T	G
MEN0101-P1	PDIA4	snp	7	148718171	148718171	C	T
MEN0101-P1	KRTAP4-11	snp	17	39274214	39274214	G	C
MEN0101-P1	HELZ2	snp	20	62195703	62195703	T	C
MEN0101-P1	VOPP1	snp	7	55560037	55560037	G	A
MEN0101-P1	USH1C	snp	11	17523521	17523521	G	A
MEN0101-P1	DONSON	snp	21	34955892	34955892	G	C
MEN0101-P1	TFG	snp	3	100447576	100447576	C	T
MEN0101-P1	GRIA1	snp	5	153085383	153085383	G	T
MEN0101-P1	TTC37	snp	5	94856484	94856484	G	C
MEN0101-P1	ARID4B	snp	1	235384900	235384900	A	T
MEN0101-P1	OR52E6	snp	11	5863012	5863012	A	G
MEN0101-P1	ZNF831	snp	20	57767937	57767937	G	C
MEN0101-P1	NLRP10	snp	11	7981447	7981447	T	G
MEN0101-P1	SCN10A	snp	3	38743475	38743475	C	G
MEN0101-P1	ADAM22	snp	7	87810838	87810838	T	A
MEN0101-P1	SYVN1	snp	11	64896082	64896082	G	C
MEN0101-P1	SVEP1	snp	9	113170636	113170636	C	A
MEN0101-P1	ALG10	snp	12	34179619	34179619	A	T
MEN0101-P1	ZNF442	snp	19	12461974	12461974	G	A
MEN0101-P1	PIK3C2B	snp	1	204435934	204435934	G	A
MEN0101-P1	ZNF814	snp	19	58385546	58385546	G	T
MEN0101-P1	ENTPD8	snp	9	140330478	140330478	C	T
MEN0101-P1	MAGED1	snp	X	51639619	51639619	G	T
MEN0101-P1	MMP28	snp	17	34097303	34097303	G	C
MEN0101-P1	ABCA8	snp	17	66898860	66898860	T	C
MEN0101-P1	LGI3	snp	8	22011505	22011505	C	T
MEN0101-P1	STXBP5L	snp	3	121134750	121134750	A	G
MEN0101-P1	SCN9A	snp	2	167168161	167168161	T	G
MEN0101-P1	LRBA	snp	4	151207126	151207126	G	A
MEN0101-P1	NEK6	snp	9	127074797	127074797	A	G
MEN0101-P1	RNF135	snp	17	29326126	29326126	G	C

MEN0101-P1	SDCBP	snp	8	59490691	59490691	G	A
MEN0101-P1	GNG2	snp	14	52433364	52433364	A	G
MEN0101-P1	EGLN2	snp	19	41306722	41306722	G	A
MEN0101-P1	ZNF687	snp	1	151259363	151259363	T	A
MEN0101-P1	KRTAP4-11	snp	17	39274214	39274214	G	C
MEN0101-P1	UBE2S	snp	19	55918233	55918233	A	G
MEN0101-P1	KRTAP4-11	snp	17	39274416	39274416	C	T
MEN0101-P1	PDIA4	snp	7	148718171	148718171	C	T
MEN0102-P	ZAN	snp	7	100365542	100365542	C	A
MEN0102-P	TBC1D31	snp	8	124132401	124132401	T	A
MEN0102-P	CHEK2	snp	22	29091840	29091840	T	C
MEN0102-P	CDK20	snp	9	90584208	90584208	C	T
MEN0102-P	NOTCH2	snp	1	120469147	120469147	T	C
MEN0102-P	NOTCH2NL	snp	1	145281473	145281473	T	G
MEN0102-P	BRCA2	snp	13	32911929	32911929	A	G
MEN0102-P	MT-ATP6	snp	MT	8902	8902	G	A
MEN0102-P	TBC1D31	snp	8	124132401	124132401	T	A
MEN0102-P	ZAN	snp	7	100365542	100365542	C	A
MEN0102-P	CHEK2	snp	22	29091840	29091840	T	C
MEN0102-P	MT-ATP6	snp	MT	8902	8902	G	A
MEN0102-P	NOTCH2	snp	1	120469147	120469147	T	C
MEN0102-P	CDK20	snp	9	90584208	90584208	C	T
MEN0102-P	BRCA2	snp	13	32911929	32911929	A	G
MEN0102-P	NOTCH2NL	snp	1	145281473	145281473	T	G
MEN0103-P	SGMS1	snp	10	52103409	52103409	C	T
MEN0103-P	ING3	snp	7	120604810	120604810	G	C
MEN0103-P	CCDC18	snp	1	93676420	93676420	C	T
MEN0103-P	ZNF93	snp	19	20044827	20044827	A	G
MEN0103-P	SETD5	snp	3	9486866	9486866	G	A
MEN0103-P	TBX5	snp	12	114832692	114832692	G	T
MEN0103-P	LPCAT1	snp	5	1501646	1501646	G	A
MEN0103-P	ACCSL	snp	11	44069708	44069708	C	T
MEN0103-P	AC011841.1	snp	GL000205.1	117444	117444	G	T
MEN0103-P	PHYHIPL	snp	10	60936719	60936719	G	C
MEN0103-P	MYH6	snp	14	23853800	23853800	C	T
MEN0103-P	UACA	snp	15	70959718	70959718	G	C
MEN0103-P	PNP	snp	14	20944723	20944723	T	C
MEN0103-P	ECI2	snp	6	4116219	4116219	T	G
MEN0103-P	PMFBP1	snp	16	72154019	72154019	T	C
MEN0103-P	TAOK1	snp	17	27805302	27805302	C	A
MEN0103-P	TAOK1	snp	17	27805301	27805301	G	T
MEN0103-P	FCHSD2	snp	11	72549864	72549864	A	G
MEN0103-P	PHAX	snp	5	125939818	125939818	G	A
MEN0103-P	FAM47C	snp	X	37028425	37028425	A	G
MEN0103-P	CASP10	snp	2	202050508	202050508	C	G
MEN0103-P	ZNF93	snp	19	20044827	20044827	A	G
MEN0103-P	PNP	snp	14	20944723	20944723	T	C

MEN0103-P	ACCSL	snp	11	44069708	44069708	C	T
MEN0103-P	LPCAT1	snp	5	1501646	1501646	G	A
MEN0103-P	AC011841.1	snp	GL000205.1	117444	117444	G	T
MEN0103-P	CCDC18	snp	1	93676420	93676420	C	T
MEN0103-P	TAOK1	snp	17	27805301	27805301	G	T
MEN0103-P	SGMS1	snp	10	52103409	52103409	C	T
MEN0103-P	ING3	snp	7	120604810	120604810	G	C
MEN0103-P	ECI2	snp	6	4116219	4116219	T	G
MEN0103-P	TBX5	snp	12	114832692	114832692	G	T
MEN0103-P	PHYHIPL	snp	10	60936719	60936719	G	C
MEN0103-P	SETD5	snp	3	9486866	9486866	G	A
MEN0103-P	PMFBP1	snp	16	72154019	72154019	T	C
MEN0103-P	UACA	snp	15	70959718	70959718	G	C
MEN0103-P	PHAX	snp	5	125939818	125939818	G	A
MEN0103-P	MYH6	snp	14	23853800	23853800	C	T
MEN0103-P	FAM47C	snp	X	37028425	37028425	A	G
MEN0103-P	FCHSD2	snp	11	72549864	72549864	A	G
MEN0103-P	CASP10	snp	2	202050508	202050508	C	G
MEN0104-P	PLCZ1	snp	12	18836196	18836196	C	A
MEN0104-P	BMP3	snp	4	81974620	81974620	T	C
MEN0104-P	RTTN	snp	18	67833342	67833342	G	C
MEN0104-P	GPR126	snp	6	142741040	142741040	T	A
MEN0104-P	KDR	snp	4	55972049	55972049	G	A
MEN0104-P	GIGYF2	snp	2	233675964	233675964	G	T
MEN0104-P	TAGLN2	snp	1	159890322	159890322	G	A
MEN0104-P	SYNPO	snp	5	150028001	150028001	C	T
MEN0104-P	PDE4DIP	snp	1	144859983	144859983	G	A
MEN0104-P	ACER3	snp	11	76687335	76687335	A	G
MEN0104-P	NRXN3	snp	14	79433664	79433664	G	T
MEN0104-P	GDPD1	snp	17	57326146	57326146	G	A
MEN0104-P	IMPG1	snp	6	76734934	76734934	C	A
MEN0104-P	MED12L	snp	3	151082880	151082880	G	A
MEN0104-P	ORC1	snp	1	52849153	52849153	G	A
MEN0104-P	CLUH	snp	17	2603739	2603739	C	A
MEN0104-P	GRIA4	snp	11	105795185	105795185	T	A
MEN0104-P	KCNH4	snp	17	40328236	40328236	T	C
MEN0104-P	TYRO3	snp	15	41853375	41853375	C	T
MEN0104-P	TTN	snp	2	179614561	179614561	A	T
MEN0104-P	PRDM2	snp	1	14075965	14075965	C	A
MEN0104-P	ACE	snp	17	61561894	61561894	A	G
MEN0104-P	NLRP11	snp	19	56307519	56307519	G	A
MEN0104-P	CIT	snp	12	120150416	120150416	A	T
MEN0104-P	PLEKHN1	snp	1	906348	906348	C	G
MEN0104-P	PRAMEF2	snp	1	12919972	12919972	G	T
MEN0104-P	CHEK2	snp	22	29091840	29091840	T	C
MEN0104-P	PRSS48	snp	4	152203538	152203538	G	A
MEN0104-P	GRAP	snp	17	18927643	18927643	T	C

MEN0104-P	EIF4E1B	snp	5	176070195	176070195	T	C
MEN0104-P	RTTN	snp	18	67833342	67833342	G	C
MEN0104-P	KCNH4	snp	17	40328236	40328236	T	C
MEN0104-P	TAGLN2	snp	1	159890322	159890322	G	A
MEN0104-P	NLRP11	snp	19	56307519	56307519	G	A
MEN0104-P	ORC1	snp	1	52849153	52849153	G	A
MEN0104-P	GRIA4	snp	11	105795185	105795185	T	A
MEN0104-P	NRXN3	snp	14	79433664	79433664	G	T
MEN0104-P	CIT	snp	12	120150416	120150416	A	T
MEN0104-P	MED12L	snp	3	151082880	151082880	G	A
MEN0104-P	ACER3	snp	11	76687335	76687335	A	G
MEN0104-P	KDR	snp	4	55972049	55972049	G	A
MEN0104-P	TYRO3	snp	15	41853375	41853375	C	T
MEN0104-P	GRAP	snp	17	18927643	18927643	T	C
MEN0104-P	IMPG1	snp	6	76734934	76734934	C	A
MEN0104-P	GIGYF2	snp	2	233675964	233675964	G	T
MEN0104-P	GDPD1	snp	17	57326146	57326146	G	A
MEN0104-P	PRAMEF2	snp	1	12919972	12919972	G	T
MEN0104-P	GPR126	snp	6	142741040	142741040	T	A
MEN0104-P	CHEK2	snp	22	29091840	29091840	T	C
MEN0104-P	BMP3	snp	4	81974620	81974620	T	C
MEN0104-P	PLCZ1	snp	12	18836196	18836196	C	A
MEN0104-P	PDE4DIP	snp	1	144859983	144859983	G	A
MEN0104-P	ACE	snp	17	61561894	61561894	A	G
MEN0104-P	TTN	snp	2	179614561	179614561	A	T
MEN0104-P	PRSS48	snp	4	152203538	152203538	G	A
MEN0104-P	CLUH	snp	17	2603739	2603739	C	A
MEN0104-P	SYNPO	snp	5	150028001	150028001	C	T
MEN0104-P	PLEKHN1	snp	1	906348	906348	C	G
MEN0104-P	PRDM2	snp	1	14075965	14075965	C	A
MEN0104-P	EIF4E1B	snp	5	176070195	176070195	T	C
MEN0105-P	CACNA1F	snp	X	49068717	49068717	C	T
MEN0105-P	OR8D2	snp	11	124190032	124190032	G	C
MEN0105-P	PAF1	snp	19	39879048	39879048	C	A
MEN0105-P	RAD51AP2	snp	2	17697824	17697824	T	G
MEN0105-P	PTPN13	snp	4	87643406	87643406	A	T
MEN0105-P	METTL13	snp	1	171761249	171761249	G	A
MEN0105-P	UBE3C	snp	7	156974807	156974807	A	T
MEN0105-P	POLE	snp	12	133254220	133254220	G	A
MEN0105-P	PCSK7	snp	11	117094902	117094902	G	A
MEN0105-P	ZC4H2	snp	X	64140091	64140091	G	C
MEN0105-P	TMCC2	snp	1	205238703	205238703	C	T
MEN0105-P	CUX2	snp	12	111758019	111758019	G	A
MEN0105-P	MYBPH	snp	1	203141093	203141093	T	C
MEN0105-P	NUP214	snp	9	134021562	134021562	G	A
MEN0105-P	POLR2B	snp	4	57887119	57887119	C	G
MEN0105-P	IL1RAPL1	snp	X	29301057	29301057	G	A

MEN0105-P	SECISBP2	snp	9	91940861	91940861	G	C
MEN0105-P	CMBL	snp	5	10290861	10290861	G	T
MEN0105-P	RAD51AP2	snp	2	17697824	17697824	T	G
MEN0105-P	TMCC2	snp	1	205238703	205238703	C	T
MEN0105-P	CACNA1F	snp	X	49068717	49068717	C	T
MEN0105-P	MYBPH	snp	1	203141093	203141093	T	C
MEN0105-P	CUX2	snp	12	111758019	111758019	G	A
MEN0105-P	PCSK7	snp	11	117094902	117094902	G	A
MEN0105-P	POLE	snp	12	133254220	133254220	G	A
MEN0105-P	NUP214	snp	9	134021562	134021562	G	A
MEN0105-P	PAF1	snp	19	39879048	39879048	C	A
MEN0105-P	OR8D2	snp	11	124190032	124190032	G	C
MEN0105-P	POLR2B	snp	4	57887119	57887119	C	G
MEN0105-P	UBE3C	snp	7	156974807	156974807	A	T
MEN0105-P	PTPN13	snp	4	87643406	87643406	A	T
MEN0105-P	METTL13	snp	1	171761249	171761249	G	A
MEN0105-P	ZC4H2	snp	X	64140091	64140091	G	C
MEN0105-P	SECISBP2	snp	9	91940861	91940861	G	C
MEN0105-P	IL1RAPL1	snp	X	29301057	29301057	G	A
MEN0105-P	CMBL	snp	5	10290861	10290861	G	T
MEN0106-P	KCNV1	snp	8	110980814	110980814	C	A
MEN0106-P	PRG4	snp	1	186276481	186276481	T	C
MEN0106-P	IGKV1D-16	snp	2	90139477	90139477	G	A
MEN0106-P	HSP90AB1	snp	6	44220861	44220861	G	A
MEN0106-P	MUC17	snp	7	100684086	100684086	T	C
MEN0106-P	C6	snp	5	41155074	41155074	G	A
MEN0106-P	CLCA2	snp	1	86913234	86913234	T	G
MEN0106-P	PRAMEF2	snp	1	12919840	12919840	T	C
MEN0106-P	EPB41L2	snp	6	131188709	131188709	T	C
MEN0106-P	PRAMEF11	snp	1	12887453	12887453	C	T
MEN0106-P	GGTLC2	snp	22	22989241	22989241	G	A
MEN0106-P	FAM83B	snp	6	54805523	54805523	C	T
MEN0106-P	IGF2BP3	snp	7	23352422	23352422	G	A
MEN0106-P	CDC5L	snp	6	44414384	44414384	A	G
MEN0106-P	PRMT8	snp	12	3649787	3649787	T	C
MEN0106-P	ADAD2	snp	16	84224883	84224883	A	G
MEN0106-P	KRTAP5-2	snp	11	1619378	1619378	G	A
MEN0106-P	PRAMEF2	snp	1	12919840	12919840	T	C
MEN0106-P	EPB41L2	snp	6	131188709	131188709	T	C
MEN0106-P	PRAMEF11	snp	1	12887453	12887453	C	T
MEN0106-P	IGF2BP3	snp	7	23352422	23352422	G	A
MEN0106-P	IGKV1D-16	snp	2	90139477	90139477	G	A
MEN0106-P	CDC5L	snp	6	44414384	44414384	A	G
MEN0106-P	GGTLC2	snp	22	22989241	22989241	G	A
MEN0106-P	ADAD2	snp	16	84224883	84224883	A	G
MEN0106-P	HSP90AB1	snp	6	44220861	44220861	G	A
MEN0106-P	KCNV1	snp	8	110980814	110980814	C	A

MEN0106-P	MUC17	snp	7	100684086	100684086	T	C
MEN0106-P	FAM83B	snp	6	54805523	54805523	C	T
MEN0106-P	CLCA2	snp	1	86913234	86913234	T	G
MEN0106-P	C6	snp	5	41155074	41155074	G	A
MEN0106-P	PRMT8	snp	12	3649787	3649787	T	C
MEN0106-P	PRG4	snp	1	186276481	186276481	T	C
MEN0106-P	KRTAP5-2	snp	11	1619378	1619378	G	A
MEN0107-P	AQP7	snp	9	33385614	33385614	A	T
MEN0107-P	DENND4B	snp	1	153908604	153908604	G	A
MEN0107-P	SLCO1C1	snp	12	20868190	20868190	C	T
MEN0107-P	NBPF10	snp	1	145311916	145311916	A	G
MEN0107-P	NACA	snp	12	57112087	57112087	A	G
MEN0107-P	POM121	snp	7	72398976	72398976	A	G
MEN0107-P	DFNA5	snp	7	24745946	24745946	T	C
MEN0107-P	AC109135.1	snp	GL000192.1	311344	311344	C	T
MEN0107-P	PCSK5	snp	9	78790192	78790192	C	G
MEN0107-P	PRAMEF11	snp	1	12887453	12887453	C	T
MEN0107-P	BHLHB9	snp	X	102004492	102004492	C	G
MEN0107-P	AL592183.1	snp	GL000219.1	83216	83216	G	C
MEN0107-P	IWS1	snp	2	128262691	128262691	G	A
MEN0107-P	POM121	snp	7	72398976	72398976	A	G
MEN0107-P	DFNA5	snp	7	24745946	24745946	T	C
MEN0107-P	NACA	snp	12	57112087	57112087	A	G
MEN0107-P	DENND4B	snp	1	153908604	153908604	G	A
MEN0107-P	SLCO1C1	snp	12	20868190	20868190	C	T
MEN0107-P	PRAMEF11	snp	1	12887453	12887453	C	T
MEN0107-P	NBPF10	snp	1	145311916	145311916	A	G
MEN0107-P	AC109135.1	snp	GL000192.1	311344	311344	C	T
MEN0107-P	PCSK5	snp	9	78790192	78790192	C	G
MEN0107-P	AQP7	snp	9	33385614	33385614	A	T
MEN0107-P	BHLHB9	snp	X	102004492	102004492	C	G
MEN0107-P	IWS1	snp	2	128262691	128262691	G	A
MEN0107-P	AL592183.1	snp	GL000219.1	83216	83216	G	C
MEN0108-P	TMPRSS6	snp	22	37494473	37494473	C	T
MEN0108-P	MMP19	snp	12	56230872	56230872	C	T
MEN0108-P	KMT2C	snp	7	151970855	151970855	G	C
MEN0108-P	OR1L3	snp	9	125437821	125437821	G	A
MEN0108-P	CEACAM8	snp	19	43097837	43097837	C	T
MEN0108-P	PRB4	snp	12	11461583	11461583	C	G
MEN0108-P	EPO	snp	7	100320626	100320626	C	T
MEN0108-P	TTC12	snp	11	113194101	113194101	A	C
MEN0108-P	LUM	snp	12	91498084	91498084	T	G
MEN0108-P	SIRPA	snp	20	1902301	1902301	G	A
MEN0108-P	PRB4	snp	12	11461589	11461589	A	G
MEN0108-P	SSX3	snp	X	48206952	48206952	T	C
MEN0108-P	LUM	snp	12	91498084	91498084	T	G
MEN0108-P	OR1L3	snp	9	125437821	125437821	G	A

MEN0108-P	TMPRSS6	snp	22	37494473	37494473	C	T
MEN0108-P	EPO	snp	7	100320626	100320626	C	T
MEN0108-P	KMT2C	snp	7	151970855	151970855	G	C
MEN0108-P	TTC12	snp	11	113194101	113194101	A	C
MEN0108-P	MMP19	snp	12	56230872	56230872	C	T
MEN0108-P	SIRPA	snp	20	1902301	1902301	G	A
MEN0108-P	SSX3	snp	X	48206952	48206952	T	C
MEN0108-P	CEACAM8	snp	19	43097837	43097837	C	T
MEN0108-P	PRB4	snp	12	11461583	11461583	C	G
MEN0109-P	KLF4	snp	9	110249348	110249348	T	G
MEN0109-P	TOR1A	snp	9	132584984	132584984	C	T
MEN0109-P	SIN3A	snp	15	75684817	75684817	C	T
MEN0109-P	PRSS33	snp	16	2834665	2834665	G	T
MEN0109-P	HMGCR	snp	5	74655888	74655888	G	A
MEN0109-P	ATP1A4	snp	1	160137092	160137092	G	A
MEN0109-P	IKZF4	snp	12	56428651	56428651	G	C
MEN0109-P	MSH6	snp	2	48027231	48027231	G	C
MEN0109-P	GPR26	snp	10	125426309	125426309	A	G
MEN0109-P	TRAF7	snp	16	2226146	2226146	A	G
MEN0109-P	UBL7	snp	15	74741604	74741604	G	C
MEN0109-P	PRB3	snp	12	11420584	11420584	C	T
MEN0109-P	HLA-B	snp	6	31324165	31324165	A	G
MEN0109-P	EID2	snp	19	40030163	40030163	C	A
MEN0109-P	SLCO1C1	snp	12	20874978	20874978	C	A
MEN0109-P	PLEKHA6	snp	1	204198175	204198175	G	A
MEN0109-P	RBAK-RBAKDN	snp	7	5112706	5112706	G	C
MEN0109-P	PKHD1	snp	6	51524329	51524329	C	A
MEN0109-P	SCUBE2	snp	11	9069616	9069616	G	A
MEN0109-P	LIPG	snp	18	47101933	47101933	G	A
MEN0109-P	UBL7	snp	15	74741604	74741604	G	C
MEN0109-P	GPR26	snp	10	125426309	125426309	A	G
MEN0109-P	HMGCR	snp	5	74655888	74655888	G	A
MEN0109-P	TRAF7	snp	16	2226146	2226146	A	G
MEN0109-P	LIPG	snp	18	47101933	47101933	G	A
MEN0109-P	EID2	snp	19	40030163	40030163	C	A
MEN0109-P	PLEKHA6	snp	1	204198175	204198175	G	A
MEN0109-P	SLCO1C1	snp	12	20874978	20874978	C	A
MEN0109-P	PKHD1	snp	6	51524329	51524329	C	A
MEN0109-P	HLA-B	snp	6	31324165	31324165	A	G
MEN0109-P	PRSS33	snp	16	2834665	2834665	G	T
MEN0109-P	SIN3A	snp	15	75684817	75684817	C	T
MEN0109-P	MSH6	snp	2	48027231	48027231	G	C
MEN0109-P	TOR1A	snp	9	132584984	132584984	C	T
MEN0109-P	TOR1A	snp	9	132584984	132584984	C	T
MEN0109-P	ATP1A4	snp	1	160137092	160137092	G	A
MEN0109-P	RBAK-RBAKDN	snp	7	5112706	5112706	G	C
MEN0109-P	SCUBE2	snp	11	9069616	9069616	G	A

MEN0109-P	IKZF4	snp	12	56428651	56428651	G	C
MEN0109-P	KLF4	snp	9	110249348	110249348	T	G
MEN0109-P	PRB3	snp	12	11420584	11420584	C	T
MEN0110-P	FAM169A	snp	5	74109692	74109692	A	G
MEN0110-P	STAR	snp	8	38008284	38008284	C	A
MEN0110-P	OR10A6	snp	11	7950136	7950136	C	A
MEN0110-P	HIVEP1	snp	6	12125120	12125120	C	G
MEN0110-P	LRFN2	snp	6	40400530	40400530	T	G
MEN0110-P	GLI3	snp	7	42005786	42005786	T	C
MEN0110-P	NDUFB5	snp	3	179322625	179322625	C	T
MEN0110-P	POLR2B	snp	4	57891598	57891598	C	T
MEN0110-P	PCDHGB4	snp	5	140769259	140769259	C	A
MEN0110-P	B3GALT5	snp	21	41033394	41033394	G	A
MEN0110-P	CENPI	snp	X	100382161	100382161	C	T
MEN0110-P	FAM222A	snp	12	110205942	110205942	C	T
MEN0110-P	CDKN2A	snp	9	21974573	21974573	T	C
MEN0110-P	LRFN2	snp	6	40400530	40400530	T	G
MEN0110-P	LRFN2	snp	6	40400530	40400530	T	G
MEN0110-P	LRFN2	snp	6	40400530	40400530	T	G
MEN0110-P	LRFN2	snp	6	40400530	40400530	T	G
MEN0110-P	NDUFB5	snp	3	179322625	179322625	C	T
MEN0110-P	PCDHGB4	snp	5	140769259	140769259	C	A
MEN0110-P	FAM222A	snp	12	110205942	110205942	C	T
MEN0110-P	POLR2B	snp	4	57891598	57891598	C	T
MEN0110-P	FAM169A	snp	5	74109692	74109692	A	G
MEN0110-P	STAR	snp	8	38008284	38008284	C	A
MEN0110-P	CDKN2A	snp	9	21974573	21974573	T	C
MEN0110-P	CENPI	snp	X	100382161	100382161	C	T
MEN0110-P	OR10A6	snp	11	7950136	7950136	C	A
MEN0110-P	GLI3	snp	7	42005786	42005786	T	C
MEN0110-P	HIVEP1	snp	6	12125120	12125120	C	G
MEN0110-P	B3GALT5	snp	21	41033394	41033394	G	A
MEN0111-P	VANGL1	snp	1	116206697	116206697	G	A
MEN0111-P	OR10Q1	snp	11	57995474	57995474	G	T
MEN0111-P	NF2	snp	22	30054233	30054233	G	A
MEN0111-P	SLC35G6	snp	17	7386121	7386121	C	T
MEN0111-P	CDK5R1	snp	17	30815311	30815311	G	A
MEN0111-P	SOGA2	snp	18	8793011	8793011	G	A
MEN0111-P	SUGP2	snp	19	19115445	19115445	C	A
MEN0111-P	ENAM	snp	4	71508093	71508093	G	A
MEN0111-P	PITPNC1	snp	17	65688811	65688811	G	A
MEN0111-P	IGHV3-30	snp	14	106791295	106791295	C	A
MEN0111-P	AHNAK2	snp	14	105415786	105415786	G	A
MEN0111-P	IFNA2	snp	9	21384781	21384781	C	T
MEN0111-P	AHNAK	snp	11	62295549	62295549	C	T
MEN0111-P	ZNF878	snp	19	12155673	12155673	A	C
MEN0111-P	POLR3C	snp	1	145608598	145608598	C	T

MEN0111-P	MUC17	snp	7	100682124	100682124	T	A
MEN0111-P	PHLDA1	snp	12	76424708	76424708	A	T
MEN0111-P	UBBP4	snp	17	21731144	21731144	T	G
MEN0111-P	CUL4B	snp	X	119693998	119693998	A	G
MEN0111-P	ZNF57	snp	19	2917842	2917842	G	A
MEN0111-P	SMG1	snp	16	18937330	18937330	T	C
MEN0111-P	RPTN	snp	1	152128689	152128689	C	T
MEN0111-P	TCHH	snp	1	152082220	152082220	G	C
MEN0111-P	MAPKBP1	snp	15	42067544	42067544	G	A
MEN0111-P	MAPKBP1	snp	15	42067544	42067544	G	A
MEN0111-P	SUGP2	snp	19	19115445	19115445	C	A
MEN0111-P	SLC35G6	snp	17	7386121	7386121	C	T
MEN0111-P	ZNF57	snp	19	2917842	2917842	G	A
MEN0111-P	OR10Q1	snp	11	57995474	57995474	G	T
MEN0111-P	VANGL1	snp	1	116206697	116206697	G	A
MEN0111-P	IFNA2	snp	9	21384781	21384781	C	T
MEN0111-P	UBBP4	snp	17	21731144	21731144	T	G
MEN0111-P	ZNF878	snp	19	12155673	12155673	A	C
MEN0111-P	AHNAK2	snp	14	105415786	105415786	G	A
MEN0111-P	PITPNC1	snp	17	65688811	65688811	G	A
MEN0111-P	NF2	snp	22	30054233	30054233	G	A
MEN0111-P	CDK5R1	snp	17	30815311	30815311	G	A
MEN0111-P	POLR3C	snp	1	145608598	145608598	C	T
MEN0111-P	AHNAK	snp	11	62295549	62295549	C	T
MEN0111-P	IGHV3-30	snp	14	106791295	106791295	C	A
MEN0111-P	PHLDA1	snp	12	76424708	76424708	A	T
MEN0111-P	SOGA2	snp	18	8793011	8793011	G	A
MEN0111-P	CUL4B	snp	X	119693998	119693998	A	G
MEN0111-P	MUC17	snp	7	100682124	100682124	T	A
MEN0111-P	ENAM	snp	4	71508093	71508093	G	A
MEN0111-P	TCHH	snp	1	152082220	152082220	G	C
MEN0111-P	SMG1	snp	16	18937330	18937330	T	C
MEN0111-P	RPTN	snp	1	152128689	152128689	C	T
MEN0112-P	ZNF681	snp	19	23927139	23927139	T	G
MEN0112-P	KRTAP19-3	snp	21	31864074	31864074	G	A
MEN0112-P	MYO15A	snp	17	18023231	18023231	G	A
MEN0112-P	SCN5A	snp	3	38671826	38671826	G	A
MEN0112-P	CCDC82	snp	11	96086844	96086844	G	A
MEN0112-P	MASTL	snp	10	27462084	27462084	G	A
MEN0112-P	KRT26	snp	17	38922856	38922856	G	A
MEN0112-P	RNF144A	snp	2	7170338	7170338	C	T
MEN0112-P	REPS2	snp	X	17024441	17024441	C	T
MEN0112-P	TPTE2	snp	13	20056679	20056679	T	G
MEN0112-P	DEDD	snp	1	161092178	161092178	C	T
MEN0112-P	ZNF563	snp	19	12429722	12429722	T	C
MEN0112-P	FRG1B	snp	20	29628243	29628243	T	C
MEN0112-P	DOCK11	snp	X	117814641	117814641	G	A

MEN0112-P	MYO1D	snp	17	31039073	31039073	C	T
MEN0112-P	ZNF714	snp	19	21300738	21300738	T	C
MEN0112-P	LRRTM4	snp	2	77746912	77746912	G	A
MEN0112-P	RBM22	snp	5	150071393	150071393	G	A
MEN0112-P	CNTNAP2	snp	7	146741075	146741075	G	A
MEN0112-P	WDR65	snp	1	43663300	43663300	G	A
MEN0112-P	NBPF3	snp	1	21806573	21806573	A	G
MEN0112-P	TRIP12	snp	2	230723956	230723956	G	A
MEN0112-P	FRAS1	snp	4	79410104	79410104	G	A
MEN0112-P	GPR179	snp	17	36499269	36499269	C	T
MEN0112-P	AKT1	snp	14	105246551	105246551	C	T
MEN0112-P	CDC40	snp	6	110551173	110551173	G	A
MEN0112-P	FRG1B	snp	20	29628245	29628245	G	A
MEN0112-P	DOK1	snp	2	74783547	74783547	G	A
MEN0112-P	TMED6	snp	16	69385478	69385478	G	C
MEN0112-P	NCAPD3	snp	11	134054813	134054813	G	A
MEN0112-P	ANKZF1	snp	2	220098094	220098094	G	A
MEN0112-P	CYP21A2	snp	6	32007918	32007918	G	A
MEN0112-P	TRAF7	snp	16	2223978	2223978	C	G
MEN0112-P	RPTN	snp	1	152128689	152128689	C	T
MEN0112-P	ACRC	snp	X	70823743	70823743	G	A
MEN0112-P	FLG2	snp	1	152325523	152325523	G	C
MEN0112-P	TMC6	snp	17	76118730	76118730	G	A
MEN0112-P	RBM15	snp	1	110884048	110884048	G	A
MEN0112-P	TAF3	snp	10	8006836	8006836	G	A
MEN0112-P	AKT1	snp	14	105246551	105246551	C	T
MEN0112-P	REPS2	snp	X	17024441	17024441	C	T
MEN0112-P	SCN5A	snp	3	38671826	38671826	G	A
MEN0112-P	FRG1B	snp	20	29628243	29628243	T	C
MEN0112-P	ANKZF1	snp	2	220098094	220098094	G	A
MEN0112-P	TPTE2	snp	13	20056679	20056679	T	G
MEN0112-P	WDR65	snp	1	43663300	43663300	G	A
MEN0112-P	MYO1D	snp	17	31039073	31039073	C	T
MEN0112-P	DEDD	snp	1	161092178	161092178	C	T
MEN0112-P	CYP21A2	snp	6	32007918	32007918	G	A
MEN0112-P	RNF144A	snp	2	7170338	7170338	C	T
MEN0112-P	ZNF563	snp	19	12429722	12429722	T	C
MEN0112-P	KRT26	snp	17	38922856	38922856	G	A
MEN0112-P	LRRTM4	snp	2	77746912	77746912	G	A
MEN0112-P	TRIP12	snp	2	230723956	230723956	G	A
MEN0112-P	TRAF7	snp	16	2223978	2223978	C	G
MEN0112-P	ZNF681	snp	19	23927139	23927139	T	G
MEN0112-P	GPR179	snp	17	36499269	36499269	C	T
MEN0112-P	TMC6	snp	17	76118730	76118730	G	A
MEN0112-P	RBM22	snp	5	150071393	150071393	G	A
MEN0112-P	NCAPD3	snp	11	134054813	134054813	G	A
MEN0112-P	CDC40	snp	6	110551173	110551173	G	A

MEN0112-P	DOCK11	snp	X	117814641	117814641	G	A
MEN0112-P	KRTAP19-3	snp	21	31864074	31864074	G	A
MEN0112-P	TAF3	snp	10	8006836	8006836	G	A
MEN0112-P	MASTL	snp	10	27462084	27462084	G	A
MEN0112-P	DOK1	snp	2	74783547	74783547	G	A
MEN0112-P	CNTNAP2	snp	7	146741075	146741075	G	A
MEN0112-P	MYO15A	snp	17	18023231	18023231	G	A
MEN0112-P	FLG2	snp	1	152325523	152325523	G	C
MEN0112-P	RP11-343C2.7	snp	16	69385478	69385478	G	C
MEN0112-P	FRAS1	snp	4	79410104	79410104	G	A
MEN0112-P	CCDC82	snp	11	96086844	96086844	G	A
MEN0112-P	ZNF714	snp	19	21300738	21300738	T	C
MEN0112-P	NBPF3	snp	1	21806573	21806573	A	G
MEN0112-P	RPTN	snp	1	152128689	152128689	C	T
MEN0112-P	RBM15	snp	1	110884048	110884048	G	A
MEN0112-P	ACRC	snp	X	70823743	70823743	G	A
MEN0113-P	DGKE	snp	17	54912308	54912308	G	A
MEN0113-P	PRPF8	snp	17	1584844	1584844	G	A
MEN0113-P	MAGOH	snp	1	53692748	53692748	A	G
MEN0113-P	GRK5	snp	10	121214568	121214568	G	A
MEN0113-P	OR5M1	snp	11	56380819	56380819	G	T
MEN0113-P	ZNF844	snp	19	12187275	12187275	G	C
MEN0113-P	ARFGEF1	snp	8	68140356	68140356	G	C
MEN0113-P	ZNF253	snp	19	20002885	20002885	G	A
MEN0113-P	EDC4	snp	16	67911491	67911491	A	G
MEN0113-P	ZNF253	snp	19	20002883	20002883	T	A
MEN0113-P	TCHH	snp	1	152084423	152084423	G	C
MEN0113-P	PRDX6	snp	1	173446601	173446601	G	A
MEN0113-P	SMARCA1	snp	X	128657281	128657281	C	A
MEN0113-P	PLA2R1	snp	2	160804026	160804026	T	C
MEN0113-P	SREK1	snp	5	65458302	65458302	C	T
MEN0113-P	ACRC	snp	X	70823870	70823870	G	A
MEN0113-P	OR5M1	snp	11	56380819	56380819	G	T
MEN0113-P	SMARCA1	snp	X	128657281	128657281	C	A
MEN0113-P	PRPF8	snp	17	1584844	1584844	G	A
MEN0113-P	ZNF253	snp	19	20002883	20002883	T	A
MEN0113-P	MAGOH	snp	1	53692748	53692748	A	G
MEN0113-P	ZNF844	snp	19	12187275	12187275	G	C
MEN0113-P	PLA2R1	snp	2	160804026	160804026	T	C
MEN0113-P	SREK1	snp	5	65458302	65458302	C	T
MEN0113-P	ARFGEF1	snp	8	68140356	68140356	G	C
MEN0113-P	EDC4	snp	16	67911491	67911491	A	G
MEN0113-P	DGKE	snp	17	54912308	54912308	G	A
MEN0113-P	PRDX6	snp	1	173446601	173446601	G	A
MEN0113-P	GRK5	snp	10	121214568	121214568	G	A
MEN0113-P	ACRC	snp	X	70823870	70823870	G	A
MEN0113-P	TCHH	snp	1	152084423	152084423	G	C

MEN0114-P	ZNF878	snp	19	12155673	12155673	A	C
MEN0114-P	SLC26A9	snp	1	205902078	205902078	G	A
MEN0114-P	CYP2D7P	snp	22	42538870	42538870	A	C
MEN0114-P	MUC17	snp	7	100683689	100683689	C	T
MEN0114-P	PLD3	snp	19	40883780	40883780	G	A
MEN0114-P	PSG11	snp	19	43528993	43528993	C	T
MEN0114-P	LRIG3	snp	12	59284514	59284514	A	C
MEN0114-P	SRSF8	snp	11	94801036	94801036	C	G
MEN0114-P	CTR9	snp	11	10791833	10791833	A	C
MEN0114-P	GPR3	snp	1	27721240	27721240	G	A
MEN0114-P	DDX27	snp	20	47852916	47852916	G	A
MEN0114-P	SLC12A6	snp	15	34551102	34551102	C	T
MEN0114-P	SON	snp	21	34923783	34923783	A	G
MEN0114-P	ADCY10	snp	1	167871047	167871047	C	T
MEN0114-P	LCA5L	snp	21	40777986	40777986	T	A
MEN0114-P	LRP2	snp	2	170112684	170112684	C	A
MEN0114-P	PTPRH	snp	19	55711693	55711693	C	A
MEN0114-P	PAK3	snp	X	110406794	110406794	A	C
MEN0114-P	NLRP1	snp	17	5462323	5462323	C	T
MEN0114-P	NUP205	snp	7	135282939	135282939	G	A
MEN0114-P	DHX38	snp	16	72132855	72132855	C	T
MEN0114-P	LIPN	snp	10	90521985	90521985	A	G
MEN0114-P	TUB	snp	11	8111174	8111174	G	A
MEN0114-P	FOLH1	snp	11	49208203	49208203	C	A
MEN0114-P	ARL4D	snp	17	41477249	41477249	T	G
MEN0114-P	RAB14	snp	9	123952919	123952919	T	A
MEN0114-P	RB1CC1	snp	8	53586653	53586653	C	T
MEN0114-P	ANKRD36	snp	2	97869931	97869931	A	T
MEN0114-P	ZNF814	snp	19	58385546	58385546	G	T
MEN0114-P	RGPD3	snp	2	107049681	107049681	T	C
MEN0114-P	FLG	snp	1	152280877	152280877	C	A
MEN0114-P	CLUH	snp	17	2600121	2600121	C	T
MEN0114-P	ZCCHC4	snp	4	25315716	25315716	G	A
MEN0114-P	NUP205	snp	7	135282939	135282939	G	A
MEN0114-P	LRP2	snp	2	170112684	170112684	C	A
MEN0114-P	LRP2	snp	2	170112684	170112684	C	A
MEN0114-P	CYP2D7P	snp	22	42538870	42538870	A	C
MEN0114-P	SLC26A9	snp	1	205902078	205902078	G	A
MEN0114-P	DDX27	snp	20	47852916	47852916	G	A
MEN0114-P	MUC17	snp	7	100683689	100683689	C	T
MEN0114-P	SRSF8	snp	11	94801036	94801036	C	G
MEN0114-P	NLRP1	snp	17	5462323	5462323	C	T
MEN0114-P	FOLH1	snp	11	49208203	49208203	C	A
MEN0114-P	PAK3	snp	X	110406794	110406794	A	C
MEN0114-P	ARL4D	snp	17	41477249	41477249	T	G
MEN0114-P	LCA5L	snp	21	40777986	40777986	T	A
MEN0114-P	CTR9	snp	11	10791833	10791833	A	C

MEN0114-P	ADCY10	snp	1	167871047	167871047	C	T
MEN0114-P	SON	snp	21	34923783	34923783	A	G
MEN0114-P	CLUH	snp	17	2600121	2600121	C	T
MEN0114-P	ZNF878	snp	19	12155673	12155673	A	C
MEN0114-P	PTPRH	snp	19	55711693	55711693	C	A
MEN0114-P	ZCCHC4	snp	4	25315716	25315716	G	A
MEN0114-P	SLC12A6	snp	15	34551102	34551102	C	T
MEN0114-P	PSG11	snp	19	43528993	43528993	C	T
MEN0114-P	ZNF814	snp	19	58385546	58385546	G	T
MEN0114-P	RAB14	snp	9	123952919	123952919	T	A
MEN0114-P	LRIG3	snp	12	59284514	59284514	A	C
MEN0114-P	RB1CC1	snp	8	53586653	53586653	C	T
MEN0114-P	PLD3	snp	19	40883780	40883780	G	A
MEN0114-P	GPR3	snp	1	27721240	27721240	G	A
MEN0114-P	RGPD3	snp	2	107049681	107049681	T	C
MEN0114-P	TUB	snp	11	8111174	8111174	G	A
MEN0114-P	LIPN	snp	10	90521985	90521985	A	G
MEN0114-P	FLG	snp	1	152280877	152280877	C	A
MEN0114-P	ANKRD36	snp	2	97869931	97869931	A	T
MEN0114-P	DHX38	snp	16	72132855	72132855	C	T
MEN0115-P	ZAN	snp	7	100358090	100358090	A	C
MEN0115-P	CHEK2	snp	22	29091840	29091840	T	C
MEN0115-P	GOLGA6L18	snp	15	82934804	82934804	T	A
MEN0115-P	MT-CYB	snp	MT	15119	15119	G	A
MEN0115-P	TSC2	snp	16	2138570	2138570	C	T
MEN0115-P	KMT2D	snp	12	49427495	49427495	G	C
MEN0115-P	FRG1B	snp	20	29624081	29624081	A	T
MEN0115-P	NBEA	snp	13	36239224	36239224	G	A
MEN0115-P	MT-CYB	snp	MT	15314	15314	G	A
MEN0115-P	PRAMEF11	snp	1	12885318	12885318	C	T
MEN0115-P	NUP155	snp	5	37349296	37349296	C	T
MEN0115-P	POTEC	snp	18	14537970	14537970	C	T
MEN0115-P	SACS	snp	13	23911063	23911063	C	T
MEN0115-P	DMD	snp	X	32509625	32509625	A	C
MEN0115-P	MT-CYB	snp	MT	15119	15119	G	A
MEN0115-P	MT-CYB	snp	MT	15314	15314	G	A
MEN0115-P	ZAN	snp	7	100358090	100358090	A	C
MEN0115-P	KMT2D	snp	12	49427495	49427495	G	C
MEN0115-P	SACS	snp	13	23911063	23911063	C	T
MEN0115-P	FRG1B	snp	20	29624081	29624081	A	T
MEN0115-P	PRAMEF11	snp	1	12885318	12885318	C	T
MEN0115-P	CHEK2	snp	22	29091840	29091840	T	C
MEN0115-P	DMD	snp	X	32509625	32509625	A	C
MEN0115-P	NUP155	snp	5	37349296	37349296	C	T
MEN0115-P	NBEA	snp	13	36239224	36239224	G	A
MEN0115-P	POTEC	snp	18	14537970	14537970	C	T
MEN0115-P	TSC2	snp	16	2138570	2138570	C	T

MEN0115-P	GOLGA6L18	snp	15	82934804	82934804	T	A
MEN0116-P	MT-CO1	snp	MT	5992	5992	G	A
MEN0116-P	MT-ND5	snp	MT	12340	12340	A	T
MEN0116-P	PRAMEF11	snp	1	12887337	12887337	A	G
MEN0116-P	PRAMEF11	snp	1	12887332	12887332	C	A
MEN0116-P	SVEP1	snp	9	113169968	113169968	C	T
MEN0116-P	NF2	snp	22	30054233	30054233	G	A
MEN0116-P	FAT1	snp	4	187518135	187518135	C	A
MEN0116-P	MT-CO2	snp	MT	7691	7691	T	C
MEN0116-P	APOBR	snp	16	28507245	28507245	A	G
MEN0116-P	ARHGEF12	snp	11	120298916	120298916	C	T
MEN0116-P	RANBP2	snp	2	109384211	109384211	G	A
MEN0116-P	PRB4	snp	12	11461589	11461589	A	G
MEN0116-P	FAM155A	snp	13	108518718	108518718	T	C
MEN0116-P	MT-CO2	snp	MT	7691	7691	T	C
MEN0116-P	MT-CO1	snp	MT	5992	5992	G	A
MEN0116-P	PRAMEF11	snp	1	12887332	12887332	C	A
MEN0116-P	MT-ND5	snp	MT	12340	12340	A	T
MEN0116-P	FAT1	snp	4	187518135	187518135	C	A
MEN0116-P	ARHGEF12	snp	11	120298916	120298916	C	T
MEN0116-P	NF2	snp	22	30054233	30054233	G	A
MEN0116-P	APOBR	snp	16	28507245	28507245	A	G
MEN0116-P	SVEP1	snp	9	113169968	113169968	C	T
MEN0116-P	RANBP2	snp	2	109384211	109384211	G	A
MEN0116-P	FAM155A	snp	13	108518718	108518718	T	C
MEN0116-P	PRB4	snp	12	11461589	11461589	A	G
MEN0117-P	TPTE	snp	21	10952935	10952935	T	C
MEN0117-P	POTEC	snp	18	14543057	14543057	T	G
MEN0117-P	RGPD3	snp	2	107049698	107049698	A	G
MEN0117-P	POTEC	snp	18	14537949	14537949	A	G
MEN0117-P	ESRP1	snp	8	95654206	95654206	T	C
MEN0117-P	RGPD3	snp	2	107049710	107049710	A	C
MEN0117-P	ANKAR	snp	2	190597863	190597863	G	A
MEN0117-P	MT-ATP6	snp	MT	8932	8932	C	T
MEN0117-P	GOLGA6L18	snp	15	82934804	82934804	T	A
MEN0117-P	ARID2	snp	12	46123850	46123850	C	A
MEN0117-P	LRP1B	snp	2	141739831	141739831	C	A
MEN0117-P	CCNB1	snp	5	68463814	68463814	C	T
MEN0117-P	RANBP2	snp	2	109347314	109347314	C	G
MEN0117-P	PREX2	snp	8	68989671	68989671	G	A
MEN0117-P	MEGF8	snp	19	42857184	42857184	C	T
MEN0117-P	ABL1	snp	9	133760171	133760171	C	G
MEN0117-P	PRG4	snp	1	186276814	186276814	G	A
MEN0117-P	HMGB1	snp	13	31035509	31035509	A	T
MEN0117-P	CCNB1	snp	5	68463814	68463814	C	T
MEN0117-P	ABL1	snp	9	133760171	133760171	C	G
MEN0117-P	TPTE	snp	21	10952935	10952935	T	C

MEN0117-P	MEGF8	snp	19	42857184	42857184	C	T
MEN0117-P	RANBP2	snp	2	109347314	109347314	C	G
MEN0117-P	ANKAR	snp	2	190597863	190597863	G	A
MEN0117-P	ARID2	snp	12	46123850	46123850	C	A
MEN0117-P	MT-ATP6	snp	MT	8932	8932	C	T
MEN0117-P	POTEC	snp	18	14543057	14543057	T	G
MEN0117-P	RGPD3	snp	2	107049698	107049698	A	G
MEN0117-P	LRP1B	snp	2	141739831	141739831	C	A
MEN0117-P	PREX2	snp	8	68989671	68989671	G	A
MEN0117-P	ESRP1	snp	8	95654206	95654206	T	C
MEN0117-P	POTEC	snp	18	14537949	14537949	A	G
MEN0117-P	GOLGA6L18	snp	15	82934804	82934804	T	A
MEN0117-P	PRG4	snp	1	186276814	186276814	G	A
MEN0117-P	HMGB1	snp	13	31035509	31035509	A	T
MEN0118-P0	MUC7	snp	4	71347185	71347185	T	C
MEN0118-P0	DCLK1	snp	13	36700147	36700147	C	A
MEN0118-P0	CRISPLD1	snp	8	75932247	75932247	G	T
MEN0118-P0	ELTD1	snp	1	79392671	79392671	T	G
MEN0118-P0	DISP1	snp	1	223176649	223176649	C	T
MEN0118-P0	MUC4	snp	3	195505836	195505836	G	C
MEN0118-P0	DMD	snp	X	32456492	32456492	T	A
MEN0118-P0	TTN	snp	2	179659702	179659702	C	A
MEN0118-P0	EPAS1	snp	2	46607399	46607399	G	A
MEN0118-P0	CH25H	snp	10	90966460	90966460	G	A
MEN0118-P0	TTN	snp	2	179532174	179532174	G	A
MEN0118-P0	ACBD5	snp	10	27499994	27499994	G	A
MEN0118-P0	NOS3	snp	7	150704077	150704077	G	A
MEN0118-P0	PPL	snp	16	4934500	4934500	G	A
MEN0118-P0	ABCA4	snp	1	94463499	94463499	G	A
MEN0118-P0	AMER2	snp	13	25744296	25744296	C	A
MEN0118-P0	POTEC	snp	18	14513764	14513764	C	T
MEN0118-P0	KCNQ5	snp	6	73902363	73902363	G	C
MEN0118-P0	MPZL2	snp	11	118133212	118133212	T	G
MEN0118-P0	FAM91A1	snp	8	124797968	124797968	G	A
MEN0118-P0	SNRK	snp	3	43389633	43389633	G	A
MEN0118-P0	PTPN3	snp	9	112168799	112168799	T	A
MEN0118-P0	IMPG2	snp	3	100976379	100976379	T	A
MEN0118-P0	PCNT	snp	21	47852061	47852061	G	A
MEN0118-P0	IL5	snp	5	131877714	131877714	C	T
MEN0118-P0	SHCBP1	snp	16	46651598	46651598	G	A
MEN0118-P0	DMD	snp	X	32486629	32486629	G	C
MEN0118-P0	UQCRFS1	snp	19	29698751	29698751	G	A
MEN0118-P0	SH2D2A	snp	1	156777073	156777073	T	G
MEN0118-P0	RSPRY1	snp	16	57255184	57255184	G	A
MEN0118-P0	ABCA4	snp	1	94463499	94463499	G	A
MEN0118-P0	DCLK1	snp	13	36700147	36700147	C	A
MEN0118-P0	SHCBP1	snp	16	46651598	46651598	G	A

MEN0118-P0	RSPRY1	snp	16	57255184	57255184	G	A
MEN0118-P0	PTPN3	snp	9	112168799	112168799	T	A
MEN0118-P0	IMPG2	snp	3	100976379	100976379	T	A
MEN0118-P0	CH25H	snp	10	90966460	90966460	G	A
MEN0118-P0	PPL	snp	16	4934500	4934500	G	A
MEN0118-P0	DMD	snp	X	32486629	32486629	G	C
MEN0118-P0	FAM91A1	snp	8	124797968	124797968	G	A
MEN0118-P0	DISP1	snp	1	223176649	223176649	C	T
MEN0118-P0	IL5	snp	5	131877714	131877714	C	T
MEN0118-P0	TTN	snp	2	179659702	179659702	C	A
MEN0118-P0	ELTD1	snp	1	79392671	79392671	T	G
MEN0118-P0	KCNQ5	snp	6	73902363	73902363	G	C
MEN0118-P0	AMER2	snp	13	25744296	25744296	C	A
MEN0118-P0	ACBD5	snp	10	27499994	27499994	G	A
MEN0118-P0	DMD	snp	X	32456492	32456492	T	A
MEN0118-P0	CRISPLD1	snp	8	75932247	75932247	G	T
MEN0118-P0	UQCRFS1	snp	19	29698751	29698751	G	A
MEN0118-P0	SNRK	snp	3	43389633	43389633	G	A
MEN0118-P0	TTN	snp	2	179532174	179532174	G	A
MEN0118-P0	SH2D2A	snp	1	156777073	156777073	T	G
MEN0118-P0	NOS3	snp	7	150704077	150704077	G	A
MEN0118-P0	EPAS1	snp	2	46607399	46607399	G	A
MEN0118-P0	PCNT	snp	21	47852061	47852061	G	A
MEN0118-P0	MUC4	snp	3	195505836	195505836	G	C
MEN0118-P0	MPZL2	snp	11	118133212	118133212	T	G
MEN0118-P0	POTEC	snp	18	14513764	14513764	C	T
MEN0118-P0	MUC7	snp	4	71347185	71347185	T	C
MEN0118-P1	C1orf95	snp	1	226789707	226789707	G	T
MEN0118-P1	TSPAN18	snp	11	44928010	44928010	G	T
MEN0118-P1	MUC17	snp	7	100683801	100683801	G	A
MEN0118-P1	CRISPLD1	snp	8	75932247	75932247	G	T
MEN0118-P1	ELTD1	snp	1	79392671	79392671	T	G
MEN0118-P1	DMD	snp	X	32456492	32456492	T	A
MEN0118-P1	TTN	snp	2	179659702	179659702	C	A
MEN0118-P1	USP42	snp	7	6175546	6175546	G	A
MEN0118-P1	EPAS1	snp	2	46607399	46607399	G	A
MEN0118-P1	CHEK2	snp	22	29091840	29091840	T	C
MEN0118-P1	TOM1L1	snp	17	52993164	52993164	G	A
MEN0118-P1	CHRND	snp	2	233396266	233396266	G	A
MEN0118-P1	TTN	snp	2	179532174	179532174	G	A
MEN0118-P1	C6orf25	snp	6	31692375	31692375	C	T
MEN0118-P1	QRICH2	snp	17	74289520	74289520	G	A
MEN0118-P1	RPAP3	snp	12	48091499	48091499	G	A
MEN0118-P1	ABCA4	snp	1	94463499	94463499	G	A
MEN0118-P1	AMER2	snp	13	25744296	25744296	C	A
MEN0118-P1	NOL6	snp	9	33467193	33467193	C	T
MEN0118-P1	REL	snp	2	61118941	61118941	G	A

MEN0118-P1	POTEC	snp	18	14513764	14513764	C	T
MEN0118-P1	ICA1	snp	7	8258024	8258024	T	A
MEN0118-P1	FAM91A1	snp	8	124797968	124797968	G	A
MEN0118-P1	PEG3	snp	19	57328801	57328801	G	A
MEN0118-P1	SNRK	snp	3	43389633	43389633	G	A
MEN0118-P1	PTPN3	snp	9	112168799	112168799	T	A
MEN0118-P1	DNAJB1	snp	19	14626895	14626895	G	A
MEN0118-P1	KLHL42	snp	12	27950726	27950726	G	A
MEN0118-P1	PAMR1	snp	11	35456101	35456101	G	A
MEN0118-P1	LHCGR	snp	2	48914996	48914996	C	T
MEN0118-P1	PIP5K1C	snp	19	3642936	3642936	G	A
MEN0118-P1	MMP14	snp	14	23310742	23310742	C	T
MEN0118-P1	DMD	snp	X	32486629	32486629	G	C
MEN0118-P1	ZNF208	snp	19	22154342	22154342	A	C
MEN0118-P1	RSPRY1	snp	16	57255184	57255184	G	A
MEN0118-P1	ZNF814	snp	19	58385546	58385546	G	T
MEN0118-P1	CNGA1	snp	4	47938974	47938974	C	T
MEN0118-P1	GRHL1	snp	2	10104056	10104056	G	A
MEN0118-P1	ABCA4	snp	1	94463499	94463499	G	A
MEN0118-P1	TOM1L1	snp	17	52993164	52993164	G	A
MEN0118-P1	C1orf95	snp	1	226789707	226789707	G	T
MEN0118-P1	C6orf25	snp	6	31692375	31692375	C	T
MEN0118-P1	RSPRY1	snp	16	57255184	57255184	G	A
MEN0118-P1	CNGA1	snp	4	47938974	47938974	C	T
MEN0118-P1	PTPN3	snp	9	112168799	112168799	T	A
MEN0118-P1	USP42	snp	7	6175546	6175546	G	A
MEN0118-P1	CHEK2	snp	22	29091840	29091840	T	C
MEN0118-P1	DMD	snp	X	32486629	32486629	G	C
MEN0118-P1	TSPAN18	snp	11	44928010	44928010	G	T
MEN0118-P1	FAM91A1	snp	8	124797968	124797968	G	A
MEN0118-P1	GRHL1	snp	2	10104056	10104056	G	A
MEN0118-P1	TTN	snp	2	179659702	179659702	C	A
MEN0118-P1	ELTD1	snp	1	79392671	79392671	T	G
MEN0118-P1	CHRND	snp	2	233396266	233396266	G	A
MEN0118-P1	ICA1	snp	7	8258024	8258024	T	A
MEN0118-P1	KLHL42	snp	12	27950726	27950726	G	A
MEN0118-P1	AMER2	snp	13	25744296	25744296	C	A
MEN0118-P1	DMD	snp	X	32456492	32456492	T	A
MEN0118-P1	CRISPLD1	snp	8	75932247	75932247	G	T
MEN0118-P1	NOL6	snp	9	33467193	33467193	C	T
MEN0118-P1	PAMR1	snp	11	35456101	35456101	G	A
MEN0118-P1	LHCGR	snp	2	48914996	48914996	C	T
MEN0118-P1	SNRK	snp	3	43389633	43389633	G	A
MEN0118-P1	TTN	snp	2	179532174	179532174	G	A
MEN0118-P1	DNAJB1	snp	19	14626895	14626895	G	A
MEN0118-P1	MMP14	snp	14	23310742	23310742	C	T
MEN0118-P1	QRICH2	snp	17	74289520	74289520	G	A

MEN0118-P1	ZNF814	snp	19	58385546	58385546	G	T
MEN0118-P1	REL	snp	2	61118941	61118941	G	A
MEN0118-P1	ZNF208	snp	19	22154342	22154342	A	C
MEN0118-P1	RPAP3	snp	12	48091499	48091499	G	A
MEN0118-P1	EPAS1	snp	2	46607399	46607399	G	A
MEN0118-P1	PIP5K1C	snp	19	3642936	3642936	G	A
MEN0118-P1	PEG3	snp	19	57328801	57328801	G	A
MEN0118-P1	POTEC	snp	18	14513764	14513764	C	T
MEN0118-P1	MUC17	snp	7	100683801	100683801	G	A
MEN0119-P0	ZDHHC14	snp	6	157963631	157963631	C	T
MEN0119-P0	NCAPG	snp	4	17844978	17844978	G	A
MEN0119-P0	NBPF3	snp	1	21806564	21806564	A	C
MEN0119-P0	SLC6A6	snp	3	14489143	14489143	G	A
MEN0119-P0	MUC17	snp	7	100682094	100682094	C	T
MEN0119-P0	ZNF681	snp	19	23926839	23926839	A	G
MEN0119-P0	ZNF93	snp	19	20045361	20045361	A	G
MEN0119-P0	ZNF747	snp	16	30545892	30545892	C	A
MEN0119-P0	CNTRL	snp	9	123860776	123860776	G	A
MEN0119-P0	NPIPB5	snp	16	22545865	22545865	G	C
MEN0119-P0	ZNF737	snp	19	20728254	20728254	C	G
MEN0119-P0	ARHGAP31	snp	3	119134679	119134679	G	C
MEN0119-P0	MYH7B	snp	20	33581993	33581993	C	T
MEN0119-P0	ZNF493	snp	19	21606614	21606614	G	A
MEN0119-P0	CSRNP1	snp	3	39186567	39186567	G	A
MEN0119-P0	INTS6	snp	13	52026580	52026580	C	T
MEN0119-P0	NOL8	snp	9	95077417	95077417	C	A
MEN0119-P0	ZAN	snp	7	100350474	100350474	T	C
MEN0119-P0	ZNF208	snp	19	22156766	22156766	G	A
MEN0119-P0	RPTN	snp	1	152128485	152128485	C	T
MEN0119-P0	NBPF14	snp	1	148012533	148012533	T	G
MEN0119-P0	NPC1	snp	18	21125070	21125070	T	C
MEN0119-P0	TRIM42	snp	3	140401786	140401786	A	C
MEN0119-P0	CPT1B	snp	22	51009914	51009914	C	T
MEN0119-P0	S100B	snp	21	48022235	48022235	C	T
MEN0119-P0	EPS8	snp	12	15811507	15811507	G	A
MEN0119-P0	NXF1	snp	11	62571391	62571391	G	A
MEN0119-P0	C2	snp	6	31911969	31911969	G	A
MEN0119-P0	MUC4	snp	3	195505836	195505836	G	C
MEN0119-P0	RPTN	snp	1	152128464	152128464	T	C
MEN0119-P0	GLI1	snp	12	57860065	57860065	G	A
MEN0119-P0	RPTN	snp	1	152128689	152128689	C	T
MEN0119-P0	FBXL3	snp	13	77592829	77592829	G	A
MEN0119-P0	CCDC82	snp	11	96098268	96098268	G	C
MEN0119-P0	KRTAP1-5	snp	17	39183145	39183145	A	G
MEN0119-P0	ZNF141	snp	4	367058	367058	G	A
MEN0119-P0	MUC17	snp	7	100684301	100684301	A	C
MEN0119-P0	ZCWPW1	snp	7	99998657	99998657	C	T

MEN0119-P0	HIST1H1B	snp	6	27835139	27835139	G	A
MEN0119-P0	PCLO	snp	7	82784468	82784468	C	T
MEN0119-P0	KRTAP21-2	snp	21	32119350	32119350	A	C
MEN0119-P0	HTT	snp	4	3129152	3129152	A	G
MEN0119-P0	ZNF208	snp	19	22154342	22154342	A	C
MEN0119-P0	MUC17	snp	7	100681940	100681940	A	G
MEN0119-P0	CGREF1	snp	2	27324405	27324405	T	C
MEN0119-P0	CGREF1	snp	2	27324405	27324405	T	C
MEN0119-P0	ZNF141	snp	4	367058	367058	G	A
MEN0119-P0	ZNF208	snp	19	22156766	22156766	G	A
MEN0119-P0	ZNF493	snp	19	21606614	21606614	G	A
MEN0119-P0	SLC6A6	snp	3	14489143	14489143	G	A
MEN0119-P0	ZNF737	snp	19	20728254	20728254	C	G
MEN0119-P0	HIST1H1B	snp	6	27835139	27835139	G	A
MEN0119-P0	NCAPG	snp	4	17844978	17844978	G	A
MEN0119-P0	ZDHHC14	snp	6	157963631	157963631	C	T
MEN0119-P0	MYH7B	snp	20	33581993	33581993	C	T
MEN0119-P0	ZNF93	snp	19	20045361	20045361	A	G
MEN0119-P0	INTS6	snp	13	52026580	52026580	C	T
MEN0119-P0	ARHGAP31	snp	3	119134679	119134679	G	C
MEN0119-P0	EPS8	snp	12	15811507	15811507	G	A
MEN0119-P0	NPC1	snp	18	21125070	21125070	T	C
MEN0119-P0	S100B	snp	21	48022235	48022235	C	T
MEN0119-P0	C2	snp	6	31911969	31911969	G	A
MEN0119-P0	CSRNP1	snp	3	39186567	39186567	G	A
MEN0119-P0	CNTRL	snp	9	123860776	123860776	G	A
MEN0119-P0	ZNF208	snp	19	22154342	22154342	A	C
MEN0119-P0	ZNF681	snp	19	23926839	23926839	A	G
MEN0119-P0	MUC17	snp	7	100682094	100682094	C	T
MEN0119-P0	ZNF747	snp	16	30545892	30545892	C	A
MEN0119-P0	CPT1B	snp	22	51009914	51009914	C	T
MEN0119-P0	MUC17	snp	7	100681940	100681940	A	G
MEN0119-P0	TRIM42	snp	3	140401786	140401786	A	C
MEN0119-P0	CCDC82	snp	11	96098268	96098268	G	C
MEN0119-P0	NOL8	snp	9	95077417	95077417	C	A
MEN0119-P0	FBXL3	snp	13	77592829	77592829	G	A
MEN0119-P0	MUC4	snp	3	195505836	195505836	G	C
MEN0119-P0	NBPF14	snp	1	148012533	148012533	T	G
MEN0119-P0	MUC17	snp	7	100684301	100684301	A	C
MEN0119-P0	HTT	snp	4	3129152	3129152	A	G
MEN0119-P0	NBPF3	snp	1	21806564	21806564	A	C
MEN0119-P0	GLI1	snp	12	57860065	57860065	G	A
MEN0119-P0	CGREF1	snp	2	27324405	27324405	T	C
MEN0119-P0	NXF1	snp	11	62571391	62571391	G	A
MEN0119-P0	ZCWPW1	snp	7	99998657	99998657	C	T
MEN0119-P0	NPIPB5	snp	16	22545865	22545865	G	C
MEN0119-P0	KRTAP21-2	snp	21	32119350	32119350	A	C

MEN0119-P0	RPTN	snp	1	152128689	152128689	C	T
MEN0119-P0	RPTN	snp	1	152128485	152128485	C	T
MEN0119-P0	KRTAP1-5	snp	17	39183145	39183145	A	G
MEN0119-P0	RPTN	snp	1	152128464	152128464	T	C
MEN0119-P0	ZAN	snp	7	100350474	100350474	T	C
MEN0119-P0	PCLO	snp	7	82784468	82784468	C	T
MEN0119-P1	TMEM175	snp	4	944278	944278	G	A
MEN0119-P1	GJB6	snp	13	20797397	20797397	G	A
MEN0119-P1	NCAPG	snp	4	17844978	17844978	G	A
MEN0119-P1	EEF1B2	snp	2	207025366	207025366	G	A
MEN0119-P1	OR56A3	snp	11	5969053	5969053	G	A
MEN0119-P1	PDIA2	snp	16	336685	336685	G	A
MEN0119-P1	MYH4	snp	17	10360874	10360874	C	T
MEN0119-P1	DCAF10	snp	9	37861204	37861204	G	T
MEN0119-P1	FRG1B	snp	20	29628245	29628245	G	A
MEN0119-P1	KCNH8	snp	3	19575323	19575323	C	T
MEN0119-P1	NPIPB5	snp	16	22545865	22545865	G	C
MEN0119-P1	PCSK5	snp	9	78773999	78773999	C	T
MEN0119-P1	ARHGAP31	snp	3	119134679	119134679	G	C
MEN0119-P1	CDHR5	snp	11	619539	619539	G	A
MEN0119-P1	LCTL	snp	15	66844071	66844071	G	A
MEN0119-P1	CTTNBP2	snp	7	117400674	117400674	C	G
MEN0119-P1	NOL8	snp	9	95077417	95077417	C	A
MEN0119-P1	ZNF208	snp	19	22156766	22156766	G	A
MEN0119-P1	ADAMTS9	snp	3	64635369	64635369	C	T
MEN0119-P1	CDK7	snp	5	68551329	68551329	T	C
MEN0119-P1	ACSM2B	snp	16	20554485	20554485	G	A
MEN0119-P1	TLN2	snp	15	62944301	62944301	G	C
MEN0119-P1	TSPAN9	snp	12	3387629	3387629	G	A
MEN0119-P1	NPC1	snp	18	21125070	21125070	T	C
MEN0119-P1	TRIM42	snp	3	140401786	140401786	A	C
MEN0119-P1	SETD1A	snp	16	30992415	30992415	C	T
MEN0119-P1	POLR1B	snp	2	113330181	113330181	G	A
MEN0119-P1	ACE2	snp	X	15609901	15609901	C	T
MEN0119-P1	LAMA3	snp	18	21511155	21511155	G	A
MEN0119-P1	EEF1B2	snp	2	207025358	207025358	A	G
MEN0119-P1	ZNF836	snp	19	52659243	52659243	A	C
MEN0119-P1	ZNF780B	snp	19	40541567	40541567	C	T
MEN0119-P1	DFNB31	snp	9	117228555	117228555	C	T
MEN0119-P1	TAF1L	snp	9	32631725	32631725	C	T
MEN0119-P1	RPTN	snp	1	152128689	152128689	C	T
MEN0119-P1	MUC4	snp	3	195511076	195511076	T	A
MEN0119-P1	SIRPA	snp	20	1902301	1902301	G	A
MEN0119-P1	FBXL3	snp	13	77592829	77592829	G	A
MEN0119-P1	CCP110	snp	16	19548834	19548834	C	A
MEN0119-P1	AHNAK	snp	11	62296070	62296070	A	G
MEN0119-P1	AARS	snp	16	70303618	70303618	C	T

MEN0119-P1	HTT	snp	4	3129152	3129152	A	G
MEN0119-P1	ZNF208	snp	19	22156766	22156766	G	A
MEN0119-P1	SETD1A	snp	16	30992415	30992415	C	T
MEN0119-P1	NCAPG	snp	4	17844978	17844978	G	A
MEN0119-P1	TMEM175	snp	4	944278	944278	G	A
MEN0119-P1	CDHR5	snp	11	619539	619539	G	A
MEN0119-P1	PCSK5	snp	9	78773999	78773999	C	T
MEN0119-P1	KCNH8	snp	3	19575323	19575323	C	T
MEN0119-P1	LAMA3	snp	18	21511155	21511155	G	A
MEN0119-P1	CTTNBP2	snp	7	117400674	117400674	C	G
MEN0119-P1	OR56A3	snp	11	5969053	5969053	G	A
MEN0119-P1	ARHGAP31	snp	3	119134679	119134679	G	C
MEN0119-P1	NPC1	snp	18	21125070	21125070	T	C
MEN0119-P1	EEF1B2	snp	2	207025366	207025366	G	A
MEN0119-P1	FRG1B	snp	20	29628245	29628245	G	A
MEN0119-P1	POLR1B	snp	2	113330181	113330181	G	A
MEN0119-P1	GJB6	snp	13	20797397	20797397	G	A
MEN0119-P1	AARS	snp	16	70303618	70303618	C	T
MEN0119-P1	LCTL	snp	15	66844071	66844071	G	A
MEN0119-P1	PDIA2	snp	16	336685	336685	G	A
MEN0119-P1	SIRPA	snp	20	1902301	1902301	G	A
MEN0119-P1	EEF1B2	snp	2	207025358	207025358	A	G
MEN0119-P1	TSPAN9	snp	12	3387629	3387629	G	A
MEN0119-P1	TLN2	snp	15	62944301	62944301	G	C
MEN0119-P1	TAF1L	snp	9	32631725	32631725	C	T
MEN0119-P1	ZNF780B	snp	19	40541567	40541567	C	T
MEN0119-P1	TRIM42	snp	3	140401786	140401786	A	C
MEN0119-P1	ZNF836	snp	19	52659243	52659243	A	C
MEN0119-P1	NOL8	snp	9	95077417	95077417	C	A
MEN0119-P1	MYH4	snp	17	10360874	10360874	C	T
MEN0119-P1	FBXL3	snp	13	77592829	77592829	G	A
MEN0119-P1	DCAF10	snp	9	37861204	37861204	G	T
MEN0119-P1	CDK7	snp	5	68551329	68551329	T	C
MEN0119-P1	AHNAK	snp	11	62296070	62296070	A	G
MEN0119-P1	ADAMTS9	snp	3	64635369	64635369	C	T
MEN0119-P1	ACSM2B	snp	16	20554485	20554485	G	A
MEN0119-P1	HTT	snp	4	3129152	3129152	A	G
MEN0119-P1	CCP110	snp	16	19548834	19548834	C	A
MEN0119-P1	ACE2	snp	X	15609901	15609901	C	T
MEN0119-P1	MUC4	snp	3	195511076	195511076	T	A
MEN0119-P1	DFNB31	snp	9	117228555	117228555	C	T
MEN0119-P1	NPIPB5	snp	16	22545865	22545865	G	C
MEN0119-P1	RPTN	snp	1	152128689	152128689	C	T
MEN0120-P	VPS13A	snp	9	79890997	79890997	A	G
MEN0120-P	DEFB134	snp	8	11853720	11853720	C	A
MEN0120-P	PRAMEF11	snp	1	12887480	12887480	C	T
MEN0120-P	STX16	snp	20	57242559	57242559	G	A

MEN0120-P	ADAMTS7	snp	15	79059041	79059041	T	C
MEN0120-P	CHML	snp	1	241797957	241797957	C	A
MEN0120-P	ATXN7L2	snp	1	110032989	110032989	C	T
MEN0120-P	DDX55	snp	12	124099851	124099851	G	A
MEN0120-P	AC109135.1	snp	GL000192.1	311344	311344	C	T
MEN0120-P	NPHP3	snp	3	132407603	132407603	G	C
MEN0120-P	PRAMEF11	snp	1	12887612	12887612	T	C
MEN0120-P	GABBR1	snp	6	29588897	29588897	G	A
MEN0120-P	OR4P4	snp	11	55406689	55406689	A	G
MEN0120-P	SIGLEC8	snp	19	51961502	51961502	C	A
MEN0120-P	FAT3	snp	11	92565096	92565096	G	T
MEN0120-P	LIN7A	snp	12	81283094	81283094	G	T
MEN0120-P	PRAMEF2	snp	1	12919840	12919840	T	C
MEN0120-P	SELM	snp	22	31503410	31503410	G	A
MEN0120-P	DNAJB12	snp	10	74104883	74104883	G	T
MEN0120-P	POTEH	snp	22	16287770	16287770	C	T
MEN0120-P	SFPQ	snp	1	35656367	35656367	A	G
MEN0120-P	GRIN2B	snp	12	13716759	13716759	C	T
MEN0120-P	CHSY3	snp	5	129521313	129521313	A	T
MEN0120-P	BTNL3	snp	5	180431747	180431747	G	A
MEN0120-P	PLCG2	snp	16	81929442	81929442	C	T
MEN0120-P	NR1H4	snp	12	100904876	100904876	G	A
MEN0120-P	NBPF10	snp	1	145327548	145327548	A	G
MEN0120-P	IL1RN	snp	2	113885296	113885296	C	T
MEN0120-P	MT-ND4	snp	MT	11316	11316	T	C
MEN0120-P	DENND2A	snp	7	140221673	140221673	G	A
MEN0120-P	IYD	snp	6	150715311	150715311	G	A
MEN0120-P	MDK	snp	11	46404331	46404331	C	G
MEN0120-P	MUC21	snp	6	30954806	30954806	T	C
MEN0120-P	HCFC1	snp	X	153219888	153219888	G	A
MEN0120-P	PRMT8	snp	12	3649787	3649787	T	C
MEN0120-P	OR2G6	snp	1	248685648	248685648	G	A
MEN0120-P	ATRX	snp	X	76854964	76854964	A	T
MEN0120-P	SELM	snp	22	31503410	31503410	G	A
MEN0120-P	MDK	snp	11	46404331	46404331	C	G
MEN0120-P	MUC21	snp	6	30954806	30954806	T	C
MEN0120-P	ATXN7L2	snp	1	110032989	110032989	C	T
MEN0120-P	STX16	snp	20	57242559	57242559	G	A
MEN0120-P	DDX55	snp	12	124099851	124099851	G	A
MEN0120-P	HCFC1	snp	X	153219888	153219888	G	A
MEN0120-P	GABBR1	snp	6	29588897	29588897	G	A
MEN0120-P	GABBR1	snp	6	29588897	29588897	G	A
MEN0120-P	SFPQ	snp	1	35656367	35656367	A	G
MEN0120-P	DEFB134	snp	8	11853720	11853720	C	A
MEN0120-P	CHSY3	snp	5	129521313	129521313	A	T
MEN0120-P	PRAMEF2	snp	1	12919840	12919840	T	C
MEN0120-P	OR4P4	snp	11	55406689	55406689	A	G

MEN0120-P	PRAMEF11	snp	1	12887612	12887612	T	C
MEN0120-P	OR2G6	snp	1	248685648	248685648	G	A
MEN0120-P	ADAMTS7	snp	15	79059041	79059041	T	C
MEN0120-P	PRAMEF11	snp	1	12887480	12887480	C	T
MEN0120-P	FAT3	snp	11	92565096	92565096	G	T
MEN0120-P	DENND2A	snp	7	140221673	140221673	G	A
MEN0120-P	IL1RN	snp	2	113885296	113885296	C	T
MEN0120-P	AC109135.1	snp	GL000192.1	311344	311344	C	T
MEN0120-P	PRMT8	snp	12	3649787	3649787	T	C
MEN0120-P	MT-ND4	snp	MT	11316	11316	T	C
MEN0120-P	CHML	snp	1	241797957	241797957	C	A
MEN0120-P	IYD	snp	6	150715311	150715311	G	A
MEN0120-P	ATRX	snp	X	76854964	76854964	A	T
MEN0120-P	LIN7A	snp	12	81283094	81283094	G	T
MEN0120-P	VPS13A	snp	9	79890997	79890997	A	G
MEN0120-P	GRIN2B	snp	12	13716759	13716759	C	T
MEN0120-P	NR1H4	snp	12	100904876	100904876	G	A
MEN0120-P	DNAJB12	snp	10	74104883	74104883	G	T
MEN0120-P	BTNL3	snp	5	180431747	180431747	G	A
MEN0120-P	PLCG2	snp	16	81929442	81929442	C	T
MEN0120-P	SIGLEC8	snp	19	51961502	51961502	C	A
MEN0120-P	NBPF10	snp	1	145327548	145327548	A	G
MEN0120-P	NPHP3	snp	3	132407603	132407603	G	C
MEN0120-P	POTEH	snp	22	16287770	16287770	C	T
MEN0120-P1	VPS13A	snp	9	79890997	79890997	A	G
MEN0120-P1	MT-CYB	snp	MT	15200	15200	G	A
MEN0120-P1	PIM1	snp	6	37140772	37140772	G	A
MEN0120-P1	UNC13C	snp	15	54817829	54817829	G	T
MEN0120-P1	MT-CO2	snp	MT	8019	8019	C	T
MEN0120-P1	CHML	snp	1	241797957	241797957	C	A
MEN0120-P1	XPO5	snp	6	43517248	43517248	C	T
MEN0120-P1	LIPA	snp	10	90974676	90974676	G	A
MEN0120-P1	ATXN7L2	snp	1	110032989	110032989	C	T
MEN0120-P1	OR6K6	snp	1	158725115	158725115	C	G
MEN0120-P1	NPHP3	snp	3	132407603	132407603	G	C
MEN0120-P1	SSTR1	snp	14	38679007	38679007	C	T
MEN0120-P1	ARFGEF1	snp	8	68130340	68130340	C	T
MEN0120-P1	FAT3	snp	11	92565096	92565096	G	T
MEN0120-P1	CHPF	snp	2	220405109	220405109	G	A
MEN0120-P1	ZNF547	snp	19	57888802	57888802	G	A
MEN0120-P1	RRP12	snp	10	99140635	99140635	G	A
MEN0120-P1	DNAJB12	snp	10	74104883	74104883	G	T
MEN0120-P1	GGT7	snp	20	33440271	33440271	C	A
MEN0120-P1	TEX37	snp	2	88828886	88828886	T	C
MEN0120-P1	PRICKLE2	snp	3	64132745	64132745	G	A
MEN0120-P1	GRIN2B	snp	12	13716759	13716759	C	T
MEN0120-P1	ANKRD36C	snp	2	96593000	96593000	A	G

MEN0120-P1	CHSY3	snp	5	129521313	129521313	A	T
MEN0120-P1	BTNL3	snp	5	180431747	180431747	G	A
MEN0120-P1	PLCG2	snp	16	81929442	81929442	C	T
MEN0120-P1	IL1RN	snp	2	113885296	113885296	C	T
MEN0120-P1	TJP3	snp	19	3750145	3750145	C	T
MEN0120-P1	RIN3	snp	14	93118173	93118173	G	A
MEN0120-P1	MT-ND4	snp	MT	11316	11316	T	C
MEN0120-P1	DENND2A	snp	7	140221673	140221673	G	A
MEN0120-P1	COL15A1	snp	9	101824554	101824554	G	A
MEN0120-P1	MYLK	snp	3	123419230	123419230	C	T
MEN0120-P1	GGT7	snp	20	33440271	33440271	C	A
MEN0120-P1	ATXN7L2	snp	1	110032989	110032989	C	T
MEN0120-P1	MT-CYB	snp	MT	15200	15200	G	A
MEN0120-P1	RRP12	snp	10	99140635	99140635	G	A
MEN0120-P1	MT-CO2	snp	MT	8019	8019	C	T
MEN0120-P1	CHSY3	snp	5	129521313	129521313	A	T
MEN0120-P1	TJP3	snp	19	3750145	3750145	C	T
MEN0120-P1	XPO5	snp	6	43517248	43517248	C	T
MEN0120-P1	UNC13C	snp	15	54817829	54817829	G	T
MEN0120-P1	SSTR1	snp	14	38679007	38679007	C	T
MEN0120-P1	LIPA	snp	10	90974676	90974676	G	A
MEN0120-P1	FAT3	snp	11	92565096	92565096	G	T
MEN0120-P1	DENND2A	snp	7	140221673	140221673	G	A
MEN0120-P1	ZNF547	snp	19	57888802	57888802	G	A
MEN0120-P1	IL1RN	snp	2	113885296	113885296	C	T
MEN0120-P1	MT-ND4	snp	MT	11316	11316	T	C
MEN0120-P1	PRICKLE2	snp	3	64132745	64132745	G	A
MEN0120-P1	COL15A1	snp	9	101824554	101824554	G	A
MEN0120-P1	CHML	snp	1	241797957	241797957	C	A
MEN0120-P1	PIM1	snp	6	37140772	37140772	G	A
MEN0120-P1	PIM1	snp	6	37140772	37140772	G	A
MEN0120-P1	TEX37	snp	2	88828886	88828886	T	C
MEN0120-P1	VPS13A	snp	9	79890997	79890997	A	G
MEN0120-P1	MYLK	snp	3	123419230	123419230	C	T
MEN0120-P1	GRIN2B	snp	12	13716759	13716759	C	T
MEN0120-P1	DNAJB12	snp	10	74104883	74104883	G	T
MEN0120-P1	BTNL3	snp	5	180431747	180431747	G	A
MEN0120-P1	OR6K6	snp	1	158725115	158725115	C	G
MEN0120-P1	PLCG2	snp	16	81929442	81929442	C	T
MEN0120-P1	RIN3	snp	14	93118173	93118173	G	A
MEN0120-P1	ARFGEF1	snp	8	68130340	68130340	C	T
MEN0120-P1	ANKRD36C	snp	2	96593000	96593000	A	G
MEN0120-P1	NPHP3	snp	3	132407603	132407603	G	C
MEN0120-P1	CHPF	snp	2	220405109	220405109	G	A
MEN0121-P1	ADCYAP1R1	snp	7	31126128	31126128	A	G
MEN0121-P1	DDT	snp	22	24313793	24313793	G	A
MEN0121-P1	TMEM165	snp	4	56262405	56262405	C	T

MEN0121-P1	NPIPA5	snp	16	15457770	15457770	A	C
MEN0121-P1	JUN	snp	1	59248709	59248709	C	T
MEN0121-P1	ZNF563	snp	19	12429722	12429722	T	C
MEN0121-P1	NBPF14	snp	1	148012529	148012529	T	C
MEN0121-P1	MMP13	snp	11	102815867	102815867	A	G
MEN0121-P1	ZNF91	snp	19	23544783	23544783	C	T
MEN0121-P1	DDT	snp	22	24313792	24313792	G	A
MEN0121-P1	USP17L2	snp	8	11995276	11995276	C	T
MEN0121-P1	NBPF3	snp	1	21806573	21806573	A	G
MEN0121-P1	METTL10	snp	10	126450989	126450989	G	A
MEN0121-P1	IKBKB	snp	8	42173736	42173736	C	T
MEN0121-P1	MYH14	snp	19	50713828	50713828	C	T
MEN0121-P1	IL17RD	snp	3	57132491	57132491	C	T
MEN0121-P1	ZNF878	snp	19	12155757	12155757	C	A
MEN0121-P1	APOB	snp	2	21247929	21247929	G	A
MEN0121-P1	RP11-166B2.1	snp	16	12027558	12027558	T	C
MEN0121-P1	BPIFB3	snp	20	31656748	31656748	G	A
MEN0121-P1	ZNF626	snp	19	20807601	20807601	C	A
MEN0121-P1	ARMC7	snp	17	73124919	73124919	C	T
MEN0121-P1	GOLGA8S	snp	15	23605381	23605381	C	G
MEN0121-P1	NBPF1	snp	1	16892277	16892277	G	A
MEN0121-P1	DMD	snp	X	32827654	32827654	A	T
MEN0121-P1	DSCAM	snp	21	41550989	41550989	C	T
MEN0121-P1	ZNF708	snp	19	21476625	21476625	A	T
MEN0121-P1	MTRNR2L8	snp	11	10529739	10529739	G	A
MEN0121-P1	RP11-166B2.1	snp	16	12027521	12027521	G	C
MEN0121-P1	NBEAL2	snp	3	47037938	47037938	C	G
MEN0121-P1	POU5F1B	snp	8	128428443	128428443	C	T
MEN0121-P1	SLAIN1	snp	13	78320909	78320909	C	A
MEN0121-P1	NBPF10	snp	1	145323695	145323695	C	T
MEN0121-P1	FRY	snp	13	32783812	32783812	T	G
MEN0121-P1	ZNF431	snp	19	21366366	21366366	G	A
MEN0121-P1	ZNF678	snp	1	227843257	227843257	A	G
MEN0121-P1	VCL	snp	10	75865010	75865010	A	G
MEN0121-P1	ZNF721	snp	4	435565	435565	G	T
MEN0121-P1	ZNF729	snp	19	22497191	22497191	T	A
MEN0121-P1	KIAA1324L	snp	7	86542320	86542320	G	T
MEN0121-P1	CAPZA3	snp	12	18891878	18891878	G	T
MEN0121-P1	ZNF728	snp	19	23159304	23159304	C	T
MEN0121-P1	ZNF141	snp	4	367245	367245	C	A
MEN0121-P1	FSTL5	snp	4	162459446	162459446	C	A
MEN0121-P1	ZNF429	snp	19	21719781	21719781	A	G
MEN0121-P1	ZNF728	snp	19	23159288	23159288	T	C
MEN0121-P1	ARPP21	snp	3	35763297	35763297	C	T
MEN0121-P1	ZNF254	snp	19	24310154	24310154	A	C
MEN0121-P1	TICAM1	snp	19	4817402	4817402	C	T
MEN0121-P1	ZNF91	snp	19	23542561	23542561	T	C

MEN0121-P1	WDR65	snp	1	43675591	43675591	A	G
MEN0121-P1	RAB21	snp	12	72179430	72179430	G	A
MEN0121-P1	ZNF208	snp	19	22154249	22154249	A	G
MEN0121-P1	ZNF431	snp	19	21366365	21366365	T	G
MEN0121-P1	ZNF208	snp	19	22156362	22156362	C	T
MEN0121-P1	ZNF724P	snp	19	23405270	23405270	T	C
MEN0121-P1	NCOA4	snp	10	51573319	51573319	G	A
MEN0121-P1	ZNF257	snp	19	22271309	22271309	A	G
MEN0121-P1	ZNF729	snp	19	22497504	22497504	A	G
MEN0121-P1	ZNF208	snp	19	22156983	22156983	T	A
MEN0121-P1	ZNF90	snp	19	20229964	20229964	C	A
MEN0121-P1	KIAA1324L	snp	7	86542320	86542320	G	T
MEN0121-P1	ARMC7	snp	17	73124919	73124919	C	T
MEN0121-P1	DDT	snp	22	24313792	24313792	G	A
MEN0121-P1	FRY	snp	13	32783812	32783812	T	G
MEN0121-P1	IL17RD	snp	3	57132491	57132491	C	T
MEN0121-P1	RP11-166B2.1	snp	16	12027521	12027521	G	C
MEN0121-P1	ZNF729	snp	19	22497191	22497191	T	A
MEN0121-P1	TMEM165	snp	4	56262405	56262405	C	T
MEN0121-P1	CAPZA3	snp	12	18891878	18891878	G	T
MEN0121-P1	ZNF721	snp	4	435565	435565	G	T
MEN0121-P1	APOB	snp	2	21247929	21247929	G	A
MEN0121-P1	ZNF626	snp	19	20807601	20807601	C	A
MEN0121-P1	ZNF729	snp	19	22497504	22497504	A	G
MEN0121-P1	WDR65	snp	1	43675591	43675591	A	G
MEN0121-P1	ZNF254	snp	19	24310154	24310154	A	C
MEN0121-P1	METTL10	snp	10	126450989	126450989	G	A
MEN0121-P1	NBEAL2	snp	3	47037938	47037938	C	G
MEN0121-P1	MTRNR2L8	snp	11	10529739	10529739	G	A
MEN0121-P1	ZNF563	snp	19	12429722	12429722	T	C
MEN0121-P1	FSTL5	snp	4	162459446	162459446	C	A
MEN0121-P1	ZNF708	snp	19	21476625	21476625	A	T
MEN0121-P1	MMP13	snp	11	102815867	102815867	A	G
MEN0121-P1	ZNF90	snp	19	20229964	20229964	C	A
MEN0121-P1	ARPP21	snp	3	35763297	35763297	C	T
MEN0121-P1	ZNF91	snp	19	23544783	23544783	C	T
MEN0121-P1	IKBKB	snp	8	42173736	42173736	C	T
MEN0121-P1	NBPF14	snp	1	148012529	148012529	T	C
MEN0121-P1	GOLGA8S	snp	15	23605381	23605381	C	G
MEN0121-P1	ZNF208	snp	19	22154249	22154249	A	G
MEN0121-P1	ZNF429	snp	19	21719781	21719781	A	G
MEN0121-P1	TICAM1	snp	19	4817402	4817402	C	T
MEN0121-P1	NPIPA5	snp	16	15457770	15457770	A	C
MEN0121-P1	BPIFB3	snp	20	31656748	31656748	G	A
MEN0121-P1	RP11-166B2.1	snp	16	12027558	12027558	T	C
MEN0121-P1	USP17L2	snp	8	11995276	11995276	C	T
MEN0121-P1	ZNF431	snp	19	21366365	21366365	T	G

MEN0121-P1	JUN	snp	1	59248709	59248709	C	T
MEN0121-P1	MYH14	snp	19	50713828	50713828	C	T
MEN0121-P1	ZNF678	snp	1	227843257	227843257	A	G
MEN0121-P1	DMD	snp	X	32827654	32827654	A	T
MEN0121-P1	MTRNR2L2	snp	5	79945871	79945871	G	A
MEN0121-P1	ZNF208	snp	19	22156983	22156983	T	A
MEN0121-P1	ZNF878	snp	19	12155757	12155757	C	A
MEN0121-P1	ADCYAP1R1	snp	7	31126128	31126128	A	G
MEN0121-P1	VCL	snp	10	75865010	75865010	A	G
MEN0121-P1	POU5F1B	snp	8	128428443	128428443	C	T
MEN0121-P1	ZNF257	snp	19	22271309	22271309	A	G
MEN0121-P1	ZNF91	snp	19	23542561	23542561	T	C
MEN0121-P1	ZNF728	snp	19	23159288	23159288	T	C
MEN0121-P1	DSCAM	snp	21	41550989	41550989	C	T
MEN0121-P1	ZNF728	snp	19	23159304	23159304	C	T
MEN0121-P1	NBPF1	snp	1	16892277	16892277	G	A
MEN0121-P1	ZNF724P	snp	19	23405270	23405270	T	C
MEN0121-P1	ZNF141	snp	4	367245	367245	C	A
MEN0121-P1	MTRNR2L1	snp	17	22023422	22023422	C	T
MEN0121-P1	SLAIN1	snp	13	78320909	78320909	C	A
MEN0121-P1	NBPF10	snp	1	145323695	145323695	C	T
MEN0121-P1	NCOA4	snp	10	51573319	51573319	G	A
MEN0121-P1	NBPF3	snp	1	21806573	21806573	A	G
MEN0121-P1	RAB21	snp	12	72179430	72179430	G	A
MEN0121-P1	ZNF208	snp	19	22156362	22156362	C	T
MEN0121-P2	ZNF208	snp	19	22155896	22155896	A	C
MEN0121-P2	FAT1	snp	4	187540099	187540099	C	A
MEN0121-P2	ADCYAP1R1	snp	7	31126128	31126128	A	G
MEN0121-P2	TMEM165	snp	4	56262405	56262405	C	T
MEN0121-P2	JUN	snp	1	59248709	59248709	C	T
MEN0121-P2	NBPF3	snp	1	21806573	21806573	A	G
MEN0121-P2	IKBKB	snp	8	42173736	42173736	C	T
MEN0121-P2	AOC1	snp	7	150554087	150554087	T	A
MEN0121-P2	IL17RD	snp	3	57132491	57132491	C	T
MEN0121-P2	APOB	snp	2	21247929	21247929	G	A
MEN0121-P2	GRM5	snp	11	88780856	88780856	T	A
MEN0121-P2	ZNF844	snp	19	12187275	12187275	G	C
MEN0121-P2	ARMC7	snp	17	73124919	73124919	C	T
MEN0121-P2	NBPF3	snp	1	21806606	21806606	T	G
MEN0121-P2	PDE4DIP	snp	1	144994680	144994680	C	G
MEN0121-P2	NBPF1	snp	1	16892277	16892277	G	A
MEN0121-P2	DSCAM	snp	21	41550989	41550989	C	T
MEN0121-P2	POU5F1B	snp	8	128428443	128428443	C	T
MEN0121-P2	EPPIN	snp	20	44171423	44171423	A	G
MEN0121-P2	SLAIN1	snp	13	78320909	78320909	C	A
MEN0121-P2	KMT2C	snp	7	151932922	151932922	C	T
MEN0121-P2	FRY	snp	13	32783812	32783812	T	G

MEN0121-P2	ZNF724P	snp	19	23405522	23405522	T	C
MEN0121-P2	VCL	snp	10	75865010	75865010	A	G
MEN0121-P2	ZNF208	snp	19	22156590	22156590	C	T
MEN0121-P2	ZNF93	snp	19	20044524	20044524	A	G
MEN0121-P2	TYW1	snp	7	66548448	66548448	C	T
MEN0121-P2	POLR1C	snp	6	43497019	43497019	A	G
MEN0121-P2	ZNF737	snp	19	20728407	20728407	A	T
MEN0121-P2	ARPP21	snp	3	35763297	35763297	C	T
MEN0121-P2	PSTPIP1	snp	15	77300212	77300212	A	G
MEN0121-P2	TICAM1	snp	19	4817402	4817402	C	T
MEN0121-P2	WDR65	snp	1	43675591	43675591	A	G
MEN0121-P2	ZNF90	snp	19	20229884	20229884	T	A
MEN0121-P2	ZNF729	snp	19	22498260	22498260	A	G
MEN0121-P2	FBXO31	snp	16	87417161	87417161	G	A
MEN0121-P2	ZNF235	snp	19	44792890	44792890	T	C
MEN0121-P2	ARMC7	snp	17	73124919	73124919	C	T
MEN0121-P2	ZNF208	snp	19	22156590	22156590	C	T
MEN0121-P2	GRM5	snp	11	88780856	88780856	T	A
MEN0121-P2	ZNF729	snp	19	22498260	22498260	A	G
MEN0121-P2	FRY	snp	13	32783812	32783812	T	G
MEN0121-P2	IL17RD	snp	3	57132491	57132491	C	T
MEN0121-P2	ZNF844	snp	19	12187275	12187275	G	C
MEN0121-P2	FBXO31	snp	16	87417161	87417161	G	A
MEN0121-P2	TMEM165	snp	4	56262405	56262405	C	T
MEN0121-P2	APOB	snp	2	21247929	21247929	G	A
MEN0121-P2	PDE4DIP	snp	1	144994680	144994680	C	G
MEN0121-P2	ZNF208	snp	19	22155896	22155896	A	C
MEN0121-P2	WDR65	snp	1	43675591	43675591	A	G
MEN0121-P2	KMT2C	snp	7	151932922	151932922	C	T
MEN0121-P2	PSTPIP1	snp	15	77300212	77300212	A	G
MEN0121-P2	ARPP21	snp	3	35763297	35763297	C	T
MEN0121-P2	POLR1C	snp	6	43497019	43497019	A	G
MEN0121-P2	IKBKB	snp	8	42173736	42173736	C	T
MEN0121-P2	TICAM1	snp	19	4817402	4817402	C	T
MEN0121-P2	AOC1	snp	7	150554087	150554087	T	A
MEN0121-P2	ZNF235	snp	19	44792890	44792890	T	C
MEN0121-P2	FAT1	snp	4	187540099	187540099	C	A
MEN0121-P2	JUN	snp	1	59248709	59248709	C	T
MEN0121-P2	ZNF724P	snp	19	23405522	23405522	T	C
MEN0121-P2	ADCYAP1R1	snp	7	31126128	31126128	A	G
MEN0121-P2	TYW1	snp	7	66548448	66548448	C	T
MEN0121-P2	VCL	snp	10	75865010	75865010	A	G
MEN0121-P2	POU5F1B	snp	8	128428443	128428443	C	T
MEN0121-P2	ZNF90	snp	19	20229884	20229884	T	A
MEN0121-P2	DSCAM	snp	21	41550989	41550989	C	T
MEN0121-P2	ZNF93	snp	19	20044524	20044524	A	G
MEN0121-P2	NBPF1	snp	1	16892277	16892277	G	A

MEN0121-P2	ZNF737	snp	19	20728407	20728407	A	T
MEN0121-P2	NBPF3	snp	1	21806606	21806606	T	G
MEN0121-P2	SLAIN1	snp	13	78320909	78320909	C	A
MEN0121-P2	EPPIN	snp	20	44171423	44171423	A	G
MEN0121-P2	NBPF3	snp	1	21806573	21806573	A	G
MEN0121-P3	ULK4	snp	3	41939929	41939929	T	A
MEN0121-P3	CYP2C9	snp	10	96708931	96708931	G	T
MEN0121-P3	OR6N1	snp	1	158736390	158736390	A	G
MEN0121-P3	ADCYAP1R1	snp	7	31126128	31126128	A	G
MEN0121-P3	ARHGAP21	snp	10	24896527	24896527	G	A
MEN0121-P3	ZNF777	snp	7	149129698	149129698	G	C
MEN0121-P3	BDH2	snp	4	104006545	104006545	A	C
MEN0121-P3	TPRG1L	snp	1	3545097	3545097	G	C
MEN0121-P3	LMO7	snp	13	76408406	76408406	A	T
MEN0121-P3	SPIC	snp	12	101880180	101880180	G	T
MEN0121-P3	ULK4	snp	3	41939933	41939933	T	C
MEN0121-P3	RAP1B	snp	12	69050902	69050902	C	G
MEN0121-P3	TTN	snp	2	179482678	179482678	C	A
MEN0121-P3	IL17RD	snp	3	57132491	57132491	C	T
MEN0121-P3	APOB	snp	2	21247929	21247929	G	A
MEN0121-P3	UGT2B10	snp	4	69683758	69683758	A	T
MEN0121-P3	C11orf82	snp	11	82643984	82643984	C	T
MEN0121-P3	NIN	snp	14	51221546	51221546	C	A
MEN0121-P3	WDR72	snp	15	53907889	53907889	A	T
MEN0121-P3	QARS	snp	3	49140143	49140143	C	G
MEN0121-P3	FSIP2	snp	2	186669313	186669313	A	C
MEN0121-P3	ROS1	snp	6	117710556	117710556	C	A
MEN0121-P3	RNF213	snp	17	78286933	78286933	C	T
MEN0121-P3	ZNF678	snp	1	227842511	227842511	A	G
MEN0121-P3	ANKRD55	snp	5	55472088	55472088	G	A
MEN0121-P3	IGFL3	snp	19	46627402	46627402	C	A
MEN0121-P3	POU5F1B	snp	8	128428443	128428443	C	T
MEN0121-P3	FBXO38	snp	5	147813239	147813239	G	C
MEN0121-P3	COG1	snp	17	71193091	71193091	G	C
MEN0121-P3	NELL2	snp	12	45004687	45004687	C	A
MEN0121-P3	COG1	snp	17	71193469	71193469	G	C
MEN0121-P3	DNAH11	snp	7	21778423	21778423	G	A
MEN0121-P3	OR5M1	snp	11	56380959	56380959	G	A
MEN0121-P3	COG1	snp	17	71193063	71193063	G	C
MEN0121-P3	ARPP21	snp	3	35763297	35763297	C	T
MEN0121-P3	ANKRD17	snp	4	73957695	73957695	C	A
MEN0121-P3	WDR65	snp	1	43675591	43675591	A	G
MEN0121-P3	MAEL	snp	1	166958594	166958594	C	T
MEN0121-P3	COLGALT2	snp	1	184006433	184006433	A	T
MEN0121-P3	FAM155A	snp	13	108518715	108518715	C	T
MEN0121-P3	ULK4	snp	3	41939929	41939929	T	A
MEN0121-P3	COLGALT2	snp	1	184006433	184006433	A	T

MEN0121-P3	CYP2C9	snp	10	96708931	96708931	G	T
MEN0121-P3	RNF213	snp	17	78286933	78286933	C	T
MEN0121-P3	ZNF777	snp	7	149129698	149129698	G	C
MEN0121-P3	C11orf82	snp	11	82643984	82643984	C	T
MEN0121-P3	FBXO38	snp	5	147813239	147813239	G	C
MEN0121-P3	COG1	snp	17	71193063	71193063	G	C
MEN0121-P3	IL17RD	snp	3	57132491	57132491	C	T
MEN0121-P3	TPRG1L	snp	1	3545097	3545097	G	C
MEN0121-P3	ROS1	snp	6	117710556	117710556	C	A
MEN0121-P3	FSIP2	snp	2	186669313	186669313	A	C
MEN0121-P3	APOB	snp	2	21247929	21247929	G	A
MEN0121-P3	BDH2	snp	4	104006545	104006545	A	C
MEN0121-P3	OR6N1	snp	1	158736390	158736390	A	G
MEN0121-P3	ANKRD55	snp	5	55472088	55472088	G	A
MEN0121-P3	LMO7	snp	13	76408406	76408406	A	T
MEN0121-P3	WDR65	snp	1	43675591	43675591	A	G
MEN0121-P3	COG1	snp	17	71193469	71193469	G	C
MEN0121-P3	ARHGAP21	snp	10	24896527	24896527	G	A
MEN0121-P3	DNAH11	snp	7	21778423	21778423	G	A
MEN0121-P3	ANKRD17	snp	4	73957695	73957695	C	A
MEN0121-P3	ARPP21	snp	3	35763297	35763297	C	T
MEN0121-P3	WDR72	snp	15	53907889	53907889	A	T
MEN0121-P3	RAP1B	snp	12	69050902	69050902	C	G
MEN0121-P3	COG1	snp	17	71193091	71193091	G	C
MEN0121-P3	TTN	snp	2	179482678	179482678	C	A
MEN0121-P3	UGT2B10	snp	4	69683758	69683758	A	T
MEN0121-P3	IGFL3	snp	19	46627402	46627402	C	A
MEN0121-P3	QARS	snp	3	49140143	49140143	C	G
MEN0121-P3	OR5M1	snp	11	56380959	56380959	G	A
MEN0121-P3	ZNF678	snp	1	227842511	227842511	A	G
MEN0121-P3	NIN	snp	14	51221546	51221546	C	A
MEN0121-P3	MAEL	snp	1	166958594	166958594	C	T
MEN0121-P3	NELL2	snp	12	45004687	45004687	C	A
MEN0121-P3	ADCYAP1R1	snp	7	31126128	31126128	A	G
MEN0121-P3	SPIC	snp	12	101880180	101880180	G	T
MEN0121-P3	POU5F1B	snp	8	128428443	128428443	C	T
MEN0121-P3	FAM155A	snp	13	108518715	108518715	C	T
MEN0121-P4	ADCYAP1R1	snp	7	31126128	31126128	A	G
MEN0121-P4	TMEM165	snp	4	56262405	56262405	C	T
MEN0121-P4	JUN	snp	1	59248709	59248709	C	T
MEN0121-P4	DNAAF3	snp	19	55677738	55677738	G	T
MEN0121-P4	HECW2	snp	2	197085580	197085580	T	C
MEN0121-P4	KIAA1324L	snp	7	86574279	86574279	T	C
MEN0121-P4	IKBKB	snp	8	42173736	42173736	C	T
MEN0121-P4	IL17RD	snp	3	57132491	57132491	C	T
MEN0121-P4	APOB	snp	2	21247929	21247929	G	A
MEN0121-P4	GBP7	snp	1	89607325	89607325	C	T

MEN0121-P4	ARMC7	snp	17	73124919	73124919	C	T
MEN0121-P4	PCDHGA2	snp	5	140720207	140720207	G	A
MEN0121-P4	DSCAM	snp	21	41550989	41550989	C	T
MEN0121-P4	POU5F1B	snp	8	128428443	128428443	C	T
MEN0121-P4	SLAIN1	snp	13	78320909	78320909	C	A
MEN0121-P4	ZNF594	snp	17	5085547	5085547	T	C
MEN0121-P4	FRY	snp	13	32783812	32783812	T	G
MEN0121-P4	VCL	snp	10	75865010	75865010	A	G
MEN0121-P4	ZNF792	snp	19	35448889	35448889	G	A
MEN0121-P4	MCF2	snp	X	138667305	138667305	A	G
MEN0121-P4	ARHGAP31	snp	3	119135085	119135085	G	A
MEN0121-P4	AASS	snp	7	121773582	121773582	T	A
MEN0121-P4	ANKRD30A	snp	10	37433929	37433929	G	T
MEN0121-P4	ARPP21	snp	3	35763297	35763297	C	T
MEN0121-P4	TICAM1	snp	19	4817402	4817402	C	T
MEN0121-P4	DLC1	snp	8	12957624	12957624	C	G
MEN0121-P4	SYTL2	snp	11	85445592	85445592	C	G
MEN0121-P4	WDR65	snp	1	43675591	43675591	A	G
MEN0121-P4	ALOX15	snp	17	4542725	4542725	C	T
MEN0121-P4	AC012313.1	snp	19	58907941	58907941	C	A
MEN0121-P4	ARMC7	snp	17	73124919	73124919	C	T
MEN0121-P4	KIAA1324L	snp	7	86574279	86574279	T	C
MEN0121-P4	HECW2	snp	2	197085580	197085580	T	C
MEN0121-P4	AASS	snp	7	121773582	121773582	T	A
MEN0121-P4	FRY	snp	13	32783812	32783812	T	G
MEN0121-P4	IL17RD	snp	3	57132491	57132491	C	T
MEN0121-P4	TMEM165	snp	4	56262405	56262405	C	T
MEN0121-P4	APOB	snp	2	21247929	21247929	G	A
MEN0121-P4	ZNF792	snp	19	35448889	35448889	G	A
MEN0121-P4	GBP7	snp	1	89607325	89607325	C	T
MEN0121-P4	WDR65	snp	1	43675591	43675591	A	G
MEN0121-P4	DNAAF3	snp	19	55677738	55677738	G	T
MEN0121-P4	AC012313.1	snp	19	58907941	58907941	C	A
MEN0121-P4	ZNF594	snp	17	5085547	5085547	T	C
MEN0121-P4	ARPP21	snp	3	35763297	35763297	C	T
MEN0121-P4	ARHGAP31	snp	3	119135085	119135085	G	A
MEN0121-P4	IKBKB	snp	8	42173736	42173736	C	T
MEN0121-P4	TICAM1	snp	19	4817402	4817402	C	T
MEN0121-P4	JUN	snp	1	59248709	59248709	C	T
MEN0121-P4	ADCYAP1R1	snp	7	31126128	31126128	A	G
MEN0121-P4	SYTL2	snp	11	85445592	85445592	C	G
MEN0121-P4	VCL	snp	10	75865010	75865010	A	G
MEN0121-P4	PCDHGA2	snp	5	140720207	140720207	G	A
MEN0121-P4	POU5F1B	snp	8	128428443	128428443	C	T
MEN0121-P4	MCF2	snp	X	138667305	138667305	A	G
MEN0121-P4	DSCAM	snp	21	41550989	41550989	C	T
MEN0121-P4	DLC1	snp	8	12957624	12957624	C	G

MEN0121-P4	ALOX15	snp	17	4542725	4542725	C	T
MEN0121-P4	SLAIN1	snp	13	78320909	78320909	C	A
MEN0121-P4	ANKRD30A	snp	10	37433929	37433929	G	T
MEN0122-RBM-P1	ZDHHC15	snp	X	74742756	74742756	G	A
MEN0122-RBM-P1	LRRC37A3	snp	17	62856239	62856239	A	G
MEN0122-RBM-P1	ZNF253	snp	19	20002995	20002995	C	A
MEN0122-RBM-P1	NPIPA5	snp	16	15457638	15457638	A	G
MEN0122-RBM-P1	AC092964.1	snp	3	172039500	172039500	A	T
MEN0122-RBM-P1	ZNF99	snp	19	22941531	22941531	G	T
MEN0122-RBM-P1	SUMO1	snp	2	203096321	203096321	A	C
MEN0122-RBM-P1	ZNF721	snp	4	437339	437339	G	T
MEN0122-RBM-P1	LPA	snp	6	161026093	161026093	G	T
MEN0122-RBM-P1	ZNF385B	snp	2	180308170	180308170	A	G
MEN0122-RBM-P1	ZNF195	snp	11	3380694	3380694	G	A
MEN0122-RBM-P1	ANKRD36	snp	2	97875427	97875427	G	A
MEN0122-RBM-P1	ZNF138	snp	7	64292354	64292354	G	A
MEN0122-RBM-P1	ZNF257	snp	19	22271655	22271655	A	G
MEN0122-RBM-P1	NPIPA1	snp	16	15045638	15045638	T	A
MEN0122-RBM-P1	ZNF729	snp	19	22499682	22499682	T	A
MEN0122-RBM-P1	CTB-134H23.2	snp	16	29063558	29063558	C	G
MEN0122-RBM-P1	CTAGE5	snp	14	39746243	39746243	T	C
MEN0122-RBM-P1	TMPRSS12	snp	12	51279241	51279241	G	T
MEN0122-RBM-P1	CDC27	snp	17	45234359	45234359	T	A
MEN0122-RBM-P1	PAN3	snp	13	28841340	28841340	C	T
MEN0122-RBM-P1	PRB4	snp	12	11461672	11461672	G	C
MEN0122-RBM-P1	ZNF708	snp	19	21476519	21476519	T	C
MEN0122-RBM-P1	NUP153	snp	6	17652073	17652073	T	C
MEN0122-RBM-P1	ZNF724P	snp	19	23406281	23406281	G	A
MEN0122-RBM-P1	ZNF732	snp	4	265139	265139	C	T
MEN0122-RBM-P1	ANKRD36	snp	2	97875432	97875432	A	C
MEN0122-RBM-P1	ZNF93	snp	19	20045338	20045338	T	C
MEN0122-RBM-P1	FAM47C	snp	X	37027815	37027815	G	T
MEN0122-RBM-P1	KHDRBS3	snp	8	136594267	136594267	G	T
MEN0122-RBM-P1	ZNF841	snp	19	52569771	52569771	G	T
MEN0122-RBM-P1	TPRXL	snp	3	14106310	14106310	T	C
MEN0122-RBM-P1	ZNF257	snp	19	22271654	22271654	A	G
MEN0122-RBM-P1	ADM2	snp	22	50921449	50921449	C	T
MEN0122-RBM-P1	ADAMTSL3	snp	15	84611798	84611798	C	A
MEN0122-RBM-P1	ZNF257	snp	19	22256329	22256329	T	G
MEN0122-RBM-P1	MUC4	snp	3	195506638	195506638	G	T
MEN0122-RBM-P1	ZNF486	snp	19	20307959	20307959	C	A
MEN0122-RBM-P1	ZNF681	snp	19	23927139	23927139	T	G
MEN0122-RBM-P1	BFSP1	snp	20	17475524	17475524	T	C
MEN0122-RBM-P1	FLG2	snp	1	152323303	152323303	G	C
MEN0122-RBM-P1	ACTR6	snp	12	100602573	100602573	A	G
MEN0122-RBM-P1	ZNF728	snp	19	23159220	23159220	C	T
MEN0122-RBM-P1	ZNF107	snp	7	64168240	64168240	A	G

MEN0122-RBM-P1	G3BP1	snp	5	151170505	151170505	G	A
MEN0122-RBM-P1	ADM2	snp	22	50921652	50921652	C	T
MEN0122-RBM-P1	ZNF90	snp	19	20229706	20229706	G	C
MEN0122-RBM-P1	ARHGAP8	snp	22	45204596	45204596	T	C
MEN0122-RBM-P1	CTAGE5	snp	14	39746235	39746235	C	T
MEN0122-RBM-P1	PDLIM5	snp	4	95377618	95377618	A	G
MEN0122-RBM-P1	CDHR5	snp	11	618674	618674	C	T
MEN0122-RBM-P1	NPIPA5	snp	16	15457656	15457656	G	T
MEN0122-RBM-P1	CSMD3	snp	8	113662572	113662572	G	T
MEN0122-RBM-P1	ZNF431	snp	19	21366366	21366366	G	A
MEN0122-RBM-P1	NBPF3	snp	1	21804692	21804692	G	T
MEN0122-RBM-P1	EN1	snp	2	119604523	119604523	A	G
MEN0122-RBM-P1	ZNF729	snp	19	22499016	22499016	A	G
MEN0122-RBM-P1	ZNF845	snp	19	53856360	53856360	A	C
MEN0122-RBM-P1	SLC7A8	snp	14	23607157	23607157	A	G
MEN0122-RBM-P1	ZNF737	snp	19	20727966	20727966	C	T
MEN0122-RBM-P1	ZNF678	snp	1	227843138	227843138	G	A
MEN0122-RBM-P1	IGHV3-11	snp	14	106573490	106573490	T	G
MEN0122-RBM-P1	ZNF93	snp	19	20045017	20045017	G	T
MEN0122-RBM-P1	ZNF626	snp	19	20807415	20807415	C	T
MEN0122-RBM-P1	ZNF729	snp	19	22497318	22497318	G	A
MEN0122-RBM-P1	NPIPB15	snp	16	74415479	74415479	C	G
MEN0122-RBM-P1	ELMO1	snp	7	37243809	37243809	T	C
MEN0122-RBM-P1	RP11-467N20.5	snp	15	23407305	23407305	T	C
MEN0122-RBM-P1	ZNF727	snp	7	63538372	63538372	T	A
MEN0122-RBM-P1	ZNF492	snp	19	22847878	22847878	G	A
MEN0122-RBM-P1	USP17L2	snp	8	11995002	11995002	C	T
MEN0122-RBM-P1	ZNF141	snp	4	367336	367336	T	A
MEN0122-RBM-P1	HRNR	snp	1	152191890	152191890	T	C
MEN0122-RBM-P1	ZNF736	snp	7	63809266	63809266	T	A
MEN0122-RBM-P1	ZNF85	snp	19	21132356	21132356	A	G
MEN0122-RBM-P1	ZNF678	snp	1	227842419	227842419	C	A
MEN0122-RBM-P1	UBE2I	snp	16	1374783	1374783	G	T
MEN0122-RBM-P1	ZNF678	snp	1	227843156	227843156	T	A
MEN0122-RBM-P1	KATNBL1	snp	15	34434556	34434556	A	G
MEN0122-RBM-P1	ZNF66	snp	19	20989766	20989766	G	A
MEN0122-RBM-P1	ZNF732	snp	4	265500	265500	A	T
MEN0122-RBM-P1	ZNF208	snp	19	22155518	22155518	A	C
MEN0122-RBM-P1	ZNF91	snp	19	23544222	23544222	A	C
MEN0122-RBM-P1	ZNF678	snp	1	227842915	227842915	C	A
MEN0122-RBM-P1	ZNF729	snp	19	22499446	22499446	G	A
MEN0122-RBM-P1	ZNF729	snp	19	22497267	22497267	C	T
MEN0122-RBM-P1	PBX4	snp	19	19681631	19681631	G	A
MEN0122-RBM-P1	ZNF729	snp	19	22497504	22497504	A	G
MEN0122-RBM-P1	ZNF253	snp	19	20002885	20002885	G	A
MEN0122-RBM-P1	ZNF43	snp	19	21990645	21990645	G	T
MEN0122-RBM-P1	ZNF43	snp	19	21990711	21990711	T	C

MEN0122-RBM-P1	ZNF43	snp	19	21991811	21991811	G	T
MEN0122-RBM-P1	ZNF208	snp	19	22157070	22157070	A	G
MEN0122-RBM-P1	CTAGE5	snp	14	39746235	39746235	C	T
MEN0122-RBM-P1	ZNF737	snp	19	20727966	20727966	C	T
MEN0122-RBM-P1	ZDHHC15	snp	X	74742756	74742756	G	A
MEN0122-RBM-P1	ZNF845	snp	19	53856360	53856360	A	C
MEN0122-RBM-P1	NUP153	snp	6	17652073	17652073	T	C
MEN0122-RBM-P1	ACTR6	snp	12	100602573	100602573	A	G
MEN0122-RBM-P1	LRRC37A3	snp	17	62856239	62856239	A	G
MEN0122-RBM-P1	TMPRSS12	snp	12	51279241	51279241	G	T
MEN0122-RBM-P1	SUMO1	snp	2	203096321	203096321	A	C
MEN0122-RBM-P1	ZNF385B	snp	2	180308170	180308170	A	G
MEN0122-RBM-P1	ZNF727	snp	7	63538372	63538372	T	A
MEN0122-RBM-P1	ZNF681	snp	19	23927139	23927139	T	G
MEN0122-RBM-P1	ELMO1	snp	7	37243809	37243809	T	C
MEN0122-RBM-P1	ZNF93	snp	19	20045017	20045017	G	T
MEN0122-RBM-P1	IGHV3-11	snp	14	106573490	106573490	T	G
MEN0122-RBM-P1	PAN3	snp	13	28841340	28841340	C	T
MEN0122-RBM-P1	SLC7A8	snp	14	23607157	23607157	A	G
MEN0122-RBM-P1	ADM2	snp	22	50921449	50921449	C	T
MEN0122-RBM-P1	PDLIM5	snp	4	95377618	95377618	A	G
MEN0122-RBM-P1	ZNF729	snp	19	22499682	22499682	T	A
MEN0122-RBM-P1	BFSP1	snp	20	17475524	17475524	T	C
MEN0122-RBM-P1	ZNF841	snp	19	52569771	52569771	G	T
MEN0122-RBM-P1	ARHGAP8	snp	22	45204596	45204596	T	C
MEN0122-RBM-P1	ZNF107	snp	7	64168240	64168240	A	G
MEN0122-RBM-P1	ZNF678	snp	1	227843138	227843138	G	A
MEN0122-RBM-P1	CSMD3	snp	8	113662572	113662572	G	T
MEN0122-RBM-P1	MUC4	snp	3	195506638	195506638	G	T
MEN0122-RBM-P1	ZNF138	snp	7	64292354	64292354	G	A
MEN0122-RBM-P1	ZNF486	snp	19	20307959	20307959	C	A
MEN0122-RBM-P1	PBX4	snp	19	19681631	19681631	G	A
MEN0122-RBM-P1	CDC27	snp	17	45234359	45234359	T	A
MEN0122-RBM-P1	ZNF732	snp	4	265500	265500	A	T
MEN0122-RBM-P1	ZNF729	snp	19	22499016	22499016	A	G
MEN0122-RBM-P1	ZNF728	snp	19	23159220	23159220	C	T
MEN0122-RBM-P1	ZNF208	snp	19	22155518	22155518	A	C
MEN0122-RBM-P1	ZNF729	snp	19	22497267	22497267	C	T
MEN0122-RBM-P1	KATNBL1	snp	15	34434556	34434556	A	G
MEN0122-RBM-P1	ZNF708	snp	19	21476519	21476519	T	C
MEN0122-RBM-P1	ZNF85	snp	19	21132356	21132356	A	G
MEN0122-RBM-P1	ZNF90	snp	19	20229706	20229706	G	C
MEN0122-RBM-P1	ZNF729	snp	19	22497318	22497318	G	A
MEN0122-RBM-P1	ZNF729	snp	19	22497504	22497504	A	G
MEN0122-RBM-P1	NBPF3	snp	1	21804692	21804692	G	T
MEN0122-RBM-P1	ZNF99	snp	19	22941531	22941531	G	T
MEN0122-RBM-P1	ZNF195	snp	11	3380694	3380694	G	A

MEN0122-RBM-P1	G3BP1	snp	5	151170505	151170505	G	A
MEN0122-RBM-P1	ZNF43	snp	19	21990711	21990711	T	C
MEN0122-RBM-P1	ZNF43	snp	19	21990645	21990645	G	T
MEN0122-RBM-P1	FLG2	snp	1	152323303	152323303	G	C
MEN0122-RBM-P1	ZNF678	snp	1	227842419	227842419	C	A
MEN0122-RBM-P1	ZNF724P	snp	19	23406281	23406281	G	A
MEN0122-RBM-P1	ZNF257	snp	19	22271654	22271654	A	G
MEN0122-RBM-P1	NPIPA1	snp	16	15045638	15045638	T	A
MEN0122-RBM-P1	LPA	snp	6	161026093	161026093	G	T
MEN0122-RBM-P1	ZNF208	snp	19	22157070	22157070	A	G
MEN0122-RBM-P1	ZNF93	snp	19	20045338	20045338	T	C
MEN0122-RBM-P1	ZNF431	snp	19	21366366	21366366	G	A
MEN0122-RBM-P1	ZNF492	snp	19	22847878	22847878	G	A
MEN0122-RBM-P1	ZNF91	snp	19	23544222	23544222	A	C
MEN0122-RBM-P1	ZNF721	snp	4	437339	437339	G	T
MEN0122-RBM-P1	ANKRD36	snp	2	97875427	97875427	G	A
MEN0122-RBM-P1	ZNF257	snp	19	22256329	22256329	T	G
MEN0122-RBM-P1	ZNF253	snp	19	20002885	20002885	G	A
MEN0122-RBM-P1	ZNF141	snp	4	367336	367336	T	A
MEN0122-RBM-P1	UBE2I	snp	16	1374783	1374783	G	T
MEN0122-RBM-P1	USP17L2	snp	8	11995002	11995002	C	T
MEN0122-RBM-P1	HRNR	snp	1	152191890	152191890	T	C
MEN0122-RBM-P1	NPIPA5	snp	16	15457638	15457638	A	G
MEN0122-RBM-P1	ZNF732	snp	4	265139	265139	C	T
MEN0122-RBM-P1	NPIPB15	snp	16	74415479	74415479	C	G
MEN0122-RBM-P1	ZNF66	snp	19	20989766	20989766	G	A
MEN0122-RBM-P1	CTB-134H23.2	snp	16	29063558	29063558	C	G
MEN0122-RBM-P1	ZNF728	snp	19	23158787	23158787	T	G
MEN0122-RBM-P1	ZNF626	snp	19	20807415	20807415	C	T
MEN0122-RBM-P1	ZNF43	snp	19	21991811	21991811	G	T
MEN0122-RBM-P1	ZNF678	snp	1	227842915	227842915	C	A
MEN0122-RBM-P1	ZNF736	snp	7	63809266	63809266	T	A
MEN0122-RBM-P1	ZNF729	snp	19	22499446	22499446	G	A
MEN0122-RBM-P1	ZNF253	snp	19	20002995	20002995	C	A
MEN0122-RBM-P1	KHDRBS3	snp	8	136594267	136594267	G	T
MEN0122-RBM-P1	AC092964.1	snp	3	172039500	172039500	A	T
MEN0122-RBM-P1	EN1	snp	2	119604523	119604523	A	G
MEN0122-RBM-P1	ADAMTSL3	snp	15	84611798	84611798	C	A
MEN0122-RBM-P1	FAM47C	snp	X	37027815	37027815	G	T
MEN0122-RBM-P1	CDHR5	snp	11	618674	618674	C	T
MEN0122-RBM-P1	ADM2	snp	22	50921652	50921652	C	T
MEN0122-RBM-P1	ZNF678	snp	1	227843156	227843156	T	A
MEN0122-RBM-P1	TPRXL	snp	3	14106310	14106310	T	C
MEN0122-RBM-P1	RP11-467N20.5	snp	15	23407305	23407305	T	C
MEN0122-RBM-P1	PRB4	snp	12	11461672	11461672	G	C
MEN0122-RBM-P2	ZDHHC15	snp	X	74742756	74742756	G	A
MEN0122-RBM-P2	GPR112	snp	X	135429389	135429389	C	A

MEN0122-RBM-P2	CDC27	snp	17	45247367	45247367	T	C
MEN0122-RBM-P2	PCMTD1	snp	8	52733227	52733227	C	T
MEN0122-RBM-P2	PRPF8	snp	17	1561862	1561862	C	A
MEN0122-RBM-P2	DPPA4	snp	3	109050809	109050809	G	T
MEN0122-RBM-P2	TMPRSS12	snp	12	51279241	51279241	G	T
MEN0122-RBM-P2	UHRF1BP1	snp	6	34824162	34824162	G	A
MEN0122-RBM-P2	ULK4	snp	3	41939933	41939933	T	C
MEN0122-RBM-P2	ADM2	snp	22	50921449	50921449	C	T
MEN0122-RBM-P2	LCT	snp	2	136575021	136575021	G	T
MEN0122-RBM-P2	CDH6	snp	5	31305516	31305516	C	T
MEN0122-RBM-P2	ATL3	snp	11	63426361	63426361	A	C
MEN0122-RBM-P2	ADM2	snp	22	50921652	50921652	C	T
MEN0122-RBM-P2	FBXL17	snp	5	107700659	107700659	C	A
MEN0122-RBM-P2	WFDC8	snp	20	44207861	44207861	C	T
MEN0122-RBM-P2	SLC25A5	snp	X	118603742	118603742	A	C
MEN0122-RBM-P2	CSMD3	snp	8	113353774	113353774	T	C
MEN0122-RBM-P2	SIDT1	snp	3	113326974	113326974	C	A
MEN0122-RBM-P2	ULK4	snp	3	41939933	41939933	T	C
MEN0122-RBM-P2	PCMTD1	snp	8	52733227	52733227	C	T
MEN0122-RBM-P2	ZDHHC15	snp	X	74742756	74742756	G	A
MEN0122-RBM-P2	SLC25A5	snp	X	118603742	118603742	A	C
MEN0122-RBM-P2	SIDT1	snp	3	113326974	113326974	C	A
MEN0122-RBM-P2	CDC27	snp	17	45247367	45247367	T	C
MEN0122-RBM-P2	TMPRSS12	snp	12	51279241	51279241	G	T
MEN0122-RBM-P2	ATL3	snp	11	63426361	63426361	A	C
MEN0122-RBM-P2	DPPA4	snp	3	109050809	109050809	G	T
MEN0122-RBM-P2	ADM2	snp	22	50921449	50921449	C	T
MEN0122-RBM-P2	CDH6	snp	5	31305516	31305516	C	T
MEN0122-RBM-P2	PRPF8	snp	17	1561862	1561862	C	A
MEN0122-RBM-P2	FBXL17	snp	5	107700659	107700659	C	A
MEN0122-RBM-P2	UHRF1BP1	snp	6	34824162	34824162	G	A
MEN0122-RBM-P2	LCT	snp	2	136575021	136575021	G	T
MEN0122-RBM-P2	GPR112	snp	X	135429389	135429389	C	A
MEN0122-RBM-P2	CSMD3	snp	8	113353774	113353774	T	C
MEN0122-RBM-P2	WFDC8	snp	20	44207861	44207861	C	T
MEN0122-RBM-P2	ADM2	snp	22	50921652	50921652	C	T
MEN0122-RBM-P3	ZDHHC15	snp	X	74742756	74742756	G	A
MEN0122-RBM-P3	GPR112	snp	X	135429389	135429389	C	A
MEN0122-RBM-P3	ZNF267	snp	16	31927455	31927455	C	G
MEN0122-RBM-P3	ZNF479	snp	7	57188238	57188238	C	T
MEN0122-RBM-P3	ZNF626	snp	19	20808090	20808090	C	T
MEN0122-RBM-P3	SLC11A1	snp	2	219250207	219250207	A	G
MEN0122-RBM-P3	FAM230A	snp	22	20710439	20710439	C	T
MEN0122-RBM-P3	ZNF430	snp	19	21239983	21239983	G	A
MEN0122-RBM-P3	NBPF10	snp	1	145359146	145359146	T	C
MEN0122-RBM-P3	ULK4	snp	3	41939929	41939929	T	A
MEN0122-RBM-P3	DPPA4	snp	3	109050809	109050809	G	T

MEN0122-RBM-P3	TMPRSS12	snp	12	51279241	51279241	G	T
MEN0122-RBM-P3	ZNF880	snp	19	52877732	52877732	A	G
MEN0122-RBM-P3	CDC27	snp	17	45234359	45234359	T	A
MEN0122-RBM-P3	ZNF729	snp	19	22498764	22498764	A	G
MEN0122-RBM-P3	ZNF714	snp	19	21300416	21300416	C	G
MEN0122-RBM-P3	ZNF732	snp	4	265139	265139	C	T
MEN0122-RBM-P3	ULK4	snp	3	41939933	41939933	T	C
MEN0122-RBM-P3	KHDRBS3	snp	8	136594267	136594267	G	T
MEN0122-RBM-P3	ZNF99	snp	19	22941699	22941699	T	C
MEN0122-RBM-P3	ADM2	snp	22	50921449	50921449	C	T
MEN0122-RBM-P3	LCT	snp	2	136575021	136575021	G	T
MEN0122-RBM-P3	ATL3	snp	11	63426361	63426361	A	C
MEN0122-RBM-P3	ABHD17A	snp	19	1881408	1881408	A	G
MEN0122-RBM-P3	ZNF66	snp	19	20989586	20989586	A	G
MEN0122-RBM-P3	ZNF430	snp	19	21240003	21240003	A	G
MEN0122-RBM-P3	FBXL17	snp	5	107700659	107700659	C	A
MEN0122-RBM-P3	SEC23A	snp	14	39562360	39562360	T	C
MEN0122-RBM-P3	WFDC8	snp	20	44207861	44207861	C	T
MEN0122-RBM-P3	ZNF714	snp	19	21300550	21300550	G	A
MEN0122-RBM-P3	ZNF254	snp	19	24310331	24310331	T	G
MEN0122-RBM-P3	CSMD3	snp	8	113353774	113353774	T	C
MEN0122-RBM-P3	ZNF716	snp	7	57528987	57528987	C	T
MEN0122-RBM-P3	ADAMTS2	snp	5	178578171	178578171	A	T
MEN0122-RBM-P3	ZNF681	snp	19	23926572	23926572	T	C
MEN0122-RBM-P3	ZNF728	snp	19	23158787	23158787	T	G
MEN0122-RBM-P3	ZNF91	snp	19	23544222	23544222	A	C
MEN0122-RBM-P3	ZNF724P	snp	19	23405690	23405690	T	C
MEN0122-RBM-P3	ZNF695	snp	1	247150500	247150500	A	T
MEN0122-RBM-P3	ZNF716	snp	7	57528892	57528892	G	A
MEN0122-RBM-P3	ZNF43	snp	19	21991811	21991811	G	T
MEN0122-RBM-P3	ULK4	snp	3	41939929	41939929	T	A
MEN0122-RBM-P3	ZDHHC15	snp	X	74742756	74742756	G	A
MEN0122-RBM-P3	ZNF880	snp	19	52877732	52877732	A	G
MEN0122-RBM-P3	TMPRSS12	snp	12	51279241	51279241	G	T
MEN0122-RBM-P3	ATL3	snp	11	63426361	63426361	A	C
MEN0122-RBM-P3	DPPA4	snp	3	109050809	109050809	G	T
MEN0122-RBM-P3	FAM230A	snp	22	20710439	20710439	C	T
MEN0122-RBM-P3	ADM2	snp	22	50921449	50921449	C	T
MEN0122-RBM-P3	NBPF10	snp	1	145359146	145359146	T	C
MEN0122-RBM-P3	FBXL17	snp	5	107700659	107700659	C	A
MEN0122-RBM-P3	SLC11A1	snp	2	219250207	219250207	A	G
MEN0122-RBM-P3	ZNF66	snp	19	20989586	20989586	A	G
MEN0122-RBM-P3	CDC27	snp	17	45234359	45234359	T	A
MEN0122-RBM-P3	ZNF729	snp	19	22498764	22498764	A	G
MEN0122-RBM-P3	ZNF714	snp	19	21300550	21300550	G	A
MEN0122-RBM-P3	ZNF716	snp	7	57528987	57528987	C	T
MEN0122-RBM-P3	LCT	snp	2	136575021	136575021	G	T

MEN0122-RBM-P3	GPR112	snp	X	135429389	135429389	C	A
MEN0122-RBM-P3	ZNF716	snp	7	57528892	57528892	G	A
MEN0122-RBM-P3	ZNF430	snp	19	21239983	21239983	G	A
MEN0122-RBM-P3	ZNF99	snp	19	22941699	22941699	T	C
MEN0122-RBM-P3	ABHD17A	snp	19	1881408	1881408	A	G
MEN0122-RBM-P3	ZNF695	snp	1	247150500	247150500	A	T
MEN0122-RBM-P3	ZNF430	snp	19	21240003	21240003	A	G
MEN0122-RBM-P3	ADAMTS2	snp	5	178578171	178578171	A	T
MEN0122-RBM-P3	ZNF267	snp	16	31927455	31927455	C	G
MEN0122-RBM-P3	CSMD3	snp	8	113353774	113353774	T	C
MEN0122-RBM-P3	ZNF254	snp	19	24310331	24310331	T	G
MEN0122-RBM-P3	ZNF91	snp	19	23544222	23544222	A	C
MEN0122-RBM-P3	ZNF714	snp	19	21300416	21300416	C	G
MEN0122-RBM-P3	ZNF626	snp	19	20808090	20808090	C	T
MEN0122-RBM-P3	ZNF681	snp	19	23926572	23926572	T	C
MEN0122-RBM-P3	ZNF732	snp	4	265139	265139	C	T
MEN0122-RBM-P3	WFDC8	snp	20	44207861	44207861	C	T
MEN0122-RBM-P3	ZNF728	snp	19	23158787	23158787	T	G
MEN0122-RBM-P3	ZNF724P	snp	19	23405690	23405690	T	C
MEN0122-RBM-P3	ZNF479	snp	7	57188238	57188238	C	T
MEN0122-RBM-P3	ZNF43	snp	19	21991811	21991811	G	T
MEN0122-RBM-P3	SEC23A	snp	14	39562360	39562360	T	C
MEN0122-RBM-P3	KHDRBS3	snp	8	136594267	136594267	G	T
MEN0122-RBM-P4	ZDHHC15	snp	X	74742756	74742756	G	A
MEN0122-RBM-P4	FAM230A	snp	22	20710406	20710406	C	T
MEN0122-RBM-P4	GPR112	snp	X	135429389	135429389	C	A
MEN0122-RBM-P4	FAM230A	snp	22	20710439	20710439	C	T
MEN0122-RBM-P4	DPPA4	snp	3	109050809	109050809	G	T
MEN0122-RBM-P4	TMPRSS12	snp	12	51279241	51279241	G	T
MEN0122-RBM-P4	CDC27	snp	17	45234359	45234359	T	A
MEN0122-RBM-P4	ADM2	snp	22	50921449	50921449	C	T
MEN0122-RBM-P4	LCT	snp	2	136575021	136575021	G	T
MEN0122-RBM-P4	CDH6	snp	5	31305516	31305516	C	T
MEN0122-RBM-P4	FBXL17	snp	5	107700659	107700659	C	A
MEN0122-RBM-P4	UBXN4	snp	2	136505239	136505239	G	C
MEN0122-RBM-P4	WFDC8	snp	20	44207861	44207861	C	T
MEN0122-RBM-P4	C14orf23	snp	14	29282228	29282228	A	G
MEN0122-RBM-P4	SIDT1	snp	3	113326974	113326974	C	A
MEN0122-RBM-P4	ZDHHC15	snp	X	74742756	74742756	G	A
MEN0122-RBM-P4	SIDT1	snp	3	113326974	113326974	C	A
MEN0122-RBM-P4	TMPRSS12	snp	12	51279241	51279241	G	T
MEN0122-RBM-P4	C14orf23	snp	14	29282228	29282228	A	G
MEN0122-RBM-P4	DPPA4	snp	3	109050809	109050809	G	T
MEN0122-RBM-P4	FAM230A	snp	22	20710406	20710406	C	T
MEN0122-RBM-P4	ADM2	snp	22	50921449	50921449	C	T
MEN0122-RBM-P4	CDH6	snp	5	31305516	31305516	C	T
MEN0122-RBM-P4	FBXL17	snp	5	107700659	107700659	C	A

MEN0122-RBM-P4	UBXN4	snp	2	136505239	136505239	G	C
MEN0122-RBM-P4	CDC27	snp	17	45234359	45234359	T	A
MEN0122-RBM-P4	LCT	snp	2	136575021	136575021	G	T
MEN0122-RBM-P4	GPR112	snp	X	135429389	135429389	C	A
MEN0122-RBM-P4	FAM230A	snp	22	20710439	20710439	C	T
MEN0122-RBM-P4	WFDC8	snp	20	44207861	44207861	C	T
MEN0122-RBM-P5	ADAT1	snp	16	75632205	75632205	G	C
MEN0122-RBM-P5	CERS2	snp	1	150939101	150939101	G	T
MEN0122-RBM-P5	ZDHHC15	snp	X	74742756	74742756	G	A
MEN0122-RBM-P5	GPR112	snp	X	135429389	135429389	C	A
MEN0122-RBM-P5	ZNF729	snp	19	22497504	22497504	A	G
MEN0122-RBM-P5	DPPA4	snp	3	109050809	109050809	G	T
MEN0122-RBM-P5	TMPRSS12	snp	12	51279241	51279241	G	T
MEN0122-RBM-P5	COL23A1	snp	5	177989201	177989201	A	C
MEN0122-RBM-P5	TALDO1	snp	11	759052	759052	C	T
MEN0122-RBM-P5	KHDRBS3	snp	8	136594267	136594267	G	T
MEN0122-RBM-P5	ADM2	snp	22	50921449	50921449	C	T
MEN0122-RBM-P5	LCT	snp	2	136575021	136575021	G	T
MEN0122-RBM-P5	CDH6	snp	5	31305516	31305516	C	T
MEN0122-RBM-P5	ADM2	snp	22	50921652	50921652	C	T
MEN0122-RBM-P5	FBXL17	snp	5	107700659	107700659	C	A
MEN0122-RBM-P5	WFDC8	snp	20	44207861	44207861	C	T
MEN0122-RBM-P5	MYOZ3	snp	5	150051758	150051758	C	A
MEN0122-RBM-P5	MT-CYB	snp	MT	15498	15498	G	A
MEN0122-RBM-P5	SIDT1	snp	3	113326974	113326974	C	A
MEN0122-RBM-P5	ZDHHC15	snp	X	74742756	74742756	G	A
MEN0122-RBM-P5	SIDT1	snp	3	113326974	113326974	C	A
MEN0122-RBM-P5	TMPRSS12	snp	12	51279241	51279241	G	T
MEN0122-RBM-P5	DPPA4	snp	3	109050809	109050809	G	T
MEN0122-RBM-P5	CERS2	snp	1	150939101	150939101	G	T
MEN0122-RBM-P5	ADM2	snp	22	50921449	50921449	C	T
MEN0122-RBM-P5	CDH6	snp	5	31305516	31305516	C	T
MEN0122-RBM-P5	FBXL17	snp	5	107700659	107700659	C	A
MEN0122-RBM-P5	MT-CYB	snp	MT	15498	15498	G	A
MEN0122-RBM-P5	COL23A1	snp	5	177989201	177989201	A	C
MEN0122-RBM-P5	LCT	snp	2	136575021	136575021	G	T
MEN0122-RBM-P5	GPR112	snp	X	135429389	135429389	C	A
MEN0122-RBM-P5	TALDO1	snp	11	759052	759052	C	T
MEN0122-RBM-P5	ZNF729	snp	19	22497504	22497504	A	G
MEN0122-RBM-P5	WFDC8	snp	20	44207861	44207861	C	T
MEN0122-RBM-P5	MYOZ3	snp	5	150051758	150051758	C	A
MEN0122-RBM-P5	KHDRBS3	snp	8	136594267	136594267	G	T
MEN0122-RBM-P5	ADAT1	snp	16	75632205	75632205	G	C
MEN0122-RBM-P5	ADM2	snp	22	50921652	50921652	C	T
MENex001	RBBP5	snp	1	205066482	205066482	C	T
MENex001	ALDOC	snp	17	26901135	26901135	G	A
MENex001	VPS33B	snp	15	91551125	91551125	G	C

MENex001	MAGED1	snp	X	51643364	51643364	A	G
MENex001	OR2L5	snp	1	248185925	248185925	C	T
MENex001	PCDHA4	snp	5	140187949	140187949	G	A
MENex001	UNC45B	snp	17	33482369	33482369	G	A
MENex001	KBTBD6	snp	13	41705327	41705327	C	A
MENex001	TPTE	snp	21	10941969	10941969	C	A
MENex001	EFHC1	snp	6	52317709	52317709	G	A
MENex001	MS4A4A	snp	11	60059787	60059787	C	T
MENex001	ALG1	snp	16	5132577	5132577	G	A
MENex001	RSF1	snp	11	77388026	77388026	T	C
MENex001	ERVFRD-1	snp	6	11104217	11104217	T	G
MENex001	LIPG	snp	18	47101895	47101895	C	G
MENex001	TET3	snp	2	74274327	74274327	C	T
MENex001	ALDOC	snp	17	26901135	26901135	G	A
MENex001	PCDHA4	snp	5	140187949	140187949	G	A
MENex001	MAGED1	snp	X	51643364	51643364	A	G
MENex001	MS4A4A	snp	11	60059787	60059787	C	T
MENex001	TPTE	snp	21	10941969	10941969	C	A
MENex001	UNC45B	snp	17	33482369	33482369	G	A
MENex001	KBTBD6	snp	13	41705327	41705327	C	A
MENex001	ALG1	snp	16	5132577	5132577	G	A
MENex001	EFHC1	snp	6	52317709	52317709	G	A
MENex001	ERVFRD-1	snp	6	11104217	11104217	T	G
MENex001	OR2L5	snp	1	248185925	248185925	C	T
MENex001	RSF1	snp	11	77388026	77388026	T	C
MENex001	TET3	snp	2	74274327	74274327	C	T
MENex001	VPS33B	snp	15	91551125	91551125	G	C
MENex001	RBBP5	snp	1	205066482	205066482	C	T
MENex001	LIPG	snp	18	47101895	47101895	C	G
MENex002	ESRRA	snp	11	64083328	64083328	C	T
MENex002	GALNT13	snp	2	155098617	155098617	G	A
MENex002	RUNX3	snp	1	25228811	25228811	G	T
MENex002	HERC2	snp	15	28566548	28566548	T	C
MENex002	GNPTAB	snp	12	102163959	102163959	C	T
MENex002	PHF23	snp	17	7140709	7140709	T	G
MENex002	ESRRA	snp	11	64083328	64083328	C	T
MENex002	RUNX3	snp	1	25228811	25228811	G	T
MENex002	HERC2	snp	15	28566548	28566548	T	C
MENex002	GNPTAB	snp	12	102163959	102163959	C	T
MENex002	PHF23	snp	17	7140709	7140709	T	G
MENex002	GALNT13	snp	2	155098617	155098617	G	A
MENex004	ARL6IP1	snp	16	18805974	18805974	G	A
MENex004	SLC52A3	snp	20	744485	744485	G	T
MENex004	JARID2	snp	6	15517416	15517416	G	A
MENex004	RNF103	snp	2	86832101	86832101	G	A
MENex004	TET1	snp	10	70406483	70406483	A	G
MENex004	PPP1R14D	snp	15	41120586	41120586	T	A

MENex004	ABCB1	snp	7	87190658	87190658	C	G
MENex004	BRWD3	snp	X	79978195	79978195	G	A
MENex004	SRGN	snp	10	70863653	70863653	G	A
MENex004	ZFP92	snp	X	152683794	152683794	T	G
MENex004	ENDOV	snp	17	78409975	78409975	A	G
MENex004	CNBP	snp	3	128890507	128890507	G	A
MENex004	SDR16C5	snp	8	57219235	57219235	C	T
MENex004	IFNW1	snp	9	21141089	21141089	C	T
MENex004	DNAH17	snp	17	76498708	76498708	A	G
MENex004	EIF3D	snp	22	36914954	36914954	C	T
MENex004	NDUFV2	snp	18	9122611	9122611	T	C
MENex004	COL17A1	snp	10	105836175	105836175	C	T
MENex004	FBXW10	snp	17	18682505	18682505	T	C
MENex004	TMF1	snp	3	69082743	69082743	G	A
MENex004	FANCD2	snp	3	10114944	10114944	A	C
MENex004	CLEC18A	snp	16	69996928	69996928	G	A
MENex004	C4BPA	snp	1	207317277	207317277	C	T
MENex004	FBXW10	snp	17	18682505	18682505	T	C
MENex004	SLC52A3	snp	20	744485	744485	G	T
MENex004	PPP1R14D	snp	15	41120586	41120586	T	A
MENex004	JARID2	snp	6	15517416	15517416	G	A
MENex004	BRWD3	snp	X	79978195	79978195	G	A
MENex004	CLEC18A	snp	16	69996928	69996928	G	A
MENex004	ARL6IP1	snp	16	18805974	18805974	G	A
MENex004	RNF103	snp	2	86832101	86832101	G	A
MENex004	DNAH17	snp	17	76498708	76498708	A	G
MENex004	IFNW1	snp	9	21141089	21141089	C	T
MENex004	C4BPA	snp	1	207317277	207317277	C	T
MENex004	TMF1	snp	3	69082743	69082743	G	A
MENex004	EIF3D	snp	22	36914954	36914954	C	T
MENex004	ABCB1	snp	7	87190658	87190658	C	G
MENex004	CNBP	snp	3	128890507	128890507	G	A
MENex004	SDR16C5	snp	8	57219235	57219235	C	T
MENex004	ZFP92	snp	X	152683794	152683794	T	G
MENex004	TET1	snp	10	70406483	70406483	A	G
MENex004	SRGN	snp	10	70863653	70863653	G	A
MENex004	NDUFV2	snp	18	9122611	9122611	T	C
MENex004	COL17A1	snp	10	105836175	105836175	C	T
MENex004	FANCD2	snp	3	10114944	10114944	A	C
MENex004	ENDOV	snp	17	78409975	78409975	A	G
MENex005	ZSWIM8	snp	10	75561189	75561189	A	T
MENex005	CLEC18B	snp	16	74451961	74451961	G	A
MENex005	CLK3	snp	15	74919950	74919950	A	G
MENex005	BAI3	snp	6	69349189	69349189	G	C
MENex005	KLHDC7A	snp	1	18809555	18809555	C	T
MENex005	ESRRB	snp	14	76905940	76905940	G	C
MENex005	WIPF1	snp	2	175432731	175432731	C	A

MENex005	CYP4B1	snp	1	47280757	47280757	C	T
MENex005	CST8	snp	20	23472444	23472444	A	G
MENex005	DMWD	snp	19	46289600	46289600	T	G
MENex005	HDLBP	snp	2	242176119	242176119	C	G
MENex005	FSCB	snp	14	44974261	44974261	C	A
MENex005	DNHD1	snp	11	6592389	6592389	G	C
MENex005	ALPP	snp	2	233246249	233246249	A	G
MENex005	DMWD	snp	19	46289600	46289600	T	G
MENex005	KLHDC7A	snp	1	18809555	18809555	C	T
MENex005	DNHD1	snp	11	6592389	6592389	G	C
MENex005	ZSWIM8	snp	10	75561189	75561189	A	T
MENex005	CLK3	snp	15	74919950	74919950	A	G
MENex005	WIPF1	snp	2	175432731	175432731	C	A
MENex005	CYP4B1	snp	1	47280757	47280757	C	T
MENex005	CLEC18B	snp	16	74451961	74451961	G	A
MENex005	ALPP	snp	2	233246249	233246249	A	G
MENex005	CST8	snp	20	23472444	23472444	A	G
MENex005	ESRRB	snp	14	76905940	76905940	G	C
MENex005	BAI3	snp	6	69349189	69349189	G	C
MENex005	FSCB	snp	14	44974261	44974261	C	A
MENex005	HDLBP	snp	2	242176119	242176119	C	G
MENex006	RPS15	snp	19	1440068	1440068	G	C
MENex006	KIF16B	snp	20	16553877	16553877	C	T
MENex006	SMO	snp	7	128850341	128850341	G	T
MENex006	PCDHB7	snp	5	140552940	140552940	A	G
MENex006	MUC4	snp	3	195505855	195505855	G	T
MENex006	PRSS12	snp	4	119234488	119234488	C	T
MENex006	SUPT5H	snp	19	39965042	39965042	G	C
MENex006	LETM1	snp	4	1838284	1838284	C	T
MENex006	AL354822.1	snp	GL000218.1	40869	40869	G	A
MENex006	PRKD2	snp	19	47195049	47195049	A	G
MENex006	FAM221B	snp	9	35825358	35825358	C	T
MENex006	SMO	snp	7	128850341	128850341	G	T
MENex006	LETM1	snp	4	1838284	1838284	C	T
MENex006	PCDHB7	snp	5	140552940	140552940	A	G
MENex006	RPS15	snp	19	1440068	1440068	G	C
MENex006	PRKD2	snp	19	47195049	47195049	A	G
MENex006	FAM221B	snp	9	35825358	35825358	C	T
MENex006	KIF16B	snp	20	16553877	16553877	C	T
MENex006	SUPT5H	snp	19	39965042	39965042	G	C
MENex006	MUC4	snp	3	195505855	195505855	G	T
MENex006	PRSS12	snp	4	119234488	119234488	C	T
MENex006	AL354822.1	snp	GL000218.1	40869	40869	G	A

Allele	Peptide
HLA-A02:01	GLWRCRSTRL
HLA-C12:03	FAKERESGE
HLA-C03:04	KAAAAAAAAPA
HLA-C12:03	PSSRIVGAV
HLA-C03:04	SSSSSSSSSSS
HLA-C12:03	QQQQQQQQQ
HLA-C03:04	GSSSSSSSSE
HLA-DQA105:0	TKAAAAAAAAPA
HLA-DQA105:0	GEPKGFHEAAAP
HLA-DRB111:0	ERGLWRCRSTRL
HLA-DQA105:0	PSSRIVGAVSSEG
HLA-DQA105:0	SGSSGGSSSSSSS
HLA-DQA105:0	STAPSSSSSSSSSS
HLA-DQA105:0	QQQQQQQQPP
HLA-A02:01	GLWRCRSTRL
HLA-C12:03	FAKERESGE
HLA-C03:04	KAAAAAAAAPA
HLA-C12:03	PSSRIVGAV
HLA-C03:04	SSSSSSSSSSS
HLA-C12:03	QQQQQQQQQ
HLA-DQA105:0	TKAAAAAAAAPA
HLA-DQA105:0	GEPKGFHEAAAP
HLA-DRB111:0	ERGLWRCRSTRL
HLA-DQA105:0	PSSRIVGAVSSEG
HLA-DQA105:0	STAPSSSSSSSSSS
HLA-DQA105:0	QQQQQQQQPP
HLA-A02:01	MLLQMLWYV
HLA-A02:01	KLWFSLINI
HLA-C05:01	FADIASHPGRL
HLA-A02:01	HLLMCPCGV
HLA-B44:02	SEASVNNLW
HLA-A02:01	LLVDVVYIFL
HLA-A02:01	MLLPQSLSQSL
HLA-A02:01	RLSSCVPV
HLA-A02:01	SLSKPVGTPV
HLA-B44:02	AEEEEAEAAAF
HLA-B44:02	KEGHLRVVEFW
HLA-A02:01	SLASSHSLL
HLA-B44:02	KEKQQDMRW
HLA-A02:01	ALLLLLSL
HLA-A02:01	RLPPSWAWA
HLA-A02:01	KMLNQKCLA
HLA-A02:01	KLKPYRSVLV
HLA-A02:01	GLWRCRSTRL
HLA-B44:02	GENGGVCGL
HLA-B44:02	GEQGETNEF

HLA-C05:01	PAEEAPSEV
HLA-A02:01	TLDTLTHPDTL
HLA-A02:01	STAVFSFGA
HLA-A02:01	ALIKKKKTAPT
HLA-C05:01	TSEEVVVVA
HLA-B44:02	KEQGSSVLL
HLA-C05:01	KEEEEEASK
HLA-C05:01	VSSPLCCQT
HLA-C05:01	LEEEEEEDE
HLA-B44:02	SPQQQQQTY
HLA-C05:01	MSWRGRSTYY
HLA-A02:01	ALNPGPRTQGS
HLA-A02:01	QAFNHKKQQQV
HLA-C05:01	DEEEEEEEK
HLA-C05:01	QQQQHQQQQ
HLA-C05:01	HTGPCCCCP
HLA-C05:01	ANPPAPAAT
HLA-C05:01	PCCCRPSCC
HLA-C05:01	CPGPGPSPG
HLA-C05:01	PSNHHHHNH
HLA-A02:01	SGCGGCGSGCA
HLA-B44:02	RQQQQQQQR
HLA-B44:02	RSRERERK
HLA-B44:02	MDVRKKKKN
HLA-A02:01	GCWGKKKKKS
HLA-DRB108:0	YFCFFRTCVKKK
HLA-DRB108:0	KTNLFSALIKKKKT
HLA-DRB108:0	VVQVAKKLKPYR
HLA-DRB108:0	HVPTWSARLLTR
HLA-DRB108:0	QMLWYVGRSSA
HLA-DQA104:0	STAVFSFGAATSS
HLA-DRB108:0	WFSLINIHHRKN
HLA-DRB104:0	QVLFRRNLTPSQ
HLA-DRB108:0	KCIMKEKKSLVRL
HLA-DQA104:0	SCAGAYSSAPAG
HLA-DRB108:0	RSSLRSLSKPVGT
HLA-DRB108:0	RSTYYRPRPRRYV
HLA-DRB108:0	ERGLWRCRSTRL
HLA-DQA104:0	VCGLESASAVPG
HLA-DQA103:0	AEEEEAEAAAFP
HLA-DRB104:0	FYLMELATALLLL
HLA-DRB104:0	KQGLLSEASVNN
HLA-DRB104:0	IPSLLTLDTLTHPD
HLA-DRB108:0	TPQIQAFNHKKQ
HLA-DRB108:0	RPAIARTQSVRT
HLA-DRB108:0	SEEVVVVAKPKG
HLA-DRB104:0	HHCLQTQSLASS

HLA-DQA104:	PASTTPASTTPAS
HLA-DQA104:	PATTANPPAPAA
HLA-DRB108:0	GIIDCVVRIPKEQ
HLA-DRB104:0	VDVVYIFLTLSCL
HLA-DRB108:0	KMSQEICTLKKEK
HLA-DQA103:	CDVIEYPLEEEEE
HLA-DRB108:0	EMDVRKKKKKN
HLA-DRB108:0	HLAAKEGHLRVV
HLA-DQA104:	PQQQQQTYGAI
HLA-DQA103:	SEVQPPPAEEAP
HLA-DRB108:0	SSGCWGKKKKKK
HLA-DRB108:0	PCCCCPCCPRLLL
HLA-DQA104:	TCRPSCGVSSPLC
HLA-DQA104:	KEEEEEASKVASK
HLA-DQA104:	GLGHMPEPALN
HLA-DQA104:	GSGCGGCGSGC
HLA-DRB104:0	PTTTTTTTTTTTT
HLA-DRB108:0	GLEKSPMKKKTE
HLA-DRB108:0	NHHHHNHHSHK
HLA-DQA104:	DSETQPDEEEEEE
HLA-DRB108:0	SCCQTTCCRTTC
HLA-DQA104:	SLKFSPGPCPGP
HLA-DRB108:0	VVQQQQQQQQ
HLA-DRB108:0	RQQQQQQQQR
HLA-DRB104:0	PNRKDDRSRERE
HLA-DRB108:0	QQQQQQQQHQ
HLA-A02:01	FLVTFLTML
HLA-C05:01	FADIASHPGRL
HLA-A02:01	SLLLLAYAFYV
HLA-A02:01	FLHLVQSLV
HLA-A02:01	CLSQIFFSL
HLA-A02:01	RIFKFSPPL
HLA-B44:02	KEGHLRVVEFW
HLA-A02:01	GMWRPLTLA
HLA-A02:01	GMPEASSSSV
HLA-B44:02	RERNQFHEFW
HLA-C05:01	IADTLRQQQQL
HLA-A02:01	SLASSHSLL
HLA-B44:02	KEKQQDMRW
HLA-C05:01	YSDWKEKKNI
HLA-B44:02	REREAVGIW
HLA-A02:01	GLWRCRSTRL
HLA-B44:02	CEGCDDSSIF
HLA-C05:01	CIDTCEKEL
HLA-A02:01	IIMKKMRRPV
HLA-C05:01	PAEEAPSEV
HLA-B44:02	DEEEEEGGSW

HLA-A02:01	HINDKTYEM
HLA-A02:01	TMSSSASAAA
HLA-A02:01	TLKKKNQEIYV
HLA-B44:02	EEQEKKSRKL
HLA-B44:02	KEQGSSVLL
HLA-B44:02	QEKKSRKRAV
HLA-B44:02	RERDEKKKC
HLA-B44:02	EELEKKNSDC
HLA-C05:01	VSSPLCCQT
HLA-B44:02	EENSLVKKKK
HLA-C05:01	MSWRGRSTYY
HLA-A02:01	GLRPPPPPHDM
HLA-A02:01	RLQRERAP
HLA-B44:02	REIKRLKKRN
HLA-B44:02	LEGQKHKKKL
HLA-B44:02	RERTREREG
HLA-C05:01	ANPPAPAAT
HLA-C05:01	PCCCRPSCC
HLA-C05:01	CPGPGPSPG
HLA-C05:01	PSNHHHHNH
HLA-C05:01	SAAAAAAAAGT
HLA-B44:02	YHHHHHHHHR
HLA-A02:01	GCWGKKKKKS
HLA-DQA104:0	LKEESAAAAAAAA
HLA-DRB108:0	GQIIMKKMRRPV
HLA-DRB108:0	WRPLTLARRTKSL
HLA-DRB108:0	TLRVFRVFRIFKFS
HLA-DQA104:0	PPTMSSSASAAAI
HLA-DRB108:0	LVTFLTMLPSFHL
HLA-DRB108:0	ERLRKRFLHLVQS
HLA-DRB108:0	KTYEMHKMRPKK
HLA-DRB108:0	PREIKRLKKRNQR
HLA-DQA104:0	SCAGAYSSAPAGI
HLA-DRB108:0	RSTYYRPRPRRYV
HLA-DRB108:0	QNEIQKLQKTLKK
HLA-DRB108:0	ERGLWRCRSTRLC
HLA-DRB104:0	FHEFWRMSDSRL
HLA-DRB108:0	IALAWMPKATVC
HLA-DRB104:0	HHCLQTQSLASSH
HLA-DQA104:0	PATTANPPAPAAT
HLA-DRB108:0	LPLLIADTLRQQQ
HLA-DQA104:0	LSQNGMPEASSS
HLA-DRB108:0	LSQIFFSLHLDAW
HLA-DRB108:0	EQEKKSRKRAVSN
HLA-DRB108:0	GIIDCVVRIPKEQC
HLA-DRB108:0	KMSQEICTLKKEK
HLA-DRB108:0	KEHLEGQKHKKKI

HLA-DRB108:0	LEEQEKKSRKLMS
HLA-DQA104:0	LDSLIAWWESLLL
HLA-DRB104:0	QPLEKNRFCSSSS
HLA-DRB108:0	HHHYHHHHHHH
HLA-DRB108:0	HLAAKEGHLRVVI
HLA-DRB104:0	LSVTSEENSLVKKI
HLA-DQA103:0	CEKELGAGGDRL(
HLA-DRB108:0	REERERLQRERAF
HLA-DQA103:0	SEVQPPPAEEAPA
HLA-DRB108:0	SSGCWGKKKKKK
HLA-DQA104:0	TCRPSCGVSSPLC
HLA-DQA104:0	VSKQNSIIEELEKK
HLA-DQA104:0	GMTRDEDDDEEI
HLA-DRB108:0	NHHHHNHHSHK
HLA-DQA103:0	EWSKEREREAVG
HLA-DQA104:0	RPPPPPHDMGPA
HLA-DRB108:0	SCCQTTCCRTTCC
HLA-DQA104:0	SLKFSPGPCPGPG
HLA-DQA103:0	SSDFNERRERDEK
HLA-DRB108:0	DRDRERERTRERI
HLA-C05:01	FADIASHPGRL
HLA-B44:02	KEGHLRVVEFW
HLA-A02:01	SLASSHSLL
HLA-DQA104:0	SCAGAYSSAPAGI
HLA-DRB104:0	HHCLQTQSLASSH
HLA-DRB108:0	HLAAKEGHLRVVI
HLA-A03:01	KQMERQRLAER
HLA-B57:01	FSALIKKKKT
HLA-DRB107:0	KKTNLFSALIKKKK
HLA-DQA102:0	KARKQMERQRLA
HLA-A24:02	IYFDFYDLLF
HLA-B35:02	VPACSHVPL
HLA-C07:01	VRSSGSRNS
HLA-C07:01	WRCRSTRLQ
HLA-B35:02	FAVLGTMVM
HLA-C07:01	SRTETPPWR
HLA-B35:02	MPWPLLLLAV
HLA-B08:01	SKLNKITSL
HLA-A24:02	LFFFVAVDIF
HLA-C07:01	FNYLHSTYL
HLA-B35:02	EPAAAAAAAI
HLA-A24:02	AFGGSTAVFSF
HLA-C07:01	VRIPKEQGS
HLA-B35:02	VPIDLDPGL
HLA-B08:01	MLLLLLLGL
HLA-C07:01	ERESGEPKG
HLA-B08:01	FFHKKKTA

HLA-B35:02	RPSCGVSSPL
HLA-C07:01	QRQGREEED
HLA-B08:01	TALLLLLSL
HLA-C07:01	RREEEEDAF
HLA-C07:01	IRETFHLVSEM
HLA-B35:02	CAAAAAAACL
HLA-B35:02	TPLPPSADDNL
HLA-B08:01	TLKKEKQQDM
HLA-C07:01	SVTQQQQQS
HLA-C07:01	KSAPTTKEP
HLA-B08:01	TLLLLLGGAL
HLA-C07:01	HTHRANRRW
HLA-A01:01	LSPQQQQQTY
HLA-C07:01	HVWAQSPGE
HLA-B35:02	FPVLLLLLSG
HLA-C07:01	SAGDEEEED
HLA-B08:01	LLQEEEEL
HLA-B08:01	WGKKKKKKSQ
HLA-C07:01	SGAEEEEEN
HLA-C07:01	SKNAADWGW
HLA-C07:01	EAPPPPPAT
HLA-A01:01	SSESEEEEEA
HLA-C04:01	DDDEEEEEG
HLA-C07:01	HTGPCCCCP
HLA-C04:01	EGEEEEGEE
HLA-C04:01	ELELMPEKA
HLA-B08:01	QPQQQQQQPP
HLA-C07:01	LQQQQQQQQ
HLA-DQA105:	AVYVEPAAAAAA
HLA-DQA105:	MMEIACAAAAA
HLA-DQA105:	STAVFSFGAATSS
HLA-DQA105:	GEPKGFHEAAAP
HLA-DQA105:	LALLGASAPCGA
HLA-DQA105:	TLLLLLGGALAHP
HLA-DQA105:	PLLLLAVSGAQTT
HLA-DRB111:0	MILYVSKLNKITSL
HLA-DRB111:0	HVPMRSARLLTR
HLA-DRB111:0	AEFNYLHSTYLRT
HLA-DQA105:	VTLSLGWPAAPR
HLA-DQA105:	DLDPGLAEKAAG
HLA-DRB111:0	SPVFFHKKKTAG
HLA-DRB111:0	QHCLLGLAKPSLV
HLA-DRB111:0	IIRETFHLVSEMK
HLA-DQA105:	LAWMPKATVQA
HLA-DQA105:	FYLMELATALLLL
HLA-DRB111:0	GIIDCVVRIPKEQ
HLA-DRB111:0	SQEICTLKKEKQQ

HLA-DQA105:0	VLLLLLSGDVQSS
HLA-DRB111:0	MLLLLLLGLRLQL
HLA-DRB111:0	RGLWRCRSTRLQ
HLA-DQA105:0	AFAGKLPEPSKN
HLA-DRB111:0	SGCWGKKKKKS
HLA-DQA105:0	PQQQQQTYGAI
HLA-DQA105:0	TCRPSCGVSSPLC
HLA-DQA105:0	LEAITVSAGDEEE
HLA-DRB111:0	VVYIFLTLSCLGIF
HLA-DQA105:0	EYTVHVWAQSP
HLA-DQA105:0	EPAPTTTKSAPTT
HLA-DQA105:0	ECLLTPLPPSADD
HLA-DQA105:0	QTSGETTHTHRA
HLA-DQA105:0	QRQQEAPPPPPA
HLA-DRB111:0	QFTKIKELELMPE
HLA-DRB104:0	EQQLLQEEEELQ
HLA-DQA103:0	TMLIHSSESEEEE
HLA-DQA105:0	DTPMMVHTGPC
HLA-DQA103:0	QQSQTLSVTQQ
HLA-DQA105:0	DEEEEEGGSWG
HLA-DRB111:0	KSKAKSKARREEE
HLA-DQA103:0	DCKPRSGAEEEEE
HLA-DRB111:0	RLAHQQQRQGR
HLA-DRB111:0	ATLQLQQQQQQ
HLA-DQA105:0	QPQQQQQQPPP
HLA-DQA103:0	LGATEEEEEGEEE
HLA-DQA103:0	DEEEEEEEEEEEEE
HLA-A24:02	GYTIRMLWTF
HLA-B35:02	VPACSHVPL
HLA-C07:01	RRSCPRRAG
HLA-C07:01	VRSSGSRNS
HLA-C07:01	WRCRSTRLQ
HLA-C07:01	RRRCCRRGL
HLA-C07:01	GRSPEGQHM
HLA-C07:01	YYRPRPRRY
HLA-B08:01	EKKSRKRAV
HLA-B08:01	RLATRLKTSL
HLA-A24:02	LFFFVAVDIF
HLA-B35:02	EPAAAAAAAI
HLA-C07:01	RRVLHIPKP
HLA-C07:01	VRIPKEQGS
HLA-B08:01	FFHKKKTA
HLA-B35:02	RPSCGVSSPL
HLA-C07:01	IRETFHLVSEM
HLA-A24:02	KFYQWYCW
HLA-B08:01	TLKKEKQQDM
HLA-B08:01	MSTKKKCV

HLA-C07:01	SSSGSSSSD
HLA-C07:01	ESCSFAPTV
HLA-C07:01	HTHRANRRW
HLA-C07:01	HVWAQSPGE
HLA-B08:01	WGKKKKKKSQ
HLA-C07:01	SKNAADWGW
HLA-C07:01	TTKEPAPTA
HLA-C07:01	QQCVTLWTR
HLA-B08:01	QPQQQQQPP
HLA-DQA105:0	AVYVEPAAAAAA
HLA-DRB111:0	LNGIRRVLHIPKPI
HLA-DRB111:0	MLWTFVQSFKAL
HLA-DRB111:0	AVLLYKISNLLKFY
HLA-DRB111:0	HVPMRSARLLTR
HLA-DQA105:0	RAGSCGRAAPRA
HLA-DRB111:0	NFWPARLATRLK
HLA-DRB111:0	RSTYYRPRPRRYV
HLA-DRB111:0	GRSTYYRPRPRRY
HLA-DRB111:0	SPVFFHKKKTAGK
HLA-DQA105:0	GGGGGSGSSSGS
HLA-DRB111:0	QHCLLGLAKPSLV
HLA-DRB111:0	IIRETFHLVSEMKI
HLA-DQA105:0	TVEASGGGPCPSI
HLA-DQA105:0	LAWMPKATVQA
HLA-DRB111:0	GIIDCVVRIPKEQ
HLA-DRB111:0	SQEICTLKKEKQQ
HLA-DRB111:0	RGLWRCRSTRLQ
HLA-DQA105:0	AFAGKLPEPSKN
HLA-DRB111:0	IEELPRRRCCRRG
HLA-DRB111:0	SGCWGKKKKKS
HLA-DQA105:0	TCRPSCGVSSPLC
HLA-DQA105:0	EYTVHVWAQSP
HLA-DQA105:0	VTKPNSALESCSF
HLA-DRB111:0	GMSTKKKCVHHV
HLA-DRB111:0	EQEKKSRKRAVSI
HLA-DQA105:0	PAPTTKEPAPTAF
HLA-DQA105:0	QTSGETTHTHRA
HLA-DRB104:0	CQTHEQQCVTLV
HLA-DQA105:0	QPQQQQQQPPP
HLA-A24:02	GYTIRMLWTF
HLA-B08:01	RLKCKACRL
HLA-B35:02	VPACSHVPL
HLA-B08:01	LPQVRLSRL
HLA-A24:02	IWMPGQFTL
HLA-C07:01	VRSSGSRNS
HLA-C07:01	WRCRSTRLQ
HLA-B08:01	LIKKKKTA

HLA-C07:01	YYRPRPRRY
HLA-B08:01	KPLKKNLHL
HLA-B08:01	RLATRLKTSL
HLA-C07:01	MATTRPRGH
HLA-C07:01	RRVLHIPKP
HLA-A24:02	AYAEWRGRPPF
HLA-C07:01	VRIPKEQGS
HLA-B35:02	VPSASPWGL
HLA-B35:02	RPSCGVSSPL
HLA-A24:02	KFYQWYCW
HLA-B08:01	TLKKEKQQDM
HLA-C07:01	SSSGSSSSD
HLA-C07:01	CRPCCCRPS
HLA-C07:01	SISGLVRAS
HLA-C07:01	HVWAQSPGE
HLA-C07:01	DTLTHPDTL
HLA-B08:01	WGKKKKKKSQ
HLA-C07:01	SKNAADWGW
HLA-C07:01	TTKEPAPTA
HLA-C07:01	PQQQQQQP
HLA-A01:01	SGCGGCGSGCA
HLA-A01:01	AVEEEEEEED
HLA-DRB111:0	TNLFSALIKKKKTA
HLA-DRB111:0	RPLRHLGRLLGRR
HLA-DRB111:0	FIAVLKYMATTRP
HLA-DRB111:0	LNGIRRVLHIPKPI
HLA-DRB111:0	KKKFLIHLRKVDTI
HLA-DRB111:0	MLWTFVQSFKAL
HLA-DRB111:0	AVLLYKISNLLKFY
HLA-DRB111:0	HVPMRSARLLTR
HLA-DRB111:0	RLCYKQLKSISGLV
HLA-DRB111:0	NFWPARLATRLK
HLA-DRB111:0	RSTYYRPRPRRYV
HLA-DRB111:0	GRSTYYRPRPRRY
HLA-DQA105:0	GGGGGSGSSSGS
HLA-DRB111:0	FFHFIWMPGQFT
HLA-DQA105:0	LAWMPKATVQA
HLA-DRB104:0	HLCYYHSQSNRN
HLA-DRB104:0	IPSLLTLDTLTHPD
HLA-DRB111:0	GIIDCVVRIPKEQ
HLA-DRB111:0	SQEICTLKKEKQQ
HLA-DRB111:0	RGLWRCRSTRLQ
HLA-DQA105:0	GKRRDGAAYAEV
HLA-DQA105:0	GSGCGGCGSGCA
HLA-DQA105:0	AFAGKLPEPSKNA
HLA-DRB111:0	SGCWGKKKKKKS
HLA-DQA105:0	TCRPSCGVSSPLC

HLA-DQA105:0	EYTVHVWAQSP(
HLA-DQA105:0	PAPTTKEPAPTAF
HLA-DQA105:0	PECQKKVPSASP\
HLA-DQA103:0	YYDYAVEEEEEEE
HLA-DQA105:0	SQQQQQQQQQ(
HLA-DRB104:0	PYCCESSCCRPCC
HLA-DQA103:0	PQQQQQQQPQ\
HLA-C07:01	LRPPCPLLV
HLA-B08:01	RPRGHLLPL
HLA-A24:02	SYKEPKKITF
HLA-B08:01	RLATRLKTSL
HLA-C07:01	LRPGVPESL
HLA-A24:02	KFYQWYCW
HLA-C07:01	LRSCGTLSF
HLA-B08:01	NGIRRVLHI
HLA-C07:01	QQAETSEEL
HLA-A01:01	VIDFGQAV
HLA-C07:01	HVWAQSPGE
HLA-A01:01	FICKQASCSY
HLA-C04:01	FMDYLSDKQ
HLA-A24:02	KNAADWGW
HLA-DQA105:0	ATNMRAAPAPA/
HLA-DRB111:0	LNGIRRVLHIPKPI
HLA-DRB111:0	AVLLYKISNLLKFY
HLA-DRB111:0	NFWPARLATRLK
HLA-DQA105:0	ETSEELGAVTVKA
HLA-DRB111:0	MNTRKYTRTSKA
HLA-DRB111:0	SPWLLHLGMRST
HLA-DQA105:0	LRSLRPGVPESLA
HLA-DQA105:0	RKKDSGSKKAVIS
HLA-DQA105:0	AFAGKLPEPSKN/
HLA-DRB111:0	REDIEFICKQASCS
HLA-DRB111:0	EREDIEFICKQAS(
HLA-DQA105:0	GLRSCGTLSFVLP/
HLA-DQA105:0	EYTVHVWAQSP(
HLA-DRB104:0	LQKGISFMDYLSD
HLA-C05:01	ILDPGQTSW
HLA-B44:02	REAQQQHLY
HLA-B27:05	ARWTSTQR
HLA-C05:01	ISTQQQSHF
HLA-B44:02	YLNLSAHEY
HLA-B44:02	NGSFCLPPW
HLA-B27:05	YMNTNLVRR
HLA-DRB108:0	YGGLSLSIRAPAR\
HLA-DRB108:0	PEKMKYLNLSAHI
HLA-DRB108:0	CYMNTNLVRRTS
HLA-DQA104:0	LSDGFVAAEAKTI

HLA-DRB104:0	AQQQHLYRVNIN
HLA-DRB108:0	DTGLFISTQQQSH
HLA-DRB108:0	NGSFCLPPWPHG
HLA-B07:02	RPCRRPGRPA
HLA-A02:01	FASKSMFSL
HLA-C07:02	TVSKKKQEK
HLA-DRB115:0	AQRIFASKSMFSL
HLA-DQA101:0	LAAGRSEGALQSS
HLA-DRB113:0	LEPKAVTDQTVSK
HLA-B44:03	NESTMTSPW
HLA-B44:03	EEIDGFFARI
HLA-A11:01	TLSWALSLLR
HLA-A11:01	RPLASWCPR
HLA-B44:03	GLWRSATSW
HLA-B44:03	MEACGTHPT
HLA-B44:03	EEEEEEEEEEE
HLA-C04:01	RRRPSQPYQ
HLA-DRB108:0	LRLYARRRRPSQP
HLA-DRB108:0	PTLSWALSLLRIPP
HLA-DRB108:0	EIDGFFARITKSTV
HLA-DRB108:0	TYSGLWRSATSW
HLA-DRB108:0	PRTRGLWARCRV
HLA-DRB108:0	VQKYLLTLMNIVN
HLA-DQA102:0	PKKSNGMEACGT
HLA-DQA102:0	EEEGEEEEEEEEEI
HLA-B08:01	TPKKKRSAF
HLA-B08:01	MFIKRWKPA
HLA-B08:01	SLRKRQKF
HLA-B57:01	LACSHVPMW
HLA-C07:01	WRCRSTRLQ
HLA-B57:01	QIFFFHFIW
HLA-B57:01	KVSFRVDW
HLA-A01:01	YFDFYDLLF
HLA-B08:01	FNHKKQQQV
HLA-B57:01	GSTAVFSF
HLA-C07:01	ERLQRERAP
HLA-B57:01	RSKGLVLY
HLA-C07:01	SSSGGSSGN
HLA-B08:01	RPSCGVSSPL
HLA-C07:01	CRPCCCRPS
HLA-C07:01	GSYGSGGGS
HLA-C06:02	PAEEAPSEV
HLA-B57:01	LTTCCRTTCC
HLA-A01:01	SGCGGCGSGCA
HLA-DRB115:0	VIKHPLIFRINLRR
HLA-DRB107:0	EAQRIFASKSMFS
HLA-DRB107:0	GVRILKLCSKVSFF

HLA-DRB107:0	WSARLLTQSPGM
HLA-DRB107:0	STAVFSFGAATSS
HLA-DRB107:0	QRERGLWRCRST
HLA-DRB115:0	RSKGLVLYVSPPQ
HLA-DRB107:0	IFFFHFIWMPGQI
HLA-DRB107:0	DVVYIFLTLSCLGI
HLA-DRB107:0	PTTTTTTTTTTTTI
HLA-DRB107:0	ETPKKKRSAFPSR
HLA-DQA101:0	GKISTITPQIQAFN
HLA-DQA102:0	EVQPPPAEEAPSE
HLA-DQA102:0	RPSCGVSSPLCCC
HLA-DQA102:0	GRGSYGSGGGSY
HLA-DQA101:0	SCCLTTCCRTTCCI
HLA-DRB107:0	REERERLQRERAP
HLA-DQA102:0	DNNNGSSSGGSS
HLA-DQA101:0	SCCQTTCCRTTCC
HLA-DQA102:0	GSGCGGCGSGCA
HLA-C06:02	YRRNLCHLL
HLA-B08:01	HLRAKKYSV
HLA-B08:01	RLKCKACRL
HLA-C07:01	YYRPRPRRY
HLA-B57:01	ISNQMMTF
HLA-B57:01	TAGVPCQRW
HLA-B57:01	ASTSLSSDW
HLA-B57:01	RLLAETCW
HLA-B57:01	KVSFRVDW
HLA-B08:01	LWRCRSTRL
HLA-B57:01	AAMPYLPQGSW
HLA-C07:01	FNHKKQQQV
HLA-C07:01	LRQQQQLL
HLA-C07:01	IYFDFYDLL
HLA-B57:01	RSKGLVLY
HLA-B08:01	SPMKKKTEE
HLA-C07:01	NHHHHNHHS
HLA-B57:01	KEKQQDMRW
HLA-B57:01	GSTAVFSF
HLA-B08:01	FFHKKKTA
HLA-C06:02	FAQYNSYV
HLA-C07:01	ERLQRERAP
HLA-C07:01	CRPSCGVSSPL
HLA-B57:01	MTVAGPRGGI
HLA-A01:01	GSYGSGGGSY
HLA-C07:01	SSSGGSSGN
HLA-C07:01	CRPCCCRPS
HLA-B57:01	HTGPCCCCPCC
HLA-C07:01	PAEEAPSEV
HLA-B57:01	LTTCCRTTCC

HLA-C07:01	TVSKKKQEK
HLA-C07:01	EAAPARPRP
HLA-C07:01	CSSGGCCSS
HLA-C07:01	EDDDDDDVV
HLA-A01:01	SSESEEEEEA
HLA-A01:01	ASDCCLCCCCG
HLA-A01:01	EEEGEEEEGEE
HLA-A01:01	QPDEEEEEEEK
HLA-DRB107:0	GVRILKLCSKVSFF
HLA-DQA101:0	LCGMREVRVVAA
HLA-DRB107:0	STAVFSFGAATSS
HLA-DQA101:0	DSWALSLRAQAL
HLA-DRB107:0	QRERGLWRCRST
HLA-DRB115:0	RSKGLVLYVSPPQ
HLA-DRB107:0	AERHFDHLRAKKY
HLA-DRB107:0	FLIHLRKVDTHHL
HLA-DQA101:0	RQQQQLLSAIIEA
HLA-DRB107:0	DVVYIFLTLSCLGI
HLA-DRB115:0	STDFAQYNSYVG
HLA-DQA102:0	EYFIRLEEAAPARF
HLA-DRB107:0	GYFPLSHPMTVA
HLA-DRB107:0	RGRSTYYRPRPRF
HLA-DQA102:0	REGQAVDASTSLS
HLA-DQA101:0	GKISTITPQIQAFN
HLA-DQA102:0	GTANTTTAGVPC
HLA-DQA101:0	TCTPISNQMMTF
HLA-DRB115:0	VRKNLSPVFFHKK
HLA-DQA102:0	EDDDDDDVVFIES
HLA-DQA102:0	KQQDMRWVEQI
HLA-DRB107:0	KKKEACFKRLLAE
HLA-DQA102:0	TMLIHSSESEEEEE
HLA-DQA102:0	EVQPPPAEEAPSE
HLA-DQA102:0	RPSCGVSSPLCCC
HLA-DRB107:0	KEKHKTHPSNHH
HLA-DQA101:0	PVSSCCSVSSGGC
HLA-DQA102:0	LEPKAVTDQTVSK
HLA-DQA102:0	YTCLASDCCLCCC
HLA-DQA101:0	DTPMMVHTGPC
HLA-DQA102:0	GRGSYGSGGGSY
HLA-DQA101:0	SCCLTTCCRTTCC
HLA-DRB107:0	REERERLQRERAF
HLA-DQA102:0	DNNNGSSSGGSS
HLA-DQA101:0	SCCQTTCCRTTCC
HLA-DQA102:0	DEEEEEEEKVSQP
HLA-DQA102:0	VVQQQQQQQQ
HLA-DQA102:0	EEEREQGEQGET
HLA-DQA102:0	LGATEEEEEGEEE

HLA-B57:01	KSLCHCDRW
HLA-B57:01	HSGNSSESW
HLA-B08:01	IMKKMRRPV
HLA-B08:01	KLKPYRSVL
HLA-B57:01	LACSHVPMW
HLA-B57:01	ASTSLSSDW
HLA-B57:01	SSQETPNYSW
HLA-C07:01	WRCRSTRLQ
HLA-B08:01	YTKERKLL
HLA-B08:01	EPKKSLIPL
HLA-A01:01	LSEKKNYALY
HLA-B08:01	LPSCKKKLL
HLA-A01:01	YFDFYDLLF
HLA-C06:02	RRKEGPNSN
HLA-C06:02	WSKEREREA
HLA-B57:01	GSTAVFSF
HLA-C07:01	ERLQRERAP
HLA-B57:01	RSKGLVLY
HLA-C07:01	LRQQQQLLS
HLA-C07:01	ERESGEPKG
HLA-C07:01	YRRAISKKR
HLA-C07:01	SYVGCQRGS
HLA-B08:01	ELNYVPLAL
HLA-B08:01	SGFLKFFFL
HLA-A01:01	YFFFLFHMY
HLA-C07:01	IRETFHLVSEM
HLA-A01:01	AQDDHLTQY
HLA-C07:01	EREQGEQGE
HLA-B57:01	ITAFFLLDI
HLA-C07:01	SSSGGSSGN
HLA-B08:01	RPSCGVSSPL
HLA-C07:01	AKNGCKQDN
HLA-B57:01	TTTSSTTTTI
HLA-C07:01	CRPCCCRPS
HLA-C07:01	GSYGSGGGS
HLA-C07:01	ATTTSQPSR
HLA-A01:01	LSPQQQQQTY
HLA-C06:02	NVFLVCLIL
HLA-C07:01	CSSGGCCSS
HLA-B57:01	HTGPCCCCPCC
HLA-B08:01	NLKSKYFAVC
HLA-C07:01	SAGDEEEED
HLA-C07:01	DTLTHPDTL
HLA-B08:01	LLQEEEEL
HLA-C07:01	PSNHHHHNH
HLA-A01:01	AVDDDDDAA
HLA-B08:01	TPKKKKKDPN

HLA-C07:01	VHLGCVCGQ
HLA-B08:01	FIYIIFFS
HLA-C07:01	CQLAKKKTK
HLA-A01:01	EMDVCNAFFS
HLA-B57:01	KAKSPEKAKS
HLA-B57:01	YACKKLQKKE
HLA-A01:01	NQDDDDDDDGF
HLA-A01:01	VSEDEDDDDA
HLA-C07:01	EGEEEEGEE
HLA-A01:01	SGCGGCGSGCA
HLA-DRB115:0	AKKLKPYRSVLVLI
HLA-DRB115:0	VAKKLKPYRSVLV
HLA-DRB115:0	KKLKPYRSVLVLIK
HLA-DRB107:0	GLYFSNLKSKYFA
HLA-DRB107:0	QIDVKISSVNVIIV
HLA-DRB107:0	WSARLLTQSPGM
HLA-DRB107:0	STAVFSFGAATSS
HLA-DRB107:0	QRERGLWRCRST
HLA-DRB115:0	RSKGLVLYVSPPQ
HLA-DRB107:0	HRQLLYITAFFLLD
HLA-DRB115:0	LGQIIMKKMRRP
HLA-DRB107:0	IRETFHLVSEMKM
HLA-DRB107:0	TSSVSTTTSSTTTT
HLA-DQA102:0	GEPKGFHEAAAP
HLA-DQA101:0	RQQQQLLSAIIEA
HLA-DQA102:0	DFSKFGAGILEVFI
HLA-DRB107:0	DVVYIFLTLSCLGI
HLA-DRB107:0	PTTTTTTTTTTTTI
HLA-DQA101:0	LEAITVSAGDEEEI
HLA-DRB115:0	STDFAQYNSYVG
HLA-DQA102:0	RNQAIYAAVDDD
HLA-DRB107:0	DAQEYRRAISKKR
HLA-DQA102:0	REGQAVDASTSLS
HLA-DRB107:0	GHTQPSQVSTWI
HLA-DQA101:0	AIVEEEMFLVMG
HLA-DRB115:0	GEDCYFFFLFHM
HLA-DRB107:0	YPLSEKKNYALYV
HLA-DRB115:0	VVLPSCKKKLLLKL
HLA-DRB107:0	KTTLPTLNVFLVCI
HLA-DRB107:0	PLLFRALVHLGCV
HLA-DQA102:0	ASERTALSPQQQ
HLA-DRB107:0	VGFSSILFIYIIFFS
HLA-DQA102:0	TTDNATTTSQPSI
HLA-DQA102:0	EEFISEMDVCNAI
HLA-DQA102:0	LGSARRAAGSDA
HLA-DRB115:0	DTSVFLNWYNPE
HLA-DQA101:0	SEVSKLRCQLAKK

HLA-DRB107:0	PSLLTLDTLTHPD
HLA-DQA102:	RPSCGVSSPLCCC
HLA-DRB107:0	KEKHKTHPSNHH
HLA-DQA101:	PVSSCCSVSSGG
HLA-DQA102:	ITEHEVAQDDHL
HLA-DQA102:	EWSKEREREAVG
HLA-DRB115:0	ATGKMYACKKL
HLA-DQA102:	IDEVIYPCPVEPK
HLA-DQA102:	QLRKEQQLLQEE
HLA-DQA102:	EDDDDDATPPP
HLA-DRB115:0	KNGCKQDNSLYR
HLA-DQA101:	DTPMMVHTGPC
HLA-DQA102:	GRGSYGSGGGSY
HLA-DRB107:0	REERERLQRERA
HLA-DQA102:	DNNNGSSSGGSS
HLA-DQA101:	SCCQTTCCRTTC
HLA-DQA102:	QDDDDDDDGFF
HLA-DQA102:	GSGCGGCGSGC
HLA-DQA102:	EEEREQGEQGET
HLA-DQA102:	LGATEEEEEGEEE
HLA-DQA102:	AKSPEKAKSPEKA
HLA-DQA102:	KNRRKEGPNSNR
HLA-DRB115:0	DMGKKPKTPKKK
HLA-C06:02	FAQYNSYV
HLA-DRB115:0	STDFAQYNSYVG
HLA-A31:01	RGRPRGAGR
HLA-A01:01	EDDDDDDDDEL
HLA-DRB114:5	KRLLRLQQERGK
HLA-DQA101:	CVFLDEDDDDDD
HLA-B07:02	RPTGAWSSM
HLA-C07:02	FLPDQIRDL
HLA-B07:02	LPLLLLLLLLP
HLA-DRB111:0	LETIIKSLKSTQDE
HLA-DRB111:0	PLLLLLLLLPAPEL
HLA-DRB111:0	YLLWVFLPDQIRD
HLA-B07:02	KPRAQNLPPV
HLA-C03:03	GALGVLLAL
HLA-C03:03	MSSCLQSPPL
HLA-C07:02	RRFSGRLSGRL
HLA-B07:02	KPASWAQRRI
HLA-C03:03	VDYKKNTGI
HLA-B07:02	GPGKGIPVGGG
HLA-DRB101:0	RGALGVLLALGA
HLA-DRB101:0	RRRFSGRLSGRLG
HLA-DRB101:0	LKFQMSSCLQSP
HLA-DRB101:0	QLCIPPLNSKPAS
HLA-DRB101:0	GDKGKPRAQNL

HLA-DRB101:0	LSTPGPGKGIPVG
HLA-DRB101:0	GPADGEVDYKKN
HLA-C03:03	AASAHGRPL
HLA-B07:02	SPAWSRGAA
HLA-B07:02	RPHPRGSPA
HLA-C03:03	SGSGSGSGA
HLA-C07:02	VKEDFIQQR
HLA-B07:02	GPGKGIPVGGG
HLA-DRB101:0	EQGFRRPAAAAS
HLA-DRB101:0	KEDFIQQRNVSCI
HLA-DRB101:0	PKAGFLPRPSGA
HLA-DQA101:0	SGSGSGSGASSPI
HLA-DRB101:0	LSTPGPGKGIPVG
HLA-A02:01	VLGRELLLLV
HLA-A02:01	ILGKLILLST
HLA-C07:02	VKEDFIQQR
HLA-C07:02	DKSRSSRSP
HLA-B07:02	GPGKGIPVGGG
HLA-C07:02	GAKTKKKKN
HLA-B07:02	GPEEEEEEEED
HLA-DRB101:0	LGKLILLSTQNGSI
HLA-DRB101:0	KEDFIQQRNVSCI
HLA-DRB101:0	NEVLGRELLLLVQ
HLA-DRB101:0	PQGDKSRSSRSPI
HLA-DRB101:0	SPGIPAGAKTKKK
HLA-DRB101:0	LSTPGPGKGIPVG
HLA-DQA101:0	SKKAGPEEEEEEE
HLA-C03:03	AASVTKGTL
HLA-B15:01	LQQQQQVVQL
HLA-A02:01	ILGKLILLST
HLA-C07:02	VKEDFIQQR
HLA-C07:02	GAKTKKKKN
HLA-B07:02	PPQQQQQQQPP
HLA-A01:01	EEEEEEEEEEE
HLA-DRB101:0	LGKLILLSTQNGSI
HLA-DRB101:0	KEDFIQQRNVSCI
HLA-DQA101:0	PGRTTAASVTKGT
HLA-DQA101:0	TRISKMELQQQQ
HLA-DRB101:0	SPGIPAGAKTKKK
HLA-DRB101:0	PQQQQQQQPPC
HLA-DQA101:0	EEEGEEEEEEEEEI
HLA-A02:01	ILMNCRMYS
HLA-C06:02	SRDQMFREQ
HLA-A02:01	SMPCWTARRLI
HLA-DRB114:5	PNALTLILMNCRN
HLA-DRB114:5	SSSMPCWTARRL
HLA-DRB113:0	KGLDWVKEEAPD

HLA-C08:02	ARDEAEWNI
HLA-A24:02	VRATGGFLF
HLA-B35:01	LPPPKMPPPR
HLA-DRB101:0	SIKAVRATGGFLF
HLA-DRB101:0	TTLTPLPPPKMPP
HLA-DRB101:0	WYAEHRGRARD
HLA-B39:01	IHIWAKFSAI
HLA-C12:03	RTRPKPCTW
HLA-C12:03	NATTTSQPSRM
HLA-B39:01	GPLLLLLLVL
HLA-C12:03	MRFQPGDNC
HLA-DRB116:0	RLQDWIHIWAKF
HLA-DRB116:0	PLLLLLLVLPPRAL
HLA-DQA101:0	LLSAVKLLCMRFC
HLA-DQA101:0	SNQKRGVKVLKQ
HLA-DQA101:0	TTDNATTTSQPSF
HLA-A02:02	YQVAEACSI
HLA-C03:04	SASPRSSSSF
HLA-C03:04	IPYRPNPPPPL
HLA-A02:02	RGRCGKDWL
HLA-C16:01	QGDKSRSSR
HLA-DRB111:0	SQTPLLRSRALVK
HLA-DRB111:0	PPLSFLQVVRPTC
HLA-DQA105:0	ACSIWSPGSVSTG
HLA-DRB111:0	LWQRLRDAFPED
HLA-DQA105:0	GDKSRSSRSPPGK
HLA-A02:02	FLAQEILRL
HLA-A02:02	YVHIITRFPL
HLA-A02:02	ITSENPDEV
HLA-B44:03	NEHGSYRYF
HLA-A02:02	RIIERLLRSPI
HLA-B44:03	AETPARDGG
HLA-B44:03	HERPVLPPH
HLA-DRB111:0	YSRIIERLLRSPITS
HLA-DRB111:0	QDLPYVHIITRNL
HLA-DRB111:0	LPPNIRELLPFRTV
HLA-DRB111:0	YRYFLTGLGRLIEL
HLA-DQA105:0	TPARDGGKAAAS
HLA-DRB111:0	QLIMTFHERPVLP
HLA-DRB111:0	HVIAARITSENPD
HLA-C07:01	YYRPRPRRY
HLA-B08:01	LWRCRSTRL
HLA-C07:01	ERQRLAREN
HLA-A11:01	RQQQQQQQQ
HLA-C07:01	GSGSGSGSG
HLA-DRB107:0	QRERGLWRCRST
HLA-DRB107:0	RGRSTYYRPRPRF

HLA-DQA105:0	SGSGSGSGGSSSS
HLA-DQA105:0	KARKQMERQRLA
HLA-DQA102:0	RKQQQRQQQQQ
HLA-A68:01	HAAALGCK
HLA-A68:01	HSAAGWPPDQK
HLA-B08:01	CLCTRWSCL
HLA-C07:02	DKSRSSRSP
HLA-B08:01	FLLLLLLLLP
HLA-B08:01	GKGGGGGGGE
HLA-DQA105:0	RDDAAMRAHAA
HLA-DRB107:0	IKTSRVFIRDCAW
HLA-DQA105:0	QSHLHSAAGWPI
HLA-DRB107:0	PFLLLLLLLLLPRGI
HLA-DQA105:0	KGGGGGGGGESO
HLA-DRB107:0	PPQGDKSRSSRSI
HLA-C06:02	SRKGRSRGA
HLA-B18:01	RQQQQQQQRO
HLA-DQA102:0	QQQQRQQQQQ
HLA-DQA102:0	SGGSGKLPSRKGI
HLA-B58:01	WSSSSRPW
HLA-A11:01	STYYRPRPRR
HLA-DRB116:0	RSTYYRPRPRRYV
HLA-DRB116:0	TRSFWSWSSSSRI
HLA-A24:02	IYFDFYDLLF
HLA-A26:01	MSWRGRSTYY
HLA-DRB112:0	IIVILLLVDVVYIFL
HLA-DRB114:5	GRSTYYRPRPRRY
HLA-C07:01	WRCSVPRGT
HLA-C07:01	SSGSSSSSN
HLA-DRB101:0	GAELGEAGAARP
HLA-DRB101:0	SSSGSSGSSSSSNO
HLA-B08:01	LAKRVRVCL
HLA-C07:01	LRFGTWTQAL
HLA-C08:02	KAQEIYEKI
HLA-DRB103:0	LSEILRLAKRVRVO
HLA-DRB103:0	GSPVTTSAPLRRIS
HLA-DQA105:0	LELNFHKAQEIYEI
HLA-C07:01	FRELPLRSWSL
HLA-DRB107:0	NPAGFRELPLRSV
HLA-DQA102:0	QQQQQQQQQQ
HLA-A02:01	LLCSFRHPV
HLA-B44:02	QEHGEATRDW
HLA-B44:02	CEKEAAPGW
HLA-A02:01	KMMRCKRYQT
HLA-B44:02	VEPAAAAAAAI
HLA-A23:01	VFVRPNSCM
HLA-DRB107:0	LLCSFRHPVSTRLI

HLA-DQA102:0	YVEPAAAAAAIS
HLA-DQA102:0	LPALAGAAAAHA
HLA-DRB107:0	KLKVFVRPNSCM
HLA-DRB107:0	ACRAAGAHGSRG
HLA-DRB107:0	TKSPCESNNKKM
HLA-C12:03	YQMRPSIIFL
HLA-C08:02	WIDSLQAFSL
HLA-C12:03	LGIPHVACY
HLA-C08:02	FEDSSSSGE
HLA-C12:03	LSAEAPTSPCL
HLA-C12:03	SLSPSSSDCLV
HLA-A03:01	HTTTTTTNR
HLA-C12:03	TPKKKKKDP
HLA-DQA105:0	ASSNSSGSLSPSSS
HLA-DQA105:0	MVWLQYGQAGK
HLA-DQA105:0	SQQPLSAEAPTSP
HLA-DQA105:0	TLRWLLGIPHVAC
HLA-DQA101:0	QLMNTWIDSLQA
HLA-DQA105:0	TVFEDSSSSGESSP
HLA-DQA105:0	HTTTTTNRPGTTP
HLA-DRB113:0	PKKKKKDPNEPQ
HLA-A31:01	RMSWRGGCCR
HLA-A31:01	KLMLLRLNLR
HLA-A31:01	RTPTWTPR
HLA-A02:01	FIMVGVQGL
HLA-C07:01	ERGQQPSCL
HLA-A31:01	ILGEPDPHQR
HLA-A02:01	RQQEAPPPPPA
HLA-DQA103:0	SGSWETLWAEEE
HLA-DRB104:0	QKKLMLLRLNLRK
HLA-DRB104:0	RHSSFIMVGVQG
HLA-DRB104:0	KRPVFILGEPDPH
HLA-DRB104:0	QSLLATCVGSISEL
HLA-DQA103:0	EKQMREEAERTR
HLA-DQA103:0	APPPPPATTQNY
HLA-C12:03	AAAAAAAYL
HLA-C12:03	DACGRRPQL
HLA-C12:03	LKHIKKRPR
HLA-C12:03	QQEEEEEED
HLA-DQA105:0	QQEEEAAAAAAA
HLA-DQA105:0	QLGLRAGIPPTAL
HLA-DRB111:0	HSVQPLLKHIKKR
HLA-DRB111:0	YHHHHHHHHHH
HLA-DQA105:0	QEEEEEEDSRDEG
HLA-B44:02	MELQQQQQV
HLA-C15:02	RSTRLQESQ
HLA-B51:01	LPQQQQQPP

HLA-C05:01	QQQQHQQQQ
HLA-C05:01	QPQQQQQQP
HLA-DRB107:0	QRERGLWRCRST
HLA-DQA101:0	TRISKMELQQQQ
HLA-DQA102:0	QQRQQQQLPQQ
HLA-DQA102:0	QPQAPQQPQQQ
HLA-DQA102:0	QQQQQHQQQQ
HLA-A30:02	SSSSSSSSGY
HLA-C07:01	WRCRSTRLQ
HLA-B15:03	VGIHLSLSI
HLA-B15:03	QQQHQQQQQ
HLA-C07:01	QRQQQQQQQ
HLA-B35:01	QPQQQQQQPP
HLA-C07:01	ETTTNKQTS
HLA-B15:03	PQQQQQQQPQV
HLA-DQA105:1	SGGYKGGSSSSSS
HLA-DRB111:0	RGLWRCRSTRLQ
HLA-DRB109:0	ICLVGIHLSLSIGLK
HLA-DQA105:1	QPQQQQQQPPP
HLA-DQA103:0	PQQQQQQQPQV
HLA-DQA105:1	VNKETTVETKETT
HLA-DRB111:0	RQQQQQQQQRQ
HLA-DRB111:0	RQQQQQQQQQQ
HLA-DQA105:1	QQQQQHQQQQ
HLA-A68:02	ETYRLISTIA
HLA-A68:02	QVTYLASPA
HLA-C08:02	GEEEEEEYE
HLA-DRB101:0	ICLRETYRLISTIAI
HLA-DRB101:0	RTQVTYLASPARQ
HLA-DQA101:0	GCAWTLEGEEEE
HLA-DRB101:0	VVQQQQQQQQQ
HLA-C08:02	ILSVIGEFL
HLA-C08:02	TGDEEEEDG
HLA-DRB101:0	CKIILSVIGEFLQPS
HLA-DQA101:0	EIQEVQITGDEEE
HLA-DRB101:0	VVQQQQQQQQQ
HLA-B07:02	LPSKFKNSM
HLA-C07:02	IYDDDVSPF
HLA-C07:01	RRYQKKRQP
HLA-C07:01	LRQQQQLL
HLA-B07:02	QPSQQQLLQEL
HLA-B07:02	AAAAAAAAA
HLA-C07:02	DKSRSSRSP
HLA-A03:01	LQQQQQQQQI
HLA-DQA104:0	SSPSAAAAAAAAA
HLA-DRB108:0	AGCSFAIRRYQKK
HLA-DRB108:0	KLPSKFKNSMRSL

HLA-DQA101:0	RQQQQLLSAIIEA
HLA-DQA104:0	IYDDDVSPFLEGL
HLA-DQA101:0	GSMEASVQQPS
HLA-DQA101:0	LQLQLQQQQQ
HLA-DRB108:0	GDKSRSSRSPPG
HLA-A02:01	WLLDLTPGL
HLA-C07:01	MRRNFSMGM
HLA-B07:02	LPSKFKNSM
HLA-A02:01	ALPACVVCL
HLA-B07:02	VPTRARCCL
HLA-B07:02	EPCGMSRSSL
HLA-A02:01	AQQQQQHPV
HLA-A02:01	MLVKKEEEEES
HLA-C07:02	DKSRSSRSP
HLA-DRB108:0	LKMRRNFSMGM
HLA-DRB108:0	KLPSKFKNSMRSL
HLA-DRB108:0	LQFVVMACPPKA
HLA-DQA101:0	LSRNQLSALPACV
HLA-DQA104:0	SGGSAGAETAVP
HLA-DQA101:0	PCGMSRSSLQTS
HLA-DRB108:0	KEEEEESHRRIKKL
HLA-DRB108:0	AQQQQQHPVLH
HLA-DRB108:0	GDKSRSSRSPPG
HLA-DQA101:0	RRRQQQQQQQ
HLA-A02:01	LLLGEPGQRV
HLA-B44:02	TEQIQCWPIY
HLA-B44:02	SEIYEAGAPM
HLA-A24:02	YYRPRPRRY
HLA-A02:01	SQFSTLKKT
HLA-B44:02	EEEEEEEEEEE
HLA-DQA105:0	SEIYEAGAPMAA
HLA-DQA105:0	TRCWGVMSANP
HLA-DRB104:0	ECGKAFSQFSTLK
HLA-DRB104:0	FWEIYKMTTEQI
HLA-DQA105:0	MSWRGRSTYYR
HLA-DQA105:0	EEEGEEEEEEEEE
HLA-B44:02	AEFRPSSRPW
HLA-B44:02	TEQIQCWPIY
HLA-A24:02	RYKRWPGAW
HLA-A02:01	RMRPASWHA
HLA-C05:01	ASSRRAAAT
HLA-B18:01	CELLNRFQT
HLA-A02:01	SQFSTLKKT
HLA-DQA105:0	SGSVHGGGRSAA
HLA-DQA105:0	RGPSSGTSAASSR
HLA-DQA105:0	RASADFGAAGSP
HLA-DRB104:0	ECGKAFSQFSTLK

HLA-DRB104:0	FWEIYKMTTEQI
HLA-DQA105:	MRPASWHAPRP
HLA-DRB104:0	PTTTTTTTTTTTT
HLA-DRB104:0	LLEKQACELLNR
HLA-B41:02	SEKEIKITL
HLA-C08:02	GPDSLGLTV
HLA-A32:01	ITVGPWASF
HLA-B14:02	THKRIHTGE
HLA-DQA105:	GRLPSSAGAAGA
HLA-DRB101:0	LQQFCISSSGKM
HLA-DRB111:0	SSHLTTHKRIHTG
HLA-DQA105:	DTAGRSGTGPSQ
HLA-B41:02	MEKLLRLLL
HLA-B41:02	SEKEIKITL
HLA-C08:02	VAGITGMSH
HLA-C08:02	PAAKPAAGA
HLA-DRB101:0	AILLQLSGAWRLL
HLA-DQA105:	AAKPAAGAPPAK
HLA-DRB101:0	LQQFCISSSGKM
HLA-DQA105:	PASASQVAGITG
HLA-B41:02	SEKEIKITL
HLA-B41:02	WEKRKHFYM
HLA-B41:02	EEVTTTTTI
HLA-C08:02	QVTCSLTPL
HLA-B14:02	RRKKRRTER
HLA-DRB101:0	RKHFYMKINNLR
HLA-DRB101:0	LQQFCISSSGKM
HLA-DRB101:0	GVHLQELLLVGA
HLA-DRB111:0	VTTTTTITEKHKL
HLA-DRB111:0	AKEWRNRRKKR
HLA-B41:02	SEKEIKITL
HLA-A32:01	TLLATQTPW
HLA-A32:01	ALPRWSSPW
HLA-B41:02	FERTVEALYV
HLA-B41:02	LEWLHLHL
HLA-C08:02	GGDVTELPA
HLA-B41:02	EEVTTTTTI
HLA-C08:02	CTDGSSRL
HLA-B41:02	SELFQGHKKV
HLA-B41:02	QEGLGYRSAPS
HLA-C08:02	INGSNLQPV
HLA-DRB101:0	KLEWLHLHLSRRL
HLA-DRB111:0	QVQLRNFLNLRK
HLA-DQA105:	TQEPVLGAPAAL
HLA-DRB101:0	LQQFCISSSGKM
HLA-DRB111:0	LQPVRSFLQPPK
HLA-DRB111:0	SELFQGHKKVSR

HLA-DRB101:0	KFACFERTVEALY
HLA-DQA105:0	RDQEGLGYRSAP
HLA-DQA105:0	LPAGRWSTSLTA
HLA-DRB111:0	VTTTTTITEKHKLN
HLA-DQA105:0	PASPSGPRGVPG
HLA-B07:02	SPPARRASM
HLA-B07:02	WPNFILRML
HLA-B35:01	SASWASCAW
HLA-C07:02	SRTLPSPIRCL
HLA-A03:01	EIDPPTIIK
HLA-DRB115:0	NFILRMLKRSWF
HLA-DQA101:0	PARRASMAALSL
HLA-DQA101:0	SLLICVAAVACSP
HLA-DRB115:0	YPTFSRTLPSPIRC
HLA-DRB113:0	KTKQEIDPPTIIKG
HLA-B07:02	APRGPRRSQ
HLA-B07:02	WPNFILRML
HLA-A03:01	ASSCSTSVTK
HLA-A03:01	AQWRRRSWK
HLA-C07:02	SRTLPSPIRCL
HLA-A03:26	KLAQQIQK
HLA-B07:02	SPAGACLLLLS
HLA-DRB115:0	QELIVILKSMPIIE
HLA-DRB115:0	NFILRMLKRSWF
HLA-DQA101:0	YTFSPAGACLLLL
HLA-DRB115:0	YPTFSRTLPSPIRC
HLA-DQA101:0	LAEDGVLSPLALT
HLA-DQA101:0	RSRCGHAICLRPS
HLA-DQA101:0	QHRTRLRQILDW
HLA-A03:01	KLRPVSSYK
HLA-B07:02	WPNFILRML
HLA-A03:01	AQWRRRSWK
HLA-C07:02	SRTLPSPIRCL
HLA-A03:26	KLAQQIQK
HLA-B07:02	PPAPPPPHL
HLA-B07:02	QPPSSSSST
HLA-C07:02	VKANQKKHL
HLA-B07:02	RGGGYGGDRS
HLA-DRB115:0	QELIVILKSMPIIE
HLA-DRB115:0	NFILRMLKRSWF
HLA-DRB115:0	YPTFSRTLPSPIRC
HLA-DQA101:0	LAEDGVLSPLALT
HLA-DRB113:0	DDIVKANQKKHL
HLA-DRB115:0	EEKLRPVSSYKPK
HLA-DRB115:0	ELVGYRQPPSSSS
HLA-DQA101:0	TSVPRSVPPAPPP
HLA-DRB115:0	RGGGYGGDRSS

HLA-B07:02	WPNFILRML
HLA-C07:02	SRTLPSPIRCL
HLA-A03:26	KLAQQIQK
HLA-DRB115:0	QELIVILKSMPIIE/
HLA-DRB115:0	NFILRMLKRSWFI
HLA-DRB115:0	YPTFSRTLPSPIRC
HLA-A03:01	MMANLTWSMK
HLA-B07:02	HPRGTGRCW
HLA-B07:02	WPNFILRML
HLA-A03:01	TTCCRTTCWK
HLA-A03:01	AQWRRRSWK
HLA-B07:02	HPHHHPRHA
HLA-C07:02	SRTLPSPIRCL
HLA-A03:26	KLAQQIQK
HLA-B35:01	MADGGGGGTG
HLA-DRB115:0	QELIVILKSMPIIE/
HLA-DRB115:0	NFILRMLKRSWFI
HLA-DQA101:(	DSKMMANLTWS
HLA-DRB115:0	YPTFSRTLPSPIRC
HLA-DRB115:0	SVMEYFPTTRFSH
HLA-DQA101:(	LAEDGVLSPLALT
HLA-DRB115:0	PHHLHHHHHPHI
HLA-DQA101:(	TTCCRTTCWKPT
HLA-DQA101:(	ADGGGGGTGAV
HLA-A02:05	KLFGGKKVSV
HLA-B50:01	RELPAAAAAAV
HLA-A02:05	MLWGQELL
HLA-A02:05	GIPECAVSL
HLA-A02:05	VLPAQFRA
HLA-DQA105:(	ARELPAAAAAAVI
HLA-DRB111:0	RLKKTAMKLFGG
HLA-DQA105:(	VAGPRGGIPECA\
HLA-DRB111:0	QEHIRFIDNMLW
HLA-DRB111:0	PLPTVLPAQFRA/
HLA-C07:01	AAAAAAAYL
HLA-DQA101:(	QQEEEAAAAAAA
HLA-A02:01	KLGRRCDMFQL
HLA-DRB115:0	RRCDMFQLRKTV
HLA-A02:01	GLGNLILKNEV
HLA-A02:01	GIPECAVSL
HLA-C07:01	RHDDDDDDI
HLA-C07:01	GRKFQQQTN
HLA-B08:01	EAKSPEKAKTL
HLA-A02:01	LILKNEVNGKP
HLA-DQA105:(	VAGPRGGIPECA\
HLA-DRB113:0	WKVMSILKGRKF
HLA-DRB113:0	NLILKNEVNGKLR

HLA-DRB113:0	LILKNEVNGKPRM
HLA-DQA105:0	DDDDDIRGCAVL
HLA-DQA105:0	EEAKSPEKAKTLD
HLA-A30:02	AAAPPAPAF
HLA-DQA101:0	AAAAAAAAAAPP
HLA-DQA101:0	SLAQQQQQQQ
HLA-DQA101:0	AGAEDVAAGGED
HLA-A02:01	TLFVGVYVV
HLA-C12:03	HANECAPVI
HLA-A02:01	MVVAGVFLL
HLA-C12:03	ISKENSKIL
HLA-C12:03	FSDSRIAL
HLA-C12:03	FCTTFGQHY
HLA-A02:01	GQIQFAVVGV
HLA-C12:03	KTSPLMKLI
HLA-C12:03	MKYPLCTFY
HLA-C12:03	TTIPLHLKL
HLA-C12:03	NEKARGKVI
HLA-C12:03	VVAEAGSGL
HLA-B38:01	QHQISASII
HLA-C12:03	TPCKMTSTM
HLA-B38:01	HHYKQLDL
HLA-B38:01	DHDNSAMKTL
HLA-C12:03	VPTTKTPAV
HLA-C12:03	NADNEQDLG
HLA-C12:03	HCFPCCTGS
HLA-A02:01	KLEKPETESCP
HLA-DRB111:0	RGKVIHQANLRH
HLA-DRB111:0	ALDLNYLTNKLKN
HLA-DRB111:0	MTPMRKTSPLMI
HLA-DRB111:0	NSAMKTLRSKMC
HLA-DRB111:0	GVFLLIRFHPLAEN
HLA-DRB111:0	TIPLHLKLTALAVT
HLA-DRB111:0	TKGVLISHHYKQL
HLA-DRB111:0	SGISSMAKLTVHII
HLA-DRB111:0	ASIITSTQKGMIKL
HLA-DRB111:0	FMAVKFSDSRIAL
HLA-DRB111:0	LFVGVYVVIEIARC
HLA-DQA105:0	SVNYTAVGQIQFA
HLA-DQA105:0	PKPPVVAEAGSGI
HLA-DRB111:0	HKLKISKENSKILK
HLA-DRB111:0	VEDGILKLRNADN
HLA-DRB111:0	QFFCTTFGQHYH
HLA-DQA105:0	PCCTGSGKSNVG
HLA-DRB111:0	TMPRVRKPKTTPT
HLA-DQA105:0	VPTTKTPAVTKPE
HLA-DRB113:0	KTEEIKLEKPETES

HLA-A02:01	TLFVGVYVV
HLA-C12:03	HANECAPVI
HLA-C12:03	SAMKTLRSM
HLA-A02:01	SLLGTCLILQI
HLA-A02:01	MVVAGVFLL
HLA-C12:03	ISKENSKIL
HLA-C12:03	FSDSRIAL
HLA-C12:03	MAMHNTKGRT
HLA-C12:03	FCTTFGQHY
HLA-A02:01	GQIQFAVVGV
HLA-C12:03	KTSPLMKLI
HLA-C12:03	MKYPLCTFY
HLA-C12:03	TTIPLHLKL
HLA-C12:03	NEKARGKVI
HLA-C12:03	WLHKRGKYI
HLA-C12:03	VVAEAGSGL
HLA-B38:01	QHQISASII
HLA-C12:03	TPCKMTSTM
HLA-B38:01	HHYKQLDL
HLA-C12:03	WLHGCPPPY
HLA-B38:01	DHDNSAMKTL
HLA-C12:03	VPTTKTPAV
HLA-C12:03	NADNEQDLG
HLA-C12:03	HCFPCCTGS
HLA-A02:01	KLEKPETESCP
HLA-DRB111:0	RGKVIHQANLRH
HLA-DRB111:0	QQWLIKLTSKQ
HLA-DRB111:0	RGKYIKTWRPRY
HLA-DRB111:0	ALDLNYLTNKLK
HLA-DRB111:0	MTPMRKTSPLM
HLA-DRB111:0	NSAMKTLRSKM
HLA-DRB111:0	SAMKTLRSMMG
HLA-DRB111:0	GVFLLIRFHPLAE
HLA-DRB111:0	TIPLHLKLTALAVT
HLA-DRB111:0	TKGVLISHHYKQL
HLA-DRB111:0	TCLILQITTGLFLA
HLA-DRB111:0	SGISSMAKLTVHI
HLA-DRB111:0	ASIITSTQKGMIK
HLA-DRB111:0	FMAVKFSDSRIAL
HLA-DRB111:0	LFVGVYVVIEIAR
HLA-DQA105:	SVNYTAVGQIQF
HLA-DQA105:	PKPPVVAEAGSG
HLA-DRB111:0	HKLKISKENSKILK
HLA-DRB111:0	VEDGILKLRNAD
HLA-DRB111:0	QFFCTTFGQHYH
HLA-DQA105:	PCCTGSGKSNVG
HLA-DRB111:0	TLRSMMGKWCR

HLA-DQA105:0	NLEWLHGCPPPY
HLA-DRB111:0	TMPRVRKPKTTP
HLA-DQA105:0	VPTTKTPAVTKPE
HLA-DRB113:0	KTEEIKLEKPETES
HLA-A02:01	YLDNRFFTL
HLA-A02:01	TLIGSSPHV
HLA-A02:01	YLAIWALSSL
HLA-A02:01	YLNNVIMWFM
HLA-A02:01	ALDNGLFIL
HLA-A02:01	SLNEHLQAL
HLA-A02:01	LLASNSCFI
HLA-C05:01	SSDEKDSVL
HLA-B44:02	EEEHISFALY
HLA-A02:01	FGPIWIWEV
HLA-A02:01	ALLITARCVV
HLA-C05:01	YSDAIGPREQW
HLA-B44:02	SESKQEEDEEY
HLA-B44:02	MESNRIESNRI
HLA-A02:01	HLIYAASSL
HLA-A02:01	SLYWWGVVPC
HLA-A02:01	KQVDVPYIA
HLA-A02:01	FLPLPFDI
HLA-A02:01	RLLNEPDGTFL
HLA-A02:01	FICDVGNQEEV
HLA-A02:01	KLLKCAQHKAI
HLA-B44:02	NETVFIIVF
HLA-B44:02	KEMNSELSL
HLA-C05:01	LLDLEKSHH
HLA-A02:01	VVAPSTPPPL
HLA-C05:01	HQDRSPAPT
HLA-B44:02	GERSGRSGSF
HLA-A02:01	FLDWTELKS
HLA-C05:01	SLDRCYSTPSM
HLA-A02:01	FISSCESTKTV
HLA-B44:02	KECERLQAMM
HLA-C05:01	GLDASQPSQ
HLA-C05:01	HDDSFMKIL
HLA-C05:01	RSDASRGQS
HLA-B44:02	AEVVVPCD
HLA-C05:01	EEDQGPPCP
HLA-A02:01	HLERAFSNQL
HLA-A02:01	VEIPPNITSV
HLA-A02:01	GLSGGAAPPP
HLA-C05:01	ASDVRRRGT
HLA-C05:01	RVGGGAALA
HLA-B44:02	NEQSAKTDV
HLA-C05:01	STSTSQEFY

HLA-B44:02	GEKPYAWKEC
HLA-C05:01	PAQGGNQPQ
HLA-A02:01	SSCCRPCCCV
HLA-C05:01	HQQQHQQQQ
HLA-C05:01	PQQPGPAKP
HLA-DQA104:	LTRVGGGAALAA
HLA-DRB108:0	DSFMKILRSKMG
HLA-DRB108:0	CERLQAMMTHL
HLA-DRB104:0	PKHLIYAASSLQS
HLA-DRB104:0	LDNRFFTLTVTSL
HLA-DQA104:	VRRRGTAAAAGP
HLA-DQA104:	GVGLSGGAAPPP
HLA-DRB108:0	RLIIGFISKQYVTS
HLA-DRB104:0	VIMWFMQISED
HLA-DRB104:0	SRSYLAIWALSSL
HLA-DRB108:0	GKLLKCAQHKAIP
HLA-DRB104:0	EGTYIHALDNGL
HLA-DRB108:0	CSQRKKMLEKAR
HLA-DRB108:0	VPYIAKHYNMSK
HLA-DRB108:0	LLITARCVVPALH
HLA-DRB108:0	KYNTLHLERAFSN
HLA-DRB108:0	LHTKKGKMALLA
HLA-DRB108:0	QEEVYQTAKAVR
HLA-DQA103:	FFSELAGLDASQP
HLA-DRB104:0	KVNLQSLKNNEQ
HLA-DRB104:0	YGKYLGINSDELV
HLA-DRB108:0	SFQHYRSVFYSFE
HLA-DRB108:0	PMNVSSVVKPSF
HLA-DRB104:0	EKEMNSELSLSH
HLA-DRB104:0	AEKEMNSELSLS
HLA-DQA104:	NELQNLSSAEVV
HLA-DRB104:0	KEDAFISSCESTK
HLA-DRB104:0	VFIIVFGESLLND
HLA-DRB104:0	AAKIHPSTSTSQE
HLA-DRB104:0	GMEWNGMESN
HLA-DRB108:0	IPPNITSVQLKM
HLA-DRB108:0	KPKPLVTPRTVV
HLA-DQA104:	VDSTLIGSSPHVE
HLA-DRB104:0	SLDRCYSTPSMY
HLA-DRB104:0	QEEDEEYSWDSG
HLA-DQA104:	ASRGQSGSRSAS
HLA-DRB104:0	RSGRSGSFLYQV
HLA-DRB108:0	RSLNEHLQALLTP
HLA-DRB108:0	CCCVRPVCGRVS
HLA-DRB104:0	MWYKNSSLLDLE
HLA-DRB104:0	KEKFLDWTELKS
HLA-DRB108:0	WKECGKAFNKSS

HLA-DRB108:0	SSDEKDSVLNIAR
HLA-DRB108:0	VWETFQQRFGPI
HLA-DRB108:0	GPPCPRLSRELLE[illegible]
HLA-DQA104:[illegible]	GKPQGPPAQGG[illegible]
HLA-DQA104:[illegible]	PPPQQPGPAKPP
HLA-DQA104:[illegible]	GRHQDRSPAPT[illegible]
HLA-DRB108:0	HQQQQQQQQQ
HLA-A02:01	YLDNRFFTL
HLA-A02:01	YLITSIAVLV
HLA-A02:01	YLAIWALSSL
HLA-C05:01	FVDISVQELL
HLA-A02:01	FLSEGWIRA
HLA-A02:01	SLLNMICQQV
HLA-A02:01	ALDNGLFIL
HLA-A02:01	SLNEHLQAL
HLA-A02:01	LLASNSCFI
HLA-A02:01	VLGWWFRQPV
HLA-A02:01	AVLALLAKV
HLA-B44:02	EEEHISFALY
HLA-A02:01	MLLMLSTRDV
HLA-A02:01	FGPIWIWEV
HLA-A02:01	FLLHTGMVHCL
HLA-B44:02	QDMCVLRGASW
HLA-B44:02	EETEAQHLY
HLA-A02:01	NMSNEFISL
HLA-B44:02	SESKQEEDEEY
HLA-A02:01	TVWSHWAAEV
HLA-A02:01	YVWASLAGEEL
HLA-B44:02	MESNRIESNRI
HLA-A02:01	ILKQLFIAIV
HLA-A02:01	HLNGLIVLRL
HLA-A02:01	HLIYAASSL
HLA-A02:01	KLLAGDECA
HLA-A02:01	SLYWWGVVPC
HLA-A02:01	KQVDVPYIA
HLA-C05:01	KVDHELDDMF
HLA-B44:02	AEVSHHFRELM
HLA-A02:01	RLLNEPDGTFL
HLA-C05:01	FASALHVSWLF
HLA-A02:01	FICDVGNQEEV
HLA-A02:01	KLLKCAQHKAI
HLA-B44:02	NETVFIIVF
HLA-C05:01	LLDLEKSHH
HLA-B44:02	NDNAPKFEW
HLA-C05:01	HSDATGPRE
HLA-C05:01	YYDYNFISF
HLA-B44:02	CEFYQNGDTY

HLA-A02:01	FQGAPKGYHKV
HLA-A02:01	KLQKEQLKEEL
HLA-A02:01	FAWDTGTEKL
HLA-A02:01	FLDWTELKS
HLA-A02:01	GIPANQHPL
HLA-C05:01	NKDFKGALC
HLA-B44:02	KECERLQAMM
HLA-C05:01	KKDYYNFMA
HLA-C05:01	GLDASQPSQ
HLA-C05:01	KKDFSDPLD
HLA-A02:01	HLERAFSNQL
HLA-A02:01	VEIPPNITSV
HLA-A02:01	NTYCEGVLV
HLA-C05:01	RACGVGSAE
HLA-B44:02	MEPSFTREN
HLA-B44:02	TERCAFRKHL
HLA-C05:01	RVGGGAALA
HLA-B44:02	NEQSAKTDV
HLA-B44:02	GEKPYAWKEC
HLA-C05:01	PAQGGNQPQ
HLA-A02:01	SSCCRPCCCV
HLA-B44:02	GEKTYKCEEC
HLA-B44:02	REKPYKCEEC
HLA-C05:01	GGSKGGCGC
HLA-B44:02	RGRPVPHVLW
HLA-C05:01	PQQPGPAKP
HLA-DRB108:0	LNGLIVLRLRHLN
HLA-DQA104:	LTRVGGGAALAA
HLA-DRB104:0	YYNFMASLNLRN
HLA-DRB108:0	CERLQAMMTHL
HLA-DRB104:0	PKHLIYAASSLQS
HLA-DRB104:0	LDNRFFTLTVTSL
HLA-DQA104:	VWSHWAAEVAV
HLA-DRB108:0	TERCAFRKHLGA
HLA-DRB108:0	RLIIGFISKQYVTS
HLA-DRB108:0	ILSSRLTQHKKIHT
HLA-DRB108:0	HVSWLFPWYRY
HLA-DQA104:	AYSAYVWASLAG
HLA-DRB104:0	SRSYLAIWALSSL
HLA-DRB108:0	GKLLKCAQHKAIP
HLA-DRB104:0	EGTYIHALDNGL
HLA-DRB108:0	CSQRKKMLEKAR
HLA-DRB104:0	EPSFTRENTLMF
HLA-DQA104:	WIRAEVPGSPAV
HLA-DRB108:0	AQQLRVFQGAP
HLA-DRB104:0	LAKVLQIDSSVSF
HLA-DRB108:0	SFTMTVPFRGRP

HLA-DQA104:0	ISFWDFVDISVQE
HLA-DRB104:0	KQLFIAIVTETEKC
HLA-DRB104:0	ANLMLLMLSTRD
HLA-DRB108:0	VPYIAKHYNMSKS
HLA-DRB108:0	KYNTLHLERAFSN
HLA-DRB108:0	LHTKKGKMALLA
HLA-DRB108:0	LANPFVYSLHNKD
HLA-DQA104:0	TYCEGVLVAPAGI
HLA-DRB104:0	SNEFISLSPGTPPS
HLA-DRB108:0	QEEVYQTAKAVR
HLA-DQA104:0	DDWTISGIPANQ
HLA-DRB108:0	RVLGWWFRQPV
HLA-DQA103:0	FFSELAGLDASQP
HLA-DRB104:0	VDDFYYDYNFISF
HLA-DQA104:0	AILYLITSIAVLVER
HLA-DQA104:0	RVCRACGVGSAE
HLA-DRB108:0	VSHHFRELMQLF
HLA-DRB108:0	AAGIMAHICRKDI
HLA-DRB104:0	KVNLQSLKNNEQ
HLA-DRB108:0	SFQHYRSVFYSFE
HLA-DRB104:0	TEAQHLYQSILPD
HLA-DRB108:0	LNMICQQVEAIKI
HLA-DRB104:0	FLLASTAQDGDKL
HLA-DRB108:0	TQTVHCFLLHTGN
HLA-DRB104:0	VFIIVFGESLLNDA
HLA-DQA103:0	EWTASPYQAAEC
HLA-DQA104:0	PPTSASRVAGTTG
HLA-DRB104:0	GMEWNGMESN
HLA-DRB108:0	IPPNITSVQLKMD
HLA-DRB104:0	MCEFYQNGDTYS
HLA-DRB104:0	QEEDEEYSWDSG
HLA-DRB108:0	RSLNEHLQALLTP
HLA-DRB108:0	KREYKLQKEQLKE
HLA-DRB108:0	SSHLTQHKTIHTG
HLA-DRB108:0	CCCVRPVCGRVS(
HLA-DRB104:0	MWYKNSSLLDLE
HLA-DQA104:0	PKFEWPSYEAELS
HLA-DRB104:0	KEKFLDWTELKSS
HLA-DRB108:0	WKECGKAFNKSS
HLA-DRB108:0	VWETFQQRFGPI
HLA-DRB104:0	SDELVGHSDATGI
HLA-DRB108:0	LDDMFGKSDRPA
HLA-DQA104:0	GKPQGPPAQGGI
HLA-DQA104:0	PPPQQPGPAKPP
HLA-DQA104:0	CGGSKGGCGSCG
HLA-A02:01	ILMLLAPIMV
HLA-A02:01	YLAIWALSSL

HLA-C05:01	YEDFVVNGF
HLA-A02:01	KVFELISLL
HLA-A02:01	FGPIWIWEV
HLA-B44:02	KELLINHQRIY
HLA-A02:01	HLIYAASSL
HLA-A02:01	SLYWWGVVPC
HLA-A02:01	KQVDVPYIA
HLA-A02:01	RLLNEPDGTFL
HLA-A02:01	FICDVGNQEEV
HLA-A02:01	KLLKCAQHKAI
HLA-B44:02	NETVFIIVF
HLA-A02:01	VLEQQRVGLAV
HLA-C05:01	LLDLEKSHH
HLA-A02:01	FLDWTELKS
HLA-B44:02	KECERLQAMM
HLA-C05:01	GLDASQPSQ
HLA-A02:01	RMGHQPPP
HLA-A02:01	HLERAFSNQL
HLA-A02:01	VEIPPNITSV
HLA-C05:01	STEKPTTPT
HLA-C05:01	RASLRTSHR
HLA-B44:02	NEQSAKTDV
HLA-B44:02	GEKPYAWKEC
HLA-B44:02	KEPSPTTTK
HLA-DRB108:0	CERLQAMMTHL
HLA-DRB104:0	PKHLIYAASSLQS
HLA-DRB104:0	RKVFELISLLNYTA
HLA-DRB104:0	FRKVFELISLLNYT
HLA-DRB104:0	WPYLTAETLKNR
HLA-DRB108:0	NGFNVLYNKKPV
HLA-DRB108:0	RLIIGFISKQYVTS
HLA-DRB108:0	LAPIMVISSSYTLI
HLA-DRB104:0	SRSYLAIWALSSL
HLA-DRB108:0	GKLLKCAQHKAIP
HLA-DRB108:0	CSQRKKMLEKAR
HLA-DRB108:0	VPYIAKHYNMSK
HLA-DRB108:0	PRASLRTSHRASP
HLA-DRB108:0	KYNTLHLERAFSN
HLA-DRB108:0	QEEVYQTAKAVR
HLA-DQA104:	EQQRVGLAVDM
HLA-DRB108:0	KELLINHQRIYTN
HLA-DQA103:	FFSELAGLDASQP
HLA-DRB104:0	KVNLQSLKNNEQ
HLA-DRB104:0	VFIIVFGESLLNDA
HLA-DRB108:0	IPPNITSVQLKMD
HLA-DRB104:0	MWYKNSSLLDLE
HLA-DRB104:0	KEKFLDWTELKSS

HLA-DRB108:0	WKECGKAFNKSS
HLA-DRB108:0	VWETFQQRFGPI
HLA-DRB104:0	KPTISTEKPTTPTE
HLA-DRB104:0	PTTTKSAPTTPKE
HLA-B57:01	KSVMQELEDY
HLA-DRB107:0	SVKEMLKSVMQE
HLA-B44:03	IEWMFIDFGM
HLA-B57:01	KSVMQELEDY
HLA-DQA102:	MFIDFGMDLAA
HLA-DRB107:0	SVKEMLKSVMQE
HLA-B57:01	KSVMQELEDY
HLA-B57:01	VNLETNVEELW
HLA-DRB107:0	LIFGAFAVGLKAV
HLA-DRB107:0	SVKEMLKSVMQE
HLA-C06:02	SRDGSMRTV
HLA-C06:02	GSPQLRRSV
HLA-DRB107:0	SGGSPQLRRSVS
HLA-DQA102:	RDGSMRTVIVQD
HLA-C06:02	KRGTYVIYV
HLA-DRB107:0	TYDIFYTAAKRGT
HLA-A24:02	SYIATLLYVF
HLA-B08:01	FIKRMFNSA
HLA-C07:01	NRQQCAHSL
HLA-C07:01	TRRGSSLHV
HLA-C07:01	YRNVHAVVFI
HLA-A24:02	HYAALALYF
HLA-C07:01	VREVCPHQL
HLA-B08:01	YLDNRFFTL
HLA-C07:01	SRMDLQQL
HLA-C07:01	LRHYNIIPVL
HLA-B08:01	CVKAHHYTA
HLA-A24:02	AYLFTVSSAF
HLA-B08:01	SLYEHLQAL
HLA-B08:01	SLNEHLQAL
HLA-C07:01	THFNASPSI
HLA-A24:02	YYLAARNSL
HLA-B35:02	LPISEFVL
HLA-A24:02	RFPSWVNF
HLA-C07:01	SRVRNVQPLL
HLA-B08:01	SILTKHKVI
HLA-A24:02	TWSPGLYLI
HLA-C07:01	FRHLCPSF
HLA-C07:01	SSTEREQSM
HLA-B08:01	NPRLRVIV
HLA-B35:02	YPSPVATSYL
HLA-C07:01	ALFSRTKLL
HLA-C07:01	THRGDINDI

HLA-A24:02	SFTALVAYSF
HLA-B08:01	LQRSRGAAA
HLA-B08:01	WLISRSGTI
HLA-A24:02	SYLPGFGSL
HLA-B08:01	DFRIRLLCV
HLA-C07:01	KAFSHSSTL
HLA-B35:02	SPDPSHTTL
HLA-A24:02	RYKGWRGGLKF
HLA-B08:01	LLVYRKWRA
HLA-A01:01	LSSLIDSSPY
HLA-B35:02	HPQLGAGIVL
HLA-A24:02	KYSEKETEF
HLA-A24:02	KYGYVSVPM
HLA-B35:02	TPVDTSTPV
HLA-B08:01	HQMQQTNVAL
HLA-B35:02	EAFSGSSVF
HLA-B35:02	HPDLPTHRPL
HLA-C07:01	ERCSDSDSL
HLA-A24:02	FYSFEEEHISF
HLA-B35:02	TASEGSTPL
HLA-C07:01	SHLARHRRI
HLA-C07:01	PREEQRKEF
HLA-B35:02	TPVDTSTL
HLA-B35:02	CPLPGTCHIL
HLA-A01:01	LSDSHTLSS
HLA-B35:02	SPDLAATL
HLA-B08:01	SSNLRHGSF
HLA-B35:02	SPLTASNTSL
HLA-B08:01	MALTGCWSV
HLA-B08:01	AAKKKLRNV
HLA-C07:01	YHMTQRGRC
HLA-B08:01	STLTKHKII
HLA-C07:01	IRHKRIHTG
HLA-A01:01	EMEDNYDYY
HLA-B08:01	SHLSRHHRL
HLA-A24:02	CFLTQLAGF
HLA-C07:01	DVHDDVWLL
HLA-C07:01	SLQGAARLV
HLA-C07:01	GSCSTAREM
HLA-C07:01	KRAGVSHLQ
HLA-B35:02	NPMNALTTKF
HLA-C07:01	LEGEKAQLL
HLA-B35:02	APASLQAM
HLA-A24:02	KYSVSNIATEI
HLA-A24:02	LYECSECQKAF
HLA-C07:01	IRRQLAQEKKL
HLA-C07:01	NSCPQVTEI

HLA-B08:01	CGKAFRQSA
HLA-A24:02	KVQSHIFSI
HLA-C07:01	GRGSPPTHRGM
HLA-C07:01	SHYSQTDRQ
HLA-A01:01	FQDVFFAY
HLA-B08:01	LPKVVQAM
HLA-A24:02	TYCINNEAL
HLA-C07:01	RRDSAVIGGVI
HLA-B35:02	NASAIQSIL
HLA-C07:01	RRSHTGERP
HLA-A01:01	MTDKCHDCW
HLA-B35:02	VPWGFTRAHL
HLA-B08:01	STLTKHKRI
HLA-B08:01	CIGCRFDDI
HLA-A24:02	EYNPAACCRI
HLA-A24:02	PYGCKECGKAF
HLA-B08:01	CGKTFAHNSAL
HLA-B35:02	NADFNDMQM
HLA-C07:01	ARIVLMDDV
HLA-A24:02	RIQSSLQAF
HLA-C07:01	VKNPRAEDL
HLA-A24:02	KQAEVWSGI
HLA-C07:01	GRSGCTHCS
HLA-C07:01	YRSYLTTHQ
HLA-C04:01	IFDKASESL
HLA-B08:01	KGKKKMSTA
HLA-C07:01	FHQKGSLIQ
HLA-B35:02	SPATSKCISF
HLA-C07:01	VSSCCKPQC
HLA-B08:01	STLTKHKKI
HLA-C07:01	KAFNQSSNL
HLA-C07:01	GSSGRKQQF
HLA-A24:02	PYKCNECGKVF
HLA-C07:01	SSSVCWKIL
HLA-C07:01	NRFVKKNSQ
HLA-A01:01	TTQASPGPQVY
HLA-B08:01	EGRGVKCAL
HLA-A24:02	PYKCKECGKAF
HLA-B08:01	FIQKSVFSV
HLA-C07:01	SQYSDIGSM
HLA-C07:01	GSTKPPPQQ
HLA-C07:01	ASVAKIQLH
HLA-A24:02	EYGTEAENNI
HLA-A01:01	WSHVRWKGRY
HLA-A24:02	CLSRVLGWWF
HLA-C07:01	RILNNARDK
HLA-A24:02	VIQETLWLL

HLA-C07:01	LINGVNRTQ
HLA-A24:02	PYECKECGKAF
HLA-C07:01	YDESGPSMV
HLA-B08:01	GPKVKGDV
HLA-C07:01	ARHKIIHTG
HLA-A24:02	IWKLSHSNPTI
HLA-B08:01	NLTTHKII
HLA-C07:01	TRHKIIHTG
HLA-A24:02	PYKCEECGKAF
HLA-C07:01	NQENCVGIL
HLA-A24:02	QFKDLGEENF
HLA-A01:01	ATSVLDMLA
HLA-B08:01	TPTEKPTI
HLA-B08:01	CRKKSARPAA
HLA-C07:01	KATSNEKDS
HLA-C07:01	ERIHTGEKP
HLA-C07:01	KLIKCQRLA
HLA-A24:02	PYKCEECGKDF
HLA-B08:01	HLTTHKRI
HLA-A24:02	EWNGMEWNGI
HLA-C07:01	SYNEEDPVD
HLA-A24:02	CQIGKVVPNPF
HLA-B35:02	IPTEKPTI
HLA-B08:01	MLNAFSQAP
HLA-B35:02	QPTCCHPSCCI
HLA-A24:02	VCVCVCVCVF
HLA-A01:01	KCEECGKAFY
HLA-B08:01	TTHKRIHTG
HLA-C07:01	IHTGEKPYP
HLA-A24:02	AVLCFILWI
HLA-B08:01	SPKRGLNSQ
HLA-C07:01	KVDIEGPDV
HLA-C07:01	MSQEFRGHR
HLA-C04:01	RFDSDAASPRE
HLA-A24:02	AVPAGLQW
HLA-C07:01	FSLQETCEA
HLA-A01:01	ATDVTGGK
HLA-A01:01	KVIHTGEKPY
HLA-A01:01	QSSNLTTHK
HLA-B35:02	LAGELGTCSSV
HLA-B08:01	CEECGKAFNL
HLA-B08:01	LTKHKIIHTG
HLA-C04:01	CEECGKAFK
HLA-B08:01	ECGKAFNQSA
HLA-C04:01	CEECGKAFN
HLA-C04:01	CEECGKAFS
HLA-A24:02	PYKCEECGKAY

HLA-DQA105:[illegible]	LQRSRGAAAASA
HLA-DRB111:0	LPGFGSLVKRVV[illegible]
HLA-DRB111:0	PGFGSLVKRVVN[illegible]
HLA-DRB111:0	YLPGFGSLVKRV[illegible]
HLA-DRB111:0	GFSMFSILTKHKV
HLA-DRB111:0	YVFHAVFSLIRW[illegible]
HLA-DRB111:0	ARLLALFSRTKLL[illegible]
HLA-DRB111:0	FSYRSYLTTHRRS[illegible]
HLA-DQA105:[illegible]	RRDSAVIGGVIAV
HLA-DRB111:0	LALGLLVYRKWR[illegible]
HLA-DRB111:0	VLCFILWIHSKKQ
HLA-DRB111:0	LRVIVELHRKVSS[illegible]
HLA-DRB111:0	SVFSTLTKHKIIHT[illegible]
HLA-DRB111:0	QSQYYLAARNSL[illegible]
HLA-DRB111:0	GLRFIQLKNPWS[illegible]
HLA-DQA105:[illegible]	CALVGDGAVGKT
HLA-DRB104:0	LSTFIHLSPDLAAT
HLA-DRB104:0	LTTKFVRTSTNKV
HLA-DRB111:0	PYKIFKNTSFIKR[illegible]
HLA-DRB111:0	APIPQGFSCLSRV
HLA-DRB111:0	PIPQGFSCLSRVL[illegible]
HLA-DRB111:0	QGFSCLSRVLGW
HLA-DRB111:0	WRGGLKFARKPL
HLA-DRB111:0	NQTSHLIRHKRIH
HLA-DQA105:[illegible]	GEEETAAAGSSG[illegible]
HLA-DQA105:[illegible]	EYHQPQAPASVA
HLA-DQA105:[illegible]	HPGLHGPQAVP[illegible]
HLA-DRB111:0	GAGIVLLLRAGFY[illegible]
HLA-DQA105:[illegible]	LPHRNASAAAKK[illegible]
HLA-DQA105:[illegible]	WLISRSGTIAEVG
HLA-DRB104:0	NRAVKSLQNASA
HLA-DRB111:0	GKAFRQSANLYV[illegible]
HLA-DRB111:0	QGKLIKCQRLAK[illegible]
HLA-DRB104:0	MLNAFSQAPST[illegible]
HLA-DRB104:0	LDNRFFTLTVTSL[illegible]
HLA-DRB111:0	GKVFTQNSHLAR
HLA-DQA105:[illegible]	MDLQQLGALAV[illegible]
HLA-DRB111:0	VAQMHSLFRHL[illegible]
HLA-DQA105:[illegible]	KKSARPAAGVGD
HLA-DRB104:0	GKTFAHNSALRA[illegible]
HLA-DRB111:0	GKAFKWSSTLTK[illegible]
HLA-DQA105:[illegible]	WAATTARNALV[illegible]
HLA-DRB111:0	QETLWLLSGVKK[illegible]
HLA-DQA105:[illegible]	PRLGPGWAGAH[illegible]
HLA-DRB104:0	GKAFNQSSNLTT[illegible]
HLA-DRB111:0	GKAFRQSSHLTT[illegible]
HLA-DRB104:0	GKVFNQSSNLTT[illegible]

HLA-DRB104:0	KGYLTDLNSMIPT
HLA-DRB111:0	WLLEFAAKRKQA
HLA-DRB104:0	SQCNVYFANLSN
HLA-DRB104:0	GKAFSHSSTLAKH
HLA-DQA105:C	MCRSLQGAARLV
HLA-DQA105:C	SLSSYPSPVATSYL
HLA-DQA105:C	IITHFNASPSITEST
HLA-DQA105:C	LQVFDAGSCSTA
HLA-DQA105:C	DKYGYVSVPMSS
HLA-DRB111:0	QLAVCQQRIRRQ
HLA-DQA105:C	VKAHHYAALALY
HLA-DRB104:0	EECGKAFNLSSTL
HLA-DQA105:C	MSGILAVGPMFV
HLA-DRB104:0	TSEVVRIQSSLQA
HLA-DRB111:0	CWKILLQFQCRN
HLA-DRB111:0	VEHYYRNVHAVV
HLA-DQA105:C	PISTASEGSTPLTS
HLA-DRB104:0	GKAFNQSSTLTKH
HLA-DRB104:0	TAYLFTVSSAFQG
HLA-DQA105:C	WAEYNPAACGRI
HLA-DQA105:C	DHAYHGHLSSLID
HLA-DRB111:0	NWWSQLTSHKK
HLA-DRB104:0	GKAFNQSSTLTKH
HLA-DRB111:0	GEVVLSRKVLLDG
HLA-DQA105:C	GCKECGKAFASLS
HLA-DQA105:C	PRVETEAPASLQA
HLA-DRB111:0	LFKLKPLSNKTATI
HLA-DQA105:C	NEIEAFSGSSVFQ
HLA-DQA105:C	PQQPGSAKPSAQ
HLA-DQA105:C	LLIAAGRTIKVWD
HLA-DQA105:C	FVLLVAGETKPSS
HLA-DRB111:0	RSLYEHLQALLTP
HLA-DRB111:0	CIGCRFDDISRLSS
HLA-DQA105:C	FQEKRAGVSHLQ
HLA-DRB111:0	SSTLTKHKRIHTG
HLA-DQA105:C	ESKGLAGELGTCS
HLA-DQA105:C	ANGVPASFVPKP
HLA-DRB104:0	TQFVRFDSDAAS
HLA-DQA105:C	PMSVIIVGVGNA
HLA-DRB104:0	EECGKAFNQSSTL
HLA-DQA105:C	LHLGTVAYGLDS
HLA-DQA105:C	TNEFHTSMALTG
HLA-DRB111:0	QVPPLGHLPKVV
HLA-DRB104:0	GKAFNESSNLTTH
HLA-DRB111:0	FIGFEGVKGKKK
HLA-DRB104:0	RQIWKLSHSNPTI
HLA-DRB111:0	SILTTHKRIHTGEK

HLA-DRB104:0	EECGKAFSNSSTL
HLA-DQA105:0	NARDKTGSSAQK
HLA-DRB111:0	KQSSHLTRHKIIH
HLA-DRB111:0	FNEQSHLSRHHR
HLA-DRB111:0	EECGKAFKHSSAL
HLA-DQA105:0	KQEYDESGPSMV
HLA-DRB104:0	NECGKVFSDRSA
HLA-DRB111:0	NNEALYDICFRTL
HLA-DQA105:0	ILGNIGICGAYGK
HLA-DRB111:0	RIRLLCVGLKHLL
HLA-DQA105:0	FLTQLAGFLANQ
HLA-DQA105:0	STLVTTSTGTSSS
HLA-DQA105:0	NLSPALGQMPIS
HLA-DQA105:0	PGSVVEASSNLR
HLA-DRB111:0	SSTLTTHKRIHTG
HLA-DQA105:0	PTQPGGTGLSDS
HLA-DQA105:0	QCAHSLFLASAE
HLA-DQA105:0	KCISFPGSSESPLS
HLA-DRB111:0	TKIPVTLSRVRNV
HLA-DRB111:0	TSVLDMLAKNLH
HLA-DQA105:0	SLHVEPSKTGVSH
HLA-DQA105:0	EFDPVPVGVTKV
HLA-DRB111:0	GKGFIQKSVFSVH
HLA-DRB111:0	GKAFNQSSKLTE
HLA-DRB111:0	QLLSPVFSIAPKN
HLA-DQA105:0	ENNIGTEGSVPSD
HLA-DRB111:0	EECGKAFYQSSIL
HLA-DRB111:0	SFQHYRSVFYSFE
HLA-DQA105:0	PESSPLTASNTSLL
HLA-DRB111:0	FNQSSTLARHKII
HLA-DRB111:0	HLRHYNIIPVLSD
HLA-DRB104:0	QPLHQMQQTNV
HLA-DRB111:0	NYDYYGQQRVQ
HLA-DQA105:0	DEKYSVSNIATEIK
HLA-DRB104:0	QDVFFAYPNRPD
HLA-DRB111:0	RVIKNYKRCFPVI
HLA-DQA105:0	EEAGAPVPEDAT
HLA-DQA105:0	MVDACASFLTEA
HLA-DRB111:0	SSTLTYHKVIHTG
HLA-DQA105:0	ELTQKAARIVLM
HLA-DRB111:0	GKCFHQKGSLIQ
HLA-DRB111:0	SGYHMTQRGRC
HLA-DQA105:0	FVVPWGFTRAHL
HLA-DRB111:0	KECGKAFNQSAH
HLA-DQA105:0	TQHQSIHAGVKP
HLA-DRB111:0	EECGKAFNRSSQ
HLA-DRB111:0	TDLNQHKKIHTG

HLA-DRB111:0	SILAKHKVIHTGE
HLA-DRB111:0	SILTKHKIIHTGEK
HLA-DRB111:0	SYDCIIHERIHTGE
HLA-DQA105:0	VFRRDAHKSEVA
HLA-DRB104:0	HDDVWLLSDAT
HLA-DQA105:0	KGVCQIGKVVPN
HLA-DQA105:0	THRPLTGWFPCV
HLA-DQA105:0	RGIVTDGRSGCT
HLA-DQA105:0	TTPVDTSTPVTTS
HLA-DRB111:0	HILVEHQKIHSGE
HLA-DRB111:0	PAYGKDPRVVTN
HLA-DQA105:0	KADVDVSGPKVD
HLA-DRB104:0	DSQYSDIGSMQ
HLA-DQA105:0	WNGMEWNGIE
HLA-DRB104:0	EQSMRKSSTFPR
HLA-DRB111:0	DVDLHLKGPKVK
HLA-DQA105:0	ARVWSVDKGQ
HLA-DQA105:0	SDSDSLAPPQHLI
HLA-DQA105:0	APPVTSARFPSW
HLA-DRB104:0	RSLNEHLQALLTP
HLA-DQA105:0	QNKGQTWSPGL
HLA-DRB111:0	QSSHYSQTDRQG
HLA-DQA105:0	SMENSCPQVTEI
HLA-DRB111:0	SNLTKHKIIHTGE
HLA-DRB104:0	SKPFSLQETCEAK
HLA-DRB111:0	SCCISSCCRPSCC
HLA-DQA103:0	KCHDCWAILEEY
HLA-DRB104:0	LYPINTPDQKEIV
HLA-DRB104:0	KPTISPEKLTTPTE
HLA-DQA105:0	SSCCRPSCCVSSC
HLA-DQA105:0	KLTIPTEKPTIPTE
HLA-DQA105:0	RRGVCVCVCVCV
HLA-A24:02	SYIATLLYVF
HLA-A24:02	CYSTPSMFF
HLA-B08:01	YLDNRFFTL
HLA-B08:01	SLNEHLQAL
HLA-A01:01	QSSSTPYLY
HLA-B08:01	KPRGRFLSG
HLA-C07:01	VRYFDNLSL
HLA-A24:02	YYLAARNSL
HLA-C07:01	IHFGHIWQK
HLA-A24:02	RFPSWVNF
HLA-A24:02	TWSPGLYLI
HLA-A24:02	FYMIVIPML
HLA-A24:02	KYMRNKQPFSW
HLA-B08:01	NPRLRVIV
HLA-B08:01	LQRSRGAAA

HLA-B08:01	WLISRSGTI
HLA-C07:01	GRIAEPRQS
HLA-A24:02	AFGCSSWLI
HLA-A01:01	LSSLIDSSPY
HLA-C07:01	VRVGAIQYA
HLA-B35:02	HPDLPTHRPL
HLA-C07:01	ERCSDSDSL
HLA-A24:02	FYSFEEEHISF
HLA-B35:02	FAIGGLMGTL
HLA-C07:01	MHFSNFTII
HLA-C07:01	KAFSQSSTL
HLA-B08:01	SSNLRHGSF
HLA-B35:02	SPLTASNTSL
HLA-B08:01	MALTGCWSV
HLA-B08:01	AAKKKLRNV
HLA-A24:02	AFLLAFPQCF
HLA-B08:01	TKRTRPYL
HLA-C07:01	TAHTQGVVV
HLA-C07:01	KRAGVSHLQ
HLA-C07:01	VRQEQESPCFL
HLA-A01:01	CTMKFSSWSY
HLA-C07:01	SVQPDILEL
HLA-B08:01	FPFYKNKA
HLA-A24:02	LYECSECQKAF
HLA-B08:01	VPKAQRHISA
HLA-B35:02	VPWGFTRAHL
HLA-A24:02	RIQSSLQAF
HLA-A24:02	SFQCNESGKAF
HLA-A24:02	LWQWSSCRHI
HLA-A24:02	KQAEVWSGI
HLA-C07:01	GSSGRKQQF
HLA-C07:01	SSMKPTPKE
HLA-B08:01	DPKGQQQM
HLA-A01:01	TTQASPGPQVY
HLA-C07:01	ASVAKIQLH
HLA-A24:02	IWKLSHSNPTI
HLA-C07:01	DYSEGGPLY
HLA-B08:01	NLTTHKII
HLA-A24:02	PYKCEECGKAF
HLA-A24:02	KFLLLQELV
HLA-B08:01	CRKKSARPAA
HLA-B35:02	QPTCCRPSCSI
HLA-C07:01	GRKEGEENQ
HLA-C07:01	HKAVDQPRQL
HLA-A24:02	AVPAGLQW
HLA-C07:01	GSLADAWTH
HLA-A24:02	QNLENKYLKDF

HLA-A01:01	ATDVTGGK
HLA-A01:01	ISDLTEQIG
HLA-C07:01	HKHEHDQGH
HLA-DQA105:	LQRSRGAAAASA
HLA-DQA105:	TQGVVVQPAAA
HLA-DRB111:0	YVFHAVFSLIRW
HLA-DQA105:	RGRFLSGPVSVSI
HLA-DRB111:0	LRVIVELHRKVSS
HLA-DRB111:0	QSQYYLAARNSL
HLA-DQA105:	VSIFAIGGLMGTL
HLA-DRB111:0	SNFTIITQRVSLSI
HLA-DRB111:0	FSNFTIITQRVSLS
HLA-DQA105:	GEEETAAAGSSG
HLA-DQA105:	EYHQPQAPASVA
HLA-DQA105:	TSSLTQAQVASV
HLA-DQA105:	HPGLHGPQAVP
HLA-DQA105:	LPHRNASAAAKK
HLA-DQA105:	WLISRSGTIAEVG
HLA-DRB104:0	LDNRFFTLTVTSL
HLA-DRB111:0	LWQWSSCRHIR
HLA-DQA105:	KKSARPAAGVGD
HLA-DQA105:	SSGSVVSSVPKA
HLA-DRB111:0	GMFNIAPHKVRV
HLA-DRB104:0	QSGPFQSSSTPYL
HLA-DRB111:0	PQCFYSKTKVMP
HLA-DQA105:	SRLSPGGSLADA
HLA-DRB104:0	GKAFSQSSTLTTH
HLA-DRB111:0	NKYLKDFEIVKRK
HLA-DRB111:0	WLLEFAAKRKQA
HLA-DQA105:	TRPYLFGAGCFSI
HLA-DRB111:0	KNLFSRKFPFYKN
HLA-DRB104:0	TSEVVRIQSSLQA
HLA-DQA105:	DHAYHGHLSSLID
HLA-DRB111:0	LFKLKPLSNKTAT
HLA-DRB111:0	FGCSSWLIRHQR
HLA-DQA105:	LLIAAGRTIKVWD
HLA-DQA105:	FQEKRAGVSHLQ
HLA-DRB111:0	IHFGHIWQKTLT
HLA-DQA105:	TNEFHTSMALTG
HLA-DRB104:0	GKAFNESSNLTT
HLA-DRB104:0	RQIWKLSHSNPT
HLA-DRB104:0	EECGKAFSNSSTL
HLA-DRB111:0	GPKYMRNKQPF
HLA-DQA105:	PGSVVEASSNLR
HLA-DRB111:0	SFQHYRSVFYSFE
HLA-DQA105:	PESSPLTASNTSL
HLA-DQA105:	DSWGRIAEPRQS

HLA-DQA105:0	LLPFSTGPPTECV
HLA-DRB104:0	PHPVRYFDNLSLG
HLA-DQA105:0	FVVPWGFTRAHL
HLA-DRB111:0	KGQQQMWQILC
HLA-DRB104:0	PSMFFELPDSFQ
HLA-DQA105:0	EDYSEGGPLYKN
HLA-DQA105:0	FQCNESGKAFNC
HLA-DRB111:0	DKKFLLLQELVEK
HLA-DQA105:0	THRPLTGWFPCV
HLA-DQA105:0	GRVCRIGFSVQP
HLA-DRB111:0	HILVEHQKIHSGE
HLA-DQA105:0	FSSWSYDKAKIDL
HLA-DQA105:0	SDSDSLAPPQHLI
HLA-DQA105:0	APPVTSARFPSW
HLA-DRB104:0	RSLNEHLQALLTP
HLA-DQA105:0	QNKGQTWSPGL
HLA-DQA105:0	PTCCRPSCSISSC
HLA-DRB111:0	SNKVKSDPHKAV
HLA-DQA105:0	LTEQIGETGKNLC
HLA-DQA105:0	KEEPCNIFPCPAF
HLA-DQA105:0	HDQGHGHQRGH
HLA-DQA105:0	NQNPPCPRLNGV
HLA-A24:02	SYIATLLYVF
HLA-A24:02	CYSTPSMFF
HLA-B08:01	YLDNRFFTL
HLA-C07:01	SRMDLQQL
HLA-B08:01	SLNEHLQAL
HLA-A01:01	QSSSTPYLY
HLA-B08:01	KPRGRFLSG
HLA-C07:01	VRYFDNLSL
HLA-A24:02	YYLAARNSL
HLA-A24:02	RFPSWVNF
HLA-A24:02	TWSPGLYLI
HLA-C07:01	FRHLCPSF
HLA-A24:02	KYMRNKQPFSW
HLA-B08:01	NPRLRVIV
HLA-B08:01	LQRSRGAAA
HLA-B08:01	WLISRSGTI
HLA-C07:01	GRIAEPRQS
HLA-A01:01	LSSLIDSSPY
HLA-B35:02	HPDLPTHRPL
HLA-C07:01	ERCSDSDSL
HLA-A24:02	FYSFEEEHISF
HLA-C07:01	LRFSVGPLC
HLA-C07:01	MHFSNFTII
HLA-B08:01	SSNLRHGSF
HLA-B35:02	SPLTASNTSL

HLA-B08:01	MALTGCWSV
HLA-B08:01	AAKKKLRNV
HLA-A24:02	AFLLAFPQCF
HLA-C07:01	HRGLHGPQA
HLA-C07:01	TAHTQGVVV
HLA-B35:02	FALDVDNRF
HLA-C07:01	KRAGVSHLQ
HLA-C07:01	VRQEQESPCFL
HLA-A01:01	CTMKFSSWSY
HLA-B08:01	FPFYKNKA
HLA-A24:02	LYECSECQKAF
HLA-B08:01	VPKAQRHISA
HLA-B35:02	VPWGFTRAHL
HLA-A24:02	RIQSSLQAF
HLA-A24:02	LWQWSSCRHI
HLA-A24:02	KQAEVWSGI
HLA-C07:01	DSSSKKRER
HLA-C07:01	GSSGRKQQF
HLA-A01:01	TTQASPGPQVY
HLA-C07:01	ASVAKIQLH
HLA-A24:02	IWKLSHSNPTI
HLA-C07:01	DYSEGGPLY
HLA-A24:02	KFLLLQELV
HLA-B08:01	CRKKSARPAA
HLA-A24:02	EWNGMEWNGI
HLA-B08:01	QDKEALTL
HLA-C07:01	SSSGGGGSG
HLA-B35:02	EPAPTTPEEPT
HLA-A24:02	AVPAGLQW
HLA-A01:01	ATDVTGGK
HLA-A01:01	ISDLTEQIG
HLA-C07:01	GSQEKQGKS
HLA-C07:01	LSGGAAPPP
HLA-DQA105:0	LQRSRGAAAASA
HLA-DQA105:0	GVGLSGGAAPPP
HLA-DQA105:0	TQGVVVQPAAA
HLA-DQA105:0	AAAAAGSSSSGG
HLA-DRB111:0	YVFHAVFSLIRW
HLA-DQA105:0	RGRFLSGPVSVSI
HLA-DRB111:0	LRVIVELHRKVSS
HLA-DRB111:0	QSQYYLAARNSL
HLA-DRB111:0	FSNFTIITQRVSLS
HLA-DRB111:0	SNFTIITQRVSLSI
HLA-DQA105:0	GEEETAAAGSSG
HLA-DQA105:0	EYHQPQAPASVA
HLA-DQA105:0	HPGLHGPQAVP
HLA-DQA105:0	LPHRNASAAAKK

HLA-DQA105:(	WLISRSGTIAEVG
HLA-DRB104:0	LDNRFFTLTVTSLI
HLA-DRB111:0	LWQWSSCRHIRR
HLA-DQA105:(	MDLQQLGALAVI
HLA-DRB111:0	VAQMHSLFRHLC
HLA-DQA105:(	KKSARPAAGVGD
HLA-DQA105:(	SSGSVVSSVPKAC
HLA-DRB104:0	QSGPFQSSSTPYL
HLA-DRB111:0	PQCFYSKTKVMP
HLA-DRB111:0	WLLEFAAKRKQA
HLA-DRB111:0	KNLFSRKFPFYKN
HLA-DRB104:0	TSEVVRIQSSLQA
HLA-DQA105:(	DHAYHGHLSSLID
HLA-DRB111:0	LFKLKPLSNKTATI
HLA-DQA105:(	LLIAAGRTIKVWD
HLA-DQA105:(	FQEKRAGVSHLQ
HLA-DQA105:(	TNEFHTSMALTG
HLA-DRB104:0	RQIWKLSHSNPTI
HLA-DRB111:0	GPKYMRNKQPFS
HLA-DRB111:0	ALTLLIQVPRIQPC
HLA-DQA105:(	PGSVVEASSNLRH
HLA-DRB111:0	TGLQVLPLPRPIPI
HLA-DRB111:0	SFQHYRSVFYSFE
HLA-DQA105:(	PESSPLTASNTSLL
HLA-DQA105:(	DSWGRIAEPRQS
HLA-DQA105:(	LLPFSTGPPTECVI
HLA-DRB104:0	PHPVRYFDNLSLG
HLA-DQA105:(	FVVPWGFTRAHL
HLA-DRB104:0	PSMFFELPDSFQH
HLA-DQA105:(	EDYSEGGPLYKNC
HLA-DRB104:0	REKLLSGNDSSSK
HLA-DRB111:0	DKKFLLLQELVEKI
HLA-DQA105:(	THRPLTGWFPCV
HLA-DRB111:0	HILVEHQKIHSGE
HLA-DQA105:(	FSSWSYDKAKIDL
HLA-DQA105:(	WNGMEWNGIES
HLA-DQA105:(	SDSDSLAPPQHLI
HLA-DQA105:(	APPVTSARFPSW
HLA-DRB104:0	RSLNEHLQALLTP
HLA-DQA105:(	QNKGQTWSPGLY
HLA-DQA105:(	LTEQIGETGKNLC
HLA-DQA105:(	PAPTTPEEPAPTT
HLA-DQA105:(	KSEGEGKPQGEG
HLA-A24:02	SYIATLLYVF
HLA-C07:01	YRHSQRASF
HLA-C07:01	SRMDLQQL
HLA-A01:01	QSSSTPYLY

HLA-A24:02	YYLAARNSL
HLA-C07:01	IHFGHIWQK
HLA-B35:02	FPEGIPTL
HLA-A24:02	RFPSWVNF
HLA-A24:02	YFFCKCVPGF
HLA-A24:02	TWSPGLYLI
HLA-C07:01	FRHLCPSF
HLA-A24:02	KYMRNKQPFSW
HLA-B08:01	NPRLRVIV
HLA-C07:01	VRTEGGSDV
HLA-A01:01	FLSALACYY
HLA-B35:02	LPSPLALTSL
HLA-B08:01	LQRSRGAAA
HLA-B08:01	WLISRSGTI
HLA-C07:01	LRNHQPRET
HLA-A24:02	YYLTENDIERW
HLA-A01:01	LSSLIDSSPY
HLA-B35:02	HPDLPTHRPL
HLA-C07:01	ERCSDSDSL
HLA-B35:02	FPGASYLV
HLA-A24:02	QWLDLSNNF
HLA-B08:01	SSNLRHGSF
HLA-B35:02	SPLTASNTSL
HLA-B08:01	MALTGCWSV
HLA-B08:01	AAKKKLRNV
HLA-A24:02	FYAPGSEPV
HLA-B35:02	LAVAPDYVPPL
HLA-A24:02	FYPGAGLGDL
HLA-C07:01	SLYNAKQML
HLA-A24:02	QYPVMLTAM
HLA-C07:01	KRAGVSHLQ
HLA-C07:01	VRQEQESPCFL
HLA-B35:02	WPASLLSVTNV
HLA-B35:02	APASLQAM
HLA-C07:01	FRICSPLPW
HLA-B08:01	FPFYKNKA
HLA-A24:02	LYECSECQKAF
HLA-B08:01	LPGSSFSKL
HLA-C07:01	LRCTVPPAV
HLA-C07:01	SRWVFSGM
HLA-B35:02	VPWGFTRAHL
HLA-C07:01	LHDPKNTHI
HLA-C07:01	FRTSSTCSC
HLA-A24:02	RIQSSLQAF
HLA-C07:01	SRQCRDVDY
HLA-C07:01	SHLEKSLGT
HLA-A24:02	KQAEVWSGI

HLA-C07:01	GSSGRKQQF
HLA-C07:01	TERETAMGM
HLA-A01:01	TTQASPGPQVY
HLA-C07:01	ASVAKIQLH
HLA-A01:01	DTDVVEPLCF
HLA-A24:02	IWKLSHSNPTI
HLA-B08:01	EVKQQLEQAL
HLA-B08:01	IPRGKEDQQAF
HLA-C07:01	VSKELERQN
HLA-A01:01	ATDVTGGK
HLA-A01:01	ISDLTEQIG
HLA-B35:02	VPGMGGDV
HLA-DQA105:0	LQRSRGAAAASA
HLA-DRB111:0	GLLYARLARAYRH
HLA-DRB111:0	YVFHAVFSLIRWK
HLA-DRB111:0	LRVIVELHRKVSSL
HLA-DRB111:0	QSQYYLAARNSLV
HLA-DQA105:0	CFPGEGAGSEASD
HLA-DQA105:0	GEEETAAAGSSGI
HLA-DQA105:0	GLGDLGGGALGP
HLA-DQA105:0	EYHQPQAPASVA
HLA-DRB111:0	GNEVLKMCRSLYI
HLA-DQA105:0	LPHRNASAAAKKI
HLA-DQA105:0	WLISRSGTIAEVG
HLA-DQA105:0	FSHEEIAMATVTA
HLA-DQA105:0	MDLQQLGALAVI
HLA-DRB111:0	VAQMHSLFRHLC
HLA-DQA105:0	IVAALTWPASLLS
HLA-DRB104:0	QSGPFQSSSTPYL
HLA-DQA105:0	RRQGAAFYAPGS
HLA-DQA105:0	ALPSWAFANLSSI
HLA-DRB111:0	WLLEFAAKRKQA
HLA-DRB111:0	LPGSSFSKLLLPYR
HLA-DRB111:0	KNLFSRKFPFYKN
HLA-DRB104:0	TSEVVRIQSSLQA
HLA-DQA105:0	DHAYHGHLSSLID
HLA-DQA105:0	PRVETEAPASLQA
HLA-DRB111:0	LFKLKPLSNKTATI
HLA-DQA105:0	HLEKSLGTGLSSIL
HLA-DRB104:0	AFAFEWTKTLPSF
HLA-DRB111:0	TTSRWVFSGMRF
HLA-DQA105:0	HCDLFPGASYLVT
HLA-DRB111:0	LAPVFRICSPLPW
HLA-DQA105:0	LLIAAGRTIKVWD
HLA-DQA105:0	FQEKRAGVSHLQ
HLA-DRB111:0	LRGYFFCKCVPGF
HLA-DRB104:0	TSCSWQSFRTSST

HLA-DRB111:0	IHFGHIWQKTLTV
HLA-DQA105:0	NDLSGPSVPGMG
HLA-DQA105:0	TNEFHTSMALTG
HLA-DRB104:0	RQIWKLSHSNPTI
HLA-DQA105:0	IPTLHFSMGELSTI
HLA-DRB111:0	GPKYMRNKQPFS
HLA-DRB104:0	AAYFGVYDTAKG
HLA-DQA105:0	DVRTEGGSDVIIT
HLA-DQA105:0	PGSVVEASSNLRH
HLA-DRB111:0	QYPVMLTAMEKI
HLA-DRB111:0	IVELQIILEKYLAVA
HLA-DQA105:0	PESSPLTASNTSLL
HLA-DRB111:0	IERWIKEHREYSTI
HLA-DQA105:0	LLPFSTGPPTECVI
HLA-DQA105:0	THCDLETSGTVTV
HLA-DQA105:0	FVVPWGFTRAHL
HLA-DQA105:0	KTTCFLSALACYYG
HLA-DQA105:0	ERETAMGMIIDV
HLA-DQA105:0	THRPLTGWFPCV
HLA-DRB111:0	HILVEHQKIHSGE
HLA-DRB111:0	EEKIFGRGSRQCR
HLA-DQA105:0	SDSDSLAPPQHLI
HLA-DQA105:0	APPVTSARFPSWY
HLA-DQA105:0	QNKGQTWSPGLY
HLA-DRB104:0	QALRIQDIELKSVT
HLA-DQA105:0	IPPKSEGLLIPRGK
HLA-DQA105:0	LTEQIGETGKNLC
HLA-DRB104:0	TKLDHIQEQLVSK
HLA-C05:01	LADLASPFH
HLA-C05:01	MIDMETEML
HLA-C05:01	KSDRIVNETV
HLA-C05:01	KSDFSKDVAV
HLA-A25:01	EVFPVDLMY
HLA-C05:01	SIDITSVL
HLA-B27:05	GRNSPAPAVR
HLA-B44:02	QELHVPSDKL
HLA-A25:01	EVKNSDCVLSF
HLA-B44:02	CEEDNGGCY
HLA-A25:01	MLAGAILGGAY
HLA-B44:02	GEYLPSKGV
HLA-C05:01	QQDVSQNTV
HLA-C05:01	FLSNPHFL
HLA-B44:02	YENAKLLSA
HLA-C05:01	SFDFLAQKL
HLA-C05:01	VTENKVKNL
HLA-C05:01	EPDHTTVNN
HLA-B27:05	GQLGWLLPN

HLA-A25:01	TVNPCSGLY
HLA-B44:02	LEIVNKQGH
HLA-DQA104:(	NTVSRGIAPAPAL
HLA-DQA104:(	LAFMLAGAILGG/
HLA-DRB104:0	NPHFLNADPVLA
HLA-DQA104:(	GRNSPAPAVRAA
HLA-DRB108:0	ETEMLKILSPLDRI
HLA-DRB108:0	VDLMYRPWKHA
HLA-DRB108:0	FDFLAQKLQFYQI
HLA-DRB108:0	LEIVNKQGHTKVL
HLA-DRB104:0	KSDFSKDVAVQV
HLA-DQA104:(	DALCEFDAVLADI
HLA-DRB108:0	NHYMEQILNVSH
HLA-DRB104:0	CYHLCLLSPSEPFY
HLA-DRB108:0	NETLYENAKLLSA
HLA-DRB108:0	PGQLGWLLPNQI
HLA-DRB104:0	LSWYNGTVNPCS
HLA-DRB108:0	LELTLAKVEKEKH
HLA-DRB108:0	VGNNVLVIDGQC
HLA-DQA104:(	RHSIDITSVLWND
HLA-DRB108:0	QRELLQHQELHV
HLA-DRB104:0	DHTTVNNSTSTQ
HLA-A02:01	MLSWVYFWI
HLA-A02:01	FLLGGWKAL
HLA-A02:01	ILNPISIFM
HLA-A02:01	RLRGFVDEV
HLA-B07:02	LPHLHLGLAV
HLA-A02:01	KVMALNSAEKV
HLA-B07:02	SPAAAAAAAV
HLA-C07:02	LRGHMVRYL
HLA-A02:01	LLLQADKAGL
HLA-B44:02	AEKATSDEKY
HLA-C07:02	VFKAHRIVL
HLA-B44:02	NEKDSVSNI
HLA-B07:02	MPTDGEKDHEL
HLA-A02:01	TVFSSLCLNLI
HLA-B07:02	KPPIPPLHQF
HLA-C07:02	FTHEQIMRRY
HLA-C07:02	VRGPQGPPE
HLA-A02:01	SVSNTATEI
HLA-C07:02	QRLTFLKQQ
HLA-B07:02	KPQGPPQQ
HLA-C07:02	ILTAHKRIH
HLA-A02:01	VIAHDSGDPPL
HLA-DRB115:0	RGHMVRYLMRSI
HLA-DQA101:(	AAAAAAAAVAVA
HLA-DRB115:0	HRIVLFASSGYFKI

HLA-DRB115:0	ATLMVMLSWVY
HLA-DRB115:0	SSLCLNLIPHHLYF
HLA-DQA101:0	AARRSVARLLLQA
HLA-DRB113:0	IKVMALNSAEKVT
HLA-DRB115:0	FTVILNPISIFMTH
HLA-DQA101:0	DSVSNTATEIKEG
HLA-DRB115:0	IPPLHQFSSWESR
HLA-DRB115:0	FHLPHLHLGLAVA
HLA-DQA101:0	VLLWGAAVFLLG
HLA-DRB115:0	FTHEQIMRRYGTI
HLA-DQA101:0	VSNTATEIKEGQC
HLA-DRB115:0	KAFKRSSILTAHKF
HLA-DQA101:0	ESLSAVADIIVRGF
HLA-DRB115:0	KFETARRLRGFVD
HLA-DQA101:0	YEQFRDLELRVIAI
HLA-DRB115:0	QQQLLATKEQRLT
HLA-DQA101:0	SASQERSLDEAM
HLA-DQA101:0	TATEIKEGQQSGT
HLA-DRB113:0	GPPQQGGNKPQ
HLA-B44:03	MENYVAQRY
HLA-A11:01	TTYIFWLAAK
HLA-B35:01	WPPALAMPM
HLA-A29:02	NFWFQPADY
HLA-B35:01	LPVGMTVRF
HLA-A11:01	KIFWEVFHK
HLA-B35:01	YPQPGNDGY
HLA-B35:01	LALTEVLAF
HLA-A29:02	GVLDHGYRY
HLA-A11:01	VVYALAEMLK
HLA-A11:01	SVPVPVHCFK
HLA-A11:01	KVAVTTTPFK
HLA-A11:01	VTSSYPVSMK
HLA-A11:01	SSGLALLPLK
HLA-A29:02	YSFHSEQFVY
HLA-A29:02	RVIANCHEY
HLA-B35:01	SAALGSLGY
HLA-A11:01	STAALPENAK
HLA-B35:01	NAWGHQHGY
HLA-B35:01	LAVADGLFM
HLA-A29:02	SFGCDRALY
HLA-A29:02	YAFRNPAIGY
HLA-A11:01	GVPTSFVPK
HLA-A11:01	KSLMSNHQK
HLA-A11:01	LLAGHNYMLK
HLA-A29:02	LYASHLADY
HLA-B44:03	RELQVGCIW
HLA-A11:01	TVSFLLQGK

HLA-A11:01	ASARGSLRK
HLA-A11:01	GVLWEQYLQK
HLA-A11:01	MMMGNLVMFR
HLA-A11:01	QVHDYLHSK
HLA-B35:01	NAAKMMLIM
HLA-A11:01	FIWLIRSLYK
HLA-A11:01	VLCDPMTPK
HLA-B35:01	LPAEVWETL
HLA-A29:02	ILAELLMGY
HLA-A11:01	ISLVLGIMAK
HLA-B44:03	GEIVMVHEHW
HLA-A29:02	HLHDPAVLY
HLA-B35:01	YAGLMVAIF
HLA-A29:02	LMQVLNTHY
HLA-A11:01	STDGECLVK
HLA-A11:01	RVADRPDIKK
HLA-B35:01	MPPAPVLHF
HLA-A11:01	YSKGRVLYK
HLA-A11:01	FIDNPVNFEK
HLA-B44:03	AELHLNNNEF
HLA-A11:01	GTSGVDCNHK
HLA-A11:01	KLAQRLHIHK
HLA-A11:01	KAFRCPTYFRR
HLA-B44:03	QEMGLLTFRDI
HLA-A11:01	LLYGLHHSK
HLA-A11:01	MLSSELICK
HLA-A11:01	TVPEAIRSNK
HLA-B35:01	VPQKHVTFY
HLA-B35:01	TPVAFLHNF
HLA-A11:01	MTATTPETVK
HLA-A11:01	FTLHSKLSK
HLA-B44:03	TELTEELGW
HLA-B35:01	CPNAHLPVL
HLA-A11:01	SLSSSREEAK
HLA-B35:01	LPLGVPPPQF
HLA-B44:03	LELLIRTGW
HLA-B35:01	NSMVGELVY
HLA-A11:01	STGRRQGSHHK
HLA-A11:01	AVMDQRPINY
HLA-A11:01	VTQRFRLSR
HLA-A11:01	AQSLKNLGFK
HLA-B35:01	HASSFNCNF
HLA-A11:01	KTKDVKNPEK
HLA-B35:01	NPCLVPYAL
HLA-B35:01	MPSHTHSSV
HLA-A11:01	KTVAALWSGDK
HLA-B44:03	KETDSVYGEW

HLA-A11:01	GSTPRTPSK
HLA-B35:01	SPDEAQAAH
HLA-A11:01	GTLVLGGGK
HLA-B35:01	TACTIQTAF
HLA-A11:01	AVVCCYTDK
HLA-B35:01	RAFLEQLPM
HLA-A11:01	HIRHARSCVYK
HLA-A11:01	SIGIDIPVPIK
HLA-A11:01	ATWKTSLSR
HLA-B35:01	RASITHVPF
HLA-A11:01	STIFLNGNHER
HLA-A11:01	QMHPVTNPAK
HLA-B35:01	MPWRLTGRSRY
HLA-B35:01	LVIQYTSQGRY
HLA-A11:01	VVGMLDPPCK
HLA-B44:03	AEIKNLKEQI
HLA-A11:01	FISGKRWAK
HLA-B35:01	FAFNMGSTT
HLA-A11:01	HTAREFSEEDK
HLA-A11:01	RTAFGAVCWR
HLA-A11:01	GNIDIMSLK
HLA-A11:01	MLWMGIQAK
HLA-A11:01	ESSMSSHMK
HLA-B35:01	CPGPPVGPIKF
HLA-A11:01	GTMFDCETCR
HLA-A29:02	LLWALSLLW
HLA-A11:01	RLPSNLHPIK
HLA-A29:02	LYIFILALM
HLA-A11:01	HIFKICHQHTK
HLA-B44:03	GEIDEQIERY
HLA-A11:01	STASTALCMAR
HLA-A11:01	SLLGKSNHK
HLA-B44:03	HEGCVCDAGF
HLA-A11:01	PSYIQSLRK
HLA-A29:02	QFNGCQKVY
HLA-A11:01	GILMNDVGK
HLA-B35:01	MAVAGGIMG
HLA-A11:01	TVITHHCCER
HLA-A11:01	QMHPVTTPGK
HLA-A11:01	RVFYVSQRVAK
HLA-A11:01	KSQAMRIIR
HLA-B44:03	AELLQDEYSGW
HLA-B44:03	REDIQNAAW
HLA-A11:01	GGMLRGIIK
HLA-A11:01	ELNHPATSYLK
HLA-A11:01	YVAQATHLR
HLA-B35:01	ISFRCNDPM

HLA-B35:01	LPVLAPSSS
HLA-A11:01	CVHQLLLQNK
HLA-A29:02	RVDGNCQIIGY
HLA-A11:01	CLLWACKVCK
HLA-A29:02	FVTRADKFQMY
HLA-A29:02	ALLTQIQRY
HLA-A11:01	KAFGDILHR
HLA-B44:03	REYWRRLW
HLA-A11:01	AQQDLFWDIK
HLA-B35:01	DVQTDSVTF
HLA-A11:01	ILAHFLCGTK
HLA-B35:01	LPVLLDFGT
HLA-B44:03	QEQLRHQERY
HLA-A11:01	AVCRALIEER
HLA-A29:02	PLRPILGPRKY
HLA-B35:01	FVVQLQDPW
HLA-B35:01	IPVPTPPQIAT
HLA-B35:01	LAMSAITVI
HLA-A11:01	ELMRYCTTK
HLA-A29:02	MMWGTLTICF
HLA-A11:01	RTLGLIDASR
HLA-B44:03	WETYGELSPW
HLA-B44:03	DENGNAPAL
HLA-B44:03	LEHLKNLAF
HLA-A11:01	STNICELCTCR
HLA-B44:03	DECSAAFLL
HLA-B44:03	CEISLADAGTY
HLA-B35:01	AIIQHHHPF
HLA-A29:02	KSLMGTARY
HLA-A29:02	YITEADGKEVY
HLA-B44:03	KEVWDKLWM
HLA-A11:01	IITAICHLR
HLA-B44:03	QEHGGWAVII
HLA-B35:01	MSRDTKLIM
HLA-B44:03	IENCGLLQI
HLA-A11:01	ASYAVILCCIR
HLA-A11:01	AVQKLLTGR
HLA-B44:03	WEVCRELW
HLA-C16:01	ITFEIGAPL
HLA-A11:01	KIHILQKHGK
HLA-A11:01	KGPLVMLNK
HLA-A29:02	LFWVGHMVM
HLA-B35:01	MAPQGRMGF
HLA-B44:03	EDITNFQGRW
HLA-B35:01	VPTAGVPSSLF
HLA-B35:01	CVNCSREAM
HLA-B35:01	FQGFAAAEF

HLA-B35:01	KPVHLRLSSF
HLA-B44:03	MEFVPRQLGL
HLA-A11:01	ALKEEVISRK
HLA-A11:01	VVSDAVWDVR
HLA-A11:01	QIHQRNHTEEK
HLA-A11:01	VVWWIRASNTK
HLA-B35:01	QAWESPAAE
HLA-A11:01	LVSRQCMEDIK
HLA-A11:01	YACRRYYR
HLA-B44:03	YEDSRGQRF
HLA-B35:01	LPGIFDDIH
HLA-B44:03	AESATCQAGA
HLA-B35:01	TPQPGCALVM
HLA-A11:01	GTILNWTER
HLA-B44:03	SELITETI
HLA-B35:01	HTSPETAHRLF
HLA-B44:03	GEASDLPRM
HLA-B44:03	AEICLHPPT
HLA-B35:01	YALRVNLSVVL
HLA-B44:03	IEIIYATTSVI
HLA-A29:02	HLYYYCSLH
HLA-A29:02	QIHKKRHCSSY
HLA-B35:01	MPNLKDKLH
HLA-B35:01	LAANMLLLL
HLA-B35:01	DGEEDPDCY
HLA-B35:01	VAVEVGDAA
HLA-B44:03	GESLGAFPV
HLA-A11:01	KTARLQDK
HLA-B44:03	LENGTLYFHI
HLA-B44:03	HEIKDVLVWNN
HLA-B35:01	LPLDQNGNNL
HLA-B35:01	YPFTSVPLGR
HLA-B44:03	GEWGLVGI
HLA-B44:03	SEVQRLEGEL
HLA-A11:01	RTCSECLAH
HLA-A11:01	SQHACTSGR
HLA-B35:01	EPVRSRQPINM
HLA-B35:01	MPSLLGQQG
HLA-B35:01	YNADMTTSVDF
HLA-A11:01	PSQPEGLCK
HLA-A11:01	RSDGDSDSPEK
HLA-B35:01	LATWLIHPS
HLA-B44:03	EEVASCDQEV
HLA-A11:01	MMTAILER
HLA-B44:03	LEFLSSTSH
HLA-A11:01	HSASPEPAPR
HLA-B44:03	VEISHRLRH

HLA-B44:03	LEAPGRLLI
HLA-B35:01	LRISSRPAF
HLA-B35:01	LPKISHLTGM
HLA-B35:01	FAVGEAWAPV
HLA-B35:01	MASFPLLL
HLA-B35:01	YACSSGHHEL
HLA-A11:01	ALGATEEEEK
HLA-B35:01	DAVSSCTGL
HLA-B35:01	QCIDEFEAM
HLA-A29:02	KMDPWSLLA
HLA-A11:01	KTVLDLTR
HLA-B44:03	QELHLALKQEH
HLA-B35:01	TVRLDESSM
HLA-C16:01	IAHLEQNGSPL
HLA-C04:01	SYDEDIMGQLV
HLA-C16:01	ISFCINGQPV
HLA-C16:01	VARDCGMTL
HLA-A29:02	SNNNTRITY
HLA-A11:01	RAQARDHATNR
HLA-B35:01	QAVGIGIF
HLA-B44:03	GCYHLHLAAW
HLA-A29:02	TMSNITAGH
HLA-A11:01	TVDDYRLWH
HLA-A11:01	TINWTLLEKQR
HLA-B35:01	IASLTVLEI
HLA-B44:03	RELHIETI
HLA-A11:01	HSDEQNDTQK
HLA-C16:01	CTDPGLMPL
HLA-B44:03	REGTRVQSM
HLA-B35:01	APNKCCAPSAI
HLA-A29:02	LTDSTTAPNF
HLA-A11:01	EVQEETTRREK
HLA-B44:03	NELASAAARGG
HLA-B35:01	CSADHSNLSF
HLA-B44:03	REGHTLIL
HLA-A11:01	PTKKCNGLEEK
HLA-C16:01	WTYFTQTGV
HLA-C16:01	FGYRSGGM
HLA-B35:01	IPPEHSEII
HLA-B35:01	MVLSIVLTSLL
HLA-B35:01	LPSVLVLNN
HLA-C16:01	VLPPPPRPV
HLA-B44:03	IENGNGTVRC
HLA-C16:01	STHDSGWTV
HLA-A11:01	DSNDSVEADK
HLA-B35:01	CPKMHISIW
HLA-B35:01	YCIDPNVLL

HLA-B35:01	LPSNSCPN
HLA-A29:02	SLCGGEPNF
HLA-A11:01	RHGEPPLIK
HLA-B44:03	GETACLDCQV
HLA-B44:03	QEEERCEQQL
HLA-C16:01	SSDPASGPSV
HLA-B44:03	KEVITVSCG
HLA-C16:01	WAKERTLTCL
HLA-B35:01	LQVNPNTAL
HLA-A11:01	SSQEQSRDGSR
HLA-B35:01	NVNDNNVL
HLA-C16:01	SSHNRVNACL
HLA-B35:01	LVSGKQHSLEL
HLA-B35:01	KPHGVAVNL
HLA-A11:01	TSQKNTLRKHR
HLA-B35:01	SPCMQGLLG
HLA-C16:01	FSLDDLAGPGA
HLA-B44:03	KQHLVDADGII
HLA-B44:03	REDEDVLEQ
HLA-C04:01	IGDDDEDIG
HLA-DRB107:0	LLSHIRHASSLSLS
HLA-DRB107:0	RRIFNMSRDTKLI
HLA-DRB107:0	HPATSYLKATHPE
HLA-DRB107:0	VVWWIRASNTKL
HLA-DRB107:0	HPFFKTINWTLLE
HLA-DRB107:0	EATWKTSLSRIVI
HLA-DRB107:0	RKSIEIIYATTSVIL
HLA-DRB107:0	VHHPSFLSYTVSF
HLA-DRB107:0	VTGHFTFTLHSKL
HLA-DRB108:0	RFTLKKLAQRLHII
HLA-DRB107:0	FQVYKLLYASHLA
HLA-DRB108:0	NLRTRLFSRHHHH
HLA-DRB108:0	ALRRVLTAYAFRN
HLA-DRB107:0	FQSMMTSTASTA
HLA-DRB108:0	RYACRRYYRLLQS
HLA-DRB108:0	QKVYTKSSHLKAH
HLA-DRB107:0	TLYFHISMSSSGQ
HLA-DRB108:0	CSLLAAYTRKTAR
HLA-DRB108:0	LALCLLVRRTRRH
HLA-DRB108:0	QKIFHINPRLPSNI
HLA-DRB107:0	VLFMMVLSIVLTS
HLA-DRB107:0	LREHTSWVLLAAI
HLA-DRB108:0	MPGFIWLIRSLYK
HLA-DRB108:0	LHCIRLSIPRVFYV
HLA-DRB108:0	LQTKLRISSRPAFN
HLA-DRB107:0	FFIVYALRVNLSVV
HLA-DRB107:0	GFFIVYALRVNLS

HLA-DRB108:0	DYFKKRLKNIFLKT
HLA-DRB108:0	YLLLLAKKARGKP
HLA-DRB107:0	KAFGDILHRSESV
HLA-DRB107:0	QQLVQDIASLTVL
HLA-DRB108:0	EADYVAQATHLR
HLA-DRB107:0	HGYRIQIHSPLGI
HLA-DRB108:0	FRCPTYFRRHVKT
HLA-DRB108:0	TTYIFWLAAKNR
HLA-DRB108:0	NARVIKWLYGLR
HLA-DRB107:0	HVPFYDLVSTRKK
HLA-DRB108:0	PARLRTLHNLVIQ
HLA-DRB107:0	VTQRFRLSRDRK
HLA-DRB108:0	KGLHKVWKKKLS
HLA-DRB108:0	QKTFYTLRAQAR
HLA-DRB107:0	TVEVQAVLSALLT
HLA-DQA106:0	VQIGKAGLAMSA
HLA-DRB107:0	IVFCRMLGYSKG
HLA-DRB107:0	ISPIKLHLSVSLSSS
HLA-DQA102:0	LLDWVGAPAFA
HLA-DRB107:0	HLRSSTPTTIFPIR
HLA-DRB107:0	AVLWLWSSHNR
HLA-DRB107:0	FVVCFTYFHIFKIC
HLA-DRB107:0	NNDRVIRWIQAI
HLA-DRB108:0	ISEVLRRKFALKEE
HLA-DRB107:0	HTHSSVQRTRSL
HLA-DRB107:0	HLKNLAFSPATM
HLA-DRB107:0	FDIYYVYSFHSEQ
HLA-DRB108:0	RYKLLLYGLHHSK
HLA-DRB107:0	MQVLNTHYVAP
HLA-DRB108:0	RGSLRKRSVPMA
HLA-DQA102:0	GESLGAFPVAAQ
HLA-DRB108:0	GTMKIHILQKHG
HLA-DRB107:0	FLLILALTEVLAFA
HLA-DRB108:0	CQVIHRLPMPNL
HLA-DRB108:0	GNIDIMSLKRML
HLA-DRB108:0	VGMTVRFRVAC
HLA-DQA102:0	WGNELASAAAR
HLA-DRB108:0	WRRLWKQNIWR
HLA-DRB108:0	HTNYVRNSRAVG
HLA-DRB108:0	ISLVLGIMAKLLPL
HLA-DRB108:0	RPSYIQSLRKAEK
HLA-DRB107:0	EIKLPVKWTVPEA
HLA-DRB108:0	PVNFEKMRMIAN
HLA-DRB108:0	LECWHQLQPRR
HLA-DRB107:0	LLLKYREGHTLILS
HLA-DRB108:0	FMPWRLTGRSRY
HLA-DRB108:0	MKQGLVRRVLEA

HLA-DRB108:0	QIERYRRVEKLRN
HLA-DRB108:0	GDSKAVQKLLTGI
HLA-DRB108:0	KSQAMRIIRTVGC
HLA-DRB108:0	WEVFHKLPLEKKI
HLA-DQA102:0	QLAAEAAARLEA(
HLA-DRB108:0	CNYILSLARLADLD
HLA-DRB107:0	IKITVVWLISIGIDI
HLA-DRB107:0	CHYQTIPPHILLAT
HLA-DRB107:0	KMDPWSLLAVLG
HLA-DRB108:0	REKIKKSLMSNHC
HLA-DRB108:0	TSQKNTLRKHRVV
HLA-DRB108:0	ALDFISGKRWAK(
HLA-DRB107:0	YFGLFSYCVGNM
HLA-DRB108:0	LLWACKVCKRKSV
HLA-DRB107:0	DRVEVYSRSAALG
HLA-DRB108:0	HKSLMGTARYMS
HLA-DRB108:0	HKAMETLRRSMS
HLA-DRB108:0	KGPLVMLNKPQG
HLA-DRB108:0	HHLPLRPILGPRKY
HLA-DRB107:0	LGWLRELSSLQGI
HLA-DRB108:0	KNTSIRIGPARRSS
HLA-DQA102:0	QSLFQGFAAAEFI
HLA-DRB107:0	AVADGLFMLVLP
HLA-DRB107:0	LPSLEFLSSTSHGT
HLA-DRB107:0	QLPSLEFLSSTSHC
HLA-DRB108:0	IITAICHLRERGLEI
HLA-DRB108:0	ALPKISHLTGMAT
HLA-DRB108:0	YGGHLVSRRTACT
HLA-DRB107:0	LERCVRAPSSGLA
HLA-DRB108:0	DPAVLYNLKERYA
HLA-DQA102:0	MLEASGDAALYA
HLA-DQA102:0	GITFEIGAPLEALD
HLA-DRB108:0	VKNLILELKPHGV
HLA-DRB108:0	KCQRAIIQHHHPI
HLA-DRB107:0	EHLYYYCSLHGIPI
HLA-DRB107:0	YQVHDYLHSKLCS
HLA-DRB108:0	NKELRTKRKFSLD
HLA-DRB108:0	TPLIYACSSGHHEI
HLA-DRB107:0	VCVLLWALSLLWS
HLA-DRB108:0	LGVEISHRLRHSN
HLA-DQA106:0	FQDMAVAGGIM
HLA-DRB107:0	PTPPQIATSTSLLA
HLA-DRB108:0	YRLWHTLTGHSG
HLA-DRB108:0	TVMVKYTSHYPQ
HLA-DRB108:0	MMMGNLVMFR
HLA-DRB107:0	DKYLKETCSVPVP
HLA-DRB107:0	KYLKETCSVPVPV

HLA-DRB107:0	WDKYLKETCSVP[illegible]
HLA-DRB107:0	TWDKYLKETCSVI
HLA-DRB107:0	FTWDKYLKETCS[illegible]
HLA-DRB108:0	MMTAILERLSTLS
HLA-DQA102:0	ANGIFSTAALPEN
HLA-DRB108:0	KGCYHLHLAAW[illegible]
HLA-DRB107:0	MRYCTTKDIILPF[illegible]
HLA-DRB107:0	SNDPVVYALAEM
HLA-DRB107:0	SKYLQVNPNTAL[illegible]
HLA-DRB108:0	NQIHKKRHCSSYL
HLA-DQA102:0	GYSSAVAPKTVA[illegible]
HLA-DRB108:0	PRTFLVSGKQHSL
HLA-DRB107:0	CDRALYQEHAKR
HLA-DRB107:0	FLGLLASYAVILC[illegible]
HLA-DRB108:0	QEQLRHQERYRK
HLA-DRB107:0	APCMRAFLEQLP
HLA-DRB107:0	SMSSHMKIRSQS
HLA-DRB107:0	HQDQIQQVVNH[illegible]
HLA-DRB107:0	LISFSYLLILKTVLD
HLA-DQA102:0	FLGEVGNAAKM[illegible]
HLA-DRB107:0	LHIETIAASSTPTP
HLA-DRB107:0	GSDLLLFWVGH[illegible]
HLA-DRB107:0	CVLGFWATVITHI
HLA-DRB108:0	HCFVTRADKFQ[illegible]
HLA-DRB107:0	ECSAAFLLSVMA[illegible]
HLA-DRB108:0	RYRIRVSTHDSG[illegible]
HLA-DRB108:0	KSMQLLKNLQCA
HLA-DRB107:0	YMLKIEYSANISS[illegible]
HLA-DRB107:0	LLGRIFALLLGNLY
HLA-DRB107:0	HLNNNEFTVLEA[illegible]
HLA-DQA102:0	VQEHGGWAVIIS
HLA-DRB108:0	AVRLKEKKLVKEV
HLA-DRB108:0	TLLREGPVLKISFR
HLA-DQA102:0	LGPGVPTAGVPS[illegible]
HLA-DRB107:0	LIWFTLTDSTTAP[illegible]
HLA-DQA102:0	RTGWLPEAAFLA
HLA-DRB108:0	QELHLALKQEHR[illegible]
HLA-DRB108:0	KLFVADLQRVIA[illegible]
HLA-DRB108:0	SRSAIRIGIYALHL[illegible]
HLA-DQA102:0	VLDENGNAPALL[illegible]
HLA-DRB108:0	RQHMLWMGIQ[illegible]
HLA-DRB108:0	EYIKGGMLRGIIK[illegible]
HLA-DRB107:0	IHLYQTFVVQLQ[illegible]
HLA-DRB107:0	NTIPLLYGILAHFL
HLA-DRB108:0	WEQYLQKYISPQ[illegible]
HLA-DRB107:0	PETKIWNSMVGE
HLA-DQA102:0	ITFRSGALPVLCD[illegible]

HLA-DRB107:0	GELSPWIEETRAL
HLA-DRB108:0	YADYAQSLKNLGI
HLA-DRB108:0	RNLSSFQIHQRNH
HLA-DRB108:0	MEQIGEVASVKA
HLA-DQA102:0	EEEYSAPLPVLAPS
HLA-DRB108:0	PIDISQLTKQHLVI
HLA-DQA102:0	PVLPGSHIAAEICL
HLA-DRB108:0	EWGLVGIPGKRSI
HLA-DRB107:0	FGYSWYKGERVD
HLA-DRB107:0	PQPGCALVMTRN
HLA-DRB108:0	HSEIITNMAHLGV
HLA-DQA102:0	AFEPLVANGVPTS
HLA-DRB108:0	AAMGRQMMPSI
HLA-DRB108:0	NEYKAEINKLKEQ
HLA-DQA102:0	GAGGASACSRCD
HLA-DQA102:0	RLLIGLSGVLTVGS
HLA-DRB108:0	YKTLRQIRQGNTK
HLA-DRB108:0	RMRVFKQLCYEC
HLA-DRB107:0	TVCWTYFTQTGV
HLA-DRB108:0	LTALKGALKEEVA
HLA-DQA102:0	VNGGKASPDEAC
HLA-DQA102:0	SVGSGSPYAYGVI
HLA-DRB107:0	TQHQLALVQSEV
HLA-DQA102:0	QHACTSGRVAVE
HLA-DQA106:0	SMLTAVSVARDC
HLA-DQA106:0	VRREFQLDLDEIE
HLA-DQA102:0	MAARTAFGAVCV
HLA-DQA102:0	RYNSSDPASGPSV
HLA-DRB107:0	GQRFMCSGPDKV
HLA-DRB107:0	LALREGHSKLVED
HLA-DRB108:0	AGQCMQNTPVA
HLA-DRB108:0	IENCGLLQILKLQC
HLA-DRB108:0	VHLHMPPAPVLH
HLA-DRB108:0	PKMHISIWQCLSA
HLA-DRB108:0	MKDRLNLPSVLVI
HLA-DQA102:0	SVPLGRGAPGTRA
HLA-DRB108:0	GIFALCMLRDGTL
HLA-DRB108:0	SFPLLLTLLTHCAG
HLA-DRB108:0	WEVCRELWCLSK
HLA-DQA102:0	GSGFKMGEASDL
HLA-DRB107:0	GSQEMGLLTFRD
HLA-DQA102:0	QFSDEGTCVAVM
HLA-DQA106:0	VKARLALLEVQEE
HLA-DRB108:0	DCETCRNRYRKLE
HLA-DRB108:0	MENYVAQRYIEN
HLA-DRB108:0	RTSTIFLNGNHER
HLA-DRB108:0	LTYKYMIHEDSLN

HLA-DRB107:0	GIFDDIHGSHGRC
HLA-DRB108:0	LRQYPPVTLVSRC
HLA-DQA102:(	LQNSDSSASAPNI
HLA-DRB107:0	RTCSECLAHHPRT
HLA-DRB108:0	VVCCYTDKILIACL
HLA-DQA102:(	ICEISLADAGTYYC
HLA-DRB108:0	KQAVCRALIEERG
HLA-DRB108:0	KENLHYTRVADRI
HLA-DQA102:(	SCCLDLGVPSISFC
HLA-DQA102:(	PFAFNMGSTTESI
HLA-DRB107:0	AVGIGIFPNLGLVI
HLA-DRB107:0	SKPTSHMTATTPI
HLA-DRB108:0	TEMLYGSTPRTPS
HLA-DRB107:0	REFGKDVLSSSGL
HLA-DQA102:(	IDMWSLGCILAEL
HLA-DRB108:0	ALSYLNEPVRSRQ
HLA-DQA102:(	EGFFSVPEDAVSS
HLA-DRB107:0	VYRTYNADMTTS
HLA-DRB107:0	LINPDTYCIDPNVI
HLA-DQA102:(	SHLYGPGETACLD
HLA-DRB107:0	DSEICGVVSDAVV
HLA-DRB108:0	QVGCIWTTQPSP
HLA-DRB107:0	NNTRITYALTEDT
HLA-DQA102:(	PVSRHSASPEPAF
HLA-DRB108:0	EDVLEQARRAHE
HLA-DQA102:(	LLSSSLGTSGVDCI
HLA-DQA102:(	QDEYSGWGIITW
HLA-DRB108:0	RDETLSCVGLSPK
HLA-DRB108:0	GKNPLQTCLSATN
HLA-DRB107:0	DPPCKEVTGSIQL
HLA-DRB108:0	SPNIVQDLNKSRE
HLA-DRB108:0	RNKKHHYRELPAI
HLA-DRB108:0	LKNKRNPCLVPYA
HLA-DQA102:(	AAQEQALGATEE
HLA-DRB108:0	REDIQNAAWHKH
HLA-DQA102:(	SQPEGLCKAASLT
HLA-DRB107:0	DHILTSELITETIRV
HLA-DRB108:0	LPKVLGLQQSCVN
HLA-DRB108:0	LEGILMNDVGKT(
HLA-DRB108:0	LGTASKEMRLNV
HLA-DRB108:0	GKEVYGRHQREIV
HLA-DQA102:(	REEWGTLDPAQC
HLA-DQA102:(	VNPFFGSLMDVT
HLA-DQA106:(	ITDLGLTLSYDEDI
HLA-DRB108:0	GECLVKAAQCGH
HLA-DRB108:0	QPADYNQCLQISI
HLA-DQA102:(	RSFGYRSGGMCG

HLA-DRB107:0	MLELETSQSPCM
HLA-DQA102:0	PIMGHGSQEPP
HLA-DRB108:0	ETKAKMTPTKKC
HLA-DQA102:0	KVEEEGEIVMVH
HLA-DQA102:0	HEGCVCDAGFVL
HLA-DQA102:0	ECGNNAQSPIDV
HLA-DRB107:0	FWFSDGSLSDKS
HLA-DQA102:0	DCYVCSGVPGRP
HLA-DRB108:0	SLQKYMEHHCP
HLA-DQA102:0	ANSLCGGEPNFL
HLA-DQA102:0	HTLPLPLGVPPPC
HLA-DQA102:0	LRNGKLEEAPPP
HLA-DQA102:0	VSDPKGPPAKIA
HLA-DRB108:0	AELQDWAKERTL
HLA-DQA102:0	ETDSVYGEWVPV
HLA-DRB107:0	RLSGSASRNHHG
HLA-DRB107:0	PIVDHTSPETAHR
HLA-DQA102:0	NDSVEADKSSPG
HLA-DQA102:0	PENLEDAPLLPSN
HLA-DQA106:0	VLLDFGTNNEELL
HLA-DRB107:0	FRELHTAREFSEE
HLA-DQA102:0	KQARDSSRHSAS
HLA-DQA102:0	EIQFDDIGDDDE
HLA-DRB108:0	LGARLHHYDERS
HLA-DQA102:0	QKQLSEEQNTGI
HLA-DRB108:0	ERCEQQLRREQE
HLA-C07:01	FRLPNQCVL
HLA-B08:01	YLDNRFFTL
HLA-B08:01	SLNEHLQAL
HLA-C07:01	WRLTNVTGI
HLA-B08:01	RLMKCRKSV
HLA-B57:01	KTHHAPGSTAW
HLA-C07:01	FRVLKTGQSM
HLA-A01:01	VLEETEAQHLY
HLA-C07:01	LRHFTGYHIEL
HLA-B57:01	YSTPSMFF
HLA-B57:01	RARGSYGF
HLA-B08:01	SLHSRTSST
HLA-B57:01	MNHEYMYW
HLA-B57:01	RLMQDEIAW
HLA-A01:01	YTDVNGYQI
HLA-A01:01	LSDSRTALK
HLA-B08:01	EMIQRLYGCL
HLA-B57:01	FAKYDVDPRKW
HLA-B57:01	KSVMQELEDY
HLA-C07:01	IRCEKEEVA
HLA-B57:01	KTFCEVDRSL

HLA-C06:02	LKSGHGKYL
HLA-C07:01	RKSPRGRRR
HLA-C07:01	QSSDRDLFV
HLA-C07:01	VYSVDQRDT
HLA-C07:01	SSDEKDSVL
HLA-C07:01	FRSVSAEEPLC
HLA-C06:02	CRPQCCQPV
HLA-C07:01	ISLSKPVEL
HLA-B57:01	HALDDGLF
HLA-B57:01	ISAAEGTL
HLA-B08:01	LVKPSGTLSL
HLA-B08:01	GLKPRQQQDL
HLA-B08:01	ELHTKKGKMDL
HLA-C07:01	IRGAGAASR
HLA-C07:01	SVNSPPPQP
HLA-C06:02	IAKVFQIDV
HLA-B57:01	RCYSTPSMF
HLA-B08:01	IPTEKPTI
HLA-C07:01	VSCGGSKGG
HLA-C07:01	TISTEKPTT
HLA-C06:02	DGYDPVQEN
HLA-C06:02	PAPPHHPTV
HLA-C06:02	PAKPPSQQP
HLA-C07:01	LSGGAAPPP
HLA-DRB107:0	LDNRFFTLTVTSL
HLA-DRB107:0	VISGLRHFTGYHI
HLA-DRB107:0	SSLPCISLSKPVEL
HLA-DQA101:	RVLKTGQSMTLL
HLA-DRB115:0	KIYIVGGYNWRLT
HLA-DQA102:	SACPTSVSSAAA
HLA-DRB107:0	RKWIKQYTGINA
HLA-DRB107:0	FQEMIQRLYGCL
HLA-DRB107:0	TEAQHLYQSILPD
HLA-DQA102:	NRCVTNSISAAE
HLA-DQA102:	KGDIRGAGAASR
HLA-DRB107:0	SLDRCYSTPSMF
HLA-DQA102:	GTNAVIAYTVQS
HLA-DRB107:0	KSQNRFYTDVNG
HLA-DRB107:0	SRIALKSGHGKYL
HLA-DQA102:	PCPRLNGVVME
HLA-DRB107:0	PGLVKPSGTLSLT
HLA-DRB107:0	HVYTFRSVSAEEP
HLA-DRB107:0	SSFSTAKTSTSLH
HLA-DRB107:0	SLDGCYSTPSMF
HLA-DQA102:	DLVSGLSPTVAVL
HLA-DRB107:0	KKTFCEVDRSLLC
HLA-DQA102:	NSTDEEVASSLTL

HLA-DQA101:0	RSLNEHLQALLTP
HLA-DRB107:0	KGKMDLLASNGC
HLA-DRB107:0	TSWPDLIAKVFQI
HLA-DQA101:0	SVKEMLKSVMQE
HLA-DQA101:0	MDEGTYIHALDD
HLA-DQA101:0	NRLMQDEIAWLF
HLA-DQA102:0	KEKGVGLSGGAA
HLA-DRB107:0	GFKLKEVSSVNSP
HLA-DRB115:0	MYWYRQDPGKG
HLA-DRB107:0	GHGKYLGINSDEL
HLA-DQA101:0	APPHHPTVIVPAF
HLA-DQA102:0	RCEKEEVATQSSE
HLA-DQA102:0	RGSYGFGGAGSG
HLA-DQA101:0	SSDEKDSVLNIAR
HLA-DRB107:0	PGLHSFTDHQRLI
HLA-DQA101:0	KPTTPTEETTISTE
HLA-DRB107:0	RDNCQYVYSVDC
HLA-DRB115:0	PPGVVRKSPRGRI
HLA-DQA102:0	SCGGSKGGCVSC
HLA-DQA102:0	CRPQCCQPVCCC
HLA-DQA102:0	KPTIPTEKPTIPTEI
HLA-DQA102:0	QQPGPAKPPSQC
HLA-C07:01	FRLPNQCVL
HLA-B57:01	VAIGPREQW
HLA-B08:01	YLDNRFFTL
HLA-B57:01	MSLLDFSF
HLA-B08:01	FILFHFLLI
HLA-B08:01	SLNEHLQAL
HLA-B08:01	RRVKRFLSV
HLA-C07:01	WRLTNVTGI
HLA-C07:01	FRVLKTGQSM
HLA-C07:01	SRFKMPKFKM
HLA-C07:01	FRDECELLR
HLA-B57:01	YSTPSMFF
HLA-B08:01	IVKRKREDL
HLA-B08:01	SLHSRTSST
HLA-C07:01	FSIDQKEDV
HLA-B57:01	KATKQLGF
HLA-B57:01	RLMQDEIAW
HLA-C07:01	IRNQNLEKKYL
HLA-A01:01	LSDSRTALK
HLA-C07:01	VRNAEKAMTAL
HLA-B57:01	VASGVPASF
HLA-B08:01	EMIQRLYGCL
HLA-B57:01	KAAALSTKI
HLA-C07:01	LNSGYGKYL
HLA-B57:01	KSVMQELEDY

HLA-C07:01	QSSDRDLFV
HLA-B08:01	DLEERTPAM
HLA-B08:01	RMLTHLTGSI
HLA-C07:01	LRSKMGKWC
HLA-C07:01	SSDEKDSVL
HLA-C07:01	FRSVSAEEPLC
HLA-C06:02	CRPQCCQPV
HLA-B08:01	SLKTPPECL
HLA-B57:01	HALDDGLF
HLA-C07:01	FSSIMPPAM
HLA-B08:01	LVKPSGTLSL
HLA-B08:01	TPGTTHTATVL
HLA-C07:01	SVNSPPPQP
HLA-C06:02	IAKVFQIDV
HLA-C07:01	KATSNEKDS
HLA-C07:01	VSCGGSKGG
HLA-B08:01	IPPEKPTI
HLA-C07:01	ISTATNSES
HLA-B57:01	TSSGANTATN
HLA-C07:01	LALDVDRIK
HLA-C06:02	DGYDPVQEN
HLA-DRB115:0	FHFLLILGSYVRILS
HLA-DRB107:0	LDNRFFTLTVTSLI
HLA-DRB107:0	AAFTTAKAAALST
HLA-DRB115:0	RHSMIRRVKRFLS
HLA-DQA101:0	RVLKTGQSMTLLG
HLA-DRB115:0	KIYIVGGYNWRLT
HLA-DQA102:0	SACPTSVSSAAAC
HLA-DRB107:0	KATKQLGFIMAAI
HLA-DRB107:0	FQEMIQRLYGCLL
HLA-DQA101:0	TPGTTHTATVLTT
HLA-DQA102:0	FEPLVASGVPASF
HLA-DRB107:0	QKEDVDFLARFSK
HLA-DQA101:0	MVRNAEKAMTA
HLA-DRB115:0	SRFKMPKFKMLS
HLA-DRB107:0	QFMAVKLSDSRIA
HLA-DRB107:0	DRMLTHLTGSISY
HLA-DQA101:0	MCFSSIMPPAMA
HLA-DRB107:0	FVEAFLKQCMSLL
HLA-DQA102:0	GTNAVIAYTVQSS
HLA-DQA102:0	LEERTPAMLSSAE
HLA-DRB107:0	PGLVKPSGTLSLTG
HLA-DRB107:0	HVYTFRSVSAEEP
HLA-DRB107:0	SSFSTAKTSTSLHS
HLA-DRB107:0	SLDGCYSTPSMFF
HLA-DQA101:0	ESSTVSSGISTATN
HLA-DQA102:0	DELVGHSVAIGPF

HLA-DQA102:0	NSTDEEVASSLTL
HLA-DQA101:0	RSLNEHLQALLTP
HLA-DQA102:0	NEKDSVSNIATEI
HLA-DRB107:0	TSWPDLIAKVFQI
HLA-DQA102:0	LEEQYLGLALDVD
HLA-DQA101:0	SVKEMLKSVMQE
HLA-DQA101:0	MDEGTYIHALDD
HLA-DQA101:0	NRLMQDEIAWLI
HLA-DQA102:0	ESSTTSSGANTAT
HLA-DRB107:0	KKYLKDFEIVKRK
HLA-DQA102:0	ENRLMQDEIAWI
HLA-DRB107:0	GFKLKEVSSVNSP
HLA-DRB115:0	FDLRSKMGKWC
HLA-DRB115:0	ECELLRHPPAPH
HLA-DQA101:0	SSDEKDSVLNIAR
HLA-DQA102:0	SMEETIISTEKPTI
HLA-DRB107:0	DNSLSLKTPPECL
HLA-DQA102:0	SCGGSKGGCVSC
HLA-DQA102:0	CRPQCCQPVCCC
HLA-C07:01	YRHPRNHIGKM
HLA-C07:01	FRLPNQCVL
HLA-B08:01	LLREHISVL
HLA-B57:01	LAGALESVW
HLA-B57:01	VAIGPREQW
HLA-B08:01	YLDNRFFTL
HLA-B08:01	SMRLYVGSL
HLA-C06:02	FRLNHDELA
HLA-C07:01	NRYTESISV
HLA-B08:01	SLNEHLQAL
HLA-C07:01	FVYCHIHRI
HLA-C07:01	SRSRRGYL
HLA-C07:01	WRLTNVTGI
HLA-C07:01	RRPHRPLRV
HLA-C07:01	LHIDYIRYY
HLA-B08:01	WARLLRPIL
HLA-C06:02	RRFVRLAATTF
HLA-C07:01	FRDECELLR
HLA-B57:01	YSTPSMFF
HLA-B08:01	SLHSRTSST
HLA-C07:01	TLQTRPSLI
HLA-A01:01	LSDSRTALK
HLA-B08:01	FAVMRFSL
HLA-B08:01	EMIQRLYGCL
HLA-C07:01	LRSQDPHLPL
HLA-A01:01	ATHMCFCFGSY
HLA-C07:01	AREVAERKR
HLA-C07:01	QYDGNSSFL

HLA-B57:01	KSVMQELEDY
HLA-C06:02	LKSGHGKYL
HLA-B57:01	LALEESGTF
HLA-C06:02	FSNPDGNVSLI
HLA-C07:01	QSSDRDLFV
HLA-B08:01	MPTQLDMAM
HLA-C07:01	PRQSPKRWR
HLA-C07:01	SSDEKDSVL
HLA-C07:01	FRSVSAEEPLC
HLA-B08:01	VPKSKAAKEL
HLA-C06:02	CRPQCCQPV
HLA-C07:01	SASVPKPDK
HLA-B57:01	HQHVVYGPW
HLA-C07:01	IHTGAKHYK
HLA-B57:01	RSQLGEKFQI
HLA-B57:01	HALDDGLF
HLA-B08:01	QLRGVKLL
HLA-B08:01	LVKPSGTLSL
HLA-C07:01	LSHYWTPCN
HLA-C06:02	TYKASRAGV
HLA-B08:01	ELHTKKGKMDL
HLA-C07:01	SVNSPPPQP
HLA-C06:02	IAKVFQIDV
HLA-B08:01	MCSHHANCF
HLA-B57:01	RTLAHSRPQTQ
HLA-C07:01	VSCGGSKGG
HLA-B57:01	WEVPFVTW
HLA-C07:01	SAQRRSQGR
HLA-C07:01	WSLGEDDHH
HLA-B08:01	KLKKDLQEVL
HLA-C06:02	DGYDPVQEN
HLA-C07:01	IGEDPDPLN
HLA-A01:01	LLDGDDNEDR
HLA-C06:02	VAQDEEENA
HLA-DRB107:0	SSRRFVRLAATTF
HLA-DRB107:0	PAYFVYCHIHRIPP
HLA-DRB107:0	LDNRFFTLTVTSL
HLA-DRB107:0	VKLLFNETINVSV
HLA-DRB107:0	EIGLSATLQTRPSL
HLA-DRB107:0	RPHRPLRVCPARL
HLA-DRB107:0	SKHKIIHTGAKHY
HLA-DQA101:0	LNHDELAAVQAC
HLA-DRB107:0	EGIVPQYDGSNS
HLA-DRB115:0	KIYIVGGYNWRLT
HLA-DRB107:0	RNRYTESISVAKIS
HLA-DQA102:0	SACPTSVSSAAAC
HLA-DRB115:0	SILFAVMRFSLKT

HLA-DRB115:0	WGVKILHIDYIRY
HLA-DRB115:0	AGSMRLYVGSLH
HLA-DRB107:0	SVWILKSAEGRVL
HLA-DRB107:0	FQEMIQRLYGCLL
HLA-DRB107:0	AQVIYSLLRSQDP
HLA-DRB115:0	DMAMDTMIRIFH
HLA-DRB107:0	ISVLSRTFANQRR
HLA-DQA102:C	RPILQFGLVAVSIY
HLA-DQA102:C	TYKASRAGVHAQ
HLA-DQA102:C	GTNAVIAYTVQSS
HLA-DQA101:C	NPDGNVSLIQVG
HLA-DRB107:0	LWVVHVSGPINP
HLA-DQA102:C	KGKLAAVAQDEE
HLA-DRB107:0	SRIALKSGHGKYL
HLA-DRB115:0	QEVKILRSQLGEK
HLA-DQA102:C	TIIGPPAMQVEVL
HLA-DRB107:0	PGLVKPSGTLSLT
HLA-DRB107:0	HVYTFRSVSAEEP
HLA-DRB107:0	PFFDLKPVSVDLA
HLA-DRB107:0	SSFSTAKTSTSLHS
HLA-DRB107:0	SLDGCYSTPSMFF
HLA-DRB107:0	TLYKAKMWSLGE
HLA-DRB107:0	VFTVSCGPSVPQ
HLA-DQA102:C	DELVGHSVAIGPF
HLA-DQA102:C	NSTDEEVASSLTL
HLA-DQA101:C	RSLNEHLQALLTP
HLA-DRB107:0	KGKMDLLASNGC
HLA-DRB115:0	ATHMCFCFGSYV
HLA-DRB107:0	TSWPDLIAKVFQI
HLA-DRB107:0	PHKDRTLAHSRP
HLA-DQA101:C	SVKEMLKSVMQE
HLA-DQA101:C	MDEGTYIHALDD
HLA-DRB115:0	RSRRGYLAYRERC
HLA-DRB115:0	DKDFLLSHWNDF
HLA-DRB107:0	SDLYRHPRNHIGK
HLA-DRB115:0	WEVPFVTWFFKC
HLA-DRB107:0	CTMDSHMCSHH
HLA-DRB107:0	GFKLKEVSSVNSP
HLA-DRB115:0	CLPNHQHVVYGP
HLA-DRB107:0	LQEVLELTKDLLST
HLA-DQA102:C	LGINSDELVGHSV
HLA-DQA102:C	PLNQSSSPSQMF
HLA-DRB115:0	ECELLRHPPAPHC
HLA-DQA101:C	SSDEKDSVLNIAR
HLA-DQA102:C	KEQQRTSAQRRS
HLA-DQA102:C	SCGGSKGGCVSC
HLA-DQA102:C	GGADDSAEEGDL

HLA-DQA102:0	CRPQCCQPVCCC
HLA-DQA102:0	RNEGSPSPRQSPI
HLA-C07:01	FRLPNQCVL
HLA-B08:01	VNRARIKSL
HLA-B08:01	REKRRRQEL
HLA-C07:01	WRLTNVTGI
HLA-C07:01	RRYLREPLWL
HLA-C07:01	SFSECSSLI
HLA-C07:01	FRDECELLR
HLA-C07:01	KRENEERLF
HLA-B08:01	SLHSRTSST
HLA-C07:01	HRHHVDVHL
HLA-B08:01	EMIQRLYGCL
HLA-C06:02	TSMPVSTTL
HLA-B08:01	NPQQRLQMSA
HLA-B08:01	LALLKKLSG
HLA-B57:01	KAAALSTKI
HLA-B57:01	YSWSPLQQHFM
HLA-C06:02	KRPGSRPQI
HLA-B08:01	LPLTSLSSV
HLA-C07:01	QSSDRDLFV
HLA-C06:02	QRPQMSAVYV
HLA-B57:01	KSTISPEKPTI
HLA-B57:01	HPQPQPPLW
HLA-B08:01	MPLTPQQAQEL
HLA-B08:01	CLCQFKKSI
HLA-B57:01	ISTEKLTI
HLA-C07:01	NRTTQQDNP
HLA-C07:01	LTPPQAQEL
HLA-C07:01	EQQEKQPEL
HLA-B08:01	IPLTPQQAQAL
HLA-C07:01	LSEGTTPLT
HLA-C06:02	IAKVFQIDV
HLA-B57:01	MGLLGLCEEVF
HLA-C07:01	RSCAEKETT
HLA-B08:01	NPKWNSNCE
HLA-B08:01	LPVTSLSSA
HLA-A01:01	VTDTSSAST
HLA-C07:01	SIATSTPSE
HLA-C06:02	DGYDPVQEN
HLA-C07:01	TTTTGTPTP
HLA-A01:01	PTEKPTIPI
HLA-C06:02	EAEGEAQEA
HLA-B57:01	PTISPEKPTI
HLA-DRB107:0	AAFTTAKAAALST
HLA-DRB107:0	RARIKSLCALAAE(
HLA-DRB115:0	KIYIVGGYNWRLT

HLA-DQA102:0	SACPTSVSSAAAC
HLA-DQA101:0	SMPVSTTLVASSE
HLA-DRB107:0	LLMSYAFLLALLKI
HLA-DRB107:0	FQEMIQRLYGCLL
HLA-DQA101:0	QQRLQMSAVYAI
HLA-DRB115:0	LVPIMYSWSPLQ
HLA-DRB107:0	KSFSECSSLIKHRR
HLA-DQA102:0	GTNAVIAYTVQSS
HLA-DRB107:0	QATPLPLTSLSSVS
HLA-DQA101:0	PQQAQALGILLTP
HLA-DRB107:0	TTPLTSTPVSHTLV
HLA-DQA102:0	TPAEGTSIATSTPS
HLA-DRB107:0	RYLREPLWLTHEV
HLA-DRB107:0	SSFSTAKTSTSLHS
HLA-DQA102:0	QTLGIPLTPQQAC
HLA-DQA102:0	NSTDEEVASSLTL
HLA-DQA102:0	QQAQALGMPLTI
HLA-DRB107:0	TSWPDLIAKVFQI
HLA-DQA102:0	PESEGVEAPEAEG
HLA-DRB107:0	EVSKSTRSHRHHV
HLA-DRB115:0	MACCLCQFKKSIE
HLA-DQA101:0	TIPIEETTISTEKLT
HLA-DQA102:0	GLCEEVFGTADLY
HLA-DQA102:0	STGDTTSLPVTDT
HLA-DRB107:0	TPTEKPTISTEKLT
HLA-DRB115:0	ECELLRHPPAPHC
HLA-DQA102:0	CEFFIRDLEQEVL
HLA-DQA102:0	RENEERLFGSQEI
HLA-DQA102:0	SASTGDTTPLPVT
HLA-DRB107:0	IGDYNRTVGKRP
HLA-DQA102:0	TQSPTPTAITTTTT
HLA-DQA101:0	EEMIKIRSCAEKE
HLA-DQA102:0	IPTEKSTISPEKPTI
HLA-DQA102:0	SWPTAASPPGGP
HLA-DQA102:0	PTISTEKLTIPTEKI
HLA-DQA102:0	LKYLEQQEGQLKI
HLA-DQA102:0	LQQEEEQLLGEEI
HLA-DQA102:0	TTQQDNPRTSCA
HLA-DQA102:0	DQMPRNPNPQC
HLA-A31:01	VVYPLDFVR
HLA-A31:01	KSLPKKINAR
HLA-C14:02	TYDEFRSFF
HLA-C14:02	IFHGSILFL
HLA-C07:01	SRKKRVIKV
HLA-C14:02	TFHGAGLKRYL
HLA-A31:01	RTPQLQAEPR
HLA-C14:02	RAHCEGQEM

HLA-C14:02	YTIGVEAKV
HLA-C14:02	KYKKTSLSTQV
HLA-A31:01	QIMQQMSDHR
HLA-DQA101:0	YDEFRSFFQFLNN
HLA-DRB115:0	SILFLYCVPNSKNS
HLA-DQA101:0	IAIGSSESAQKAL
HLA-DRB114:5	PLDIVITGKYKKTS
HLA-DRB114:5	MATFHGAGLKRY
HLA-DRB114:5	VYPLDFVRTRLGV
HLA-DQA101:0	GLAQVPQVAPVR
HLA-DRB114:5	SQKQVKSLPKKIN
HLA-DQA101:0	RDSYTIGVEAKVG
HLA-DQA101:0	KKRVIKVYSEDETS
HLA-DQA101:0	EKKLEDARAHCE
HLA-B35:01	FAFNVKTSF
HLA-B07:02	KPRLSFAHRAL
HLA-A11:01	RMPFYGYHEK
HLA-A11:01	VVVSPSEFK
HLA-C07:02	YRRCSDFHDF
HLA-C07:02	VYMLSVKGL
HLA-B07:02	HPGHTHSQA
HLA-B07:02	QPHPGFTGEL
HLA-B07:02	SPCSSLPPI
HLA-B07:02	QPPPQPQPPL
HLA-B07:02	PPQSPHQSPL
HLA-B07:02	RALGCSSSV
HLA-C07:02	LKESVSSIL
HLA-A11:01	GLPDCLDK
HLA-DRB111:0	DVYMLSVKGLAR
HLA-DRB111:0	QHVFKVSLVSRM
HLA-DRB111:0	KPRLSFAHRALIN
HLA-DRB111:0	IAFAFNVKTSFM
HLA-DQA105:0	TSSTASPPASSPC
HLA-DRB111:0	LVVARSVGTFAR
HLA-DQA105:0	SVSSILAGVNIVG
HLA-DRB111:0	FAKLIQTIWTSSP
HLA-DQA105:0	AAPVKTALPAGP
HLA-DRB111:0	SEEMWKTYRRCS
HLA-DQA105:0	HPGFTGELSKPAL
HLA-DQA105:0	PGHTHSQAGSRH
HLA-DQA105:0	HQSPLDSCSSPLL
HLA-DQA105:0	CIPGAWQCDGLP
HLA-C03:03	LCLGHFHNL
HLA-C03:03	VTYRETTLL
HLA-C03:03	THVSGSLPL
HLA-C03:03	YTCQTAAL
HLA-B15:01	VQSQFNIY

HLA-B15:01	LIHDHTYY
HLA-B15:01	AVVPLIKGVF
HLA-B07:02	VPKSISVLPV
HLA-C03:03	LILSHMQKL
HLA-C07:02	YRKRKLDIIL
HLA-B15:01	ASAEGPVADAF
HLA-B15:01	EQKENFSGM
HLA-A02:01	SLHYWDEDV
HLA-A02:01	MLYDCMSDS
HLA-A02:01	AMCEMEKKV
HLA-B07:02	HPTRRSCSPT
HLA-B15:01	GIKSGRMMF
HLA-B07:02	APPGASLLLLL
HLA-B07:02	CPDNGSCV
HLA-A02:01	VSFEDEDEIV
HLA-C03:03	YVSTGHQPH
HLA-C07:02	GRWKEEDSK
HLA-C03:03	AAAQEGEDE
HLA-DRB101:0	CQTAALQQVLST
HLA-DRB101:0	EEVFNTAVVPLIK
HLA-DRB101:0	LGHFHNLHVFQ
HLA-DRB101:0	FVIIRREKLILSHM
HLA-DRB101:0	VPKSISVLPVNPH
HLA-DRB101:0	FWVVADEALSKY
HLA-DRB101:0	QHLHTHVSGSLP
HLA-DQA101:	AAPPGASLLLLLQ
HLA-DQA101:	YQVSCMLCAES
HLA-DRB101:0	SYRELARVQSQF
HLA-DRB101:0	PSSFSTLIHDHTY
HLA-DQA101:	SPGSLHYWDED
HLA-DRB113:0	EDEDEIVRINPRD
HLA-DRB101:0	DLVTYRETTLLID
HLA-DRB101:0	ARRAEGIKSGRM
HLA-DQA101:	EEAKSVAMCEM
HLA-DQA101:	QQQGLSNASAE
HLA-DRB101:0	KENFSGMNGDL
HLA-DRB101:0	AQNYRKRKLDIIL
HLA-DRB101:0	QRSLDEIHPTRRS
HLA-DRB101:0	PATQRQDPAAA
HLA-DRB113:0	DNGSCVPDGPGL
HLA-DQA101:	EDRQGRWKEED
HLA-A02:01	ILHENLLLV
HLA-C03:03	SAISTLSATPV
HLA-B15:01	GQPDRQGQSY
HLA-C03:03	KAFSQSSTL
HLA-C03:03	VTYRETTLL
HLA-C03:03	SVVKPSFLPL

HLA-C03:03	FQHSNCAL
HLA-C03:03	KAFRQSANL
HLA-A02:01	GLVENLLVICI
HLA-C03:03	AANVRSTL
HLA-C03:03	TAAPPTPPA
HLA-B15:01	KIIHTGEKPY
HLA-B07:02	TPSERQLTPL
HLA-B07:02	SPSKSGSEA
HLA-B15:01	KLVCTSPEY
HLA-C03:03	KSFTCSSSL
HLA-B07:02	SPEKLTTPT
HLA-C03:03	KAFYKRYRL
HLA-B15:01	LQISTSTDRPV
HLA-B15:01	KLHTGEKPY
HLA-B07:02	DPSCPRLSREL
HLA-B07:02	SPEKLTIPT
HLA-A02:01	SLHYWDEDV
HLA-B15:01	KIHTGEKPY
HLA-A02:01	AMCEMEKKV
HLA-C07:02	FRETGNNF
HLA-B15:01	VTLPVFKHY
HLA-B15:01	RIHTGEKLY
HLA-B07:02	KPHGCSLCGKA
HLA-B07:02	CPDNGSCV
HLA-B15:01	YKCEECSKAY
HLA-C03:03	YVSTGHQPH
HLA-B07:02	RPYKCEECGKA
HLA-B15:01	YKCEECDKAY
HLA-B15:01	TQKDSTSKSG
HLA-C07:02	IHTGEKPYK
HLA-B15:01	YKCEECCKAF
HLA-C03:03	SEIEDENQI
HLA-C03:03	KPYKCEECG
HLA-B07:02	KCEECGKAFNQ
HLA-DRB101:0	LPLPFDIMKGLTL
HLA-DRB101:0	FNQFSNLTTHKII
HLA-DRB101:0	CALSTPYIHSLAPI
HLA-DRB101:0	SSAISTLSATPVD
HLA-DRB101:0	GKAFRQSANLYV
HLA-DRB101:0	GKAFSQSSTLTTH
HLA-DRB101:0	AEDLRILHENLLL
HLA-DRB101:0	KLKLVCTVPLSGS
HLA-DRB101:0	MFVVGLVENLLV
HLA-DRB101:0	FYKRYRLTEHERA
HLA-DRB101:0	FWVVADEALSKY
HLA-DQA101:	GNNFSDVAIQIR
HLA-DRB101:0	GKAFNQSSTLTR

HLA-DRB101:0	CGKSFTCSSSLIKH
HLA-DRB101:0	ERQLTPLPPSADD
HLA-DRB101:0	KKDNVAGVTLPV
HLA-DRB101:0	SKAYRWFSDLAKI
HLA-DRB101:0	EECGKAFNQSSTL
HLA-DQA101:0	SPGSLHYWDEDV
HLA-DRB101:0	EECCKAFNKFSNL
HLA-DRB101:0	ECGKAFNRSSNLI
HLA-DRB101:0	DLVTYRETTLLIDP
HLA-DQA101:0	EEAKSVAMCEME
HLA-DQA101:0	SCPRLSRELLEVVA
HLA-DRB113:0	ELQISTSTDRPVTP
HLA-DQA101:0	EKGTADAANVRS
HLA-DRB101:0	KWSSTLSYHKKIH
HLA-DRB113:0	ILTKHKIIHTGEKPY
HLA-DRB113:0	TIISTEKPTISPEKL
HLA-DRB113:0	ENQILENSSQKEN
HLA-DRB101:0	PTPPATTPAPPSS
HLA-DQA101:0	KDSTSKSGSEAQT
HLA-DQA101:0	KCEECGKAFNCSS
HLA-DRB113:0	DNGSCVPDGPGL
HLA-DQA101:0	EKPYKCEECGKAF
HLA-DRB113:0	HKRIHTGKKPHGC
HLA-DQA101:0	PDRQGQSYHYGC
HLA-DQA101:0	TEKPTISPEKLTTP
HLA-C03:03	FGVYVGTAI
HLA-B07:02	RPKDAATPL
HLA-C03:03	YRIDANQDL
HLA-A02:01	YLAYQNNQV
HLA-C03:03	KAFPFYSSYL
HLA-B15:01	YLMGTELF
HLA-C03:03	KAMSVTKPI
HLA-B15:01	ALREGTRVQSM
HLA-B15:01	AQALEELFPHY
HLA-B07:02	VVRSPHGVL
HLA-B15:01	VQYGHTQM
HLA-B07:02	LPGHLKLTTL
HLA-B07:02	TPSERQLTPL
HLA-A02:01	KLSDFLTLEGI
HLA-C03:03	FALSTGDAS
HLA-A02:01	NIFVDVILV
HLA-A02:01	GLIGVQQPPQI
HLA-B07:02	SPPVPSWNEL
HLA-B07:02	EPRRETTWSI
HLA-C03:03	VISHVGDVL
HLA-C07:02	SFKDNLKDF
HLA-A02:01	LLDPYGGHRNV

HLA-B07:02	EPPGPRGDL
HLA-A02:01	WLMLISMANDL
HLA-A02:01	CLLNNVDLA
HLA-A02:01	TLLEDKDVSVI
HLA-A02:01	SLHYWDEDV
HLA-A02:01	AMCEMEKKV
HLA-C03:03	HSTGDNLLL
HLA-C07:02	RRKSGHPVH
HLA-C03:03	TSSTHHAEV
HLA-C07:02	LREEREKRR
HLA-A02:01	YMQRKCQECRL
HLA-B07:02	CPDNGSCV
HLA-B07:02	KPNNSNAH
HLA-C03:03	YVSTGHQPH
HLA-C03:03	TATTSESST
HLA-DRB101:0	LSDFLTLEGINSKI
HLA-DRB101:0	DLLAIGLTNMLGS
HLA-DRB101:0	RCVWLMLISMAN
HLA-DRB101:0	VVRYLAYQNNQV
HLA-DRB101:0	MPGLKGLKGLPG
HLA-DRB101:0	IAQLKDLLPGHLK
HLA-DRB101:0	IRCFGVYVGTAILV
HLA-DRB101:0	PENLYLMGTELFE
HLA-DRB101:0	HRGVTVVRSPHG
HLA-DRB101:0	ASQPVRLFRGRTS
HLA-DRB101:0	QGFQGLIGVQQP
HLA-DRB101:0	LFPHYTSLRPGPP
HLA-DRB113:0	SVTKPITVTKSVPV
HLA-DRB101:0	FWVVADEALSKY
HLA-DRB113:0	FVDVILVDPNHW
HLA-DRB101:0	SLYQALREGTRVC
HLA-DQA101:0	RRETTWSIGLQVT
HLA-DRB101:0	ERQLTPLPPSADD
HLA-DQA101:0	SPGSLHYWDEDV
HLA-DQA101:0	YGGHRNVAYIVV
HLA-DRB101:0	EEPYTPSPPVPSW
HLA-DRB101:0	AFPFYSSYLRHERT
HLA-DRB113:0	RTKCLLNNVDLAP
HLA-DQA101:0	KNGGNCVMDMY
HLA-DQA101:0	RKGDGEILVELVC
HLA-DRB113:0	SRTLLEDKDVSVIY
HLA-DRB101:0	DNLKDFLLVIPFD
HLA-DQA101:0	GGVSTGVISHVGI
HLA-DQA101:0	EEAKSVAMCEME
HLA-DQA101:0	SSTTSSGASTATTS
HLA-DRB101:0	TSTSLHSHTSSTHI
HLA-DQA101:0	FENTENATITDQH

HLA-DRB101:0	LEHLQQQRRKSG
HLA-DQA101:0	HRPKDAATPLRST
HLA-DRB101:0	SEGEQQLKPNNS
HLA-DRB113:0	DNGSCVPDGPGL
HLA-DRB113:0	EEQLLREEREKRR
HLA-B07:02	RPRVAKTWM
HLA-C03:03	YASNLSKDPVL
HLA-B07:02	RPKDAATPL
HLA-A02:01	YLAYQNNQV
HLA-C03:03	AAAVATPWM
HLA-B15:01	VQMCKMKVF
HLA-C03:03	LDYPRIVKL
HLA-B07:02	VVRSPHGVL
HLA-B07:02	LPGHLKLTTL
HLA-A02:01	KLSDFLTLEGI
HLA-C03:03	FALSTGDAS
HLA-B15:01	KQQLSHIY
HLA-A02:01	GLIGVQQPPQI
HLA-B07:02	SPPVPSWNEL
HLA-B07:02	EPRRETTWSI
HLA-C03:03	VISHVGDVL
HLA-C07:02	SFKDNLKDF
HLA-A02:01	LLDPYGGHRNV
HLA-B07:02	EPPGPRGDL
HLA-B07:02	RPEPPDTGV
HLA-B07:02	SPKGSPAT
HLA-B07:02	APTTTKKPA
HLA-B15:01	YQGAQGPPG
HLA-A02:01	TLLEDKDVSVI
HLA-A02:01	SLHYWDEDV
HLA-B07:02	SPEKLTIST
HLA-B15:01	RVNKEQDHF
HLA-A02:01	AMCEMEKKV
HLA-C03:03	HSTGDNLLL
HLA-C07:02	EREKRRRQE
HLA-C07:02	IYLNTGDL
HLA-A02:01	YMQRKCQECRL
HLA-B07:02	CPDNGSCV
HLA-B07:02	KPNNSNAH
HLA-C03:03	YVSTGHQPH
HLA-C07:02	GRDFNSTKN
HLA-DRB101:0	LSDFLTLEGINSKI
HLA-DRB101:0	NNAYAVAVANYA
HLA-DRB101:0	VVRYLAYQNNQV
HLA-DRB101:0	MPGLKGLKGLPG
HLA-DRB101:0	IAQLKDLLPGHLK
HLA-DRB101:0	VAKTWMFLLLG

HLA-DRB101:0	HRGVTVVRSPHG
HLA-DRB101:0	ASQPVRLFRGRTS
HLA-DRB101:0	SDTYVTLRVNKEC
HLA-DRB101:0	KGDIYLNTGDLIV
HLA-DRB101:0	QGFQGLIGVQQP
HLA-DRB101:0	LLRVLVQMCKMK
HLA-DRB101:0	VATPWMRAGAL
HLA-DRB101:0	FWVVADEALSKY
HLA-DQA101:0	RRETTWSIGLQVT
HLA-DRB101:0	YPRIVKLYDYFSLD
HLA-DQA101:0	SPGSLHYWDEDV
HLA-DRB101:0	FVEYNKQQLSHIY
HLA-DQA101:0	YGGHRNVAYIVV
HLA-DRB101:0	EKGYQGAQGPPC
HLA-DRB101:0	EEPYTPSPPVPSW
HLA-DRB101:0	GRDFNSTKNVKN
HLA-DRB101:0	KTRMYSLRPEPPD
HLA-DQA101:0	KNGGNCVMDMY
HLA-DRB113:0	SRTLLEDKDVSVIY
HLA-DRB101:0	DNLKDFLLVIPFD
HLA-DQA101:0	GGVSTGVISHVGI
HLA-DQA101:0	EEAKSVAMCEME
HLA-DQA101:0	FENTENATITDQH
HLA-DQA101:0	HRPKDAATPLRST
HLA-DRB113:0	PEKLTISTEKPTIPT
HLA-DRB101:0	APTTTKKPAPTTP
HLA-DRB101:0	SEGEQQLKPNNS
HLA-DRB113:0	DNGSCVPDGPGL
HLA-DQA101:0	LQQEEEQLLGEEF
HLA-DRB101:0	APTTPAATPPSPK
HLA-A02:01	ALFSGSLEPV
HLA-A02:01	RLTRFLPKV
HLA-A02:01	SLLNMICQQV
HLA-A02:01	SLHSFSVEV
HLA-A02:01	YLHVCPQPL
HLA-A02:01	GILCLPLFHLV
HLA-C06:02	SRTNCVNIF
HLA-A02:01	ALEWQLPPL
HLA-A02:01	SQLEDELEV
HLA-C06:02	YRRMSGVRPL
HLA-A02:01	SVLSHSNLV
HLA-A02:01	SLYASGGFAM
HLA-A02:01	LLVNKMLLEA
HLA-A02:01	RLSHSPEHV
HLA-A02:01	FLGSANFGEAV
HLA-C06:02	SRPADRLL
HLA-C06:02	TSRGYTPYV

HLA-A02:01	YLRHCQEYKL
HLA-A02:01	HLAPKCRTPEL
HLA-A02:01	KMISTQQLMM
HLA-A02:01	GLREAQAFLRV
HLA-A02:01	GLTSPDPYI
HLA-A02:01	TSFHDILWHFV
HLA-C06:02	FRIPLKNV
HLA-A02:01	RLLEWQCGG
HLA-A02:01	HLQPSLEEGFI
HLA-C06:02	YRTYSKKHFWI
HLA-A02:01	TLITITNNM
HLA-A02:01	GMESMSQDTEV
HLA-C06:02	QRRETTHSA
HLA-C06:02	MTMTRPTTC
HLA-C06:02	ERKICGLTL
HLA-C06:02	SRYRNNIKN
HLA-C06:02	YSDSDLSSS
HLA-C06:02	NSSEQGVSL
HLA-C06:02	PRRERRQAN
HLA-A02:01	CMNDKDVSGKM
HLA-C06:02	HSRDFQILY
HLA-C06:02	VRQCILNRI
HLA-C06:02	QRTRVAPFL
HLA-C06:02	HSNSHNEFW
HLA-A02:01	IQILPKYSA
HLA-A02:01	GLGQGLKDI
HLA-A02:01	CQLELEEHYYN
HLA-C06:02	FTDKWEDHP
HLA-C06:02	TSEEPDECE
HLA-C06:02	CSSNPKPSN
HLA-C06:02	NCYGNSCGY
HLA-DRB113:0	ALSLIHSRTNCVN
HLA-DRB113:0	SGSVLSHSNLVIKI
HLA-DRB113:0	ILSGSVLSHSNLVI
HLA-DRB113:0	LSGSVLSHSNLVIK
HLA-DRB114:5	LVLSVKGLPRLTRI
HLA-DRB114:5	LKDIKRTCNRFRLI
HLA-DRB113:0	TLITITNNMGSIAI
HLA-DRB114:5	VRQCILNRIKANV
HLA-DRB114:5	DSGLREAQAFLRV
HLA-DQA101:0	QWFGYHIAWEVI
HLA-DQA101:0	TSFHDILWHFVAS
HLA-DQA101:0	SLYASGGFAMIQI
HLA-DRB113:0	SRDFQILYRNEEV
HLA-DRB113:0	RDFQILYRNEEVP
HLA-DRB113:0	DFQILYRNEEVPII
HLA-DRB114:5	MAKYRRMSGVR

HLA-DQA101:0	NKALEWQLPPLQ
HLA-DRB114:5	WEHLKKVASRYR
HLA-DRB114:5	ECEKFRKAFSHLT
HLA-DRB114:5	LSQEQIQILPKYSA
HLA-DRB114:5	RKKMISTQQLMN
HLA-DRB113:0	HCQEYKLDPVNA
HLA-DQA101:0	YSKKHFWIFREEN
HLA-DQA101:0	EHYYNEMFGVW
HLA-DQA101:0	LPLFHLVLSDLPCE
HLA-DQA101:0	ESLHSFSVEVPVS
HLA-DQA101:0	RKICGLTLVSAPH
HLA-DRB114:5	PDPYIIVGSHHHT
HLA-DQA101:0	EFSTALFSGSLEPV
HLA-DQA101:0	QNSLLNMICQQV
HLA-DQA101:0	GSANFGEAVHFV
HLA-DQA101:0	SHNEFWCALVEK
HLA-DRB114:5	IYTWARGRVVSSI
HLA-DRB114:5	TERYLHVCPQPLA
HLA-DRB114:5	GKAFSVSGSLQRF
HLA-DQA101:0	EQGVSLDIDLWD
HLA-DRB114:5	KTDYMTMTRPTT
HLA-DQA101:0	SSHWKCARLLEW
HLA-DRB113:0	MRLRLSHSPEHVI
HLA-DQA101:0	RGYTPYVSHVGL
HLA-DQA101:0	PFLEKQGFKQQA
HLA-DRB113:0	LEDELEVGNVPLK
HLA-DQA101:0	PLNAAPYGMESN
HLA-DQA101:0	QPSLEEGFIDTHC
HLA-DRB114:5	TTPGFRIPLKNVH
HLA-DRB114:5	PLNLAHQQSRPA
HLA-DRB114:5	SDPFTHLAPKCRT
HLA-DRB113:0	VDDGFCSSNPKPS
HLA-DRB114:5	WIYMNRTFTDKV
HLA-DQA101:0	SSNSTDLPLNIEC
HLA-DRB114:5	PRRERRQANLLK
HLA-DQA101:0	MCCNCYGNSCG
HLA-B35:01	LPTRLTQTF
HLA-B35:01	YLIGCGNAY
HLA-B35:01	LATDLLFLM
HLA-B35:01	FLAFNERLY
HLA-C08:02	FKDAWLLHL
HLA-A24:02	TYAIYVRF
HLA-B35:01	MALSASNSCF
HLA-C08:02	NFDLKNNSL
HLA-B35:01	SPLVVQYVL
HLA-A26:01	EVLSSMSWI
HLA-A26:01	ETIGEILNKI

HLA-A26:01	ETAGGVLTAL
HLA-B35:01	RPLCLSKSIRF
HLA-B35:01	LPAETMRM
HLA-B35:01	FISTQKQVTF
HLA-B35:01	EAIRVPPTL
HLA-C08:02	ITSSNAAKL
HLA-B14:02	LLGLRDLVL
HLA-B35:01	WGTTTSPRVNY
HLA-C08:02	LGQPAQKCL
HLA-B35:01	FARVEPAL
HLA-B14:02	NQLERVPPL
HLA-C08:02	VNIKAGKAL
HLA-B35:01	RPLLAATTTKL
HLA-C08:02	ITQLEKKLL
HLA-C08:02	RVTNVPCAS
HLA-B35:01	EAPPALETL
HLA-C08:02	QGGGGQAPT
HLA-B35:01	VPLPPARPR
HLA-DRB101:0	PPLIRGLRGLTRLF
HLA-DRB101:0	TDLLFLMPSLTSG
HLA-DRB101:0	NRFVAITSSNAAK
HLA-DRB101:0	AVNIKAGKALLRL
HLA-DRB101:0	DDEYLALPTRLTQ
HLA-DRB101:0	SRPLLAATTTKLS(
HLA-DRB101:0	ARCFARVEPALKS
HLA-DRB101:0	LRDLVLLKVKLGD
HLA-DRB101:0	TEVLEKLRPLCLSK
HLA-DRB101:0	KQVTFLLKIKRTRK
HLA-DRB101:0	VVQYVLRPTSTV(
HLA-DRB101:0	ISKAIHLPAETMRI
HLA-DRB101:0	NERLYGPLQAYLF
HLA-DRB101:0	YDIFYTAAKPGTY/
HLA-DRB101:0	KEHITQLEKKLLLN
HLA-DRB101:0	KCLLQVVMRVSV
HLA-DRB101:0	SLGLETAGGVLTA
HLA-DRB101:0	AWLLHLHSGPW/
HLA-DRB101:0	GEILNKIIPTLEEY(
HLA-DRB101:0	WEPVFQNGKMA
HLA-DRB101:0	VGHYTQVVWYSS
HLA-DRB101:0	ETLHVVQRGEHSI
HLA-DRB101:0	LKQEVLSSMSWIS
HLA-DRB101:0	PLRGTANLSEAIR'
HLA-DRB101:0	EERNFDLKNNSLE
HLA-DRB101:0	RVNYPKTLQCAN
HLA-DRB101:0	EVKEAYMGNVLC
HLA-DQA103:(	NVPCASPGSVAG
HLA-DRB101:0	ASDPVPLPPARPF

HLA-C12:03	QAYPLLSVI
HLA-C12:03	FIRDVLQHV
HLA-B39:01	SPDPMTMAL
HLA-B40:02	KEMNSELSL
HLA-C12:03	IQESPGKVV
HLA-C12:03	NLLPSSRTL
HLA-C12:03	VARKMLLASTF
HLA-B39:01	YRLTTDPYRM
HLA-B40:02	AEQQRLAAG
HLA-B40:02	SEGKHKHSH
HLA-DRB113:0	FHVFIRDVLQHVD
HLA-DQA101:0	PDPMTMALSVLV
HLA-DQA101:0	GSPSLQAYPLLSV
HLA-DQA101:0	VARKMLLASTFSG
HLA-DRB113:0	SPTLNLLPSSRTLG
HLA-DRB113:0	YQRIRIQESPGKV
HLA-DRB116:0	TDPYRMSPRPYR
HLA-DQA101:0	CCGRAEQQRLAA
HLA-DQA101:0	AEKEMNSELSLSH
HLA-DRB116:0	KHKHSHSKGSSRE
HLA-A02:02	FLGNSAPVSL
HLA-A02:02	LLIEEPAFNV
HLA-A02:02	RLFRENSSLV
HLA-A02:02	FIFAVVDMQL
HLA-A02:02	QLAEVELRL
HLA-A02:02	LLLPLPQSFL
HLA-A02:02	IMWDYKDPL
HLA-A02:02	SLIPLMVNGI
HLA-A02:02	WLCGPDEEGV
HLA-A02:02	RMVQLESIDQV
HLA-A02:02	SLEMPDDFGV
HLA-A02:02	TLPRDIFQPL
HLA-A02:02	FQLFFADGIL
HLA-B44:03	DECDKVFNW
HLA-C03:04	KAFSRSSTL
HLA-A02:02	GTSYDSYWL
HLA-A02:02	QLKAAVGEL
HLA-B44:03	TELQENGCW
HLA-A02:02	KMPPLQAFT
HLA-A02:02	KLIDRVYKG
HLA-A02:02	SIPSPIAPL
HLA-C03:04	FVNGQPRPF
HLA-A02:02	NLIQQLIRA
HLA-A02:02	FYALEEQHV
HLA-C03:04	KAFNQSSNL
HLA-A02:02	TLERNPMSV
HLA-A02:02	KTFSKVSTL

HLA-A29:02	IIHTGEKPY
HLA-A29:02	KVIHTGEKPY
HLA-A02:02	TQISPKSWGV
HLA-A02:02	PLTPHPSGI
HLA-A02:02	VMFKSDRQAA
HLA-A02:02	RLDLNHKEV
HLA-C03:04	FGSASHLQI
HLA-A02:02	AMYYETFVW
HLA-A29:02	IIHTGKKPY
HLA-A02:02	ILTEHKIIHT
HLA-B44:03	HEKIHTDAF
HLA-A02:02	NLRQQNDTA
HLA-A02:02	SLTSHSQI
HLA-B44:03	CEECGKAF
HLA-A02:02	GLAVDNLF
HLA-C03:04	SAASSGNNSSA
HLA-B44:03	AEGQAEAEG
HLA-C03:04	YGQEGSGGAV
HLA-A02:02	TIATRFDRT
HLA-A02:02	TLTEHKRIHNG
HLA-B44:03	AEAKTQKGS
HLA-A02:02	CKPQCCQSV
HLA-C03:04	KTPPKRERKPL
HLA-B15:10	SQRSHRSPS
HLA-DRB111:0	AGFFIRRRMYPPL
HLA-DRB111:0	FSRFSYLTAHKIIH
HLA-DRB111:0	NIKNLIQQLIRAT
HLA-DRB111:0	GQLLIRLDLNHKE
HLA-DRB111:0	QTWLLNRYRMV
HLA-DRB111:0	EKAFVRKSHLVQ
HLA-DRB111:0	FESSQVKYLRREL
HLA-DQA105:0	YGQEGSGGAVST
HLA-DQA105:0	SRSTSTGSAASSG
HLA-DQA105:0	GISFLGNSAPVSL
HLA-DRB111:0	VDNLFHRPRATV
HLA-DRB111:0	FSTFSILTEHKIIHT
HLA-DRB111:0	FSKVSTLTTHKAI
HLA-DRB111:0	PFDIMKGLTLER
HLA-DQA105:0	RTHSNFILANAQ
HLA-DRB111:0	LQEIYQELTQLKA
HLA-DRB111:0	GKAFSRSSTLAKH
HLA-DRB111:0	WEKYKSSRKLIDR
HLA-DRB111:0	KHEIIHTGKKPYK
HLA-DRB111:0	DYKDPLVNLINKY
HLA-DQA105:0	TQISPKSWGVAT
HLA-DRB111:0	MELLLPLPQSFLL
HLA-DQA105:0	FCFGPGNVVSQS

HLA-DQA105:0	KQCGKAFGSASH
HLA-DRB111:0	LFRENSTLVRHQF
HLA-DRB111:0	VVDMQLFGKSYK
HLA-DRB111:0	ETVMFKSDRQAA
HLA-DRB111:0	KSSTLTYHKVIHT(
HLA-DRB111:0	MPDDFGVYSYLF
HLA-DQA105:0	LEEFQLFFADGILN
HLA-DRB111:0	PTDFCRKHKMPL
HLA-DRB111:0	EECGKAFNQSSTL
HLA-DRB111:0	DKVFNWWSQLT
HLA-DQA105:0	PEFAMYYETFVW
HLA-DQA105:0	QPYGSSFYALEEC
HLA-DQA105:0	VLLPGASVQLPM
HLA-DRB111:0	AFKWSSTLTEHKF
HLA-DQA105:0	SPNRSGAEAKTQ
HLA-DQA105:0	IQPPGIPPLGGSA
HLA-DQA105:0	MESIPSPIAPLPTF
HLA-DRB111:0	NQSSNLTNHKKIH
HLA-DRB111:0	NNNLQTLPRDIFC
HLA-DQA105:0	TSYDSYWLCDSLT
HLA-DQA105:0	ERKPLGGSSRGNS
HLA-DQA105:0	PRGEAEGQAEAE
HLA-DRB111:0	QLAEVELRLKDCL
HLA-DQA105:0	DPDADRLAATEL(
HLA-DQA105:0	DTASKIIDSQQDE
HLA-DQA105:0	SQRSHRSPSERSH
HLA-DRB111:0	PSCCVSSCCKPQC
HLA-A02:02	FMFFICLFTV
HLA-A02:02	FLIGNSTTL
HLA-A02:02	LLIEEPAFNV
HLA-A02:02	FFSVYLTAL
HLA-A02:02	LMMAKAAVV
HLA-A02:02	KMFSQEKWHV
HLA-A02:02	YVCTYQFSV
HLA-A29:02	CVFSFLDPLAY
HLA-A02:02	SLYEATIKNL
HLA-A02:02	LLYECTISPS
HLA-A02:02	VLNSAAGYKV
HLA-A29:02	AFRQDFWKY
HLA-A02:02	TLPRDIFQPL
HLA-A02:02	GLSQSWSERI
HLA-A29:02	ALTPPPFPSSY
HLA-A02:02	LIINETGASI
HLA-C03:04	FVNGQPRPF
HLA-A02:02	KLDECQEYG
HLA-A02:02	VVAACCFSRL
HLA-B44:03	SEDPFREKKF

HLA-A29:02	YQPPLQGQFY
HLA-A02:02	VVCAELGLV
HLA-A02:02	KVSPNEEDL
HLA-A02:02	YITFDAGHI
HLA-B44:03	EENTKILGKI
HLA-C03:04	RAAAKRTSTKM
HLA-A02:02	TQNPPGSPPNV
HLA-C03:04	FGEQDGGEL
HLA-B44:03	ADSPSQHW
HLA-B44:03	IETPEARNRGF
HLA-B44:03	GENCARWSS
HLA-B44:03	SEATTRTPGA
HLA-A29:02	HQRVHTEKKHY
HLA-A02:02	CKPQCCQSV
HLA-B15:10	QRHPDTLSF
HLA-A02:02	RIAERKSKEPQ
HLA-B44:03	EEDDDEEKDDL
HLA-A02:02	RCCRPQCCQSV
HLA-DRB111:0	ACCFSRLLRRHRL
HLA-DRB111:0	AGFFIRRRMYPPL
HLA-DRB111:0	KRMVVYQTAFRC
HLA-DQA105:0	SVLFRAAGLAGSD
HLA-DRB111:0	FESSQVKYLRRELI
HLA-DRB111:0	GISIMIKKPQKSKF
HLA-DQA105:0	ECHLMMAKAAV
HLA-DRB111:0	GYKVKCAYKKYLY
HLA-DRB110:0	SVYLTALLGNAAII
HLA-DRB111:0	QRRLKMFSQEKW
HLA-DRB111:0	YEATIKNLVKGIQI
HLA-DRB111:0	NTKILGKINQFFV/
HLA-DRB111:0	YNNIFNFRSNGLS
HLA-DQA105:0	ATTRTPGASPPAF
HLA-DRB110:0	VGGFFLIGNSTTL(
HLA-DRB111:0	FMFFICLFTVIVPC
HLA-DRB111:0	LNCYITFDAGHKL
HLA-DQA105:0	HWVMPPGPASW
HLA-DRB111:0	SKYASFSNHQRVI
HLA-DRB111:0	QGQFYAFSNFYYT
HLA-DRB111:0	SPPNVLWQTPLA
HLA-DRB111:0	PLMAQGAPWRT
HLA-DQA105:0	LLIINETGASIDDFI
HLA-DRB111:0	NNNLQTLPRDIFC
HLA-DRB111:0	EARNRGFLKGLFC
HLA-DRB111:0	LIEQRIAERKSKEP
HLA-DQA105:0	VAVCAELGLVLSG
HLA-DRB111:0	DTLSFRCSLADFQ
HLA-DRB110:0	KLLYECTISPSSPLY

HLA-DRB111:0	RWSSDKAHKVLK
HLA-DRB111:0	SEDPFREKKFFCA
HLA-DQA105:0	ATSTTASPLRDGF
HLA-DQA105:0	TREGALTPPPFPS
HLA-DRB111:0	CCISSCCRPSCCVS
HLA-DQA105:0	VTTLTADPPDGIK
HLA-DQA105:0	TTYCRPSCCVSSC
HLA-DQA105:0	KDDLEVKEENGV
HLA-A11:01	SSNLVLLYK
HLA-A02:01	FQNNQLISFEV
HLA-C07:01	TDFGHSEIL
HLA-B08:01	YPWRRCCL
HLA-A11:01	ASNMPGGFICR
HLA-A02:01	FSCKCLTGV
HLA-B44:27	FEVPGNQMTI
HLA-A02:01	ALTHFLPQGT
HLA-DRB107:0	NNQLISFEVIATLI
HLA-DRB107:0	RVTIRLSSNLVLLY
HLA-DRB103:0	HSEILGETSLMRT
HLA-DRB107:0	MGFRSKIKNALTH
HLA-DQA105:0	CLNGGTCAVASN
HLA-DRB107:0	KISFKEQVPIPLEE
HLA-DRB107:0	GNQMTILKTTSEE
HLA-DRB107:0	QFSCKCLTGVTGC
HLA-A68:01	TTVMISVIHER
HLA-A68:01	QIYDLVDRHFR
HLA-A68:01	MSSFLNKEDR
HLA-A68:01	FIASSALSR
HLA-A68:01	ETHRRKARR
HLA-A68:01	HIIINSMHK
HLA-B08:01	MLLAWSLAL
HLA-B08:01	AMMEHFVQL
HLA-A68:01	TSEDLENFK
HLA-A68:01	LVFEELTSK
HLA-A68:01	EAEQITLK
HLA-A68:01	EILGVQNLLQK
HLA-B41:01	LEQFVSIPM
HLA-B41:01	RERDKLHAV
HLA-A68:01	RASSPSTTR
HLA-B41:01	IEAITHGAL
HLA-B41:01	VEISAITHGAL
HLA-B41:01	AEKTEDVEI
HLA-B41:01	PEMNYKGQYEI
HLA-C07:02	LRPEPPDTG
HLA-C07:02	MKCQGQHWY
HLA-DRB107:0	GKAFIASSALSRH
HLA-DRB107:0	KLEQFVSIPMASI

HLA-DRB107:0	MEHFVQLTSRQ(
HLA-DQA105:(	LLAWSLALVASLG
HLA-DRB107:0	SEDLENFKALRVI\
HLA-DRB107:0	FKSYLSKYQMSSF
HLA-DQA105:(	SAITHGALQGLAY
HLA-DRB107:0	ALSCFVLTTVMIS'
HLA-DRB103:0	ANQIYDLVDRHFI
HLA-DRB103:0	ALRISKEVIRKRER
HLA-DRB107:0	HLDPFGHIIINSM
HLA-DQA102:(	RNKSQDSGIAEM
HLA-DQA105:(	PPPSLPTIGAETHI
HLA-DQA105:(	REPTRASSPSTTRI
HLA-DQA105:(	AKQTSEILGVQNL
HLA-DQA105:(	YKGQYEITAEDSC
HLA-DRB103:0	DLQHRLDEAEQIT
HLA-DQA105:(	PEPPDTGVSHLCF
HLA-DQA105:(	RRPAEKTEDVEIT
HLA-DRB107:0	CQGQHWYSSSDI
HLA-A02:01	SLFFYVWYV
HLA-C12:03	FSDENKNVV
HLA-A02:01	MLSHPLPGV
HLA-C12:03	IAMIIVVMV
HLA-C12:03	YKCEVVNKV
HLA-A02:01	FLIQQQYSQV
HLA-C12:03	FSLALALVSSL
HLA-A02:01	SLAPPPAEV
HLA-C12:03	YSLDVCHQIPL
HLA-C12:03	ASYLALTAV
HLA-C12:03	ITRDNINTHSL
HLA-C12:03	RSFRVGEAV
HLA-C12:03	TLGEPVETI
HLA-C12:03	SAHSINYSM
HLA-C12:03	EARLVVLVI
HLA-C06:02	MRSCRLRGSCL
HLA-C12:03	ISKPFMSL
HLA-A02:01	LLLHCSVSKA
HLA-A02:01	KLTVSQGQSV
HLA-A02:01	CLISGEPQPDV
HLA-C12:03	YPKSRKATA
HLA-C12:03	DNYPGGIDL
HLA-A02:01	KLTLSSNSL
HLA-C12:03	GASEHANTA
HLA-A02:01	HLEKQTALV
HLA-C12:03	LFFSRCHHY
HLA-C12:03	ITDFGHSEI
HLA-C06:02	TRWGKVKES
HLA-B18:01	REASGKCF

HLA-C12:03	NSPRTLPSL
HLA-DRB107:0	QKVLLHMLSHPLI
HLA-DRB107:0	GSRCLLLHCSVSK
HLA-DQA101:0	MSAFSLALALVSS
HLA-DRB107:0	GSLRKLTLSSNSLF
HLA-DRB107:0	EARLVVLVIANTN
HLA-DRB107:0	DISKPFMSLGISIN
HLA-DRB107:0	VVITRDNINTHSLI
HLA-DQA102:0	DVSIHGAVGASEH
HLA-DRB107:0	SAHSINYSMLGKI
HLA-DQA101:0	EKRYIASYLALTAV
HLA-DQA101:0	VIQAANVSALYKC
HLA-DRB107:0	PVKLTVSQGQSVI
HLA-DRB107:0	HFKVLREASGKCF
HLA-DRB113:0	VETIVISTDVANVS
HLA-DQA101:0	FLIQQQYSQVLAC
HLA-DRB113:0	KLDVSFQRSFRVG
HLA-DRB107:0	KTLCKLFFSRCHH
HLA-DRB107:0	YFGVMFFLNIAM
HLA-DRB113:0	HSEILGETSLMRT
HLA-DQA101:0	VPEKMSSLSILFSD
HLA-DQA102:0	FSRMGESLEPASL
HLA-DRB107:0	YGNSKKGLKAYSL
HLA-DQA102:0	GGIDLVTDEAEAS
HLA-DQA101:0	DVTWFQNGVLLK
HLA-DRB107:0	AIPPFCWVTRWG
HLA-DQA101:0	SCRLRGSCLARTC
HLA-DQA102:0	EAQQSSLAPPPAI
HLA-DQA101:0	LYHLEKQTALVGG
HLA-DQA102:0	YPKSRKATASAW
HLA-DQA102:0	PSLRGKARTGGPI
HLA-A02:01	AMIFFIYAI
HLA-B27:05	RRELQLPLFL
HLA-A02:01	KVYEENYFFI
HLA-A02:01	TIVENHTAYLV
HLA-A02:01	NLINLEIML
HLA-B51:08	FPKSCIDAMEI
HLA-B27:05	ARQLVFTAF
HLA-A02:01	DMCEYDVPYHI
HLA-A02:01	CQGFGSIFVV
HLA-A02:01	KQSENDLNKV
HLA-B27:05	VRALSGSATL
HLA-A02:01	SLATTSQNGA
HLA-A02:01	RQVGETVNLRI
HLA-C16:02	SSSQSTPPM
HLA-A02:01	CVIMNRSAV
HLA-A02:01	SANGCTDWSI

HLA-B51:08	FDTERADGTI
HLA-B51:08	MANEDYPC
HLA-DRB115:0	IFKCMLYLKYPKTI
HLA-DQA101:0	VRALSGSATLVSS
HLA-DRB107:0	AMIFFIYAIIGMQ
HLA-DRB107:0	QTYIRQVGETVNI
HLA-DQA102:0	ATTSQNGAPALV
HLA-DQA102:0	AGCQGFGSIFVVA
HLA-DRB107:0	CEYDVPYHIRLSID
HLA-DQA102:0	TAAATSTPVSSSQ
HLA-DQA101:0	WNVKNKASKVYE
HLA-DQA101:0	SSGAEFILAGLTQ
HLA-DRB115:0	IVAIASYTGYNQE
HLA-DQA102:0	LVFTAFTEEFLAA
HLA-DQA101:0	FEGNLINLEIMLKI
HLA-DQA101:0	AMEIDPSMLEVA
HLA-DRB107:0	ENDLNKVLESTRR
HLA-DQA102:0	TERADGTISSEIKS
HLA-DRB107:0	FTQSLKVVTKRGS
HLA-DQA102:0	DYPCPCDIGHRLE
HLA-B58:01	HSTGLRSLW
HLA-A11:01	TTKPAPTTPK
HLA-A11:01	SSLQSGVPSK
HLA-A11:01	TANMEQIMK
HLA-B35:01	TPADTSTPV
HLA-B58:01	RQGDVECQW
HLA-A11:01	YTLNNTHHSR
HLA-A11:01	YLTPIKHLRK
HLA-B35:01	EPPVVETEM
HLA-A11:01	CLLLWVKQRK
HLA-C03:02	FGSKVHSPV
HLA-B35:01	TPKEVPDTPM
HLA-B58:01	SSAEVVVPC
HLA-C03:02	RQQERELEL
HLA-B58:01	VSSVNSPPPQ
HLA-B35:01	RPRLAASL
HLA-B58:01	GSGCGGCGSGC
HLA-DRB116:0	YLTPIKHLRKSLKI
HLA-DRB113:0	LQLIEGKPPVVET
HLA-DRB116:0	LTCLLLWVKQRKD
HLA-DQA101:0	NELQNLSSAEVVV
HLA-DQA101:0	LIYAASSLQSGVPS
HLA-DQA101:0	RELQHRYADLLLE
HLA-DQA101:0	HSPVSEILFNDEM
HLA-DQA101:0	RRRPRLAASLQISI
HLA-DQA101:0	WTANMEQIMKA
HLA-DQA101:0	LRSLWMTITQCYI

HLA-DQA101:0	PVTSSAISTLSTTP
HLA-DRB113:0	TPKEVPDTPMNV
HLA-DRB116:0	RQALKVTVTSRAS
HLA-DQA101:0	WTECIKPVVQEVI
HLA-DRB113:0	FKLKEVSSVNSPP
HLA-DQA101:0	TKEPAPTTTKPAP
HLA-DQA101:0	CGGCGSGCGGCS
HLA-B55:01	RPIPLRFSV
HLA-A24:02	HYGQPVLSVW
HLA-A26:01	FVSEDNAAF
HLA-A24:02	PYRSAFYVL
HLA-B55:01	SPKGAPTPPA
HLA-B55:01	EPLVASGVPA
HLA-A26:01	PTVAVLGGL
HLA-A26:01	EVLALLITAR
HLA-A24:02	EWNGMEWNGI
HLA-A24:02	LWVKQRKDLL
HLA-B55:01	DPNRKPVSRIV
HLA-B55:01	MMKIRSCA
HLA-B35:03	DPPRHQASDL
HLA-DQA105:0	FEPLVASGVPASF
HLA-DQA105:0	DLVSGLSPTVAVL
HLA-DQA105:0	SPKGAPTPPAATF
HLA-DQA105:0	QLCSHYGQPVLSV
HLA-DQA105:0	QPLGIAYLDDFVS
HLA-DRB114:5	RKDLLHLCCKKLK
HLA-DQA105:0	QPYRSAFYVLEQC
HLA-DQA105:0	ALLITARCVVPALI
HLA-DQA105:0	EWNGMEWNGIE
HLA-DQA105:0	IPLRFSVGPLCVEC
HLA-DQA105:0	NWFWEGDDTSF
HLA-DQA105:0	PRHQASDLENEEI
HLA-DRB112:0	EEEMMKIRSCAEI
HLA-C07:01	FRSETTKAQKM
HLA-B35:01	TPSGTDITL
HLA-B35:01	YPCAAGAGSF
HLA-B08:01	VMNHRCCVL
HLA-C07:01	PTYSNRETI
HLA-C07:01	QRTPPPPGK
HLA-B35:01	SPPDVASAAPL
HLA-C07:01	KRLLLMEDD
HLA-B08:01	TSFCKILGPL
HLA-B35:01	HITLQGDPL
HLA-B35:01	QPQGTPPPPG
HLA-C07:01	ISDPEGDDE
HLA-DRB101:0	ITSFCKILGPLSYSK
HLA-DRB101:0	PFHYVTVMNHRC

HLA-DRB101:0	SSAFRSETTKAQK
HLA-DRB101:0	PPDVASAAPLRT
HLA-DRB101:0	TQMYHYPCAAG
HLA-DRB103:0	KAVLETEKRLLLM
HLA-DRB103:0	GTDITLDTTLSAT
HLA-DRB101:0	EVAHITLQGDPL
HLA-DQA105:	RKQLVIYEEISDPE
HLA-DRB101:0	TYSNRETIYPNAS
HLA-DRB101:0	QGGNQSQRTPP
HLA-A03:01	KTYTQSSHLK
HLA-A03:01	SLHGWTGTDK
HLA-A03:01	LLFSNTATQK
HLA-A03:01	SVATYSPWIK
HLA-C07:01	ARIVCGTIM
HLA-C08:02	TGDASKSAL
HLA-C08:02	LADGRPLL
HLA-C08:02	LIDQELLSI
HLA-A03:01	ALMVACTWLH
HLA-C08:02	ISTPDQTEV
HLA-C08:02	TESELATAL
HLA-A03:01	SQGPPPHPGK
HLA-C08:02	GCDVGPDGRPL
HLA-C07:01	RRFVEACI
HLA-B08:01	MERDFLPSL
HLA-C08:02	WAEEPGGHA
HLA-C08:02	QATPTGSGE
HLA-A03:01	LLNESIVANY
HLA-B08:01	SVKESFCYV
HLA-C08:02	GLNNVLGSI
HLA-DQA105:	TESELATALALAS
HLA-DQA105:	CTWLHALTFPAA
HLA-DQA105:	IMAGELSLMAAL
HLA-DQA105:	TVYALAVISTPDQ
HLA-DQA105:	NVLGSIAYGTITE
HLA-DQA105:	RRRFVEACIAREA
HLA-DQA105:	VDISNLEAALWA
HLA-DRB103:0	MEMERDFLPSLK
HLA-DRB113:0	SASLLLNESIVAN
HLA-DRB103:0	GCDVGPDGRPLR
HLA-DQA105:	PGVYTSVATYSP
HLA-DRB113:0	KSKLLFSNTATQK
HLA-DRB103:0	LKKCLIDQELLSIA
HLA-DRB113:0	TDKNFVSKIIAEN
HLA-DRB113:0	DKNFVSKIIAENIY
HLA-DQA105:	ATTGDASKSALLK
HLA-DQA105:	PTGSGEHGAFPV
HLA-DRB113:0	SFCYVNLTCSSGK

HLA-DRB103:0	EGPEDLADGRPLI
HLA-DQA105:0	CDYAGCGKTYTQ
HLA-DQA105:0	PPQGGNQSQGPI
HLA-C07:01	LRHPLSSLM
HLA-C07:01	YRHMLNMKGL
HLA-B15:132	LQVQLFVAF
HLA-C12:03	LANPEKEFL
HLA-C12:03	LASNRLPSL
HLA-C12:03	GALEPGVAL
HLA-A02:05	AMSLLWRV
HLA-B15:132	SQIFIGLTY
HLA-A02:05	WLYYHVLQA
HLA-A02:05	SQGEDCPPV
HLA-A02:05	NLWKMALLAV
HLA-C07:01	IRVSQHKHL
HLA-C07:01	ASILRKRGR
HLA-DRB107:0	FSFLDLESLRSLHL
HLA-DRB107:0	SFLDLESLRSLHLA
HLA-DRB107:0	LDLESLRSLHLASN
HLA-DRB107:0	FLDLESLRSLHLAS
HLA-DRB107:0	MSLLWRVSVTAV
HLA-DRB107:0	WLYYHVLQASEP
HLA-DRB107:0	SPLSIKIFPTNIRVS
HLA-DRB107:0	IFIGLTYYQRLKHN
HLA-DRB107:0	DALGLRHPLSSLM
HLA-DRB107:0	ATFKLCAGSSYRH
HLA-DRB107:0	AGELESGSASILRK
HLA-DRB107:0	RKKIYFKNSENLW
HLA-DRB107:0	ELQVQLFVAFLVI
HLA-DRB107:0	MERMSLKTRLAL
HLA-DRB107:0	SVPTLQKGHQNA
HLA-DQA102:0	PRTLLDYWQALEI
HLA-A02:01	WLFYGVHIL
HLA-A02:01	LLNTLLYSL
HLA-A23:01	MYGMNYFAI
HLA-A02:01	ALVSFTCVSYV
HLA-A02:01	ILLTCLYL
HLA-A02:01	ILPEQSVSI
HLA-A23:01	HYQNSSAFKF
HLA-B44:02	HENHPYPNI
HLA-A02:01	LLPSSVHSA
HLA-A02:01	ALLRGVQCQL
HLA-A02:01	KMPSFGVLA
HLA-A02:01	SLSTNLQENL
HLA-A02:01	KMPEMHFKT
HLA-A02:01	KAFRFPSSV
HLA-A02:01	LVSYQVHKHGV

HLA-B44:02	SEAGTHSTTPV
HLA-A02:01	RNAEITLQMV
HLA-B44:03	KESTLHLVLRR
HLA-A23:01	KSSTTVPSF
HLA-C05:01	FTSSRSFQGHL
HLA-A02:01	RLSGGGGGGT
HLA-C05:01	YSQTDRQDQSY
HLA-B44:02	REKRRRQEL
HLA-A02:01	KLHRSKAG
HLA-DRB107:0	RIKNLLRSPSIKLH
HLA-DRB107:0	WFLHYQNSSAFK
HLA-DRB107:0	VSYVVTKAHPAL\
HLA-DRB107:0	GKAFTSSRSFQGH
HLA-DRB107:0	QIALVYTFVTPLLN
HLA-DRB107:0	FLFVVSYWLFYGV
HLA-DRB107:0	IMRSFSLSTNLQE
HLA-DRB107:0	YNIQKESTLHLVLI
HLA-DRB107:0	GKAFRFPSSVRRH
HLA-DRB107:0	KFKMPKFKMPSF
HLA-DRB107:0	AISISSIPLLPSSVH
HLA-DRB107:0	MNYFAIRNKKGT
HLA-DRB107:0	ILLTCLYLSYSYMG
HLA-DRB107:0	QHNLVSYQVHKH
HLA-DRB107:0	LKMPEMHFKTPK
HLA-DRB107:0	WVFLVALLRGVQ
HLA-DQA102:0	DQGRNAEITLQM
HLA-DRB107:0	QTCFSLELRGPPIL
HLA-DRB107:0	HANGLAKSSTTVI
HLA-DQA102:0	PVSTTPVVSSEAG
HLA-DRB107:0	QIPWRPSQPNIHI
HLA-DQA102:0	EEVQQEEEQLLRE
HLA-DRB107:0	MSRRAPGSRLSG
HLA-DQA102:0	DRQGQSSHYSQT
HLA-C12:03	KAFNQSSHL
HLA-C12:03	YGYGCYCPSYY
HLA-C12:03	DPYRVHYTV
HLA-C12:03	SPFHPIRRV
HLA-B18:01	EEYLNFVDY
HLA-C12:03	TPYQTPKSV
HLA-C12:03	FSLRVHSV
HLA-C12:03	VIWHWTQVV
HLA-C12:03	IAAAQSGLLV
HLA-A03:01	KSMLARLSK
HLA-C12:03	KSQDLGSII
HLA-C12:03	KALSHSSSF
HLA-C12:03	FQNGKMASL
HLA-A25:01	HTILTTSNSF

HLA-C12:03	FIRTPQTL
HLA-A03:01	IIHTGEKPYK
HLA-A03:01	LMGAQRACPK
HLA-C12:03	FMWAPGPI
HLA-C12:03	IARYVHIVPL
HLA-A03:01	ITGLATCIHK
HLA-C08:02	LLDEKGPEV
HLA-A03:01	ATGGSWSQK
HLA-C12:03	ITQSGDLSY
HLA-C12:03	YQALVHSV
HLA-C12:03	WLHKRGKYI
HLA-C12:03	YVISGNGNGKL
HLA-C12:03	MALLTSNSCFI
HLA-A03:01	AIHQQKAQGK
HLA-C12:03	WQFGHQTGYF
HLA-C12:03	STWDKVTDAV
HLA-C12:03	DARDGPSHS
HLA-C08:02	LIDGTETTA
HLA-C12:03	FVGHQGAV
HLA-C12:03	YSQTDRQDQSY
HLA-C08:02	NSDDSSNDN
HLA-C08:02	SSDSEVHSG
HLA-C08:02	VTQKWASRL
HLA-C08:02	SPDRNPELS
HLA-A25:01	STPLPLSSG
HLA-DRB113:0	RGKYIKTWRPRY
HLA-DQA105:0	IALKLIAAAQSGLL
HLA-DRB113:0	LSPFHPIRRVAVK
HLA-DRB113:0	TSNSCFIRCNEAG
HLA-DQA105:0	RDGPSHSAGANL
HLA-DQA105:0	KGAALVSPISKSM
HLA-DQA105:0	PLHSAPITGLATC
HLA-DRB113:0	KTKIFIRTPQTLFT
HLA-DRB113:0	QFERFNQANTIL
HLA-DQA105:0	LEGHVHMAAVD
HLA-DQA105:0	RNKLGHSRASVIV
HLA-DRB113:0	GKALSHSSSFRRH
HLA-DRB113:0	QIGNLFSLRVHSV
HLA-DRB113:0	TLLLVMLMGAQ
HLA-DQA105:0	TRKSGATGGSWS
HLA-DQA105:0	CKGTFVGHQGA
HLA-DRB113:0	GKAFNQSSHLTR
HLA-DQA105:0	VEWYQALVHSV
HLA-DRB113:0	SWDYKVTQKWA
HLA-DQA105:0	PPFMWAPGPIH
HLA-DRB113:0	CVIGHIAKHLPKS
HLA-DRB113:0	MSYVISGNGNG

HLA-DRB113:0	RHTILTTSNSFPYV
HLA-DQA105:0	YCPSYYGGYGFSG
HLA-DQA105:0	NNFTKSGSTPLPL
HLA-DQA105:0	QTPKSVRRGVAPY
HLA-DQA105:0	GNDIFQAVETAIH
HLA-DRB113:0	LQHPIIARYVHIVP
HLA-DQA105:0	RVHYTVPYAEGVY
HLA-DQA105:0	SDSEVHSGGSHRI
HLA-DRB113:0	FWQFGHQTGYFY
HLA-DQA105:0	KEKEGVLHVPITQ
HLA-DQA101:0	QLEEYLNFVDYFC
HLA-DRB113:0	SNLTKHKIIHTGEK
HLA-DQA105:0	LLDEKGPEVLQDS
HLA-DQA105:0	GQTDRQGQSSHY
HLA-DQA105:0	PDRNPELSSSRDR
HLA-DQA105:0	SNDNSDDSDVPD
HLA-A31:01	RSRRQLHHR
HLA-A02:01	YLFDLKAFFM
HLA-A02:01	SLTGLHFKI
HLA-A31:01	HVSSNSTR
HLA-A02:01	NLQTPMYFFL
HLA-C15:02	LTLERNPMSV
HLA-A02:01	AIVDFVGWL
HLA-C15:02	RSTDLTTHKII
HLA-A02:01	RIIWCPFV
HLA-C15:02	RSTDLTTHKKV
HLA-A31:01	QLRREQELR
HLA-A31:01	ANTTVGHIR
HLA-C15:02	ATATIVFI
HLA-A31:01	TSVPWIKFK
HLA-A02:01	WMAGDETQPT
HLA-C15:02	NSDDSEAPD
HLA-DRB104:0	LCMILLIRTNSNLC
HLA-DQA103:0	AADATATIVFIEDI
HLA-DRB104:0	DLKAFFMSKALNI
HLA-DRB104:0	KAFNRSTDLTTHK
HLA-DRB104:0	LKCLVFSLTGLHFK
HLA-DRB104:0	MKGLTLERNPMS
HLA-DRB104:0	VPWIKFKSNCYSP
HLA-DRB104:0	EVKFVWMAGDE
HLA-DRB104:0	FVGWLCAVSMDI
HLA-DRB104:0	LNHFRRIIWCPFV
HLA-DRB104:0	TFWCSLQRSRRQ
HLA-DRB104:0	APNFEANTTVGH
HLA-DRB104:0	HINSNHVSSNSTR
HLA-DQA103:0	PDDNSDDSEAPD
HLA-DQA103:0	LRREQELRREQEE

HLA-C12:03	KAFRFPSSV
HLA-A02:01	VLQGMAFAL
HLA-C12:03	AAFPRGVPGAL
HLA-A02:01	SMSVSTMSV
HLA-A02:01	YMVTERTTYI
HLA-C12:03	PTYSGRETV
HLA-C12:03	KEFQSLETV
HLA-A02:01	GLTRSLGPPV
HLA-A02:01	YLARALFTC
HLA-A02:01	VLWAVCYCC
HLA-C12:03	YVHQVGRTA
HLA-A02:01	SLLNHMANYT
HLA-C12:03	SQMLASSTM
HLA-A02:01	SLSKGDALL
HLA-C12:03	FGSGYVLKTV
HLA-B38:01	EHSTFDVL
HLA-C12:03	VTAEILEPGTL
HLA-C12:03	KSISTRSVV
HLA-A02:01	ALQAQPLRPQL
HLA-C12:03	RAYQRAAEK
HLA-C12:03	YSDDMPLPT
HLA-C12:03	YPSEECEVT
HLA-A02:01	VLDDEDRNL
HLA-C12:03	KVFRVNKV
HLA-C12:03	FVQSGPTKGF
HLA-C12:03	VTAGQERFRAV
HLA-A02:01	KSLLTSFPKSV
HLA-C12:03	SSDEKDSVL
HLA-C12:03	RVHTEKKHY
HLA-A02:01	VMQELEDYS
HLA-C12:03	SVDRSVHSG
HLA-C12:03	SKDPPGQEA
HLA-A66:01	ETQRFYACS
HLA-DRB111:0	SVFLRFRTRAYQR
HLA-DRB111:0	STFDVLDRRRLG
HLA-DRB111:0	HSTFDVLDRRRL
HLA-DQA105:	GALWARVARAE
HLA-DQA105:	DLLGGLSGGSIQ
HLA-DQA105:	QVGRTARAGRA
HLA-DQA105:	VSTMSVASSEAS
HLA-DQA105:	GLTRSLGPPVALA
HLA-DQA101:	CLHYLAQALQAQ
HLA-DRB111:0	SGKIVIARYGKVF
HLA-DQA105:	STRSVVESIASPA
HLA-DRB111:0	SLLYRLKFKEFVQ
HLA-DQA105:	LKTVQSSPGVAK
HLA-DRB111:0	EVVLYLARALFTC

HLA-DQA105:0	MSLSKGDALLAL\
HLA-DQA101:0	ASNTMDSQMLA
HLA-DQA105:0	SLENGGPSSLESK:
HLA-DRB111:0	KQCGKAFRFPSS\
HLA-DQA105:0	RNTTNTSVTAEILI
HLA-DRB111:0	TLLFVKVTQGKEE
HLA-DRB111:0	RPKVSSLLNHMA
HLA-DQA105:0	TSYVVDGQIIIYGF
HLA-DRB111:0	KYASFSNHQRVH
HLA-DQA105:0	KLQIWVTAGQER
HLA-DQA101:0	VDLSSNNISELQT
HLA-DRB111:0	NKSLLTSFPKSVEI
HLA-DRB111:0	HNKYMVTERTTY
HLA-DQA101:0	FRNQDVQKVLW
HLA-DQA101:0	VKEMLKSVMQEL
HLA-DQA105:0	WKEGREIARVLDI
HLA-DQA105:0	EIIIYNGYPSEECE
HLA-DQA105:0	VDRSVHSGSHHS
HLA-DQA105:0	SSDEKDSVLNIAR
HLA-DRB111:0	RDRDRSVRGKYSI
HLA-B08:01	GASGRFVAL
HLA-C07:01	TDFGHSEIL
HLA-C07:01	LRVQEERLH
HLA-B57:01	TTAFMGYVLPW
HLA-C06:02	QRKCLISSV
HLA-B57:01	MALAGQPGGPF
HLA-A01:01	FSDSRIALK
HLA-C07:01	SNYPAPRAV
HLA-B57:01	ITALATLHLLF
HLA-B08:01	SLKVLTITNCM
HLA-A01:01	QSEKGVIQVY
HLA-C07:01	KRTALIKAI
HLA-A01:01	YQENITNTCY
HLA-C06:02	MVNEGVKAD
HLA-DRB101:0	IGIILLLATMTTAF
HLA-DRB101:0	PFIITALATLHLLFL
HLA-DRB101:0	KGVFLGASGRFV
HLA-DRB101:0	AGGLRLTPGGM
HLA-DRB101:0	NTCYKYLHEALM
HLA-DRB101:0	FMAVKFSDSRIAL
HLA-DRB101:0	KTSLKVLTITNCM
HLA-DRB101:0	HSEILGETSLMRT
HLA-DRB101:0	AQALVEQMVNE
HLA-DRB101:0	SRNILYTQSEKGV
HLA-DRB101:0	NSSNYPAPRAVLT
HLA-DQA101:0	KKRTALIKAIQCQI
HLA-DQA101:0	RKCLISSVEDFTEF

HLA-DRB101:0	QEERLRVQEERLH
HLA-C05:01	VLDTALSLL
HLA-C15:02	MSMHTTMTTL
HLA-B44:02	QEVEVNRKW
HLA-B44:02	QEVEVNCNW
HLA-B44:02	QENDMMEVPY
HLA-B44:02	LEMYGMNYFAI
HLA-B44:02	AEGIGIVLF
HLA-A01:01	FLICLLVLY
HLA-C05:01	VLDGEEARTI
HLA-C15:02	HSPGAFGNM
HLA-C05:01	TCDFADGER
HLA-C05:01	GGNQPQGTP
HLA-B44:02	KEHQQQRRQ
HLA-DRB107:0	LLVLYALFLTLTTK
HLA-DRB107:0	GAWAGVLDTALS
HLA-DQA101:0	EVNCNWILPILTQ
HLA-DRB107:0	SMHTTMTTLTLTS
HLA-DQA102:0	WNIGLAEGIGIVL
HLA-DRB107:0	GAFGNMERITSPY
HLA-DRB107:0	MNYFAIRNKKGT
HLA-DQA102:0	GREEARAVLDGE
HLA-DQA102:0	YFEQENDMMEV
HLA-DQA102:0	ERVWLWTTCDFA
HLA-DRB107:0	AKLTRARDKEHQ
HLA-DQA102:0	PPPQGGNQPQG
HLA-B15:03	KKIVHSVVSSF
HLA-B15:03	SKMGKWFHPRF
HLA-B15:03	AMQELENY
HLA-B15:03	CQEDERVLM
HLA-B15:03	GQLHEVPV
HLA-B15:03	KERLKSVM
HLA-B15:03	VAFSLACII
HLA-B35:01	LPQGTPTSL
HLA-C07:01	LRVQEERLH
HLA-B15:03	KKIPEVGGKEL
HLA-B15:03	SRTCQLDQF
HLA-B15:03	AKRVPTAPAAI
HLA-A30:02	KTDKAVECY
HLA-B35:01	EAMIFGIGL
HLA-B35:01	LPPPTPAL
HLA-C07:01	ASGLPDKEE
HLA-B35:01	KPTPTTPEE
HLA-C07:01	EEDEDEEEE
HLA-DQA105:1	AKRVPTAPAAISK
HLA-DQA105:1	RRQVTVAPASGL
HLA-DRB109:0	IKKIVHSVVSSFAF

HLA-DQA105:1	PPTPALGPPAPR(
HLA-DQA105:1	AHRFLGLLYELEEI
HLA-DQA105:1	IKVEVAFSLACIILC
HLA-DRB109:0	QFHHSRGSPFKKI
HLA-DQA105:1	ALAHFLPQGTPTS
HLA-DRB111:0	FDLRSKMGKWFH
HLA-DRB111:0	LESVKEMLKSAM
HLA-DQA105:1	LDQFSCGNGRCIF
HLA-DQA105:1	EAMIFGIGLCDN(
HLA-DRB109:0	NKKVGQLHEVPV
HLA-DQA105:1	ALIKAVQCQEDEF
HLA-DQA105:1	REQEERLRVQEEF
HLA-DQA105:1	TTPEELAPTTPEKI
HLA-DQA105:1	EEEEEDEEDEDEE
HLA-A68:02	TTAAPPTPPA
HLA-A68:02	HSAHCSFYL
HLA-A68:02	TVSKVTVQSV
HLA-A68:02	EAERVISSV
HLA-A68:02	TAIRCFGVYV
HLA-A68:02	DTSSASTGQA
HLA-C08:02	VLDSLENLL
HLA-A68:02	LSASASYAA
HLA-A68:02	ETLTPYIPM
HLA-A68:02	LLTTLTFHV
HLA-A68:02	ETPAIKEPD
HLA-A24:02	SFMSNNGPF
HLA-C08:02	NTDSAGTL
HLA-C08:02	QIDKLWAEL
HLA-A68:02	QVSSSSLVRI
HLA-B14:02	FKRRRLNRI
HLA-C08:02	HSDEQNDTQ
HLA-A68:02	DTTDDLSML
HLA-A68:02	VTNPPDVDGV
HLA-A68:02	HSSWKNVPSI
HLA-B14:02	TRRCASPSNSM
HLA-A68:02	HTHDQVVMFF
HLA-B35:02	NPDPDSLQLF
HLA-B14:02	KAARRSTEA
HLA-C08:02	YIDDQKKKC
HLA-C08:02	FLEKVLEL
HLA-A68:02	KVRKIQNHI
HLA-A68:02	FVCHRTQKEI
HLA-C08:02	IGQGPPLPP
HLA-C08:02	KISPHGLET
HLA-DRB101:0	VRIFQLLLSHKDSI
HLA-DRB101:0	HCSFYLTRTLQTLS
HLA-DRB101:0	LEKVLELSGWRA\

HLA-DRB101:0	VSEYLKISPHGLET
HLA-DRB101:0	QVVMFFKASRES
HLA-DRB101:0	SLQLFNVRGVLRH
HLA-DRB101:0	LGCHLLTTLTFHV
HLA-DRB101:0	LRQIDKLWAELRF
HLA-DRB101:0	MNKVRKIQNHIQ
HLA-DRB101:0	VPSINRLKSTLDP
HLA-DRB101:0	YVSNITAIRCFGV
HLA-DRB101:0	RLFKNLSLIKKYID
HLA-DRB101:0	QVTISGAAGAAA
HLA-DRB101:0	AERVISSVISVSM
HLA-DRB101:0	DTTDDLSMLGRV
HLA-DRB101:0	ASSFKRRRLNRIP
HLA-DRB101:0	LDSFMSNNGPFC
HLA-DRB101:0	ISEVLDSLENLLRH
HLA-DRB101:0	ANSFTVSKVTVQ
HLA-DRB101:0	MAFKWRGKPLF
HLA-DRB101:0	SPSNSMQLASRS
HLA-DRB101:0	KIYVTIPKKRETPA
HLA-DRB101:0	SENSVIGQGPPLP
HLA-DRB101:0	TLGTLRFCVFGLG
HLA-DRB101:0	ELDLETLTPYIPMI
HLA-DRB101:0	RLKEQEGRKAAR
HLA-DRB101:0	SASTGQATPLPVT
HLA-DRB101:0	NGTYTCQVTNPP
HLA-DRB101:0	TQKQLSEEQNTG
HLA-DRB101:0	PTPPATTPAPLSS
HLA-A68:02	MVILATSTV
HLA-A24:02	KYLMFLFNF
HLA-A68:02	TTAEGTDIPI
HLA-A68:02	TVSKVTVQSV
HLA-A68:02	EAERVISSV
HLA-C08:02	VLDSLENLL
HLA-A68:02	LSASASYAA
HLA-A68:02	DIIKPMFVI
HLA-A68:02	ETLTPYIPM
HLA-C08:02	ITDFGHSEI
HLA-A68:02	MNVRVMSTI
HLA-A68:02	DMVLVTMVV
HLA-A68:02	ETPAIKEPD
HLA-B14:02	WLHRRLPL
HLA-C08:02	MDDWELIPF
HLA-C08:02	DVDCILDEL
HLA-A68:02	QVSSSSLVRI
HLA-B14:02	FKRRRLNRI
HLA-C08:02	FQDGAIQEAV
HLA-C08:02	STDNNQTYP

HLA-C08:02	HSDEQNDTQ
HLA-C08:02	RSEYRGALL
HLA-A68:02	HSSWKNVPSI
HLA-C08:02	SSDWSQRFP
HLA-B14:02	TRRCASPSNSM
HLA-A68:02	HTHDQVVMFF
HLA-A68:02	DVIRPGMWRKV
HLA-A68:02	HIHKQQMVSV
HLA-C08:02	WDDDRDEKT
HLA-C08:02	GCCKRQAEL
HLA-B14:02	PRYRWRTQS
HLA-A68:02	CTHTQRSPQSL
HLA-A68:02	KVRKIQNHI
HLA-A24:02	SYHKKIHTG
HLA-C08:02	KISPHGLET
HLA-B14:02	NHQRVHTEK
HLA-A68:02	DIRREMYII
HLA-B14:02	LQQKPGDSTM
HLA-DRB101:0	VRIFQLLLSHKDSL
HLA-DRB101:0	VKMNVRVMSTIL
HLA-DRB101:0	GMDMVILATSTV
HLA-DRB101:0	RLPLQPIRPLPRF
HLA-DRB101:0	VSEYLKISPHGLET
HLA-DRB101:0	RREMYIIKEGKLA
HLA-DRB101:0	QVVMFFKASRES
HLA-DRB101:0	IIKPMFVINEMRR
HLA-DRB101:0	HSEILGETSLMRT
HLA-DRB101:0	MNKVRKIQNHIQ
HLA-DRB101:0	LFNFFIFLGGACLL
HLA-DRB101:0	VPSINRLKSTLDP
HLA-DRB101:0	NFEYTLEASKSLQ
HLA-DRB101:0	QVTISGAAGAAA
HLA-DRB101:0	AERVISSVISVSM
HLA-DRB101:0	KFLLFDMVLVTM
HLA-DRB101:0	RSEYRGALLWMK
HLA-DRB101:0	DVHIHKQQMVSV
HLA-DRB101:0	ASSFKRRRLNRIPI
HLA-DRB101:0	ISEVLDSLENLLRH
HLA-DRB101:0	ANSFTVSKVTVQS
HLA-DRB101:0	QEAVVWEAASM
HLA-DRB101:0	IKTADLKVVLGKF
HLA-DRB101:0	DFFLLLSKFGCCK
HLA-DRB101:0	SPSNSMQLASRS
HLA-DRB101:0	KIYVTIPKKRETPA
HLA-DRB101:0	VVFKDVIRPGMW
HLA-DRB101:0	LQQYGYLPPGDL
HLA-DQA101:0	EMDDWELIPFVV

HLA-DRB101:0	SKYASFSNHQRVI
HLA-DRB101:0	QTYPSIQIMNYYG
HLA-DRB101:0	KWSSTLSYHKKIH
HLA-DQA101:(	AWAKLDVDCILD
HLA-DRB101:0	ELDLETLTPYIPMI
HLA-DRB101:0	QPRYRWRTQSSG
HLA-DRB101:0	SSDWSQRFPRMS
HLA-DRB101:0	TQKQLSEEQNTG
HLA-DRB101:0	TDIPISTPSEGSTP
HLA-A02:01	FAFDCPYLV
HLA-B07:02	LPRQAKTAA
HLA-A02:01	LLAEKEPEV
HLA-A02:01	SLLNVYYII
HLA-A02:01	SMPVSTMPV
HLA-A03:01	RIHTGEKPYK
HLA-A03:01	KIHTGEKPYK
HLA-A02:01	ALSFADVAV
HLA-A02:01	FTIFHLHSL
HLA-B07:02	TPSERQLTPL
HLA-A03:01	IIHTGEKPYK
HLA-A02:01	NLLSTHDAVV
HLA-A02:01	IQWNIRAFNV
HLA-A03:01	KTFSIFSTLTK
HLA-C07:01	RRFSLAEFV
HLA-A02:01	YLDTTKGAV
HLA-A02:01	QLAGIDLKV
HLA-B07:02	SPEKPTIPT
HLA-B08:01	SILIKHKVI
HLA-C07:01	NRQGQSSHY
HLA-A02:01	ALEEQHVGFSL
HLA-C07:02	YKNPNLTVSF
HLA-A02:01	VAMQDPVFV
HLA-A02:01	ALADDMEL
HLA-B08:01	LKKSKLKEL
HLA-B08:01	MLRAKPPPP
HLA-A03:01	KGRGPFWWK
HLA-A02:01	ALNGNKLNINL
HLA-A02:01	STGQATPLPV
HLA-C07:01	DRQGQSSHY
HLA-C07:01	IHGERKEFM
HLA-C07:01	SHYSQTDRQ
HLA-A02:01	EVACDILSQLV
HLA-B08:01	HLKNRENCSC
HLA-B07:02	QPSCCQTSSC
HLA-C07:01	KRIHTGEKP
HLA-B07:02	APSSTTAE
HLA-B07:02	FPMALFGK

HLA-A02:01	AVAASKECNGL
HLA-B07:02	PPQQPGSTK
HLA-A03:01	CGYGSGWGY
HLA-C07:01	SSAADGDEE
HLA-C07:02	SYHKKIHTG
HLA-A02:01	SEAGTHSTTPV
HLA-C07:02	EGDAPGPRG
HLA-C07:01	EAEGDAPGP
HLA-DRB108:0	FNRFTTLTKHKRII
HLA-DRB108:0	FSKFSILIKHKVIHT
HLA-DRB108:0	TFSIFSTLTKHKIIH
HLA-DRB115:0	NVYYIIILAWATYY
HLA-DRB108:0	FSRFSYLTAHKIIH
HLA-DRB108:0	CNGLSLAALKKAL
HLA-DRB108:0	SEMKMRLPRQAI
HLA-DRB108:0	PYLVVKITPAIPAV
HLA-DRB115:0	AFNVVKNWSWN
HLA-DRB108:0	SSTLIKHKKIHTGE
HLA-DRB108:0	TKGAVETFMKLR
HLA-DRB108:0	THDAVVQCRKRN
HLA-DRB108:0	PGEGVLMLRAKP
HLA-DRB108:0	FINFVKNYKNPNL
HLA-DRB108:0	GDKHKLKKSKLKE
HLA-DRB115:0	PAHFVALNGNKL
HLA-DRB108:0	LRHSACRRFSLAE
HLA-DRB115:0	HYLQFTIFHLHSLE
HLA-DRB108:0	KWSSTLSYHKKIH
HLA-DRB108:0	SILTTHKRIHTGEK
HLA-DQA101:0	PLASMPVSTMPV
HLA-DQA104:0	GALSFADVAVYFS
HLA-DQA104:0	IESSYQVAKALAD
HLA-DQA101:0	SVPVSTMPVVSSI
HLA-DQA104:0	LKAFHSDVAMQD
HLA-DRB108:0	VENYSNVSIHLKN
HLA-DQA101:0	RVNLTLADLEQLA
HLA-DQA104:0	SSKESAEVACDILS
HLA-DQA104:0	VTDTSSASTGQAT
HLA-DQA101:0	QPYGSSFYALEEC
HLA-DQA104:0	PSSTTAEGTSIPTS
HLA-DQA104:0	DLASCDLTSSAAD
HLA-DRB108:0	ATSPRLSRELLAEK
HLA-DRB108:0	MCHWGGCSREL
HLA-DQA104:0	PRGEAEGQAEAE
HLA-DRB108:0	VNFPQRKKKGRG
HLA-DQA104:0	SNSDGEDFPMAL
HLA-DRB108:0	PSERQLTPLPPSA
HLA-DQA104:0	GWGYGCGYSSSC

HLA-DRB115:0	SQTDRQDQSYHY
HLA-DRB108:0	GQCYHYDQTNR(
HLA-DQA104:(	TSSCGTGCGIGG(
HLA-DRB115:0	TNRQGQSSHYSC
HLA-DQA104:(	KPTIPTEKPTIPTEI
HLA-DQA104:(	QQPGSTKPPSQC
HLA-A02:01	YLMTFLIMTV
HLA-A02:01	WLWALQLIFV
HLA-B07:02	LPRQAKTAA
HLA-A02:01	HLPTCVMPYV
HLA-A02:01	VLITLPIPI
HLA-A03:01	AMHGFPTLK
HLA-A02:01	SLVHYADTV
HLA-B07:02	RPVSPRCSLL
HLA-A02:01	ALLTSNSCFI
HLA-A02:01	SMLTPLQSI
HLA-B07:02	TPSERQLTPL
HLA-A02:01	YLEHVVVCI
HLA-A02:01	NLLSTHDAVV
HLA-C07:01	YRITNHSHFWM
HLA-B07:02	KPCYPKASV
HLA-A03:01	TISALNIRK
HLA-A02:01	QLAGIDLKV
HLA-B08:01	SILIKHKVI
HLA-B08:01	YNMNKQCEL
HLA-A02:01	VLDFMETDLEV
HLA-A02:01	FMGWADDII
HLA-A02:01	TVAELLVTI
HLA-A02:01	GLLGVGIWLSM
HLA-C07:02	YKNPNLTVSF
HLA-A02:01	VAMQDPVFV
HLA-C07:01	LRFGRSWI
HLA-A03:01	LTYQDQSDNK
HLA-C07:01	WRSEVDKQL
HLA-C07:01	SNNSGLRLL
HLA-B07:02	GPPPADLCHAL
HLA-A02:01	KVFNDSGNLSI
HLA-C07:01	KSFNHSSNL
HLA-C07:02	SRLKVGDQIL
HLA-A02:01	KLKCGTCGA
HLA-C07:01	SHYSQTDRQ
HLA-B07:02	SASSGDTTPL
HLA-A02:01	VICEVAHITL
HLA-A02:01	EVACDILSQLV
HLA-A03:01	RQNLPEKRYPK
HLA-B07:02	GPKVKGDADV
HLA-A02:01	NGIDMAYRV

HLA-C07:01	SSAADGDEE
HLA-DRB108:0	FSKFSILIKHKVIHT
HLA-DRB108:0	QLKFRKKKLRFGR
HLA-DRB108:0	SEMKMRLPRQA
HLA-DRB108:0	LIMTVAWAAHT
HLA-DRB108:0	SAITYRITNHSHF
HLA-DRB108:0	VVVCITITHPRRG
HLA-DRB108:0	HLQFLRCISPHSD
HLA-DQA101:0	VISNNSGLRLLID
HLA-DRB108:0	ENTISALNIRKQT
HLA-DRB108:0	KAAMFILTRNVLI
HLA-DRB108:0	THDAVVQCRKR
HLA-DRB108:0	FINFVKNYKNPNL
HLA-DRB108:0	KQMWQYLKPCP
HLA-DQA101:0	PVFQNGKMALLT
HLA-DRB108:0	QSDNKLYRLQTP
HLA-DRB115:0	WLWALQLIFVST
HLA-DRB108:0	NGIDMAYRVLAD
HLA-DRB108:0	CYPKASVQYYKKI
HLA-DRB108:0	AFAMHGFPTLKY
HLA-DQA101:0	VICEVAHITLQGD
HLA-DQA104:0	VPSQADVAVFEA
HLA-DQA101:0	LLGVGIWLSMSQ
HLA-DRB108:0	AELLVTICSRIGIT
HLA-DRB108:0	TCGAIGHMRTNK
HLA-DRB115:0	HSSNLIQHQSIHA
HLA-DQA104:0	LKAFHSDVAMQ
HLA-DRB108:0	DSGNLSIHKRIHT
HLA-DQA101:0	RVNLTLADLEQL
HLA-DQA101:0	KPEAHFSLVHYAD
HLA-DQA104:0	SSKESAEVACDIL
HLA-DRB115:0	PVSPRCSLLLTHY
HLA-DQA101:0	KSNISLVLDFMET
HLA-DRB115:0	DVDLHLKGPKVK
HLA-DRB108:0	LPGILYNMNKQC
HLA-DRB115:0	WADDIINSSGYRI
HLA-DQA104:0	DLASCDLTSSAAD
HLA-DQA104:0	PYVITSGITEQER
HLA-DRB115:0	NERLWAWESW
HLA-DQA104:0	PLPVTSTSSASSG
HLA-DQA101:0	PGSEAEGSRLKV
HLA-DRB108:0	PSERQLTPLPPSA
HLA-DRB115:0	SQTDRQDQSYHY
HLA-A02:01	VLIEFCHLV
HLA-A02:01	FLLDPVLAGI
HLA-A02:01	YLTYLLLWV
HLA-C05:01	LADDHMALV

HLA-A24:02	FYYDYNFISF
HLA-A02:01	FLQCLGRFV
HLA-A02:01	SMLEWHLSTHM
HLA-B44:02	MEFHKLQSAF
HLA-A02:01	ALLITARCVV
HLA-B44:02	AEELYKQALEI
HLA-A02:01	AMARGCFLNA
HLA-A02:01	MLNPANIRSI
HLA-A02:01	PMFNPLIYAL
HLA-B44:02	QEGLCVHVPF
HLA-A24:02	KYIGTNAEI
HLA-A02:01	KLQESGEVQV
HLA-A02:01	YLTPIKHL
HLA-A24:02	AYWPDWNRL
HLA-A24:02	LYPTPRVRAMI
HLA-C05:01	GSDKSNVGT
HLA-B44:02	AEFASKPAA
HLA-C05:01	YLDQFQTKE
HLA-C05:01	SQDVGVVHI
HLA-B44:02	AELRDTRKHAV
HLA-A02:01	KLVIYHGWTRT
HLA-C05:01	CGDRTSGYH
HLA-C05:01	LQDSLDRCY
HLA-A24:02	KFSKMQAF
HLA-A02:01	ILLLTLTAQEP
HLA-B44:02	RELCRQDAKGL
HLA-A02:01	LIFKQVHGFAT
HLA-A02:01	GLSGGAAPPP
HLA-A02:01	TVSSGISTA
HLA-C05:01	LCETHETGT
HLA-B18:01	TEVNSPPPQ
HLA-C05:01	AAGHQKAFG
HLA-B44:02	VEVIKVRNS
HLA-DQA105:0	LAALVAPATAATA
HLA-DQA105:0	GVGLSGGAAPPP
HLA-DQA105:0	ESSTVSSGISTATN
HLA-DRB104:0	LDRFCSALSSMLE
HLA-DQA105:0	SPLHSRSIAALADI
HLA-DRB104:0	FMEFHKLQSAFVI
HLA-DQA105:0	TSNAGSAQRVCS
HLA-DRB104:0	PANIRSISNMTSC
HLA-DRB104:0	ANIRSISNMTSQE
HLA-DQA105:0	GRSTGKGIAEFAS
HLA-DQA105:0	LDPVLAGINSLSSE
HLA-DRB103:0	NKVILSGLRPFRS(
HLA-DRB103:0	TPIKHLRKSLKIIYI
HLA-DRB104:0	NPLIYALRNTEMK

HLA-DQA105:0	NFWMVWSEAM
HLA-DRB104:0	TQAVLRIKSAAGH
HLA-DQA105:0	YYDYNFISFHEDLS
HLA-DRB103:0	YLLLWVKQRRDLI
HLA-DQA105:0	TSKYIGTNAEITYL
HLA-DRB103:0	RELCRQDAKGLFE
HLA-DRB104:0	GRKFSKMQAFRIV
HLA-DQA105:0	DVEGIGEEVLALL
HLA-DRB104:0	GFKLKEVSSVNSP
HLA-DRB104:0	GSLNILLLTLTAQE
HLA-DRB104:0	LQCLGRFVNTPFL
HLA-DRB104:0	LILIFKQVHGFATN
HLA-DQA105:0	VRNSRSRGGGEG
HLA-DRB104:0	EVQVHKLQSLKKV
HLA-DQA105:0	LIEFCHLVGDCELS
HLA-DRB104:0	DFYLDQFQTKENS
HLA-DRB103:0	LCVVCGDRTSGYH
HLA-DRB104:0	PRVRAMIESLNQI
HLA-DQA105:0	TRKHAVEVTLDPE
HLA-DQA105:0	LVIYHGWTRTTKI
HLA-DQA105:0	LVTVQEGLCVHVI
HLA-DRB103:0	EPEVLQDSLDRCY
HLA-DRB104:0	ELYKQALEISENAY
HLA-DQA105:0	FAWCRGSDKSNV
HLA-A02:01	VLIEFCHLV
HLA-A02:01	NLLSTIPYI
HLA-C05:01	YTDFDETRV
HLA-A02:01	VLDELSVTYV
HLA-A02:01	LLIEAPIRMMI
HLA-A02:01	FLQCLGRFV
HLA-A02:01	KMCHDYPQLV
HLA-A02:01	SILEWEHLDFI
HLA-A02:01	SMLEWHLSTHM
HLA-A02:01	IMIPKLCMQL
HLA-B44:02	AEELYKQALEI
HLA-C05:01	SVDVVNMF
HLA-A02:01	TLYAICDV
HLA-A24:02	KYIGTNAEI
HLA-A02:01	ALWLQKQQLV
HLA-A02:01	HMAEKTFLC
HLA-A02:01	GLTYKFHV
HLA-A24:02	LYPTPRVRAMI
HLA-A02:01	PLLPFYELDGA
HLA-B44:02	AETAKGYLL
HLA-A02:01	FIKECREDYYL
HLA-C05:01	YLDQFQTKE
HLA-C05:01	TSDEKDSVSNT

HLA-C05:01	SQDVGVVHI
HLA-B44:02	AELRDTRKHAV
HLA-A02:01	KLVIYHGWTRT
HLA-A24:02	KFSKMQAF
HLA-C05:01	QVDSCHPQG
HLA-A02:01	PLGNCPAHPL
HLA-A02:01	ILLLTLTAQEP
HLA-B44:02	RELCRQDAKGL
HLA-C05:01	PANSPPPPA
HLA-C05:01	VGNTKPAET
HLA-DQA105:0	FYELDGAPTAAQ[illegible]
HLA-DRB104:0	LDRFCSALSSMLE
HLA-DQA105:0	MSFWGATVITNL
HLA-DQA105:0	TYKFHVAWSSVL[illegible]
HLA-DRB103:0	RVVLLIEAPIRM[illegible]
HLA-DRB103:0	NKVILSGLRPFRS[illegible]
HLA-DQA105:0	GRISAHQGAQVD
HLA-DRB103:0	IIKMCHDYPQLVL
HLA-DQA105:0	VTYVESFQVIIEE[illegible]
HLA-DRB103:0	RLVLSVDVVNMF
HLA-DQA105:0	VFHESILEWEHLD
HLA-DQA105:0	TSKYIGTNAEITYL
HLA-DRB103:0	RELCRQDAKGLF[illegible]
HLA-DRB103:0	RPTFVKNHRVH[illegible]
HLA-DRB104:0	GRKFSKMQAFRI[illegible]
HLA-DRB104:0	GSLNILLLTLTAQ[illegible]
HLA-DRB104:0	YYLGRLRSQESYS[illegible]
HLA-DQA105:0	LIPIPANSPPPPAL
HLA-DRB104:0	LQCLGRFVNTPFL
HLA-DRB103:0	KDTVYTDFDETR[illegible]
HLA-DRB103:0	LKDTVYTDFDET[illegible]
HLA-DQA105:0	PKHQPAAETAKG
HLA-DQA105:0	LIEFCHLVGDCEL[illegible]
HLA-DQA105:0	PLKPVGNTKPAE[illegible]
HLA-DRB104:0	DFYLDQFQTKEN[illegible]
HLA-DRB104:0	PRVRAMIESLNQ[illegible]
HLA-DQA105:0	TRKHAVEVTLDP[illegible]
HLA-DRB104:0	IPKLCMQLTVGS[illegible]
HLA-DQA105:0	LVIYHGWTRTTKI
HLA-DQA105:0	HPLPPTSDATSPT
HLA-DQA105:0	TLYAICDVFTQYL[illegible]
HLA-DQA105:0	TATEIKEGQQSG[illegible]
HLA-DRB104:0	ELYKQALEISENA[illegible]
HLA-DRB104:0	LNRRYHPALWLQ
HLA-A23:01	RYFCWYTII
HLA-A32:01	RILIRFFSL
HLA-A32:01	RLFLLFLVPLL

HLA-B41:02	AERLLYPL
HLA-A23:01	FYDNALNASF
HLA-A32:01	KALSHSSSF
HLA-B41:02	EEKRVGFSL
HLA-A32:01	RSSWDYRLYLF
HLA-A32:01	KAFSHSSTL
HLA-A32:01	RILIRFFLL
HLA-A23:01	GWSCHNGHYF
HLA-C08:02	LLDEKGPEV
HLA-A32:01	IIFVFLVETRF
HLA-B41:02	VERLEKWLQL
HLA-B41:02	SELHGFEAAVL
HLA-A32:01	KIHKSQFII
HLA-B41:02	KECGKAFSF
HLA-B41:02	LEARAYLRI
HLA-B41:02	QEGLRSVCM
HLA-B41:02	KETPESLFEL
HLA-A32:01	STLTTHKI
HLA-A32:01	HLSPLGRSF
HLA-B41:02	AERLSSRSKA
HLA-B41:02	RELLEVVEPEV
HLA-B41:02	LEHAFNNA
HLA-A32:01	RTKDISPQL
HLA-A32:01	RSSHLTKHKVI
HLA-A32:01	RGFSCLLLL
HLA-C08:02	VSSFRGNKI
HLA-B41:02	LEEGSIDSTL
HLA-B41:02	VESNSNGAF
HLA-A32:01	SQYFTSNNY
HLA-C08:02	DQDPSCPRL
HLA-A32:01	RITVQFLI
HLA-B41:02	NESSNLTTHKI
HLA-B41:02	CEECGKAF
HLA-B41:02	SEDPRFREAV
HLA-B41:02	GECGKTFRQSA
HLA-B41:02	CEECGKTF
HLA-B14:02	CQYIRSMAK
HLA-B41:02	AQLALSFSRL
HLA-B41:02	IEHKRSHTG
HLA-B41:02	GEKPYKCEEC
HLA-B41:02	KQLTLQANV
HLA-B41:02	GEKPYKCKEC
HLA-B41:02	GEKPCKCEEC
HLA-C08:02	RGDSTSSIR
HLA-A32:01	STLTTHKRI
HLA-C08:02	CSVKNQTPL
HLA-A32:01	RIHTGEKPY

HLA-C08:02	PITKVGRAL
HLA-C08:02	QAQTSGRGC
HLA-A32:01	KVIHTGEKPY
HLA-B14:02	THKRIHTGE
HLA-C08:02	ECDKATHTG
HLA-B41:02	CEECGKAFNQC
HLA-C08:02	CTPAWTAEL
HLA-B41:02	TQHKVIHTG
HLA-B14:02	KHKIIHTGEK
HLA-B14:02	YKCEECGKAFS
HLA-B14:02	SKHKIIHTGE
HLA-DRB101:0	TCQYIRSMAKRL\
HLA-DRB101:0	ITTLMHLSPLGRS
HLA-DRB101:0	LIRFFLLESWQIGK
HLA-DRB101:0	RITVQFLISLCGVS
HLA-DRB111:0	REWVIQKIHKSQI
HLA-DRB111:0	RSICMHTKKRVSS
HLA-DRB101:0	ECGKTFNHFSALF
HLA-DRB101:0	FLLFLVPLLWAPA
HLA-DRB101:0	IVNQAQLALSFSR
HLA-DRB101:0	ECGKTFRQSANLY
HLA-DRB101:0	VLEARAYLRILGEE
HLA-DRB101:0	GKAFKYSSTLTTH
HLA-DRB111:0	QFSTLKKHKIIHTG
HLA-DRB111:0	AGPITKVGRALSP
HLA-DRB101:0	GKAFIWSSTLTTH
HLA-DRB111:0	IIFVFLVETRFCHV
HLA-DQA105:0	STGLGWGSGLVA
HLA-DRB101:0	FSCLLLLTSEIDLP\
HLA-DRB111:0	GKALSHSSSFRRH
HLA-DRB101:0	LSKQLTLQANVSE
HLA-DRB111:0	GKAFNRSSHLTKH
HLA-DRB111:0	PGFKRFSCLSLRSS
HLA-DRB111:0	RSSVLSKHKIIHTG
HLA-DRB111:0	TSSIRSTGKLSKAG
HLA-DRB111:0	FSHSSTLAKHKRIH
HLA-DRB111:0	SVLVERLEKWLQL
HLA-DRB101:0	GSSFYALEEKRVG
HLA-DRB111:0	LSAVTATQKKKAE
HLA-DRB111:0	SILAKHKVIHTGEK
HLA-DRB111:0	SISSTFTKHKIIHTG
HLA-DRB101:0	PCSVKNQTPLQLS
HLA-DRB101:0	AERLLYPLPPSAD
HLA-DRB101:0	ETPESLFELNSVM
HLA-DRB111:0	RQEGLRSVCMHT
HLA-DRB101:0	VLYAVLVHAGWS
HLA-DRB111:0	SSNLTTHKRIHTG

HLA-DRB101:0	TTFYDNALNASFL
HLA-DQA105:0	PSELHGFEAAVLR
HLA-DRB101:0	EECGKAFYQSSILS
HLA-DRB101:0	AFNNARYQLGIEK
HLA-DRB101:0	LLLLTSEIDLPVKR
HLA-DRB111:0	ILTKHKIIHTGEKP
HLA-DRB111:0	SFPSSVRRHERIHS
HLA-DQA105:0	CWYTIIGWGTPT
HLA-DQA105:0	PRFREAVKAASDE
HLA-DQA105:0	SEAGVGVESNSN
HLA-DRB111:0	NRSSHLTQHKVIH
HLA-DRB111:0	SSTLNGHKRIHTG
HLA-DRB111:0	SSSLIEHKRSHTGE
HLA-DRB101:0	DISPQLNSATIIDII
HLA-DRB111:0	FTRSSSLIEHKRSH
HLA-DQA105:0	RLSRELLEVVEPE
HLA-DRB101:0	EECGKAFNQCSN
HLA-DRB111:0	LTKHKRIHTGEKP
HLA-DRB101:0	LLLTSEIDLPVKRR
HLA-DRB111:0	SIRECRRSPSSQYF
HLA-DRB101:0	PSCPRLSRELLEV
HLA-DQA105:0	CTPAWTAELDSV
HLA-DRB101:0	SRELLDEKGPEVL
HLA-DQA105:0	VQIIDDEPQAQTS
HLA-DQA105:0	DKATHTGEKPYK
HLA-A32:01	KTFSKVSTL
HLA-A23:01	FYINERGHIF
HLA-A23:01	RYFCWYTII
HLA-A32:01	RLFLLFLVPLL
HLA-A23:01	FYDNALNASF
HLA-C08:02	LLDEKGPEV
HLA-B41:02	VERLEKWLQL
HLA-C08:02	FQDSHDRCL
HLA-A32:01	KIHKSQFII
HLA-B41:02	LEARAYLRI
HLA-B41:02	HERKCGAVRV
HLA-B41:02	LERNPMSV
HLA-A32:01	HLSPLGRSF
HLA-B41:02	LDRCYSTPF
HLA-A32:01	KQNFSLKL
HLA-B41:02	RELLEVVEPEV
HLA-A32:01	RTKDISPQL
HLA-B41:02	VESNSNGAF
HLA-C08:02	WYDKKDNTCPM
HLA-A32:01	SQYFTSNNY
HLA-A32:01	KLKSGIRAVVL
HLA-A32:01	RITVQFLI

HLA-B41:02	CEECGKAF
HLA-B41:02	SEDPRFREAV
HLA-A32:01	SMFSILTKHKV
HLA-B41:02	GEKPYKCEEC
HLA-A23:01	WWWKMDQPEI
HLA-B14:02	AHACNPSTL
HLA-A32:01	KIHTGEKPY
HLA-C08:02	RGDSTSSIR
HLA-C08:02	WASQSAGITGV
HLA-C08:02	CSVKNQTPL
HLA-C08:02	PITKVGRAL
HLA-A32:01	KIIHSGEKPY
HLA-A32:01	KIIHTGEKPY
HLA-B14:02	SPPPPCCSL
HLA-B14:02	HKRGKVHSN
HLA-DRB101:0	ITTLMHLSPLGRS
HLA-DRB111:0	GFSMFSILTKHKV
HLA-DRB101:0	VQYGIQRVEAML
HLA-DRB111:0	HFSALRRHKIIHT(
HLA-DRB101:0	RITVQFLISLCGVS
HLA-DRB111:0	REWVIQKIHKSQI
HLA-DRB101:0	LPFDIMKGLTLER
HLA-DRB101:0	CCSLLELPPELLVE
HLA-DRB101:0	FLLFLVPLLWAPA
HLA-DRB101:0	VLEARAYLRILGEI
HLA-DRB111:0	LKKQNFSLKLRIYI
HLA-DRB101:0	FSKVSTLTTHKAIH
HLA-DRB111:0	AGPITKVGRALSP
HLA-DRB101:0	KSGIRAVVLPGVS
HLA-DRB111:0	SAGITGVSHRARL
HLA-DRB111:0	TSSIRSTGKLSKAG
HLA-DRB101:0	KKDLLAAVAHACI
HLA-DRB111:0	SVLVERLEKWLQI
HLA-DRB101:0	PCSVKNQTPLQLS
HLA-DRB101:0	HQFFLNTTGFSFC
HLA-DRB111:0	DDNIVHKRGKVH
HLA-DRB101:0	KDRFYINERGHIFT
HLA-DRB101:0	TTFYDNALNASFL
HLA-DRB101:0	EECGKAFNQSSTL
HLA-DQA105:0	CWYTIIGWGTPT\
HLA-DRB101:0	EWWWKMDQPE
HLA-DQA105:0	PRFREAVKAASDI
HLA-DQA105:0	SEAGVGVESNSN(
HLA-DRB111:0	THKIIHSGEKPYK(
HLA-DRB101:0	DISPQLNSATIIDI
HLA-DRB111:0	KHEIIHTGEKPYK(
HLA-DQA105:0	RLSRELLEVVEPE\

HLA-DRB111:0	TLTTHKKIHTGEK
HLA-DQA105:0	CYSTPFEYLELPDL
HLA-DRB111:0	SIRECRRSPSSQY
HLA-DQA105:0	PMFVYGGCQGN
HLA-DRB101:0	SRELLDEKGPEVL
HLA-A32:01	KILHLPTF
HLA-A32:01	KLLKNFAF
HLA-A23:01	IYLSLLLLLI
HLA-A23:01	RYFCWYTII
HLA-B41:02	HEFVGPPSL
HLA-A32:01	KSLRLKINLII
HLA-C08:02	AADVIQQGI
HLA-A23:01	TYVALMSFI
HLA-A23:01	IFAINNTKF
HLA-B41:02	HESLYNPEI
HLA-A23:01	AYSVDKLLF
HLA-B41:02	REINRKTPV
HLA-B41:02	IELRDNTSI
HLA-A32:01	KIHKSQFII
HLA-B41:02	LEARAYLRI
HLA-B41:02	SEVLGRPTSL
HLA-B41:02	SEYFLLNPYL
HLA-A32:01	KLQQENSIF
HLA-B41:02	NEDKFSLML
HLA-B41:02	QEAEVAVSA
HLA-C08:02	QADNIIRHV
HLA-A32:01	VLFGSHHAL
HLA-A32:01	KNMLTSLGASF
HLA-C08:02	KCDECGKVF
HLA-B41:02	SEGCTPLMHVV
HLA-C08:02	TTDAPVCSG
HLA-B41:02	VESNSNGAF
HLA-A32:01	KNLVGVTI
HLA-B41:02	CQGVSHQAV
HLA-B41:02	DENALCFNTVV
HLA-B41:02	PEGLLPHPAL
HLA-A32:01	NLYGTVQPHTL
HLA-A23:01	MFSPNHII
HLA-B41:02	HESNMLLKC
HLA-C08:02	RGDSTSSIR
HLA-A23:01	LPLSYPPPQF
HLA-C08:02	PITKVGRAL
HLA-A32:01	MLNRKASRNAY
HLA-B41:02	AQLREGCRARF
HLA-B41:02	KEHQQQQQ
HLA-DRB101:0	DAKILHLPAYSVD
HLA-DRB101:0	AWSLLLLSSAQLR

HLA-DRB101:0	LLKNFAFMKSYILI
HLA-DRB101:0	KNMLTSLGASFTY
HLA-DRB111:0	LRLKINLIIHQRNH
HLA-DRB101:0	LNPYLSALSSSSKD
HLA-DRB101:0	QVLVLKSKNLVGV
HLA-DRB101:0	FRSTILHESNMLLI
HLA-DRB111:0	REWVIQKIHKSQI
HLA-DRB101:0	VLILERPLLIETYVA
HLA-DRB101:0	HHALVQWKPPAI
HLA-DRB101:0	DNIIRHVLNIITKD
HLA-DRB101:0	VLEARAYLRILGEE
HLA-DRB101:0	AAVIGLTKSVAAD
HLA-DRB101:0	FPHLQGVQIYLSL
HLA-DRB101:0	CTPLMHVVSGRC
HLA-DRB101:0	DDEFIAINNTKFSY
HLA-DRB111:0	AGPITKVGRALSP
HLA-DRB101:0	AHALFYTLPEGLLI
HLA-DRB111:0	APLIRRQLSHDHE
HLA-DRB101:0	VNLYGTVQPHTLI
HLA-DRB101:0	SYPPPQFAHALLA
HLA-DRB111:0	TSSIRSTGKLSKAG
HLA-DRB101:0	DKFSLMLPGWDL
HLA-DRB111:0	IFYDLVREINRKTP
HLA-DQA105:0	QGVSHQAVAEAL
HLA-DRB101:0	NTSIRWDTAMTV
HLA-DRB101:0	DQFYSEVLGRPTS
HLA-DRB101:0	CSGLWLCQPTPR
HLA-DQA105:0	EVDMQEAEVAVS
HLA-DRB101:0	FSPNHIIVTEFILLG
HLA-DRB101:0	GKVFNWWSQLT
HLA-DRB101:0	ENSIFRNEITTLNE
HLA-DRB111:0	MLNRKASRNAYY
HLA-DRB101:0	ALCFNTVVGHNC
HLA-DQA105:0	CWYTIIGWGTPTY
HLA-DRB101:0	ESLYNPEIASCIQV
HLA-DQA105:0	SEAGVGVESNSN
HLA-DRB111:0	KLTRARDKEHQQ
HLA-A23:01	RYFCWYTII
HLA-A32:01	RLFLLFLVPLL
HLA-A23:01	FYDNALNASF
HLA-A23:01	VYWWGLSPAL
HLA-B41:02	IERMVRWRI
HLA-A23:01	VHLRKSGYVF
HLA-B41:02	VERLEKWLQL
HLA-A32:01	KIHKSQFII
HLA-B41:02	LEARAYLRI
HLA-C08:02	KADKVLQSFL

HLA-A32:01	HLSPLGRSF
HLA-C08:02	VLDQNNNAPEI
HLA-A32:01	RTKDISPQL
HLA-B41:02	VESNSNGAF
HLA-A32:01	SQYFTSNNY
HLA-B41:02	SQRSHLAA
HLA-A32:01	RITVQFLI
HLA-B41:02	SEDPRFREAV
HLA-C08:02	HSSTHPGEV
HLA-B41:02	AEIPEEPA
HLA-C08:02	ILDRRSGRQI
HLA-A32:01	VLIQPSNRRAF
HLA-A32:01	KASANDQMF
HLA-C08:02	RGDSTSSIR
HLA-C08:02	CSVKNQTPL
HLA-C08:02	TSSSSQSET
HLA-C08:02	STDSLKARG
HLA-C08:02	PITKVGRAL
HLA-B41:02	PEGTSRTV
HLA-A23:01	LYLRPPQPP
HLA-DRB101:0	ITTLMHLSPLGRS
HLA-DRB111:0	TVSLIYAVHLRKS(
HLA-DRB111:0	KKEYIERMVRWR
HLA-DRB101:0	GYKVLIQPSNRRA
HLA-DRB101:0	RITVQFLISLCGVS
HLA-DRB111:0	REWVIQKIHKSQ[
HLA-DRB101:0	FLLFLVPLLWAPA
HLA-DRB101:0	VLEARAYLRILGEE
HLA-DRB111:0	GAVNYKLKLVHSS
HLA-DRB101:0	KVLQSFLQSQVVI
HLA-DRB111:0	AGPITKVGRALSP
HLA-DRB101:0	SVYWWGLSPALD
HLA-DRB101:0	RRGLLYLRPPQPP
HLA-DRB101:0	CGKAFSQRSHLA/
HLA-DRB111:0	TSSIRSTGKLSKAG
HLA-DRB111:0	QRRSVILDRRSGR
HLA-DRB111:0	SVLVERLEKWLQI
HLA-DRB101:0	PCSVKNQTPLQLS
HLA-DRB101:0	TTFYDNALNASFL
HLA-DQA105:0	CWYTIIGWGTPT'
HLA-DRB101:0	STDSLKARGAPR(
HLA-DQA105:0	PRFREAVKAASDE
HLA-DRB101:0	LSLFVLDQNNNAI
HLA-DQA105:0	SEAGVGVESNSN(
HLA-DQA105:0	ANTVWTEASQSA
HLA-DRB101:0	DISPQLNSATIIDII
HLA-DQA105:0	TSSSSQSETSSAVS

HLA-DQA105:0	WVEGNGVLSLPE
HLA-DRB111:0	SIRECRRSPSSQYI
HLA-DQA105:0	TKESSTKASANDC
HLA-C07:02	FYYAYVFEL
HLA-B07:02	RPPFSAAKSL
HLA-A03:01	KIHTRGKPYK
HLA-B07:02	TPAERLLTPL
HLA-C07:02	YRHAPPHPANF
HLA-A03:01	IIHTGKKPYK
HLA-B07:02	SPRLECSGAI
HLA-A03:01	RIHTGEKPYK
HLA-B35:01	HSHSRTPEY
HLA-A03:01	KLAFQKDTMK
HLA-A03:01	KIHTGEKPYK
HLA-A03:01	KTKRVSSQK
HLA-A03:01	IIHTGEKPYK
HLA-A03:01	IIHTREKPYK
HLA-B07:02	TPSECQLTPL
HLA-A03:01	IIHTVEKPYK
HLA-B07:02	TPPECVLTPL
HLA-A03:01	LLFLWRSFR
HLA-C07:02	FYNFLVVL
HLA-B35:01	VPLGTGTSIF
HLA-B35:01	FVEPSLVFAY
HLA-B07:02	RPQGGNQSQ
HLA-A03:01	VIHTGEKPYK
HLA-B07:02	WPLKVLGLQEM
HLA-A03:01	KSYKCEECGK
HLA-A03:01	TIHTGEKPYK
HLA-A03:01	VSSRKQPALK
HLA-A03:26	KAFNQSSTLTK
HLA-B07:02	RPEPPDTGV
HLA-B07:02	TPRARGVPPTV
HLA-B35:01	HQIIHTGEKPY
HLA-B07:02	SPSSSSPST
HLA-A03:01	IIHTEEKPYK
HLA-B07:02	SPSELQTAGPV
HLA-C07:02	ARTECPPHL
HLA-B07:02	EPWNMKRHEM
HLA-B07:02	TPLHVTSTSSA
HLA-A03:01	RGYNGLNQCLK
HLA-A03:26	KAFNQSSHLTR
HLA-C07:02	TRLVQVVL
HLA-C07:02	CRSSRASHF
HLA-A03:26	AQAGVWWHK
HLA-A03:26	KTIHTGEK
HLA-A03:01	IVHTEEKLNK

HLA-C07:02	HHVDAHATL
HLA-B07:02	GPEYTGPCL
HLA-A03:26	STHKIIHTGEK
HLA-C07:02	WLTPVIPAL
HLA-A03:26	VVLFFLWRSFR
HLA-B07:02	LPTSASQSARI
HLA-B07:02	TPSGGTAQT
HLA-B35:01	LPSADDNIK
HLA-A03:26	KIHYESVK
HLA-A03:26	TTHKIIHTGEK
HLA-B35:01	APQESWDEGY
HLA-B07:02	HPHPPPHPP
HLA-A03:26	TVHKIIHTGEK
HLA-B07:02	KTFRHNSAL
HLA-B35:01	FGGANGSLF
HLA-B07:02	KAFKYFSSL
HLA-A03:01	KFSSLTQHK
HLA-B07:02	QPGGSLRL
HLA-A03:26	SSHKRIHTGEK
HLA-A03:26	TTHKKIHTGEK
HLA-A03:01	SQSSTLRKHK
HLA-A03:26	GVRDQPGQHGK
HLA-A03:01	FILEVSETK
HLA-A03:26	KIREQEEK
HLA-A03:01	HTGEKPYKCK
HLA-A03:01	HLTTHKIIH
HLA-B07:02	APELDEHL
HLA-B07:02	RPYKCEECGKA
HLA-C07:02	SRSGQSSRG
HLA-C07:02	IHTGEKPYN
HLA-B07:02	KPYKCKECGKA
HLA-B07:02	KPYKCEECGKV
HLA-C07:02	VRAQAKKFS
HLA-A03:26	TQHRRIHTGEK
HLA-B35:01	DVDAYSLQL
HLA-B07:02	KPYKCEDCGKA
HLA-A03:01	TLNKHKRIH
HLA-C07:02	SYHKKIHTG
HLA-B07:02	KPYKCEECGKA
HLA-C07:02	YKCEECGKTF
HLA-C07:02	YKCEECDKA
HLA-C07:02	GKKPYKCEE
HLA-C07:02	VSICGIQDE
HLA-A03:01	KKHKIIHTGEK
HLA-A03:01	STDLTTHKIIH
HLA-A03:01	IEHKKIHTGEK
HLA-A03:01	KCEECGKAFNR

HLA-B07:02	KCEECGKAFNQ
HLA-A03:01	KHKVIHTGEKP
HLA-DRB115:0	LLFLWRSFRSVRS
HLA-DRB115:0	KAFKYFSSLTTHKI
HLA-DRB115:0	VIVLVVLWFYYAY
HLA-DQA101:C	TFRHNSALVIHKA
HLA-DRB113:0	AQAIIPSWPLKVL
HLA-DQA101:C	LRVNGSCSVAQA
HLA-DRB113:0	LSRLMLSNRPPFS
HLA-DRB115:0	NLSNKAFYNFLVV
HLA-DRB113:0	NLFFLRRSLALSPF
HLA-DQA101:C	SPSILAAKLAFQKI
HLA-DQA101:C	KECGKAFMWISA
HLA-DRB115:0	QSSHLTRHKSIHT
HLA-DQA101:C	LTVRVHGFILEVSI
HLA-DRB115:0	KAFKYSSTLSSHKF
HLA-DQA101:C	QPGGSLRLSCAAS
HLA-DQA101:C	VFTTKAFVEPSLVI
HLA-DQA101:C	WIMPISVALSTFG
HLA-DQA101:C	PSELQTAGPVATI
HLA-DQA101:C	TSASQSARITGVS
HLA-DRB113:0	QSSILTKHKIIHTV
HLA-DQA101:C	LKGLEDTRLVQVV
HLA-DRB113:0	NSQLVRHQIIHTG
HLA-DQA101:C	PWLTPVIPALWEA
HLA-DRB115:0	NQSYQLTRHKIVH
HLA-DRB115:0	GKAFNKFSSLTQH
HLA-DRB113:0	KIHYESVKVNTYS
HLA-DQA101:C	SASTGHATPLHVT
HLA-DRB113:0	FHQSSILTKHKIIH
HLA-DRB115:0	KTTQSKIFQCGKY
HLA-DQA101:C	IKEKTVVSICGIQD
HLA-DQA101:C	VPLGTGTSIFSKQ
HLA-DRB113:0	KAFRQSATLNKHI
HLA-DRB113:0	WLSKLTVHKIIHT
HLA-DQA101:C	FSKASTLTAHKTIH
HLA-DRB115:0	KWSSTLSYHKKIH
HLA-DRB113:0	KHKIIHTGKKPYK
HLA-DRB113:0	TGNIAKDVDAYSL
HLA-DRB115:0	KSSTLTYHKVIHT
HLA-DQA101:C	ECGKAFNQSAHL
HLA-DQA101:C	FKRSSALSTHKIIH
HLA-DRB115:0	FSQSSTLRKHKIIH
HLA-DRB115:0	QFSTLKKHKIIHTG
HLA-DQA101:C	EGYWTLSIPPDM
HLA-DRB113:0	KHEIIHTGKKPYK
HLA-DRB113:0	NIFKQLSDLTKHK

HLA-DQA101:0	CHTKIHHVDAHA
HLA-DRB113:0	ECGKAFNRSSNLI
HLA-DRB115:0	SSNLIEHKKIHTGE
HLA-DRB113:0	QTGCRSSRASHF
HLA-DRB115:0	EECGKVFNWWS
HLA-DQA101:0	CEECGKAFNISSH
HLA-DRB113:0	NRSSHLTQHKIIH
HLA-DQA101:0	ECQLTPLPPSALP
HLA-DRB115:0	PEYYPNAGLIKNY
HLA-DRB113:0	SILTKHKVIHTGEK
HLA-DRB115:0	SSTLTKHKKIHTRE
HLA-DQA101:0	FNESSNLTTHKIIH
HLA-DRB113:0	FNQSSHLTTHKIIH
HLA-DQA101:0	CEECGKAFRQSLT
HLA-DQA101:0	KCEVCGKAFRQS
HLA-DQA101:0	KRVSSRKQPALK
HLA-DQA101:0	WNMKRHEMVA
HLA-DQA101:0	RSTDLTTHKIIHTG
HLA-DQA101:0	EECGKAFGRSRV
HLA-DRB113:0	YEKRVRAQAKKF
HLA-DQA101:0	LDEHLVERATQE
HLA-DQA101:0	GEQHGSSSGLSS
HLA-DQA101:0	NIKTPAECLLTPL
HLA-DRB115:0	LNKHKTIHTGEKP
HLA-DQA101:0	RETDFGVGVRDC
HLA-DRB113:0	HKIIHTGEKPYKC
HLA-DQA101:0	PPECVLTPLPPSA
HLA-DRB113:0	SLTKHKVIHTGEK
HLA-DRB115:0	SSNLTTHKKIHTG
HLA-DQA101:0	KCEECGKAFNQF
HLA-DRB115:0	LTRHKRIHTGEKP
HLA-DQA101:0	NCEECGKAFTRSS
HLA-DRB113:0	EHKVIHTGEKPYK
HLA-DRB115:0	VTTHKKIHTRGKP
HLA-DQA101:0	LLTPRARGVPPTV
HLA-DRB115:0	SSWDYRHAPPHP
HLA-DQA101:0	PAAPCLPPLAHH
HLA-DQA101:0	ECPPHLAVGDWS
HLA-DQA101:0	PPKTRGSHLRPEP
HLA-DQA101:0	PGMGTSTSHQPT
HLA-DRB115:0	DVEQKLGPEYTG
HLA-DQA101:0	EKPYKCEECGKVF
HLA-DQA101:0	PSSSSPSTSSPSSS
HLA-DRB113:0	EKVWRQEEKIRE
HLA-DQA101:0	RRPQGGNQSQG
HLA-C07:02	FYYAYVFEL
HLA-B35:01	TPEATQPIY

HLA-A03:01	ILSGGVFSK
HLA-A03:01	RIYIRHTLR
HLA-B35:01	CFVDDTNVY
HLA-C07:02	RYQSLNYLL
HLA-C07:02	FYNFLVVL
HLA-A03:01	GVNSLFRRK
HLA-B07:02	LPAYSVDKL
HLA-B07:02	SPSELQTAGPV
HLA-A03:26	STFGDSVK
HLA-B07:02	DPDVARNPV
HLA-B35:01	HANWLGDPE
HLA-B07:02	GPEYTGPCL
HLA-C07:02	SRIQNIIEI
HLA-C07:02	VRTEGEHFPL
HLA-C07:02	FWRGNLATV
HLA-A03:26	SSLFSTTR
HLA-B35:01	QVLLGFLF
HLA-DRB115:0	DAKILHLPAYSVD
HLA-DRB113:0	LARIYIRHTLRNFII
HLA-DRB115:0	VIVLVVLWFYYAY
HLA-DRB115:0	LATVIRYFPTQALI
HLA-DRB113:0	FNNILSNLGQVLL
HLA-DRB113:0	VECLVSRKAMMI
HLA-DRB115:0	NLSNKAFYNFLVV
HLA-DRB115:0	FMLRYLYSQKESG
HLA-DRB115:0	RRRYQSLNYLLNC
HLA-DQA101:(	PSELQTAGPVATI
HLA-DQA101:(	INTTIGSVTAQDP
HLA-DRB113:0	IICFVDDTNVYRV
HLA-DQA101:(	KIASRIQNIIEINIS
HLA-DRB115:0	HLGVNSLFRRKAD
HLA-DRB115:0	SVKLWVTFHEPW
HLA-DQA101:(	YQVEETSTYALSFI
HLA-DRB115:0	RETSLYFHSDYSQ
HLA-DRB115:0	EHFPLHSPTFSWF
HLA-DRB115:0	DVEQKLGPEYTGI
HLA-C07:02	FYYAYVFEL
HLA-B35:01	TPEATQPIY
HLA-A03:01	RIHTGERPYK
HLA-A03:01	RIHTGEKPYK
HLA-A03:01	KIHTGEKPYK
HLA-C07:02	SRVPRTVL
HLA-C07:02	TRSPPMASL
HLA-A03:01	IIHTGEKPYK
HLA-C07:02	YRSAFYAL
HLA-B35:01	LPTFSVDKLLF
HLA-C07:02	RYQSLNYLL

HLA-C07:02	FYNFLVVL
HLA-C07:02	FRQGLALSPRL
HLA-B35:01	VPLGTGTSIF
HLA-A03:01	VIHTGEKPYK
HLA-A03:01	KSYKCEECGK
HLA-A03:01	TIHTGEKPYK
HLA-B07:02	LPAYSVDKL
HLA-B07:02	TPRARGVPPTV
HLA-B35:01	HQIIHTGEKPY
HLA-B07:02	SPSELQTAGPV
HLA-A03:26	STFGDSVK
HLA-B35:01	HANWLGDPE
HLA-B07:02	APSGTLRASS
HLA-B35:01	IIHTGEKPY
HLA-C07:02	YRCEECGKAF
HLA-C07:02	SRIQNIIEI
HLA-B07:02	HPLHAGRGGSC
HLA-C07:02	VRTEGEHFPL
HLA-A03:26	TTHKIIHTGEK
HLA-A03:26	TTHKRIHTGEK
HLA-A03:26	SSLFSTTR
HLA-B35:01	YTCEECGKVF
HLA-B35:01	HACNPSTL
HLA-B35:01	YQCEECGKAF
HLA-B07:02	KPYKCEECGKV
HLA-B35:01	KCEECGKAF
HLA-A03:01	KCEECGKAFK
HLA-B35:01	CEECGKAFNWF
HLA-C04:01	KCEECGKAFSW
HLA-B07:02	KCEECGKAFNQ
HLA-DRB115:0	DAKILHLPAYSVD
HLA-DRB115:0	VIVLVVLWFYYAY
HLA-DRB115:0	IFFFFRQGLALSPF
HLA-DRB115:0	NLSNKAFYNFLVV
HLA-DRB115:0	FMLRYLYSQKESG
HLA-DRB115:0	RRRYQSLNYLLNC
HLA-DRB113:0	RASLTRSPPMASL
HLA-DQA101:0	PSELQTAGPVATI
HLA-DQA101:0	FYALEQQRVGLAY
HLA-DQA101:0	KIASRIQNIIEINIS
HLA-DRB113:0	RCQLTATSTSRVP
HLA-DQA101:0	YSCSLTAHKIIHTG
HLA-DQA101:0	VPLGTGTSIFSKQY
HLA-DRB113:0	HFSALRKHKVIHT
HLA-DRB115:0	GKAFNVSSHLTTH
HLA-DRB115:0	CGKVFSRSTLTNY
HLA-DRB115:0	SVKLWVTFHEPW

HLA-DQA101:	YQVEETSTYALSF
HLA-DQA101:	KCEECGKAFSWS
HLA-DRB115:0	CGKAFNRSSHLT
HLA-DRB113:0	HFSALRKHQIIHT
HLA-DQA101:	AAPSGTLRASSG
HLA-DRB115:0	EECGKAFNWFSY
HLA-DRB115:0	GKAFNRSSHLTT
HLA-DQA101:	GKFRPGAVAHAC
HLA-DRB113:0	YSSDVIQHRRIHT
HLA-DRB115:0	RETSLYFHSDYSQ
HLA-DQA101:	KSFSQSSTLTTHK
HLA-DQA101:	CEECGKAFRQSLT
HLA-DRB113:0	HKIIHSGEKSYKC
HLA-DRB115:0	FSTLTTHKKIHTG
HLA-DQA101:	ECGKAFNQSSNL
HLA-DRB115:0	LNKHKTIHTGEKP
HLA-DRB115:0	EHFPLHSPTFSW
HLA-DRB113:0	SLTKHKVIHTGEK
HLA-DRB115:0	EECGKAFKRSSNL
HLA-DRB113:0	SSALTNHKRIHTG
HLA-DQA101:	KCEECGKAFNQF
HLA-DRB113:0	QRNQVRKHPLH
HLA-DQA101:	LLTPRARGVPPT
HLA-C07:02	FYYAYVFEL
HLA-B07:02	LPRASRTRTL
HLA-B35:01	TPEATQPIY
HLA-C07:02	TRSPPMASL
HLA-C07:02	RYQSLNYLL
HLA-C07:02	FYNFLVVL
HLA-B35:01	VPLGTGTSIF
HLA-B07:02	SPSELQTAGPV
HLA-A03:26	STFGDSVK
HLA-B07:02	DPDVARNPV
HLA-C07:02	SRIQNIIEI
HLA-C07:02	VFVADGLTL
HLA-C07:02	VRTEGEHFPL
HLA-A03:01	CSGAISAHCK
HLA-B35:01	QVLLGFLF
HLA-DRB115:0	VIVLVVLWFYYAY
HLA-DRB113:0	FNNILSNLGQVLL
HLA-DRB115:0	NLSNKAFYNFLVV
HLA-DRB115:0	AISAHCKLRLIHIIL
HLA-DRB115:0	RRRYQSLNYLLNC
HLA-DRB113:0	RASLTRSPPMASL
HLA-DQA101:	PSELQTAGPVATI
HLA-DQA101:	INTTIGSVTAQDP
HLA-DQA101:	KIASRIQNIIEINIS

HLA-DRB115:0	GAVFVVFVADGL
HLA-DQA101:(	VPLGTGTSIFSKQ
HLA-DRB115:0	SVKLWVTFHEPW
HLA-DQA101:(	YQVEETSTYALSFI
HLA-DRB113:0	MASLRRTPSRASI
HLA-DRB115:0	EHFPLHSPTFSWI
HLA-B07:02	RPRWADHEV
HLA-B35:01	WIMHCTLVY
HLA-C07:02	FYYAYVFEL
HLA-B35:01	TPEATQPIY
HLA-A03:01	IIHTGEKPYK
HLA-C07:02	RYQSLNYLL
HLA-C07:02	FYNFLVVL
HLA-C07:02	VRWRDLGSL
HLA-C07:02	HRSRCKALL
HLA-B07:02	TPRARGVPPTV
HLA-B07:02	SPSELQTAGPV
HLA-A03:26	STFGDSVK
HLA-B07:02	DPDVARNPV
HLA-B07:02	GPEYTGPCL
HLA-C07:02	SRIQNIIEI
HLA-C07:02	VRTEGEHFPL
HLA-B35:01	CWSKCGFPF
HLA-B35:01	SPDLLDDPDNY
HLA-B35:01	QVLLGFLF
HLA-DRB115:0	VIVLVVLWFYYAY
HLA-DRB113:0	FNNILSNLGQVLL
HLA-DRB115:0	NLSNKAFYNFLVV
HLA-DRB115:0	RRRYQSLNYLLNC
HLA-DRB115:0	FIITRLVILPFWIM
HLA-DQA101:(	PSELQTAGPVATI
HLA-DQA101:(	INTTIGSVTAQDP
HLA-DQA101:(	KIASRIQNIIEINIS
HLA-DRB115:0	LSLMTLTLFSPDLI
HLA-DQA101:(	MESRSGIQAGVR
HLA-DRB115:0	SVKLWVTFHEPW
HLA-DQA101:(	YQVEETSTYALSFI
HLA-DRB113:0	DSGPSIHRSRCKA
HLA-DRB115:0	QFSTLKKHKIIHTC
HLA-DRB115:0	EHFPLHSPTFSWI
HLA-DQA101:(	MGRGLLCWSKC(
HLA-DQA101:(	LLTPRARGVPPTV
HLA-DRB115:0	KWPHSLRRPRW/
HLA-DRB115:0	DVEQKLGPEYTGI
HLA-B15:01	TSMDPIAAF
HLA-A02:05	VMATVTAL
HLA-B15:01	LSMELREF

HLA-C06:02	SRMKVLRFI
HLA-A02:05	LLAVYCMHSA
HLA-B15:01	SLTSHIPF
HLA-A02:05	LMAMENEEMSL
HLA-A02:05	ILWGRDPITGV
HLA-A26:01	YVTELIIAM
HLA-A02:05	LLAKGLDSCI
HLA-A02:05	NMVVIHSHL
HLA-A02:05	LLLGSADLGI
HLA-C06:02	GRGKGISTI
HLA-A02:05	HQSGKVQDNI
HLA-A26:01	DIYRNGGDF
HLA-A02:05	AIPRTEFPEA
HLA-DRB111:0	EEIVMATVTALRF
HLA-DRB111:0	HIPFKLVSTFKNY
HLA-DRB111:0	SYHETSRMKVLRI
HLA-DRB111:0	LGNMVVIHSHLW
HLA-DRB111:0	TELIIAMSFPSSGF
HLA-DRB111:0	LHAVRIDRICSLM
HLA-DRB111:0	LNGYIYILWGRDF
HLA-DRB107:0	DLGICLHTSSSGLI
HLA-DRB111:0	KCDLHLLAKGLDS
HLA-DRB107:0	DEKCCFWVHQS(
HLA-DRB111:0	AVYCMHSAEGRK
HLA-DRB111:0	GKGISTITGHRGK
HLA-DQA105:0	FAPDSSAFPPAIPI
HLA-DRB111:0	VDLLSMELREFFR
HLA-DQA105:0	VDVTSMDPIAAF
HLA-DRB111:0	SIGIQMPVGHIDI
HLA-C07:01	FRQTAGKVL
HLA-C07:01	FHNEAWNTL
HLA-C07:01	SRSGGDRSP
HLA-A11:01	LAARARLDSK
HLA-C07:01	DVFQNLSHL
HLA-C07:01	YAGYIPPSQ
HLA-DRB110:0	TLPFLRQTAGKVL
HLA-DRB110:0	GSYQFSMVAGSR
HLA-DRB115:0	SESFCLAARARLD
HLA-DRB110:0	VFQNLSHLPTFSS
HLA-DRB115:0	CSFVLAYAGYIPP
HLA-DRB110:0	WNTLLRTVYSVIN
HLA-A02:01	FMTMIVSLAV
HLA-B07:02	KPRCWEASV
HLA-A02:01	VMFCLECAL
HLA-A02:01	FLQAMDSFL
HLA-A02:01	LMHQGLPTL
HLA-A02:01	FLSLALSPRL

HLA-B07:02	KPGAVAEEVL
HLA-A02:01	ALHLMPPPFL
HLA-C07:01	TRIQDLNHI
HLA-C07:01	IRLTTRPKV
HLA-B07:02	RPRLVPDRT
HLA-C07:01	MRSCGRGGF
HLA-A02:01	GMFEGCTTDCP
HLA-A02:01	KYSNCAWEV
HLA-C07:01	GHENAIRDY
HLA-A02:01	KLTKTQGNV
HLA-B07:02	KPVGKYHIQA
HLA-A01:01	STQGHASTSSY
HLA-B07:02	DPGKASKAAWI
HLA-A02:01	SQISSWEKL
HLA-C07:01	ERNQKTDGS
HLA-C07:01	FNWNNQRCK
HLA-A02:01	SGYGVVGLQSI
HLA-DRB113:0	ASKAAWIRKIKGL
HLA-DRB115:0	CLVAFFVLQRKPR
HLA-DRB115:0	QELFLSLALSPRLE
HLA-DRB113:0	LSMVVQENENV
HLA-DRB115:0	QQALHLMPPPFL
HLA-DQA101:0	NCVELQASAAFN
HLA-DRB115:0	PKMYFMTMIVSL
HLA-DRB115:0	EFIFLQAMDSFLR
HLA-DQA101:0	TTEVGLAFARLEE
HLA-DQA101:0	NCAWEVVRMEI
HLA-DRB113:0	NVTIQCDSGYGV
HLA-DQA101:0	QIENLQATLGSQI
HLA-DQA101:0	TKTQGNVFATDA
HLA-DRB115:0	DKELLAYAKPGA
HLA-DRB115:0	CGRGFQFVSSSLP
HLA-DRB115:0	DCPSLLPILEPKYA
HLA-DRB113:0	MAAIRLTTRPKVP
HLA-DRB115:0	KEQLMHQGLPTL
HLA-DRB113:0	KTRIQDLNHIFPL
HLA-DQA101:0	KYHIQACTTTPC
HLA-DQA101:0	YINSTGSTQGHA
HLA-DRB115:0	AISAKIRKKGKIER
HLA-DQA101:0	ERGGESTRPRLVP
HLA-A02:01	GMMQFIDIL
HLA-C07:01	ARNATCTHYM
HLA-C07:01	DREHHTTIV
HLA-C07:01	IHDQSLILL
HLA-A24:02	VYCCSASTRL
HLA-C07:01	SYEDCASRI
HLA-C07:01	SRALPATPHL

HLA-A02:01	KMISNCQMQT
HLA-B08:01	NANVKRCL
HLA-C07:01	TRAEAAATA
HLA-C07:01	GREAKHCDP
HLA-C07:01	PAEEAPSEV
HLA-B08:01	QPPCHWLRQL
HLA-C07:01	QSAVPLDGE
HLA-DQA105:0	YTTRAEAAATAA
HLA-DRB113:0	YRFDLNRSLGIAV
HLA-DRB113:0	PCHWLRQLSRRG
HLA-DRB113:0	MQFIDILQNLKRS
HLA-DRB113:0	REHHTTIVATRHY
HLA-DQA105:0	SRNGSTSRALPAT
HLA-DRB113:0	TSWTFSWVPGM
HLA-DRB113:0	HYMQLVWATSS
HLA-DQA105:0	DGETHAGEDVAV
HLA-DQA105:0	LKPVNASNANVK
HLA-DRB113:0	ASRIMEDSAIKCE
HLA-DRB113:0	HDQSLILLNNVVL
HLA-DQA105:0	SEVQPPPAEEAP
HLA-DQA105:0	ENGDEAGEGREA
HLA-A03:01	RLNPGLRRK
HLA-A03:01	RVRPMNHREK
HLA-A03:01	IAMSTLVWTK
HLA-A30:02	SNYTISPSAY
HLA-A30:02	TTPLPVTDTY
HLA-A03:01	WLDDISCSGK
HLA-A30:02	HASPSPSPVGY
HLA-C07:01	LRICTDLFRLV
HLA-A30:02	KSLPCSAST
HLA-B44:03	QENVDIATVH
HLA-B18:01	HQLGNTAHPVF
HLA-DQA101:0	FAMFGTGIAMST
HLA-DRB110:0	RRQFLRICTDLFR
HLA-DRB110:0	LSNYTISPSAYFH
HLA-DRB110:0	LMQLYSARQRRR
HLA-DQA101:0	IQENVDIATVHQ
HLA-DQA101:0	QLGNTAHPVFPA
HLA-DQA101:0	MASVKVAVRVR
HLA-DRB110:0	PASYHPTPSPMA
HLA-DQA101:0	YSASTGHATPLP
HLA-DQA101:0	EESTGPIWLDDIS
HLA-DQA101:0	QQETNKSLPCSA

Transcript Name
NM_001173984,NM_013263,NM_001173984,NM_013263
NM_000721,NM_001205293,NM_001205294,NM_000721,NM_001205293,NM_001205294
NM_001309434,NM_003134,NM_001309434,NM_003134
NM_001289000,NM_001289001,NM_153609,NM_001289000,NM_001289001,NM_153609
NM_001127892,NM_002968,NM_001127892,NM_002968
NM_001145444,NM_001145445,NM_020706,NM_001145444,NM_001145445,NM_020706
NM_003086,NM_003086
NM_001309434,NM_003134
NM_000721,NM_001205293,NM_001205294
NM_001173984,NM_013263
NM_001289000,NM_001289001,NM_153609
NM_003086
NM_001127892,NM_002968
NM_001145444,NM_001145445,NM_020706
NM_001173984,NM_013263,NM_001173984,NM_013263
NM_000721,NM_001205293,NM_001205294,NM_000721,NM_001205293,NM_001205294
NM_001309434,NM_003134,NM_001309434,NM_003134
NM_001289000,NM_001289001,NM_153609,NM_001289000,NM_001289001,NM_153609
NM_001127892,NM_002968,NM_001127892,NM_002968
NM_001145444,NM_001145445,NM_020706,NM_001145444,NM_001145445,NM_020706
NM_001309434,NM_003134
NM_000721,NM_001205293,NM_001205294
NM_001173984,NM_013263
NM_001289000,NM_001289001,NM_153609
NM_001127892,NM_002968
NM_001145444,NM_001145445,NM_020706
NM_177998,NM_177998
NM_170606,NM_170606
NM_000930,NM_001319189,NM_033011,NM_000930,NM_001319189,NM_033011
NM_175918,NM_175918
NM_001079518,NM_001267797,NM_014815,NM_001079518,NM_001267797,NM_014815
NM_001290224,NM_199259,NM_199260,NM_199261,NM_001290224,NM_199259,NM_199260,NM_1992[illegible]
NM_003037,NM_003037
NM_001024847,NM_003242,NM_001024847,NM_003242
NM_015409,NM_015409,NM_015409,NM_015409
NM_001159531,NM_020836,NM_001159531,NM_020836
NM_001262,NM_078626,NM_001262,NM_078626
NM_001291415,NM_001291416,NM_001291417,NM_001291418,NM_001291421,NM_021140,NM_001291[illegible]
NM_001282484,NM_001282484
NM_001143819,NM_001301214,NM_017901,NM_001143819,NM_001301214,NM_017901
NM_003327,NM_003327
NM_005760,NM_005760
NM_001278055,NM_014363,NM_001278055,NM_014363
NM_001173984,NM_013263,NM_001173984,NM_013263
NM_000481,NM_001164710,NM_001164712,NM_000481,NM_001164710,NM_001164712
NM_001271,NM_001271

NM_032135,NM_032135
NM_182832,NM_182832
NM_001257190,NM_172020,NM_001257190,NM_172020
NM_005157,NM_007313,NM_005157,NM_007313
NM_174924,NM_174924
NM_001152,NM_001152
NM_032727,NM_032727
NM_033060,NM_033060
NM_001256876,NM_001256877,NM_015158,NM_153186,NM_001256876,NM_001256877,NM_015158,NM
NM_014698,NM_014698
NM_001098407,NM_001127212,NM_001472,NM_012196,NM_001098407,NM_001127212,NM_001472,NM
NM_014272,NM_014272
NM_001293089,NM_001293089
NM_018975,NM_018975
NM_000332,NM_001128164,NM_000332,NM_001128164
NM_152672,NM_152672
NM_152243,NM_152243
NM_033188,NM_033188
NM_001077262,NM_145345,NM_183008,NM_001077262,NM_145345,NM_183008
NM_001240,NM_001240
NM_001001480,NM_001001480
NM_001080396,NM_001080396
NM_001282959,NM_001282960,NM_018237,NM_001282959,NM_001282960,NM_018237
NM_001297590,NM_012099,NM_001297590,NM_012099
NM_001288715,NM_001288716,NM_001288717,NM_001332,NM_001288715,NM_001288716,NM_00128[illegible]
NM_005760
NM_005157,NM_007313
NM_001278055,NM_014363
NM_175918
NM_177998
NM_001257190,NM_172020
NM_170606
NM_003037
NM_001024847,NM_003242
NM_000930,NM_001319189,NM_033011
NM_015409,NM_015409
NM_001098407,NM_001127212,NM_001472,NM_012196
NM_001173984,NM_013263
NM_000481,NM_001164710,NM_001164712
NM_001159531,NM_020836
NM_001143819,NM_001301214,NM_017901
NM_001079518,NM_001267797,NM_014815
NM_182832
NM_001293089
NM_003327
NM_174924
NM_001291415,NM_001291416,NM_001291417,NM_001291418,NM_001291421,NM_021140

NM_005392
NM_152243
NM_001152
NM_001290224,NM_199259,NM_199260,NM_199261
NM_001282484
NM_001256876,NM_001256877,NM_015158,NM_153186
NM_001297590,NM_012099
NM_001262,NM_078626
NM_014698
NM_032135
NM_001288715,NM_001288716,NM_001288717,NM_001332
NM_152672
NM_033060
NM_032727
NM_014272
NM_001001480
NM_001301043,NM_001301044,NM_014333
NM_001271
NM_001240
NM_018975
NM_033188
NM_001077262,NM_145345,NM_183008
NM_001243799,NM_183422
NM_001080396
NM_001282959,NM_001282960,NM_018237
NM_000332,NM_001128164
NM_001160,NM_013229,NM_181861,NM_181868,NM_001160,NM_013229,NM_181861,NM_181868
NM_000930,NM_001319189,NM_033011,NM_000930,NM_001319189,NM_033011
NM_001301031,NM_001301032,NM_001301033,NM_004727,NM_001301031,NM_001301032,NM_001301
NM_030979,NM_030979
NM_198944,NM_198944
NM_004980,NM_172198,NM_004980,NM_172198
NM_001262,NM_078626,NM_001262,NM_078626
NM_015719,NM_015719
NM_001258411,NM_001258412,NM_001258413,NM_001258414,NM_052944,NM_001258411,NM_00125[illegible]
NM_004742,NM_015520,NM_004742,NM_015520
NM_001204087,NM_002249,NM_170782,NM_001204087,NM_002249,NM_170782
NM_001291415,NM_001291416,NM_001291417,NM_001291418,NM_001291421,NM_021140,NM_001291
NM_001282484,NM_001282484
NM_001114121,NM_001114122,NM_001244846,NM_001274,NM_001114121,NM_001114122,NM_00124[illegible]
NM_001080850,NM_001080850
NM_001173984,NM_013263,NM_001173984,NM_013263
NM_139173,NM_139173
NM_018727,NM_080704,NM_080705,NM_080706,NM_018727,NM_080704,NM_080705,NM_080706
NM_000466,NM_001282677,NM_001282678,NM_000466,NM_001282677,NM_001282678
NM_032135,NM_032135
NM_006118,NM_006118

NM_001168221,NM_152525,NM_001168221,NM_152525
NM_182614,NM_182614
NM_001319179,NM_001319180,NM_018284,NM_001319179,NM_001319180,NM_018284
NM_018365,NM_018365
NM_001152,NM_001152
NM_001184985,NM_014823,NM_018979,NM_213655,NM_001184985,NM_014823,NM_018979,NM_2136[illegible]
NM_015172,NM_015172
NM_001118887,NM_001118888,NM_001147,NM_001118887,NM_001118888,NM_001147
NM_033060,NM_033060
NM_001276270,NM_001276271,NM_001276272,NM_003925,NM_001276270,NM_001276271,NM_00127[illegible]
NM_001098407,NM_001127212,NM_001472,NM_012196,NM_001098407,NM_001127212,NM_001472,N[illegible]
NM_002653,NM_002653
NM_001013843,NM_024755,NM_001013843,NM_024755
NM_001297644,NM_001297645,NM_001318000,NM_005869,NM_001297644,NM_001297645,NM_00131[illegible]
NM_016107,NM_016107
NM_001134937,NM_001134938,NM_030917,NM_001134937,NM_001134938,NM_030917
NM_152243,NM_152243
NM_033188,NM_033188
NM_001077262,NM_145345,NM_183008,NM_001077262,NM_145345,NM_183008
NM_001240,NM_001240
NM_018660,NM_018660
NM_015576,NM_015576
NM_001288715,NM_001288716,NM_001288717,NM_001332,NM_001288715,NM_001288716,NM_00128[illegible]
NM_018660
NM_000466,NM_001282677,NM_001282678
NM_015719
NM_004980,NM_172198
NM_182614
NM_001160,NM_013229,NM_181861,NM_181868
NM_030979
NM_001168221,NM_152525
NM_001297644,NM_001297645,NM_001318000,NM_005869
NM_000930,NM_001319189,NM_033011
NM_001098407,NM_001127212,NM_001472,NM_012196
NM_001319179,NM_001319180,NM_018284
NM_001173984,NM_013263
NM_004742,NM_015520
NM_139173
NM_001291415,NM_001291416,NM_001291417,NM_001291418,NM_001291421,NM_021140
NM_152243
NM_001204087,NM_002249,NM_170782
NM_001258411,NM_001258412,NM_001258413,NM_001258414,NM_052944
NM_198944
NM_001184985,NM_014823,NM_018979,NM_213655
NM_001152
NM_001282484
NM_016107

NM_018365
NM_001301031,NM_001301032,NM_001301033,NM_004727
NM_001114121,NM_001114122,NM_001244846,NM_001274
NM_015576
NM_001262,NM_078626
NM_001276270,NM_001276271,NM_001276272,NM_003925
NM_018727,NM_080704,NM_080705,NM_080706
NM_001013843,NM_024755
NM_032135
NM_001288715,NM_001288716,NM_001288717,NM_001332
NM_033060
NM_001118887,NM_001118888,NM_001147
NM_006118
NM_001240
NM_001080850
NM_002653
NM_033188
NM_001077262,NM_145345,NM_183008
NM_015172
NM_001134937,NM_001134938,NM_030917
NM_000930,NM_001319189,NM_033011,NM_000930,NM_001319189,NM_033011
NM_001262,NM_078626,NM_001262,NM_078626
NM_001291415,NM_001291416,NM_001291417,NM_001291418,NM_001291421,NM_021140,NM_001291
NM_000930,NM_001319189,NM_033011
NM_001291415,NM_001291416,NM_001291417,NM_001291418,NM_001291421,NM_021140
NM_001262,NM_078626
NM_000268,NM_016418,NM_181825,NM_181828,NM_181829,NM_181830,NM_181831,NM_181832,NM_
NM_005157,NM_007313,NM_005157,NM_007313
NM_005157,NM_007313
NM_000268,NM_016418,NM_181825,NM_181828,NM_181829,NM_181830,NM_181831,NM_181832
NM_001290224,NM_199259,NM_199260,NM_199261,NM_001290224,NM_199259,NM_199260,NM_1992
NM_175918,NM_175918,NM_175918,NM_175918,NM_175918,NM_175918
NM_139173,NM_139173
NM_001173984,NM_013263,NM_001173984,NM_013263
NM_001288607,NM_001288608,NM_019032,NM_025008,NM_001288607,NM_001288608,NM_019032,NM
NM_001318502,NM_019892,NM_001318502,NM_019892
NM_001258210,NM_001318477,NM_001318478,NM_001318479,NM_015516,NM_001258210,NM_00131
NM_001286990,NM_007001,NM_001286990,NM_007001
NM_001098479,NM_001098479
NM_002830,NM_002830
NM_173493,NM_173493
NM_001257190,NM_172020,NM_001257190,NM_172020
NM_001152,NM_001152
NM_002515,NM_006489,NM_006491,NM_002515,NM_006489,NM_006491
NM_001632,NM_001632
NM_000721,NM_001205293,NM_001205294,NM_000721,NM_001205293,NM_001205294
NM_001105659,NM_001322315,NM_001105659,NM_001322315

NM_033060,NM_033060
NM_001197295,NM_001197296,NM_001393,NM_001197295,NM_001197296,NM_001393
NM_001143819,NM_001301214,NM_017901,NM_001143819,NM_001301214,NM_017901
NM_004628,NM_004628
NM_001002924,NM_001284,NM_001318091,NM_001318093,NM_001318094,NM_001002924,NM_001284
NM_001017395,NM_001017395,NM_001128224,NM_001128224,NM_001017395,NM_001017395,NM_001
NM_001277325,NM_001277325
NM_001282484,NM_001282484
NM_001267843,NM_004898,NM_001267843,NM_004898
NM_001127708,NM_001127708,NM_001127709,NM_001127709,NM_001127710,NM_001127710,NM_001
NM_001329226,NM_014045,NM_001329226,NM_014045
NM_001145966,NM_002417,NM_001145966,NM_002417
NM_014698,NM_014698
NM_022093,NM_022093
NM_020154,NM_020154
NM_015063,NM_015063
NM_007113,NM_007113
NM_001288715,NM_001288716,NM_001288717,NM_001332,NM_001288715,NM_001288716,NM_00128[illegible]
NM_001142524,NM_001142525,NM_001142526,NM_001142527,NM_001142528,NM_001142529,NM_001
NM_006063,NM_006063
NM_016107,NM_016107
NM_005401,NM_005401
NM_006118,NM_006118
NM_152672,NM_152672
NM_030785,NM_030785
NM_001269040,NM_001269041,NM_001269042,NM_001269043,NM_001269044,NM_001269045,NM_001
NM_001145444,NM_001145445,NM_020706,NM_001145444,NM_001145445,NM_020706
NM_001177465,NM_001177466,NM_005491,NM_001177465,NM_001177466,NM_005491
NM_173493
NM_001017395,NM_001017395,NM_001128224,NM_001128224
NM_001257190,NM_172020
NM_000721,NM_001205293,NM_001205294
NM_001288607,NM_001288608,NM_019032,NM_025008
NM_001329226,NM_014045
NM_001258210,NM_001318477,NM_001318478,NM_001318479,NM_015516
NM_001286990,NM_007001
NM_175918,NM_175918,NM_175918
NM_002830
NM_001318502,NM_019892
NM_002515,NM_006489,NM_006491
NM_001105659,NM_001322315
NM_001098479
NM_001002924,NM_001284,NM_001318091,NM_001318093,NM_001318094
NM_139173
NM_001143819,NM_001301214,NM_017901
NM_001152
NM_001282484

NM_020154
NM_001632
NM_001173984,NM_013263
NM_006063
NM_001288715,NM_001288716,NM_001288717,NM_001332
NM_014698
NM_033060
NM_015063
NM_001290224,NM_199259,NM_199260,NM_199261
NM_022093
NM_001127708,NM_001127708,NM_001127709,NM_001127709,NM_001127710,NM_001127710,NM_001
NM_001277325
NM_001145966,NM_002417
NM_016107
NM_001269040,NM_001269041,NM_001269042,NM_001269043,NM_001269044,NM_001269045,NM_001
NM_007113
NM_005401
NM_152672
NM_001267843,NM_004898
NM_006118
NM_004628
NM_001142524,NM_001142525,NM_001142526,NM_001142527,NM_001142528,NM_001142529,NM_001
NM_001197295,NM_001197296,NM_001393
NM_001177465,NM_001177466,NM_005491
NM_001145444,NM_001145445,NM_020706
NM_030785
NM_001301228,NM_001301229,NM_001301231,NM_005342
NM_000718,NM_001243812,NM_000718,NM_001243812
NM_175918,NM_175918,NM_175918,NM_175918
NM_198525,NM_198525
NM_139173,NM_139173
NM_001173984,NM_013263,NM_001173984,NM_013263
NM_001207019,NM_001220500,NM_002002,NM_001207019,NM_001220500,NM_002002
NM_032603,NM_032603
NM_001098407,NM_001127212,NM_001472,NM_012196,NM_001098407,NM_001127212,NM_001472,NM
NM_001184985,NM_014823,NM_018979,NM_213655,NM_001184985,NM_014823,NM_018979,NM_2136
NM_015560,NM_130831,NM_130832,NM_130833,NM_130834,NM_130835,NM_130836,NM_130837,NM_
NM_001098479,NM_001098479
NM_173493,NM_173493
NM_001031855,NM_001289109,NM_024778,NM_001031855,NM_001289109,NM_024778
NM_001152,NM_001152
NM_001105659,NM_001322315,NM_001105659,NM_001322315
NM_033060,NM_033060
NM_001002924,NM_001284,NM_001318091,NM_001318093,NM_001318094,NM_001002924,NM_001284
NM_001145079,NM_020751,NM_001145079,NM_020751
NM_001282484,NM_001282484
NM_007126,NM_007126

NM_001085377,NM_001085377
NM_001278651,NM_001317930,NM_002883,NM_001278651,NM_001317930,NM_002883
NM_001145966,NM_002417,NM_001145966,NM_002417
NM_022093,NM_022093
NM_001288715,NM_001288716,NM_001288717,NM_001332,NM_001288715,NM_001288716,NM_00128[illegible]
NM_006063,NM_006063
NM_001127708,NM_001127709,NM_001127710,NM_001303232,NM_005807,NM_001127708,NM_00112[illegible]
NM_001288973,NM_001288974,NM_001288975,NM_003474,NM_021641,NM_001288973,NM_00128897[illegible]
NM_001145444,NM_001145445,NM_020706,NM_001145444,NM_001145445,NM_020706
NM_173493
NM_001031855,NM_001289109,NM_024778
NM_000718,NM_001243812
NM_001145079,NM_020751
NM_175918,NM_175918
NM_198525
NM_015560,NM_130831,NM_130832,NM_130833,NM_130834,NM_130835,NM_130836,NM_130837
NM_001098407,NM_001127212,NM_001472,NM_012196
NM_001098407,NM_001127212,NM_001472,NM_012196
NM_001105659,NM_001322315
NM_001085377
NM_001098479
NM_001002924,NM_001284,NM_001318091,NM_001318093,NM_001318094
NM_032603
NM_139173
NM_001152
NM_001282484
NM_001173984,NM_013263
NM_006063
NM_001207019,NM_001220500,NM_002002
NM_001288715,NM_001288716,NM_001288717,NM_001332
NM_033060
NM_022093
NM_001278651,NM_001317930,NM_002883
NM_007126
NM_001184985,NM_014823,NM_018979,NM_213655
NM_001127708,NM_001127709,NM_001127710,NM_001303232,NM_005807
NM_001145966,NM_002417
NM_001288973,NM_001288974,NM_001288975,NM_003474,NM_021641
NM_001145444,NM_001145445,NM_020706
NM_000718,NM_001243812,NM_000718,NM_001243812
NM_001007257,NM_012231,NM_015866,NM_001007257,NM_012231,NM_015866
NM_175918,NM_175918,NM_175918,NM_175918,NM_175918,NM_175918
NM_024501,NM_024501
NM_198944,NM_198944
NM_139173,NM_139173
NM_001173984,NM_013263,NM_001173984,NM_013263
NM_005157,NM_007313,NM_005157,NM_007313

NM_001098407,NM_001127212,NM_001472,NM_012196,NM_001098407,NM_001127212,NM_001472,NM
NM_007214,NM_007214
NM_015560,NM_130831,NM_130832,NM_130833,NM_130834,NM_130835,NM_130836,NM_130837,NM_
NM_000260,NM_001127180,NM_000260,NM_001127180
NM_001031855,NM_001289109,NM_024778,NM_001031855,NM_001289109,NM_024778
NM_001282674,NM_001282674
NM_001152,NM_001152
NM_018353,NM_018353
NM_033060,NM_033060
NM_001145079,NM_020751,NM_001145079,NM_020751
NM_001282484,NM_001282484
NM_001085377,NM_001085377
NM_033188,NM_033188
NM_005886,NM_005886
NM_022093,NM_022093
NM_182832,NM_182832
NM_001288715,NM_001288716,NM_001288717,NM_001332,NM_001288715,NM_001288716,NM_00128
NM_006063,NM_006063
NM_001127708,NM_001127709,NM_001127710,NM_001303232,NM_005807,NM_001127708,NM_00112
NM_001164766,NM_006885,NM_001164766,NM_006885
NM_001001480,NM_001001480
NM_021963,NM_021963
NM_005157,NM_007313
NM_024501
NM_000260,NM_001127180
NM_001031855,NM_001289109,NM_024778
NM_001007257,NM_012231,NM_015866
NM_000718,NM_001243812
NM_001145079,NM_020751
NM_175918,NM_175918,NM_175918
NM_005886
NM_015560,NM_130831,NM_130832,NM_130833,NM_130834,NM_130835,NM_130836,NM_130837
NM_001098407,NM_001127212,NM_001472,NM_012196
NM_001098407,NM_001127212,NM_001472,NM_012196
NM_001085377
NM_198944
NM_139173
NM_007214
NM_182832
NM_001152
NM_001282484
NM_001173984,NM_013263
NM_001282674
NM_001001480
NM_006063
NM_001288715,NM_001288716,NM_001288717,NM_001332
NM_033060

NM_022093
NM_001127708,NM_001127709,NM_001127710,NM_001303232,NM_005807
NM_018353
NM_021963
NM_001177465,NM_001177466,NM_005491
NM_033188
NM_001164766,NM_006885
NM_001127891,NM_001302508,NM_001302509,NM_001302510,NM_004530,NM_001127891,NM_00130[illegible]
NM_002205,NM_002205
NM_000132,NM_000132
NM_015560,NM_130831,NM_130832,NM_130833,NM_130834,NM_130835,NM_130836,NM_130837,NM_[illegible]
NM_020730,NM_020730
NM_001145079,NM_020751,NM_001145079,NM_020751
NM_004629,NM_004629
NM_001031855,NM_001289109,NM_024778,NM_001031855,NM_001289109,NM_024778
NM_199242,NM_199242
NM_003845,NM_003845
NM_022093,NM_022093
NM_001256721,NM_006430,NM_001256721,NM_006430
NM_015054,NM_015054
NM_006063,NM_006063
NM_001127891,NM_001302508,NM_001302509,NM_001302510,NM_004530
NM_001031855,NM_001289109,NM_024778
NM_001145079,NM_020751
NM_015560,NM_130831,NM_130832,NM_130833,NM_130834,NM_130835,NM_130836,NM_130837
NM_199242
NM_003845
NM_002205
NM_020730
NM_000132
NM_006063
NM_001256721,NM_006430
NM_001256721,NM_006430
NM_004629
NM_022093
NM_015054
NM_001285439,NM_001285440,NM_152756,NM_001285439,NM_001285440,NM_152756
NM_001079846,NM_004380,NM_001079846,NM_004380
NM_001110556,NM_001456,NM_001110556,NM_001456
NM_002001,NM_002001
NM_001318787,NM_001318789,NM_001318790,NM_001318791,NM_001318793,NM_001318795,NM_001[illegible]
NM_000888,NM_001282353,NM_001282354,NM_001282355,NM_001282388,NM_001282389,NM_00128[illegible]
NM_199242,NM_199242
NM_001110556,NM_001456
NM_001318787,NM_001318789,NM_001318790,NM_001318791,NM_001318793,NM_001318795,NM_001[illegible]
NM_199242
NM_001285439,NM_001285440,NM_152756

NM_001079846,NM_004380
NM_002001
NM_000888,NM_001282353,NM_001282354,NM_001282355,NM_001282388,NM_001282389,NM_001282
NM_198471,NM_198471
NM_198557,NM_198557
NM_004645,NM_004645
NM_198557
NM_198471
NM_004645
NM_014244,NM_021599,NM_014244,NM_021599
NM_001004128,NM_002826,NM_001004128,NM_002826
NM_000527,NM_001195798,NM_001195799,NM_001195800,NM_001195803,NM_000527,NM_001195798
NM_030651,NM_030651
NM_152390,NM_152390
NM_001014797,NM_001161352,NM_001161353,NM_001271518,NM_001271519,NM_001322829,NM_001
NM_207371,NM_207371
NM_000966,NM_001042728,NM_001243730,NM_001243731,NM_001243732,NM_000966,NM_001042728
NM_000966,NM_001042728,NM_001243730,NM_001243731,NM_001243732
NM_000527,NM_001195798,NM_001195799,NM_001195800,NM_001195803
NM_001004128,NM_002826
NM_152390
NM_030651
NM_014244,NM_021599
NM_001014797,NM_001161352,NM_001161353,NM_001271518,NM_001271519,NM_001322829,NM_001
NM_207371
NM_002356,NM_002356
NM_001164380,NM_001164381,NM_001164382,NM_001164383,NM_001164384,NM_001164385,NM_014
NM_198557,NM_198557
NM_175918,NM_175918,NM_175918,NM_175918
NM_001173984,NM_013263,NM_001173984,NM_013263
NM_198944,NM_198944
NM_001136112,NM_004347,NM_001136112,NM_004347
NM_001290224,NM_199259,NM_199260,NM_199261,NM_001290224,NM_199259,NM_199260,NM_19926
NM_001293089,NM_001293089
NM_001257190,NM_172020,NM_001257190,NM_172020
NM_001013843,NM_024755,NM_001013843,NM_024755
NM_002568,NM_002568
NM_001126057,NM_001126058,NM_001190348,NM_001190349,NM_033317,NM_001126057,NM_001126
NM_033060,NM_033060
NM_033188,NM_033188
NM_006121,NM_006121
NM_032135,NM_032135
NM_033187,NM_033187
NM_001001480,NM_001001480
NM_001164380,NM_001164381,NM_001164382,NM_001164383,NM_001164384,NM_001164385,NM_014
NM_198557
NM_001136112,NM_004347

NM_175918,NM_175918
NM_001257190,NM_172020
NM_001173984,NM_013263
NM_002568
NM_198944
NM_001290224,NM_199259,NM_199260,NM_199261
NM_001301043,NM_001301044,NM_014333
NM_002356
NM_001114091,NM_001256,NM_001293089,NM_001293091
NM_032135
NM_033060
NM_006121
NM_033187
NM_001013843,NM_024755
NM_001126057,NM_001126058,NM_001190348,NM_001190349,NM_033317
NM_033188
NM_001001480
NM_004638,NM_080686,NM_004638,NM_080686
NM_001320715,NM_003921,NM_001320715,NM_003921
NM_001007257,NM_012231,NM_015866,NM_001007257,NM_012231,NM_015866
NM_001098407,NM_001127212,NM_001472,NM_012196,NM_001098407,NM_001127212,NM_001472,NM
NM_001130960,NM_014996,NM_001130960,NM_014996
NM_001271733,NM_001271733
NM_014949,NM_014949
NM_001278579,NM_001278580,NM_001616,NM_001278579,NM_001278580,NM_001616
NM_001136112,NM_004347,NM_001136112,NM_004347
NM_001173984,NM_013263,NM_001173984,NM_013263
NM_014696,NM_014696
NM_001293089,NM_001293089
NM_001204087,NM_002249,NM_170782,NM_001204087,NM_002249,NM_170782
NM_001290224,NM_199259,NM_199260,NM_199261,NM_001290224,NM_199259,NM_199260,NM_1992
NM_002568,NM_002568
NM_001271,NM_001271
NM_001240,NM_001240
NM_001282484,NM_001282484
NM_001257190,NM_172020,NM_001257190,NM_172020
NM_001105659,NM_001322315,NM_001105659,NM_001322315
NM_001003407,NM_001003408,NM_001322882,NM_001322883,NM_001322884,NM_001322885,NM_001
NM_001013843,NM_024755,NM_001013843,NM_024755
NM_033060,NM_033060
NM_006678,NM_006678
NM_006121,NM_006121
NM_001126057,NM_001126058,NM_001190348,NM_001190349,NM_033317,NM_001126057,NM_00112
NM_033188,NM_033188
NM_152672,NM_152672
NM_032135,NM_032135
NM_033187,NM_033187

NM_004645,NM_004645
NM_001080395,NM_004920,NM_001080395,NM_004920
NM_178354,NM_178354
NM_001039649,NM_001039650,NM_001142684,NM_001039649,NM_001039650,NM_001142684
NM_005401,NM_005401
NM_020815,NM_032961,NM_020815,NM_032961
NM_030785,NM_030785
NM_018975,NM_018975
NM_001136112,NM_004347
NM_014696
NM_001257190,NM_172020
NM_004638,NM_080686
NM_001173984,NM_013263
NM_002568
NM_001320715,NM_003921
NM_001007257,NM_012231,NM_015866
NM_001204087,NM_002249,NM_170782
NM_001290224,NM_199259,NM_199260,NM_199261
NM_001003407,NM_001003408,NM_001322882,NM_001322883,NM_001322884,NM_001322885,NM_001
NM_001080395,NM_004920
NM_006678
NM_001098407,NM_001127212,NM_001472,NM_012196
NM_014949
NM_001114091,NM_001256,NM_001293089,NM_001293091
NM_001271733
NM_001130960,NM_014996
NM_001105659,NM_001322315
NM_001039649,NM_001039650,NM_001142684
NM_001282484
NM_001278579,NM_001278580,NM_001616
NM_005401
NM_032135
NM_033060
NM_001240
NM_178354
NM_004645
NM_020815,NM_032961
NM_152672
NM_006121
NM_033187
NM_001013843,NM_024755
NM_001126057,NM_001126058,NM_001190348,NM_001190349,NM_033317
NM_033188
NM_018975
NM_001243799,NM_183422
NM_001271
NM_030785

NM_001010870,NM_001168359,NM_001010870,NM_001168359
NM_001315529,NM_006031,NM_001315529,NM_006031
NM_000466,NM_001282677,NM_001282678,NM_000466,NM_001282677,NM_001282678
NM_001278055,NM_014363,NM_001278055,NM_014363
NM_175918,NM_175918,NM_175918,NM_175918,NM_175918,NM_175918
NM_014949,NM_014949
NM_001014342,NM_001014342
NM_001173984,NM_013263,NM_001173984,NM_013263
NM_001207005,NM_181756,NM_001207005,NM_181756
NM_001271783,NM_001271784,NM_018099,NM_001271783,NM_001271784,NM_018099
NM_001272011,NM_014226,NM_001272011,NM_014226
NM_152945,NM_152945
NM_001290224,NM_199259,NM_199260,NM_199261,NM_001290224,NM_199259,NM_199260,NM_1992
NM_001321859,NM_001321860,NM_001321861,NM_001321862,NM_001321863,NM_001321904,NM_020
NM_001080850,NM_001080850
NM_001257190,NM_172020,NM_001257190,NM_172020
NM_001013843,NM_024755,NM_001013843,NM_024755
NM_002568,NM_002568
NM_001204087,NM_002249,NM_170782,NM_001204087,NM_002249,NM_170782
NM_000721,NM_001205293,NM_001205294,NM_000721,NM_001205293,NM_001205294
NM_001308079,NM_001308079
NM_001003407,NM_001003408,NM_001322882,NM_001322883,NM_001322884,NM_001322885,NM_001
NM_001040108,NM_014381,NM_001040108,NM_014381
NM_001282606,NM_001282610,NM_016056,NM_001282606,NM_001282610,NM_016056
NM_001319238,NM_001319239,NM_014827,NM_001319238,NM_001319239,NM_014827
NM_001002924,NM_001284,NM_001318091,NM_001318093,NM_001318094,NM_001002924,NM_001284
NM_003008,NM_003008
NM_001271,NM_001271
NM_001203260,NM_001203261,NM_001204054,NM_004549,NM_001203260,NM_001203261,NM_001204
NM_001126057,NM_001126058,NM_001190348,NM_001190349,NM_033317,NM_001126057,NM_00112
NM_033060,NM_033060
NM_201269,NM_201269
NM_001308116,NM_024947,NM_001308116,NM_024947
NM_033188,NM_033188
NM_006121,NM_006121
NM_017481,NM_017481
NM_014698,NM_014698
NM_002976,NM_002976
NM_178354,NM_178354
NM_152672,NM_152672
NM_000254,NM_001291939,NM_001291940,NM_000254,NM_001291939,NM_001291940
NM_015063,NM_015063
NM_182832,NM_182832
NM_007113,NM_007113
NM_001240,NM_001240
NM_016216,NM_016216
NM_014729,NM_014729

NM_006231,NM_006231
NM_014005,NM_014005
NM_001142556,NM_001142557,NM_012484,NM_012485,NM_001142556,NM_001142557,NM_012484,NM
NM_001276385,NM_001276385
NM_021076,NM_021076
NM_001004056,NM_001004057,NM_005307,NM_182982,NM_001004056,NM_001004057,NM_005307,NM
NM_001316951,NM_018559,NM_001316951,NM_018559
NM_001128620,NM_002576,NM_001128620,NM_002576
NM_030785,NM_030785
NM_001001480,NM_001001480
NM_001278055,NM_014363
NM_001278055,NM_014363
NM_001278055,NM_014363
NM_000254,NM_001291939,NM_001291940
NM_001207005,NM_181756
NM_175918,NM_175918,NM_175918
NM_001257190,NM_172020
NM_001173984,NM_013263
NM_002568
NM_001203260,NM_001203261,NM_001204054,NM_004549
NM_000466,NM_001282677,NM_001282678
NM_001002924,NM_001284,NM_001318091,NM_001318093,NM_001318094
NM_001308116,NM_024947
NM_000721,NM_001205293,NM_001205294
NM_001204087,NM_002249,NM_170782
NM_001282609
NM_001290224,NM_199259,NM_199260,NM_199261
NM_001301043,NM_001301044,NM_014333
NM_015063
NM_001003407,NM_001003408,NM_001322882,NM_001322883,NM_001322884,NM_001322885,NM_001
NM_016216
NM_001308079
NM_014949
NM_001014342
NM_001040108,NM_014381
NM_001319238,NM_001319239,NM_014827
NM_001272011,NM_014226
NM_152945
NM_002976
NM_006231
NM_014698
NM_014005
NM_017481
NM_001276385
NM_001315529,NM_006031
NM_001010870,NM_001168359
NM_001142556,NM_001142557,NM_012484,NM_012485

NM_182832
NM_033060
NM_001240
NM_178354
NM_003008
NM_001080850
NM_001004056,NM_001004057,NM_005307,NM_182982
NM_001271783,NM_001271784,NM_018099
NM_007113
NM_001128620,NM_002576
NM_201269
NM_152672
NM_006121
NM_001013843,NM_024755
NM_001126057,NM_001126058,NM_001190348,NM_001190349,NM_033317
NM_033188
NM_001316951,NM_018559
NM_001001480
NM_001271
NM_030785
NM_021076
NM_001321859,NM_001321860,NM_001321861,NM_001321862,NM_001321863,NM_001321904,NM_02[illegible]
NM_014729
NM_001003407,NM_001003408,NM_001322882,NM_001322883,NM_001322884,NM_001322885,NM_001[illegible]
NM_001003407,NM_001003408,NM_001322882,NM_001322883,NM_001322884,NM_001322885,NM_001[illegible]
NM_003683,NM_003683
NM_003108,NM_003108
NM_003683
NM_003108
NM_001258374,NM_001258375,NM_001258376,NM_021235,NM_001258374,NM_001258375,NM_00125[illegible]
NM_001271866,NM_001271867,NM_001271868,NM_001516,NM_001271866,NM_001271867,NM_00127[illegible]
NM_001318842,NM_001318847,NM_001318848,NM_001318857,NM_001318858,NM_006586,NM_00131[illegible]
NM_001258374,NM_001258375,NM_001258376,NM_021235
NM_001318842,NM_001318847,NM_001318848,NM_001318857,NM_001318858,NM_006586
NM_001271866,NM_001271867,NM_001271868,NM_001516
NM_001023563,NM_001023563
NM_003012,NM_003012
NM_001099218,NM_001321233,NM_001099218,NM_001321233
NM_004065,NM_004065
NM_001199160,NM_001199161,NM_001199162,NM_006677,NM_001199160,NM_001199161,NM_00119[illegible]
NM_001304562,NM_001304563,NM_173618,NM_001304562,NM_001304563,NM_173618
NM_001130110,NM_015559,NM_001130110,NM_015559
NM_003012
NM_004065
NM_001099218,NM_001321233
NM_001199160,NM_001199161,NM_001199162,NM_006677
NM_001023563

NM_001130110,NM_015559
NM_001304562,NM_001304563,NM_173618
NM_022454,NM_022454
NM_015656,NM_015656
NM_015656,NM_015656
NM_001080826,NM_001080826
NM_000552,NM_000552
NM_001130110,NM_015559,NM_001130110,NM_015559
NM_022454
NM_000552
NM_015656
NM_001080826
NM_001130110,NM_015559
NM_198148,NM_198148
NM_006015,NM_139135,NM_006015,NM_139135
NM_000552,NM_000552
NM_006248,NM_006248
NM_001130110,NM_015559,NM_001130110,NM_015559
NM_001038640,NM_001038640
NM_001317012,NM_001317019,NM_022482,NM_001317012,NM_001317019,NM_022482
NM_006015,NM_139135
NM_000552
NM_198148
NM_006248
NM_001038640
NM_001130110,NM_015559
NM_001317012,NM_001317019,NM_022482
NM_014246,NM_014246
NM_001017395,NM_001128224,NM_001017395,NM_001128224
NM_006015,NM_139135,NM_006015,NM_139135
NM_000552,NM_000552
NM_001038640,NM_001038640
NM_018416,NM_018416
NM_207371,NM_207371
NM_006015,NM_139135
NM_000552
NM_014246
NM_001017395,NM_001128224
NM_001038640
NM_018416
NM_207371
NM_006080,NM_006080
NM_001244249,NM_001641,NM_080648,NM_080649,NM_001244249,NM_001641,NM_080648,NM_08064[illegible]
NM_004933,NM_004933
NM_006080
NM_004933
NM_001244249,NM_001641,NM_080648,NM_080649

NM_000268,NM_016418,NM_181825,NM_181828,NM_181829,NM_181830,NM_181831,NM_181832,NM_
NM_001009608,NM_001009608
NM_001304562,NM_173618,NM_001304562,NM_173618
NM_001009608
NM_001304562,NM_173618
NM_000268,NM_016418,NM_181825,NM_181828,NM_181829,NM_181830,NM_181831,NM_181832
NM_014639,NM_014639
NM_021021,NM_021021
NM_017481,NM_017481
NM_033068,NM_033068
NM_015306,NM_015306
NM_014639
NM_033068
NM_015306
NM_021021
NM_017481
NM_001291980,NM_002011,NM_022963,NM_213647,NM_001291980,NM_002011,NM_022963,NM_2136
NM_001286581,NM_001286582,NM_001286583,NM_020901,NM_001286581,NM_001286582,NM_00128
NM_020528,NM_020528
NM_183373,NM_183373
NM_006248,NM_006248
NM_001286581,NM_001286582,NM_001286583,NM_020901
NM_020528
NM_001291980,NM_002011,NM_022963,NM_213647
NM_183373
NM_006248
NM_001170690,NM_001170690
NM_001142319,NM_001142319
NM_198834,NM_198836,NM_198837,NM_198838,NM_198839,NM_198834,NM_198836,NM_198837,NM_
NM_000548,NM_001077183,NM_001114382,NM_001318827,NM_001318829,NM_001318831,NM_00131
NM_012079,NM_012079
NM_138348,NM_138348
NM_001007471,NM_020952,NM_024971,NM_206944,NM_206945,NM_206946,NM_206947,NM_001007
NM_012079
NM_025203
NM_001170690
NM_000548,NM_001077183,NM_001114382,NM_001318827,NM_001318829,NM_001318831,NM_00131
NM_138348
NM_001007471,NM_020952,NM_024971,NM_206944,NM_206945,NM_206946,NM_206947
NM_198834,NM_198836,NM_198837,NM_198838,NM_198839
NM_001098407,NM_001127212,NM_001472,NM_012196,NM_001098407,NM_001127212,NM_001472,NM
NM_001173984,NM_013263,NM_001173984,NM_013263
NM_000268,NM_016418,NM_181825,NM_181828,NM_181829,NM_181830,NM_181831,NM_181832,NM_
NM_001278629,NM_001278630,NM_152786,NM_001278629,NM_001278630,NM_152786
NM_152414,NM_152414
NM_001173984,NM_013263
NM_001098407,NM_001127212,NM_001472,NM_012196

NM_152414
NM_000268,NM_016418,NM_181825,NM_181828,NM_181829,NM_181830,NM_181831,NM_181832
NM_001278629,NM_001278630,NM_152786
NM_174922,NM_174922
NM_001114380,NM_002209,NM_001114380,NM_002209
NM_198963,NM_198963
NM_006248,NM_006248
NM_001297439,NM_001528,NM_001297439,NM_001528
NM_001305424,NM_020066,NM_001305424,NM_020066
NM_174922
NM_198963
NM_001114380,NM_002209
NM_001297439,NM_001528
NM_001305424,NM_020066
NM_006248
NM_006768,NM_006768
NM_001080396,NM_001080396
NM_001080396
NM_006768
NM_033071,NM_182961,NM_033071,NM_182961
NM_001098407,NM_001127212,NM_001472,NM_012196,NM_001098407,NM_001127212,NM_001472,NM
NM_001098407,NM_001127212,NM_001472,NM_012196
NM_033071,NM_182961
NM_001290224,NM_199259,NM_199260,NM_199261,NM_001290224,NM_199259,NM_199260,NM_1992
NM_001098407,NM_001127212,NM_001472,NM_012196,NM_001098407,NM_001127212,NM_001472,NM
NM_001290224,NM_199259,NM_199260,NM_199261
NM_001098407,NM_001127212,NM_001472,NM_012196
NM_001098515,NM_145015,NM_001098515,NM_145015
NM_004599,NM_004599
NM_001098515,NM_145015
NM_004599
NM_031303,NM_031303
NM_005148,NM_054035,NM_005148,NM_054035
NM_001256486,NM_001256487,NM_001256488,NM_004487,NM_001256486,NM_001256487,NM_00125
NM_031303
NM_005148,NM_054035
NM_001256486,NM_001256487,NM_001256488,NM_004487
NM_001098627,NM_001098629,NM_001098630,NM_032643,NM_001098627,NM_001098629,NM_00109
NM_001098627,NM_001098629,NM_001098630,NM_032643
NM_001204087,NM_002249
NM_001080458,NM_001080458
NM_001080434,NM_001080434
NM_032853,NM_032853
NM_001009609,NM_001009609
NM_173493,NM_173493
NM_001962,NM_001962
NM_001080458

NM_173493
NM_001080434
NM_001962
NM_032853
NM_001009609
NM_014109,NM_014109
NM_014674,NM_014674
NM_001321196,NM_012254,NM_001321196,NM_012254
NM_001172698,NM_001172699,NM_133263,NM_001172698,NM_001172699,NM_133263
NM_015711,NM_015711
NM_001112808,NM_015978,NM_001112808,NM_015978
NM_001184808,NM_001242614,NM_031462,NM_001184808,NM_001242614,NM_031462
NM_014729,NM_014729
NM_001112808,NM_015978
NM_014109
NM_015711
NM_001321196,NM_012254
NM_014674
NM_001172698,NM_001172699,NM_133263
NM_001184808,NM_001242614,NM_031462
NM_014729
NM_000268,NM_016418,NM_181825,NM_181828,NM_181829,NM_181830,NM_181831,NM_181832,NM_
NM_001077206,NM_001077207,NM_001077208,NM_001191049,NM_001300744,NM_001300745,NM_001
NM_145804,NM_145804
NM_001243403,NM_001243403
NM_001318015,NM_001318023,NM_032799,NM_001318015,NM_001318023,NM_032799
NM_015016,NM_015016
NM_016107,NM_016107
NM_001318015,NM_001318023,NM_032799
NM_001077206,NM_001077207,NM_001077208,NM_001191049,NM_001300744,NM_001300745,NM_001
NM_001243403
NM_015016
NM_145804
NM_000268,NM_016418,NM_181825,NM_181828,NM_181829,NM_181830,NM_181831,NM_181832
NM_016107
NM_012168,NM_012168
NM_006887,NM_006887
NM_001286074,NM_004606,NM_138923,NM_001286074,NM_004606,NM_138923
NM_001142864,NM_001142864
NM_012168
NM_006887
NM_001286074,NM_004606,NM_138923
NM_006981,NM_173199,NM_173200
NM_001142864
NM_001017395,NM_001128224,NM_001017395,NM_001128224
NM_001173984,NM_013263,NM_001173984,NM_013263
NM_001103146,NM_001103147,NM_001103148,NM_015575,NM_001103146,NM_001103147,NM_00110[illegible]

NM_000332,NM_001128164,NM_000332,NM_001128164
NM_001145444,NM_001145445,NM_020706,NM_001145444,NM_001145445,NM_020706
NM_001173984,NM_013263
NM_001017395,NM_001128224
NM_001103146,NM_001103147,NM_001103148,NM_015575
NM_001145444,NM_001145445,NM_020706
NM_000332,NM_001128164
NM_015848,NM_015848
NM_001173984,NM_013263,NM_001173984,NM_013263
NM_001005735,NM_001257387,NM_007194,NM_145862,NM_001005735,NM_001257387,NM_007194,N[illegible]
NM_000332,NM_001128164,NM_000332,NM_001128164
NM_001080396,NM_001080396
NM_001145444,NM_001145445,NM_020706,NM_001145444,NM_001145445,NM_020706
NM_001127708,NM_001127709,NM_001127710,NM_001303232,NM_005807,NM_001127708,NM_00112[illegible]
NM_001164766,NM_006885,NM_001164766,NM_006885
NM_015848
NM_001173984,NM_013263
NM_001005735,NM_001257387,NM_007194,NM_145862
NM_001145444,NM_001145445,NM_020706
NM_001164766,NM_006885
NM_001127708,NM_001127709,NM_001127710,NM_001303232,NM_005807
NM_001080396
NM_001136002
NM_000332,NM_001128164
NM_001284291,NM_024814,NM_001284291,NM_024814
NM_003038,NM_003038
NM_153757,NM_153757
NM_001284291,NM_024814
NM_003038
NM_153757
NM_001243799,NM_183422
NM_032142,NM_032142
NM_001142730,NM_001142730
NM_032142
NM_001142730
NM_001243799,NM_183422
NM_001100397,NM_001282899,NM_001282900,NM_001282901,NM_007023,NM_001100397,NM_00128[illegible]
NM_002786,NM_148976,NM_002786,NM_148976
NM_004585,NM_004585
NM_001204087,NM_002249,NM_170782,NM_001204087,NM_002249,NM_170782
NM_138426,NM_138426
NM_021614,NM_021614
NM_006248,NM_006248
NM_001166018,NM_007252,NM_001166018,NM_007252
NM_021614
NM_004585
NM_001100397,NM_001282899,NM_001282900,NM_001282901,NM_007023

NM_001204087,NM_002249,NM_170782
NM_002786,NM_148976
NM_138426
NM_001166018,NM_007252
NM_006248
NM_001136046,NM_032265,NM_001136046,NM_032265
NM_014345,NM_014345
NM_001100397,NM_001282899,NM_001282900,NM_001282901,NM_007023,NM_001100397,NM_001282
NM_001164211,NM_001164213,NM_015116,NM_001164211,NM_001164213,NM_015116
NM_080473,NM_080473
NM_001287489,NM_194248,NM_001287489,NM_194248
NM_007349,NM_007349
NM_020747,NM_020747
NM_006248,NM_006248
NM_014345
NM_001100397,NM_001282899,NM_001282900,NM_001282901,NM_007023
NM_001136046,NM_001267822,NM_032265
NM_001164211,NM_001164213,NM_015116
NM_080473
NM_001287489,NM_194248
NM_020747
NM_007349
NM_006248
NM_001136002
NM_144505,NM_144505
NM_000051,NM_000051
NM_001276343,NM_133446,NM_001276343,NM_133446
NM_001098407,NM_001127212,NM_001472,NM_012196,NM_001098407,NM_001127212,NM_001472,NM
NM_001242680,NM_001242680
NM_207371,NM_207371
NM_001276343,NM_133446
NM_007196
NM_001242680
NM_000051
NM_001098407,NM_001127212,NM_001472,NM_012196
NM_207371
NM_015134,NM_201274,NM_015134,NM_201274
NM_000051,NM_000051
NM_178448,NM_178448
NM_004104,NM_004104
NM_177424,NM_177424
NM_053284,NM_053284
NM_001242680,NM_001242680
NM_015134,NM_201274
NM_177424
NM_178448
NM_001242680

NM_000051
NM_004104
NM_001301043,NM_001301044,NM_014333
NM_053284
NM_001018159,NM_001018160,NM_001286500,NM_003905,NM_001018159,NM_001018160,NM_00128[illegible]
NM_080863,NM_080863
NM_002867,NM_002867
NM_001267716,NM_001267716
NM_080863
NM_001018159,NM_001018160,NM_001286500,NM_003905
NM_001267716
NM_002867
NM_198827,NM_198827
NM_001018159,NM_001018160,NM_001286500,NM_003905,NM_001018159,NM_001018160,NM_00128[illegible]
NM_001320780,NM_001320780
NM_001199629,NM_002475,NM_001199629,NM_002475
NM_198827
NM_001199629,NM_002475
NM_001018159,NM_001018160,NM_001286500,NM_003905
NM_001320780
NM_001018159,NM_001018160,NM_001286500,NM_003905,NM_001018159,NM_001018160,NM_00128[illegible]
NM_001282541,NM_001282542,NM_003176,NM_001282541,NM_001282542,NM_003176
NM_001127493,NM_001148,NM_020977,NM_001127493,NM_001148,NM_020977
NM_018902,NM_031861,NM_018902,NM_031861
NM_001081676,NM_001081677,NM_006922,NM_001081676,NM_001081677,NM_006922
NM_001282541,NM_001282542,NM_003176
NM_001018159,NM_001018160,NM_001286500,NM_003905
NM_018902,NM_031861
NM_001127493,NM_001148,NM_020977
NM_001081676,NM_001081677,NM_006922
NM_001018159,NM_001018160,NM_001286500,NM_003905,NM_001018159,NM_001018160,NM_00128[illegible]
NM_024867,NM_024867
NM_003565,NM_003565
NM_002635,NM_005888,NM_213611,NM_002635,NM_005888,NM_213611
NM_001291366,NM_001291367,NM_001291366,NM_001291367
NM_001256071,NM_001256071
NM_001127493,NM_001148,NM_020977,NM_001127493,NM_001148,NM_020977
NM_001291965,NM_006646,NM_001291965,NM_006646
NM_004621,NM_004621
NM_001199237,NM_001199238,NM_001199239,NM_001199240,NM_033177,NM_001199237,NM_00119[illegible]
NM_001040153,NM_001242868,NM_001242869,NM_001242870,NM_001242871,NM_144595,NM_00104[illegible]
NM_001291366,NM_001291367
NM_024867
NM_003565
NM_001018159,NM_001018160,NM_001286500,NM_003905
NM_001040153,NM_001242868,NM_001242869,NM_001242870,NM_001242871,NM_144595
NM_004621

NM_002635,NM_005888,NM_213611
NM_001199237,NM_001199238,NM_001199239,NM_001199240,NM_033177
NM_001256071
NM_001127493,NM_001148,NM_020977
NM_001291965,NM_006646
NM_001304482,NM_015466,NM_001304482,NM_015466
NM_000268,NM_016418,NM_181825,NM_181832,NM_181833,NM_000268,NM_016418,NM_181825,NM_
NM_020780,NM_020780
NM_001522,NM_001522
NM_144633,NM_144633
NM_000268,NM_016418,NM_181825,NM_181832,NM_181833
NM_001304482,NM_015466
NM_020780
NM_001522
NM_144633
NM_175885,NM_175885
NM_000268,NM_016418,NM_181825,NM_181832,NM_181833,NM_000268,NM_016418,NM_181825,NM_
NM_001100122,NM_014963,NM_001100122,NM_014963
NM_004656,NM_004656
NM_001522,NM_001522
NM_148894,NM_148894
NM_004455,NM_004455
NM_148894
NM_000268,NM_016418,NM_181825,NM_181832,NM_181833
NM_004455
NM_001522
NM_004656
NM_175885
NM_001100122,NM_014963
NM_001105521,NM_001323086,NM_001323087,NM_001323088,NM_001323090,NM_001105521,NM_001
NM_000268,NM_016418,NM_181825,NM_181832,NM_181833,NM_000268,NM_016418,NM_181825,NM_
NM_004656,NM_004656
NM_001522,NM_001522
NM_148894,NM_148894
NM_173587,NM_173587
NM_138983,NM_138983
NM_001145207,NM_001145207
NM_003487,NM_139215,NM_003487,NM_139215
NM_148894
NM_000268,NM_016418,NM_181825,NM_181832,NM_181833
NM_001522
NM_004656
NM_001145207
NM_001105521,NM_001323086,NM_001323087,NM_001323088,NM_001323090
NM_138983
NM_173587
NM_003487,NM_139215

NM_000268,NM_016418,NM_181825,NM_181832,NM_181833,NM_000268,NM_016418,NM_181825,NM_
NM_001522,NM_001522
NM_148894,NM_148894
NM_148894
NM_000268,NM_016418,NM_181825,NM_181832,NM_181833
NM_001522
NM_145912,NM_145912
NM_001308174,NM_138790,NM_001308174,NM_138790
NM_000268,NM_016418,NM_181825,NM_181832,NM_181833,NM_000268,NM_016418,NM_181825,NM_
NM_030975,NM_030975
NM_004656,NM_004656
NM_001039792,NM_001039792
NM_001522,NM_001522
NM_148894,NM_148894
NM_001159651,NM_138369,NM_001159651,NM_138369
NM_148894
NM_000268,NM_016418,NM_181825,NM_181832,NM_181833
NM_145912
NM_001522
NM_001308174,NM_138790
NM_004656
NM_001039792
NM_030975
NM_001159651,NM_138369
NM_152424,NM_152424
NM_001271604,NM_001271604
NM_001195573,NM_001271282,NM_001291628,NM_030621,NM_177438,NM_001195573,NM_00127128[illegible]
NM_007261,NM_007261
NM_001282301,NM_001282301
NM_001271604
NM_152424
NM_007261
NM_001195573,NM_001271282,NM_001291628,NM_030621,NM_177438
NM_001282301
NM_012168,NM_012168
NM_012168
NM_001083585,NM_001291581,NM_004703,NM_001083585,NM_001291581,NM_004703
NM_001083585,NM_001291581,NM_004703
NM_001164463,NM_005054,NM_182588,NM_001164463,NM_005054,NM_182588
NM_006678,NM_006678
NM_006039,NM_006039
NM_023919,NM_023919
NM_021076,NM_021076
NM_182588,NM_182588
NM_006678
NM_023919
NM_001164463,NM_005054

NM_182588
NM_006039
NM_021076
NM_002439,NM_002439
NM_002439
NM_006387
NM_001304388
ENST00000361681
ENST00000441802,ENST00000509647
ENST00000361567
ENST00000346061,ENST00000418749
ENST00000278882,ENST00000358464,ENST00000439954
ENST00000262189,ENST00000355193,ENST00000558084
ENST00000348028,ENST00000349350,ENST00000421100,ENST00000427578,ENST00000443370,ENST00000
ENST00000361789
ENST00000361567
ENST00000361567
ENST00000299440,ENST00000534663
ENST00000367482,ENST00000367483,ENST00000367484,ENST00000367485,ENST00000367486,ENST00000
ENST00000361567
ENST00000367483,ENST00000367484,ENST00000367485,ENST00000367486,ENST00000445192
ENST00000389484
ENST00000356920,ENST00000357462,ENST00000358087,ENST00000360967,ENST00000361163,ENST00000
ENST00000367482,ENST00000367483,ENST00000367484,ENST00000367485,ENST00000367486,ENST00000
ENST00000302763,ENST00000355078,ENST00000518825,ENST00000518910,ENST00000520158
ENST00000357462,ENST00000360967,ENST00000361163,ENST00000409914
ENST00000262189,ENST00000355193,ENST00000424877
ENST00000299440,ENST00000299440,ENST00000534663,ENST00000534663
ENST00000361567
ENST00000361789
ENST00000356920,ENST00000356920,ENST00000356920,ENST00000356920,ENST00000356920,ENST00000
ENST00000361567
ENST00000361567
ENST00000389484
ENST00000441802,ENST00000441802,ENST00000509647,ENST00000509647
ENST00000361567
ENST00000278882,ENST00000278882,ENST00000278882,ENST00000358464,ENST00000358464,ENST00000
ENST00000361681
ENST00000348028,ENST00000348028,ENST00000348028,ENST00000348028,ENST00000348028,ENST00000
ENST00000367482,ENST00000367482,ENST00000367482,ENST00000367482,ENST00000367482,ENST00000
ENST00000346061,ENST00000346061,ENST00000418749,ENST00000418749
ENST00000302763,ENST00000302763,ENST00000302763,ENST00000302763,ENST00000302763,ENST00000
ENST00000262189,ENST00000262189,ENST00000262189,ENST00000355193,ENST00000355193,ENST00000
ENST00000357462,ENST00000357462,ENST00000357462,ENST00000357462,ENST00000360967,ENST00000
ENST00000367483,ENST00000367483,ENST00000367483,ENST00000367483,ENST00000367483,ENST00000
ENST00000367482,ENST00000367482,ENST00000367482,ENST00000367482,ENST00000367482,ENST00000
ENST00000262189,ENST00000262189,ENST00000262189,ENST00000355193,ENST00000355193,ENST00000

ENST00000361681
ENST00000441802,ENST00000509647
ENST00000357462,ENST00000360967,ENST00000361163,ENST00000409914
ENST00000361789
ENST00000361567
ENST00000346061,ENST00000418749
ENST00000278882,ENST00000358464,ENST00000439954
ENST00000361899
ENST00000262189,ENST00000355193,ENST00000558084
ENST00000348028,ENST00000349350,ENST00000421100,ENST00000427578,ENST00000443370,ENST00000
ENST00000361789
ENST00000361567
ENST00000361567
ENST00000299440,ENST00000534663
ENST00000349310,ENST00000402615,ENST00000407796,ENST00000554581,ENST00000554848,ENST00000
ENST00000367482,ENST00000367483,ENST00000367484,ENST00000367485,ENST00000367486,ENST00000
ENST00000361567
ENST00000367483,ENST00000367484,ENST00000367485,ENST00000367486,ENST00000445192
ENST00000389484
ENST00000361624
ENST00000356920,ENST00000357462,ENST00000358087,ENST00000360967,ENST00000361163,ENST00000
ENST00000367482,ENST00000367483,ENST00000367484,ENST00000367485,ENST00000367486,ENST00000
ENST00000302763,ENST00000355078,ENST00000518825,ENST00000518910,ENST00000520158
ENST00000357462,ENST00000360967,ENST00000361163,ENST00000409914
ENST00000262189,ENST00000355193,ENST00000424877
ENST00000299440,ENST00000299440,ENST00000534663,ENST00000534663
ENST00000361899
ENST00000349310,ENST00000349310,ENST00000349310,ENST00000349310,ENST00000349310,ENST00000
ENST00000361567
ENST00000361789
ENST00000356920,ENST00000356920,ENST00000356920,ENST00000356920,ENST00000356920,ENST00000
ENST00000357462,ENST00000357462,ENST00000357462,ENST00000357462,ENST00000357462,ENST00000
ENST00000361567
ENST00000361567
ENST00000389484
ENST00000361789
ENST00000441802,ENST00000441802,ENST00000509647,ENST00000509647
ENST00000361567
ENST00000278882,ENST00000278882,ENST00000278882,ENST00000358464,ENST00000358464,ENST00000
ENST00000361681
ENST00000348028,ENST00000348028,ENST00000348028,ENST00000348028,ENST00000348028,ENST00000
ENST00000367482,ENST00000367482,ENST00000367482,ENST00000367482,ENST00000367482,ENST00000
ENST00000346061,ENST00000346061,ENST00000418749,ENST00000418749
ENST00000302763,ENST00000302763,ENST00000302763,ENST00000302763,ENST00000302763,ENST00000
ENST00000262189,ENST00000262189,ENST00000262189,ENST00000355193,ENST00000355193,ENST00000
ENST00000357462,ENST00000357462,ENST00000357462,ENST00000357462,ENST00000360967,ENST00000
ENST00000357462,ENST00000357462,ENST00000357462,ENST00000357462,ENST00000357462,ENST00000

ENST00000361624
ENST00000367483,ENST00000367483,ENST00000367483,ENST00000367483,ENST00000367483,ENST00000[illegible]
ENST00000367482,ENST00000367482,ENST00000367482,ENST00000367482,ENST00000367482,ENST00000[illegible]
ENST00000262189,ENST00000262189,ENST00000262189,ENST00000355193,ENST00000355193,ENST00000[illegible]
ENST00000281815,ENST00000338347
ENST00000342960,ENST00000369338,ENST00000369339,ENST00000490598
ENST00000407877,ENST00000542587,ENST00000586703
ENST00000355086,ENST00000357825,ENST00000543397
ENST00000439954
ENST00000342960,ENST00000490598
ENST00000346061,ENST00000418749
ENST00000420699,ENST00000461153
ENST00000281815,ENST00000338347
ENST00000263093,ENST00000601355
ENST00000425945
ENST00000278882,ENST00000358464,ENST00000439954
ENST00000358984,ENST00000447268,ENST00000580206
ENST00000376767
ENST00000490686
ENST00000220959,ENST00000520539,ENST00000521922
ENST00000310816,ENST00000359221,ENST00000361569,ENST00000395161,ENST00000503911,ENST00000[illegible]
ENST00000439326
ENST00000300134,ENST00000454075,ENST00000537215,ENST00000538913,ENST00000543873,ENST00000[illegible]
ENST00000376223,ENST00000430996
ENST00000367500,ENST00000367501
ENST00000299798
ENST00000358970
ENST00000328886,ENST00000538566
ENST00000334984,ENST00000349384,ENST00000379933,ENST00000379938,ENST00000483150
ENST00000283507,ENST00000366832
ENST00000368799
ENST00000355867,ENST00000375398,ENST00000375400,ENST00000535393,ENST00000538146
ENST00000281815,ENST00000338347
ENST00000389793,ENST00000389794
ENST00000350908,ENST00000360232,ENST00000390668,ENST00000393489,ENST00000393491,ENST00000[illegible]
ENST00000408960
ENST00000358970,ENST00000511306
ENST00000368799
ENST00000258729
ENST00000342960
ENST00000300061
ENST00000340446
ENST00000489525
ENST00000510045
ENST00000355014,ENST00000368282,ENST00000368284,ENST00000368285,ENST00000368286
ENST00000336930,ENST00000529015
ENST00000265368,ENST00000341594,ENST00000356820,ENST00000367255,ENST00000423061,ENST00000[illegible]

ENST00000328070
ENST00000389362
ENST00000391413
ENST00000244769,ENST00000436367
ENST00000333891,ENST00000423517
ENST00000355014,ENST00000355014,ENST00000355014,ENST00000355014,ENST00000355014,ENST00000[illegible]
ENST00000358970,ENST00000358970,ENST00000511306,ENST00000511306
ENST00000350908,ENST00000350908,ENST00000350908,ENST00000350908,ENST00000350908,ENST00000[illegible]
ENST00000490686
ENST00000281815,ENST00000281815,ENST00000338347,ENST00000338347
ENST00000510045
ENST00000489525
ENST00000300134,ENST00000300134,ENST00000300134,ENST00000300134,ENST00000300134,ENST00000[illegible]
ENST00000355086,ENST00000355086,ENST00000355086,ENST00000357825,ENST00000357825,ENST00000[illegible]
ENST00000407877,ENST00000407877,ENST00000407877,ENST00000542587,ENST00000542587,ENST00000[illegible]
ENST00000367500,ENST00000367500,ENST00000367501,ENST00000367501
ENST00000439954
ENST00000220959,ENST00000220959,ENST00000220959,ENST00000520539,ENST00000520539,ENST00000[illegible]
ENST00000310816,ENST00000310816,ENST00000310816,ENST00000310816,ENST00000310816,ENST00000[illegible]
ENST00000425945
ENST00000300061
ENST00000418749
ENST00000376223,ENST00000376223,ENST00000430996,ENST00000430996
ENST00000408960
ENST00000336930,ENST00000336930,ENST00000529015,ENST00000529015
ENST00000278882,ENST00000278882,ENST00000278882,ENST00000358464,ENST00000358464,ENST00000[illegible]
ENST00000281815,ENST00000281815,ENST00000338347,ENST00000338347
ENST00000439326
ENST00000358970
ENST00000358970
ENST00000258729
ENST00000389793,ENST00000389793,ENST00000389794,ENST00000389794
ENST00000299798
ENST00000265368,ENST00000265368,ENST00000265368,ENST00000265368,ENST00000265368,ENST00000[illegible]
ENST00000376767
ENST00000340446
ENST00000334984,ENST00000334984,ENST00000334984,ENST00000334984,ENST00000334984,ENST00000[illegible]
ENST00000342960,ENST00000342960,ENST00000342960,ENST00000342960,ENST00000369338,ENST00000[illegible]
ENST00000281815,ENST00000281815,ENST00000338347,ENST00000338347
ENST00000358984,ENST00000358984,ENST00000358984,ENST00000447268,ENST00000447268,ENST00000[illegible]
ENST00000368799
ENST00000368799
ENST00000342960,ENST00000342960,ENST00000490598,ENST00000490598
ENST00000391413
ENST00000328886,ENST00000328886,ENST00000538566,ENST00000538566
ENST00000355867,ENST00000355867,ENST00000355867,ENST00000355867,ENST00000355867,ENST00000[illegible]
ENST00000328070

ENST00000420699,ENST00000420699,ENST00000461153,ENST00000461153
ENST00000263093,ENST00000263093,ENST00000601355,ENST00000601355
ENST00000342960
ENST00000389362
ENST00000333891,ENST00000333891,ENST00000423517,ENST00000423517
ENST00000283507,ENST00000283507,ENST00000366832,ENST00000366832
ENST00000244769,ENST00000244769,ENST00000436367,ENST00000436367
ENST00000281815,ENST00000338347
ENST00000376322,ENST00000376327
ENST00000407877,ENST00000542587,ENST00000586703
ENST00000307886,ENST00000528419
ENST00000359999,ENST00000373629,ENST00000373631,ENST00000460253
ENST00000283195
ENST00000439954
ENST00000342960,ENST00000490598
ENST00000346061,ENST00000418749
ENST00000312513,ENST00000580762,ENST00000582535
ENST00000314191,ENST00000338368
ENST00000281815,ENST00000338347
ENST00000283195
ENST00000263093,ENST00000601355
ENST00000243052,ENST00000538346,ENST00000550620
ENST00000275607,ENST00000395435,ENST00000395436,ENST00000413756,ENST00000413952,ENST00000[illegible]
ENST00000402038
ENST00000260947,ENST00000449967
ENST00000358984,ENST00000447268,ENST00000580206
ENST00000557056
ENST00000261883
ENST00000376767
ENST00000314367,ENST00000356521,ENST00000377400,ENST00000382447
ENST00000355300,ENST00000557798,ENST00000561030
ENST00000490686
ENST00000301421,ENST00000326856,ENST00000416184,ENST00000596931,ENST00000598239,ENST00000[illegible]
ENST00000220959,ENST00000520539,ENST00000521922
ENST00000310816,ENST00000359221,ENST00000361569,ENST00000395161,ENST00000503911,ENST00000[illegible]
ENST00000378802
ENST00000271883,ENST00000368331,ENST00000437809
ENST00000300134,ENST00000454075,ENST00000537215,ENST00000538913,ENST00000543873,ENST00000[illegible]
ENST00000441024
ENST00000376223,ENST00000430996
ENST00000367500,ENST00000367501
ENST00000299798
ENST00000328886,ENST00000538566
ENST00000287008,ENST00000357517,ENST00000394536,ENST00000456271,ENST00000503492,ENST00000[illegible]
ENST00000278882,ENST00000358464,ENST00000439954
ENST00000388820
ENST00000353796,ENST00000396912

ENST00000225296
ENST00000261716
ENST00000371494
ENST00000355867,ENST00000375398,ENST00000375400,ENST00000535393,ENST00000538146
ENST00000273395,ENST00000355385,ENST00000495514
ENST00000304833
ENST00000350908,ENST00000360232,ENST00000390668,ENST00000393489,ENST00000393491,ENST00000[illegible]
ENST00000255882,ENST00000572273
ENST00000408960
ENST00000509274
ENST00000300061
ENST00000340446
ENST00000377833
ENST00000301067
ENST00000361381
ENST00000262189,ENST00000355193,ENST00000558084
ENST00000355014,ENST00000368282,ENST00000368284,ENST00000368285,ENST00000368286
ENST00000336930,ENST00000529015
ENST00000328070
ENST00000389362
ENST00000391413
ENST00000397104
ENST00000358491
ENST00000376536
ENST00000342175,ENST00000342992,ENST00000359218,ENST00000460472,ENST00000589042,ENST00000[illegible]
ENST00000333891,ENST00000423517
ENST00000355300,ENST00000355300,ENST00000355300,ENST00000557798,ENST00000557798,ENST00000[illegible]
ENST00000355014,ENST00000355014,ENST00000355014,ENST00000355014,ENST00000355014,ENST00000[illegible]
ENST00000255882,ENST00000255882,ENST00000572273,ENST00000572273
ENST00000350908,ENST00000350908,ENST00000350908,ENST00000350908,ENST00000350908,ENST00000[illegible]
ENST00000490686
ENST00000281815,ENST00000281815,ENST00000338347,ENST00000338347
ENST00000557056
ENST00000262189,ENST00000262189,ENST00000262189,ENST00000355193,ENST00000355193,ENST00000[illegible]
ENST00000300134,ENST00000300134,ENST00000300134,ENST00000300134,ENST00000300134,ENST00000[illegible]
ENST00000358491
ENST00000441024
ENST00000261883
ENST00000407877,ENST00000407877,ENST00000407877,ENST00000542587,ENST00000542587,ENST00000[illegible]
ENST00000367500,ENST00000367500,ENST00000367501,ENST00000367501
ENST00000439954
ENST00000220959,ENST00000220959,ENST00000220959,ENST00000520539,ENST00000520539,ENST00000[illegible]
ENST00000361381
ENST00000359999,ENST00000359999,ENST00000359999,ENST00000359999,ENST00000373629,ENST00000[illegible]
ENST00000225296
ENST00000314191,ENST00000314191,ENST00000338368,ENST00000338368
ENST00000342175,ENST00000342175,ENST00000342175,ENST00000342175,ENST00000342175,ENST00000[illegible]

ENST00000307886,ENST00000307886,ENST00000528419,ENST00000528419
ENST00000314367,ENST00000314367,ENST00000314367,ENST00000314367,ENST00000356521,ENST00000
ENST00000283195
ENST00000310816,ENST00000310816,ENST00000310816,ENST00000310816,ENST00000310816,ENST00000
ENST00000300061
ENST00000418749
ENST00000304833
ENST00000377833
ENST00000260947,ENST00000260947,ENST00000449967,ENST00000449967
ENST00000376223,ENST00000376223,ENST00000430996,ENST00000430996
ENST00000273395,ENST00000273395,ENST00000273395,ENST00000355385,ENST00000355385,ENST00000
ENST00000312513,ENST00000312513,ENST00000312513,ENST00000580762,ENST00000580762,ENST00000
ENST00000408960
ENST00000388820
ENST00000376322,ENST00000376322,ENST00000376327,ENST00000376327
ENST00000301067
ENST00000271883,ENST00000271883,ENST00000271883,ENST00000368331,ENST00000368331,ENST00000
ENST00000371494
ENST00000336930,ENST00000336930,ENST00000529015,ENST00000529015
ENST00000281815,ENST00000281815,ENST00000338347,ENST00000338347
ENST00000402038
ENST00000283195
ENST00000326856,ENST00000326856,ENST00000326856,ENST00000596931,ENST00000596931,ENST00000
ENST00000243052,ENST00000243052,ENST00000243052,ENST00000538346,ENST00000538346,ENST00000
ENST00000299798
ENST00000275607,ENST00000275607,ENST00000275607,ENST00000275607,ENST00000275607,ENST00000
ENST00000509274
ENST00000376767
ENST00000340446
ENST00000353796,ENST00000353796,ENST00000396912,ENST00000396912
ENST00000358984,ENST00000358984,ENST00000358984,ENST00000447268,ENST00000447268,ENST00000
ENST00000342960,ENST00000342960,ENST00000490598,ENST00000490598
ENST00000536202
ENST00000397104
ENST00000391413
ENST00000328886,ENST00000328886,ENST00000538566,ENST00000538566
ENST00000287008,ENST00000287008,ENST00000287008,ENST00000287008,ENST00000287008,ENST00000
ENST00000355867,ENST00000355867,ENST00000355867,ENST00000355867,ENST00000355867,ENST00000
ENST00000328070
ENST00000263093,ENST00000263093,ENST00000601355,ENST00000601355
ENST00000278882,ENST00000278882,ENST00000278882,ENST00000358464,ENST00000358464,ENST00000
ENST00000378802
ENST00000389362
ENST00000333891,ENST00000333891,ENST00000423517,ENST00000423517
ENST00000398531
ENST00000328782
ENST00000407877,ENST00000542587,ENST00000586703

ENST00000325823,ENST00000396410,ENST00000447912,ENST00000450288,ENST00000455313,ENST00000
ENST00000399703,ENST00000602295
ENST00000263093,ENST00000601355
ENST00000301310,ENST00000586929
ENST00000490686
ENST00000220959,ENST00000520539,ENST00000521922
ENST00000310816,ENST00000359221,ENST00000361569,ENST00000395161,ENST00000503911,ENST00000
ENST00000300134,ENST00000454075,ENST00000537215,ENST00000538913,ENST00000543873,ENST00000
ENST00000376223,ENST00000430996
ENST00000367500,ENST00000367501
ENST00000299798
ENST00000369226,ENST00000369228
ENST00000328886,ENST00000538566
ENST00000355867,ENST00000375398,ENST00000375400,ENST00000535393,ENST00000538146
ENST00000350908,ENST00000360232,ENST00000390668,ENST00000393489,ENST00000393491,ENST00000
ENST00000408960
ENST00000532254,ENST00000533640
ENST00000300061
ENST00000340446
ENST00000348028,ENST00000349350,ENST00000421100,ENST00000427578,ENST00000443370,ENST00000
ENST00000391545
ENST00000336930,ENST00000529015
ENST00000328070
ENST00000367482,ENST00000367483,ENST00000367484,ENST00000367485,ENST00000367486,ENST00000
ENST00000350908,ENST00000350908,ENST00000350908,ENST00000350908,ENST00000350908,ENST00000
ENST00000490686
ENST00000399703,ENST00000399703,ENST00000602295,ENST00000602295
ENST00000399703,ENST00000399703,ENST00000602295,ENST00000602295
ENST00000532254,ENST00000532254,ENST00000533640,ENST00000533640
ENST00000325823,ENST00000325823,ENST00000325823,ENST00000325823,ENST00000325823,ENST00000
ENST00000300134,ENST00000300134,ENST00000300134,ENST00000300134,ENST00000300134,ENST00000
ENST00000328782
ENST00000407877,ENST00000407877,ENST00000407877,ENST00000542587,ENST00000542587,ENST00000
ENST00000367500,ENST00000367500,ENST00000367501,ENST00000367501
ENST00000220959,ENST00000220959,ENST00000220959,ENST00000520539,ENST00000520539,ENST00000
ENST00000310816,ENST00000310816,ENST00000310816,ENST00000310816,ENST00000310816,ENST00000
ENST00000391545
ENST00000300061
ENST00000376223,ENST00000376223,ENST00000430996,ENST00000430996
ENST00000369226,ENST00000369226,ENST00000369228,ENST00000369228
ENST00000301310,ENST00000301310,ENST00000586929,ENST00000586929
ENST00000408960
ENST00000336930,ENST00000336930,ENST00000529015,ENST00000529015
ENST00000299798
ENST00000340446
ENST00000328886,ENST00000328886,ENST00000538566,ENST00000538566
ENST00000355867,ENST00000355867,ENST00000355867,ENST00000355867,ENST00000355867,ENST00000

ENST00000328070
ENST00000263093,ENST00000263093,ENST00000601355,ENST00000601355
ENST00000348028,ENST00000348028,ENST00000348028,ENST00000348028,ENST00000348028,ENST00000
ENST00000367482,ENST00000367482,ENST00000367482,ENST00000367482,ENST00000367482,ENST00000
ENST00000304514,ENST00000409886
ENST00000304514,ENST00000304514,ENST00000409886,ENST00000409886
ENST00000226207
ENST00000304514,ENST00000409886
ENST00000226207
ENST00000304514,ENST00000304514,ENST00000409886,ENST00000409886
ENST00000304514,ENST00000409886
ENST00000331920,ENST00000375274,ENST00000375290,ENST00000430669,ENST00000437951,ENST00000
ENST00000331920,ENST00000331920,ENST00000331920,ENST00000331920,ENST00000331920,ENST00000
ENST00000304514,ENST00000304514,ENST00000409886,ENST00000409886
ENST00000263816
ENST00000262189,ENST00000355193,ENST00000558665
ENST00000262189,ENST00000262189,ENST00000262189,ENST00000355193,ENST00000355193,ENST00000
ENST00000263816
ENST00000295956,ENST00000348383,ENST00000357272,ENST00000358537,ENST00000419752,ENST00000
ENST00000295956,ENST00000295956,ENST00000295956,ENST00000295956,ENST00000295956,ENST00000
ENST00000309988,ENST00000349807,ENST00000353004,ENST00000354078
ENST00000374518
ENST00000266000,ENST00000374542,ENST00000414083,ENST00000446403,ENST00000453407
ENST00000346193
ENST00000257017
ENST00000254260,ENST00000400226
ENST00000253435,ENST00000598265,ENST00000602198
ENST00000281815,ENST00000338347
ENST00000300113
ENST00000355221,ENST00000359530,ENST00000396774,ENST00000520188
ENST00000254260,ENST00000400226
ENST00000370742,ENST00000401034
ENST00000342960,ENST00000369338,ENST00000369339,ENST00000490598
ENST00000342960,ENST00000490598
ENST00000397910
ENST00000372880
ENST00000301607,ENST00000586740
ENST00000267540,ENST00000555447
ENST00000378491
ENST00000397126
ENST00000417435
ENST00000343905
ENST00000409757,ENST00000444368,ENST00000448507
ENST00000293925,ENST00000397412,ENST00000436138
ENST00000239938
ENST00000311519,ENST00000333478,ENST00000421160,ENST00000447444,ENST00000541401
ENST00000266079,ENST00000535781

ENST00000361193,ENST00000411502,ENST00000546047,ENST00000571215,ENST00000572725
ENST00000374694
ENST00000294724,ENST00000361302,ENST00000361522,ENST00000361915,ENST00000370161,ENST00000[illegible]
ENST00000303319,ENST00000422782,ENST00000428879
ENST00000336119,ENST00000348069,ENST00000366496,ENST00000366497,ENST00000391827,ENST00000[illegible]
ENST00000300619,ENST00000397082
ENST00000367796,ENST00000367797
ENST00000388996,ENST00000519445,ENST00000521134
ENST00000246006
ENST00000308158,ENST00000323594,ENST00000510913
ENST00000340027,ENST00000376071,ENST00000376072,ENST00000376073
ENST00000269844,ENST00000398548,ENST00000422911,ENST00000433067,ENST00000447016,ENST00000[illegible]
ENST00000326637,ENST00000370891,ENST00000557284
ENST00000306151,ENST00000379439
ENST00000338608,ENST00000432063,ENST00000535545,ENST00000542951
ENST00000361320
ENST00000367662
ENST00000269305,ENST00000359597,ENST00000413465,ENST00000420246,ENST00000445888,ENST00000[illegible]
ENST00000281815,ENST00000338347
ENST00000306151,ENST00000379439
ENST00000334197,ENST00000452676,ENST00000601469
ENST00000367549
ENST00000306151,ENST00000379439
ENST00000225550
ENST00000316924,ENST00000394066,ENST00000394067,ENST00000417856,ENST00000421552
ENST00000256339,ENST00000393151,ENST00000553484,ENST00000555664
ENST00000262189,ENST00000355193
ENST00000289292,ENST00000376020,ENST00000460112
ENST00000325945,ENST00000361035
ENST00000333229
ENST00000354476,ENST00000390003,ENST00000402934,ENST00000404525,ENST00000404816,ENST00000[illegible]
ENST00000356929
ENST00000282869
ENST00000357258,ENST00000393785,ENST00000462705,ENST00000464560,ENST00000471418,ENST00000[illegible]
ENST00000359798,ENST00000487863
ENST00000342960,ENST00000369338,ENST00000490598
ENST00000158526,ENST00000393600
ENST00000375394,ENST00000544581
ENST00000313735
ENST00000321725
ENST00000322313,ENST00000393006,ENST00000408998,ENST00000409658,ENST00000436787
ENST00000357362,ENST00000399222,ENST00000399232,ENST00000426342,ENST00000439988
ENST00000536728
ENST00000456556
ENST00000336128,ENST00000366197
ENST00000001008
ENST00000257766,ENST00000403245

ENST00000338977,ENST00000511833
ENST00000311637,ENST00000338821,ENST00000402822
ENST00000360131
ENST00000316073
ENST00000354258,ENST00000425148
ENST00000260818
ENST00000317702,ENST00000590103
ENST00000377564
ENST00000296870
ENST00000300870
ENST00000256339,ENST00000393151,ENST00000553484,ENST00000555664
ENST00000262441,ENST00000574745
ENST00000354959,ENST00000594012,ENST00000595461,ENST00000598381
ENST00000331035,ENST00000381469,ENST00000432757
ENST00000371365
ENST00000419318,ENST00000430385
ENST00000377626,ENST00000602553
ENST00000429747,ENST00000502818,ENST00000511604,ENST00000512055,ENST00000512332
ENST00000278460,ENST00000378615,ENST00000378618,ENST00000395460,ENST00000526827,ENST00000[illegible]
ENST00000367462,ENST00000534846
ENST00000245907
ENST00000296869,ENST00000357096,ENST00000379240,ENST00000379244,ENST00000379246,ENST00000[illegible]
ENST00000379370
ENST00000300870
ENST00000276344,ENST00000360243,ENST00000370335,ENST00000370337,ENST00000370340,ENST00000[illegible]
ENST00000379802,ENST00000418664
ENST00000317178
ENST00000268489,ENST00000397992
ENST00000391413
ENST00000311048
ENST00000328178,ENST00000345030,ENST00000596534,ENST00000601023
ENST00000490820,ENST00000525639,ENST00000527447
ENST00000600228
ENST00000361937,ENST00000401393,ENST00000558357
ENST00000303592
ENST00000293879,ENST00000549091,ENST00000552728
ENST00000366691
ENST00000328178,ENST00000328178,ENST00000345030,ENST00000345030,ENST00000596534,ENST00000[illegible]
ENST00000312908,ENST00000531805,ENST00000532828
ENST00000295025,ENST00000394479
ENST00000333891,ENST00000423517
ENST00000523833
ENST00000372588
ENST00000253693
ENST00000312513,ENST00000580762,ENST00000582535
ENST00000322644
ENST00000283645,ENST00000453949

ENST00000261588
ENST00000221355,ENST00000434248
ENST00000331925,ENST00000573283,ENST00000575087,ENST00000575842,ENST00000576544
ENST00000378024
ENST00000418100
ENST00000245838,ENST00000355725,ENST00000491737
ENST00000311048,ENST00000344930,ENST00000395391,ENST00000423627
ENST00000307355,ENST00000359735,ENST00000397136,ENST00000437743,ENST00000440155
ENST00000300619,ENST00000300619,ENST00000300619,ENST00000305570,ENST00000343776,ENST00000[illegible]
ENST00000341319,ENST00000468734
ENST00000295897,ENST00000401494,ENST00000415165,ENST00000441319,ENST00000509063
ENST00000420323
ENST00000348028,ENST00000349350,ENST00000421100,ENST00000427578,ENST00000443370,ENST00000[illegible]
ENST00000291707,ENST00000598614
ENST00000456556
ENST00000314686,ENST00000429348,ENST00000432264,ENST00000447116,ENST00000456778,ENST00000[illegible]
ENST00000371068,ENST00000433625,ENST00000480623,ENST00000538167
ENST00000338977,ENST00000511833
ENST00000395385,ENST00000402377
ENST00000376767
ENST00000396073
ENST00000262327,ENST00000378526,ENST00000585941,ENST00000592690
ENST00000348028,ENST00000349350,ENST00000421100,ENST00000427578,ENST00000443370,ENST00000[illegible]
ENST00000265354,ENST00000457278
ENST00000391413
ENST00000598347
ENST00000343776,ENST00000397097
ENST00000282869,ENST00000395385,ENST00000402377
ENST00000343776,ENST00000397097
ENST00000368850,ENST00000368851
ENST00000257622,ENST00000358357,ENST00000395270,ENST00000434423,ENST00000446813
ENST00000378024
ENST00000334493,ENST00000469830,ENST00000477459
ENST00000376228,ENST00000376237,ENST00000383329
ENST00000528685
ENST00000343984,ENST00000354228,ENST00000354416,ENST00000360156,ENST00000394610,ENST00000[illegible]
ENST00000427495,ENST00000442594,ENST00000536534
ENST00000356929,ENST00000397126,ENST00000397126
ENST00000344930,ENST00000395391,ENST00000423627
ENST00000299927,ENST00000559607,ENST00000560079
ENST00000344930,ENST00000395391,ENST00000423627
ENST00000356929,ENST00000356929
ENST00000300619,ENST00000397082
ENST00000328178,ENST00000345030,ENST00000596534,ENST00000601023
ENST00000305570,ENST00000328178,ENST00000344930,ENST00000345030,ENST00000356929,ENST00000[illegible]
ENST00000300619,ENST00000397082
ENST00000397126

ENST00000374694
ENST00000303319,ENST00000303319,ENST00000303319,ENST00000422782,ENST00000422782,ENST00000[illegible]
ENST00000303319,ENST00000303319,ENST00000303319,ENST00000422782,ENST00000422782,ENST00000[illegible]
ENST00000303319,ENST00000303319,ENST00000303319,ENST00000422782,ENST00000422782,ENST00000[illegible]
ENST00000397126
ENST00000309988,ENST00000309988,ENST00000309988,ENST00000309988,ENST00000349807,ENST00000[illegible]
ENST00000311519,ENST00000311519,ENST00000311519,ENST00000311519,ENST00000311519,ENST00000[illegible]
ENST00000300870,ENST00000300870
ENST00000377564
ENST00000246006
ENST00000368850,ENST00000368850,ENST00000368851,ENST00000368851
ENST00000293925,ENST00000293925,ENST00000293925,ENST00000397412,ENST00000397412,ENST00000[illegible]
ENST00000356929
ENST00000372880
ENST00000253693
ENST00000366691
ENST00000256339,ENST00000256339,ENST00000256339,ENST00000256339,ENST00000393151,ENST00000[illegible]
ENST00000322313,ENST00000322313,ENST00000322313,ENST00000322313,ENST00000393006,ENST00000[illegible]
ENST00000374518
ENST00000312513,ENST00000312513,ENST00000312513,ENST00000580762,ENST00000580762,ENST00000[illegible]
ENST00000312513,ENST00000312513,ENST00000312513,ENST00000580762,ENST00000580762,ENST00000[illegible]
ENST00000312513,ENST00000312513,ENST00000312513,ENST00000580762,ENST00000580762,ENST00000[illegible]
ENST00000388996,ENST00000388996,ENST00000388996,ENST00000519445,ENST00000519445,ENST00000[illegible]
ENST00000282869
ENST00000490820,ENST00000490820,ENST00000490820,ENST00000525639,ENST00000525639,ENST00000[illegible]
ENST00000389131,ENST00000389131,ENST00000389131,ENST00000389131,ENST00000389131,ENST00000[illegible]
ENST00000528685
ENST00000340027,ENST00000340027,ENST00000340027,ENST00000340027,ENST00000376071,ENST00000[illegible]
ENST00000333229
ENST00000294724,ENST00000294724,ENST00000294724,ENST00000294724,ENST00000294724,ENST00000[illegible]
ENST00000296870
ENST00000338977,ENST00000338977,ENST00000511833,ENST00000511833
ENST00000371068,ENST00000371068,ENST00000371068,ENST00000371068,ENST00000433625,ENST00000[illegible]
ENST00000265354,ENST00000265354,ENST00000457278,ENST00000457278
ENST00000281815,ENST00000281815,ENST00000338347,ENST00000338347
ENST00000334197,ENST00000334197,ENST00000334197,ENST00000452676,ENST00000452676,ENST00000[illegible]
ENST00000300113
ENST00000343905
ENST00000291707,ENST00000291707,ENST00000598614,ENST00000598614
ENST00000377626,ENST00000377626,ENST00000602553,ENST00000602553
ENST00000354959,ENST00000354959,ENST00000354959,ENST00000354959,ENST00000594012,ENST00000[illegible]
ENST00000361193,ENST00000361193,ENST00000361193,ENST00000361193,ENST00000361193,ENST00000[illegible]
ENST00000283645,ENST00000283645,ENST00000453949,ENST00000453949
ENST00000487078
ENST00000328178,ENST00000328178,ENST00000328178,ENST00000328178,ENST00000345030,ENST00000[illegible]
ENST00000395385,ENST00000395385,ENST00000402377,ENST00000402377
ENST00000344930,ENST00000344930,ENST00000344930,ENST00000395391,ENST00000395391,ENST00000[illegible]

ENST00000266079,ENST00000266079,ENST00000535781,ENST00000535781
ENST00000296869,ENST00000296869,ENST00000296869,ENST00000296869,ENST00000296869,ENST00000
ENST00000303592
ENST00000300619,ENST00000300619,ENST00000397082,ENST00000397082
ENST00000375394,ENST00000375394,ENST00000544581,ENST00000544581
ENST00000239938
ENST00000397910
ENST00000313735
ENST00000326637,ENST00000326637,ENST00000326637,ENST00000370891,ENST00000370891,ENST00000
ENST00000001008
ENST00000254260,ENST00000254260,ENST00000254260,ENST00000254260,ENST00000400226,ENST00000
ENST00000344930,ENST00000344930,ENST00000344930,ENST00000395391,ENST00000395391,ENST00000
ENST00000253435,ENST00000253435,ENST00000253435,ENST00000598265,ENST00000598265,ENST00000
ENST00000367462,ENST00000367462,ENST00000534846,ENST00000534846
ENST00000361937,ENST00000361937,ENST00000361937,ENST00000401393,ENST00000401393,ENST00000
ENST00000257017
ENST00000306151,ENST00000306151,ENST00000379439,ENST00000379439
ENST00000356929
ENST00000370742,ENST00000370742,ENST00000401034,ENST00000401034
ENST00000277549,ENST00000277549,ENST00000277549,ENST00000277549,ENST00000277549,ENST00000
ENST00000308158,ENST00000308158,ENST00000308158,ENST00000323594,ENST00000323594,ENST00000
ENST00000343776,ENST00000343776,ENST00000397097,ENST00000397097
ENST00000311048
ENST00000245907
ENST00000419318,ENST00000419318,ENST00000430385,ENST00000430385
ENST00000536728
ENST00000427495,ENST00000427495,ENST00000427495,ENST00000442594,ENST00000442594,ENST00000
ENST00000361320
ENST00000333891,ENST00000333891,ENST00000423517,ENST00000423517
ENST00000293879,ENST00000293879,ENST00000293879,ENST00000549091,ENST00000549091,ENST00000
ENST00000301607,ENST00000301607,ENST00000586740,ENST00000586740
ENST00000342960,ENST00000342960,ENST00000342960,ENST00000342960,ENST00000369338,ENST00000
ENST00000331035,ENST00000331035,ENST00000331035,ENST00000381469,ENST00000381469,ENST00000
ENST00000321725
ENST00000300619,ENST00000300619,ENST00000300619,ENST00000300619,ENST00000354959,ENST00000
ENST00000299927,ENST00000299927,ENST00000299927,ENST00000559607,ENST00000559607,ENST00000
ENST00000257622,ENST00000257622,ENST00000257622,ENST00000257622,ENST00000257622,ENST00000
ENST00000376228,ENST00000376228,ENST00000376228,ENST00000376237,ENST00000376237,ENST00000
ENST00000429747,ENST00000429747,ENST00000429747,ENST00000429747,ENST00000429747,ENST00000
ENST00000356929
ENST00000311637,ENST00000311637,ENST00000311637,ENST00000338821,ENST00000338821,ENST00000
ENST00000325945,ENST00000325945,ENST00000361035,ENST00000361035
ENST00000260818
ENST00000311048
ENST00000379802,ENST00000379802,ENST00000418664,ENST00000418664
ENST00000245838,ENST00000245838,ENST00000245838,ENST00000355725,ENST00000355725,ENST00000
ENST00000395385,ENST00000395385,ENST00000402377,ENST00000402377

ENST00000300619,ENST00000300619,ENST00000397082,ENST00000397082
ENST00000322644
ENST00000307355,ENST00000307355,ENST00000307355,ENST00000307355,ENST00000307355,ENST00000
ENST00000359798,ENST00000359798,ENST00000487863,ENST00000487863
ENST00000300619,ENST00000300619,ENST00000397082,ENST00000397082
ENST00000331925,ENST00000331925,ENST00000331925,ENST00000331925,ENST00000331925,ENST00000
ENST00000600228
ENST00000317702,ENST00000317702,ENST00000590103,ENST00000590103
ENST00000225550
ENST00000336119,ENST00000336119,ENST00000336119,ENST00000336119,ENST00000336119,ENST00000
ENST00000342960,ENST00000342960,ENST00000342960,ENST00000369338,ENST00000369338,ENST00000
ENST00000306151,ENST00000306151,ENST00000379439,ENST00000379439
ENST00000367796,ENST00000367796,ENST00000367797,ENST00000367797
ENST00000262189,ENST00000262189,ENST00000355193,ENST00000355193
ENST00000282869
ENST00000316924,ENST00000316924,ENST00000316924,ENST00000316924,ENST00000316924,ENST00000
ENST00000266000,ENST00000266000,ENST00000266000,ENST00000266000,ENST00000266000,ENST00000
ENST00000268489,ENST00000268489,ENST00000397992,ENST00000397992
ENST00000378491
ENST00000420323
ENST00000346193
ENST00000367549
ENST00000312908,ENST00000312908,ENST00000312908,ENST00000531805,ENST00000531805,ENST00000
ENST00000328178,ENST00000328178,ENST00000328178,ENST00000328178,ENST00000345030,ENST00000
ENST00000357362,ENST00000357362,ENST00000357362,ENST00000357362,ENST00000357362,ENST00000
ENST00000372588
ENST00000343776,ENST00000343776,ENST00000397097,ENST00000397097
ENST00000281815,ENST00000281815,ENST00000338347,ENST00000338347
ENST00000289292,ENST00000289292,ENST00000289292,ENST00000376020,ENST00000376020,ENST00000
ENST00000418100
ENST00000355221,ENST00000355221,ENST00000355221,ENST00000355221,ENST00000359530,ENST00000
ENST00000338608,ENST00000338608,ENST00000338608,ENST00000338608,ENST00000432063,ENST00000
ENST00000357258,ENST00000357258,ENST00000357258,ENST00000357258,ENST00000357258,ENST00000
ENST00000456556,ENST00000456556
ENST00000354258,ENST00000354258,ENST00000425148,ENST00000425148
ENST00000276344,ENST00000276344,ENST00000276344,ENST00000276344,ENST00000276344,ENST00000
ENST00000269844,ENST00000269844,ENST00000269844,ENST00000269844,ENST00000269844,ENST00000
ENST00000341319,ENST00000341319,ENST00000468734,ENST00000468734
ENST00000356929
ENST00000278460,ENST00000278460,ENST00000278460,ENST00000278460,ENST00000278460,ENST00000
ENST00000317178
ENST00000354476,ENST00000354476,ENST00000354476,ENST00000354476,ENST00000354476,ENST00000
ENST00000262441,ENST00000262441,ENST00000574745,ENST00000574745
ENST00000328178,ENST00000328178,ENST00000328178,ENST00000328178,ENST00000345030,ENST00000
ENST00000221355,ENST00000221355,ENST00000434248,ENST00000434248
ENST00000305570,ENST00000305570,ENST00000358296,ENST00000358296
ENST00000338977,ENST00000338977,ENST00000511833,ENST00000511833

ENST00000397126
ENST00000397121
ENST00000314686,ENST00000314686,ENST00000314686,ENST00000314686,ENST00000314686,ENST00000
ENST00000295897,ENST00000295897,ENST00000295897,ENST00000295897,ENST00000295897,ENST00000
ENST00000158526,ENST00000158526,ENST00000393600,ENST00000393600
ENST00000262327,ENST00000262327,ENST00000262327,ENST00000262327,ENST00000378526,ENST00000
ENST00000367662
ENST00000379370
ENST00000306151,ENST00000306151,ENST00000379439,ENST00000379439
ENST00000336128,ENST00000336128,ENST00000366197,ENST00000366197
ENST00000261588
ENST00000378024
ENST00000295025,ENST00000295025,ENST00000394479,ENST00000394479
ENST00000376767
ENST00000409757,ENST00000409757,ENST00000448507,ENST00000448507
ENST00000378024
ENST00000334493,ENST00000334493,ENST00000469830,ENST00000469830
ENST00000269305,ENST00000269305,ENST00000269305,ENST00000269305,ENST00000269305,ENST00000
ENST00000267540,ENST00000267540,ENST00000555447,ENST00000555447
ENST00000342960,ENST00000342960,ENST00000490598,ENST00000490598
ENST00000417435
ENST00000316073
ENST00000257766,ENST00000257766,ENST00000403245,ENST00000403245
ENST00000596143,ENST00000596143,ENST00000600435,ENST00000600435
ENST00000343984,ENST00000343984,ENST00000343984,ENST00000343984,ENST00000343984,ENST00000
ENST00000391413
ENST00000256339,ENST00000256339,ENST00000256339,ENST00000256339,ENST00000393151,ENST00000
ENST00000396073
ENST00000348028,ENST00000348028,ENST00000348028,ENST00000348028,ENST00000348028,ENST00000
ENST00000391413
ENST00000348028,ENST00000348028,ENST00000348028,ENST00000348028,ENST00000348028,ENST00000
ENST00000598347
ENST00000309988,ENST00000349807,ENST00000353004,ENST00000354078
ENST00000281815,ENST00000338347
ENST00000281815,ENST00000338347
ENST00000342960,ENST00000490598
ENST00000352853,ENST00000359524,ENST00000371432,ENST00000371436,ENST00000371438,ENST00000
ENST00000297954,ENST00000349097,ENST00000395477,ENST00000427277
ENST00000265148,ENST00000380026
ENST00000372880
ENST00000332990,ENST00000415895
ENST00000267540,ENST00000555447
ENST00000417435
ENST00000302124
ENST00000304434,ENST00000370918,ENST00000541407,ENST00000542638
ENST00000293925,ENST00000397412,ENST00000436138
ENST00000374694

ENST00000294724,ENST00000361302,ENST00000361522,ENST00000361915,ENST00000370161,ENST00000[illegible]
ENST00000372129
ENST00000325241,ENST00000446747,ENST00000528372,ENST00000544249
ENST00000308158,ENST00000323594,ENST00000510913
ENST00000358511,ENST00000453409
ENST00000367662
ENST00000269305,ENST00000359597,ENST00000413465,ENST00000420246,ENST00000445888,ENST00000[illegible]
ENST00000281815,ENST00000338347
ENST00000264784,ENST00000309065,ENST00000506583,ENST00000513129
ENST00000265425,ENST00000360488,ENST00000377850
ENST00000342944,ENST00000357002
ENST00000262189,ENST00000355193
ENST00000289292,ENST00000376020,ENST00000460112
ENST00000325945,ENST00000361035
ENST00000333229
ENST00000304883
ENST00000390652,ENST00000585744,ENST00000588462
ENST00000409792
ENST00000321725
ENST00000356297,ENST00000522628,ENST00000543296
ENST00000326828,ENST00000348639,ENST00000559658
ENST00000220959,ENST00000520539,ENST00000521922
ENST00000346964,ENST00000357674,ENST00000450955
ENST00000336128,ENST00000366197
ENST00000328020,ENST00000379500,ENST00000533953
ENST00000262441,ENST00000574745
ENST00000367462,ENST00000534846
ENST00000359798,ENST00000487863
ENST00000246657,ENST00000579344
ENST00000296869,ENST00000357096,ENST00000379240,ENST00000379244,ENST00000379246,ENST00000[illegible]
ENST00000490820,ENST00000525639,ENST00000527447
ENST00000261862,ENST00000355327,ENST00000357769
ENST00000269081
ENST00000293879,ENST00000549091,ENST00000552728
ENST00000523833
ENST00000245838,ENST00000355725,ENST00000491737
ENST00000304514,ENST00000409886
ENST00000311048
ENST00000300619,ENST00000342944,ENST00000357002,ENST00000397082
ENST00000296792,ENST00000508491,ENST00000543251
ENST00000291707,ENST00000598614
ENST00000391413
ENST00000369202
ENST00000156825,ENST00000434436,ENST00000590550,ENST00000592012
ENST00000528685
ENST00000331710
ENST00000419039,ENST00000420871,ENST00000456556

ENST00000427495,ENST00000442594,ENST00000536534
ENST00000252172,ENST00000418404
ENST00000265023
ENST00000374694
ENST00000409792
ENST00000309988,ENST00000309988,ENST00000309988,ENST00000309988,ENST00000349807,ENST00000
ENST00000297954,ENST00000297954,ENST00000297954,ENST00000297954,ENST00000349097,ENST00000
ENST00000293925,ENST00000293925,ENST00000293925,ENST00000397412,ENST00000397412,ENST00000
ENST00000372880
ENST00000264784,ENST00000264784,ENST00000264784,ENST00000264784,ENST00000309065,ENST00000
ENST00000265425,ENST00000265425,ENST00000265425,ENST00000360488,ENST00000360488,ENST00000
ENST00000265425,ENST00000265425,ENST00000265425,ENST00000360488,ENST00000360488,ENST00000
ENST00000490820,ENST00000490820,ENST00000490820,ENST00000525639,ENST00000525639,ENST00000
ENST00000389131,ENST00000389131,ENST00000389131,ENST00000389131,ENST00000389131,ENST00000
ENST00000302124
ENST00000528685
ENST00000333229
ENST00000294724,ENST00000294724,ENST00000294724,ENST00000294724,ENST00000294724,ENST00000
ENST00000281815,ENST00000281815,ENST00000338347,ENST00000338347
ENST00000246657,ENST00000246657,ENST00000579344,ENST00000579344
ENST00000291707,ENST00000291707,ENST00000598614,ENST00000598614
ENST00000328020,ENST00000328020,ENST00000328020,ENST00000379500,ENST00000379500,ENST00000
ENST00000358511,ENST00000358511,ENST00000453409,ENST00000453409
ENST00000352853,ENST00000352853,ENST00000352853,ENST00000352853,ENST00000352853,ENST00000
ENST00000304883
ENST00000331710
ENST00000342944,ENST00000342944,ENST00000357002,ENST00000357002
ENST00000419039,ENST00000419039,ENST00000419039,ENST00000420871,ENST00000420871,ENST00000
ENST00000296869,ENST00000296869,ENST00000296869,ENST00000296869,ENST00000296869,ENST00000
ENST00000390652,ENST00000390652,ENST00000390652,ENST00000585744,ENST00000585744,ENST00000
ENST00000346964,ENST00000346964,ENST00000346964,ENST00000357674,ENST00000357674,ENST00000
ENST00000367462,ENST00000367462,ENST00000534846,ENST00000534846
ENST00000308158,ENST00000308158,ENST00000308158,ENST00000323594,ENST00000323594,ENST00000
ENST00000427495,ENST00000427495,ENST00000427495,ENST00000442594,ENST00000442594,ENST00000
ENST00000325241,ENST00000325241,ENST00000325241,ENST00000325241,ENST00000446747,ENST00000
ENST00000293879,ENST00000293879,ENST00000293879,ENST00000549091,ENST00000549091,ENST00000
ENST00000321725
ENST00000332990,ENST00000332990,ENST00000415895,ENST00000415895
ENST00000325945,ENST00000325945,ENST00000361035,ENST00000361035
ENST00000311048
ENST00000245838,ENST00000245838,ENST00000245838,ENST00000355725,ENST00000355725,ENST00000
ENST00000300619,ENST00000300619,ENST00000397082,ENST00000397082
ENST00000304434,ENST00000304434,ENST00000304434,ENST00000304434,ENST00000370918,ENST00000
ENST00000262189,ENST00000262189,ENST00000355193,ENST00000355193
ENST00000281815,ENST00000281815,ENST00000338347,ENST00000338347
ENST00000289292,ENST00000289292,ENST00000289292,ENST00000376020,ENST00000376020,ENST00000
ENST00000372129

ENST00000356297,ENST00000356297,ENST00000356297,ENST00000522628,ENST00000522628,ENST00000
ENST00000265148,ENST00000265148,ENST00000380026,ENST00000380026
ENST00000262441,ENST00000262441,ENST00000574745,ENST00000574745
ENST00000269081
ENST00000281815,ENST00000281815,ENST00000338347,ENST00000338347
ENST00000304514,ENST00000304514,ENST00000409886,ENST00000409886
ENST00000359798,ENST00000359798,ENST00000487863,ENST00000487863
ENST00000296792,ENST00000296792,ENST00000296792,ENST00000508491,ENST00000508491,ENST00000
ENST00000367662
ENST00000220959,ENST00000220959,ENST00000220959,ENST00000520539,ENST00000520539,ENST00000
ENST00000336128,ENST00000336128,ENST00000366197,ENST00000366197
ENST00000326828,ENST00000326828,ENST00000326828,ENST00000348639,ENST00000348639,ENST00000
ENST00000269305,ENST00000269305,ENST00000269305,ENST00000269305,ENST00000269305,ENST00000
ENST00000267540,ENST00000267540,ENST00000555447,ENST00000555447
ENST00000342960,ENST00000342960,ENST00000490598,ENST00000490598
ENST00000417435
ENST00000391413
ENST00000156825,ENST00000156825,ENST00000156825,ENST00000156825,ENST00000434436,ENST00000
ENST00000252172,ENST00000252172,ENST00000418404,ENST00000418404
ENST00000261862,ENST00000261862,ENST00000261862,ENST00000355327,ENST00000355327,ENST00000
ENST00000265023
ENST00000369202
ENST00000309988,ENST00000349807,ENST00000353004,ENST00000354078
ENST00000281815,ENST00000338347
ENST00000281815,ENST00000338347
ENST00000300113
ENST00000342960,ENST00000490598
ENST00000352853,ENST00000359524,ENST00000371432,ENST00000371436,ENST00000371438,ENST00000
ENST00000297954,ENST00000349097,ENST00000395477,ENST00000427277
ENST00000265148,ENST00000380026
ENST00000372880
ENST00000267540,ENST00000555447
ENST00000417435
ENST00000343905
ENST00000304434,ENST00000370918,ENST00000541407,ENST00000542638
ENST00000293925,ENST00000397412,ENST00000436138
ENST00000374694
ENST00000294724,ENST00000361302,ENST00000361522,ENST00000361915,ENST00000370161,ENST00000
ENST00000372129
ENST00000308158,ENST00000323594,ENST00000510913
ENST00000367662
ENST00000269305,ENST00000359597,ENST00000413465,ENST00000420246,ENST00000445888,ENST00000
ENST00000281815,ENST00000338347
ENST00000541274
ENST00000265425,ENST00000360488,ENST00000377850
ENST00000262189,ENST00000355193
ENST00000289292,ENST00000376020,ENST00000460112

ENST00000325945,ENST00000361035
ENST00000333229
ENST00000304883
ENST00000528685
ENST00000409792
ENST00000281815,ENST00000338347
ENST00000321725
ENST00000356297,ENST00000522628,ENST00000543296
ENST00000326828,ENST00000348639,ENST00000559658
ENST00000346964,ENST00000357674,ENST00000450955
ENST00000336128,ENST00000366197
ENST00000328020,ENST00000379500,ENST00000533953
ENST00000262441,ENST00000574745
ENST00000367462,ENST00000534846
ENST00000246657,ENST00000579344
ENST00000296869,ENST00000357096,ENST00000379240,ENST00000379244,ENST00000379246,ENST00000
ENST00000322139,ENST00000332647,ENST00000355813,ENST00000538690
ENST00000490820,ENST00000525639,ENST00000527447
ENST00000293879,ENST00000549091,ENST00000552728
ENST00000523833
ENST00000245838,ENST00000355725,ENST00000491737
ENST00000304514,ENST00000409886
ENST00000296792,ENST00000508491,ENST00000543251
ENST00000291707,ENST00000598614
ENST00000376767
ENST00000298292,ENST00000406043
ENST00000408903
ENST00000367482,ENST00000367483,ENST00000367485,ENST00000367486,ENST00000445192
ENST00000528685
ENST00000427495,ENST00000442594,ENST00000536534
ENST00000252172,ENST00000418404
ENST00000243286,ENST00000372627,ENST00000372628
ENST00000489525
ENST00000374694
ENST00000489525
ENST00000409792
ENST00000408903
ENST00000309988,ENST00000309988,ENST00000309988,ENST00000309988,ENST00000349807,ENST00000
ENST00000297954,ENST00000297954,ENST00000297954,ENST00000297954,ENST00000349097,ENST00000
ENST00000293925,ENST00000293925,ENST00000293925,ENST00000397412,ENST00000397412,ENST00000
ENST00000372880
ENST00000265425,ENST00000265425,ENST00000265425,ENST00000360488,ENST00000360488,ENST00000
ENST00000265425,ENST00000265425,ENST00000265425,ENST00000360488,ENST00000360488,ENST00000
ENST00000490820,ENST00000490820,ENST00000490820,ENST00000525639,ENST00000525639,ENST00000
ENST00000389131,ENST00000389131,ENST00000389131,ENST00000389131,ENST00000389131,ENST00000
ENST00000528685,ENST00000528685
ENST00000333229

ENST00000294724,ENST00000294724,ENST00000294724,ENST00000294724,ENST00000294724,ENST00000
ENST00000281815,ENST00000281815,ENST00000281815,ENST00000281815,ENST00000338347,ENST00000
ENST00000246657,ENST00000246657,ENST00000579344,ENST00000579344
ENST00000300113
ENST00000343905
ENST00000291707,ENST00000291707,ENST00000598614,ENST00000598614
ENST00000328020,ENST00000328020,ENST00000328020,ENST00000379500,ENST00000379500,ENST00000
ENST00000352853,ENST00000352853,ENST00000352853,ENST00000352853,ENST00000352853,ENST00000
ENST00000304883
ENST00000296869,ENST00000296869,ENST00000296869,ENST00000296869,ENST00000296869,ENST00000
ENST00000346964,ENST00000346964,ENST00000346964,ENST00000357674,ENST00000357674,ENST00000
ENST00000367462,ENST00000367462,ENST00000534846,ENST00000534846
ENST00000308158,ENST00000308158,ENST00000308158,ENST00000323594,ENST00000323594,ENST00000
ENST00000427495,ENST00000427495,ENST00000427495,ENST00000442594,ENST00000442594,ENST00000
ENST00000293879,ENST00000293879,ENST00000293879,ENST00000549091,ENST00000549091,ENST00000
ENST00000321725
ENST00000325945,ENST00000325945,ENST00000361035,ENST00000361035
ENST00000245838,ENST00000245838,ENST00000245838,ENST00000355725,ENST00000355725,ENST00000
ENST00000304434,ENST00000304434,ENST00000304434,ENST00000304434,ENST00000370918,ENST00000
ENST00000298292,ENST00000298292,ENST00000406043,ENST00000406043
ENST00000262189,ENST00000262189,ENST00000355193,ENST00000355193
ENST00000541274
ENST00000281815,ENST00000281815,ENST00000338347,ENST00000338347
ENST00000289292,ENST00000289292,ENST00000289292,ENST00000376020,ENST00000376020,ENST00000
ENST00000372129
ENST00000356297,ENST00000356297,ENST00000356297,ENST00000522628,ENST00000522628,ENST00000
ENST00000265148,ENST00000265148,ENST00000380026,ENST00000380026
ENST00000262441,ENST00000262441,ENST00000574745,ENST00000574745
ENST00000281815,ENST00000281815,ENST00000338347,ENST00000338347
ENST00000304514,ENST00000304514,ENST00000409886,ENST00000409886
ENST00000322139,ENST00000322139,ENST00000322139,ENST00000322139,ENST00000332647,ENST00000
ENST00000296792,ENST00000296792,ENST00000296792,ENST00000508491,ENST00000508491,ENST00000
ENST00000367662
ENST00000336128,ENST00000336128,ENST00000366197,ENST00000366197
ENST00000326828,ENST00000326828,ENST00000326828,ENST00000348639,ENST00000348639,ENST00000
ENST00000376767
ENST00000269305,ENST00000269305,ENST00000269305,ENST00000269305,ENST00000269305,ENST00000
ENST00000267540,ENST00000267540,ENST00000555447,ENST00000555447
ENST00000342960,ENST00000342960,ENST00000490598,ENST00000490598
ENST00000417435
ENST00000252172,ENST00000252172,ENST00000418404,ENST00000418404
ENST00000367482,ENST00000367482,ENST00000367482,ENST00000367482,ENST00000367482,ENST00000
ENST00000243286,ENST00000243286,ENST00000243286,ENST00000372627,ENST00000372627,ENST00000
ENST00000309988,ENST00000349807,ENST00000353004,ENST00000354078
ENST00000313135
ENST00000300113
ENST00000352853,ENST00000359524,ENST00000371432,ENST00000371436,ENST00000371438,ENST00000

ENST00000372880
ENST00000332990,ENST00000415895
ENST00000330891,ENST00000488961,ENST00000553096
ENST00000267540,ENST00000555447
ENST00000370616,ENST00000370621,ENST00000503581
ENST00000417435
ENST00000343905
ENST00000304434,ENST00000370918,ENST00000541407,ENST00000542638
ENST00000293925,ENST00000397412,ENST00000436138
ENST00000410061
ENST00000375379,ENST00000375383,ENST00000375401,ENST00000404049,ENST00000452825
ENST00000160298,ENST00000446248
ENST00000374694
ENST00000294724,ENST00000361302,ENST00000361522,ENST00000361915,ENST00000370161,ENST00000
ENST00000277549,ENST00000277551,ENST00000371355,ENST00000371357,ENST00000371363,ENST00000
ENST00000305866,ENST00000535481,ENST00000544910
ENST00000308158,ENST00000323594,ENST00000510913
ENST00000367662
ENST00000269305,ENST00000359597,ENST00000413465,ENST00000420246,ENST00000445888,ENST00000
ENST00000056217
ENST00000299194,ENST00000535041,ENST00000543818
ENST00000262189,ENST00000355193
ENST00000289292,ENST00000376020,ENST00000460112
ENST00000325945,ENST00000361035
ENST00000333229
ENST00000427522,ENST00000467148
ENST00000287647,ENST00000383806,ENST00000383807,ENST00000419585
ENST00000252034,ENST00000320399,ENST00000320492,ENST00000357036,ENST00000358929,ENST00000
ENST00000281513,ENST00000423602,ENST00000433283,ENST00000441750
ENST00000334933,ENST00000348806,ENST00000360135,ENST00000360301,ENST00000381879,ENST00000
ENST00000321725
ENST00000356297,ENST00000522628,ENST00000543296
ENST00000294543,ENST00000375122,ENST00000375127
ENST00000536728
ENST00000378347
ENST00000346964,ENST00000357674,ENST00000450955
ENST00000336128,ENST00000366197
ENST00000258526
ENST00000338110,ENST00000395240,ENST00000395248,ENST00000412304,ENST00000563060,ENST00000
ENST00000542129
ENST00000262441,ENST00000574745
ENST00000381401
ENST00000538322
ENST00000367462,ENST00000534846
ENST00000349721,ENST00000357248,ENST00000382194,ENST00000382203
ENST00000367500,ENST00000367501
ENST00000296869,ENST00000357096,ENST00000379240,ENST00000379244,ENST00000379246,ENST00000

ENST00000490820,ENST00000525639,ENST00000527447
ENST00000296370
ENST00000293879,ENST00000549091,ENST00000552728
ENST00000523833
ENST00000395542,ENST00000431948
ENST00000245838,ENST00000355725,ENST00000491737
ENST00000358984
ENST00000317721,ENST00000409261
ENST00000272831,ENST00000389175,ENST00000409270,ENST00000448409
ENST00000427495,ENST00000442594,ENST00000536534
ENST00000252172,ENST00000418404
ENST00000358334,ENST00000359263,ENST00000371489,ENST00000371501,ENST00000371502
ENST00000374694
ENST00000313135
ENST00000309988,ENST00000309988,ENST00000309988,ENST00000309988,ENST00000349807,ENST00000
ENST00000293925,ENST00000293925,ENST00000293925,ENST00000397412,ENST00000397412,ENST00000
ENST00000372880
ENST00000395542,ENST00000395542,ENST00000431948,ENST00000431948
ENST00000490820,ENST00000490820,ENST00000490820,ENST00000525639,ENST00000525639,ENST00000
ENST00000252034,ENST00000252034,ENST00000252034,ENST00000252034,ENST00000252034,ENST00000
ENST00000389131,ENST00000389131,ENST00000389131,ENST00000389131,ENST00000389131,ENST00000
ENST00000281513,ENST00000281513,ENST00000281513,ENST00000281513,ENST00000423602,ENST00000
ENST00000333229
ENST00000294724,ENST00000294724,ENST00000294724,ENST00000294724,ENST00000294724,ENST00000
ENST00000338110,ENST00000395240,ENST00000395248,ENST00000412304,ENST00000563060,ENST00000
ENST00000300113
ENST00000343905
ENST00000294543,ENST00000294543,ENST00000294543,ENST00000375122,ENST00000375122,ENST00000
ENST00000352853,ENST00000352853,ENST00000352853,ENST00000352853,ENST00000352853,ENST00000
ENST00000427522,ENST00000427522,ENST00000467148,ENST00000467148
ENST00000299194,ENST00000299194,ENST00000299194,ENST00000535041,ENST00000535041,ENST00000
ENST00000296869,ENST00000296869,ENST00000296869,ENST00000296869,ENST00000296869,ENST00000
ENST00000258526
ENST00000346964,ENST00000346964,ENST00000346964,ENST00000357674,ENST00000357674,ENST00000
ENST00000367462,ENST00000367462,ENST00000534846,ENST00000534846
ENST00000308158,ENST00000308158,ENST00000308158,ENST00000323594,ENST00000323594,ENST00000
ENST00000536728
ENST00000427495,ENST00000427495,ENST00000427495,ENST00000442594,ENST00000442594,ENST00000
ENST00000367500,ENST00000367500,ENST00000367501,ENST00000367501
ENST00000160298,ENST00000160298,ENST00000446248,ENST00000446248
ENST00000542129
ENST00000056217
ENST00000378347
ENST00000293879,ENST00000293879,ENST00000293879,ENST00000549091,ENST00000549091,ENST00000
ENST00000321725
ENST00000370616,ENST00000370616,ENST00000370616,ENST00000370621,ENST00000370621,ENST00000
ENST00000538322

ENST00000332990,ENST00000332990,ENST00000415895,ENST00000415895
ENST00000358334,ENST00000358334,ENST00000358334,ENST00000358334,ENST00000358334,ENST000003
ENST00000325945,ENST00000325945,ENST00000361035,ENST00000361035
ENST00000245838,ENST00000245838,ENST00000245838,ENST00000355725,ENST00000355725,ENST000003
ENST00000330891,ENST00000330891,ENST00000330891,ENST00000488961,ENST00000488961,ENST000004
ENST00000304434,ENST00000304434,ENST00000304434,ENST00000304434,ENST00000370918,ENST000003
ENST00000381401
ENST00000410061
ENST00000262189,ENST00000262189,ENST00000355193,ENST00000355193
ENST00000334933,ENST00000334933,ENST00000334933,ENST00000334933,ENST00000334933,ENST000003
ENST00000287647,ENST00000287647,ENST00000287647,ENST00000287647,ENST00000383806,ENST000003
ENST00000289292,ENST00000289292,ENST00000289292,ENST00000376020,ENST00000376020,ENST000003
ENST00000305866,ENST00000305866,ENST00000305866,ENST00000535481,ENST00000535481,ENST000005
ENST00000356297,ENST00000356297,ENST00000356297,ENST00000522628,ENST00000522628,ENST000005
ENST00000277549,ENST00000277549,ENST00000277549,ENST00000277549,ENST00000277549,ENST000003
ENST00000262441,ENST00000262441,ENST00000574745,ENST00000574745
ENST00000375379,ENST00000375379,ENST00000375379,ENST00000375379,ENST00000375379,ENST000003
ENST00000296370
ENST00000367662
ENST00000336128,ENST00000336128,ENST00000366197,ENST00000366197
ENST00000349721,ENST00000349721,ENST00000349721,ENST00000349721,ENST00000357248,ENST000003
ENST00000269305,ENST00000269305,ENST00000269305,ENST00000269305,ENST00000269305,ENST000003
ENST00000267540,ENST00000267540,ENST00000555447,ENST00000555447
ENST00000417435
ENST00000358984
ENST00000317721,ENST00000317721,ENST00000409261,ENST00000409261
ENST00000252172,ENST00000252172,ENST00000418404,ENST00000418404
ENST00000272831,ENST00000272831,ENST00000272831,ENST00000272831,ENST00000389175,ENST000003
ENST00000379359
ENST00000441802
ENST00000247829,ENST00000393330,ENST00000546561,ENST00000552128
ENST00000261244,ENST00000354386,ENST00000423743,ENST00000554463,ENST00000555203,ENST000005
ENST00000317147,ENST00000441024,ENST00000567188,ENST00000569240
ENST00000216807,ENST00000543183
ENST00000216277,ENST00000392990,ENST00000555626
ENST00000378472,ENST00000407775,ENST00000517361,ENST00000520492
ENST00000266497,ENST00000433979,ENST00000538779
ENST00000294304,ENST00000529993
ENST00000378549,ENST00000378565
ENST00000382869,ENST00000382871,ENST00000382874,ENST00000382881
ENST00000523130,ENST00000524189
ENST00000261693,ENST00000339570,ENST00000376788,ENST00000415380,ENST00000540495,ENST000005
ENST00000247829,ENST00000393330,ENST00000546561,ENST00000552128
ENST00000327475,ENST00000359357,ENST00000441566,ENST00000449981
ENST00000226209,ENST00000583535
ENST00000388726,ENST00000392650,ENST00000424778
ENST00000452400

ENST00000356972
ENST00000409923,ENST00000432176
ENST00000523130,ENST00000523130,ENST00000524189,ENST00000524189
ENST00000378549,ENST00000378549,ENST00000378565,ENST00000378565
ENST00000261693,ENST00000261693,ENST00000261693,ENST00000261693,ENST00000261693,ENST00000
ENST00000216277,ENST00000216277,ENST00000216277,ENST00000392990,ENST00000392990,ENST00000
ENST00000441802
ENST00000317147,ENST00000317147,ENST00000317147,ENST00000317147,ENST00000441024,ENST00000
ENST00000327475,ENST00000327475,ENST00000327475,ENST00000327475,ENST00000359357,ENST00000
ENST00000409923,ENST00000409923,ENST00000432176,ENST00000432176
ENST00000261244,ENST00000261244,ENST00000261244,ENST00000261244,ENST00000261244,ENST00000
ENST00000379359
ENST00000266497,ENST00000266497,ENST00000266497,ENST00000433979,ENST00000433979,ENST00000
ENST00000294304,ENST00000294304,ENST00000529993,ENST00000529993
ENST00000247829,ENST00000247829,ENST00000247829,ENST00000247829,ENST00000247829,ENST00000
ENST00000452400
ENST00000356972
ENST00000226209,ENST00000226209,ENST00000583535,ENST00000583535
ENST00000382869,ENST00000382869,ENST00000382869,ENST00000382869,ENST00000382871,ENST00000
ENST00000216807,ENST00000216807,ENST00000543183,ENST00000543183
ENST00000378472,ENST00000378472,ENST00000378472,ENST00000378472,ENST00000407775,ENST00000
ENST00000388726,ENST00000388726,ENST00000388726,ENST00000392650,ENST00000392650,ENST00000
ENST00000369451,ENST00000435811,ENST00000454853
ENST00000381566,ENST00000381567,ENST00000400841
ENST00000296499,ENST00000433996
ENST00000329245,ENST00000519139,ENST00000519148,ENST00000519591,ENST00000521193,ENST00000
ENST00000379423
ENST00000343141,ENST00000356436,ENST00000379300,ENST00000379307,ENST00000450347,ENST00000
ENST00000366920,ENST00000392142,ENST00000544041
ENST00000343810,ENST00000357123,ENST00000546244,ENST00000548646,ENST00000550165,ENST00000
ENST00000250156
ENST00000456556
ENST00000316459,ENST00000373510
ENST00000456556
ENST00000357570,ENST00000358338,ENST00000360108,ENST00000367567,ENST00000367570,ENST00000
ENST00000205214,ENST00000434343,ENST00000513376
ENST00000329153
ENST00000372984,ENST00000432648,ENST00000446189
ENST00000350110,ENST00000446702,ENST00000539053
ENST00000456556
ENST00000343537,ENST00000391878
ENST00000500254,ENST00000545626,ENST00000546254
ENST00000427401
ENST00000398587,ENST00000518882
ENST00000343810,ENST00000343810,ENST00000343810,ENST00000343810,ENST00000343810,ENST00000
ENST00000366920,ENST00000366920,ENST00000366920,ENST00000392142,ENST00000392142,ENST00000
ENST00000316459,ENST00000316459,ENST00000373510,ENST00000373510

ENST00000369451,ENST00000369451,ENST00000369451,ENST00000435811,ENST00000435811,ENST00000[illegible]
ENST00000205214,ENST00000205214,ENST00000205214,ENST00000434343,ENST00000434343,ENST00000[illegible]
ENST00000250156
ENST00000343141,ENST00000343141,ENST00000343141,ENST00000343141,ENST00000343141,ENST00000[illegible]
ENST00000296499,ENST00000296499,ENST00000433996,ENST00000433996
ENST00000456556,ENST00000456556,ENST00000456556
ENST00000329153
ENST00000379423
ENST00000381566,ENST00000381566,ENST00000381566,ENST00000381567,ENST00000381567,ENST00000[illegible]
ENST00000372984,ENST00000372984,ENST00000372984,ENST00000432648,ENST00000432648,ENST00000[illegible]
ENST00000456556,ENST00000456556
ENST00000427401
ENST00000350110,ENST00000350110,ENST00000350110,ENST00000446702,ENST00000446702,ENST00000[illegible]
ENST00000329245,ENST00000329245,ENST00000329245,ENST00000329245,ENST00000329245,ENST00000[illegible]
ENST00000398587,ENST00000398587,ENST00000518882,ENST00000518882
ENST00000343537,ENST00000343537,ENST00000391878,ENST00000391878
ENST00000357570,ENST00000357570,ENST00000357570,ENST00000357570,ENST00000357570,ENST00000[illegible]
ENST00000456556
ENST00000500254,ENST00000500254,ENST00000500254,ENST00000545626,ENST00000545626,ENST00000[illegible]
ENST00000310998,ENST00000375921,ENST00000375922,ENST00000375934,ENST00000375938,ENST00000[illegible]
ENST00000359947,ENST00000372413,ENST00000372414,ENST00000422171,ENST00000438120
ENST00000262300,ENST00000431515,ENST00000440027,ENST00000572059,ENST00000572619,ENST00000[illegible]
ENST00000472091,ENST00000542862
ENST00000312358
ENST00000277817,ENST00000373700,ENST00000395198,ENST00000412272
ENST00000393079,ENST00000446465
ENST00000321871,ENST00000393130,ENST00000510183,ENST00000511555,ENST00000512137,ENST00000[illegible]
ENST00000408907
ENST00000259365,ENST00000375175,ENST00000395211
ENST00000326197,ENST00000337495,ENST00000397174,ENST00000402904
ENST00000251472
ENST00000529308
ENST00000525985
ENST00000251772,ENST00000393409
ENST00000225916
ENST00000246957,ENST00000538171,ENST00000575671,ENST00000576335
ENST00000366956
ENST00000253799,ENST00000452774
ENST00000369768
ENST00000290178,ENST00000331923
ENST00000355235,ENST00000356834
ENST00000257622,ENST00000358357,ENST00000395270,ENST00000434423,ENST00000446813
ENST00000304141
ENST00000231368,ENST00000395770
ENST00000308284
ENST00000493964
ENST00000218507

ENST00000339994,ENST00000528731
ENST00000371744,ENST00000371752,ENST00000371754,ENST00000396105,ENST00000455070
ENST00000318967,ENST00000435409,ENST00000456435,ENST00000512605
ENST00000336640,ENST00000356826,ENST00000394409,ENST00000394410,ENST00000394411,ENST00000
ENST00000321919,ENST00000381983,ENST00000436648,ENST00000591451
ENST00000300589
ENST00000361897,ENST00000431696,ENST00000464284,ENST00000493151,ENST00000530878
ENST00000433197
ENST00000344096,ENST00000393555
ENST00000292199,ENST00000409109,ENST00000409265,ENST00000409991,ENST00000525863
ENST00000296859,ENST00000307984,ENST00000308008,ENST00000507093,ENST00000509018,ENST00000
ENST00000226207
ENST00000335350,ENST00000373192
ENST00000344626,ENST00000358026,ENST00000413806,ENST00000429416,ENST00000444061,ENST00000
ENST00000341919,ENST00000346156,ENST00000395111,ENST00000443272,ENST00000586919,ENST00000
ENST00000172853,ENST00000397345,ENST00000409198,ENST00000413693,ENST00000427231,ENST00000
ENST00000334634,ENST00000368382,ENST00000368383,ENST00000545012
ENST00000327097,ENST00000541757
ENST00000264111,ENST00000397081,ENST00000397085,ENST00000397087,ENST00000535187,ENST00000
ENST00000273739,ENST00000503823,ENST00000503837,ENST00000504154
ENST00000361003,ENST00000389707,ENST00000389708,ENST00000396441,ENST00000403290,ENST00000
ENST00000262715,ENST00000555727,ENST00000556935
ENST00000314531
ENST00000420713
ENST00000375244,ENST00000375247
ENST00000360215,ENST00000373853
ENST00000538210,ENST00000606080
ENST00000253952,ENST00000326266,ENST00000574549,ENST00000575576
ENST00000303372,ENST00000426174
ENST00000465301,ENST00000540313
ENST00000433626
ENST00000361722
ENST00000050961,ENST00000455469,ENST00000517585,ENST00000518282,ENST00000520307,ENST00000
ENST00000357409,ENST00000360280,ENST00000376634,ENST00000376636
ENST00000319715,ENST00000348022,ENST00000564314
ENST00000333188,ENST00000507777
ENST00000264357,ENST00000371251,ENST00000371256,ENST00000541091,ENST00000542149
ENST00000368799
ENST00000264187
ENST00000317393,ENST00000340177
ENST00000263461
ENST00000264233
ENST00000381356,ENST00000556351,ENST00000556885,ENST00000556927,ENST00000557540
ENST00000389232,ENST00000415757
ENST00000251091,ENST00000357634,ENST00000392067,ENST00000554335,ENST00000555192
ENST00000360374
ENST00000290239,ENST00000300278,ENST00000356577,ENST00000381692,ENST00000421541,ENST00000

ENST00000361188,ENST00000361919,ENST00000394249,ENST00000442656
ENST00000315677,ENST00000515196,ENST00000581527,ENST00000584874
ENST00000262233,ENST00000327921,ENST00000334192
ENST00000326261,ENST00000382841,ENST00000538872
ENST00000217420
ENST00000443617
ENST00000553796
ENST00000258400
ENST00000345941,ENST00000378434
ENST00000248901
ENST00000375244,ENST00000375247,ENST00000451343
ENST00000392413,ENST00000600328,ENST00000600359
ENST00000259605,ENST00000350199,ENST00000353739,ENST00000357058,ENST00000377877,ENST00000[illegible]
ENST00000316924,ENST00000394065,ENST00000394066,ENST00000394067,ENST00000394078,ENST00000[illegible]
ENST00000357084,ENST00000395503,ENST00000536376
ENST00000332047,ENST00000337090,ENST00000354446,ENST00000383229,ENST00000513612,ENST00000[illegible]
ENST00000299821,ENST00000395698,ENST00000395701,ENST00000420993,ENST00000523045
ENST00000257665,ENST00000453279
ENST00000245923,ENST00000344680,ENST00000587597,ENST00000591286
ENST00000053468
ENST00000337917,ENST00000376049,ENST00000376059
ENST00000299853,ENST00000359210,ENST00000418581,ENST00000564209
ENST00000296782,ENST00000357387
ENST00000342175,ENST00000342992,ENST00000359218,ENST00000460472,ENST00000589042,ENST00000[illegible]
ENST00000379388
ENST00000302797
ENST00000322644
ENST00000297784,ENST00000340019,ENST00000396237
ENST00000367685
ENST00000361952,ENST00000578339
ENST00000477478,ENST00000487415,ENST00000574508,ENST00000575403,ENST00000575478,ENST00000[illegible]
ENST00000349953,ENST00000351153,ENST00000358768,ENST00000406610,ENST00000462231
ENST00000221347
ENST00000261509,ENST00000333488,ENST00000505667,ENST00000508898
ENST00000309446,ENST00000412414,ENST00000421199
ENST00000270570,ENST00000395585,ENST00000457293,ENST00000571335,ENST00000575023
ENST00000337233,ENST00000359949,ENST00000538701,ENST00000542067,ENST00000543171
ENST00000335875,ENST00000454748
ENST00000392413,ENST00000600328,ENST00000600359
ENST00000320574,ENST00000535270,ENST00000539006
ENST00000361897,ENST00000430120,ENST00000530878
ENST00000452804
ENST00000322090,ENST00000446778
ENST00000336180,ENST00000418310,ENST00000538333
ENST00000355619
ENST00000014930
ENST00000274963

ENST00000274392,ENST00000304400,ENST00000346918,ENST00000348126,ENST00000379787,ENST00000
ENST00000361897,ENST00000431696,ENST00000493151,ENST00000530878
ENST00000221992,ENST00000398599,ENST00000405816,ENST00000598976
ENST00000241651
ENST00000240874,ENST00000360013,ENST00000460856
ENST00000320580,ENST00000529757
ENST00000417545
ENST00000330133
ENST00000396878,ENST00000416391,ENST00000574902,ENST00000576416
ENST00000369111
ENST00000255759,ENST00000573429,ENST00000575744,ENST00000575938
ENST00000323418,ENST00000359636,ENST00000393324,ENST00000524826,ENST00000526504,ENST00000
ENST00000368804
ENST00000338779
ENST00000242480,ENST00000411732,ENST00000439032
ENST00000374202,ENST00000464642,ENST00000487883
ENST00000299612,ENST00000308406,ENST00000395602,ENST00000395604
ENST00000343816,ENST00000352432,ENST00000360273,ENST00000383800,ENST00000397077,ENST00000
ENST00000292807,ENST00000382456,ENST00000411763,ENST00000432591,ENST00000439647
ENST00000360215,ENST00000373853
ENST00000331351
ENST00000289893,ENST00000317713,ENST00000361689,ENST00000372915,ENST00000539005,ENST00000
ENST00000378123,ENST00000531613
ENST00000336301,ENST00000508628,ENST00000582970
ENST00000327564,ENST00000341671,ENST00000394713,ENST00000543378,ENST00000578674,ENST00000
ENST00000261187,ENST00000543448,ENST00000547379,ENST00000548610,ENST00000552024,ENST00000
ENST00000381621,ENST00000381623
ENST00000251481,ENST00000326853,ENST00000409880,ENST00000437390,ENST00000438339
ENST00000255641
ENST00000345122,ENST00000432921,ENST00000539826,ENST00000556611
ENST00000397071,ENST00000534993,ENST00000545240
ENST00000289004,ENST00000543163
ENST00000258083
ENST00000261374
ENST00000393217
ENST00000333629
ENST00000489294,ENST00000545294
ENST00000381055,ENST00000464680
ENST00000337920,ENST00000395376,ENST00000395379,ENST00000395386,ENST00000395390,ENST00000
ENST00000243914,ENST00000371196,ENST00000422869,ENST00000423479,ENST00000426658,ENST00000
ENST00000383820,ENST00000418713,ENST00000424438,ENST00000433972
ENST00000321897,ENST00000416670,ENST00000541562,ENST00000542001
ENST00000234739
ENST00000287898,ENST00000296794,ENST00000426542,ENST00000437974,ENST00000513042,ENST00000
ENST00000300176
ENST00000338675,ENST00000382409
ENST00000192788,ENST00000452449

ENST00000298048,ENST00000536329,ENST00000536860,ENST00000536987,ENST00000538311,ENST00000
ENST00000330394
ENST00000374300,ENST00000374301,ENST00000374303,ENST00000374307,ENST00000524618
ENST00000313386,ENST00000502814,ENST00000507457,ENST00000515098,ENST00000542098
ENST00000340684,ENST00000543802
ENST00000263981,ENST00000281708,ENST00000296555,ENST00000393956,ENST00000603548,ENST00000
ENST00000246100,ENST00000304189,ENST00000381939,ENST00000381941,ENST00000541082
ENST00000269703,ENST00000601005
ENST00000323927
ENST00000300917,ENST00000543872
ENST00000372827,ENST00000397360
ENST00000283645,ENST00000453949
ENST00000338957,ENST00000423889
ENST00000595295
ENST00000276123,ENST00000360700,ENST00000373165,ENST00000395402
ENST00000374469,ENST00000401783
ENST00000301030,ENST00000378330
ENST00000344387,ENST00000383744,ENST00000383745,ENST00000396058
ENST00000355773,ENST00000393019
ENST00000356309,ENST00000409339,ENST00000409423,ENST00000432615,ENST00000449727
ENST00000411975,ENST00000435803,ENST00000544596
ENST00000303354,ENST00000375387,ENST00000409435,ENST00000409672,ENST00000452182,ENST00000
ENST00000368868,ENST00000423070,ENST00000426705,ENST00000427867,ENST00000427977,ENST00000
ENST00000333172,ENST00000351136
ENST00000340188,ENST00000366782,ENST00000366783,ENST00000391872,ENST00000422240,ENST00000
ENST00000320574,ENST00000535270,ENST00000539006
ENST00000296440,ENST00000358459,ENST00000358536,ENST00000448774,ENST00000456774
ENST00000539556
ENST00000313400,ENST00000361209,ENST00000373996
ENST00000333447,ENST00000407050,ENST00000443686,ENST00000541017,ENST00000541570,ENST00000
ENST00000564067
ENST00000315215,ENST00000412232
ENST00000299847,ENST00000397827,ENST00000401522
ENST00000380255,ENST00000380262,ENST00000563694,ENST00000565740
ENST00000251268,ENST00000334370
ENST00000347559,ENST00000361308,ENST00000368297,ENST00000368299,ENST00000368300,ENST00000
ENST00000431683
ENST00000509479
ENST00000322937,ENST00000449493
ENST00000319274,ENST00000342981,ENST00000346169,ENST00000350481,ENST00000352767,ENST00000
ENST00000265381
ENST00000229304
ENST00000271636
ENST00000360726,ENST00000442253,ENST00000512121
ENST00000394807
ENST00000361926,ENST00000421050,ENST00000579439,ENST00000583529
ENST00000311008,ENST00000323081,ENST00000334536,ENST00000352260,ENST00000565975,ENST00000

ENST00000321725
ENST00000366578,ENST00000542672
ENST00000397910
ENST00000248248,ENST00000439557,ENST00000545553
ENST00000390294
ENST00000306065
ENST00000221538,ENST00000600188
ENST00000374263,ENST00000374264,ENST00000374270
ENST00000343115,ENST00000405097,ENST00000528498,ENST00000528900,ENST00000530085,ENST00000[illegible]
ENST00000375394,ENST00000461073
ENST00000396952
ENST00000244496
ENST00000358241
ENST00000338745,ENST00000414509,ENST00000415069,ENST00000417717,ENST00000440737,ENST00000[illegible]
ENST00000342494,ENST00000434735,ENST00000539599
ENST00000389232,ENST00000415757
ENST00000256031,ENST00000439040
ENST00000253812
ENST00000372262,ENST00000537909
ENST00000419482,ENST00000444564
ENST00000360359,ENST00000535627
ENST00000330459,ENST00000537025
ENST00000347464,ENST00000373525,ENST00000392017,ENST00000392018,ENST00000392020
ENST00000330452,ENST00000394729
ENST00000333676,ENST00000541223,ENST00000571730
ENST00000335367,ENST00000397238,ENST00000463381,ENST00000468796,ENST00000473312
ENST00000358970
ENST00000262861,ENST00000308942,ENST00000366630
ENST00000391571
ENST00000350125,ENST00000352251
ENST00000339306,ENST00000379660
ENST00000278935
ENST00000262662,ENST00000371761,ENST00000396148
ENST00000306279
ENST00000263094,ENST00000433129,ENST00000435683
ENST00000264183,ENST00000349213,ENST00000366603,ENST00000421364
ENST00000493463,ENST00000603053
ENST00000293670
ENST00000373299,ENST00000373302
ENST00000319968
ENST00000284259,ENST00000422003,ENST00000524726,ENST00000528512,ENST00000529397,ENST00000[illegible]
ENST00000433765
ENST00000312055,ENST00000354558,ENST00000415402,ENST00000526876,ENST00000529230
ENST00000335110,ENST00000394329
ENST00000376405,ENST00000376406
ENST00000313599,ENST00000396630,ENST00000416109
ENST00000221265,ENST00000221266,ENST00000595564

ENST00000288502,ENST00000374049
ENST00000373298,ENST00000434584
ENST00000310193
ENST00000361699,ENST00000361981,ENST00000370055,ENST00000370057,ENST00000370060,ENST00000[illegible]
ENST00000368804
ENST00000314672,ENST00000580652,ENST00000583023
ENST00000309244,ENST00000354232,ENST00000596253
ENST00000377850
ENST00000260762,ENST00000371547,ENST00000371552,ENST00000443748
ENST00000368799
ENST00000251582
ENST00000367600,ENST00000367602
ENST00000375135
ENST00000356338,ENST00000358212,ENST00000399229,ENST00000399231,ENST00000399233,ENST00000[illegible]
ENST00000379802,ENST00000418664
ENST00000383781,ENST00000383785,ENST00000383787,ENST00000383788,ENST00000435459,ENST00000[illegible]
ENST00000373479,ENST00000469731,ENST00000487950
ENST00000331920,ENST00000375274,ENST00000418258,ENST00000421141,ENST00000429896,ENST00000[illegible]
ENST00000263377
ENST00000379593,ENST00000379607
ENST00000542956
ENST00000261374
ENST00000355619
ENST00000263981,ENST00000281708,ENST00000296555,ENST00000393956,ENST00000603548,ENST00000[illegible]
ENST00000330452,ENST00000394729
ENST00000345941,ENST00000378434
ENST00000356309,ENST00000409339,ENST00000409423,ENST00000432615,ENST00000449727
ENST00000218507,ENST00000372927,ENST00000423383
ENST00000433626
ENST00000262715,ENST00000555727,ENST00000556935
ENST00000308284
ENST00000564067
ENST00000355235,ENST00000356834
ENST00000582857
ENST00000323927
ENST00000309446,ENST00000412414,ENST00000421199
ENST00000313400,ENST00000361209,ENST00000373996
ENST00000539556
ENST00000310193
ENST00000322644
ENST00000319968
ENST00000333172,ENST00000351136
ENST00000300589
ENST00000320574,ENST00000535270,ENST00000539006
ENST00000366578,ENST00000542672
ENST00000355773,ENST00000393019
ENST00000355773,ENST00000393019

ENST00000381356,ENST00000556351,ENST00000556885,ENST00000556927,ENST00000557540
ENST00000298048,ENST00000536329,ENST00000536860,ENST00000536987,ENST00000538311,ENST00000[illegible]
ENST00000417545
ENST00000333676,ENST00000541223,ENST00000571730
ENST00000014930
ENST00000253799
ENST00000314531
ENST00000359947,ENST00000372413,ENST00000372414,ENST00000422171,ENST00000438120
ENST00000246100,ENST00000304189,ENST00000381939,ENST00000381941,ENST00000541082
ENST00000248901
ENST00000316924,ENST00000394065,ENST00000394066,ENST00000394067,ENST00000394078,ENST00000[illegible]
ENST00000317393,ENST00000340177
ENST00000320574,ENST00000535270,ENST00000539006
ENST00000372262,ENST00000537909
ENST00000320580,ENST00000529757
ENST00000343816,ENST00000352432,ENST00000360273,ENST00000383800,ENST00000397077,ENST00000[illegible]
ENST00000327097,ENST00000541757
ENST00000357409,ENST00000360280,ENST00000376634,ENST00000376636
ENST00000248248,ENST00000439557,ENST00000545553
ENST00000253952,ENST00000326266,ENST00000574549,ENST00000575576
ENST00000367600,ENST00000367602
ENST00000367685
ENST00000333447,ENST00000407050,ENST00000443686,ENST00000541017,ENST00000541570,ENST00000[illegible]
ENST00000374300,ENST00000374301,ENST00000374303,ENST00000374307,ENST00000524618
ENST00000251091,ENST00000357634,ENST00000392067,ENST00000554335,ENST00000555192
ENST00000336301,ENST00000508628,ENST00000582970
ENST00000251772,ENST00000393409
ENST00000375244,ENST00000375247
ENST00000344626,ENST00000358026,ENST00000413806,ENST00000429416,ENST00000444061,ENST00000[illegible]
ENST00000339994,ENST00000528731
ENST00000296440,ENST00000358459,ENST00000358536,ENST00000448774,ENST00000456774
ENST00000243914,ENST00000371196,ENST00000422869,ENST00000423479,ENST00000426658,ENST00000[illegible]
ENST00000321871,ENST00000393130,ENST00000510183,ENST00000511555,ENST00000512137,ENST00000[illegible]
ENST00000368868,ENST00000423070,ENST00000426705,ENST00000427867,ENST00000427977,ENST00000[illegible]
ENST00000337917,ENST00000376049,ENST00000376059
ENST00000312358
ENST00000262662,ENST00000371761,ENST00000396148
ENST00000330133
ENST00000360215,ENST00000373853
ENST00000292199,ENST00000409109,ENST00000409265,ENST00000409991,ENST00000525863
ENST00000261509,ENST00000333488,ENST00000505667,ENST00000508898
ENST00000538210,ENST00000606080
ENST00000264111,ENST00000397081,ENST00000397087,ENST00000535187,ENST00000538974,ENST00000[illegible]
ENST00000574730
ENST00000263094,ENST00000433129,ENST00000435683
ENST00000259605,ENST00000350199,ENST00000353739,ENST00000357058,ENST00000377877,ENST00000[illegible]
ENST00000529308

ENST00000361952,ENST00000578339
ENST00000489294,ENST00000545294
ENST00000361897,ENST00000430120,ENST00000530878
ENST00000277817,ENST00000373700,ENST00000395198,ENST00000412272
ENST00000361897,ENST00000493151,ENST00000530878,ENST00000431696
ENST00000306279
ENST00000258400
ENST00000229304
ENST00000375394,ENST00000461073
ENST00000304141
ENST00000379802,ENST00000418664
ENST00000299821,ENST00000395698,ENST00000395701,ENST00000420993,ENST00000523045
ENST00000360215,ENST00000373853
ENST00000241651
ENST00000246957,ENST00000538171,ENST00000575671
ENST00000255641
ENST00000349953,ENST00000351153,ENST00000358768,ENST00000406610,ENST00000462231
ENST00000383820,ENST00000418713,ENST00000424438,ENST00000433972
ENST00000242480,ENST00000411732,ENST00000439032
ENST00000361722
ENST00000251472
ENST00000192788,ENST00000452449
ENST00000369768
ENST00000394807
ENST00000394807
ENST00000289004,ENST00000543163
ENST00000397910
ENST00000326261,ENST00000382841,ENST00000538872
ENST00000525985
ENST00000226207
ENST00000373192
ENST00000337920,ENST00000395376,ENST00000395379,ENST00000395386,ENST00000395390,ENST00000[illegible]
ENST00000356338,ENST00000358212,ENST00000399229,ENST00000399231,ENST00000399233,ENST00000[illegible]
ENST00000251481,ENST00000326853,ENST00000409880,ENST00000437390,ENST00000438339
ENST00000411975,ENST00000435803,ENST00000544596
ENST00000336640,ENST00000356826,ENST00000394409,ENST00000394410,ENST00000394411,ENST00000[illegible]
ENST00000373479,ENST00000469731,ENST00000487950
ENST00000306065
ENST00000302797
ENST00000311008,ENST00000323081,ENST00000334536,ENST00000352260,ENST00000565975,ENST00000[illegible]
ENST00000337233,ENST00000359949,ENST00000538701,ENST00000542067,ENST00000543171
ENST00000299612,ENST00000308406,ENST00000395602,ENST00000395604
ENST00000347464,ENST00000373525,ENST00000392017,ENST00000392018,ENST00000392020
ENST00000393079,ENST00000446465
ENST00000318967,ENST00000435409,ENST00000456435,ENST00000512605
ENST00000326197,ENST00000337495,ENST00000397174,ENST00000402904
ENST00000326197,ENST00000337495,ENST00000397174,ENST00000402904

ENST00000326197,ENST00000337495,ENST00000397174,ENST00000402904
ENST00000326197,ENST00000337495,ENST00000397174,ENST00000402904
ENST00000326197,ENST00000337495,ENST00000397174,ENST00000402904
ENST00000360726,ENST00000442253,ENST00000512121
ENST00000366956
ENST00000360359,ENST00000535627
ENST00000292807,ENST00000382456,ENST00000411763,ENST00000432591,ENST00000439647
ENST00000259365,ENST00000375175,ENST00000395211
ENST00000260762,ENST00000371547,ENST00000371552,ENST00000443748
ENST00000303354,ENST00000375387,ENST00000409435,ENST00000409672
ENST00000360374
ENST00000375135
ENST00000290178,ENST00000331923
ENST00000333629
ENST00000368804
ENST00000443617
ENST00000296782,ENST00000357387
ENST00000330459,ENST00000537025
ENST00000396952
ENST00000321919,ENST00000381983,ENST00000436648,ENST00000591451
ENST00000335367,ENST00000397238,ENST00000463381,ENST00000468796,ENST00000473312
ENST00000321897,ENST00000416670,ENST00000541562,ENST00000542001
ENST00000335875,ENST00000454748,ENST00000594180
ENST00000240874,ENST00000360013,ENST00000460856
ENST00000261187,ENST00000543448,ENST00000547379,ENST00000548610,ENST00000552024,ENST00000[illegible]
ENST00000335110,ENST00000394329
ENST00000283645,ENST00000453949
ENST00000231368,ENST00000395770
ENST00000297784,ENST00000340019,ENST00000396237
ENST00000273739,ENST00000503823,ENST00000503837,ENST00000504154
ENST00000258083
ENST00000309244,ENST00000354232,ENST00000596253
ENST00000274963
ENST00000300176
ENST00000339306,ENST00000379660
ENST00000333188,ENST00000507777
ENST00000244496
ENST00000225916
ENST00000331351
ENST00000378123,ENST00000531613
ENST00000299853,ENST00000359210,ENST00000418581,ENST00000564209
ENST00000336180,ENST00000418310,ENST00000538333
ENST00000374202,ENST00000464642,ENST00000487883
ENST00000255759,ENST00000573429,ENST00000575744,ENST00000575938
ENST00000371744,ENST00000371752,ENST00000371754,ENST00000396105,ENST00000455070
ENST00000264357,ENST00000371251,ENST00000371256,ENST00000541091,ENST00000542149
ENST00000361897,ENST00000464284,ENST00000493151,ENST00000530878,ENST00000431696

ENST00000289893,ENST00000317713,ENST00000361689,ENST00000372915,ENST00000539005,ENST00000
ENST00000263461
ENST00000340684
ENST00000391571
ENST00000274392,ENST00000304400,ENST00000346918,ENST00000348126,ENST00000379787,ENST00000
ENST00000331920,ENST00000375274,ENST00000418258,ENST00000421141,ENST00000429896,ENST00000
ENST00000344387,ENST00000383744,ENST00000383745,ENST00000396058
ENST00000299847,ENST00000397827,ENST00000401522
ENST00000598976,ENST00000221992,ENST00000398599,ENST00000405816
ENST00000338957,ENST00000423889
ENST00000373299,ENST00000373302
ENST00000257622,ENST00000358357,ENST00000395270,ENST00000434423,ENST00000446813
ENST00000509479
ENST00000332047,ENST00000337090,ENST00000354446,ENST00000383229,ENST00000504929,ENST00000
ENST00000330394
ENST00000321725
ENST00000343115,ENST00000405097,ENST00000528498,ENST00000528900,ENST00000530085,ENST00000
ENST00000358241
ENST00000493463,ENST00000603053
ENST00000271636
ENST00000315677,ENST00000515196,ENST00000581527,ENST00000584874
ENST00000408907
ENST00000380255,ENST00000380262,ENST00000563694,ENST00000565740
ENST00000347559,ENST00000361308,ENST00000368297,ENST00000368299,ENST00000368300,ENST00000
ENST00000256031,ENST00000439040
ENST00000312055,ENST00000354558,ENST00000415402,ENST00000526876,ENST00000529230
ENST00000053468
ENST00000314672,ENST00000580652,ENST00000583023
ENST00000300917,ENST00000543872
ENST00000287898,ENST00000296794,ENST00000426542,ENST00000437974,ENST00000513042,ENST00000
ENST00000303372,ENST00000426174
ENST00000393217
ENST00000334634,ENST00000368382,ENST00000368383,ENST00000545012
ENST00000313599,ENST00000396630,ENST00000416109
ENST00000284259,ENST00000422003,ENST00000524726,ENST00000528512,ENST00000529397,ENST00000
ENST00000315215,ENST00000412232
ENST00000262233,ENST00000327921,ENST00000334192
ENST00000390294
ENST00000381055,ENST00000464680
ENST00000301030,ENST00000378330
ENST00000420713
ENST00000264187
ENST00000278935
ENST00000379388
ENST00000310998,ENST00000375921,ENST00000375922,ENST00000375934,ENST00000375938,ENST00000
ENST00000375244,ENST00000375247,ENST00000451343
ENST00000251582

ENST00000372827,ENST00000397360
ENST00000269703,ENST00000601005
ENST00000350125,ENST00000352251
ENST00000251268,ENST00000334370
ENST00000217420
ENST00000381621,ENST00000381623
ENST00000338779
ENST00000172853,ENST00000397345,ENST00000409198,ENST00000413693,ENST00000427231,ENST00000[illegible]
ENST00000389232,ENST00000415757
ENST00000257665,ENST00000453279
ENST00000419482,ENST00000444564
ENST00000465301,ENST00000540313
ENST00000361188,ENST00000361919,ENST00000394249,ENST00000442656
ENST00000339656,ENST00000419854,ENST00000423137
ENST00000344096,ENST00000393555
ENST00000431683
ENST00000374263,ENST00000374264,ENST00000374270
ENST00000322937,ENST00000449493
ENST00000221265,ENST00000221266,ENST00000595564
ENST00000361699,ENST00000361981,ENST00000370055,ENST00000370057,ENST00000370060,ENST00000[illegible]
ENST00000313386,ENST00000502814,ENST00000507457,ENST00000515098,ENST00000542098
ENST00000493964
ENST00000253812
ENST00000361926,ENST00000421050,ENST00000579439,ENST00000583529
ENST00000263377
ENST00000361003,ENST00000389707,ENST00000389708,ENST00000396441,ENST00000403290,ENST00000[illegible]
ENST00000452804
ENST00000327564,ENST00000341671,ENST00000394713,ENST00000543378,ENST00000578674,ENST00000[illegible]
ENST00000392413,ENST00000600328,ENST00000600359,ENST00000392413,ENST00000600328,ENST00000[illegible]
ENST00000357084,ENST00000395503,ENST00000536376
ENST00000264233
ENST00000433197
ENST00000389232,ENST00000415757
ENST00000221538,ENST00000600188
ENST00000322090,ENST00000446778
ENST00000319274,ENST00000342981,ENST00000346169,ENST00000350481,ENST00000352767,ENST00000[illegible]
ENST00000276123,ENST00000360700,ENST00000373165,ENST00000395402
ENST00000338675
ENST00000270570,ENST00000395585,ENST00000457293,ENST00000571335,ENST00000575023
ENST00000342175,ENST00000342992,ENST00000359218,ENST00000460472,ENST00000589042,ENST00000[illegible]
ENST00000345122,ENST00000432921,ENST00000539826,ENST00000556611
ENST00000396878,ENST00000416391,ENST00000574902,ENST00000576416
ENST00000335125,ENST00000382755,ENST00000397071,ENST00000534993,ENST00000545240,ENST00000[illegible]
ENST00000342494,ENST00000434735,ENST00000539599
ENST00000341919,ENST00000346156,ENST00000395111,ENST00000443272,ENST00000586919,ENST00000[illegible]
ENST00000472091,ENST00000542862
ENST00000293670

ENST00000383781,ENST00000383785,ENST00000383787,ENST00000383788,ENST00000435459,ENST00000
ENST00000234739
ENST00000264183,ENST00000349213,ENST00000366603,ENST00000421364
ENST00000514667,ENST00000296859,ENST00000307984,ENST00000308008,ENST00000507093,ENST00000
ENST00000221347
ENST00000369111
ENST00000262861,ENST00000308942,ENST00000366630
ENST00000340188,ENST00000366782,ENST00000366783,ENST00000391872,ENST00000422240,ENST00000
ENST00000050961,ENST00000455469,ENST00000517585,ENST00000518282,ENST00000520307,ENST00000
ENST00000373298,ENST00000434584
ENST00000319715,ENST00000348022,ENST00000564314
ENST00000433765
ENST00000338745,ENST00000414509,ENST00000415069,ENST00000417717,ENST00000440737,ENST00000
ENST00000377850
ENST00000290239,ENST00000300278,ENST00000356577,ENST00000381692,ENST00000421541,ENST00000
ENST00000368799
ENST00000374469,ENST00000401783
ENST00000376405,ENST00000376406
ENST00000288502,ENST00000374049
ENST00000323418,ENST00000359636,ENST00000393324,ENST00000524826,ENST00000526504,ENST00000
ENST00000245923,ENST00000344680,ENST00000587597,ENST00000591286
ENST00000368799
ENST00000379593,ENST00000379607
ENST00000265381
ENST00000358970
ENST00000368804
ENST00000263671,ENST00000376332,ENST00000528789,ENST00000534276
ENST00000281815,ENST00000338347
ENST00000342960,ENST00000490598
ENST00000300976
ENST00000303015,ENST00000520439,ENST00000522317
ENST00000440491
ENST00000390373
ENST00000402038
ENST00000302850,ENST00000341500
ENST00000281815,ENST00000338347,ENST00000342960,ENST00000369338,ENST00000369339
ENST00000252252
ENST00000421673
ENST00000390373
ENST00000419039,ENST00000420871,ENST00000456556
ENST00000261483
ENST00000278882,ENST00000358464,ENST00000439954
ENST00000323927
ENST00000252071,ENST00000477871,ENST00000478393,ENST00000539352
ENST00000304514,ENST00000409886
ENST00000283351,ENST00000582539
ENST00000417545

ENST00000278882,ENST00000358464,ENST00000439954
ENST00000355673,ENST00000431731,ENST00000547545
ENST00000335110,ENST00000394329
ENST00000260356
ENST00000420699,ENST00000461153
ENST00000249007,ENST00000382088,ENST00000397468
ENST00000391417
ENST00000322396,ENST00000541232
ENST00000439954
ENST00000381055,ENST00000464680
ENST00000557788
ENST00000342947,ENST00000409775,ENST00000409970,ENST00000419307,ENST00000545231
ENST00000418749
ENST00000392002
ENST00000452611
ENST00000299440,ENST00000534663
ENST00000342960,ENST00000369338,ENST00000369339
ENST00000348028,ENST00000349350,ENST00000421100,ENST00000427578,ENST00000443370,ENST00000[illegible]
ENST00000399682
ENST00000348028,ENST00000349350,ENST00000421100,ENST00000427578,ENST00000443370,ENST00000[illegible]
ENST00000356641,ENST00000397843,ENST00000532993
ENST00000204517
ENST00000333891,ENST00000423517
ENST00000489525
ENST00000281815,ENST00000338347
ENST00000302850,ENST00000341500
ENST00000322396,ENST00000541232
ENST00000390373
ENST00000300976
ENST00000376324
ENST00000252071,ENST00000478393,ENST00000539352
ENST00000323927
ENST00000402038
ENST00000381055,ENST00000464680
ENST00000392002
ENST00000342960,ENST00000369338,ENST00000369339
ENST00000335110,ENST00000394329
ENST00000261483
ENST00000278882,ENST00000358464,ENST00000439954,ENST00000278882,ENST00000358464,ENST00000[illegible]
ENST00000281815,ENST00000338347
ENST00000557788
ENST00000249007,ENST00000382088,ENST00000397468
ENST00000421673
ENST00000281815,ENST00000338347
ENST00000342947,ENST00000409775,ENST00000409970,ENST00000419307,ENST00000545231
ENST00000417545
ENST00000356641,ENST00000397843,ENST00000532993

ENST00000342960,ENST00000490598
ENST00000418749
ENST00000299440,ENST00000534663
ENST00000304514,ENST00000409886
ENST00000439954
ENST00000419039,ENST00000420871,ENST00000456556
ENST00000489525
ENST00000452611
ENST00000390373
ENST00000278882,ENST00000358464,ENST00000439954
ENST00000204517
ENST00000283351,ENST00000582539
ENST00000252252
ENST00000420699,ENST00000461153
ENST00000303015,ENST00000520439,ENST00000522317
ENST00000348028,ENST00000349350,ENST00000421100,ENST00000427578,ENST00000443370,ENST00000[illegible]
ENST00000260356
ENST00000355673,ENST00000431731,ENST00000547545
ENST00000399682
ENST00000391417
ENST00000348028,ENST00000349350,ENST00000421100,ENST00000427578,ENST00000443370,ENST00000[illegible]
ENST00000333891,ENST00000423517
ENST00000263671,ENST00000376332,ENST00000528789,ENST00000534276
ENST00000278882,ENST00000358464,ENST00000439954
ENST00000281815,ENST00000338347
ENST00000287647,ENST00000383807,ENST00000419585
ENST00000379829
ENST00000342960,ENST00000490598
ENST00000330891,ENST00000488961,ENST00000553096
ENST00000300976
ENST00000390373
ENST00000333244
ENST00000252321
ENST00000281815,ENST00000338347
ENST00000419039,ENST00000420871,ENST00000456556
ENST00000421673
ENST00000332707
ENST00000397056,ENST00000431010,ENST00000438284
ENST00000419039,ENST00000420871,ENST00000456556
ENST00000419039,ENST00000420871,ENST00000456556
ENST00000278882,ENST00000358464,ENST00000439954
ENST00000273541,ENST00000393292,ENST00000393295,ENST00000418265,ENST00000485703
ENST00000257622,ENST00000358357,ENST00000395270,ENST00000434423,ENST00000446813
ENST00000323927
ENST00000340291,ENST00000396638,ENST00000472605,ENST00000475644,ENST00000484764
ENST00000278882,ENST00000358464,ENST00000439954
ENST00000304514,ENST00000409886

ENST00000335110,ENST00000394329
ENST00000179882,ENST00000321680,ENST00000367480,ENST00000367481
ENST00000376581
ENST00000358970,ENST00000511306
ENST00000420699,ENST00000461153
ENST00000249007,ENST00000382088,ENST00000397468
ENST00000391417
ENST00000532254,ENST00000533640
ENST00000439954
ENST00000289153,ENST00000461451,ENST00000462898,ENST00000465581,ENST00000477593,ENST00000[illegible]
ENST00000557788
ENST00000447027,ENST00000529681
ENST00000452611
ENST00000299440,ENST00000534663
ENST00000456556
ENST00000399682
ENST00000348028,ENST00000349350,ENST00000421100,ENST00000427578,ENST00000443370,ENST00000[illegible]
ENST00000376296
ENST00000376296
ENST00000338347,ENST00000440491
ENST00000356641,ENST00000397843,ENST00000532993
ENST00000379829
ENST00000281815,ENST00000338347
ENST00000340291,ENST00000396638,ENST00000472605,ENST00000475644,ENST00000484764
ENST00000330891,ENST00000488961,ENST00000553096
ENST00000390373
ENST00000300976
ENST00000376324
ENST00000397056,ENST00000431010,ENST00000438284
ENST00000323927
ENST00000447027,ENST00000529681
ENST00000257622,ENST00000358357,ENST00000395270,ENST00000434423,ENST00000446813
ENST00000332707
ENST00000273541,ENST00000393292,ENST00000393295,ENST00000485703,ENST00000418265
ENST00000333244
ENST00000278882,ENST00000358464,ENST00000439954,ENST00000278882,ENST00000358464,ENST00000[illegible]
ENST00000376581
ENST00000289153,ENST00000461451,ENST00000462898,ENST00000465581,ENST00000477593,ENST00000[illegible]
ENST00000287647,ENST00000383807,ENST00000419585
ENST00000335110,ENST00000394329
ENST00000179882,ENST00000321680,ENST00000367480,ENST00000367481
ENST00000557788
ENST00000249007,ENST00000382088,ENST00000397468
ENST00000421673
ENST00000281815,ENST00000338347
ENST00000376296
ENST00000278882,ENST00000358464,ENST00000439954

ENST00000356641,ENST00000397843,ENST00000532993
ENST00000342960,ENST00000490598
ENST00000456556
ENST00000299440,ENST00000534663
ENST00000338347,ENST00000440491
ENST00000304514,ENST00000409886
ENST00000439954
ENST00000419039,ENST00000420871,ENST00000456556,ENST00000419039,ENST00000420871,ENST00000[illegible]
ENST00000376296
ENST00000419039,ENST00000420871,ENST00000456556
ENST00000419039,ENST00000420871,ENST00000456556
ENST00000452611
ENST00000358970,ENST00000511306
ENST00000252321
ENST00000420699,ENST00000461153
ENST00000348028,ENST00000349350,ENST00000421100,ENST00000427578,ENST00000443370,ENST00000[illegible]
ENST00000532254,ENST00000533640
ENST00000399682
ENST00000391417
ENST00000278314,ENST00000536068
ENST00000263671,ENST00000376332,ENST00000528789,ENST00000534276
ENST00000288168,ENST00000321489,ENST00000393550,ENST00000393567,ENST00000425945,ENST00000[illegible]
ENST00000310271,ENST00000394965,ENST00000469224,ENST00000484952
ENST00000278882,ENST00000358464,ENST00000439954
ENST00000281815,ENST00000338347
ENST00000253363,ENST00000338163,ENST00000361162,ENST00000407261,ENST00000528062
ENST00000292432
ENST00000375631
ENST00000342960,ENST00000490598
ENST00000335875
ENST00000334304
ENST00000300976
ENST00000425825
ENST00000265728,ENST00000413643,ENST00000430279
ENST00000264775,ENST00000307259
ENST00000381967,ENST00000505986,ENST00000515668
ENST00000252321
ENST00000281815,ENST00000338347
ENST00000421673
ENST00000358812
ENST00000278882,ENST00000358464,ENST00000439954
ENST00000358951
ENST00000323927
ENST00000231137
ENST00000217961
ENST00000398718
ENST00000561462

ENST00000304514,ENST00000409886
ENST00000278882,ENST00000358464,ENST00000439954
ENST00000342992,ENST00000589042,ENST00000591111
ENST00000223190,ENST00000311967,ENST00000393230,ENST00000393231,ENST00000393232,ENST00000[illegible]
ENST00000335110,ENST00000394329
ENST00000317593,ENST00000513010
ENST00000242848,ENST00000282007
ENST00000420699,ENST00000461153
ENST00000249007,ENST00000382088,ENST00000397468
ENST00000376572,ENST00000376576
ENST00000391417
ENST00000525693
ENST00000346569,ENST00000369047,ENST00000369049
ENST00000418063
ENST00000225550
ENST00000439954
ENST00000257248,ENST00000541311
ENST00000557788
ENST00000422891
ENST00000294008
ENST00000418749
ENST00000452611
ENST00000299440,ENST00000534663
ENST00000361306,ENST00000380602
ENST00000223140,ENST00000467773,ENST00000483238
ENST00000399682
ENST00000278927,ENST00000417453
ENST00000283550,ENST00000375699
ENST00000409072
ENST00000369592,ENST00000369603
ENST00000356641,ENST00000397843,ENST00000532993
ENST00000313173,ENST00000450510,ENST00000544999,ENST00000548663
ENST00000320084,ENST00000336053,ENST00000420743,ENST00000430246,ENST00000449098,ENST00000[illegible]
ENST00000302558,ENST00000409750
ENST00000381967,ENST00000505986,ENST00000515668
ENST00000335875
ENST00000281815,ENST00000338347
ENST00000257248,ENST00000541311
ENST00000358812
ENST00000425825
ENST00000418063
ENST00000292432
ENST00000354464,ENST00000560155,ENST00000561462
ENST00000300976
ENST00000375631
ENST00000376324
ENST00000358951

ENST00000265728,ENST00000413643,ENST00000430279
ENST00000253363,ENST00000338163,ENST00000361162,ENST00000407261,ENST00000528062
ENST00000310271,ENST00000394965,ENST00000469224,ENST00000484952
ENST00000323927
ENST00000231137
ENST00000317593,ENST00000513010
ENST00000288168,ENST00000321489,ENST00000393550,ENST00000393567,ENST00000448089,ENST00000[illegible]
ENST00000264775,ENST00000307259
ENST00000294008
ENST00000335110,ENST00000394329
ENST00000223190,ENST00000311967,ENST00000393230,ENST00000393231,ENST00000393232,ENST00000[illegible]
ENST00000376572,ENST00000376576
ENST00000302558,ENST00000409750
ENST00000278882,ENST00000358464,ENST00000439954,ENST00000278882,ENST00000358464,ENST00000[illegible]
ENST00000225550
ENST00000290200,ENST00000451065
ENST00000557788
ENST00000249007,ENST00000382088,ENST00000397468
ENST00000342992,ENST00000589042,ENST00000591111
ENST00000421673
ENST00000281815,ENST00000338347
ENST00000409072
ENST00000398718
ENST00000278882,ENST00000358464,ENST00000439954
ENST00000356641,ENST00000397843,ENST00000532993
ENST00000342960,ENST00000490598
ENST00000418749
ENST00000217961
ENST00000299440,ENST00000534663
ENST00000223140,ENST00000467773,ENST00000483238
ENST00000304514,ENST00000409886
ENST00000439954
ENST00000334304
ENST00000525693
ENST00000278314,ENST00000536068
ENST00000278927,ENST00000417453
ENST00000361306,ENST00000380602
ENST00000452611
ENST00000346569,ENST00000369047,ENST00000369049
ENST00000369592,ENST00000369603
ENST00000278882,ENST00000358464,ENST00000439954,ENST00000278882,ENST00000358464,ENST00000[illegible]
ENST00000313173,ENST00000450510,ENST00000544999,ENST00000548663
ENST00000252321
ENST00000420699,ENST00000461153
ENST00000283550,ENST00000375699
ENST00000399682
ENST00000320084,ENST00000336053,ENST00000420743,ENST00000430246,ENST00000449098,ENST00000[illegible]

ENST00000391417
ENST00000242848,ENST00000282007
ENST00000263671,ENST00000376332,ENST00000528789,ENST00000534276
ENST00000332836,ENST00000590200
ENST00000368804
ENST00000300976
ENST00000179765,ENST00000379441,ENST00000549787,ENST00000549884,ENST00000551812
ENST00000312026,ENST00000536263,ENST00000595559
ENST00000252321
ENST00000265713,ENST00000396930,ENST00000406337,ENST00000485568
ENST00000421673
ENST00000513506
ENST00000323927
ENST00000306151,ENST00000379439
ENST00000376271
ENST00000315957
ENST00000340291,ENST00000396638,ENST00000472605,ENST00000475644,ENST00000484764
ENST00000415827,ENST00000428826,ENST00000586867,ENST00000590078,ENST00000592743,ENST00000
ENST00000315183,ENST00000395799,ENST00000450465
ENST00000462323,ENST00000463781,ENST00000466475,ENST00000470451,ENST00000475231,ENST00000
ENST00000335110,ENST00000394329
ENST00000376271
ENST00000348028,ENST00000349350,ENST00000421100,ENST00000427578,ENST00000443370,ENST00000
ENST00000283474,ENST00000587615,ENST00000590891,ENST00000591401,ENST00000591897
ENST00000327337,ENST00000543111
ENST00000327564,ENST00000341671,ENST00000394713,ENST00000543378,ENST00000584368
ENST00000348028,ENST00000349350,ENST00000421100,ENST00000427578,ENST00000443370,ENST00000
ENST00000406386
ENST00000327337,ENST00000543111
ENST00000368764,ENST00000392667
ENST00000327337,ENST00000543111
ENST00000306151,ENST00000379439
ENST00000299440,ENST00000534663
ENST00000372950
ENST00000278882,ENST00000358464
ENST00000381285,ENST00000381318,ENST00000399367,ENST00000437442
ENST00000462323,ENST00000463781,ENST00000466475,ENST00000470451,ENST00000475231,ENST00000
ENST00000462323,ENST00000463781,ENST00000466475,ENST00000470451,ENST00000475231,ENST00000
ENST00000306151,ENST00000379439
ENST00000356641,ENST00000397843,ENST00000532993
ENST00000359061,ENST00000441003
ENST00000348028,ENST00000349350,ENST00000421100,ENST00000427578,ENST00000443370,ENST00000
ENST00000382483
ENST00000348028,ENST00000349350,ENST00000421100,ENST00000427578,ENST00000443370,ENST00000
ENST00000340291,ENST00000396638,ENST00000472605,ENST00000475644,ENST00000484764
ENST00000332836,ENST00000590200
ENST00000300976

ENST00000376324
ENST00000306151,ENST00000379439
ENST00000315957
ENST00000323927
ENST00000376271,ENST00000376271
ENST00000415827,ENST00000428826,ENST00000586867,ENST00000590078,ENST00000592743,ENST00000
ENST00000312026,ENST00000536263,ENST00000595559
ENST00000335110,ENST00000394329
ENST00000462323,ENST00000463781,ENST00000466475,ENST00000470451,ENST00000475231,ENST00000
ENST00000327337,ENST00000543111
ENST00000306151,ENST00000379439
ENST00000306151,ENST00000379439
ENST00000179765,ENST00000379441,ENST00000549787,ENST00000549884,ENST00000551812
ENST00000421673
ENST00000327337,ENST00000543111
ENST00000356641,ENST00000397843,ENST00000532993
ENST00000327337,ENST00000543111
ENST00000299440,ENST00000534663
ENST00000382483
ENST00000513506
ENST00000327564,ENST00000341671,ENST00000394713,ENST00000543378,ENST00000584368
ENST00000348028,ENST00000349350,ENST00000421100,ENST00000427578,ENST00000443370,ENST00000
ENST00000372950
ENST00000462323,ENST00000463781,ENST00000466475,ENST00000470451,ENST00000475231,ENST00000
ENST00000348028,ENST00000349350,ENST00000421100,ENST00000427578,ENST00000443370,ENST00000
ENST00000252321
ENST00000381285,ENST00000381318,ENST00000399367,ENST00000437442
ENST00000265713,ENST00000396930,ENST00000406337,ENST00000485568
ENST00000462323,ENST00000463781,ENST00000466475,ENST00000470451,ENST00000475231,ENST00000
ENST00000315183,ENST00000395799,ENST00000450465
ENST00000359061,ENST00000441003
ENST00000278882,ENST00000358464
ENST00000348028,ENST00000349350,ENST00000421100,ENST00000427578,ENST00000443370,ENST00000
ENST00000283474,ENST00000587615,ENST00000590891,ENST00000591401,ENST00000591897
ENST00000348028,ENST00000349350,ENST00000421100,ENST00000427578,ENST00000443370,ENST00000
ENST00000368764,ENST00000392667
ENST00000368804
ENST00000406386
ENST00000376271
ENST00000281154
ENST00000264790,ENST00000394980,ENST00000508372
ENST00000318063
ENST00000333976
ENST00000263915,ENST00000424693,ENST00000446413
ENST00000367995,ENST00000367996
ENST00000205890
ENST00000329357

ENST00000576222
ENST00000221567,ENST00000335056
ENST00000284031,ENST00000426972,ENST00000535924,ENST00000539042,ENST00000542148
ENST00000318063
ENST00000333976
ENST00000284031,ENST00000426972,ENST00000535924,ENST00000539042,ENST00000542148
ENST00000221567,ENST00000335056
ENST00000367995,ENST00000367996
ENST00000281154
ENST00000205890
ENST00000264790,ENST00000394980,ENST00000508372
ENST00000576222
ENST00000263915,ENST00000424693,ENST00000446413
ENST00000329357
ENST00000252512,ENST00000433566,ENST00000434536,ENST00000517551
ENST00000263125,ENST00000397176,ENST00000539722
ENST00000358835,ENST00000368802,ENST00000368805,ENST00000435970
ENST00000260187,ENST00000455332,ENST00000525735
ENST00000409389,ENST00000428135
ENST00000331597,ENST00000427714,ENST00000524388
ENST00000388718
ENST00000340840,ENST00000354325,ENST00000372917,ENST00000372922,ENST00000541110
ENST00000389520,ENST00000424789
ENST00000368320,ENST00000368321
ENST00000298296,ENST00000409007,ENST00000443323,ENST00000536088,ENST00000544766
ENST00000331320,ENST00000405646,ENST00000406191,ENST00000434050,ENST00000438610,ENST00000
ENST00000278409
ENST00000315571
ENST00000331597,ENST00000427714,ENST00000524388
ENST00000358835,ENST00000368802,ENST00000368805,ENST00000435970
ENST00000263125,ENST00000397176,ENST00000539722
ENST00000252512,ENST00000433566,ENST00000434536,ENST00000517551
ENST00000389520,ENST00000424789
ENST00000331320,ENST00000405646,ENST00000406191,ENST00000434050,ENST00000438610,ENST00000
ENST00000278409
ENST00000260187,ENST00000455332,ENST00000525735
ENST00000368320,ENST00000368321
ENST00000409389,ENST00000428135
ENST00000340840,ENST00000354325,ENST00000372917,ENST00000372922,ENST00000541110
ENST00000388718
ENST00000298296,ENST00000409007,ENST00000443323,ENST00000536088,ENST00000544766
ENST00000315571
ENST00000295878,ENST00000491000,ENST00000545063
ENST00000312412,ENST00000518004,ENST00000520237,ENST00000544906
ENST00000394715,ENST00000608382
ENST00000269814,ENST00000312090,ENST00000325464,ENST00000395808,ENST00000586017,ENST00000
ENST00000244751

ENST00000400454
ENST00000374388,ENST00000374403
ENST00000258349,ENST00000367694,ENST00000367696
ENST00000287239,ENST00000372711,ENST00000372714,ENST00000372724,ENST00000372725
ENST00000056233
ENST00000265425,ENST00000360488,ENST00000377850
ENST00000378450,ENST00000378454
ENST00000388726,ENST00000392650,ENST00000424778
ENST00000299084
ENST00000399799
ENST00000264839,ENST00000348717,ENST00000370420,ENST00000401910,ENST00000425662,ENST00000[illegible]
ENST00000323959
ENST00000374690,ENST00000396044,ENST00000504326,ENST00000514029
ENST00000380171,ENST00000405489,ENST00000606999
ENST00000299084
ENST00000412074
ENST00000242848,ENST00000282007
ENST00000362097
ENST00000269814,ENST00000312090,ENST00000325464,ENST00000395808,ENST00000586017,ENST00000[illegible]
ENST00000374388,ENST00000374403
ENST00000295878,ENST00000491000,ENST00000545063
ENST00000287239,ENST00000372711,ENST00000372714,ENST00000372724,ENST00000372725
ENST00000258349,ENST00000367694,ENST00000367696
ENST00000412074
ENST00000394715,ENST00000608382
ENST00000374690,ENST00000396044,ENST00000504326,ENST00000514029
ENST00000299084
ENST00000244751
ENST00000400454
ENST00000388726,ENST00000392650,ENST00000424778
ENST00000299084
ENST00000312412,ENST00000518004,ENST00000520237,ENST00000544906
ENST00000323959
ENST00000399799
ENST00000265425,ENST00000360488,ENST00000377850
ENST00000378450,ENST00000378454
ENST00000056233
ENST00000264839,ENST00000348717,ENST00000370420,ENST00000401910,ENST00000425662,ENST00000[illegible]
ENST00000362097
ENST00000380171,ENST00000405489,ENST00000606999
ENST00000242848,ENST00000282007
ENST00000389173,ENST00000572933
ENST00000306151,ENST00000379439
ENST00000316073
ENST00000342944,ENST00000357002
ENST00000312412,ENST00000518004,ENST00000520237,ENST00000544906
ENST00000439326

ENST00000233099,ENST00000354531,ENST00000425467
ENST00000338977,ENST00000511833
ENST00000300098
ENST00000379214,ENST00000389858
ENST00000304887,ENST00000413702,ENST00000456088
ENST00000395385,ENST00000397126,ENST00000402377
ENST00000415833,ENST00000424340,ENST00000517539,ENST00000528249
ENST00000377386,ENST00000398263,ENST00000409015,ENST00000409232,ENST00000444342
ENST00000341550,ENST00000396807,ENST00000412586,ENST00000444704,ENST00000446105
ENST00000550760
ENST00000348028,ENST00000349350,ENST00000421100,ENST00000427578,ENST00000443370,ENST00000[illegible]
ENST00000354957,ENST00000600738
ENST00000376405,ENST00000376406
ENST00000359788
ENST00000342960
ENST00000348028,ENST00000349350,ENST00000421100,ENST00000427578,ENST00000443370,ENST00000[illegible]
ENST00000388726,ENST00000392650,ENST00000424778
ENST00000397126
ENST00000399799
ENST00000328963,ENST00000535250,ENST00000541564,ENST00000546057
ENST00000216442,ENST00000554087,ENST00000554236,ENST00000555431,ENST00000555625,ENST00000[illegible]
ENST00000356929
ENST00000354957,ENST00000600738
ENST00000380171,ENST00000405489,ENST00000606999
ENST00000355095,ENST00000423484
ENST00000412074
ENST00000331162
ENST00000355095,ENST00000423484
ENST00000377386,ENST00000398263,ENST00000409015,ENST00000409232,ENST00000444342
ENST00000397126,ENST00000397126
ENST00000343776,ENST00000397097
ENST00000362065,ENST00000561594,ENST00000561607,ENST00000565280,ENST00000565466
ENST00000354959,ENST00000356929,ENST00000397126,ENST00000594012,ENST00000595461,ENST00000[illegible]
ENST00000356929
ENST00000439326
ENST00000395385,ENST00000402377
ENST00000233099,ENST00000354531,ENST00000425467
ENST00000306151,ENST00000379439
ENST00000338977,ENST00000511833
ENST00000342944,ENST00000357002
ENST00000389173,ENST00000572933
ENST00000423703
ENST00000300098
ENST00000354957
ENST00000412074
ENST00000541564
ENST00000356929

ENST00000550760
ENST00000415833,ENST00000424340,ENST00000517539,ENST00000528249
ENST00000216442,ENST00000554087,ENST00000554236,ENST00000555431,ENST00000555625,ENST00000
ENST00000355095,ENST00000423484,ENST00000355095,ENST00000423484
ENST00000356929
ENST00000388726,ENST00000392650,ENST00000424778
ENST00000343776,ENST00000397097
ENST00000354959,ENST00000594012,ENST00000595461,ENST00000598381
ENST00000312412,ENST00000518004,ENST00000520237,ENST00000544906
ENST00000399799
ENST00000342960
ENST00000376405,ENST00000376406
ENST00000379214,ENST00000389858
ENST00000397126
ENST00000397126
ENST00000348028,ENST00000349350,ENST00000421100,ENST00000427578,ENST00000443370,ENST00000
ENST00000362065,ENST00000561594,ENST00000561607,ENST00000565280,ENST00000565466
ENST00000304887,ENST00000413702,ENST00000456088
ENST00000377386,ENST00000398263,ENST00000409015,ENST00000409232,ENST00000444342,ENST00000
ENST00000331162
ENST00000380171,ENST00000405489,ENST00000606999
ENST00000359788
ENST00000354957,ENST00000600738
ENST00000316073
ENST00000348028,ENST00000349350,ENST00000421100,ENST00000427578,ENST00000443370,ENST00000
ENST00000284476
ENST00000268482
ENST00000361193,ENST00000411502,ENST00000546047,ENST00000571215,ENST00000572725
ENST00000400555,ENST00000400556,ENST00000580745,ENST00000581250,ENST00000582240,ENST00000
ENST00000301547
ENST00000370791,ENST00000443751
ENST00000217130,ENST00000369888,ENST00000450537
ENST00000391571
ENST00000380255,ENST00000380262,ENST00000563694,ENST00000565740,ENST00000575408,ENST00000
ENST00000263087
ENST00000332509,ENST00000335539,ENST00000402064,ENST00000430886
ENST00000256495
ENST00000415833,ENST00000424340,ENST00000517539,ENST00000528249
ENST00000344113,ENST00000358025,ENST00000554584
ENST00000380113,ENST00000399838,ENST00000455664
ENST00000389554
ENST00000187397,ENST00000337271,ENST00000417925,ENST00000444190,ENST00000458225
ENST00000352105,ENST00000439576,ENST00000559004,ENST00000561243
ENST00000315891,ENST00000346785,ENST00000360370,ENST00000504035,ENST00000505996,ENST00000
ENST00000256552,ENST00000397795,ENST00000542188,ENST00000558445,ENST00000558768,ENST00000
ENST00000296871
ENST00000281928

ENST00000304990,ENST00000396578
ENST00000216500,ENST00000536410,ENST00000557185
ENST00000262963,ENST00000348075,ENST00000353284,ENST00000357368,ENST00000372412,ENST00000
ENST00000271532
ENST00000388726,ENST00000392650,ENST00000424778
ENST00000399799
ENST00000265636,ENST00000265637,ENST00000393799,ENST00000393800,ENST00000393801,ENST00000
ENST00000256737,ENST00000525139,ENST00000531568,ENST00000537978
ENST00000421673
ENST00000368804
ENST00000188403,ENST00000321046,ENST00000392986,ENST00000548884,ENST00000551379
ENST00000380171,ENST00000405489,ENST00000606999
ENST00000319560
ENST00000412074
ENST00000376296
ENST00000344113,ENST00000358025,ENST00000554584
ENST00000361193,ENST00000411502,ENST00000546047,ENST00000571215,ENST00000572725
ENST00000216500,ENST00000536410,ENST00000557185
ENST00000400555,ENST00000400556,ENST00000580745,ENST00000581250,ENST00000582240,ENST00000
ENST00000304990,ENST00000396578
ENST00000256495
ENST00000284476
ENST00000370791,ENST00000443751
ENST00000263087
ENST00000380113,ENST00000399838,ENST00000455664
ENST00000187397,ENST00000337271,ENST00000417925,ENST00000444190,ENST00000458225
ENST00000380255,ENST00000380262,ENST00000563694,ENST00000565740,ENST00000575408,ENST00000
ENST00000217130,ENST00000369888,ENST00000450537
ENST00000412074
ENST00000389554
ENST00000391571
ENST00000315891,ENST00000346785,ENST00000360370,ENST00000504035,ENST00000505996,ENST00000
ENST00000415833,ENST00000424340,ENST00000517539,ENST00000528249
ENST00000388726,ENST00000392650,ENST00000424778
ENST00000281928
ENST00000352105,ENST00000439576,ENST00000559004,ENST00000561243
ENST00000301547
ENST00000262963,ENST00000348075,ENST00000353284,ENST00000357368,ENST00000372412,ENST00000
ENST00000188403,ENST00000321046,ENST00000392986,ENST00000548884,ENST00000551379
ENST00000332509,ENST00000335539,ENST00000402064,ENST00000430886
ENST00000271532
ENST00000296871
ENST00000256552,ENST00000397795,ENST00000542188,ENST00000558445,ENST00000558768,ENST00000
ENST00000399799
ENST00000376296
ENST00000421673
ENST00000265636,ENST00000265637,ENST00000393799,ENST00000393800,ENST00000393801,ENST00000

ENST00000256737,ENST00000525139,ENST00000531568,ENST00000537978
ENST00000268482
ENST00000319560
ENST00000380171,ENST00000405489,ENST00000606999
ENST00000368804
ENST00000291726,ENST00000320838,ENST00000347619,ENST00000391806,ENST00000593490,ENST00000
ENST00000356504,ENST00000381620,ENST00000507069,ENST00000510861,ENST00000514090,ENST00000
ENST00000268482
ENST00000400555,ENST00000400556,ENST00000580745,ENST00000581250,ENST00000582240,ENST00000
ENST00000369345,ENST00000369348,ENST00000369359,ENST00000526359,ENST00000528661,ENST00000
ENST00000207549,ENST00000412096
ENST00000326270,ENST00000343388,ENST00000346027,ENST00000542523,ENST00000581041,ENST00000
ENST00000263087
ENST00000256495
ENST00000344113,ENST00000358025,ENST00000554584
ENST00000380113,ENST00000399838,ENST00000455664
ENST00000279230,ENST00000325234,ENST00000540288
ENST00000187397,ENST00000337271,ENST00000417925,ENST00000444190,ENST00000458225
ENST00000352105,ENST00000439576,ENST00000559004,ENST00000561243
ENST00000315891,ENST00000346785,ENST00000360370,ENST00000504035,ENST00000505996,ENST00000
ENST00000256552,ENST00000397795,ENST00000542188,ENST00000558445,ENST00000558768,ENST00000
ENST00000296871
ENST00000281928
ENST00000304990,ENST00000396578
ENST00000358047
ENST00000454682
ENST00000367482,ENST00000367483,ENST00000367485,ENST00000367486,ENST00000445192
ENST00000375001
ENST00000271532
ENST00000388726,ENST00000392650,ENST00000424778
ENST00000348028,ENST00000349350,ENST00000421100,ENST00000427578,ENST00000443370,ENST00000
ENST00000264436,ENST00000355733,ENST00000403045,ENST00000407644,ENST00000413157,ENST00000
ENST00000399799
ENST00000265636,ENST00000265637,ENST00000393799,ENST00000393800,ENST00000393801,ENST00000
ENST00000368804
ENST00000262462,ENST00000395266,ENST00000506176
ENST00000188403,ENST00000321046,ENST00000392986,ENST00000548884,ENST00000551379
ENST00000380171,ENST00000405489,ENST00000606999
ENST00000319560
ENST00000412074
ENST00000458270,ENST00000501748,ENST00000526926
ENST00000344113,ENST00000358025,ENST00000554584
ENST00000356504,ENST00000381620,ENST00000507069,ENST00000510861,ENST00000514090,ENST00000
ENST00000400555,ENST00000400556,ENST00000580745,ENST00000581250,ENST00000582240,ENST00000
ENST00000304990,ENST00000396578
ENST00000256495
ENST00000291726,ENST00000320838,ENST00000347619,ENST00000391806,ENST00000593490,ENST00000

ENST00000263087
ENST00000380113,ENST00000399838,ENST00000455664
ENST00000264436,ENST00000355733,ENST00000403045,ENST00000407644,ENST00000413157,ENST00000[illegible]
ENST00000262462,ENST00000395266,ENST00000506176
ENST00000187397,ENST00000337271,ENST00000417925,ENST00000444190,ENST00000458225
ENST00000207549,ENST00000412096
ENST00000369345
ENST00000412074
ENST00000315891,ENST00000346785,ENST00000360370,ENST00000504035,ENST00000505996,ENST00000[illegible]
ENST00000326270,ENST00000343388,ENST00000346027,ENST00000542523,ENST00000581041,ENST00000[illegible]
ENST00000388726,ENST00000392650,ENST00000424778
ENST00000279230,ENST00000325234,ENST00000540288
ENST00000281928
ENST00000375001
ENST00000352105,ENST00000439576,ENST00000559004,ENST00000561243
ENST00000458270,ENST00000501748,ENST00000526926
ENST00000358047
ENST00000188403,ENST00000321046,ENST00000392986,ENST00000548884,ENST00000551379
ENST00000271532
ENST00000296871
ENST00000256552,ENST00000397795,ENST00000542188,ENST00000558445,ENST00000558768,ENST00000[illegible]
ENST00000399799
ENST00000265636,ENST00000265637,ENST00000393799,ENST00000393800,ENST00000393801,ENST00000[illegible]
ENST00000268482
ENST00000348028,ENST00000349350,ENST00000421100,ENST00000427578,ENST00000443370,ENST00000[illegible]
ENST00000367482,ENST00000367483,ENST00000367485,ENST00000367486,ENST00000445192
ENST00000319560
ENST00000380171,ENST00000405489,ENST00000606999
ENST00000368804
ENST00000454682
ENST00000357311,ENST00000393651,ENST00000489828
ENST00000159087,ENST00000597643
ENST00000283195
ENST00000315368,ENST00000505991,ENST00000510092
ENST00000324103,ENST00000382687,ENST00000558468,ENST00000559275
ENST00000354905
ENST00000303824,ENST00000340726,ENST00000388740,ENST00000388741,ENST00000388742,ENST00000[illegible]
ENST00000216445,ENST00000422976,ENST00000526336,ENST00000526745,ENST00000529860,ENST00000[illegible]
ENST00000206423,ENST00000439685
ENST00000251761,ENST00000396725
ENST00000334686,ENST00000340059,ENST00000414191,ENST00000460012
ENST00000284669,ENST00000513963,ENST00000554017
ENST00000367104,ENST00000435180
ENST00000233712,ENST00000393913,ENST00000410071,ENST00000410113,ENST00000432649,ENST00000[illegible]
ENST00000339762,ENST00000554729,ENST00000556826
ENST00000277746
ENST00000333646,ENST00000374080,ENST00000374102

ENST00000302303,ENST00000358730,ENST00000382004,ENST00000457373
ENST00000343455,ENST00000393063,ENST00000526495,ENST00000527414,ENST00000541352
ENST00000347108,ENST00000361156,ENST00000361396,ENST00000376829,ENST00000466569
ENST00000194118,ENST00000432126
ENST00000454872
ENST00000357337,ENST00000407578,ENST00000544440
ENST00000394576
ENST00000320129,ENST00000519564
ENST00000287652,ENST00000437082,ENST00000448281
ENST00000328354,ENST00000348295,ENST00000382566,ENST00000382580,ENST00000398017,ENST00000[illegible]
ENST00000316562,ENST00000495692,ENST00000497424,ENST00000610179
ENST00000371621,ENST00000541713
ENST00000301547
ENST00000583868
ENST00000274400,ENST00000330280,ENST00000425596
ENST00000338193,ENST00000549549,ENST00000550770
ENST00000323434,ENST00000331302,ENST00000536489
ENST00000254066,ENST00000394081,ENST00000394086,ENST00000394089,ENST00000425707,ENST00000[illegible]
ENST00000348581,ENST00000366548,ENST00000450748,ENST00000518483
ENST00000320027,ENST00000380405,ENST00000380406,ENST00000429785,ENST00000445273
ENST00000160713,ENST00000276737,ENST00000395297
ENST00000324545,ENST00000378308
ENST00000333188,ENST00000507777
ENST00000278559,ENST00000456580,ENST00000529629
ENST00000286800,ENST00000399921
ENST00000360468,ENST00000430376,ENST00000431652,ENST00000557865,ENST00000559717
ENST00000357409,ENST00000360280,ENST00000376634,ENST00000376636
ENST00000288135,ENST00000412167
ENST00000355202,ENST00000395835,ENST00000590670
ENST00000295903,ENST00000498707
ENST00000335093
ENST00000303824,ENST00000340726,ENST00000388740,ENST00000388741,ENST00000388742,ENST00000[illegible]
ENST00000334686,ENST00000340059,ENST00000414191,ENST00000460012
ENST00000334686,ENST00000340059,ENST00000414191,ENST00000460012
ENST00000334686,ENST00000340059,ENST00000414191,ENST00000460012
ENST00000159087,ENST00000597643
ENST00000360468,ENST00000430376,ENST00000431652,ENST00000557865,ENST00000559717
ENST00000316562,ENST00000495692,ENST00000497424,ENST00000610179
ENST00000324545,ENST00000378308
ENST00000194118,ENST00000432126
ENST00000367101,ENST00000367102
ENST00000357337,ENST00000407578,ENST00000544440
ENST00000554017,ENST00000284669,ENST00000513963
ENST00000160713,ENST00000276737,ENST00000395297
ENST00000160713,ENST00000276737,ENST00000395297
ENST00000160713,ENST00000276737,ENST00000395297
ENST00000251761,ENST00000396725

ENST00000216445,ENST00000422976,ENST00000526336,ENST00000526745,ENST00000530417,ENST00000
ENST00000338193,ENST00000549549,ENST00000550770
ENST00000355202,ENST00000395835,ENST00000590670
ENST00000286800,ENST00000399921
ENST00000347108,ENST00000361156,ENST00000361396,ENST00000376829,ENST00000466569
ENST00000302303,ENST00000358730,ENST00000382004,ENST00000457373
ENST00000433028,ENST00000447421,ENST00000448511
ENST00000357409,ENST00000360280,ENST00000376634,ENST00000376636
ENST00000354905
ENST00000315368,ENST00000505991,ENST00000510092
ENST00000274400,ENST00000330280,ENST00000425596
ENST00000454872
ENST00000357311,ENST00000393651,ENST00000489828
ENST00000283195
ENST00000339762,ENST00000554729,ENST00000556826
ENST00000278559,ENST00000456580,ENST00000529629
ENST00000323434,ENST00000331302,ENST00000536489
ENST00000324103,ENST00000382687,ENST00000559275,ENST00000558468
ENST00000301547
ENST00000254066,ENST00000394081,ENST00000394086,ENST00000394089,ENST00000425707,ENST00000
ENST00000583868
ENST00000320129,ENST00000519564
ENST00000233712,ENST00000393913,ENST00000410071,ENST00000410113,ENST00000432649,ENST00000
ENST00000333646,ENST00000374080,ENST00000374102
ENST00000333188,ENST00000507777
ENST00000206423,ENST00000439685
ENST00000371621,ENST00000541713
ENST00000437082,ENST00000287652,ENST00000448281
ENST00000394576
ENST00000277746
ENST00000343455,ENST00000393063,ENST00000526495,ENST00000527414,ENST00000541352
ENST00000295903,ENST00000498707
ENST00000288135,ENST00000412167
ENST00000320027,ENST00000380405,ENST00000380406,ENST00000429785,ENST00000445273
ENST00000348581,ENST00000366548,ENST00000450748,ENST00000518483
ENST00000335093
ENST00000260569
ENST00000263045,ENST00000354620,ENST00000371159,ENST00000393666,ENST00000423399,ENST00000
ENST00000258770,ENST00000494076
ENST00000332441,ENST00000361109,ENST00000376625,ENST00000389560,ENST00000392352,ENST00000
ENST00000375592,ENST00000444116,ENST00000456164
ENST00000295956,ENST00000348383,ENST00000357272,ENST00000358537,ENST00000419752,ENST00000
ENST00000278882,ENST00000358464,ENST00000439954
ENST00000358731
ENST00000244891,ENST00000449290
ENST00000339803,ENST00000379808,ENST00000523826,ENST00000524140,ENST00000549814,ENST00000
ENST00000351839,ENST00000360384,ENST00000376263,ENST00000376264,ENST00000376281,ENST00000

ENST00000375654,ENST00000417199
ENST00000339488
ENST00000339865,ENST00000399455,ENST00000527733
ENST00000368114,ENST00000368115
ENST00000279477,ENST00000562014,ENST00000568365
ENST00000288699,ENST00000401478
ENST00000378867,ENST00000527487,ENST00000530639
ENST00000595793
ENST00000268720
ENST00000309890,ENST00000352011,ENST00000359983,ENST00000397035,ENST00000397041,ENST00000
ENST00000304735
ENST00000375442
ENST00000286485,ENST00000327040,ENST00000428502,ENST00000440756,ENST00000523619
ENST00000309863,ENST00000409896
ENST00000342595,ENST00000378220
ENST00000328333,ENST00000454817
ENST00000265838,ENST00000299355,ENST00000527942,ENST00000531813
ENST00000264122,ENST00000405772
ENST00000304735
ENST00000258770,ENST00000494076
ENST00000288699,ENST00000401478
ENST00000375442
ENST00000377990,ENST00000537589,ENST00000546106
ENST00000286485,ENST00000327040,ENST00000428502,ENST00000440756,ENST00000523619
ENST00000309890,ENST00000352011,ENST00000359983,ENST00000397035,ENST00000397041,ENST00000
ENST00000378867,ENST00000527487,ENST00000530639
ENST00000339488
ENST00000368114,ENST00000368115
ENST00000244891,ENST00000449290
ENST00000339865,ENST00000399455,ENST00000527733
ENST00000332441,ENST00000361109,ENST00000376625,ENST00000389560,ENST00000392352,ENST00000
ENST00000295956,ENST00000348383,ENST00000357272,ENST00000358537,ENST00000419752,ENST00000
ENST00000309863,ENST00000409896
ENST00000268720
ENST00000375654,ENST00000417199
ENST00000375592,ENST00000444116,ENST00000456164
ENST00000457156
ENST00000278882,ENST00000358464,ENST00000439954,ENST00000479318
ENST00000263045,ENST00000354620,ENST00000371159,ENST00000393666,ENST00000423399,ENST00000
ENST00000328333,ENST00000454817
ENST00000339803,ENST00000379808,ENST00000523826,ENST00000524140,ENST00000549814,ENST00000
ENST00000279477,ENST00000562014,ENST00000568365
ENST00000358731
ENST00000595793
ENST00000265838,ENST00000299355,ENST00000531813
ENST00000342595,ENST00000378220
ENST00000264122,ENST00000405772

ENST00000281437
ENST00000265052,ENST00000398101,ENST00000398104,ENST00000434178,ENST00000453507,ENST000004
ENST00000308664,ENST00000395494,ENST00000539276
ENST00000358970
ENST00000265056
ENST00000288561,ENST00000473989
ENST00000393923,ENST00000393925,ENST00000412622,ENST00000431670,ENST00000477695,ENST000004
ENST00000290239,ENST00000300278,ENST00000356577,ENST00000381679,ENST00000455528
ENST00000293879,ENST00000315764,ENST00000547944,ENST00000549091
ENST00000303635,ENST00000439411
ENST00000265052,ENST00000398101,ENST00000398104,ENST00000434178,ENST00000453507,ENST000004
ENST00000308664,ENST00000395494,ENST00000539276
ENST00000281437
ENST00000393923,ENST00000393925,ENST00000412622,ENST00000431670,ENST00000477695,ENST000004
ENST00000288561,ENST00000473989
ENST00000265056
ENST00000290239,ENST00000300278,ENST00000356577,ENST00000381679,ENST00000455528
ENST00000293879,ENST00000315764,ENST00000547944,ENST00000549091
ENST00000358970
ENST00000303635,ENST00000439411
ENST00000278886,ENST00000422516
ENST00000285279,ENST00000414113,ENST00000417399,ENST00000418904,ENST00000427700,ENST000004
ENST00000354197
ENST00000303354,ENST00000375387,ENST00000409435,ENST00000409672
ENST00000379655,ENST00000421376,ENST00000445690,ENST00000465878,ENST00000539578,ENST000005
ENST00000296474,ENST00000344206,ENST00000411578
ENST00000261693,ENST00000339570,ENST00000376788,ENST00000415380,ENST00000540495,ENST000005
ENST00000368057,ENST00000368059
ENST00000283351,ENST00000582539
ENST00000264938,ENST00000514375
ENST00000382584
ENST00000306317,ENST00000424267,ENST00000517694
ENST00000305202
ENST00000343776,ENST00000397097,ENST00000440339
ENST00000300619,ENST00000397082
ENST00000319004,ENST00000576778
ENST00000204801,ENST00000301357,ENST00000315591,ENST00000315599,ENST00000354397,ENST000005
ENST00000298198
ENST00000333942,ENST00000354266,ENST00000358365,ENST00000394692,ENST00000545287,ENST000005
ENST00000327454,ENST00000378174,ENST00000505415,ENST00000539424
ENST00000371741
ENST00000240851,ENST00000418917,ENST00000463568,ENST00000476228,ENST00000479672,ENST000004
ENST00000409389,ENST00000428135
ENST00000369219
ENST00000356929
ENST00000439326
ENST00000397126

ENST00000427401,ENST00000594419
ENST00000356929
ENST00000251071,ENST00000298892,ENST00000308433,ENST00000395805,ENST00000417045,ENST00000
ENST00000376463,ENST00000376499,ENST00000418319
ENST00000274054,ENST00000422287
ENST00000357443,ENST00000369644,ENST00000369645,ENST00000413052
ENST00000344980,ENST00000419886
ENST00000231021
ENST00000397104,ENST00000596209
ENST00000397121
ENST00000334181
ENST00000368488,ENST00000368491
ENST00000251071,ENST00000298892,ENST00000308433,ENST00000395805,ENST00000417045,ENST00000
ENST00000356929
ENST00000378438,ENST00000436783,ENST00000535539,ENST00000535777
ENST00000339241,ENST00000394339,ENST00000507703
ENST00000260595,ENST00000312734,ENST00000402394,ENST00000404694,ENST00000405600
ENST00000344363
ENST00000328405,ENST00000452398
ENST00000597761
ENST00000377386,ENST00000398263,ENST00000409015,ENST00000409232,ENST00000444342
ENST00000391413
ENST00000394456,ENST00000429701,ENST00000593678
ENST00000408964
ENST00000285279,ENST00000414113,ENST00000417399,ENST00000418904,ENST00000427700,ENST00000
ENST00000427401,ENST00000594419
ENST00000409389,ENST00000428135
ENST00000357443,ENST00000369644,ENST00000369645,ENST00000413052
ENST00000264938,ENST00000514375
ENST00000334181
ENST00000240851,ENST00000418917,ENST00000463568,ENST00000476228,ENST00000479672,ENST00000
ENST00000344363
ENST00000339241,ENST00000394339,ENST00000507703
ENST00000278886,ENST00000422516
ENST00000378438,ENST00000436783,ENST00000535539,ENST00000535777
ENST00000397121
ENST00000397126
ENST00000439326
ENST00000328405,ENST00000452398
ENST00000204801,ENST00000301357,ENST00000315591,ENST00000315599,ENST00000354397,ENST00000
ENST00000300619,ENST00000397082
ENST00000327454,ENST00000378174,ENST00000505415,ENST00000539424
ENST00000397104,ENST00000596209
ENST00000261693,ENST00000339570,ENST00000376788,ENST00000415380,ENST00000540495,ENST00000
ENST00000251071,ENST00000298892,ENST00000308433,ENST00000395805,ENST00000417045,ENST00000
ENST00000296474,ENST00000344206,ENST00000411578
ENST00000368057,ENST00000368059

ENST00000344980,ENST00000419886
ENST00000221735,ENST00000347466,ENST00000415379,ENST00000424930,ENST00000426954,ENST00000
ENST00000303354,ENST00000375387,ENST00000409435,ENST00000409672
ENST00000274054,ENST00000422287
ENST00000356929
ENST00000382584
ENST00000305202
ENST00000333942,ENST00000354266,ENST00000358365,ENST00000394692,ENST00000545287,ENST00000
ENST00000356929
ENST00000343776,ENST00000397097
ENST00000231021
ENST00000369219
ENST00000283351,ENST00000582539
ENST00000597761
ENST00000377386,ENST00000398263,ENST00000409015,ENST00000409232,ENST00000444342
ENST00000376463,ENST00000376499,ENST00000418319
ENST00000371741
ENST00000356929
ENST00000306317,ENST00000424267,ENST00000517694
ENST00000319004,ENST00000576778
ENST00000394456,ENST00000429701,ENST00000593678
ENST00000260595,ENST00000312734,ENST00000402394,ENST00000404694,ENST00000405600
ENST00000379655,ENST00000421376,ENST00000445690,ENST00000465878,ENST00000539578,ENST00000
ENST00000298198
ENST00000368488,ENST00000368491
ENST00000408964
ENST00000391413
ENST00000266483
ENST00000297826,ENST00000374469,ENST00000401783
ENST00000285279,ENST00000414113,ENST00000417399,ENST00000418904,ENST00000427700,ENST00000
ENST00000329322,ENST00000379946
ENST00000358746
ENST00000371030
ENST00000303071,ENST00000303113,ENST00000432378,ENST00000453626
ENST00000285900,ENST00000340592,ENST00000448073,ENST00000518142,ENST00000518783,ENST00000
ENST00000328600
ENST00000328381
ENST00000264183,ENST00000349213,ENST00000366603,ENST00000418304,ENST00000421364
ENST00000005226
ENST00000306317,ENST00000424267,ENST00000517694
ENST00000265727,ENST00000315984,ENST00000398201,ENST00000398203,ENST00000398204,ENST00000
ENST00000324048,ENST00000336715,ENST00000368879,ENST00000449313
ENST00000269080,ENST00000430352,ENST00000586539
ENST00000240851,ENST00000418917,ENST00000463568,ENST00000476228,ENST00000479672,ENST00000
ENST00000242804,ENST00000424168,ENST00000438182
ENST00000427522,ENST00000467148
ENST00000335281,ENST00000553432,ENST00000554736,ENST00000555472,ENST00000556752,ENST00000

ENST00000344119,ENST00000371506,ENST00000472938
ENST00000357115,ENST00000510413,ENST00000535741
ENST00000264552,ENST00000587845,ENST00000589978
ENST00000242804,ENST00000424168,ENST00000438182
ENST00000449082
ENST00000587639,ENST00000589061,ENST00000589103,ENST00000590643
ENST00000326587,ENST00000375695,ENST00000375722,ENST00000375772
ENST00000303961,ENST00000406058,ENST00000593726,ENST00000593972,ENST00000598654,ENST00000
ENST00000367184
ENST00000273666,ENST00000471262,ENST00000471454
ENST00000260130,ENST00000413219,ENST00000422546,ENST00000424270,ENST00000447182,ENST00000
ENST00000294256,ENST00000307289,ENST00000377190,ENST00000526060
ENST00000435989
ENST00000391413
ENST00000320246,ENST00000373596,ENST00000373600,ENST00000373603,ENST00000394199,ENST00000
ENST00000303354,ENST00000375387,ENST00000409435,ENST00000409672
ENST00000286091,ENST00000413966
ENST00000391413
ENST00000427522,ENST00000467148
ENST00000285279,ENST00000414113,ENST00000417399,ENST00000418904,ENST00000427700,ENST00000
ENST00000005226
ENST00000303071,ENST00000303113,ENST00000432378,ENST00000453626
ENST00000240851,ENST00000418917,ENST00000463568,ENST00000476228,ENST00000479672,ENST00000
ENST00000285900,ENST00000340592,ENST00000448073,ENST00000518142,ENST00000518783,ENST00000
ENST00000358746
ENST00000264183,ENST00000349213,ENST00000366603,ENST00000418304,ENST00000421364
ENST00000329322,ENST00000379946
ENST00000371030
ENST00000328600
ENST00000449082
ENST00000315984
ENST00000294256,ENST00000307289,ENST00000377190,ENST00000526060
ENST00000297826,ENST00000374469,ENST00000401783
ENST00000266483
ENST00000242804,ENST00000424168,ENST00000438182,ENST00000242804,ENST00000424168,ENST00000
ENST00000367184
ENST00000435989
ENST00000371506,ENST00000472938
ENST00000326587,ENST00000375695,ENST00000375722,ENST00000375772
ENST00000587639,ENST00000589061,ENST00000589103,ENST00000590643
ENST00000269080,ENST00000430352,ENST00000586539
ENST00000306317,ENST00000424267,ENST00000517694
ENST00000273666,ENST00000471262,ENST00000471454
ENST00000303354,ENST00000375387,ENST00000409435,ENST00000409672
ENST00000357115,ENST00000508606,ENST00000509835,ENST00000510413,ENST00000535741
ENST00000320246,ENST00000373596,ENST00000373600,ENST00000373603,ENST00000394199,ENST00000
ENST00000328381

ENST00000260130,ENST00000413219,ENST00000422546,ENST00000424270,ENST00000447182,ENST00000[illegible]
ENST00000335281,ENST00000553432,ENST00000554736,ENST00000555472,ENST00000556752,ENST00000[illegible]
ENST00000303961,ENST00000406058,ENST00000593726,ENST00000593972,ENST00000594380,ENST00000[illegible]
ENST00000324048,ENST00000336715,ENST00000368879,ENST00000449313
ENST00000391413
ENST00000264552,ENST00000587845,ENST00000589978
ENST00000391413
ENST00000286091,ENST00000413966
ENST00000348028,ENST00000349350,ENST00000421100,ENST00000427578,ENST00000443370,ENST00000[illegible]
ENST00000287380,ENST00000309336,ENST00000327098,ENST00000378080,ENST00000518805,ENST00000[illegible]
ENST00000328354,ENST00000348295,ENST00000382578,ENST00000382580,ENST00000402731,ENST00000[illegible]
ENST00000375871
ENST00000256646
ENST00000344859,ENST00000362074,ENST00000369340,ENST00000468030
ENST00000380152,ENST00000544455
ENST00000361899
ENST00000287380,ENST00000309336,ENST00000327098,ENST00000378080,ENST00000518805,ENST00000[illegible]
ENST00000348028,ENST00000349350,ENST00000421100,ENST00000427578,ENST00000443370,ENST00000[illegible]
ENST00000328354,ENST00000348295,ENST00000382578,ENST00000382580,ENST00000402731,ENST00000[illegible]
ENST00000361899
ENST00000256646
ENST00000325303,ENST00000336654,ENST00000375883,ENST00000605159
ENST00000380152,ENST00000544455
ENST00000344859,ENST00000362074,ENST00000369340,ENST00000468030
ENST00000361543,ENST00000361781
ENST00000315870,ENST00000431467
ENST00000338949,ENST00000343253,ENST00000401026,ENST00000455267,ENST00000557479
ENST00000343769
ENST00000399686,ENST00000413704
ENST00000310346,ENST00000349716,ENST00000405440,ENST00000526441
ENST00000283415,ENST00000475622
ENST00000378832,ENST00000527145
ENST00000391571
ENST00000433653,ENST00000486074
ENST00000356287,ENST00000405093
ENST00000322954,ENST00000379983,ENST00000539319,ENST00000560441
ENST00000361505
ENST00000361538,ENST00000380118,ENST00000380125,ENST00000413766,ENST00000465828
ENST00000355636
ENST00000261716,ENST00000536202
ENST00000261716,ENST00000536202
ENST00000311172,ENST00000409263,ENST00000409314,ENST00000409418,ENST00000458644
ENST00000297540
ENST00000358047
ENST00000272879,ENST00000286186,ENST00000313728,ENST00000346817,ENST00000360132,ENST00000[illegible]
ENST00000343769
ENST00000361505

ENST00000378832,ENST00000527145
ENST00000283415,ENST00000475622
ENST00000391571
ENST00000338949,ENST00000343253,ENST00000401026,ENST00000455267,ENST00000557479
ENST00000261716,ENST00000536202,ENST00000261716,ENST00000536202
ENST00000361543,ENST00000361781
ENST00000315870,ENST00000431467
ENST00000361538,ENST00000380118,ENST00000380125,ENST00000413766,ENST00000465828
ENST00000310346,ENST00000349716,ENST00000405440,ENST00000526441
ENST00000373880
ENST00000302463,ENST00000399686,ENST00000402198,ENST00000402466,ENST00000406341,ENST00000
ENST00000355636
ENST00000322954,ENST00000379983,ENST00000539319,ENST00000560441
ENST00000297540
ENST00000356287,ENST00000405093
ENST00000358047
ENST00000311172,ENST00000409263,ENST00000409314,ENST00000409418,ENST00000458644
ENST00000272879,ENST00000286186,ENST00000313728,ENST00000346817,ENST00000360132,ENST00000
ENST00000266505,ENST00000435379,ENST00000447925,ENST00000534932,ENST00000538330,ENST00000
ENST00000282701
ENST00000255674,ENST00000437017,ENST00000454359,ENST00000583043
ENST00000230173,ENST00000296932,ENST00000367608,ENST00000367609
ENST00000263923
ENST00000373563,ENST00000373566,ENST00000409196,ENST00000409451,ENST00000409480,ENST00000
ENST00000368096
ENST00000307662,ENST00000394243,ENST00000519664,ENST00000522122
ENST00000313382,ENST00000369354,ENST00000369356,ENST00000369359,ENST00000530740
ENST00000531461
ENST00000335750,ENST00000554719,ENST00000554738
ENST00000578026
ENST00000369950,ENST00000369963
ENST00000273432,ENST00000474524
ENST00000371566,ENST00000371568
ENST00000572129
ENST00000282499,ENST00000393127,ENST00000525187,ENST00000530497
ENST00000264661,ENST00000607371
ENST00000263798,ENST00000559066
ENST00000360870
ENST00000376048
ENST00000290866,ENST00000428043
ENST00000360133,ENST00000443188,ENST00000589093,ENST00000589824,ENST00000592953
ENST00000261833,ENST00000392521
ENST00000379407,ENST00000379409,ENST00000379410
ENST00000240189
ENST00000328354,ENST00000348295,ENST00000382578,ENST00000382580,ENST00000402731,ENST00000
ENST00000455694
ENST00000284154,ENST00000395635

ENST00000318682,ENST00000504597,ENST00000510660
ENST00000255674,ENST00000437017,ENST00000454359,ENST00000583043
ENST00000264661,ENST00000607371
ENST00000368096
ENST00000360133,ENST00000443188,ENST00000589093,ENST00000589824,ENST00000592953
ENST00000371566,ENST00000371568
ENST00000282499,ENST00000393127,ENST00000525187,ENST00000530497
ENST00000335750,ENST00000554719,ENST00000554738
ENST00000261833,ENST00000392521
ENST00000273432,ENST00000474524
ENST00000531461
ENST00000263923
ENST00000263798,ENST00000559066
ENST00000284154,ENST00000395635
ENST00000369950
ENST00000373563,ENST00000373566,ENST00000409196,ENST00000409451,ENST00000409480,ENST00000[illegible]
ENST00000578026
ENST00000240189
ENST00000230173,ENST00000296932,ENST00000367608,ENST00000367609
ENST00000328354,ENST00000348295,ENST00000382578,ENST00000382580,ENST00000402731,ENST00000[illegible]
ENST00000282701
ENST00000266505,ENST00000435379,ENST00000447925,ENST00000534932,ENST00000538330,ENST00000[illegible]
ENST00000313382,ENST00000369354,ENST00000369356,ENST00000369359,ENST00000530740
ENST00000290866,ENST00000428043
ENST00000360870
ENST00000455694
ENST00000572129
ENST00000307662,ENST00000394243,ENST00000519664,ENST00000522122
ENST00000379407,ENST00000379409,ENST00000379410
ENST00000376048
ENST00000318682,ENST00000504597,ENST00000510660
ENST00000323022,ENST00000376251,ENST00000376265
ENST00000357438
ENST00000221265,ENST00000221266,ENST00000416728,ENST00000595564
ENST00000399080
ENST00000316707,ENST00000411767,ENST00000427191,ENST00000436978,ENST00000511467
ENST00000361735,ENST00000362019,ENST00000367737,ENST00000458517
ENST00000348165,ENST00000389103
ENST00000320574,ENST00000535270,ENST00000537064
ENST00000320934
ENST00000337990,ENST00000374839,ENST00000447788,ENST00000545618
ENST00000329800,ENST00000330675,ENST00000358024,ENST00000545499
ENST00000261726
ENST00000255416
ENST00000359428,ENST00000451030
ENST00000314595,ENST00000381227,ENST00000431623,ENST00000441246
ENST00000302196,ENST00000378993

ENST00000339901,ENST00000375807,ENST00000534113
ENST00000296658
ENST00000399080
ENST00000329800,ENST00000330675,ENST00000358024,ENST00000545499
ENST00000323022,ENST00000376251,ENST00000376265
ENST00000255416
ENST00000261726
ENST00000320934
ENST00000320574,ENST00000535270,ENST00000537064,ENST00000539006
ENST00000359428,ENST00000451030
ENST00000221265,ENST00000221266,ENST00000416728,ENST00000595564
ENST00000357438
ENST00000314595,ENST00000381227,ENST00000431623,ENST00000441246
ENST00000348165,ENST00000389103
ENST00000316707,ENST00000411767,ENST00000427191,ENST00000436978,ENST00000511467
ENST00000361735,ENST00000362019,ENST00000367737,ENST00000458517
ENST00000337990,ENST00000374839,ENST00000447788,ENST00000545618
ENST00000339901,ENST00000375807,ENST00000534113
ENST00000302196,ENST00000378993
ENST00000296658
ENST00000297404,ENST00000524391
ENST00000367482,ENST00000367483,ENST00000367485,ENST00000367486,ENST00000445192
ENST00000492446
ENST00000353801,ENST00000371554,ENST00000371646
ENST00000306151,ENST00000379439
ENST00000263413,ENST00000337836
ENST00000370565
ENST00000240189
ENST00000337057,ENST00000368128,ENST00000524581,ENST00000527411,ENST00000529208
ENST00000437584,ENST00000535591
ENST00000448514,ENST00000480559
ENST00000306858
ENST00000258729
ENST00000371477
ENST00000452611
ENST00000268624,ENST00000315906
ENST00000412090,ENST00000412090
ENST00000240189
ENST00000445890,ENST00000528282
ENST00000437584,ENST00000535591
ENST00000258729
ENST00000492446
ENST00000371477
ENST00000448514,ENST00000480559
ENST00000268624,ENST00000315906
ENST00000353801,ENST00000371554,ENST00000371646
ENST00000297404,ENST00000524391

ENST00000306151,ENST00000379439
ENST00000306858
ENST00000370565
ENST00000263413,ENST00000337836
ENST00000452611
ENST00000367482,ENST00000367483,ENST00000367485,ENST00000367486,ENST00000445192
ENST00000412090
ENST00000541274
ENST00000361217,ENST00000368646
ENST00000266509,ENST00000381552,ENST00000545102,ENST00000545604
ENST00000369338
ENST00000454682
ENST00000257622,ENST00000358357,ENST00000395270,ENST00000434423,ENST00000446813
ENST00000342947,ENST00000409775,ENST00000409970,ENST00000419307,ENST00000545231
ENST00000425945
ENST00000376767
ENST00000437584,ENST00000535591
ENST00000361229,ENST00000372735,ENST00000447531,ENST00000448867,ENST00000457056
ENST00000346061,ENST00000418749
ENST00000295321,ENST00000409725,ENST00000455721
ENST00000257622,ENST00000358357,ENST00000395270,ENST00000434423,ENST00000446813
ENST00000342947,ENST00000409775,ENST00000409970,ENST00000419307,ENST00000545231
ENST00000454682
ENST00000361217,ENST00000368646
ENST00000266509,ENST00000381552,ENST00000545102,ENST00000545604
ENST00000437584,ENST00000535591
ENST00000369338
ENST00000425945
ENST00000376767
ENST00000541274
ENST00000361229,ENST00000372735,ENST00000447531,ENST00000448867,ENST00000457056
ENST00000295321,ENST00000409725,ENST00000455721
ENST00000346061,ENST00000418749
ENST00000346753,ENST00000381792,ENST00000406725,ENST00000406856,ENST00000423761,ENST00000[illegible]
ENST00000322569,ENST00000394182,ENST00000548629
ENST00000262189,ENST00000355193,ENST00000558084
ENST00000304820
ENST00000244336
ENST00000279575,ENST00000535904
ENST00000252723
ENST00000314756,ENST00000393020,ENST00000429951,ENST00000442859,ENST00000455306,ENST00000[illegible]
ENST00000266718
ENST00000356025,ENST00000358771,ENST00000400068
ENST00000279575,ENST00000535904
ENST00000298396,ENST00000376895
ENST00000266718
ENST00000304820

ENST00000346753,ENST00000381792,ENST00000406725,ENST00000406856,ENST00000423761,ENST00000[illegible]
ENST00000252723
ENST00000262189,ENST00000355193,ENST00000558084
ENST00000314756,ENST00000393020,ENST00000429951,ENST00000442859,ENST00000455306,ENST00000[illegible]
ENST00000322569,ENST00000394182,ENST00000548629
ENST00000356025,ENST00000358771,ENST00000400068
ENST00000298396,ENST00000376895
ENST00000244336
ENST00000279575,ENST00000535904,ENST00000279575,ENST00000535904
ENST00000374672
ENST00000351698
ENST00000360439,ENST00000394947,ENST00000394949
ENST00000293851,ENST00000570702
ENST00000287936,ENST00000343975,ENST00000511206,ENST00000511986
ENST00000368081,ENST00000477338
ENST00000262032,ENST00000431367,ENST00000547167,ENST00000547791
ENST00000234420,ENST00000538136,ENST00000540021
ENST00000284674
ENST00000326181
ENST00000361351,ENST00000395081,ENST00000564488,ENST00000565130,ENST00000565335,ENST00000[illegible]
ENST00000279573,ENST00000381842,ENST00000538488
ENST00000412585,ENST00000434333
ENST00000390658
ENST00000266509,ENST00000381552,ENST00000540354,ENST00000545102,ENST00000545604
ENST00000272203,ENST00000414478
ENST00000407184
ENST00000371117
ENST00000309263,ENST00000457346,ENST00000520467
ENST00000261292,ENST00000577628,ENST00000580036
ENST00000361351,ENST00000395081,ENST00000564488,ENST00000565335,ENST00000565494,ENST00000[illegible]
ENST00000284674
ENST00000287936,ENST00000343975,ENST00000511206,ENST00000511986
ENST00000326181
ENST00000261292,ENST00000577628,ENST00000580036
ENST00000390658
ENST00000272203,ENST00000414478
ENST00000266509,ENST00000381552,ENST00000540354,ENST00000545102,ENST00000545604
ENST00000371117
ENST00000412585,ENST00000434333
ENST00000293851,ENST00000570702
ENST00000360439,ENST00000394947,ENST00000394949
ENST00000234420,ENST00000538136,ENST00000540021
ENST00000351698
ENST00000351698
ENST00000368081,ENST00000477338
ENST00000407184
ENST00000309263,ENST00000457346,ENST00000520467

ENST00000262032,ENST00000431367,ENST00000547167,ENST00000547791
ENST00000374672
ENST00000279573,ENST00000381842,ENST00000538488
ENST00000389156,ENST00000513277
ENST00000276449
ENST00000309838
ENST00000379388
ENST00000338305
ENST00000395925
ENST00000259037,ENST00000359944,ENST00000468210,ENST00000472629,ENST00000493866
ENST00000314595,ENST00000381227,ENST00000431623,ENST00000441246
ENST00000519479
ENST00000343118,ENST00000380618,ENST00000380620,ENST00000398714
ENST00000218507,ENST00000372926,ENST00000372927,ENST00000423383
ENST00000358906,ENST00000538780
ENST00000380151
ENST00000338305
ENST00000338305
ENST00000338305
ENST00000338305
ENST00000259037,ENST00000359944,ENST00000468210,ENST00000472629,ENST00000493866
ENST00000519479
ENST00000358906,ENST00000538780
ENST00000314595,ENST00000381227,ENST00000431623,ENST00000441246
ENST00000389156
ENST00000276449
ENST00000380151
ENST00000218507,ENST00000372926,ENST00000372927,ENST00000423383
ENST00000309838
ENST00000395925
ENST00000379388
ENST00000343118,ENST00000380618,ENST00000380620,ENST00000398714
ENST00000310260,ENST00000355485,ENST00000369509,ENST00000369510
ENST00000316770
ENST00000334961,ENST00000338641,ENST00000353887,ENST00000361166,ENST00000361452,ENST00000[illegible]
ENST00000412468
ENST00000313401
ENST00000359865
ENST00000330854,ENST00000337018,ENST00000452918,ENST00000456085,ENST00000594773,ENST00000[illegible]
ENST00000396073
ENST00000335257,ENST00000581322
ENST00000390613
ENST00000333244
ENST00000380206
ENST00000378024
ENST00000547628,ENST00000602107
ENST00000334163,ENST00000369294

ENST00000306151,ENST00000379439
ENST00000266671,ENST00000602540
ENST00000578713,ENST00000584755
ENST00000336592,ENST00000371322,ENST00000404115
ENST00000306908,ENST00000523428
ENST00000389467,ENST00000446231
ENST00000316073
ENST00000368804
ENST00000221214,ENST00000456763,ENST00000457542,ENST00000505373,ENST00000510535,ENST00000
ENST00000221214,ENST00000456763,ENST00000457542,ENST00000505373,ENST00000510535,ENST00000
ENST00000330854,ENST00000337018,ENST00000452918,ENST00000456085,ENST00000594773,ENST00000
ENST00000412468
ENST00000306908,ENST00000523428
ENST00000316770
ENST00000310260,ENST00000355485,ENST00000369509,ENST00000369510
ENST00000380206
ENST00000578713,ENST00000584755
ENST00000547628,ENST00000602107
ENST00000333244
ENST00000335257,ENST00000581322
ENST00000334961,ENST00000338641,ENST00000353887,ENST00000361166,ENST00000361452,ENST00000
ENST00000313401
ENST00000334163,ENST00000369294
ENST00000378024
ENST00000390613
ENST00000266671,ENST00000602540
ENST00000359865
ENST00000336592,ENST00000371322,ENST00000404115
ENST00000306151,ENST00000379439
ENST00000396073
ENST00000368804
ENST00000389467,ENST00000446231
ENST00000316073
ENST00000395385,ENST00000402377
ENST00000334063
ENST00000205890
ENST00000333535,ENST00000413689,ENST00000414099,ENST00000423572,ENST00000425664,ENST00000
ENST00000278520,ENST00000423339,ENST00000542662
ENST00000342386,ENST00000375940,ENST00000375946
ENST00000335552
ENST00000320892
ENST00000303843,ENST00000357277
ENST00000382975,ENST00000382977,ENST00000382978,ENST00000400103,ENST00000400230,ENST00000
ENST00000368005,ENST00000368006,ENST00000392188,ENST00000458050,ENST00000464113,ENST00000
ENST00000293725
ENST00000479318
ENST00000276202,ENST00000276204

ENST00000318217,ENST00000394649,ENST00000579584
ENST00000596143,ENST00000600435
ENST00000409088,ENST00000409093,ENST00000409282,ENST00000409884,ENST00000409911
ENST00000199814,ENST00000447771,ENST00000540000
ENST00000361727
ENST00000372492,ENST00000528956
ENST00000318220,ENST00000318249,ENST00000342104,ENST00000434838,ENST00000454000
ENST00000283943,ENST00000389044,ENST00000409677,ENST00000428959,ENST00000430954,ENST00000
ENST00000264895
ENST00000342292
ENST00000349310,ENST00000402615,ENST00000407796,ENST00000554581,ENST00000554848,ENST00000
ENST00000307731,ENST00000368932
ENST00000278882,ENST00000358464,ENST00000439954
ENST00000233668,ENST00000409429
ENST00000288025,ENST00000570293
ENST00000525964,ENST00000534532,ENST00000534548
ENST00000323348,ENST00000409849,ENST00000410034
ENST00000418967,ENST00000435122
ENST00000326181
ENST00000316073
ENST00000373695,ENST00000373696
ENST00000388718
ENST00000306591,ENST00000322914,ENST00000322933,ENST00000392467,ENST00000589553,ENST00000
ENST00000369784,ENST00000487146,ENST00000602849
ENST00000344293
ENST00000349310,ENST00000402615,ENST00000407796,ENST00000554581,ENST00000554848,ENST00000
ENST00000303843,ENST00000357277
ENST00000333535,ENST00000413689,ENST00000414099,ENST00000423572,ENST00000425664,ENST00000
ENST00000278882,ENST00000358464,ENST00000439954,ENST00000479318,ENST00000278882,ENST00000
ENST00000323348,ENST00000409849,ENST00000410034
ENST00000382975,ENST00000382977,ENST00000382978,ENST00000400103,ENST00000400230,ENST00000
ENST00000372492,ENST00000528956
ENST00000318217,ENST00000394649,ENST00000579584
ENST00000368005,ENST00000368006,ENST00000392188,ENST00000458050,ENST00000464113,ENST00000
ENST00000418967,ENST00000435122
ENST00000320892
ENST00000293725
ENST00000335552
ENST00000409088,ENST00000409093,ENST00000409282,ENST00000409884,ENST00000409911,ENST00000
ENST00000283943,ENST00000389044,ENST00000409677,ENST00000428959,ENST00000435716,ENST00000
ENST00000326181
ENST00000395385,ENST00000402377
ENST00000342292
ENST00000306591,ENST00000322914,ENST00000322933,ENST00000392467,ENST00000589553,ENST00000
ENST00000199814,ENST00000447771,ENST00000540000
ENST00000525964,ENST00000534532,ENST00000534548
ENST00000307731,ENST00000368932

ENST00000276202,ENST00000276204
ENST00000334063
ENST00000344293
ENST00000342386,ENST00000375940,ENST00000375946
ENST00000233668,ENST00000409429
ENST00000361727
ENST00000205890
ENST00000388718
ENST00000570293,ENST00000288025
ENST00000264895
ENST00000278520,ENST00000423339,ENST00000542662
ENST00000596143,ENST00000600435
ENST00000318220,ENST00000318249,ENST00000342104,ENST00000434838,ENST00000454000
ENST00000316073
ENST00000369784,ENST00000487146,ENST00000602849
ENST00000373695,ENST00000373696
ENST00000284061,ENST00000572810
ENST00000304992,ENST00000572621,ENST00000577001
ENST00000371466,ENST00000371470
ENST00000369108,ENST00000392870
ENST00000526538
ENST00000439326
ENST00000262215,ENST00000520381
ENST00000355650,ENST00000589717,ENST00000592725
ENST00000358933
ENST00000355650,ENST00000589717,ENST00000592725
ENST00000368804
ENST00000340385
ENST00000371121,ENST00000371122,ENST00000371123
ENST00000283243,ENST00000392771
ENST00000334121,ENST00000380918
ENST00000373695,ENST00000373696
ENST00000526538
ENST00000371121,ENST00000371122,ENST00000371123
ENST00000304992,ENST00000572621,ENST00000577001
ENST00000355650,ENST00000589717,ENST00000592725,ENST00000355650,ENST00000589717,ENST00000[illegible]
ENST00000371466,ENST00000371470
ENST00000439326
ENST00000283243,ENST00000392771
ENST00000334121,ENST00000380918
ENST00000262215,ENST00000520381
ENST00000358933
ENST00000284061,ENST00000572810
ENST00000340385
ENST00000369108,ENST00000392870
ENST00000373695,ENST00000373696
ENST00000368804

ENST00000547628,ENST00000602107
ENST00000340781,ENST00000367134,ENST00000367135
ENST00000358097,ENST00000433992
ENST00000306151,ENST00000379439
ENST00000356508,ENST00000409281,ENST00000409419,ENST00000409587,ENST00000409735
ENST00000320078,ENST00000401740,ENST00000593983,ENST00000598133
ENST00000320743,ENST00000379141,ENST00000433272,ENST00000552267
ENST00000529911
ENST00000361367
ENST00000374024
ENST00000371764
ENST00000290209,ENST00000354181,ENST00000397702,ENST00000397707,ENST00000451844,ENST000004
ENST00000290239,ENST00000300278,ENST00000356577,ENST00000381679,ENST00000455528
ENST00000367848
ENST00000288350,ENST00000358268,ENST00000380671
ENST00000263816,ENST00000443831
ENST00000263434,ENST00000376350
ENST00000262836,ENST00000360648,ENST00000372007,ENST00000372010,ENST00000417227,ENST000004
ENST00000262467,ENST00000269280,ENST00000345221,ENST00000354411,ENST00000544378,ENST000005
ENST00000285968
ENST00000268482
ENST00000404459
ENST00000299506,ENST00000305253,ENST00000534099
ENST00000256999,ENST00000340334,ENST00000356696,ENST00000533034
ENST00000320033
ENST00000373840,ENST00000451303
ENST00000025008,ENST00000435644,ENST00000539297
ENST00000420699,ENST00000461153
ENST00000435989
ENST00000304514,ENST00000409886
ENST00000368799
ENST00000435359,ENST00000538975,ENST00000570628,ENST00000574426
ENST00000302874,ENST00000507760
ENST00000285968
ENST00000263816,ENST00000443831
ENST00000263816,ENST00000443831
ENST00000358097,ENST00000433992
ENST00000340781,ENST00000367134,ENST00000367135
ENST00000371764
ENST00000306151,ENST00000379439
ENST00000529911
ENST00000262467,ENST00000269280,ENST00000345221,ENST00000354411,ENST00000544378,ENST000005
ENST00000256999,ENST00000340334,ENST00000356696,ENST00000533034
ENST00000262836,ENST00000360648,ENST00000372007,ENST00000372010,ENST00000417227,ENST000004
ENST00000320033
ENST00000288350,ENST00000358268,ENST00000380671
ENST00000361367

ENST00000367848
ENST00000290239,ENST00000300278,ENST00000356577,ENST00000381679,ENST00000455528
ENST00000435359,ENST00000538975,ENST00000570628,ENST00000574426
ENST00000547628,ENST00000602107
ENST00000263434,ENST00000376350
ENST00000302874,ENST00000507760
ENST00000290209,ENST00000354181,ENST00000397702,ENST00000397707,ENST00000451844,ENST00000
ENST00000320078,ENST00000401740,ENST00000593983,ENST00000598133
ENST00000435989
ENST00000373840,ENST00000451303
ENST00000320743,ENST00000379141,ENST00000433272,ENST00000552267
ENST00000025008,ENST00000435644,ENST00000539297
ENST00000356508,ENST00000409281,ENST00000409419,ENST00000409587,ENST00000409735
ENST00000374024
ENST00000304514,ENST00000409886
ENST00000305253
ENST00000404459
ENST00000368799
ENST00000420699,ENST00000461153
ENST00000268482
ENST00000348028,ENST00000349350,ENST00000421100,ENST00000427578,ENST00000443370,ENST00000
ENST00000328354,ENST00000348295,ENST00000382578,ENST00000382580,ENST00000402731,ENST00000
ENST00000559949
ENST00000361789
ENST00000219476,ENST00000350773,ENST00000353929,ENST00000382538,ENST00000401874,ENST00000
ENST00000301067
ENST00000278882,ENST00000358464,ENST00000439954
ENST00000310336,ENST00000379922,ENST00000379939,ENST00000400445,ENST00000537702,ENST00000
ENST00000361789
ENST00000437584,ENST00000535591
ENST00000231498,ENST00000381843,ENST00000513532
ENST00000358970,ENST00000511306
ENST00000382292,ENST00000382298,ENST00000402364
ENST00000357033,ENST00000378677
ENST00000361789
ENST00000361789
ENST00000348028,ENST00000349350,ENST00000421100,ENST00000427578,ENST00000443370,ENST00000
ENST00000301067
ENST00000382292,ENST00000382298,ENST00000402364
ENST00000278882,ENST00000358464,ENST00000439954
ENST00000437584,ENST00000535591
ENST00000328354,ENST00000348295,ENST00000382578,ENST00000382580,ENST00000402731,ENST00000
ENST00000357033,ENST00000378677
ENST00000231498,ENST00000381843,ENST00000513532
ENST00000310336,ENST00000379922,ENST00000379939,ENST00000400445,ENST00000537702,ENST00000
ENST00000358970,ENST00000511306
ENST00000219476,ENST00000350773,ENST00000353929,ENST00000382538,ENST00000401874,ENST00000

ENST00000559949
ENST00000361624
ENST00000361567
ENST00000437584,ENST00000535591
ENST00000437584,ENST00000535591
ENST00000297826,ENST00000374469,ENST00000401783
ENST00000334961,ENST00000338641,ENST00000353887,ENST00000361166,ENST00000361452,ENST00000[illegible]
ENST00000441802
ENST00000361739
ENST00000328423,ENST00000431282,ENST00000564831
ENST00000356641,ENST00000397843,ENST00000532993
ENST00000283195
ENST00000279575,ENST00000535904
ENST00000375915
ENST00000361739
ENST00000361624
ENST00000437584,ENST00000535591,ENST00000437584,ENST00000535591
ENST00000361567
ENST00000441802
ENST00000356641,ENST00000397843,ENST00000532993
ENST00000334961,ENST00000338641,ENST00000353887,ENST00000361166,ENST00000361452,ENST00000[illegible]
ENST00000328423,ENST00000431282,ENST00000564831
ENST00000297826,ENST00000374469,ENST00000401783
ENST00000283195
ENST00000375915
ENST00000279575,ENST00000535904
ENST00000298232,ENST00000328758,ENST00000342420,ENST00000361285
ENST00000358970,ENST00000511306
ENST00000304514,ENST00000409886
ENST00000358970,ENST00000511306
ENST00000358397,ENST00000423620,ENST00000433389,ENST00000454170
ENST00000304514,ENST00000409886
ENST00000313581,ENST00000431575,ENST00000520309
ENST00000361899
ENST00000559949
ENST00000334344
ENST00000434794
ENST00000256442,ENST00000505500,ENST00000506572,ENST00000508407
ENST00000283195
ENST00000288368
ENST00000251268,ENST00000334370
ENST00000318560,ENST00000372348
ENST00000367482,ENST00000367483,ENST00000367485,ENST00000367486,ENST00000445192
ENST00000339872,ENST00000341423,ENST00000399494,ENST00000405805
ENST00000256442,ENST00000505500,ENST00000506572,ENST00000508407
ENST00000318560,ENST00000372348
ENST00000298232,ENST00000328758,ENST00000342420,ENST00000361285

ENST00000251268,ENST00000334370
ENST00000283195
ENST00000313581,ENST00000431575,ENST00000520309
ENST00000334344
ENST00000361899
ENST00000358970,ENST00000511306
ENST00000304514,ENST00000409886,ENST00000304514,ENST00000409886
ENST00000389484,ENST00000434794
ENST00000288368
ENST00000358397,ENST00000423620,ENST00000433389,ENST00000454170
ENST00000358970,ENST00000511306
ENST00000559949
ENST00000367482,ENST00000367483,ENST00000367485,ENST00000367486,ENST00000445192
ENST00000339872,ENST00000341423,ENST00000399494,ENST00000405805
ENST00000304887,ENST00000413702,ENST00000456088
ENST00000255448,ENST00000360631,ENST00000379892
ENST00000262207,ENST00000517786,ENST00000523524
ENST00000370742
ENST00000284476
ENST00000462323,ENST00000463781,ENST00000466475,ENST00000470451,ENST00000475231,ENST00000[illegible]
ENST00000357033,ENST00000378677
ENST00000342175,ENST00000342992,ENST00000359218,ENST00000360870,ENST00000460472,ENST00000[illegible]
ENST00000263734
ENST00000371852
ENST00000589042
ENST00000375888,ENST00000375897,ENST00000375901,ENST00000375905,ENST00000396271
ENST00000297494,ENST00000461406
ENST00000345988,ENST00000590782,ENST00000592772
ENST00000370225,ENST00000535881,ENST00000536513
ENST00000357816,ENST00000381853,ENST00000515384
ENST00000358970
ENST00000342056,ENST00000355194,ENST00000355635,ENST00000370398,ENST00000402622,ENST00000[illegible]
ENST00000278937,ENST00000438295
ENST00000334705,ENST00000521166
ENST00000296088,ENST00000429705,ENST00000437827,ENST00000454177
ENST00000262539,ENST00000374541,ENST00000394827,ENST00000412145,ENST00000446349
ENST00000193391
ENST00000359568
ENST00000231454
ENST00000303383
ENST00000357033,ENST00000378677
ENST00000304863
ENST00000368198,ENST00000368199,ENST00000392306
ENST00000394420,ENST00000537866
ENST00000370225,ENST00000535881,ENST00000536513
ENST00000255448,ENST00000360631,ENST00000379892
ENST00000303383

ENST00000394420,ENST00000537866
ENST00000262539,ENST00000374541,ENST00000394827,ENST00000412145,ENST00000446349
ENST00000193391
ENST00000371852
ENST00000345988,ENST00000590782,ENST00000592772
ENST00000357033,ENST00000378677
ENST00000334705,ENST00000521166
ENST00000284476
ENST00000231454
ENST00000342175,ENST00000342992,ENST00000359218,ENST00000360870,ENST00000460472,ENST00000
ENST00000370742
ENST00000342056,ENST00000355194,ENST00000355635,ENST00000370398,ENST00000402622,ENST00000
ENST00000357816,ENST00000381853,ENST00000515384
ENST00000375888,ENST00000375897,ENST00000375901,ENST00000375905,ENST00000396271
ENST00000357033,ENST00000378677
ENST00000262207,ENST00000517786,ENST00000523524
ENST00000304863
ENST00000296088,ENST00000429705,ENST00000437827,ENST00000454177
ENST00000589042
ENST00000368198,ENST00000368199,ENST00000392306
ENST00000297494,ENST00000461406
ENST00000263734
ENST00000359568
ENST00000462323,ENST00000463781,ENST00000466475,ENST00000470451,ENST00000475231,ENST00000
ENST00000278937,ENST00000438295
ENST00000358970
ENST00000304887,ENST00000413702,ENST00000456088
ENST00000366789
ENST00000340160,ENST00000520358,ENST00000520837,ENST00000520999,ENST00000533080,ENST00000
ENST00000306151,ENST00000379439
ENST00000262207,ENST00000517786,ENST00000523524
ENST00000370742
ENST00000357033,ENST00000378677
ENST00000342175,ENST00000342992,ENST00000359218,ENST00000360870,ENST00000460472,ENST00000
ENST00000306177,ENST00000426246,ENST00000465073,ENST00000521713
ENST00000263734
ENST00000328354,ENST00000348295,ENST00000382578,ENST00000382580,ENST00000402731,ENST00000
ENST00000348161,ENST00000445275,ENST00000536554,ENST00000540336,ENST00000570371,ENST00000
ENST00000258385,ENST00000457943,ENST00000543200
ENST00000589042
ENST00000375806,ENST00000375809,ENST00000375810,ENST00000480039
ENST00000262765
ENST00000005386,ENST00000380650
ENST00000370225,ENST00000535881,ENST00000536513
ENST00000357816,ENST00000381853,ENST00000515384
ENST00000297990,ENST00000353159,ENST00000379471,ENST00000455041
ENST00000295025,ENST00000394479

ENST00000358970
ENST00000265577,ENST00000317367,ENST00000339809,ENST00000396675,ENST00000401396,ENST00000[illegible]
ENST00000334705,ENST00000521166
ENST00000326441,ENST00000423103,ENST00000593695,ENST00000598410,ENST00000599534,ENST00000[illegible]
ENST00000296088,ENST00000429705,ENST00000437827,ENST00000454177
ENST00000262539,ENST00000374541,ENST00000394827,ENST00000412145,ENST00000446349
ENST00000254322,ENST00000396969
ENST00000381271
ENST00000278360,ENST00000378878,ENST00000378880,ENST00000527605,ENST00000532848
ENST00000294954,ENST00000344775,ENST00000405626
ENST00000335312,ENST00000537021,ENST00000539785,ENST00000589578
ENST00000311852,ENST00000548761
ENST00000357033,ENST00000378677
ENST00000397126
ENST00000394420,ENST00000537866
ENST00000435989
ENST00000358519,ENST00000402813,ENST00000420489,ENST00000514170,ENST00000544810
ENST00000324883,ENST00000324907,ENST00000405379,ENST00000472167
ENST00000370225,ENST00000535881,ENST00000536513
ENST00000348161,ENST00000445275,ENST00000536554,ENST00000540336,ENST00000570371,ENST00000[illegible]
ENST00000366789
ENST00000375809,ENST00000375810
ENST00000394420,ENST00000537866
ENST00000358519,ENST00000402813,ENST00000420489,ENST00000514170,ENST00000544810
ENST00000262539,ENST00000374541,ENST00000394827,ENST00000412145,ENST00000446349
ENST00000306177,ENST00000426246,ENST00000465073,ENST00000521713
ENST00000328354,ENST00000348295,ENST00000382578,ENST00000382580,ENST00000402731,ENST00000[illegible]
ENST00000357033,ENST00000378677
ENST00000340160,ENST00000520358,ENST00000520837,ENST00000520999,ENST00000533202,ENST00000[illegible]
ENST00000334705,ENST00000521166
ENST00000324883,ENST00000324907,ENST00000405379,ENST00000472167
ENST00000342175,ENST00000342992,ENST00000359218,ENST00000360870,ENST00000460472,ENST00000[illegible]
ENST00000370742
ENST00000258385,ENST00000457943,ENST00000543200
ENST00000265577,ENST00000317367,ENST00000339809,ENST00000396675,ENST00000401396,ENST00000[illegible]
ENST00000381271
ENST00000357816,ENST00000381853,ENST00000515384
ENST00000357033,ENST00000378677
ENST00000262207,ENST00000517786,ENST00000523524
ENST00000297990,ENST00000353159,ENST00000379471,ENST00000455041
ENST00000278360,ENST00000378878,ENST00000378880,ENST00000527605,ENST00000532848
ENST00000294954,ENST00000344775,ENST00000405626
ENST00000296088,ENST00000429705,ENST00000437827,ENST00000454177
ENST00000589042
ENST00000254322,ENST00000396969
ENST00000311852,ENST00000548761
ENST00000262765

ENST00000435989
ENST00000295025,ENST00000394479
ENST00000397126
ENST00000005386,ENST00000380650
ENST00000263734
ENST00000335312,ENST00000537021,ENST00000539785,ENST00000589578
ENST00000326441,ENST00000423103,ENST00000593695,ENST00000598410,ENST00000599534,ENST00000[illegible]
ENST00000358970
ENST00000306151,ENST00000379439
ENST00000359775,ENST00000414563
ENST00000251496
ENST00000318220,ENST00000318249,ENST00000342104,ENST00000434838,ENST00000454000
ENST00000360861,ENST00000416216,ENST00000427436,ENST00000454876,ENST00000458124
ENST00000306151,ENST00000379439
ENST00000395385,ENST00000402377
ENST00000343769,ENST00000397126
ENST00000252799,ENST00000395094,ENST00000535210,ENST00000568028,ENST00000569360
ENST00000238341,ENST00000373855,ENST00000373865
ENST00000415833,ENST00000424340,ENST00000517539,ENST00000528249
ENST00000427401,ENST00000594419
ENST00000264245
ENST00000262873
ENST00000355504,ENST00000392288
ENST00000273153,ENST00000514182
ENST00000311234,ENST00000398119,ENST00000420668,ENST00000442263,ENST00000463928,ENST00000[illegible]
ENST00000358855,ENST00000432670,ENST00000442668,ENST00000535387,ENST00000542053,ENST00000[illegible]
ENST00000348028,ENST00000349350,ENST00000421100,ENST00000427578,ENST00000443370,ENST00000[illegible]
ENST00000397126
ENST00000316073
ENST00000369219
ENST00000269228,ENST00000412552
ENST00000286349
ENST00000312108,ENST00000360719,ENST00000395650,ENST00000405237,ENST00000434492,ENST00000[illegible]
ENST00000291700,ENST00000367071,ENST00000397648
ENST00000281172,ENST00000540613,ENST00000542903,ENST00000543523,ENST00000543612
ENST00000294172,ENST00000439713,ENST00000525576,ENST00000530875,ENST00000531709,ENST00000[illegible]
ENST00000299367,ENST00000442278,ENST00000452323,ENST00000456570,ENST00000469372,ENST00000[illegible]
ENST00000462323,ENST00000463781,ENST00000466475,ENST00000470451,ENST00000475231,ENST00000[illegible]
ENST00000316073
ENST00000228682,ENST00000528467,ENST00000532291,ENST00000543426,ENST00000546141
ENST00000316073
ENST00000355619,ENST00000417323
ENST00000278520,ENST00000423339,ENST00000542662
ENST00000361883
ENST00000240499,ENST00000395385,ENST00000402377
ENST00000306151,ENST00000379439
ENST00000324725,ENST00000398027,ENST00000490721

ENST00000331442
ENST00000333891,ENST00000423517
ENST00000333892
ENST00000355072
ENST00000397126
ENST00000306151,ENST00000379439
ENST00000260595,ENST00000312734,ENST00000402394,ENST00000404694,ENST00000405600
ENST00000260595,ENST00000312734,ENST00000402394,ENST00000404694,ENST00000405600
ENST00000240499
ENST00000397126
ENST00000355504,ENST00000392288
ENST00000360861,ENST00000416216,ENST00000427436,ENST00000454876,ENST00000458124
ENST00000427401,ENST00000594419
ENST00000331442
ENST00000251496
ENST00000359775,ENST00000414563
ENST00000262873
ENST00000343769
ENST00000311234,ENST00000398119,ENST00000420668,ENST00000442263,ENST00000463928,ENST00000
ENST00000264245
ENST00000281172,ENST00000540613,ENST00000542903,ENST00000543523,ENST00000543612
ENST00000269228,ENST00000412552
ENST00000291700,ENST00000367071,ENST00000397648
ENST00000299367,ENST00000442278,ENST00000452323,ENST00000469372,ENST00000497706,ENST00000
ENST00000273153,ENST00000514182
ENST00000238341,ENST00000373855,ENST00000373865
ENST00000397126
ENST00000395385,ENST00000402377
ENST00000306151,ENST00000379439
ENST00000252799,ENST00000395094,ENST00000535210,ENST00000568028,ENST00000569360
ENST00000312108,ENST00000360719,ENST00000395650,ENST00000405237,ENST00000434492,ENST00000
ENST00000306151,ENST00000379439
ENST00000286349
ENST00000278520,ENST00000423339,ENST00000542662
ENST00000358855,ENST00000432670,ENST00000442668,ENST00000535387,ENST00000542053,ENST00000
ENST00000355619,ENST00000417323
ENST00000462323,ENST00000463781,ENST00000466475,ENST00000470451,ENST00000475231,ENST00000
ENST00000369219
ENST00000306151,ENST00000379439
ENST00000355072
ENST00000318220,ENST00000318249,ENST00000342104,ENST00000434838,ENST00000454000
ENST00000228682,ENST00000528467,ENST00000532291,ENST00000543426,ENST00000546141
ENST00000260595,ENST00000312734,ENST00000402394,ENST00000404694,ENST00000405600
ENST00000294172,ENST00000439713,ENST00000525576,ENST00000530875,ENST00000531709,ENST00000
ENST00000324725,ENST00000398027,ENST00000490721
ENST00000415833,ENST00000424340,ENST00000517539,ENST00000528249
ENST00000333892

ENST00000316073
ENST00000316073
ENST00000361883
ENST00000316073,ENST00000316073
ENST00000348028,ENST00000349350,ENST00000421100,ENST00000427578,ENST00000443370,ENST00000[illegible]
ENST00000333891,ENST00000423517
ENST00000264771,ENST00000507319,ENST00000514453,ENST00000514546
ENST00000241124,ENST00000356192,ENST00000400065,ENST00000400066
ENST00000251496
ENST00000455150
ENST00000329564
ENST00000219406,ENST00000404312,ENST00000435833
ENST00000255381
ENST00000242323,ENST00000377724
ENST00000278882,ENST00000358464,ENST00000439954
ENST00000328405
ENST00000415833,ENST00000424340,ENST00000517539,ENST00000528249
ENST00000376752,ENST00000376767,ENST00000424854,ENST00000545128
ENST00000264245
ENST00000349570,ENST00000358353,ENST00000397542
ENST00000341509,ENST00000537670
ENST00000160373
ENST00000358855,ENST00000432670,ENST00000442668,ENST00000535387,ENST00000542053,ENST00000[illegible]
ENST00000397126
ENST00000295903,ENST00000498707
ENST00000256443
ENST00000329697,ENST00000565232,ENST00000565322,ENST00000567001
ENST00000306829,ENST00000561311
ENST00000011898,ENST00000407263,ENST00000537971
ENST00000269228,ENST00000412552
ENST00000286349
ENST00000262519
ENST00000263331,ENST00000409894,ENST00000417433,ENST00000537335,ENST00000541869
ENST00000252519,ENST00000427411
ENST00000269217,ENST00000313654,ENST00000399516,ENST00000587184
ENST00000236957,ENST00000392221,ENST00000392222,ENST00000415904,ENST00000429769,ENST00000[illegible]
ENST00000322146,ENST00000597252
ENST00000221355,ENST00000434248
ENST00000362057
ENST00000242310
ENST00000316073
ENST00000462323,ENST00000463781,ENST00000466475,ENST00000470451,ENST00000475231,ENST00000[illegible]
ENST00000356025,ENST00000358771,ENST00000400068
ENST00000355619,ENST00000417323
ENST00000381396,ENST00000396208,ENST00000396212
ENST00000378024
ENST00000261772

ENST00000355072
ENST00000397126
ENST00000262519
ENST00000251496
ENST00000264771,ENST00000507319,ENST00000508204,ENST00000509508,ENST00000510493,ENST00000
ENST00000349570,ENST00000358353,ENST00000397542
ENST00000376752,ENST00000376767,ENST00000424854,ENST00000545128
ENST00000328405
ENST00000269217,ENST00000313654,ENST00000399516,ENST00000587184
ENST00000160373
ENST00000329564
ENST00000264245
ENST00000269228,ENST00000412552
ENST00000455150
ENST00000278882,ENST00000358464,ENST00000439954
ENST00000263331,ENST00000409894,ENST00000417433,ENST00000537335,ENST00000541869
ENST00000241124,ENST00000356192,ENST00000400065,ENST00000400066
ENST00000261772
ENST00000341509,ENST00000537670
ENST00000219406,ENST00000404312,ENST00000435833
ENST00000356025,ENST00000358771,ENST00000400068
ENST00000236957,ENST00000392221,ENST00000392222,ENST00000415904,ENST00000429769,ENST00000
ENST00000011898,ENST00000407263,ENST00000537971
ENST00000306829,ENST00000561311
ENST00000242310
ENST00000221355,ENST00000434248
ENST00000286349
ENST00000322146,ENST00000597252
ENST00000358855,ENST00000432670,ENST00000442668,ENST00000535387,ENST00000542053,ENST00000
ENST00000255381
ENST00000355619,ENST00000417323
ENST00000242323,ENST00000377724
ENST00000256443,ENST00000514676
ENST00000378024
ENST00000295903,ENST00000498707
ENST00000329697,ENST00000565232,ENST00000565322,ENST00000567001
ENST00000355072
ENST00000381396,ENST00000396208,ENST00000396212
ENST00000252519,ENST00000427411
ENST00000462323,ENST00000463781,ENST00000466475,ENST00000470451,ENST00000475231,ENST00000
ENST00000362057
ENST00000415833,ENST00000424340,ENST00000517539,ENST00000528249
ENST00000316073
ENST00000357409,ENST00000360280,ENST00000376634,ENST00000376636
ENST00000382205,ENST00000526438
ENST00000437584,ENST00000535591
ENST00000355957,ENST00000358029,ENST00000361770,ENST00000361830,ENST00000371132,ENST00000

ENST00000388820
ENST00000366553
ENST00000369870
ENST00000538744
ENST00000425945
ENST00000337331
ENST00000437584,ENST00000535591
ENST00000355973,ENST00000376977,ENST00000377012,ENST00000377016,ENST00000377034,ENST00000[illegible]
ENST00000314612
ENST00000321424,ENST00000340550,ENST00000430817
ENST00000298047,ENST00000409404,ENST00000525166
ENST00000261203,ENST00000549417,ENST00000552864
ENST00000240189
ENST00000400299,ENST00000402395
ENST00000338820,ENST00000394903,ENST00000444643
ENST00000343518
ENST00000357214
ENST00000609686
ENST00000305031
ENST00000342868
ENST00000359376
ENST00000188403,ENST00000321046,ENST00000392986,ENST00000548884,ENST00000549996,ENST00000[illegible]
ENST00000342960
ENST00000259206,ENST00000354115,ENST00000409930
ENST00000361381
ENST00000275884,ENST00000496613,ENST00000537639
ENST00000229447,ENST00000344419,ENST00000367335,ENST00000392255,ENST00000392256,ENST00000[illegible]
ENST00000489525
ENST00000376296
ENST00000310441,ENST00000354233,ENST00000369984
ENST00000382622
ENST00000343414
ENST00000373344,ENST00000395603
ENST00000400299,ENST00000402395
ENST00000489525
ENST00000376296
ENST00000369870
ENST00000358029
ENST00000538744
ENST00000310441,ENST00000354233,ENST00000369984
ENST00000355973,ENST00000376977,ENST00000377012,ENST00000377016,ENST00000377034,ENST00000[illegible]
ENST00000355973,ENST00000376977,ENST00000377012,ENST00000377016,ENST00000377034,ENST00000[illegible]
ENST00000357214
ENST00000382205,ENST00000526438
ENST00000305031
ENST00000240189
ENST00000314612

ENST00000437584,ENST00000535591
ENST00000343414
ENST00000388820
ENST00000437584,ENST00000535591
ENST00000298047,ENST00000409404,ENST00000525166
ENST00000275884,ENST00000496613,ENST00000537639
ENST00000259206,ENST00000354115,ENST00000409930
ENST00000425945
ENST00000452611
ENST00000361381
ENST00000366553
ENST00000229447,ENST00000344419,ENST00000367335,ENST00000392255,ENST00000392256,ENST00000[illegible]
ENST00000373344,ENST00000395603
ENST00000261203,ENST00000549417,ENST00000552864
ENST00000357409,ENST00000360280,ENST00000376634,ENST00000376636
ENST00000609686
ENST00000188403,ENST00000321046,ENST00000392986,ENST00000548884,ENST00000549996,ENST00000[illegible]
ENST00000338820,ENST00000394903,ENST00000444643
ENST00000342868
ENST00000359376
ENST00000321424,ENST00000340550,ENST00000430817
ENST00000342960
ENST00000337331
ENST00000343518
ENST00000357409,ENST00000360280,ENST00000376634,ENST00000376636
ENST00000361789
ENST00000373509
ENST00000260323,ENST00000537900,ENST00000545554
ENST00000361739
ENST00000366553
ENST00000265351
ENST00000336233,ENST00000371837,ENST00000456827
ENST00000369870
ENST00000368144
ENST00000337331
ENST00000267377
ENST00000262215,ENST00000518230,ENST00000520381
ENST00000298047,ENST00000409404,ENST00000525166
ENST00000243776,ENST00000535926
ENST00000282282
ENST00000315563,ENST00000370992,ENST00000414986,ENST00000536831
ENST00000338820,ENST00000394903,ENST00000444643
ENST00000336431
ENST00000303254
ENST00000295902,ENST00000564377
ENST00000609686
ENST00000456556

ENST00000305031
ENST00000342868
ENST00000359376
ENST00000259206,ENST00000354115,ENST00000409930
ENST00000262968,ENST00000382008,ENST00000539908,ENST00000541714,ENST00000587686,ENST00000[illegible]
ENST00000216487
ENST00000361381
ENST00000275884,ENST00000496613,ENST00000537639
ENST00000375001
ENST00000346322,ENST00000359169,ENST00000360304,ENST00000360772,ENST00000475616
ENST00000336431
ENST00000369870
ENST00000361789
ENST00000315563,ENST00000370992,ENST00000414986,ENST00000536831
ENST00000361739
ENST00000305031
ENST00000262968,ENST00000382008,ENST00000539908,ENST00000541714,ENST00000587686,ENST00000[illegible]
ENST00000265351
ENST00000260323,ENST00000537900,ENST00000545554
ENST00000267377
ENST00000336233,ENST00000371837,ENST00000456827
ENST00000298047,ENST00000409404,ENST00000525166
ENST00000275884,ENST00000496613,ENST00000537639
ENST00000282282
ENST00000259206,ENST00000354115,ENST00000409930
ENST00000361381
ENST00000295902,ENST00000564377
ENST00000375001
ENST00000366553
ENST00000373509
ENST00000373509
ENST00000303254
ENST00000357409,ENST00000360280,ENST00000376634,ENST00000376636
ENST00000346322,ENST00000359169,ENST00000360304,ENST00000360772,ENST00000475616
ENST00000609686
ENST00000338820,ENST00000394903,ENST00000444643
ENST00000342868
ENST00000368144
ENST00000359376
ENST00000216487
ENST00000262215,ENST00000518230,ENST00000520381
ENST00000456556
ENST00000337331
ENST00000243776,ENST00000535926
ENST00000304166,ENST00000396211,ENST00000409363,ENST00000409489
ENST00000350608,ENST00000398344,ENST00000404092
ENST00000381334,ENST00000506198,ENST00000508404

ENST00000360151
ENST00000371222
ENST00000293725
ENST00000369219
ENST00000340273
ENST00000300619,ENST00000397082
ENST00000350608,ENST00000398344,ENST00000404092
ENST00000333796
ENST00000318220,ENST00000318249,ENST00000342104,ENST00000434838,ENST00000454000
ENST00000368836
ENST00000379708,ENST00000416505,ENST00000520655,ENST00000520810,ENST00000520835,ENST00000[illegible]
ENST00000262269,ENST00000376970,ENST00000425460,ENST00000440075,ENST00000596571,ENST00000[illegible]
ENST00000296318,ENST00000320057,ENST00000427856,ENST00000463523
ENST00000547628,ENST00000602107
ENST00000233242,ENST00000399256
ENST00000399147
ENST00000375494
ENST00000601440
ENST00000245543
ENST00000562295
ENST00000430580
ENST00000288447,ENST00000357033,ENST00000378677
ENST00000400454,ENST00000404019
ENST00000356929
ENST00000536684,ENST00000540040,ENST00000604882
ENST00000399147
ENST00000292309,ENST00000450053
ENST00000391675,ENST00000465342
ENST00000267219,ENST00000351546,ENST00000358679,ENST00000418532,ENST00000441784,ENST00000[illegible]
ENST00000342960
ENST00000380250
ENST00000311048
ENST00000343776,ENST00000397097,ENST00000418100
ENST00000211998,ENST00000372755,ENST00000436396
ENST00000338977,ENST00000511833
ENST00000357491,ENST00000601693
ENST00000444627
ENST00000317658
ENST00000594710
ENST00000240499,ENST00000300619,ENST00000343776,ENST00000357491,ENST00000397082,ENST00000[illegible]
ENST00000306100,ENST00000379164,ENST00000427802,ENST00000536695
ENST00000358491
ENST00000594710
ENST00000187397,ENST00000337271,ENST00000417925,ENST00000444190,ENST00000457165,ENST00000[illegible]
ENST00000342944,ENST00000357002
ENST00000248244
ENST00000300619,ENST00000311048,ENST00000397082

ENST00000528956
ENST00000261263
ENST00000397126,ENST00000594947
ENST00000311048
ENST00000397126
ENST00000418100
ENST00000431200
ENST00000594947
ENST00000357491,ENST00000358491,ENST00000418063,ENST00000601693
ENST00000397126
ENST00000418063
ENST00000444627
ENST00000245543
ENST00000350608,ENST00000398344,ENST00000404092,ENST00000350608,ENST00000398344,ENST00000
ENST00000380250
ENST00000296318,ENST00000320057,ENST00000427856,ENST00000463523
ENST00000399147,ENST00000399147
ENST00000357491,ENST00000601693
ENST00000381334,ENST00000506198,ENST00000508404
ENST00000317658
ENST00000338977,ENST00000511833
ENST00000233242,ENST00000399256
ENST00000601440
ENST00000357491,ENST00000601693
ENST00000528956
ENST00000342944,ENST00000357002
ENST00000368836
ENST00000292309,ENST00000450053
ENST00000536684,ENST00000540040,ENST00000604882
ENST00000293725
ENST00000306100,ENST00000379164,ENST00000427802,ENST00000536695
ENST00000356929
ENST00000340273
ENST00000418063
ENST00000187397,ENST00000337271,ENST00000417925,ENST00000444190,ENST00000457165,ENST00000
ENST00000300619,ENST00000397082
ENST00000379708,ENST00000416505,ENST00000520655,ENST00000520810,ENST00000520835,ENST00000
ENST00000369219
ENST00000562295
ENST00000397126
ENST00000358491
ENST00000248244
ENST00000360151
ENST00000375494
ENST00000399147
ENST00000333796
ENST00000311048,ENST00000311048

ENST00000371222
ENST00000262269,ENST00000376970,ENST00000425460,ENST00000440075,ENST00000596571,ENST00000[illegible]
ENST00000343776,ENST00000397097
ENST00000288447,ENST00000357033,ENST00000378677
ENST00000604882
ENST00000397126
ENST00000547628,ENST00000602107
ENST00000304166,ENST00000396211,ENST00000409363,ENST00000409489
ENST00000211998,ENST00000372755,ENST00000436396
ENST00000391675,ENST00000465342
ENST00000594947
ENST00000300619,ENST00000397082
ENST00000594710,ENST00000594710
ENST00000400454,ENST00000404019
ENST00000594710
ENST00000430580
ENST00000418100
ENST00000240499
ENST00000540040
ENST00000267219,ENST00000351546,ENST00000358679,ENST00000418532,ENST00000441784,ENST00000[illegible]
ENST00000342960
ENST00000431200
ENST00000318220,ENST00000318249,ENST00000342104,ENST00000434838,ENST00000454000
ENST00000261263
ENST00000397126
ENST00000397126
ENST00000441802
ENST00000304166,ENST00000396211,ENST00000409363,ENST00000409489
ENST00000381334,ENST00000506198,ENST00000508404
ENST00000371222
ENST00000318220,ENST00000318249,ENST00000342104,ENST00000434838,ENST00000454000
ENST00000379708,ENST00000416505,ENST00000520655,ENST00000520810,ENST00000520835,ENST00000[illegible]
ENST00000360937,ENST00000416793,ENST00000460213,ENST00000467291,ENST00000483043,ENST00000[illegible]
ENST00000296318,ENST00000320057,ENST00000427856,ENST00000463523
ENST00000233242,ENST00000399256
ENST00000305432,ENST00000305447,ENST00000393294,ENST00000393297,ENST00000418177,ENST00000[illegible]
ENST00000439326
ENST00000245543
ENST00000318220,ENST00000318249,ENST00000342104,ENST00000434838,ENST00000454000
ENST00000313382,ENST00000369347,ENST00000369348,ENST00000369349,ENST00000369351,ENST00000[illegible]
ENST00000430580
ENST00000400454,ENST00000404019
ENST00000391675,ENST00000465342
ENST00000336443,ENST00000354280,ENST00000504988,ENST00000555685
ENST00000267219,ENST00000351546,ENST00000358679,ENST00000418532,ENST00000441784,ENST00000[illegible]
ENST00000262189,ENST00000355193
ENST00000380250

ENST00000418100
ENST00000211998,ENST00000372755,ENST00000436396
ENST00000397126
ENST00000343769,ENST00000357491,ENST00000418063,ENST00000418100,ENST00000427401,ENST00000
ENST00000359626
ENST00000304004
ENST00000427401,ENST00000594419
ENST00000187397,ENST00000337271,ENST00000417925,ENST00000444190,ENST00000457165,ENST00000
ENST00000560223
ENST00000248244
ENST00000528956
ENST00000418063
ENST00000357491,ENST00000601693
ENST00000311635,ENST00000565593
ENST00000291182
ENST00000245543
ENST00000397126
ENST00000305432,ENST00000305447,ENST00000393294,ENST00000393297,ENST00000418177,ENST00000
ENST00000357491,ENST00000601693
ENST00000380250
ENST00000296318,ENST00000320057,ENST00000427856,ENST00000463523
ENST00000439326
ENST00000311635,ENST00000565593
ENST00000381334,ENST00000506198,ENST00000508404
ENST00000233242,ENST00000399256
ENST00000313382,ENST00000369347,ENST00000369348,ENST00000369349,ENST00000369351,ENST00000
ENST00000397126
ENST00000528956
ENST00000262189,ENST00000355193
ENST00000560223
ENST00000187397,ENST00000337271,ENST00000417925,ENST00000444190,ENST00000457165,ENST00000
ENST00000304004
ENST00000379708,ENST00000416505,ENST00000520655,ENST00000520810,ENST00000520835,ENST00000
ENST00000248244
ENST00000360937,ENST00000416793,ENST00000460213,ENST00000467291,ENST00000483043,ENST00000
ENST00000291182
ENST00000441802
ENST00000371222
ENST00000418100
ENST00000304166,ENST00000396211,ENST00000409363,ENST00000409489
ENST00000359626
ENST00000211998,ENST00000372755,ENST00000436396
ENST00000391675,ENST00000465342
ENST00000418063
ENST00000400454,ENST00000404019
ENST00000343769
ENST00000430580

ENST00000427401,ENST00000594419
ENST00000318220,ENST00000318249,ENST00000342104,ENST00000434838,ENST00000454000
ENST00000267219,ENST00000351546,ENST00000358679,ENST00000418532,ENST00000441784,ENST000004
ENST00000336443,ENST00000354280,ENST00000555685,ENST00000504988
ENST00000318220,ENST00000318249,ENST00000342104,ENST00000434838,ENST00000454000
ENST00000301831,ENST00000420927
ENST00000260682
ENST00000335094
ENST00000304166,ENST00000396211,ENST00000409363,ENST00000409489
ENST00000320481,ENST00000376410,ENST00000396432,ENST00000446003
ENST00000247930
ENST00000296424,ENST00000508136
ENST00000344579,ENST00000378344
ENST00000321797,ENST00000341547,ENST00000357063,ENST00000377499,ENST00000377534,ENST000004
ENST00000299272,ENST00000551346
ENST00000301831,ENST00000420927
ENST00000250559,ENST00000341355,ENST00000378985,ENST00000393436,ENST00000450214,ENST000005
ENST00000342175,ENST00000342992,ENST00000359218,ENST00000460472,ENST00000589042,ENST000005
ENST00000296318,ENST00000320057,ENST00000427856,ENST00000463523
ENST00000233242,ENST00000399256
ENST00000265403
ENST00000329143,ENST00000430323,ENST00000533655
ENST00000245441,ENST00000324330,ENST00000382041,ENST00000382043,ENST00000389868,ENST000004
ENST00000360509,ENST00000396328,ENST00000557913,ENST00000559418
ENST00000417025
ENST00000343098,ENST00000424728
ENST00000368507,ENST00000368508
ENST00000319921,ENST00000456466,ENST00000508628,ENST00000582970
ENST00000343776,ENST00000397097
ENST00000341048,ENST00000504958,ENST00000513241
ENST00000341415
ENST00000391675,ENST00000465342
ENST00000296701,ENST00000340253,ENST00000394370,ENST00000513826
ENST00000299886
ENST00000333837,ENST00000395487,ENST00000429094,ENST00000437801,ENST00000452445,ENST000005
ENST00000299886
ENST00000328843,ENST00000409508,ENST00000605912
ENST00000526538
ENST00000299886
ENST00000187397,ENST00000337271,ENST00000417925,ENST00000444190,ENST00000457165,ENST000004
ENST00000330838,ENST00000358602,ENST00000509867
ENST00000528956
ENST00000367870,ENST00000367872,ENST00000447624
ENST00000361927,ENST00000546159
ENST00000375915
ENST00000301831,ENST00000420927,ENST00000301831,ENST00000420927
ENST00000361927,ENST00000546159

ENST00000260682
ENST00000319921,ENST00000456466,ENST00000508628,ENST00000582970
ENST00000247930
ENST00000329143,ENST00000430323,ENST00000533655
ENST00000296701,ENST00000340253,ENST00000394370,ENST00000513826
ENST00000299886
ENST00000296318,ENST00000320057,ENST00000427856,ENST00000463523
ENST00000344579,ENST00000378344
ENST00000368507,ENST00000368508
ENST00000343098,ENST00000424728
ENST00000233242,ENST00000399256
ENST00000296424,ENST00000508136
ENST00000335094
ENST00000341048,ENST00000504958,ENST00000513241
ENST00000321797,ENST00000341547,ENST00000357063,ENST00000377499,ENST00000377534,ENST00000
ENST00000528956
ENST00000299886
ENST00000320481,ENST00000376410,ENST00000396432,ENST00000446003
ENST00000328843,ENST00000409508,ENST00000605912
ENST00000330838,ENST00000358602,ENST00000509867
ENST00000187397,ENST00000337271,ENST00000417925,ENST00000444190,ENST00000457165,ENST00000
ENST00000360509,ENST00000396328,ENST00000557913,ENST00000559418
ENST00000250559,ENST00000341355,ENST00000378985,ENST00000393436,ENST00000450214,ENST00000
ENST00000299886
ENST00000342175,ENST00000342992,ENST00000359218,ENST00000460472,ENST00000589042,ENST00000
ENST00000265403
ENST00000341415
ENST00000417025
ENST00000526538
ENST00000343776,ENST00000397097
ENST00000245441,ENST00000324330,ENST00000382041,ENST00000382043,ENST00000389868,ENST00000
ENST00000367870,ENST00000367872,ENST00000447624
ENST00000333837,ENST00000395487,ENST00000429094,ENST00000437801,ENST00000452445,ENST00000
ENST00000304166,ENST00000396211,ENST00000409363,ENST00000409489
ENST00000299272,ENST00000551346
ENST00000391675,ENST00000465342
ENST00000375915
ENST00000304166,ENST00000396211,ENST00000409363,ENST00000409489
ENST00000381334,ENST00000506198,ENST00000508404
ENST00000371222
ENST00000391720,ENST00000524407,ENST00000526003,ENST00000527223,ENST00000528412,ENST00000
ENST00000260983,ENST00000409111
ENST00000398276,ENST00000416314,ENST00000444627,ENST00000450689
ENST00000379708,ENST00000416505,ENST00000520655,ENST00000520810,ENST00000520835,ENST00000
ENST00000296318,ENST00000320057,ENST00000427856,ENST00000463523
ENST00000233242,ENST00000399256
ENST00000294671

ENST00000245543
ENST00000394576
ENST00000400454,ENST00000404019
ENST00000391675,ENST00000465342
ENST00000267219,ENST00000351546,ENST00000358679,ENST00000418532,ENST00000441784,ENST000004
ENST00000399604,ENST00000575779
ENST00000380250
ENST00000211998,ENST00000372755,ENST00000436396
ENST00000404801,ENST00000605484
ENST00000338585,ENST00000370576,ENST00000370578,ENST00000414978,ENST00000446225,ENST000005
ENST00000264245
ENST00000358954,ENST00000393376,ENST00000417368,ENST00000431170
ENST00000361713,ENST00000374660,ENST00000602533
ENST00000187397,ENST00000337271,ENST00000417925,ENST00000444190,ENST00000457165,ENST000004
ENST00000248244
ENST00000276297,ENST00000358919,ENST00000512044,ENST00000520226
ENST00000316356,ENST00000389960,ENST00000524452,ENST00000527523,ENST00000528231
ENST00000528956
ENST00000293761,ENST00000545513,ENST00000570836,ENST00000574640
ENST00000601382
ENST00000245543
ENST00000398276,ENST00000416314,ENST00000425689,ENST00000444627,ENST00000450689
ENST00000260983,ENST00000409111
ENST00000358954,ENST00000393376,ENST00000417368,ENST00000431170
ENST00000380250
ENST00000296318,ENST00000320057,ENST00000427856,ENST00000463523
ENST00000381334,ENST00000506198,ENST00000508404
ENST00000233242,ENST00000399256
ENST00000404801,ENST00000605484
ENST00000294671
ENST00000528956
ENST00000391720,ENST00000524407,ENST00000526003,ENST00000527223,ENST00000528412,ENST000005
ENST00000601382
ENST00000399604,ENST00000575779
ENST00000187397,ENST00000337271,ENST00000417925,ENST00000444190,ENST00000457165,ENST000004
ENST00000264245
ENST00000379708,ENST00000416505,ENST00000520655,ENST00000520810,ENST00000520835,ENST000005
ENST00000248244
ENST00000371222
ENST00000304166,ENST00000396211,ENST00000409363,ENST00000409489
ENST00000316356,ENST00000389960,ENST00000524452,ENST00000527523,ENST00000528231
ENST00000211998,ENST00000372755,ENST00000436396
ENST00000394576
ENST00000391675,ENST00000465342
ENST00000338585,ENST00000370576,ENST00000370578,ENST00000414978,ENST00000446225,ENST000005
ENST00000400454,ENST00000404019
ENST00000276297,ENST00000358919,ENST00000512044,ENST00000520226

ENST00000293761,ENST00000545513,ENST00000570836,ENST00000574640
ENST00000267219,ENST00000351546,ENST00000358679,ENST00000418532,ENST00000441784,ENST00000[illegible]
ENST00000361713,ENST00000374660,ENST00000602533
ENST00000373361,ENST00000373367,ENST00000541184
ENST00000319651,ENST00000334962,ENST00000339474,ENST00000400877,ENST00000584306
ENST00000355650,ENST00000589717
ENST00000360151
ENST00000366261
ENST00000397104,ENST00000596209
ENST00000409627
ENST00000338977,ENST00000343769,ENST00000343776,ENST00000343776,ENST00000354959,ENST00000[illegible]
ENST00000316300,ENST00000447678
ENST00000336917,ENST00000409343,ENST00000409692,ENST00000410066
ENST00000005082,ENST00000343338,ENST00000354599,ENST00000354959,ENST00000397126,ENST00000[illegible]
ENST00000420699,ENST00000461153
ENST00000307355,ENST00000311048,ENST00000344519,ENST00000355650,ENST00000357491,ENST00000[illegible]
ENST00000594947
ENST00000328085
ENST00000357491,ENST00000601693
ENST00000424293
ENST00000280083,ENST00000341502,ENST00000341749,ENST00000348007,ENST00000396158,ENST00000[illegible]
ENST00000551456
ENST00000066544,ENST00000446365,ENST00000526866,ENST00000527547,ENST00000531206
ENST00000282391,ENST00000380958,ENST00000399613
ENST00000279575,ENST00000445719,ENST00000535904
ENST00000356929,ENST00000357491,ENST00000397126,ENST00000594710,ENST00000601693
ENST00000537253
ENST00000418100
ENST00000419098,ENST00000594710
ENST00000420699,ENST00000461153
ENST00000343769
ENST00000358047
ENST00000355849,ENST00000524199
ENST00000359973,ENST00000389534,ENST00000426391,ENST00000594295
ENST00000326972,ENST00000424053,ENST00000429201
ENST00000594947
ENST00000362068
ENST00000286744,ENST00000567476
ENST00000594363,ENST00000594947,ENST00000600162
ENST00000462323,ENST00000463781,ENST00000466475,ENST00000470451,ENST00000475231,ENST00000[illegible]
ENST00000335117
ENST00000395385,ENST00000402377
ENST00000377868,ENST00000377873,ENST00000536626,ENST00000544874
ENST00000388718
ENST00000547458
ENST00000594710
ENST00000344930,ENST00000395391,ENST00000423627

ENST00000356245,ENST00000394123,ENST00000507878,ENST00000520006,ENST00000520177,ENST00000[illegible]
ENST00000362068
ENST00000418063
ENST00000447333
ENST00000280083,ENST00000341502,ENST00000341749,ENST00000348007,ENST00000396158,ENST00000[illegible]
ENST00000359265
ENST00000358353,ENST00000397542
ENST00000360151
ENST00000297405,ENST00000339701,ENST00000343508,ENST00000352409,ENST00000455883
ENST00000311048,ENST00000355650,ENST00000456783,ENST00000589717
ENST00000318220,ENST00000318249,ENST00000434838,ENST00000454000
ENST00000295206
ENST00000357491
ENST00000458035,ENST00000595091
ENST00000316902,ENST00000422941,ENST00000453702,ENST00000529705
ENST00000427401
ENST00000343776,ENST00000397097
ENST00000390601
ENST00000343769
ENST00000601440
ENST00000357491,ENST00000601693
ENST00000429990
ENST00000341056
ENST00000558241
ENST00000550760
ENST00000456783
ENST00000333796
ENST00000240499
ENST00000368801
ENST00000355095,ENST00000423484
ENST00000328178,ENST00000345030,ENST00000550760,ENST00000596534,ENST00000601023
ENST00000343776,ENST00000397097,ENST00000440339,ENST00000594710
ENST00000325437,ENST00000355803,ENST00000397514,ENST00000397515,ENST00000403747,ENST00000[illegible]
ENST00000343776,ENST00000397097
ENST00000256544,ENST00000561270
ENST00000344519
ENST00000419098
ENST00000397126
ENST00000300619,ENST00000354959,ENST00000354959,ENST00000354959,ENST00000357491,ENST00000[illegible]
ENST00000343776,ENST00000397097
ENST00000357491,ENST00000601693
ENST00000357491,ENST00000397104,ENST00000596209,ENST00000601693
ENST00000251203,ENST00000557978,ENST00000558222
ENST00000357491,ENST00000601693
ENST00000355650,ENST00000589717,ENST00000592725
ENST00000354959,ENST00000594012,ENST00000595461,ENST00000598381
ENST00000354959,ENST00000594012,ENST00000595461,ENST00000598381

ENST00000354959,ENST00000395385,ENST00000402377,ENST00000594012,ENST00000595461,ENST00000
ENST00000397126,ENST00000594710
ENST00000280083,ENST00000341502,ENST00000341749,ENST00000348007,ENST00000396158,ENST00000
ENST00000427401
ENST00000373361,ENST00000373367,ENST00000541184
ENST00000458035,ENST00000595091
ENST00000537253
ENST00000547458
ENST00000319651,ENST00000334962,ENST00000339474,ENST00000400877,ENST00000584306
ENST00000551456
ENST00000409627
ENST00000336917,ENST00000409343,ENST00000409692,ENST00000410066
ENST00000550760
ENST00000395385,ENST00000402377
ENST00000341056
ENST00000343769
ENST00000390601
ENST00000282391,ENST00000380958,ENST00000399613
ENST00000316902,ENST00000422941,ENST00000453702,ENST00000529705
ENST00000362068
ENST00000359265
ENST00000357491,ENST00000601693
ENST00000377868,ENST00000377873,ENST00000536626,ENST00000544874
ENST00000359973,ENST00000389534,ENST00000426391,ENST00000594295
ENST00000447333
ENST00000344930,ENST00000395391,ENST00000423627
ENST00000343776,ENST00000397097
ENST00000297405,ENST00000339701,ENST00000343508,ENST00000352409,ENST00000455883
ENST00000462323,ENST00000463781,ENST00000466475,ENST00000470451,ENST00000475231,ENST00000
ENST00000307355,ENST00000359735,ENST00000397136,ENST00000437743,ENST00000440155
ENST00000335117
ENST00000251203,ENST00000557978,ENST00000558222
ENST00000066544,ENST00000446365,ENST00000526866,ENST00000527547,ENST00000531206
ENST00000419098
ENST00000357491
ENST00000594710
ENST00000397126
ENST00000357491,ENST00000601693
ENST00000256544,ENST00000561270
ENST00000356929
ENST00000328178,ENST00000345030,ENST00000596534,ENST00000601023
ENST00000418063
ENST00000357491,ENST00000601693
ENST00000357491,ENST00000601693
ENST00000318220,ENST00000318249,ENST00000434838,ENST00000454000
ENST00000397104,ENST00000596209
ENST00000005082,ENST00000343338,ENST00000354599,ENST00000399602,ENST00000429541,ENST00000

ENST00000356245,ENST00000394123,ENST00000507878,ENST00000520006,ENST00000520177,ENST00000
ENST00000354959,ENST00000594012,ENST00000595461,ENST00000598381
ENST00000354959,ENST00000594012,ENST00000595461,ENST00000598381
ENST00000388718
ENST00000343776,ENST00000397097,ENST00000440339
ENST00000418100
ENST00000594947,ENST00000594947
ENST00000328085,ENST00000360151
ENST00000316300,ENST00000447678
ENST00000397126
ENST00000343769
ENST00000311048
ENST00000456783
ENST00000300619,ENST00000397082
ENST00000338977,ENST00000511833
ENST00000420699,ENST00000461153,ENST00000420699,ENST00000461153
ENST00000594947
ENST00000355650,ENST00000589717
ENST00000240499
ENST00000325437,ENST00000355803,ENST00000397514,ENST00000397515,ENST00000403747,ENST00000
ENST00000333796
ENST00000368801
ENST00000360151,ENST00000360151
ENST00000419098
ENST00000429990
ENST00000344519
ENST00000424293
ENST00000594710
ENST00000601440
ENST00000354959,ENST00000594012,ENST00000595461,ENST00000598381
ENST00000343776,ENST00000397097
ENST00000355095,ENST00000423484
ENST00000357491,ENST00000601693
ENST00000355650,ENST00000589717
ENST00000355849,ENST00000524199
ENST00000366261
ENST00000295206
ENST00000286744,ENST00000567476
ENST00000358047
ENST00000358353,ENST00000397542
ENST00000362068
ENST00000343776,ENST00000397097
ENST00000326972,ENST00000424053,ENST00000429201
ENST00000558241
ENST00000279575,ENST00000445719,ENST00000535904
ENST00000373361,ENST00000373367,ENST00000541184
ENST00000287534,ENST00000370652,ENST00000394141,ENST00000394143,ENST00000412101

ENST00000066544,ENST00000446365,ENST00000526866,ENST00000527547,ENST00000531206,ENST00000[illegible]
ENST00000360540,ENST00000522514,ENST00000544451
ENST00000304992,ENST00000572621
ENST00000495679
ENST00000551456
ENST00000192788,ENST00000452449
ENST00000301831,ENST00000420927
ENST00000362068
ENST00000264162
ENST00000265071,ENST00000514738
ENST00000332645,ENST00000398868,ENST00000538786,ENST00000540699
ENST00000362068
ENST00000542267
ENST00000289953,ENST00000357199
ENST00000317881
ENST00000297405,ENST00000339701,ENST00000343508,ENST00000352409,ENST00000455883
ENST00000264852,ENST00000393830
ENST00000301831,ENST00000420927
ENST00000360540,ENST00000522514,ENST00000544451
ENST00000373361,ENST00000373367,ENST00000541184
ENST00000317881
ENST00000264852,ENST00000393830
ENST00000533415
ENST00000551456
ENST00000332645,ENST00000398868,ENST00000538786,ENST00000540699
ENST00000495679
ENST00000362068
ENST00000265071,ENST00000514738
ENST00000304992,ENST00000572621
ENST00000542267
ENST00000192788,ENST00000452449
ENST00000264162
ENST00000287534,ENST00000370652,ENST00000394141,ENST00000394143,ENST00000412101
ENST00000297405,ENST00000339701,ENST00000343508,ENST00000352409,ENST00000455883
ENST00000289953,ENST00000357199
ENST00000362068
ENST00000373361,ENST00000373367,ENST00000541184
ENST00000287534,ENST00000370652,ENST00000394141,ENST00000394143,ENST00000412101
ENST00000300870
ENST00000331162,ENST00000342944,ENST00000354959,ENST00000357002,ENST00000420713,ENST00000[illegible]
ENST00000595405,ENST00000601440
ENST00000539932
ENST00000434783
ENST00000261560,ENST00000344519,ENST00000397104,ENST00000596143,ENST00000596209,ENST00000[illegible]
ENST00000342960
ENST00000301831,ENST00000420927
ENST00000495679

ENST00000551456
ENST00000597976
ENST00000066544,ENST00000446365,ENST00000526866,ENST00000527547,ENST00000531206
ENST00000357491,ENST00000594710,ENST00000601693
ENST00000596143,ENST00000600435
ENST00000419098
ENST00000301831,ENST00000420927
ENST00000355849,ENST00000524199
ENST00000397104,ENST00000596209
ENST00000362068
ENST00000264162
ENST00000332645,ENST00000398868,ENST00000538786,ENST00000540699
ENST00000250974,ENST00000292577,ENST00000590661,ENST00000591351
ENST00000344519,ENST00000397104,ENST00000596143,ENST00000596209,ENST00000600435
ENST00000261560
ENST00000542267
ENST00000548032
ENST00000289953,ENST00000357199
ENST00000596143,ENST00000600435
ENST00000342944,ENST00000357002
ENST00000297405,ENST00000339701,ENST00000343508,ENST00000352409,ENST00000455883
ENST00000420713
ENST00000274609
ENST00000395385,ENST00000402377
ENST00000594710
ENST00000300619,ENST00000339986,ENST00000354959,ENST00000397082,ENST00000418100,ENST00000[illegible]
ENST00000418100
ENST00000339986
ENST00000420713
ENST00000354959,ENST00000594012,ENST00000595461,ENST00000598381
ENST00000301831,ENST00000420927,ENST00000301831,ENST00000420927
ENST00000373361,ENST00000373367,ENST00000541184
ENST00000597976
ENST00000551456
ENST00000332645,ENST00000398868,ENST00000538786,ENST00000540699
ENST00000495679
ENST00000434783
ENST00000362068
ENST00000342960
ENST00000542267
ENST00000539932
ENST00000344519
ENST00000066544,ENST00000446365,ENST00000526866,ENST00000527547,ENST00000531206
ENST00000357491,ENST00000601693
ENST00000596143,ENST00000600435
ENST00000420713
ENST00000264162

ENST00000287534,ENST00000370652,ENST00000394141,ENST00000394143,ENST00000412101
ENST00000420713
ENST00000261560,ENST00000261560
ENST00000397104,ENST00000596209
ENST00000250974,ENST00000292577,ENST00000590661,ENST00000591351
ENST00000339986
ENST00000261560
ENST00000274609
ENST00000300870
ENST00000297405,ENST00000339701,ENST00000343508,ENST00000352409,ENST00000455883
ENST00000342944,ENST00000357002
ENST00000300619,ENST00000397082
ENST00000596143,ENST00000600435
ENST00000595405,ENST00000601440
ENST00000395385,ENST00000402377
ENST00000419098
ENST00000289953,ENST00000357199
ENST00000594710
ENST00000418100
ENST00000331162
ENST00000354959,ENST00000594012,ENST00000595461,ENST00000598381
ENST00000548032
ENST00000355849,ENST00000524199
ENST00000373361,ENST00000373367,ENST00000541184
ENST00000434783
ENST00000287534,ENST00000370652,ENST00000394141,ENST00000394143,ENST00000412101
ENST00000434783
ENST00000495679
ENST00000551456
ENST00000066544,ENST00000446365,ENST00000526866,ENST00000527547,ENST00000531206
ENST00000362068
ENST00000264162
ENST00000265071,ENST00000514738
ENST00000542267
ENST00000415164
ENST00000289953,ENST00000357199
ENST00000548213
ENST00000264852,ENST00000393830
ENST00000373361,ENST00000373367,ENST00000541184
ENST00000264852,ENST00000393830
ENST00000551456
ENST00000548213
ENST00000495679
ENST00000434783,ENST00000434783
ENST00000362068
ENST00000265071,ENST00000514738
ENST00000542267

ENST00000415164
ENST00000066544,ENST00000446365,ENST00000526866,ENST00000527547,ENST00000531206
ENST00000264162
ENST00000287534,ENST00000370652,ENST00000394141,ENST00000394143,ENST00000412101
ENST00000434783
ENST00000289953,ENST00000357199
ENST00000566450
ENST00000271688,ENST00000368949,ENST00000368954,ENST00000561294
ENST00000373361,ENST00000373367,ENST00000541184
ENST00000287534,ENST00000370652,ENST00000394141,ENST00000394143,ENST00000412101
ENST00000357491,ENST00000601693
ENST00000495679
ENST00000551456
ENST00000407622
ENST00000530440
ENST00000355849,ENST00000524199
ENST00000362068
ENST00000264162
ENST00000265071,ENST00000514738
ENST00000362068
ENST00000542267
ENST00000289953,ENST00000357199
ENST00000520112
ENST00000361789
ENST00000264852,ENST00000393830
ENST00000373361,ENST00000373367,ENST00000541184
ENST00000264852,ENST00000393830
ENST00000551456
ENST00000495679
ENST00000271688,ENST00000368949,ENST00000368954,ENST00000561294
ENST00000362068
ENST00000265071,ENST00000514738
ENST00000542267
ENST00000361789
ENST00000407622
ENST00000264162
ENST00000287534,ENST00000370652,ENST00000394141,ENST00000394143,ENST00000412101
ENST00000530440
ENST00000357491,ENST00000601693
ENST00000289953,ENST00000357199
ENST00000520112
ENST00000355849,ENST00000524199
ENST00000566450
ENST00000362068
ENST00000264515,ENST00000367164
ENST00000226253,ENST00000395319,ENST00000395321
ENST00000333371,ENST00000535843,ENST00000535906

ENST00000326587,ENST00000375695,ENST00000375722,ENST00000375772
ENST00000355281
ENST00000356878,ENST00000512229,ENST00000530339
ENST00000268876,ENST00000378449,ENST00000394570,ENST00000433649,ENST00000591048
ENST00000379485,ENST00000499385
ENST00000298232,ENST00000342420,ENST00000361285
ENST00000480623
ENST00000337908,ENST00000355131,ENST00000395016,ENST00000532114
ENST00000262374,ENST00000544428,ENST00000588623
ENST00000308488,ENST00000360355,ENST00000480887,ENST00000531026
ENST00000472091,ENST00000542862
ENST00000261292,ENST00000577628,ENST00000580036
ENST00000305799,ENST00000409262
ENST00000226253,ENST00000395319,ENST00000395321
ENST00000356878,ENST00000512229,ENST00000530339
ENST00000326587,ENST00000375695,ENST00000375722,ENST00000375772
ENST00000337908,ENST00000355131,ENST00000395016,ENST00000532114
ENST00000298232,ENST00000342420,ENST00000361285
ENST00000268876,ENST00000378449,ENST00000394570,ENST00000433649,ENST00000591048
ENST00000379485,ENST00000499385
ENST00000262374,ENST00000544428,ENST00000588623
ENST00000480623
ENST00000472091,ENST00000542862
ENST00000355281
ENST00000308488,ENST00000360355,ENST00000480887,ENST00000531026
ENST00000305799,ENST00000409262
ENST00000333371,ENST00000535843,ENST00000535906
ENST00000264515,ENST00000367164
ENST00000261292,ENST00000577628,ENST00000580036
ENST00000000442,ENST00000405666,ENST00000406310
ENST00000392825,ENST00000409237
ENST00000308873,ENST00000338888,ENST00000399916,ENST00000540420
ENST00000261609,ENST00000564734
ENST00000299314,ENST00000549940
ENST00000320316,ENST00000454255,ENST00000570899,ENST00000571362,ENST00000573826
ENST00000000442,ENST00000405666,ENST00000406310,ENST00000545035
ENST00000308873,ENST00000338888,ENST00000399916,ENST00000540420
ENST00000261609,ENST00000564734
ENST00000299314,ENST00000549940
ENST00000320316,ENST00000454255,ENST00000570899,ENST00000571362,ENST00000573826
ENST00000392825,ENST00000409237
ENST00000304414,ENST00000546206,ENST00000567078
ENST00000217254,ENST00000381944
ENST00000341776,ENST00000397311
ENST00000237455
ENST00000373644
ENST00000427255

ENST00000265724,ENST00000543898
ENST00000373275
ENST00000242465
ENST00000338647
ENST00000518907,ENST00000520284
ENST00000422453,ENST00000441626,ENST00000451728,ENST00000504813,ENST00000512338
ENST00000303749,ENST00000396721,ENST00000522671
ENST00000380229
ENST00000389840,ENST00000585328
ENST00000216190,ENST00000405442,ENST00000455547,ENST00000541106
ENST00000318388,ENST00000400033
ENST00000353479,ENST00000369733,ENST00000393211
ENST00000301938,ENST00000308799,ENST00000395665,ENST00000395667
ENST00000398559,ENST00000543976
ENST00000287647,ENST00000383806,ENST00000383807,ENST00000419585
ENST00000288040,ENST00000393701,ENST00000449317,ENST00000568461
ENST00000367070
ENST00000301938,ENST00000308799,ENST00000395665,ENST00000395667
ENST00000217254,ENST00000381944
ENST00000427255
ENST00000341776,ENST00000397311
ENST00000373275
ENST00000288040,ENST00000393701,ENST00000449317,ENST00000568461
ENST00000304414,ENST00000546206,ENST00000567078
ENST00000237455
ENST00000389840,ENST00000585328
ENST00000380229
ENST00000367070
ENST00000398559,ENST00000543976
ENST00000216190,ENST00000405442,ENST00000455547,ENST00000541106
ENST00000265724,ENST00000543898
ENST00000446936,ENST00000502976
ENST00000303749,ENST00000396721,ENST00000522671
ENST00000338647
ENST00000373644
ENST00000242465
ENST00000318388,ENST00000400033
ENST00000353479,ENST00000369733,ENST00000393211
ENST00000287647,ENST00000383806,ENST00000383807,ENST00000419585
ENST00000518907,ENST00000520284
ENST00000398706,ENST00000603114,ENST00000604524,ENST00000604729,ENST00000605216
ENST00000339953
ENST00000345005,ENST00000352989,ENST00000395066,ENST00000563842,ENST00000566926
ENST00000370598
ENST00000400664
ENST00000261532,ENST00000380887,ENST00000505752,ENST00000509242,ENST00000512784,ENST00000[illegible]
ENST00000272746,ENST00000359761,ENST00000392546,ENST00000392547,ENST00000409891

ENST00000464439
ENST00000246012,ENST00000449810
ENST00000270223,ENST00000377735
ENST00000310931,ENST00000391975,ENST00000391976,ENST00000427183
ENST00000340446
ENST00000254579,ENST00000527990
ENST00000392027
ENST00000270223,ENST00000377735
ENST00000400664
ENST00000254579,ENST00000527990
ENST00000398706,ENST00000603114,ENST00000604524,ENST00000604729,ENST00000605216
ENST00000345005,ENST00000352989,ENST00000395066,ENST00000563842,ENST00000566926
ENST00000272746,ENST00000359761,ENST00000392546,ENST00000392547,ENST00000409891
ENST00000464439
ENST00000339953
ENST00000392027
ENST00000246012,ENST00000449810
ENST00000261532,ENST00000380887,ENST00000505752,ENST00000509242,ENST00000512784,ENST00000[illegible]
ENST00000370598
ENST00000340446
ENST00000310931,ENST00000391975,ENST00000391976,ENST00000427183
ENST00000233609,ENST00000585665,ENST00000586096,ENST00000586656,ENST00000586686,ENST00000[illegible]
ENST00000354981,ENST00000355755,ENST00000408042
ENST00000249373
ENST00000231137
ENST00000462323,ENST00000463781,ENST00000466475,ENST00000470451,ENST00000475231,ENST00000[illegible]
ENST00000296498
ENST00000359191,ENST00000402194,ENST00000432763,ENST00000598725,ENST00000599117
ENST00000302787
ENST00000400681
ENST00000291281,ENST00000433867,ENST00000595515,ENST00000600194,ENST00000601806
ENST00000377984,ENST00000388950,ENST00000423537
ENST00000249373
ENST00000302787
ENST00000231137
ENST00000233609,ENST00000586096,ENST00000586686,ENST00000589656,ENST00000593052
ENST00000291281,ENST00000433867,ENST00000595515,ENST00000600194,ENST00000601806
ENST00000377984,ENST00000388950,ENST00000423537
ENST00000354981,ENST00000355755,ENST00000408042
ENST00000359191,ENST00000402194,ENST00000432763,ENST00000598725,ENST00000599117
ENST00000462323,ENST00000463781,ENST00000466475,ENST00000470451,ENST00000475231,ENST00000[illegible]
ENST00000296498
ENST00000400681

61

.415,NM_001291416,NM_001291417,NM_001291418,NM_001291421,NM_021140

ʌ_153186

ʌ_012196

3717,NM_001332

.033,NM_004727

3412,NM_001258413,NM_001258414,NM_052944

.415,NM_001291416,NM_001291417,NM_001291418,NM_001291421,NM_021140

4846,NM_001274

55

5272,NM_003925
M_012196

3000,NM_005869

3717,NM_001332

415,NM_001291416,NM_001291417,NM_001291418,NM_001291421,NM_021140

000268,NM_016418,NM_181825,NM_181828,NM_181829,NM_181830,NM_181831,NM_181832

61

M_025008

477,NM_001318478,NM_001318479,NM_015516

4,NM_001318091,NM_001318093,NM_001318094
.128224,NM_001128224

.303232,NM_001303232,NM_005807,NM_005807,NM_001127708,NM_001127708,NM_001127709,NM_001

3717,NM_001332
.142530,NM_030639,NM_001142524,NM_001142525,NM_001142526,NM_001142527,NM_001142528,NM_

.269046,NM_001269047,NM_001269048,NM_001269051,NM_001269052,NM_080618,NM_001269040,NM_

.303232,NM_001303232,NM_005807,NM_005807

.269046,NM_001269047,NM_001269048,NM_001269051,NM_001269052,NM_080618

.142530,NM_030639

M_012196
55
.015560,NM_130831,NM_130832,NM_130833,NM_130834,NM_130835,NM_130836,NM_130837

4,NM_001318091,NM_001318093,NM_001318094

[illegible]717,NM_001332

[illegible]709,NM_001127710,NM_001303232,NM_005807
[illegible],NM_001288975,NM_003474,NM_021641

/I_012196

.015560,NM_130831,NM_130832,NM_130833,NM_130834,NM_130835,NM_130836,NM_130837

3717,NM_001332

'709,NM_001127710,NM_001303232,NM_005807

2508,NM_001302509,NM_001302510,NM_004530

015560,NM_130831,NM_130832,NM_130833,NM_130834,NM_130835,NM_130836,NM_130837

.318796,NM_003264,NM_001318787,NM_001318789,NM_001318790,NM_001318791,NM_001318793,NM_
2390,NM_000888,NM_001282353,NM_001282354,NM_001282355,NM_001282388,NM_001282389,NM_001

.318796,NM_003264

2390

3,NM_001195799,NM_001195800,NM_001195803

1322830,NM_001322832,NM_001322835,NM_001322836,NM_001322837,NM_001322838,NM_002247,NM_

3,NM_001243730,NM_001243731,NM_001243732

1322830,NM_001322832,NM_001322835,NM_001322836,NM_001322837,NM_001322838,NM_002247

4393,NM_001164380,NM_001164381,NM_001164382,NM_001164383,NM_001164384,NM_001164385,NM_

61

5058,NM_001190348,NM_001190349,NM_033317

4393

/I_012196

61

.322886,NM_001322887,NM_001322888,NM_001322889,NM_001322890,NM_001322891,NM_001322892,N

5058,NM_001190348,NM_001190349,NM_033317

.322886,NM_001322887,NM_001322888,NM_001322889,NM_001322890,NM_001322891,NM_001322892,N

61
)532,NM_207521,NM_001321859,NM_001321860,NM_001321861,NM_001321862,NM_001321863,NM_001

.322886,NM_001322887,NM_001322888,NM_001322889,NM_001322890,NM_001322891,NM_001322892,N

1,NM_001318091,NM_001318093,NM_001318094

4054,NM_004549
5058,NM_001190348,NM_001190349,NM_033317

/I_012485

/I_182982

.322886,NM_001322887,NM_001322888,NM_001322889,NM_001322890,NM_001322891,NM_001322892,N

)532,NM_207521

.322886,NM_001322887,NM_001322888,NM_001322889,NM_001322890,NM_001322891,NM_001322892,N

.322886,NM_001322887,NM_001322888,NM_001322889,NM_001322890,NM_001322891,NM_001322892,N

3376,NM_021235

.868,NM_001516

3842,NM_001318847,NM_001318848,NM_001318857,NM_001318858,NM_006586

)162,NM_006677

_000268,NM_016418,NM_181825,NM_181828,NM_181829,NM_181830,NM_181831,NM_181832

47
5583,NM_020901

_198838,NM_198839
3832,NM_000548,NM_001077183,NM_001114382,NM_001318827,NM_001318829,NM_001318831,NM_001

71,NM_020952,NM_024971,NM_206944,NM_206945,NM_206946,NM_206947

3832

M_012196

_000268,NM_016418,NM_181825,NM_181828,NM_181829,NM_181830,NM_181831,NM_181832

/I_012196

61
/I_012196

i488,NM_004487

3630,NM_032643

_000268,NM_016418,NM_181825,NM_181828,NM_181829,NM_181830,NM_181831,NM_181832
.318119,NM_001318120,NM_016211,NM_001077206,NM_001077207,NM_001077208,NM_001191049,NM_

.318119,NM_001318120,NM_016211

3148,NM_015575

/I_145862

'709,NM_001127710,NM_001303232,NM_005807

!899,NM_001282900,NM_001282901,NM_007023

2899,NM_001282900,NM_001282901,NM_007023

/I_012196

;500,NM_003905

;500,NM_003905

;500,NM_003905

;500,NM_003905

)238,NM_001199239,NM_001199240,NM_033177
)153,NM_001242868,NM_001242869,NM_001242870,NM_001242871,NM_144595

181832,NM_181833

181832,NM_181833

323086,NM_001323087,NM_001323088,NM_001323090
181832,NM_181833

_181832,NM_181833

_181833_181832,NM_181833

2,NM_001291628,NM_030621,NM_177438

449052,ENST00000538115,ENST00000542585,ENST00000546292

445192,ENST00000533951

409914
445192

356920,ENST00000357462,ENST00000357462,ENST00000357462,ENST00000357462,ENST00000357462,ENST

358464,ENST00000439954,ENST00000439954,ENST00000439954

348028,ENST00000348028,ENST00000348028,ENST00000348028,ENST00000349350,ENST00000349350,ENST
367482,ENST00000367482,ENST00000367483,ENST00000367483,ENST00000367483,ENST00000367483,ENST

355078,ENST00000355078,ENST00000355078,ENST00000355078,ENST00000355078,ENST00000518825,ENST
355193,ENST00000558084,ENST00000558084,ENST00000558084
360967,ENST00000360967,ENST00000360967,ENST00000361163,ENST00000361163,ENST00000361163,ENST
367484,ENST00000367484,ENST00000367484,ENST00000367484,ENST00000367484,ENST00000367485,ENST
367482,ENST00000367483,ENST00000367483,ENST00000367483,ENST00000367483,ENST00000367483,ENST
355193,ENST00000424877,ENST00000424877,ENST00000424877

449052,ENST00000538115,ENST00000542585,ENST00000546292

555528,ENST00000555926
445192,ENST00000533951

409914
445192

349310,ENST00000349310,ENST00000402615,ENST00000402615,ENST00000402615,ENST00000402615,ENST

356920,ENST00000357462,ENST00000357462,ENST00000357462,ENST00000357462,ENST00000357462,ENST
357462,ENST00000357462,ENST00000357462,ENST00000360967,ENST00000360967,ENST00000360967,ENST

358464,ENST00000439954,ENST00000439954,ENST00000439954

348028,ENST00000348028,ENST00000348028,ENST00000348028,ENST00000349350,ENST00000349350,ENST
367482,ENST00000367482,ENST00000367483,ENST00000367483,ENST00000367483,ENST00000367483,ENST

355078,ENST00000355078,ENST00000355078,ENST00000355078,ENST00000355078,ENST00000518825,ENST
355193,ENST00000558084,ENST00000558084,ENST00000558084
360967,ENST00000360967,ENST00000360967,ENST00000361163,ENST00000361163,ENST00000361163,ENST
357462,ENST00000357462,ENST00000357462,ENST00000360967,ENST00000360967,ENST00000360967,ENST

367484,ENST00000367484,ENST00000367484,ENST00000367484,ENST00000367484,ENST00000367485,ENST
367482,ENST00000367483,ENST00000367483,ENST00000367483,ENST00000367483,ENST00000367483,ENST
355193,ENST00000424877,ENST00000424877,ENST00000424877

506773,ENST00000511167,ENST00000511328,ENST00000512017,ENST00000513186

555375,ENST00000556155

393494,ENST00000393498,ENST00000393500,ENST00000403559,ENST00000408937

448038,ENST00000537033,ENST00000540663

368282,ENST00000368282,ENST00000368282,ENST00000368282,ENST00000368282,ENST00000368284,ENST

350908,ENST00000350908,ENST00000350908,ENST00000350908,ENST00000350908,ENST00000360232,ENST

300134,ENST00000300134,ENST00000454075,ENST00000454075,ENST00000454075,ENST00000454075,ENST
357825,ENST00000543397,ENST00000543397,ENST00000543397
542587,ENST00000586703,ENST00000586703,ENST00000586703

520539,ENST00000521922,ENST00000521922,ENST00000521922
310816,ENST00000310816,ENST00000310816,ENST00000310816,ENST00000310816,ENST00000359221,ENST

358464,ENST00000439954,ENST00000439954,ENST00000439954

265368,ENST00000265368,ENST00000265368,ENST00000341594,ENST00000341594,ENST00000341594,ENST

349384,ENST00000349384,ENST00000349384,ENST00000349384,ENST00000349384,ENST00000379933,ENST
369338,ENST00000369338,ENST00000369338,ENST00000369339,ENST00000369339,ENST00000369339,ENST

447268,ENST00000580206,ENST00000580206,ENST00000580206

375398,ENST00000375398,ENST00000375398,ENST00000375398,ENST00000375398,ENST00000375400,ENST

434526,ENST00000437307,ENST00000451338

602114

506773,ENST00000511167,ENST00000511328,ENST00000512017,ENST00000513186

555375,ENST00000556155

536585

393494,ENST00000393498,ENST00000393500,ENST00000403559,ENST00000408937

591111

557798,ENST00000561030,ENST00000561030,ENST00000561030
368282,ENST00000368282,ENST00000368282,ENST00000368282,ENST00000368282,ENST00000368284,ENST

350908,ENST00000350908,ENST00000350908,ENST00000350908,ENST00000350908,ENST00000360232,ENST

355193,ENST00000558084,ENST00000558084,ENST00000558084
300134,ENST00000300134,ENST00000454075,ENST00000454075,ENST00000454075,ENST00000454075,ENST

542587,ENST00000586703,ENST00000586703,ENST00000586703

520539,ENST00000521922,ENST00000521922,ENST00000521922

373629,ENST00000373629,ENST00000373629,ENST00000373631,ENST00000373631,ENST00000373631,ENST

342175,ENST00000342992,ENST00000342992,ENST00000342992,ENST00000342992,ENST00000342992,ENST

356521,ENST00000356521,ENST00000356521,ENST00000377400,ENST00000377400,ENST00000377400,ENST

310816,ENST00000310816,ENST00000310816,ENST00000310816,ENST00000310816,ENST00000359221,ENST

355385,ENST00000495514,ENST00000495514,ENST00000495514
580762,ENST00000582535,ENST00000582535,ENST00000582535

368331,ENST00000437809,ENST00000437809,ENST00000437809

596931,ENST00000602114,ENST00000602114,ENST00000602114
538346,ENST00000550620,ENST00000550620,ENST00000550620

275607,ENST00000275607,ENST00000275607,ENST00000395435,ENST00000395435,ENST00000395435,ENST

447268,ENST00000580206,ENST00000580206,ENST00000580206

287008,ENST00000357517,ENST00000357517,ENST00000357517,ENST00000357517,ENST00000357517,ENST
375398,ENST00000375398,ENST00000375398,ENST00000375398,ENST00000375398,ENST00000375400,ENST

358464,ENST00000439954,ENST00000439954,ENST00000439954

535621,ENST00000563679,ENST00000567306,ENST00000569715

506773,ENST00000511167,ENST00000511328,ENST00000512017,ENST00000513186
555375,ENST00000556155

393494,ENST00000393498,ENST00000393500,ENST00000403559,ENST00000408937

449052,ENST00000538115,ENST00000542585,ENST00000546292

445192
350908,ENST00000350908,ENST00000350908,ENST00000350908,ENST00000350908,ENST00000360232,ENST

325823,ENST00000325823,ENST00000325823,ENST00000325823,ENST00000396410,ENST00000396410,ENST
300134,ENST00000300134,ENST00000454075,ENST00000454075,ENST00000454075,ENST00000454075,ENST

542587,ENST00000586703,ENST00000586703,ENST00000586703

520539,ENST00000521922,ENST00000521922,ENST00000521922
310816,ENST00000310816,ENST00000310816,ENST00000310816,ENST00000310816,ENST00000359221,ENST

375398,ENST00000375398,ENST00000375398,ENST00000375398,ENST00000375398,ENST00000375400,ENST

348028,ENST00000348028,ENST00000348028,ENST00000348028,ENST00000349350,ENST00000349350,ENST
367482,ENST00000367483,ENST00000367483,ENST00000367483,ENST00000367483,ENST00000367483,ENST

468211
331920,ENST00000375274,ENST00000375274,ENST00000375274,ENST00000375274,ENST00000375274,ENST

355193,ENST00000558665,ENST00000558665,ENST00000558665

429972,ENST00000490882,ENST00000493452
295956,ENST00000295956,ENST00000295956,ENST00000348383,ENST00000348383,ENST00000348383,ENST

370163,ENST00000370165

391828

447207,ENST00000538201

455263,ENST00000509690,ENST00000514944

407925,ENST00000418533

474710,ENST00000481632

536126,ENST00000543718

379249,ENST00000379255,ENST00000379264,ENST00000379272,ENST00000413683,ENST00000431707,ENST

393920,ENST00000393921,ENST00000416020,ENST00000425182,ENST00000430273,ENST00000431963,ENST

596534,ENST00000601023,ENST00000601023

344930,ENST00000356929,ENST00000358296,ENST00000395385,ENST00000395391,ENST00000397082,ENST

449052,ENST00000538115,ENST00000542585,ENST00000546292

521353,ENST00000522736

449052,ENST00000538115,ENST00000542585,ENST00000546292

394616,ENST00000394617,ENST00000489216

358296,ENST00000395391,ENST00000423627,ENST00000596534,ENST00000601023

422782,ENST00000428879,ENST00000428879,ENST00000428879
422782,ENST00000428879,ENST00000428879,ENST00000428879
422782,ENST00000428879,ENST00000428879,ENST00000428879

349807,ENST00000349807,ENST00000349807,ENST00000353004,ENST00000353004,ENST00000353004,ENST
333478,ENST00000333478,ENST00000333478,ENST00000333478,ENST00000333478,ENST00000421160,ENST

397412,ENST00000436138,ENST00000436138,ENST00000436138

393151,ENST00000393151,ENST00000393151,ENST00000553484,ENST00000553484,ENST00000553484,ENST
393006,ENST00000393006,ENST00000393006,ENST00000409658,ENST00000409658,ENST00000409658,ENST

580762,ENST00000582535,ENST00000582535,ENST00000582535
580762,ENST00000582535,ENST00000582535,ENST00000582535
580762,ENST00000582535,ENST00000582535,ENST00000582535
519445,ENST00000521134,ENST00000521134,ENST00000521134

525639,ENST00000527447,ENST00000527447,ENST00000527447
389131,ENST00000389131,ENST00000518083,ENST00000518083,ENST00000518083,ENST00000518083,ENST

376071,ENST00000376071,ENST00000376071,ENST00000376072,ENST00000376072,ENST00000376072,ENST

294724,ENST00000294724,ENST00000361302,ENST00000361302,ENST00000361302,ENST00000361302,ENST

433625,ENST00000433625,ENST00000433625,ENST00000480623,ENST00000480623,ENST00000480623,ENST

452676,ENST00000601469,ENST00000601469,ENST00000601469

594012,ENST00000594012,ENST00000594012,ENST00000595461,ENST00000595461,ENST00000595461,ENST
411502,ENST00000411502,ENST00000411502,ENST00000411502,ENST00000411502,ENST00000546047,ENST

345030,ENST00000345030,ENST00000345030,ENST00000596534,ENST00000596534,ENST00000596534,ENST

395391,ENST00000423627,ENST00000423627,ENST00000423627

296869,ENST00000296869,ENST00000296869,ENST00000296869,ENST00000296869,ENST00000296869,ENST

370891,ENST00000557284,ENST00000557284,ENST00000557284

400226,ENST00000400226,ENST00000400226
395391,ENST00000423627,ENST00000423627,ENST00000423627
598265,ENST00000602198,ENST00000602198,ENST00000602198

401393,ENST00000558357,ENST00000558357,ENST00000558357

277549,ENST00000277551,ENST00000277551,ENST00000277551,ENST00000277551,ENST00000277551,ENST
323594,ENST00000510913,ENST00000510913,ENST00000510913

442594,ENST00000536534,ENST00000536534,ENST00000536534

549091,ENST00000552728,ENST00000552728,ENST00000552728

369338,ENST00000369338,ENST00000369338,ENST00000369339,ENST00000369339,ENST00000369339,ENST
381469,ENST00000432757,ENST00000432757,ENST00000432757

354959,ENST00000354959,ENST00000354959,ENST00000397082,ENST00000397082,ENST00000397082,ENST
559607,ENST00000560079,ENST00000560079,ENST00000560079
358357,ENST00000358357,ENST00000358357,ENST00000358357,ENST00000358357,ENST00000395270,ENST
376237,ENST00000383329,ENST00000383329,ENST00000383329
502818,ENST00000502818,ENST00000502818,ENST00000502818,ENST00000502818,ENST00000511604,ENST

338821,ENST00000402822,ENST00000402822,ENST00000402822

355725,ENST00000491737,ENST00000491737,ENST00000491737

359735,ENST00000359735,ENST00000359735,ENST00000359735,ENST00000359735,ENST00000397136,ENST

573283,ENST00000573283,ENST00000573283,ENST00000573283,ENST00000573283,ENST00000575087,ENST

336119,ENST00000348069,ENST00000348069,ENST00000348069,ENST00000348069,ENST00000348069,ENST
369338,ENST00000490598,ENST00000490598,ENST00000490598

394066,ENST00000394066,ENST00000394066,ENST00000394066,ENST00000394066,ENST00000394067,ENST
374542,ENST00000374542,ENST00000374542,ENST00000374542,ENST00000374542,ENST00000414083,ENST

531805,ENST00000532828,ENST00000532828,ENST00000532828
345030,ENST00000345030,ENST00000345030,ENST00000596534,ENST00000596534,ENST00000596534,ENST
399222,ENST00000399222,ENST00000399222,ENST00000399222,ENST00000399222,ENST00000399232,ENST

376020,ENST00000460112,ENST00000460112,ENST00000460112

359530,ENST00000359530,ENST00000359530,ENST00000396774,ENST00000396774,ENST00000396774,ENST
432063,ENST00000432063,ENST00000432063,ENST00000535545,ENST00000535545,ENST00000535545,ENST
357258,ENST00000357258,ENST00000393785,ENST00000393785,ENST00000393785,ENST00000393785,ENST

276344,ENST00000276344,ENST00000276344,ENST00000276344,ENST00000276344,ENST00000276344,ENST
269844,ENST00000269844,ENST00000398548,ENST00000398548,ENST00000398548,ENST00000398548,ENST

278460,ENST00000278460,ENST00000378615,ENST00000378615,ENST00000378615,ENST00000378615,ENST

354476,ENST00000354476,ENST00000390003,ENST00000390003,ENST00000390003,ENST00000390003,ENST

345030,ENST00000345030,ENST00000345030,ENST00000596534,ENST00000596534,ENST00000596534,ENST

314686,ENST00000314686,ENST00000429348,ENST00000429348,ENST00000429348,ENST00000429348,ENST
401494,ENST00000401494,ENST00000401494,ENST00000401494,ENST00000401494,ENST00000415165,ENST

378526,ENST00000378526,ENST00000378526,ENST00000585941,ENST00000585941,ENST00000585941,ENST

269305,ENST00000269305,ENST00000269305,ENST00000359597,ENST00000359597,ENST00000359597,ENST

343984,ENST00000343984,ENST00000343984,ENST00000354228,ENST00000354228,ENST00000354228,ENST

393151,ENST00000393151,ENST00000393151,ENST00000553484,ENST00000553484,ENST00000553484,ENST

348028,ENST00000348028,ENST00000348028,ENST00000348028,ENST00000348028,ENST00000348028,ENST

348028,ENST00000348028,ENST00000348028,ENST00000348028,ENST00000349350,ENST00000349350,ENST

465038,ENST00000541979

370163,ENST00000370165

455263,ENST00000509690,ENST00000514944

379249,ENST00000379255,ENST00000379264,ENST00000379272,ENST00000413683,ENST00000431707,ENST

349807,ENST00000349807,ENST00000349807,ENST00000353004,ENST00000353004,ENST00000353004,ENST
349097,ENST00000349097,ENST00000349097,ENST00000395477,ENST00000395477,ENST00000395477,ENST
397412,ENST00000436138,ENST00000436138,ENST00000436138

309065,ENST00000309065,ENST00000309065,ENST00000506583,ENST00000506583,ENST00000506583,ENST
360488,ENST00000377850,ENST00000377850,ENST00000377850
360488,ENST00000377850,ENST00000377850,ENST00000377850
525639,ENST00000527447,ENST00000527447,ENST00000527447
389131,ENST00000389131,ENST00000518083,ENST00000518083,ENST00000518083,ENST00000518083,ENST

294724,ENST00000294724,ENST00000361302,ENST00000361302,ENST00000361302,ENST00000361302,ENST

379500,ENST00000533953,ENST00000533953,ENST00000533953

352853,ENST00000352853,ENST00000359524,ENST00000359524,ENST00000359524,ENST00000359524,ENST

420871,ENST00000456556,ENST00000456556,ENST00000456556
296869,ENST00000296869,ENST00000296869,ENST00000296869,ENST00000296869,ENST00000296869,ENST
585744,ENST00000588462,ENST00000588462,ENST00000588462
357674,ENST00000450955,ENST00000450955,ENST00000450955

323594,ENST00000510913,ENST00000510913,ENST00000510913
442594,ENST00000536534,ENST00000536534,ENST00000536534
446747,ENST00000446747,ENST00000446747,ENST00000528372,ENST00000528372,ENST00000528372,ENST
549091,ENST00000552728,ENST00000552728,ENST00000552728

355725,ENST00000491737,ENST00000491737,ENST00000491737

370918,ENST00000370918,ENST00000370918,ENST00000541407,ENST00000541407,ENST00000541407,ENST

376020,ENST00000460112,ENST00000460112,ENST00000460112

522628,ENST00000543296,ENST00000543296,ENST00000543296

508491,ENST00000543251,ENST00000543251,ENST00000543251

520539,ENST00000521922,ENST00000521922,ENST00000521922

348639,ENST00000559658,ENST00000559658,ENST00000559658
269305,ENST00000269305,ENST00000269305,ENST00000359597,ENST00000359597,ENST00000359597,ENST

434436,ENST00000434436,ENST00000434436,ENST00000590550,ENST00000590550,ENST00000590550,ENST

355327,ENST00000357769,ENST00000357769,ENST00000357769

465038,ENST00000541979

370163,ENST00000370165

455263,ENST00000509690,ENST00000514944

379249,ENST00000379255,ENST00000379264,ENST00000379272,ENST00000413683,ENST00000431707,ENST

349807,ENST00000349807,ENST00000349807,ENST00000353004,ENST00000353004,ENST00000353004,ENST
349097,ENST00000349097,ENST00000349097,ENST00000395477,ENST00000395477,ENST00000395477,ENST
397412,ENST00000436138,ENST00000436138,ENST00000436138

360488,ENST00000377850,ENST00000377850,ENST00000377850
360488,ENST00000377850,ENST00000377850,ENST00000377850
525639,ENST00000527447,ENST00000527447,ENST00000527447
389131,ENST00000389131,ENST00000518083,ENST00000518083,ENST00000518083,ENST00000518083,ENST

294724,ENST00000294724,ENST00000361302,ENST00000361302,ENST00000361302,ENST00000361302,ENST
338347,ENST00000338347,ENST00000338347

379500,ENST00000533953,ENST00000533953,ENST00000533953
352853,ENST00000352853,ENST00000359524,ENST00000359524,ENST00000359524,ENST00000359524,ENST

296869,ENST00000296869,ENST00000296869,ENST00000296869,ENST00000296869,ENST00000296869,ENST
357674,ENST00000450955,ENST00000450955,ENST00000450955

323594,ENST00000510913,ENST00000510913,ENST00000510913
442594,ENST00000536534,ENST00000536534,ENST00000536534
549091,ENST00000552728,ENST00000552728,ENST00000552728

355725,ENST00000491737,ENST00000491737,ENST00000491737
370918,ENST00000370918,ENST00000370918,ENST00000541407,ENST00000541407,ENST00000541407,ENST

376020,ENST00000460112,ENST00000460112,ENST00000460112

522628,ENST00000543296,ENST00000543296,ENST00000543296

332647,ENST00000332647,ENST00000332647,ENST00000355813,ENST00000355813,ENST00000355813,ENST
508491,ENST00000543251,ENST00000543251,ENST00000543251

348639,ENST00000559658,ENST00000559658,ENST00000559658

269305,ENST00000269305,ENST00000269305,ENST00000359597,ENST00000359597,ENST00000359597,ENST

367483,ENST00000367483,ENST00000367483,ENST00000367483,ENST00000367483,ENST00000367485,ENST
372627,ENST00000372628,ENST00000372628,ENST00000372628

465038,ENST00000541979

370163,ENST00000370165
371367,ENST00000371372,ENST00000545473

455263,ENST00000509690,ENST00000514944

380553,ENST00000380562,ENST00000380576,ENST00000380584,ENST00000417091,ENST00000428787,ENST

381885,ENST00000396923,ENST00000420765

564546,ENST00000564595,ENST00000564688,ENST00000566897,ENST00000569545,ENST00000569798

379249,ENST00000379255,ENST00000379264,ENST00000379272,ENST00000413683,ENST00000431707,ENST

349807,ENST00000349807,ENST00000349807,ENST00000353004,ENST00000353004,ENST00000353004,ENST
397412,ENST00000436138,ENST00000436138,ENST00000436138

525639,ENST00000527447,ENST00000527447,ENST00000527447
252034,ENST00000252034,ENST00000252034,ENST00000252034,ENST00000252034,ENST00000252034,ENST
389131,ENST00000389131,ENST00000518083,ENST00000518083,ENST00000518083,ENST00000518083,ENST
423602,ENST00000423602,ENST00000423602,ENST00000433283,ENST00000433283,ENST00000433283,ENST

294724,ENST00000294724,ENST00000361302,ENST00000361302,ENST00000361302,ENST00000361302,ENST
564546,ENST00000564595,ENST00000564688,ENST00000565355,ENST00000566897,ENST00000569545,ENST

375122,ENST00000375127,ENST00000375127,ENST00000375127
352853,ENST00000352853,ENST00000359524,ENST00000359524,ENST00000359524,ENST00000359524,ENST

535041,ENST00000543818,ENST00000543818,ENST00000543818
296869,ENST00000296869,ENST00000296869,ENST00000296869,ENST00000296869,ENST00000296869,ENST

357674,ENST00000450955,ENST00000450955,ENST00000450955

323594,ENST00000510913,ENST00000510913,ENST00000510913

442594,ENST00000536534,ENST00000536534,ENST00000536534

549091,ENST00000552728,ENST00000552728,ENST00000552728

370621,ENST00000503581,ENST00000503581,ENST00000503581

359263,ENST00000359263,ENST00000359263,ENST00000359263,ENST00000359263,ENST00000371489,ENST

355725,ENST00000491737,ENST00000491737,ENST00000491737
488961,ENST00000553096,ENST00000553096,ENST00000553096
370918,ENST00000370918,ENST00000370918,ENST00000541407,ENST00000541407,ENST00000541407,ENST

334933,ENST00000334933,ENST00000334933,ENST00000348806,ENST00000348806,ENST00000348806,ENST
383806,ENST00000383806,ENST00000383806,ENST00000383807,ENST00000383807,ENST00000383807,ENST
376020,ENST00000460112,ENST00000460112,ENST00000460112
535481,ENST00000544910,ENST00000544910,ENST00000544910
522628,ENST00000543296,ENST00000543296,ENST00000543296
277549,ENST00000277549,ENST00000277549,ENST00000277551,ENST00000277551,ENST00000277551,ENST

375383,ENST00000375383,ENST00000375383,ENST00000375383,ENST00000375383,ENST00000375401,ENST

357248,ENST00000357248,ENST00000357248,ENST00000382194,ENST00000382194,ENST00000382194,ENST
269305,ENST00000269305,ENST00000269305,ENST00000359597,ENST00000359597,ENST00000359597,ENST

389175,ENST00000389175,ENST00000389175,ENST00000409270,ENST00000409270,ENST00000409270,ENST

555833,ENST00000556134

541205,ENST00000544327,ENST00000546215

261693,ENST00000261693,ENST00000261693,ENST00000339570,ENST00000339570,ENST00000339570,ENST
392990,ENST00000555626,ENST00000555626,ENST00000555626

441024,ENST00000441024,ENST00000441024,ENST00000567188,ENST00000567188,ENST00000567188,ENST
359357,ENST00000359357,ENST00000359357,ENST00000441566,ENST00000441566,ENST00000441566,ENST

261244,ENST00000261244,ENST00000354386,ENST00000354386,ENST00000354386,ENST00000354386,ENST

433979,ENST00000538779,ENST00000538779,ENST00000538779

247829,ENST00000247829,ENST00000247829,ENST00000393330,ENST00000393330,ENST00000393330,ENST

382871,ENST00000382871,ENST00000382871,ENST00000382874,ENST00000382874,ENST00000382874,ENST

407775,ENST00000407775,ENST00000407775,ENST00000517361,ENST00000517361,ENST00000517361,ENST
392650,ENST00000424778,ENST00000424778,ENST00000424778

522041,ENST00000523676

452989,ENST00000606214

551598

367573,ENST00000526775

357123,ENST00000357123,ENST00000357123,ENST00000357123,ENST00000357123,ENST00000546244,ENST
392142,ENST00000544041,ENST00000544041,ENST00000544041

435811,ENST00000454853,ENST00000454853,ENST00000454853
434343,ENST00000513376,ENST00000513376,ENST00000513376

343141,ENST00000343141,ENST00000356436,ENST00000356436,ENST00000356436,ENST00000356436,ENST

381567,ENST00000400841,ENST00000400841,ENST00000400841
432648,ENST00000446189,ENST00000446189,ENST00000446189

446702,ENST00000539053,ENST00000539053,ENST00000539053
329245,ENST00000329245,ENST00000519139,ENST00000519139,ENST00000519139,ENST00000519139,ENST

357570,ENST00000357570,ENST00000358338,ENST00000358338,ENST00000358338,ENST00000358338,ENST

545626,ENST00000546254,ENST00000546254,ENST00000546254
535842

574415,ENST00000575632

515826

394413,ENST00000394414,ENST00000453001,ENST00000504198,ENST00000508545

510071,ENST00000512052,ENST00000514667,ENST00000515170

450717,ENST00000541122,ENST00000589677,ENST00000590574
589123,ENST00000590282
603639,ENST00000604864

538974,ENST00000539331,ENST00000540783

527998,ENST00000529388,ENST00000531665,ENST00000533520,ENST00000534544

521891,ENST00000523809

455528

377885
417856,ENST00000421552,ENST00000461611

514678,ENST00000606977

591111

582857

438793,ENST00000455264

543262

452124,ENST00000460129

545844,ENST00000564288,ENST00000567887

584368
552432

486199
433949,ENST00000502686,ENST00000539382,ENST00000608263,ENST00000608425,ENST00000608440,ENST

545377

541717,ENST00000543751,ENST00000545008

603841

454569
435071,ENST00000443708,ENST00000455397,ENST00000458566

495488,ENST00000496965

548586,ENST00000549325,ENST00000549396,ENST00000550359,ENST00000550584,ENST00000552948

368301,ENST00000392353,ENST00000448611,ENST00000473598,ENST00000508500

382330,ENST00000392537,ENST00000411531,ENST00000414031,ENST00000424196,ENST00000427845,ENST

566059

530301,ENST00000530749,ENST00000544551

444341,ENST00000452260,ENST00000454909,ENST00000457005,ENST00000493952

531148,ENST00000533712

538883,ENST00000543994

553916

603808

430669,ENST00000437951

603841

541717,ENST00000543751,ENST00000545008

417856,ENST00000421552,ENST00000461611

452124,ENST00000460129

548586,ENST00000549325,ENST00000549396,ENST00000550359,ENST00000550584,ENST00000552948

450717,ENST00000541122,ENST00000589677,ENST00000590574

433949,ENST00000502686,ENST00000539382,ENST00000608263,ENST00000608425,ENST00000608440,ENST
515826
443708,ENST00000455397,ENST00000458566

539331,ENST00000540783

377885

486199
553916

394413,ENST00000394414,ENST00000453001,ENST00000504198,ENST00000508545

566059

552432

545844,ENST00000564288,ENST00000567887

438793,ENST00000455264
430669,ENST00000437951

513612,ENST00000514678,ENST00000606977

530301,ENST00000530749,ENST00000544551

368301,ENST00000392353,ENST00000448611,ENST00000473598,ENST00000508500

545377

531148,ENST00000533712

535842

434685,ENST00000603639,ENST00000604864

538883,ENST00000543994

527998,ENST00000529388,ENST00000531665,ENST00000533520,ENST00000534544

584368
600359

382330,ENST00000392537,ENST00000411531,ENST00000414031,ENST00000424196,ENST00000427845,ENST

591111

559411

589123,ENST00000590282

603808

509018,ENST00000510071,ENST00000512052,ENST00000515170

495488,ENST00000496965
521891,ENST00000523809

444341,ENST00000452260,ENST00000454909,ENST00000457005,ENST00000493952

455528

543262

449052,ENST00000538115,ENST00000542585,ENST00000546292

449052,ENST00000538115,ENST00000542585,ENST00000546292

439954

449052,ENST00000538115,ENST00000542585,ENST00000546292

449052,ENST00000538115,ENST00000542585,ENST00000546292

483968

449052,ENST00000538115,ENST00000542585,ENST00000546292

439954

483968

456556

449052,ENST00000538115,ENST00000542585,ENST00000546292

448089,ENST00000448691,ENST00000538248,ENST00000541601,ENST00000545230

539636

553300,ENST00000553753,ENST00000554383,ENST00000554455,ENST00000554539,ENST00000554969,ENST

448691,ENST00000538248,ENST00000541601,ENST00000545230,ENST00000425945

539636

439954

439954

553300,ENST00000553753,ENST00000554383,ENST00000554455,ENST00000554539,ENST00000554969,ENST

593214

477086,ENST00000477756,ENST00000478156,ENST00000479406,ENST00000480843

449052,ENST00000538115,ENST00000542585,ENST00000546292

449052,ENST00000538115,ENST00000542585,ENST00000546292

477086,ENST00000477756,ENST00000478156,ENST00000479406,ENST00000480843
477086,ENST00000477756,ENST00000478156,ENST00000479406,ENST00000480843

449052,ENST00000538115,ENST00000542585,ENST00000546292

449052,ENST00000538115,ENST00000542585,ENST00000546292

593214

477086,ENST00000477756,ENST00000478156,ENST00000479406,ENST00000480843,ENST00000462323,ENST

449052,ENST00000538115,ENST00000542585,ENST00000546292

477086,ENST00000477756,ENST00000478156,ENST00000479406,ENST00000480843
449052,ENST00000538115,ENST00000542585,ENST00000546292,ENST00000348028,ENST00000349350,ENST

477086,ENST00000477756,ENST00000478156,ENST00000479406,ENST00000480843

449052,ENST00000538115,ENST00000542585,ENST00000546292

449052,ENST00000538115,ENST00000542585,ENST00000546292

498644

498644

589119,ENST00000606248

453976,ENST00000491071,ENST00000517827,ENST00000517960,ENST00000518273,ENST00000520567,ENST

589119,ENST00000606248

453976,ENST00000491071,ENST00000517827,ENST00000517960,ENST00000518273,ENST00000520567,ENST

449052,ENST00000538115,ENST00000542585,ENST00000546292

449052,ENST00000538115,ENST00000542585,ENST00000546292

555723

598381

555723

449052,ENST00000538115,ENST00000542585,ENST00000546292

377386,ENST00000398263,ENST00000409015,ENST00000409232,ENST00000444342

449052,ENST00000538115,ENST00000542585,ENST00000546292

584074

576538

507280,ENST00000509064,ENST00000509452,ENST00000510651,ENST00000512470,ENST00000513723,ENST
560658,ENST00000560801

587303,ENST00000588012,ENST00000592099

524845,ENST00000524904,ENST00000527403,ENST00000529710,ENST00000534190,ENST00000534534

584074

576538

507280,ENST00000509064,ENST00000509452,ENST00000510651,ENST00000512470,ENST00000513723,ENST

587303,ENST00000588012,ENST00000592099

560658,ENST00000560801

524845,ENST00000524904,ENST00000527403,ENST00000529710,ENST00000534190,ENST00000534534

595238,ENST00000600767
540012

584074
530740

582809

507280,ENST00000509064,ENST00000509452,ENST00000510651,ENST00000512470,ENST00000513723,ENST
560658,ENST00000560801

449052,ENST00000538115,ENST00000542585,ENST00000546292
415348,ENST00000430656,ENST00000456320,ENST00000522886

524845,ENST00000524904,ENST00000527403,ENST00000529710,ENST00000534190,ENST00000534534

540012
584074

595238,ENST00000600767

415348,ENST00000430656,ENST00000456320,ENST00000522886

507280,ENST00000509064,ENST00000509452,ENST00000510651,ENST00000512470,ENST00000513723,ENST
582809

560658,ENST00000560801

524845,ENST00000524904,ENST00000527403,ENST00000529710,ENST00000534190,ENST00000534534

449052,ENST00000538115,ENST00000542585,ENST00000546292

388743,ENST00000419131
530417,ENST00000534126,ENST00000534528

452003

402731,ENST00000404276,ENST00000405598,ENST00000416671,ENST00000417588,ENST00000433028,ENST

475125

388743,ENST00000419131

534126

475125

452003

433368

443539,ENST00000446190

429972,ENST00000490882,ENST00000493452

552788
457156

397043

397043

443539,ENST00000446190
429972,ENST00000490882,ENST00000493452

433368

552788

484451,ENST00000487473,ENST00000493611

478922,ENST00000481953,ENST00000498699

484451,ENST00000487473,ENST00000493611

478922,ENST00000481953,ENST00000498699

428097,ENST00000428648,ENST00000433959,ENST00000453256,ENST00000454227,ENST00000455023,ENST

540079,ENST00000541240,ENST00000543930,ENST00000562503,ENST00000562903,ENST00000564136,ENST

541205,ENST00000544327,ENST00000546215

593660,ENST00000593821,ENST00000601256,ENST00000601951,ENST00000602261

581031,ENST00000581094,ENST00000581148,ENST00000583994

487505,ENST00000490574

433722,ENST00000537108

433722,ENST00000537108

428097,ENST00000428648,ENST00000433959,ENST00000453256,ENST00000454227,ENST00000455023,ENST

487505,ENST00000490574

593660,ENST00000593821,ENST00000601256,ENST00000601951,ENST00000602261

541205,ENST00000544327,ENST00000546215
433722,ENST00000537108,ENST00000251071,ENST00000298892,ENST00000308433,ENST00000395805,ENST

442920

580392,ENST00000581031,ENST00000581094,ENST00000581148,ENST00000582602,ENST00000583994

540079,ENST00000541240,ENST00000543930,ENST00000562503,ENST00000562903,ENST00000564136,ENST

428097,ENST00000428648,ENST00000433959,ENST00000453256,ENST00000454227,ENST00000455023,ENST

521843

398209,ENST00000426930

487505,ENST00000490574

556766,ENST00000557376

601733

447267,ENST00000520168,ENST00000523483

422297,ENST00000423785,ENST00000425237,ENST00000444973,ENST00000447379,ENST00000539416,ENST

428097,ENST00000428648,ENST00000433959,ENST00000453256,ENST00000454227,ENST00000455023,ENST

487505,ENST00000490574
521843

438182

422297,ENST00000423785,ENST00000425237,ENST00000444973,ENST00000447379,ENST00000539416,ENST

447267,ENST00000520168,ENST00000523483
556766,ENST00000557376
598654,ENST00000601733

449052,ENST00000538115,ENST00000542585,ENST00000546213,ENST00000546292
521676,ENST00000522420
403642,ENST00000404276,ENST00000405598,ENST00000544772

521676,ENST00000522420
449052,ENST00000538115,ENST00000542585,ENST00000546213,ENST00000546292
403642,ENST00000404276,ENST00000405598,ENST00000544772

374650,ENST00000438843,ENST00000448480

407969,ENST00000413704

374650,ENST00000438843,ENST00000448480
539875

409547,ENST00000423659,ENST00000440945,ENST00000452341

403642,ENST00000404276,ENST00000405598,ENST00000544772

409547,ENST00000423659,ENST00000440945,ENST00000452341

403642,ENST00000404276,ENST00000405598,ENST00000544772

539875

442782

464224,ENST00000483239,ENST00000494714,ENST00000525965,ENST00000529221,ENST00000529850,ENST

442782

464224,ENST00000483239,ENST00000494714,ENST00000525965,ENST00000529221,ENST00000529850,ENST

565494,ENST00000567435

567435

361676,ENST00000397789,ENST00000403435,ENST00000403999

600377,ENST00000601879

512970,ENST00000514566
512970,ENST00000514566
600377,ENST00000601879

361676,ENST00000397789,ENST00000403435,ENST00000403999

443581,ENST00000449557,ENST00000450102,ENST00000451551,ENST00000455624

457266
490843,ENST00000545495

435716,ENST00000453485,ENST00000543084

555528,ENST00000555926

590602,ENST00000591436

555528,ENST00000555926

443581,ENST00000449557,ENST00000450102,ENST00000451551,ENST00000455624
358464,ENST00000439954

457266

490843,ENST00000545495

456154
453485,ENST00000543084

590602,ENST00000591436

592725

458406,ENST00000558589,ENST00000558667,ENST00000559523,ENST00000559664,ENST00000560164,ENST

425146,ENST00000446737,ENST00000518291,ENST00000519681
571451,ENST00000572272,ENST00000577119

571451,ENST00000572272,ENST00000577119

425146,ENST00000446737,ENST00000518291,ENST00000519681

458406,ENST00000558589,ENST00000558667,ENST00000559523,ENST00000559664,ENST00000560164,ENST

449052,ENST00000538115,ENST00000542585,ENST00000546292
403642,ENST00000404276,ENST00000405598,ENST00000544772

439673,ENST00000568454

540320

449052,ENST00000538115,ENST00000542585,ENST00000546292

403642,ENST00000404276,ENST00000405598,ENST00000544772

540320

439673,ENST00000568454

361676,ENST00000397789,ENST00000403435,ENST00000403999

361676,ENST00000397789,ENST00000403435,ENST00000403999

477086,ENST00000477756,ENST00000478156,ENST00000479406,ENST00000480843

589042,ENST00000591111

403813,ENST00000414165

589042,ENST00000591111

403813,ENST00000414165

477086,ENST00000477756,ENST00000478156,ENST00000479406,ENST00000480843

533202,ENST00000533786

589042,ENST00000591111

403642,ENST00000404276,ENST00000405598,ENST00000544772
572158,ENST00000572405,ENST00000575333,ENST00000575882,ENST00000575909,ENST00000576932

402384,ENST00000406470,ENST00000407906,ENST00000422063

599577,ENST00000600833

572158,ENST00000572405,ENST00000575333,ENST00000575882,ENST00000575909,ENST00000576932

403642,ENST00000404276,ENST00000405598,ENST00000544772

533786

589042,ENST00000591111

402384,ENST00000406470,ENST00000407906,ENST00000422063

599577,ENST00000600833

469430,ENST00000483288,ENST00000485178
544867,ENST00000545558
449052,ENST00000538115,ENST00000542585,ENST00000546292

440709,ENST00000457250

532297
477310,ENST00000497706,ENST00000556679
477086,ENST00000477756,ENST00000478156,ENST00000479406,ENST00000480843

469430,ENST00000483288,ENST00000485178

456570,ENST00000477310,ENST00000556679

440709,ENST00000457250

544867,ENST00000545558

477086,ENST00000477756,ENST00000478156,ENST00000479406,ENST00000480843

532297

449052,ENST00000538115,ENST00000542585,ENST00000546292

544867,ENST00000545558

445505

477086,ENST00000477756,ENST00000478156,ENST00000479406,ENST00000480843

514453,ENST00000514546,ENST00000515492

445505

544867,ENST00000545558

477086,ENST00000477756,ENST00000478156,ENST00000479406,ENST00000480843

371141,ENST00000464640,ENST00000467096,ENST00000530122

494877

551379

425615,ENST00000500320

494877
494877

425615,ENST00000500320

551379

589378

589378

521661,ENST00000523517
598205,ENST00000599920,ENST00000601313

442759,ENST00000446759,ENST00000466548,ENST00000474663,ENST00000488699,ENST00000496045

397097,ENST00000397126,ENST00000397126,ENST00000418063,ENST00000418100,ENST00000594947,ENST

458225

404092

458225

521661,ENST00000523517

598205,ENST00000599920,ENST00000601313

442759,ENST00000446759,ENST00000466548,ENST00000474663,ENST00000488699,ENST00000496045

521661,ENST00000523517
493429

455756

369354,ENST00000369356,ENST00000369359,ENST00000526445,ENST00000528129,ENST00000530740,ENST

442759,ENST00000446759,ENST00000466548,ENST00000474663,ENST00000488699,ENST00000496045

594419,ENST00000601693

458225

455756

369354,ENST00000369356,ENST00000369359,ENST00000526445,ENST00000528129,ENST00000530740,ENST

458225

521661,ENST00000523517

493429

442759,ENST00000446759,ENST00000466548,ENST00000474663,ENST00000488699,ENST00000496045

465261,ENST00000526202

537460,ENST00000538980,ENST00000539091,ENST00000540209,ENST00000542145,ENST00000543393,ENST
591111

453196,ENST00000476352,ENST00000530997

549027,ENST00000551601

458225

447038,ENST00000465261,ENST00000524651,ENST00000525107,ENST00000526202

458225

537460,ENST00000538980,ENST00000539091,ENST00000540209,ENST00000542145,ENST00000543393,ENST

591111

453196,ENST00000476352,ENST00000530997

549027,ENST00000551601

534214

521661,ENST00000523517

442759,ENST00000446759,ENST00000466548,ENST00000474663,ENST00000488699,ENST00000496045

519895,ENST00000520602

458225

534214

458225

521661,ENST00000523517

519895,ENST00000520602

442759,ENST00000446759,ENST00000466548,ENST00000474663,ENST00000488699,ENST00000496045

397097,ENST00000397097,ENST00000511833,ENST00000594012,ENST00000595461,ENST00000598381

399602,ENST00000429541,ENST00000526601,ENST00000594012,ENST00000595461,ENST00000598381,ENST

357491,ENST00000359735,ENST00000359973,ENST00000389534,ENST00000397136,ENST00000418063,ENST

396165,ENST00000553352,ENST00000553728,ENST00000554392,ENST00000555716,ENST00000603904

477086,ENST00000477756,ENST00000478156,ENST00000479406,ENST00000480843

520578,ENST00000522367,ENST00000522761,ENST00000523519

396165,ENST00000553352,ENST00000553728,ENST00000554392,ENST00000555716,ENST00000603904

406620,ENST00000566587

397082,ENST00000397126,ENST00000419098,ENST00000427401,ENST00000594012,ENST00000594012,ENST

598381

396165,ENST00000553352,ENST00000554392,ENST00000555716,ENST00000553728,ENST00000603904,ENST

477086,ENST00000477756,ENST00000478156,ENST00000479406,ENST00000480843

526601

520578,ENST00000522367,ENST00000522761,ENST00000523519

406620,ENST00000566587

570818,ENST00000575483

594012,ENST00000595461,ENST00000598381

600435

419098,ENST00000420713,ENST00000594012,ENST00000595461,ENST00000598381

556177

589656,ENST00000591032,ENST00000591804,ENST00000593052

477086,ENST00000477756,ENST00000478156,ENST00000479406,ENST00000480843

477086,ENST00000477756,ENST00000478156,ENST00000479406,ENST00000480843

127709,NM_001127710,NM_001127710,NM_001303232,NM_001303232,NM_005807,NM_005807

001142529,NM_001142530,NM_030639

001269041,NM_001269042,NM_001269043,NM_001269044,NM_001269045,NM_001269046,NM_0012690[illegible]

.001318795,NM_001318796,NM_003264
.282390

001014797,NM_001161352,NM_001161353,NM_001271518,NM_001271519,NM_001322829,NM_0013228[illegible]

014393

NM_001322893,NM_001322894,NM_001322895,NM_001322896,NM_001322897,NM_001322898,NM_00132

[illegible]M_001322893,NM_001322894,NM_001322895,NM_001322896,NM_001322897,NM_001322898,NM_0013[illegible]

.321904,NM_020532,NM_207521

NM_001322893,NM_001322894,NM_001322895,NM_001322896,NM_001322897,NM_001322898,NM_0013[illegible]

NM_001322893,NM_001322894,NM_001322895,NM_001322896,NM_001322897,NM_001322898,NM_0013[illegible]

NM_001322893,NM_001322894,NM_001322895,NM_001322896,NM_001322897,NM_001322898,NM_00132
NM_001322893,NM_001322894,NM_001322895,NM_001322896,NM_001322897,NM_001322898,NM_00132

.318832

_001300744,NM_001300745,NM_001318119,NM_001318120,NM_016211

00000357462,ENST00000358087,ENST00000358087,ENST00000358087,ENST00000358087,ENST0000035808

00000349350,ENST00000349350,ENST00000349350,ENST00000349350,ENST00000349350,ENST0000034935
00000367483,ENST00000367483,ENST00000367483,ENST00000367484,ENST00000367484,ENST0000036748

00000518825,ENST00000518825,ENST00000518825,ENST00000518825,ENST00000518910,ENST0000051891

00000361163,ENST00000409914,ENST00000409914,ENST00000409914,ENST00000409914
00000367485,ENST00000367485,ENST00000367485,ENST00000367485,ENST00000367486,ENST0000036748
00000367483,ENST00000367484,ENST00000367484,ENST00000367484,ENST00000367484,ENST0000036748

00000402615,ENST00000402615,ENST00000402615,ENST00000407796,ENST00000407796,ENST0000040779

00000357462,ENST00000357462,ENST00000357462,ENST00000357462,ENST00000357462,ENST0000035746

00000360967,ENST00000360967,ENST00000360967,ENST00000360967,ENST00000360967,ENST0000036116

00000349350,ENST00000349350,ENST00000349350,ENST00000349350,ENST00000349350,ENST0000034935

00000367483,ENST00000367483,ENST00000367483,ENST00000367484,ENST00000367484,ENST0000036748

00000518825,ENST00000518825,ENST00000518825,ENST00000518825,ENST00000518910,ENST0000051891

00000361163,ENST00000409914,ENST00000409914,ENST00000409914,ENST00000409914

00000360967,ENST00000360967,ENST00000360967,ENST00000360967,ENST00000360967,ENST0000036116

00000367485,ENST00000367485,ENST00000367485,ENST00000367485,ENST00000367486,ENST0000036748
00000367483,ENST00000367484,ENST00000367484,ENST00000367484,ENST00000367484,ENST0000036748

'00000368284,ENST00000368284,ENST00000368284,ENST00000368284,ENST00000368285,ENST0000036828

'00000360232,ENST00000360232,ENST00000360232,ENST00000360232,ENST00000360232,ENST0000036023

'00000454075,ENST00000454075,ENST00000454075,ENST00000537215,ENST00000537215,ENST0000053721

'00000359221,ENST00000359221,ENST00000359221,ENST00000359221,ENST00000359221,ENST0000035922

'00000341594,ENST00000341594,ENST00000341594,ENST00000341594,ENST00000341594,ENST0000035682

'00000379933,ENST00000379933,ENST00000379933,ENST00000379933,ENST00000379938,ENST0000037993
'00000369339,ENST00000490598,ENST00000490598,ENST00000490598,ENST00000490598,ENST0000060517

'00000375400,ENST00000375400,ENST00000375400,ENST00000375400,ENST00000535393,ENST0000053539

'00000368284,ENST00000368284,ENST00000368284,ENST00000368284,ENST00000368285,ENST0000036828

'00000360232,ENST00000360232,ENST00000360232,ENST00000360232,ENST00000360232,ENST0000036023

'00000454075,ENST00000454075,ENST00000454075,ENST00000537215,ENST00000537215,ENST0000053721

'00000373631,ENST00000460253,ENST00000460253,ENST00000460253,ENST00000460253

'00000342992,ENST00000359218,ENST00000359218,ENST00000359218,ENST00000359218,ENST0000035921

'00000377400,ENST00000382447,ENST00000382447,ENST00000382447,ENST00000382447

'00000359221,ENST00000359221,ENST00000359221,ENST00000359221,ENST00000359221,ENST0000035922

'00000395435,ENST00000395435,ENST00000395435,ENST00000395435,ENST00000395435,ENST0000039543

'00000357517,ENST00000394536,ENST00000394536,ENST00000394536,ENST00000394536,ENST0000039453
'00000375400,ENST00000375400,ENST00000375400,ENST00000375400,ENST00000535393,ENST0000053539

'00000360232,ENST00000360232,ENST00000360232,ENST00000360232,ENST00000360232,ENST0000036023

'00000396410,ENST00000396410,ENST00000396410,ENST00000396410,ENST00000396410,ENST0000039641
'00000454075,ENST00000454075,ENST00000454075,ENST00000537215,ENST00000537215,ENST0000053721

'00000359221,ENST00000359221,ENST00000359221,ENST00000359221,ENST00000359221,ENST0000035922

'00000375400,ENST00000375400,ENST00000375400,ENST00000375400,ENST00000535393,ENST0000053539

'00000349350,ENST00000349350,ENST00000349350,ENST00000349350,ENST00000349350,ENST0000034935
'00000367483,ENST00000367484,ENST00000367484,ENST00000367484,ENST00000367484,ENST0000036748

'00000375274,ENST00000375290,ENST00000375290,ENST00000375290,ENST00000375290,ENST0000037529

'00000348383,ENST00000348383,ENST00000348383,ENST00000348383,ENST00000348383,ENST0000035727

'00000543479,ENST00000544770

'00000431971,ENST00000441865,ENST00000448295,ENST00000457310

00000397082,ENST00000397082,ENST00000397097,ENST00000397121,ENST00000402377,ENST0000042362

00000353004,ENST00000354078,ENST00000354078,ENST00000354078,ENST00000354078
00000421160,ENST00000421160,ENST00000421160,ENST00000421160,ENST00000447444,ENST0000044744

00000553484,ENST00000555664,ENST00000555664,ENST00000555664,ENST00000555664
00000409658,ENST00000436787,ENST00000436787,ENST00000436787,ENST00000436787

00000518083,ENST00000518083,ENST00000518083,ENST00000519243,ENST00000519243,ENST0000051924

00000376072,ENST00000376073,ENST00000376073,ENST00000376073,ENST00000376073

00000361302,ENST00000361302,ENST00000361302,ENST00000361522,ENST00000361522,ENST0000036152

00000480623,ENST00000538167,ENST00000538167,ENST00000538167,ENST00000538167

00000595461,ENST00000598381,ENST00000598381,ENST00000598381,ENST00000598381
00000546047,ENST00000546047,ENST00000546047,ENST00000546047,ENST00000571215,ENST0000057121

00000596534,ENST00000601023,ENST00000601023,ENST00000601023,ENST00000601023

'00000296869,ENST00000296869,ENST00000357096,ENST00000357096,ENST00000357096,ENST0000035709

'00000277551,ENST00000371355,ENST00000371355,ENST00000371355,ENST00000371355,ENST0000037135

'00000369339,ENST00000490598,ENST00000490598,ENST00000490598,ENST00000490598

'00000397082,ENST00000594012,ENST00000594012,ENST00000594012,ENST00000594012,ENST0000059546

'00000395270,ENST00000395270,ENST00000395270,ENST00000395270,ENST00000434423,ENST0000043442

'00000511604,ENST00000511604,ENST00000511604,ENST00000511604,ENST00000512055,ENST0000051205

00000397136,ENST00000397136,ENST00000397136,ENST00000397136,ENST00000437743,ENST0000043774

00000575087,ENST00000575087,ENST00000575087,ENST00000575087,ENST00000575842,ENST0000057584

00000348069,ENST00000366496,ENST00000366496,ENST00000366496,ENST00000366496,ENST0000036649

00000394067,ENST00000394067,ENST00000394067,ENST00000394067,ENST00000417856,ENST0000041785
00000414083,ENST00000414083,ENST00000414083,ENST00000414083,ENST00000446403,ENST0000044640

00000596534,ENST00000601023,ENST00000601023,ENST00000601023,ENST00000601023
00000399232,ENST00000399232,ENST00000399232,ENST00000399232,ENST00000426342,ENST0000042634

00000396774,ENST00000520188,ENST00000520188,ENST00000520188,ENST00000520188
00000535545,ENST00000542951,ENST00000542951,ENST00000542951,ENST00000542951
00000393785,ENST00000393785,ENST00000393785,ENST00000462705,ENST00000462705,ENST0000046270

00000276344,ENST00000276344,ENST00000276344,ENST00000276344,ENST00000360243,ENST0000036024
00000398548,ENST00000398548,ENST00000398548,ENST00000422911,ENST00000422911,ENST0000042291

00000378615,ENST00000378615,ENST00000378615,ENST00000378618,ENST00000378618,ENST0000037861

00000390003,ENST00000390003,ENST00000390003,ENST00000402934,ENST00000402934,ENST0000040293

00000596534,ENST00000601023,ENST00000601023,ENST00000601023,ENST00000601023

00000429348,ENST00000429348,ENST00000429348,ENST00000432264,ENST00000432264,ENST0000043226
00000415165,ENST00000415165,ENST00000415165,ENST00000415165,ENST00000441319,ENST0000044131

00000585941,ENST00000592690,ENST00000592690,ENST00000592690,ENST00000592690

00000359597,ENST00000359597,ENST00000359597,ENST00000359597,ENST00000359597,ENST0000041346

00000354228,ENST00000354228,ENST00000354228,ENST00000354228,ENST00000354228,ENST0000035441

00000553484,ENST00000555664,ENST00000555664,ENST00000555664,ENST00000555664

00000348028,ENST00000348028,ENST00000348028,ENST00000348028,ENST00000348028,ENST0000034802

00000349350,ENST00000349350,ENST00000349350,ENST00000349350,ENST00000349350,ENST0000034935

'00000543479,ENST00000544770

00000353004,ENST00000354078,ENST00000354078,ENST00000354078,ENST00000354078
00000395477,ENST00000427277,ENST00000427277,ENST00000427277,ENST00000427277

00000506583,ENST00000513129,ENST00000513129,ENST00000513129,ENST00000513129

00000518083,ENST00000518083,ENST00000518083,ENST00000519243,ENST00000519243,ENST0000051924

00000361302,ENST00000361302,ENST00000361302,ENST00000361522,ENST00000361522,ENST0000036152

00000359524,ENST00000359524,ENST00000359524,ENST00000371432,ENST00000371432,ENST0000037143

00000296869,ENST00000296869,ENST00000357096,ENST00000357096,ENST00000357096,ENST0000035709

00000528372,ENST00000544249,ENST00000544249,ENST00000544249,ENST00000544249

00000541407,ENST00000542638,ENST00000542638,ENST00000542638,ENST00000542638

'00000359597,ENST00000359597,ENST00000359597,ENST00000359597,ENST00000359597,ENST0000041346

'00000590550,ENST00000592012,ENST00000592012,ENST00000592012,ENST00000592012

'00000543479,ENST00000544770

'00000353004,ENST00000354078,ENST00000354078,ENST00000354078,ENST00000354078
'00000395477,ENST00000427277,ENST00000427277,ENST00000427277,ENST00000427277

'00000518083,ENST00000518083,ENST00000518083,ENST00000519243,ENST00000519243,ENST0000051924

00000361302,ENST00000361302,ENST00000361302,ENST00000361522,ENST00000361522,ENST0000036152

00000359524,ENST00000359524,ENST00000359524,ENST00000371432,ENST00000371432,ENST0000037143

00000296869,ENST00000296869,ENST00000357096,ENST00000357096,ENST00000357096,ENST0000035709

00000541407,ENST00000542638,ENST00000542638,ENST00000542638,ENST00000542638

00000355813,ENST00000538690,ENST00000538690,ENST00000538690,ENST00000538690

00000359597,ENST00000359597,ENST00000359597,ENST00000359597,ENST00000359597,ENST0000041346

00000367485,ENST00000367485,ENST00000367485,ENST00000367485,ENST00000367486,ENST0000036748

T00000429192,ENST00000438880,ENST00000438906,ENST00000442310,ENST00000445912

T00000543479,ENST00000544770

'00000353004,ENST00000354078,ENST00000354078,ENST00000354078,ENST00000354078

'00000252034,ENST00000252034,ENST00000252034,ENST00000252034,ENST00000252034,ENST0000032039
'00000518083,ENST00000518083,ENST00000518083,ENST00000519243,ENST00000519243,ENST0000051924
'00000433283,ENST00000441750,ENST00000441750,ENST00000441750,ENST00000441750

'00000361302,ENST00000361302,ENST00000361302,ENST00000361522,ENST00000361522,ENST0000036152
'00000569798

'00000359524,ENST00000359524,ENST00000359524,ENST00000371432,ENST00000371432,ENST0000037143

'00000296869,ENST00000296869,ENST00000357096,ENST00000357096,ENST00000357096,ENST0000035709

'00000371489,ENST00000371489,ENST00000371489,ENST00000371489,ENST00000371501,ENST0000037150

'00000541407,ENST00000542638,ENST00000542638,ENST00000542638,ENST00000542638

'00000348806,ENST00000348806,ENST00000348806,ENST00000348806,ENST00000348806,ENST0000036013
'00000383807,ENST00000419585,ENST00000419585,ENST00000419585,ENST00000419585

'00000277551,ENST00000277551,ENST00000277551,ENST00000277551,ENST00000277551,ENST0000037135

'00000375401,ENST00000375401,ENST00000375401,ENST00000375401,ENST00000404049,ENST0000040404

'00000382194,ENST00000382203,ENST00000382203,ENST00000382203,ENST00000382203
'00000359597,ENST00000359597,ENST00000359597,ENST00000359597,ENST00000359597,ENST0000041346

'00000409270,ENST00000448409,ENST00000448409,ENST00000448409,ENST00000448409

'00000339570,ENST00000339570,ENST00000339570,ENST00000339570,ENST00000339570,ENST0000037678

'00000567188,ENST00000569240,ENST00000569240,ENST00000569240,ENST00000569240
'00000441566,ENST00000449981,ENST00000449981,ENST00000449981,ENST00000449981

'00000354386,ENST00000354386,ENST00000354386,ENST00000423743,ENST00000423743,ENST0000042374

'00000393330,ENST00000393330,ENST00000393330,ENST00000393330,ENST00000393330,ENST0000054656

'00000382874,ENST00000382881,ENST00000382881,ENST00000382881,ENST00000382881

'00000517361,ENST00000520492,ENST00000520492,ENST00000520492,ENST00000520492

'00000546244,ENST00000546244,ENST00000546244,ENST00000546244,ENST00000550165,ENST0000055016

'00000356436,ENST00000356436,ENST00000356436,ENST00000379300,ENST00000379300,ENST0000037930

'00000519139,ENST00000519139,ENST00000519139,ENST00000519148,ENST00000519148,ENST0000051914

'00000358338,ENST00000358338,ENST00000358338,ENST00000360108,ENST00000360108,ENST0000036010

T00000608903,ENST00000609232

'00000434061,ENST00000435046,ENST00000441154

'00000608903,ENST00000609232

'00000434061,ENST00000435046,ENST00000441154

'00000555309,ENST00000555883,ENST00000555914,ENST00000556628,ENST00000556897,ENST0000055715

'00000555309,ENST00000555883,ENST00000555914,ENST00000556628,ENST00000556897,ENST0000055715

'00000463781,ENST00000466475,ENST00000470451,ENST00000475231,ENST00000477086,ENST0000047775

'00000421100,ENST00000427578,ENST00000443370,ENST00000449052,ENST00000538115,ENST0000054258

'00000521978,ENST00000522291,ENST00000523963

'00000521978,ENST00000522291,ENST00000523963

'00000514333,ENST00000514677,ENST00000514891

'00000514333,ENST00000514677,ENST00000514891

00000514333,ENST00000514677,ENST00000514891

00000514333,ENST00000514677,ENST00000514891

00000433728,ENST00000439200,ENST00000447421,ENST00000448511

'00000545390

'00000565481

'00000545390

'00000417045,ENST00000433722,ENST00000537108

'00000565481

'00000545390

00000540326,ENST00000545174,ENST00000546191

00000545390

00000540326,ENST00000545174,ENST00000546191

'00000531164

'00000531164

[illegible]00000560332,ENST00000560611,ENST00000561080,ENST00000561120

'00000560332,ENST00000560611,ENST00000561080,ENST00000561120

'00000601693

'00000530822,ENST00000531369,ENST00000534536

'00000530822,ENST00000531369,ENST00000534536

'00000543697

'00000543697

T00000601440

T00000426391,ENST00000437743,ENST00000440155,ENST00000456783,ENST00000589717,ENST0000059429

'00000594012,ENST00000595461,ENST00000595461,ENST00000595461,ENST00000598381,ENST0000059838

'00000280083,ENST00000341502,ENST00000341749,ENST00000348007,ENST00000396158,ENST0000039616

47,NM_001269048,NM_001269051,NM_001269052,NM_080618

30,NM_001322832,NM_001322835,NM_001322836,NM_001322837,NM_001322838,NM_002247

22899,NM_001322900,NM_002313,NM_006720,NM_001003407,NM_001003408,NM_001322882,NM_0013[illegible]

22899,NM_001322900,NM_002313,NM_006720

22899,NM_001322900,NM_002313,NM_006720,NM_001003407,NM_001003408,NM_001322882,NM_00132

22899,NM_001322900,NM_002313,NM_006720

22899,NM_001322900,NM_002313,NM_006720,NM_001003407,NM_001003408,NM_001322882,NM_00132
22899,NM_001322900,NM_002313,NM_006720

7,ENST00000358087,ENST00000360967,ENST00000360967,ENST00000360967,ENST00000360967,ENST00000

0,ENST00000349350,ENST00000421100,ENST00000421100,ENST00000421100,ENST00000421100,ENST00000
4,ENST00000367484,ENST00000367484,ENST00000367484,ENST00000367484,ENST00000367485,ENST00000

0,ENST00000518910,ENST00000518910,ENST00000518910,ENST00000520158,ENST00000520158,ENST00000

6,ENST00000367486,ENST00000367486,ENST00000367486,ENST00000445192,ENST00000445192,ENST00000
4,ENST00000367484,ENST00000367485,ENST00000367485,ENST00000367485,ENST00000367485,ENST00000

6,ENST00000407796,ENST00000407796,ENST00000407796,ENST00000407796,ENST00000554581,ENST00000

2,ENST00000357462,ENST00000358087,ENST00000358087,ENST00000358087,ENST00000358087,ENST00000
3,ENST00000361163,ENST00000361163,ENST00000361163,ENST00000361163,ENST00000361163,ENST00000

0,ENST00000349350,ENST00000421100,ENST00000421100,ENST00000421100,ENST00000421100,ENST00000
4,ENST00000367484,ENST00000367484,ENST00000367484,ENST00000367484,ENST00000367485,ENST00000

0,ENST00000518910,ENST00000518910,ENST00000518910,ENST00000520158,ENST00000520158,ENST00000

3,ENST00000361163,ENST00000361163,ENST00000361163,ENST00000361163,ENST00000361163,ENST00000

6,ENST00000367486,ENST00000367486,ENST00000367486,ENST00000445192,ENST00000445192,ENST00000
4,ENST00000367484,ENST00000367485,ENST00000367485,ENST00000367485,ENST00000367485,ENST00000

5,ENST00000368285,ENST00000368285,ENST00000368285,ENST00000368286,ENST00000368286,ENST00000

2,ENST00000360232,ENST00000360232,ENST00000360232,ENST00000390668,ENST00000390668,ENST00000

5,ENST00000537215,ENST00000537215,ENST00000537215,ENST00000537215,ENST00000538913,ENST00000

1,ENST00000359221,ENST00000359221,ENST00000359221,ENST00000361569,ENST00000361569,ENST00000

0,ENST00000356820,ENST00000356820,ENST00000356820,ENST00000356820,ENST00000356820,ENST00000

8,ENST00000379938,ENST00000379938,ENST00000379938,ENST00000483150,ENST00000483150,ENST00000
6,ENST00000605176,ENST00000605176,ENST00000605176

3,ENST00000535393,ENST00000535393,ENST00000535393,ENST00000538146,ENST00000538146,ENST00000

5,ENST00000368285,ENST00000368285,ENST00000368285,ENST00000368286,ENST00000368286,ENST00000C

2,ENST00000360232,ENST00000360232,ENST00000360232,ENST00000390668,ENST00000390668,ENST00000C

5,ENST00000537215,ENST00000537215,ENST00000537215,ENST00000537215,ENST00000538913,ENST00000C

8,ENST00000359218,ENST00000460472,ENST00000460472,ENST00000460472,ENST00000460472,ENST00000C

1,ENST00000359221,ENST00000359221,ENST00000359221,ENST00000361569,ENST00000361569,ENST00000

6,ENST00000395436,ENST00000395436,ENST00000395436,ENST00000395436,ENST00000395436,ENST00000

6,ENST00000394536,ENST00000456271,ENST00000456271,ENST00000456271,ENST00000456271,ENST00000
3,ENST00000535393,ENST00000535393,ENST00000535393,ENST00000538146,ENST00000538146,ENST00000

2,ENST00000360232,ENST00000360232,ENST00000360232,ENST00000390668,ENST00000390668,ENST00000

0,ENST00000396410,ENST00000447912,ENST00000447912,ENST00000447912,ENST00000447912,ENST00000
5,ENST00000537215,ENST00000537215,ENST00000537215,ENST00000537215,ENST00000538913,ENST00000

1,ENST00000359221,ENST00000359221,ENST00000359221,ENST00000361569,ENST00000361569,ENST00000

3,ENST00000535393,ENST00000535393,ENST00000535393,ENST00000538146,ENST00000538146,ENST00000

0,ENST00000349350,ENST00000421100,ENST00000421100,ENST00000421100,ENST00000421100,ENST00000
4,ENST00000367484,ENST00000367485,ENST00000367485,ENST00000367485,ENST00000367485,ENST00000

0,ENST00000375290,ENST00000430669,ENST00000430669,ENST00000430669,ENST00000430669,ENST00000

2,ENST00000357272,ENST00000357272,ENST00000357272,ENST00000357272,ENST00000357272,ENST00000

7,ENST00000596143,ENST00000600435

4,ENST00000447444,ENST00000447444,ENST00000447444,ENST00000541401,ENST00000541401,ENST0000C

3,ENST00000519243,ENST00000519243,ENST00000519243,ENST00000519243,ENST00000520871,ENST0000C

2,ENST00000361522,ENST00000361522,ENST00000361522,ENST00000361522,ENST00000361915,ENST0000C

5,ENST00000571215,ENST00000571215,ENST00000571215,ENST00000572725,ENST00000572725,ENST0000C

6,ENST00000357096,ENST00000357096,ENST00000357096,ENST00000357096,ENST00000357096,ENST00000

5,ENST00000371355,ENST00000371357,ENST00000371357,ENST00000371357,ENST00000371357,ENST00000

1,ENST00000595461,ENST00000595461,ENST00000595461,ENST00000598381,ENST00000598381,ENST00000

3,ENST00000434423,ENST00000434423,ENST00000434423,ENST00000446813,ENST00000446813,ENST00000

5,ENST00000512055,ENST00000512055,ENST00000512055,ENST00000512332,ENST00000512332,ENST00000

3,ENST00000437743,ENST00000437743,ENST00000437743,ENST00000440155,ENST00000440155,ENST0000C

2,ENST00000575842,ENST00000575842,ENST00000575842,ENST00000576544,ENST00000576544,ENST0000C

6,ENST00000366496,ENST00000366497,ENST00000366497,ENST00000366497,ENST00000366497,ENST0000C

6,ENST00000417856,ENST00000417856,ENST00000417856,ENST00000421552,ENST00000421552,ENST0000C
3,ENST00000446403,ENST00000446403,ENST00000446403,ENST00000453407,ENST00000453407,ENST0000C

2,ENST00000426342,ENST00000426342,ENST00000426342,ENST00000439988,ENST00000439988,ENST0000C

5,ENST00000462705,ENST00000462705,ENST00000462705,ENST00000462705,ENST00000464560,ENST0000C

3,ENST00000360243,ENST00000360243,ENST00000360243,ENST00000360243,ENST00000360243,ENST0000C
1,ENST00000422911,ENST00000422911,ENST00000422911,ENST00000422911,ENST00000433067,ENST0000C

8,ENST00000378618,ENST00000378618,ENST00000378618,ENST00000378618,ENST00000395460,ENST0000C

4,ENST00000402934,ENST00000402934,ENST00000402934,ENST00000402934,ENST00000404525,ENST0000C

4,ENST00000432264,ENST00000432264,ENST00000432264,ENST00000432264,ENST00000447116,ENST00000
9,ENST00000441319,ENST00000441319,ENST00000441319,ENST00000509063,ENST00000509063,ENST00000

5,ENST00000413465,ENST00000413465,ENST00000413465,ENST00000413465,ENST00000413465,ENST00000

6,ENST00000354416,ENST00000354416,ENST00000354416,ENST00000354416,ENST00000354416,ENST00000

8,ENST00000348028,ENST00000349350,ENST00000349350,ENST00000349350,ENST00000349350,ENST00000

0,ENST00000349350,ENST00000421100,ENST00000421100,ENST00000421100,ENST00000421100,ENST00000

3,ENST00000519243,ENST00000519243,ENST00000519243,ENST00000519243,ENST00000520871,ENST00000

2,ENST00000361522,ENST00000361522,ENST00000361522,ENST00000361522,ENST00000361915,ENST00000

2,ENST00000371432,ENST00000371432,ENST00000371432,ENST00000371432,ENST00000371436,ENST00000

6,ENST00000357096,ENST00000357096,ENST00000357096,ENST00000357096,ENST00000357096,ENST00000

5,ENST00000413465,ENST00000413465,ENST00000413465,ENST00000413465,ENST00000413465,ENST00000

3,ENST00000519243,ENST00000519243,ENST00000519243,ENST00000519243,ENST00000520871,ENST0000C

2,ENST00000361522,ENST00000361522,ENST00000361522,ENST00000361522,ENST00000361915,ENST00000

2,ENST00000371432,ENST00000371432,ENST00000371432,ENST00000371432,ENST00000371436,ENST00000

6,ENST00000357096,ENST00000357096,ENST00000357096,ENST00000357096,ENST00000357096,ENST00000

5,ENST00000413465,ENST00000413465,ENST00000413465,ENST00000413465,ENST00000413465,ENST00000

6,ENST00000367486,ENST00000367486,ENST00000367486,ENST00000445192,ENST00000445192,ENST00000

9,ENST00000320399,ENST00000320399,ENST00000320399,ENST00000320399,ENST00000320399,ENST00000
3,ENST00000519243,ENST00000519243,ENST00000519243,ENST00000519243,ENST00000520871,ENST00000

2,ENST00000361522,ENST00000361522,ENST00000361522,ENST00000361522,ENST00000361915,ENST00000

2,ENST00000371432,ENST00000371432,ENST00000371432,ENST00000371432,ENST00000371436,ENST00000

6,ENST00000357096,ENST00000357096,ENST00000357096,ENST00000357096,ENST00000357096,ENST00000

1,ENST00000371501,ENST00000371501,ENST00000371501,ENST00000371502,ENST00000371502,ENST0000C

5,ENST00000360135,ENST00000360135,ENST00000360135,ENST00000360135,ENST00000360135,ENST0000C

5,ENST00000371355,ENST00000371355,ENST00000371355,ENST00000371355,ENST00000371355,ENST0000C

9,ENST00000404049,ENST00000404049,ENST00000404049,ENST00000452825,ENST00000452825,ENST0000C

5,ENST00000413465,ENST00000413465,ENST00000413465,ENST00000413465,ENST00000413465,ENST0000C

8,ENST00000376788,ENST00000376788,ENST00000376788,ENST00000376788,ENST00000376788,ENST00000C

3,ENST00000423743,ENST00000423743,ENST00000423743,ENST00000423743,ENST00000554463,ENST00000C

1,ENST00000546561,ENST00000546561,ENST00000546561,ENST00000546561,ENST00000546561,ENST00000C

5,ENST00000550165,ENST00000550165,ENST00000550165,ENST00000551598,ENST00000551598,ENST00000C

0,ENST00000379300,ENST00000379300,ENST00000379300,ENST00000379300,ENST00000379307,ENST0000C

8,ENST00000519148,ENST00000519148,ENST00000519148,ENST00000519148,ENST00000519591,ENST0000C

8,ENST00000360108,ENST00000360108,ENST00000360108,ENST00000360108,ENST00000367567,ENST0000C

7,ENST00000557201

7,ENST00000557201

6,ENST00000478156,ENST00000479406,ENST00000480843

5,ENST00000546292

5,ENST00000601693,ENST00000601693

1,ENST00000598381,ENST00000601440,ENST00000601693

5,ENST00000553352,ENST00000554392,ENST00000555716,ENST00000553728,ENST00000603904

22883,NM_001322884,NM_001322885,NM_001322886,NM_001322887,NM_001322888,NM_001322889,NM

22883,NM_001322884,NM_001322885,NM_001322886,NM_001322887,NM_001322888,NM_001322889,NM

22883,NM_001322884,NM_001322885,NM_001322886,NM_001322887,NM_001322888,NM_001322889,NM

360967,ENST00000360967,ENST00000361163,ENST00000361163,ENST00000361163,ENST00000361163,ENS

421100,ENST00000421100,ENST00000421100,ENST00000421100,ENST00000421100,ENST00000427578,ENS
367485,ENST00000367485,ENST00000367485,ENST00000367485,ENST00000367485,ENST00000367485,ENS

520158,ENST00000520158,ENST00000520158

445192,ENST00000445192,ENST00000445192
367485,ENST00000367485,ENST00000367486,ENST00000367486,ENST00000367486,ENST00000367486,ENS

)554581,ENST00000554581,ENST00000554581,ENST00000554581,ENST00000554581,ENST00000554581,ENS

)358087,ENST00000358087,ENST00000360967,ENST00000360967,ENST00000360967,ENST00000360967,ENS
)361163,ENST00000361163,ENST00000409914,ENST00000409914,ENST00000409914,ENST00000409914,ENS

)421100,ENST00000421100,ENST00000421100,ENST00000421100,ENST00000421100,ENST00000427578,ENS
)367485,ENST00000367485,ENST00000367485,ENST00000367485,ENST00000367485,ENST00000367485,ENS

)520158,ENST00000520158,ENST00000520158

)361163,ENST00000361163,ENST00000409914,ENST00000409914,ENST00000409914,ENST00000409914,ENS

)445192,ENST00000445192,ENST00000445192
)367485,ENST00000367485,ENST00000367486,ENST00000367486,ENST00000367486,ENST00000367486,ENS

368286,ENST00000368286,ENST00000368286

390668,ENST00000390668,ENST00000390668,ENST00000390668,ENST00000390668,ENST00000390668,ENS

538913,ENST00000538913,ENST00000538913,ENST00000538913,ENST00000538913,ENST00000538913,ENS

361569,ENST00000361569,ENST00000361569,ENST00000361569,ENST00000361569,ENST00000361569,ENS

356820,ENST00000356820,ENST00000367255,ENST00000367255,ENST00000367255,ENST00000367255,ENS

483150,ENST00000483150,ENST00000483150

538146,ENST00000538146,ENST00000538146

)368286,ENST00000368286,ENST00000368286

)390668,ENST00000390668,ENST00000390668,ENST00000390668,ENST00000390668,ENST00000390668,ENS

)538913,ENST00000538913,ENST00000538913,ENST00000538913,ENST00000538913,ENST00000538913,ENS

)460472,ENST00000460472,ENST00000589042,ENST00000589042,ENST00000589042,ENST00000589042,ENS

)361569,ENST00000361569,ENST00000361569,ENST00000361569,ENST00000361569,ENST00000361569,ENS

)395436,ENST00000395436,ENST00000413756,ENST00000413756,ENST00000413756,ENST00000413756,ENS

)456271,ENST00000456271,ENST00000503492,ENST00000503492,ENST00000503492,ENST00000503492,ENS
)538146,ENST00000538146,ENST00000538146

)390668,ENST00000390668,ENST00000390668,ENST00000390668,ENST00000390668,ENST00000390668,ENS

)447912,ENST00000447912,ENST00000447912,ENST00000447912,ENST00000447912,ENST00000450288,ENS
)538913,ENST00000538913,ENST00000538913,ENST00000538913,ENST00000538913,ENST00000538913,ENS

)361569,ENST00000361569,ENST00000361569,ENST00000361569,ENST00000361569,ENST00000361569,ENS

)538146,ENST00000538146,ENST00000538146

)421100,ENST00000421100,ENST00000421100,ENST00000421100,ENST00000421100,ENST00000427578,ENS
)367485,ENST00000367485,ENST00000367486,ENST00000367486,ENST00000367486,ENST00000367486,ENS

)430669,ENST00000430669,ENST00000437951,ENST00000437951,ENST00000437951,ENST00000437951,ENS

)357272,ENST00000357272,ENST00000358537,ENST00000358537,ENST00000358537,ENST00000358537,ENS

541401,ENST00000541401,ENST00000541401

520871,ENST00000520871,ENST00000520871,ENST00000520871,ENST00000520871,ENST00000520871,ENS

361915,ENST00000361915,ENST00000361915,ENST00000361915,ENST00000361915,ENST00000361915,ENS

572725,ENST00000572725,ENST00000572725

)357096,ENST00000357096,ENST00000357096,ENST00000357096,ENST00000379240,ENST00000379240,ENS[illegible]

)371357,ENST00000371357,ENST00000371363,ENST00000371363,ENST00000371363,ENST00000371363,ENS[illegible]

)598381,ENST00000598381

)446813,ENST00000446813,ENST00000446813

)512332,ENST00000512332,ENST00000512332

)440155,ENST00000440155,ENST00000440155

)576544,ENST00000576544,ENST00000576544

)366497,ENST00000366497,ENST00000391827,ENST00000391827,ENST00000391827,ENST00000391827,ENS

)421552,ENST00000421552,ENST00000421552
)453407,ENST00000453407,ENST00000453407

)439988,ENST00000439988,ENST00000439988

)464560,ENST00000464560,ENST00000464560,ENST00000464560,ENST00000464560,ENST00000464560,ENS

)360243,ENST00000360243,ENST00000360243,ENST00000360243,ENST00000360243,ENST00000360243,ENS
)433067,ENST00000433067,ENST00000433067,ENST00000433067,ENST00000433067,ENST00000433067,ENS

)395460,ENST00000395460,ENST00000395460,ENST00000395460,ENST00000395460,ENST00000395460,ENS

)404525,ENST00000404525,ENST00000404525,ENST00000404525,ENST00000404525,ENST00000404525,ENS

)447116,ENST00000447116,ENST00000447116,ENST00000447116,ENST00000447116,ENST00000447116,ENS
)509063,ENST00000509063,ENST00000509063

)413465,ENST00000413465,ENST00000420246,ENST00000420246,ENST00000420246,ENST00000420246,ENS

)354416,ENST00000354416,ENST00000360156,ENST00000360156,ENST00000360156,ENST00000360156,ENS

)349350,ENST00000349350,ENST00000349350,ENST00000349350,ENST00000349350,ENST00000349350,ENS

)421100,ENST00000421100,ENST00000421100,ENST00000421100,ENST00000421100,ENST00000427578,ENS

)520871,ENST00000520871,ENST00000520871,ENST00000520871,ENST00000520871,ENST00000520871,ENS

)361915,ENST00000361915,ENST00000361915,ENST00000361915,ENST00000361915,ENST00000361915,ENS

)371436,ENST00000371436,ENST00000371436,ENST00000371436,ENST00000371436,ENST00000371436,ENS

)357096,ENST00000357096,ENST00000357096,ENST00000357096,ENST00000379240,ENST00000379240,ENS

)413465,ENST00000413465,ENST00000420246,ENST00000420246,ENST00000420246,ENST00000420246,ENS

)520871,ENST00000520871,ENST00000520871,ENST00000520871,ENST00000520871,ENST00000520871,ENST00000520871,ENS

)361915,ENST00000361915,ENST00000361915,ENST00000361915,ENST00000361915,ENST00000361915,ENS

)371436,ENST00000371436,ENST00000371436,ENST00000371436,ENST00000371436,ENST00000371436,ENS

)357096,ENST00000357096,ENST00000357096,ENST00000357096,ENST00000379240,ENST00000379240,ENS

)413465,ENST00000413465,ENST00000420246,ENST00000420246,ENST00000420246,ENST00000420246,ENS

)445192,ENST00000445192,ENST00000445192

320399,ENST00000320399,ENST00000320399,ENST00000320399,ENST00000320399,ENST00000320399,ENS
520871,ENST00000520871,ENST00000520871,ENST00000520871,ENST00000520871,ENST00000520871,ENS

361915,ENST00000361915,ENST00000361915,ENST00000361915,ENST00000361915,ENST00000361915,ENS

371436,ENST00000371436,ENST00000371436,ENST00000371436,ENST00000371436,ENST00000371436,ENS

357096,ENST00000357096,ENST00000357096,ENST00000357096,ENST00000379240,ENST00000379240,ENS

0371502,ENST00000371502,ENST00000371502

0360135,ENST00000360135,ENST00000360301,ENST00000360301,ENST00000360301,ENST00000360301,ENS

0371355,ENST00000371355,ENST00000371357,ENST00000371357,ENST00000371357,ENST00000371357,ENS

0452825,ENST00000452825,ENST00000452825

0413465,ENST00000413465,ENST00000420246,ENST00000420246,ENST00000420246,ENST00000420246,ENS

)376788,ENST00000376788,ENST00000415380,ENST00000415380,ENST00000415380,ENST00000415380,ENS

)554463,ENST00000554463,ENST00000554463,ENST00000554463,ENST00000554463,ENST00000554463,ENS

)546561,ENST00000546561,ENST00000552128,ENST00000552128,ENST00000552128,ENST00000552128,ENS

)551598,ENST00000551598,ENST00000551598

379307,ENST00000379307,ENST00000379307,ENST00000379307,ENST00000379307,ENST00000379307,ENS

519591,ENST00000519591,ENST00000519591,ENST00000519591,ENST00000519591,ENST00000519591,ENS

367567,ENST00000367567,ENST00000367567,ENST00000367567,ENST00000367567,ENST00000367567,ENS

I_001322890,NM_001322891,NM_001322892,NM_001322893,NM_001322894,NM_001322895,NM_0013228

I_001322890,NM_001322891,NM_001322892,NM_001322893,NM_001322894,NM_001322895,NM_0013228

I_001322890,NM_001322891,NM_001322892,NM_001322893,NM_001322894,NM_001322895,NM_0013228

T00000361163,ENST00000361163,ENST00000409914,ENST00000409914,ENST00000409914,ENST0000040991

T00000427578,ENST00000427578,ENST00000427578,ENST00000427578,ENST00000427578,ENST0000042757
T00000367486,ENST00000367486,ENST00000367486,ENST00000367486,ENST00000367486,ENST0000036748

T00000367486,ENST00000367486,ENST00000445192,ENST00000445192,ENST00000445192,ENST0000044519

T00000554848,ENST00000554848,ENST00000554848,ENST00000554848,ENST00000554848,ENST0000055484

T00000360967,ENST00000360967,ENST00000360967,ENST00000360967,ENST00000360967,ENST0000036096
T00000409914,ENST00000409914,ENST00000409914,ENST00000409914

T00000427578,ENST00000427578,ENST00000427578,ENST00000427578,ENST00000427578,ENST0000042757
T00000367486,ENST00000367486,ENST00000367486,ENST00000367486,ENST00000367486,ENST0000036748

T00000409914,ENST00000409914,ENST00000409914,ENST00000409914

T00000367486,ENST00000367486,ENST00000445192,ENST00000445192,ENST00000445192,ENST00000044519

T00000390668,ENST00000390668,ENST00000393489,ENST00000393489,ENST00000393489,ENST0000039348

T00000543873,ENST00000543873,ENST00000543873,ENST00000543873,ENST00000543873,ENST0000054387

T00000361569,ENST00000361569,ENST00000395161,ENST00000395161,ENST00000395161,ENST0000039516

T00000367255,ENST00000367255,ENST00000367255,ENST00000367255,ENST00000423061,ENST0000042306

T00000390668,ENST00000390668,ENST00000393489,ENST00000393489,ENST00000393489,ENST0000039348

T00000543873,ENST00000543873,ENST00000543873,ENST00000543873,ENST00000543873,ENST0000054387

T00000589042,ENST00000589042,ENST00000591111,ENST00000591111,ENST00000591111,ENST0000059111

T00000361569,ENST00000361569,ENST00000395161,ENST00000395161,ENST00000395161,ENST0000039516

T00000413756,ENST00000413756,ENST00000413756,ENST00000413756,ENST00000413952,ENST0000041395

T00000503492,ENST00000503492,ENST00000536585,ENST00000536585,ENST00000536585,ENST0000053658

T00000390668,ENST00000390668,ENST00000393489,ENST00000393489,ENST00000393489,ENST0000039348

T00000450288,ENST00000450288,ENST00000450288,ENST00000450288,ENST00000450288,ENST0000045028
T00000543873,ENST00000543873,ENST00000543873,ENST00000543873,ENST00000543873,ENST0000054387

T00000361569,ENST00000361569,ENST00000395161,ENST00000395161,ENST00000395161,ENST0000039516

T00000427578,ENST00000427578,ENST00000427578,ENST00000427578,ENST00000427578,ENST0000042757
T00000367486,ENST00000367486,ENST00000445192,ENST00000445192,ENST00000445192,ENST000004451[illegible]

T00000437951,ENST00000437951,ENST00000468211,ENST00000468211,ENST00000468211,ENST0000046821

T00000358537,ENST00000358537,ENST00000358537,ENST00000358537,ENST00000419752,ENST0000041975

T00000523833,ENST00000523833,ENST00000523833,ENST00000523833,ENST00000523833,ENST0000052383

T00000370161,ENST00000370161,ENST00000370161,ENST00000370161,ENST00000370161,ENST0000037016

T00000379240,ENST00000379240,ENST00000379240,ENST00000379240,ENST00000379240,ENST0000037924

T00000371363,ENST00000371363,ENST00000371372,ENST00000371372,ENST00000371372,ENST0000037137

T00000391827,ENST00000391827,ENST00000391828,ENST00000391828,ENST00000391828,ENST0000039182

T00000471418,ENST00000471418,ENST00000471418,ENST00000471418,ENST00000471418,ENST0000047141

T00000360243,ENST00000360243,ENST00000370335,ENST00000370335,ENST00000370335,ENST0000037033
T00000447016,ENST00000447016,ENST00000447016,ENST00000447016,ENST00000447016,ENST0000044701

T00000526827,ENST00000526827,ENST00000526827,ENST00000526827,ENST00000526827,ENST0000052682

T00000404816,ENST00000404816,ENST00000404816,ENST00000404816,ENST00000404816,ENST0000040481

T00000456778,ENST00000456778,ENST00000456778,ENST00000456778,ENST00000456778,ENST0000045677

T00000420246,ENST00000420246,ENST00000420246,ENST00000420246,ENST00000445888,ENST0000044588

T00000360156,ENST00000360156,ENST00000360156,ENST00000360156,ENST00000394610,ENST0000039461

T00000349350,ENST00000349350,ENST00000349350,ENST00000349350,ENST00000349350,ENST0000034935

T00000427578,ENST00000427578,ENST00000427578,ENST00000427578,ENST00000427578,ENST0000042757

T00000523833,ENST00000523833,ENST00000523833,ENST00000523833,ENST00000523833,ENST0000052383

T00000370161,ENST00000370161,ENST00000370161,ENST00000370161,ENST00000370161,ENST0000037016

T00000371438,ENST00000371438,ENST00000371438,ENST00000371438,ENST00000371438,ENST0000037143

T00000379240,ENST00000379240,ENST00000379240,ENST00000379240,ENST00000379240,ENST0000037924

T00000420246,ENST00000420246,ENST00000420246,ENST00000420246,ENST00000445888,ENST0000044588

T00000523833,ENST00000523833,ENST00000523833,ENST00000523833,ENST00000523833,ENST0000052383

T00000370161,ENST00000370161,ENST00000370161,ENST00000370161,ENST00000370161,ENST0000037016

T00000371438,ENST00000371438,ENST00000371438,ENST00000371438,ENST00000371438,ENST0000037143

T00000379240,ENST00000379240,ENST00000379240,ENST00000379240,ENST00000379240,ENST0000037924

T00000420246,ENST00000420246,ENST00000420246,ENST00000420246,ENST00000445888,ENST0000044588

T00000320399,ENST00000320399,ENST00000320399,ENST00000320399,ENST00000320492,ENST0000032049
T00000523833,ENST00000523833,ENST00000523833,ENST00000523833,ENST00000523833,ENST0000052383

T00000370161,ENST00000370161,ENST00000370161,ENST00000370161,ENST00000370161,ENST0000037016

T00000371438,ENST00000371438,ENST00000371438,ENST00000371438,ENST00000371438,ENST0000037143

T00000379240,ENST00000379240,ENST00000379240,ENST00000379240,ENST00000379240,ENST0000037924

T00000360301,ENST00000360301,ENST00000360301,ENST00000360301,ENST00000381879,ENST0000038187

T00000371357,ENST00000371357,ENST00000371357,ENST00000371357,ENST00000371363,ENST0000037136

T00000420246,ENST00000420246,ENST00000420246,ENST00000420246,ENST00000445888,ENST0000044588

T00000415380,ENST00000415380,ENST00000415380,ENST00000415380,ENST00000540495,ENST0000054049

T00000555203,ENST00000555203,ENST00000555203,ENST00000555203,ENST00000555203,ENST000005552C

T00000552128,ENST00000552128,ENST00000552128,ENST00000552128

T00000450347,ENST00000450347,ENST00000450347,ENST00000450347,ENST00000450347,ENST0000045034

T00000521193,ENST00000521193,ENST00000521193,ENST00000521193,ENST00000521193,ENST0000052119

T00000367570,ENST00000367570,ENST00000367570,ENST00000367570,ENST00000367570,ENST0000036757

[illegible]96,NM_001322897,NM_001322898,NM_001322899,NM_001322900,NM_002313,NM_006720

[illegible]896,NM_001322897,NM_001322898,NM_001322899,NM_001322900,NM_002313,NM_006720

896,NM_001322897,NM_001322898,NM_001322899,NM_001322900,NM_002313,NM_006720

.4,ENST00000409914,ENST00000409914

'8,ENST00000427578,ENST00000427578,ENST00000443370,ENST00000443370,ENST00000443370,ENST0000C
36,ENST00000367486,ENST00000445192,ENST00000445192,ENST00000445192,ENST00000445192,ENST0000C

)2,ENST00000445192,ENST00000445192

ı8,ENST00000554848,ENST00000555528,ENST00000555528,ENST00000555528,ENST00000555528,ENST00000(

ı7,ENST00000360967,ENST00000360967,ENST00000361163,ENST00000361163,ENST00000361163,ENST00000(

'8,ENST00000427578,ENST00000427578,ENST00000443370,ENST00000443370,ENST00000443370,ENST00000(
ı6,ENST00000367486,ENST00000445192,ENST00000445192,ENST00000445192,ENST00000445192,ENST00000(

)2,ENST00000445192,ENST00000445192

9,ENST00000393489,ENST00000393489,ENST00000393489,ENST00000393489,ENST00000393489,ENST00000

3,ENST00000543873,ENST00000555375,ENST00000555375,ENST00000555375,ENST00000555375,ENST00000

1,ENST00000395161,ENST00000395161,ENST00000395161,ENST00000395161,ENST00000395161,ENST00000

1,ENST00000423061,ENST00000423061,ENST00000423061,ENST00000423061,ENST00000423061,ENST00000

39,ENST00000393489,ENST00000393489,ENST00000393489,ENST00000393489,ENST00000393489,ENST0000(

73,ENST00000543873,ENST00000555375,ENST00000555375,ENST00000555375,ENST00000555375,ENST0000(

11,ENST00000591111,ENST00000591111

1,ENST00000395161,ENST00000395161,ENST00000395161,ENST00000395161,ENST00000395161,ENST0000

2,ENST00000413952,ENST00000413952,ENST00000413952,ENST00000413952,ENST00000413952,ENST0000

5,ENST00000536585,ENST00000536585

39,ENST00000393489,ENST00000393489,ENST00000393489,ENST00000393489,ENST00000393489,ENST0000(

38,ENST00000450288,ENST00000450288,ENST00000455313,ENST00000455313,ENST00000455313,ENST0000(
73,ENST00000543873,ENST00000555375,ENST00000555375,ENST00000555375,ENST00000555375,ENST0000(

51,ENST00000395161,ENST00000395161,ENST00000395161,ENST00000395161,ENST00000395161,ENST0000(

'8,ENST00000427578,ENST00000427578,ENST00000443370,ENST00000443370,ENST00000443370,ENST00000(
)2,ENST00000445192,ENST00000445192

1,ENST00000468211,ENST00000468211

2,ENST00000419752,ENST00000419752,ENST00000419752,ENST00000419752,ENST00000419752,ENST00000(

3,ENST00000523833,ENST00000524144,ENST00000524144,ENST00000524144,ENST00000524144,ENST0000C

1,ENST00000370161,ENST00000370163,ENST00000370163,ENST00000370163,ENST00000370163,ENST0000C

40,ENST00000379240,ENST00000379240,ENST00000379240,ENST00000379240,ENST00000379240,ENST00000

72,ENST00000371372,ENST00000371372

28,ENST00000391828,ENST00000391828

18,ENST00000471418,ENST00000474710,ENST00000474710,ENST00000474710,ENST00000474710,ENST0000

35,ENST00000370335,ENST00000370335,ENST00000370335,ENST00000370335,ENST00000370335,ENST0000
16,ENST00000447016,ENST00000447207,ENST00000447207,ENST00000447207,ENST00000447207,ENST0000

27,ENST00000526827,ENST00000536126,ENST00000536126,ENST00000536126,ENST00000536126,ENST0000

16,ENST00000404816,ENST00000407925,ENST00000407925,ENST00000407925,ENST00000407925,ENST0000

'8,ENST00000456778,ENST00000521353,ENST00000521353,ENST00000521353,ENST00000521353,ENST0000(

;8,ENST00000445888,ENST00000445888,ENST00000445888,ENST00000445888,ENST00000445888,ENST0000(

.0,ENST00000394610,ENST00000394610,ENST00000394610,ENST00000394610,ENST00000394610,ENST0000(

;0,ENST00000349350,ENST00000349350,ENST00000421100,ENST00000421100,ENST00000421100,ENST0000(

'8,ENST00000427578,ENST00000427578,ENST00000443370,ENST00000443370,ENST00000443370,ENST0000(

33,ENST00000523833,ENST00000524144,ENST00000524144,ENST00000524144,ENST00000524144,ENST00000

61,ENST00000370161,ENST00000370163,ENST00000370163,ENST00000370163,ENST00000370163,ENST00000

38,ENST00000371438,ENST00000465038,ENST00000465038,ENST00000465038,ENST00000465038,ENST00000

40,ENST00000379240,ENST00000379240,ENST00000379240,ENST00000379240,ENST00000379240,ENST00000

38,ENST00000445888,ENST00000445888,ENST00000445888,ENST00000445888,ENST00000445888,ENST0000(

3,ENST00000523833,ENST00000524144,ENST00000524144,ENST00000524144,ENST00000524144,ENST00000

51,ENST00000370161,ENST00000370163,ENST00000370163,ENST00000370163,ENST00000370163,ENST0000C

38,ENST00000371438,ENST00000465038,ENST00000465038,ENST00000465038,ENST00000465038,ENST0000C

40,ENST00000379240,ENST00000379240,ENST00000379240,ENST00000379240,ENST00000379240,ENST0000C

38,ENST00000445888,ENST00000445888,ENST00000445888,ENST00000445888,ENST00000445888,ENST0000C

)2,ENST00000320492,ENST00000320492,ENST00000320492,ENST00000320492,ENST00000320492,ENST0000(
33,ENST00000523833,ENST00000524144,ENST00000524144,ENST00000524144,ENST00000524144,ENST0000(

51,ENST00000370161,ENST00000370163,ENST00000370163,ENST00000370163,ENST00000370163,ENST0000(

38,ENST00000371438,ENST00000465038,ENST00000465038,ENST00000465038,ENST00000465038,ENST0000(

40,ENST00000379240,ENST00000379240,ENST00000379240,ENST00000379240,ENST00000379240,ENST0000(

[illegible]79,ENST00000381879,ENST00000381879,ENST00000381879,ENST00000381879,ENST00000381879,ENST0000[illegible]

[illegible]63,ENST00000371363,ENST00000371363,ENST00000371363,ENST00000371363,ENST00000371363,ENST0000[illegible]

[illegible]88,ENST00000445888,ENST00000445888,ENST00000445888,ENST00000445888,ENST00000445888,ENST0000[illegible]

)5,ENST00000540495,ENST00000540495,ENST00000540495,ENST00000540495,ENST00000540495,ENST00000540495,ENST0000(

)3,ENST00000555203,ENST00000555833,ENST00000555833,ENST00000555833,ENST00000555833,ENST0000(

ı7,ENST00000450347,ENST00000452989,ENST00000452989,ENST00000452989,ENST00000452989,ENST0000C

ı3,ENST00000521193,ENST00000522041,ENST00000522041,ENST00000522041,ENST00000522041,ENST0000C

'0,ENST00000367570,ENST00000367573,ENST00000367573,ENST00000367573,ENST00000367573,ENST0000C

)443370,ENST00000443370,ENST00000443370,ENST00000443370,ENST00000443370,ENST00000443370,ENS
)445192,ENST00000445192,ENST00000445192,ENST00000533951,ENST00000533951,ENST00000533951,ENS

)555528,ENST00000555528,ENST00000555528,ENST00000555926,ENST00000555926,ENST00000555926,ENS

)361163,ENST00000361163,ENST00000361163,ENST00000361163,ENST00000361163,ENST00000361163,ENS

)443370,ENST00000443370,ENST00000443370,ENST00000443370,ENST00000443370,ENST00000443370,ENS
)445192,ENST00000445192,ENST00000445192,ENST00000533951,ENST00000533951,ENST00000533951,ENS

)393489,ENST00000393491,ENST00000393491,ENST00000393491,ENST00000393491,ENST00000393491,ENS

)555375,ENST00000555375,ENST00000555375,ENST00000556155,ENST00000556155,ENST00000556155,ENS

)395161,ENST00000503911,ENST00000503911,ENST00000503911,ENST00000503911,ENST00000503911,ENS

)423061,ENST00000448038,ENST00000448038,ENST00000448038,ENST00000448038,ENST00000448038,ENS

)393489,ENST00000393491,ENST00000393491,ENST00000393491,ENST00000393491,ENST00000393491,ENS

)555375,ENST00000555375,ENST00000555375,ENST00000556155,ENST00000556155,ENST00000556155,ENS

)395161,ENST00000503911,ENST00000503911,ENST00000503911,ENST00000503911,ENST00000503911,ENS

)413952,ENST00000434526,ENST00000434526,ENST00000434526,ENST00000434526,ENST00000434526,ENS

)393489,ENST00000393491,ENST00000393491,ENST00000393491,ENST00000393491,ENST00000393491,ENS

)455313,ENST00000455313,ENST00000455313,ENST00000455313,ENST00000455313,ENST00000455313,ENS
)555375,ENST00000555375,ENST00000555375,ENST00000556155,ENST00000556155,ENST00000556155,ENS

)395161,ENST00000503911,ENST00000503911,ENST00000503911,ENST00000503911,ENST00000503911,ENS

)443370,ENST00000443370,ENST00000443370,ENST00000443370,ENST00000443370,ENST00000443370,ENS

)419752,ENST00000429972,ENST00000429972,ENST00000429972,ENST00000429972,ENST00000429972,ENS

)524144,ENST00000524144,ENST00000524144,ENST00000524168,ENST00000524168,ENST00000524168,ENS

)370163,ENST00000370163,ENST00000370163,ENST00000370165,ENST00000370165,ENST00000370165,ENS

)379244,ENST00000379244,ENST00000379244,ENST00000379244,ENST00000379244,ENST00000379244,ENS

)474710,ENST00000474710,ENST00000474710,ENST00000481632,ENST00000481632,ENST00000481632,ENS

)370335,ENST00000370335,ENST00000370335,ENST00000370335,ENST00000370335,ENST00000370335,ENS
)447207,ENST00000447207,ENST00000447207,ENST00000538201,ENST00000538201,ENST00000538201,ENS

)536126,ENST00000536126,ENST00000536126,ENST00000543718,ENST00000543718,ENST00000543718,ENS

)407925,ENST00000407925,ENST00000407925,ENST00000418533,ENST00000418533,ENST00000418533,ENS

)521353,ENST00000521353,ENST00000521353,ENST00000522736,ENST00000522736,ENST00000522736,ENS

)445888,ENST00000455263,ENST00000455263,ENST00000455263,ENST00000455263,ENST00000455263,ENS

)394610,ENST00000394616,ENST00000394616,ENST00000394616,ENST00000394616,ENST00000394616,ENS

)421100,ENST00000421100,ENST00000421100,ENST00000421100,ENST00000421100,ENST00000421100,ENS

)443370,ENST00000443370,ENST00000443370,ENST00000443370,ENST00000443370,ENST00000443370,ENS

)524144,ENST00000524144,ENST00000524144,ENST00000524168,ENST00000524168,ENST00000524168,ENS

)370163,ENST00000370163,ENST00000370163,ENST00000370165,ENST00000370165,ENST00000370165,ENS

)465038,ENST00000465038,ENST00000465038,ENST00000541979,ENST00000541979,ENST00000541979,ENS

)379244,ENST00000379244,ENST00000379244,ENST00000379244,ENST00000379244,ENST00000379244,ENS

)445888,ENST00000455263,ENST00000455263,ENST00000455263,ENST00000455263,ENST00000455263,ENS

)524144,ENST00000524144,ENST00000524144,ENST00000524168,ENST00000524168,ENST00000524168,ENS

)370163,ENST00000370163,ENST00000370163,ENST00000370165,ENST00000370165,ENST00000370165,ENS

)465038,ENST00000465038,ENST00000465038,ENST00000541979,ENST00000541979,ENST00000541979,ENS

)379244,ENST00000379244,ENST00000379244,ENST00000379244,ENST00000379244,ENST00000379244,ENS

)445888,ENST00000455263,ENST00000455263,ENST00000455263,ENST00000455263,ENST00000455263,ENS

)320492,ENST00000320492,ENST00000320492,ENST00000320492,ENST00000320492,ENST00000320492,ENS
)524144,ENST00000524144,ENST00000524144,ENST00000524168,ENST00000524168,ENST00000524168,ENS

)370163,ENST00000370163,ENST00000370163,ENST00000370165,ENST00000370165,ENST00000370165,ENS

)465038,ENST00000465038,ENST00000465038,ENST00000541979,ENST00000541979,ENST00000541979,ENS

)379244,ENST00000379244,ENST00000379244,ENST00000379244,ENST00000379244,ENST00000379244,ENS

)381879,ENST00000381885,ENST00000381885,ENST00000381885,ENST00000381885,ENST00000381885,ENS

)371363,ENST00000371367,ENST00000371367,ENST00000371367,ENST00000371367,ENST00000371367,ENS

)445888,ENST00000455263,ENST00000455263,ENST00000455263,ENST00000455263,ENST00000455263,ENS

)540495,ENST00000541205,ENST00000541205,ENST00000541205,ENST00000541205,ENST00000541205,ENS

)555833,ENST00000555833,ENST00000555833,ENST00000556134,ENST00000556134,ENST00000556134,ENS

)452989,ENST00000452989,ENST00000452989,ENST00000606214,ENST00000606214,ENST00000606214,ENS

)522041,ENST00000522041,ENST00000522041,ENST00000523676,ENST00000523676,ENST00000523676,ENS

)367573,ENST00000367573,ENST00000367573,ENST00000526775,ENST00000526775,ENST00000526775,ENS

T00000449052,ENST00000449052,ENST00000449052,ENST00000449052,ENST00000449052,ENST00000449052,ENST0000044905
T00000533951,ENST00000533951,ENST00000533951,ENST00000533951

T00000555926,ENST00000555926,ENST00000555926,ENST00000555926

T00000361163,ENST00000361163,ENST00000361163,ENST00000409914,ENST00000409914,ENST000004099[illegible]

T00000449052,ENST00000449052,ENST00000449052,ENST00000449052,ENST00000449052,ENST000004490[illegible]
T00000533951,ENST00000533951,ENST00000533951,ENST00000533951

T00000393491,ENST00000393491,ENST00000393491,ENST00000393491,ENST00000393491,ENST00000393491,ENST00000393[illegible]

T00000556155,ENST00000556155,ENST00000556155,ENST00000556155

T00000503911,ENST00000503911,ENST00000503911,ENST00000503911,ENST00000503911,ENST00000503911,ENST0000050[illegible]

T00000448038,ENST00000448038,ENST00000448038,ENST00000537033,ENST00000537033,ENST0000053[illegible]

T00000393491,ENST00000393491,ENST00000393491,ENST00000393491,ENST00000393491,ENST00000393491,ENST0000039349

T00000556155,ENST00000556155,ENST00000556155,ENST00000556155

T00000503911,ENST00000503911,ENST00000503911,ENST00000503911,ENST00000503911,ENST00000506[illegible]

T00000434526,ENST00000434526,ENST00000434526,ENST00000437307,ENST00000437307,ENST0000043730[illegible]

T00000393491,ENST00000393491,ENST00000393491,ENST00000393491,ENST00000393491,ENST000003934[illegible]

T00000535621,ENST00000535621,ENST00000535621,ENST00000535621,ENST00000535621,ENST000005356[illegible]
T00000556155,ENST00000556155,ENST00000556155,ENST00000556155

T00000503911,ENST00000503911,ENST00000503911,ENST00000503911,ENST00000503911,ENST000005067[illegible]

T00000449052,ENST00000449052,ENST00000449052,ENST00000449052,ENST00000449052,ENST0000044905

T00000429972,ENST00000429972,ENST00000429972,ENST00000490882,ENST00000490882,ENST0000049088

T00000524168,ENST00000524168,ENST00000524168,ENST00000524168

T00000370165,ENST00000370165,ENST00000370165,ENST00000370165

T00000379244,ENST00000379244,ENST00000379244,ENST00000379244,ENST00000379244,ENST00000379244,ENST000003792

T00000481632,ENST00000481632,ENST00000481632,ENST00000481632

T00000370337,ENST00000370337,ENST00000370337,ENST00000370337,ENST00000370337,ENST000003703[illegible]
T00000538201,ENST00000538201,ENST00000538201,ENST00000538201

T00000543718,ENST00000543718,ENST00000543718,ENST00000543718

T00000418533,ENST00000418533,ENST00000418533,ENST00000418533

T00000522736,ENST00000522736,ENST00000522736,ENST00000522736

T00000455263,ENST00000455263,ENST00000455263,ENST00000509690,ENST00000509690,ENST00000509[illegible]

T00000394616,ENST00000394616,ENST00000394616,ENST00000394617,ENST00000394617,ENST00000394[illegible]

T00000421100,ENST00000421100,ENST00000421100,ENST00000421100,ENST00000421100,ENST00000421[illegible]

T00000449052,ENST00000449052,ENST00000449052,ENST00000449052,ENST00000449052,ENST00000449[illegible]

T00000524168,ENST00000524168,ENST00000524168,ENST00000524168

T00000370165,ENST00000370165,ENST00000370165,ENST00000370165

T00000541979,ENST00000541979,ENST00000541979,ENST00000541979

T00000379244,ENST00000379244,ENST00000379244,ENST00000379244,ENST00000379244,ENST00000379[illegible]

T00000455263,ENST00000455263,ENST00000455263,ENST00000509690,ENST00000509690,ENST000005096[illegible]

T00000524168,ENST00000524168,ENST00000524168,ENST00000524168

T00000370165,ENST00000370165,ENST00000370165,ENST00000370165

T00000541979,ENST00000541979,ENST00000541979,ENST00000541979

T00000379244,ENST00000379244,ENST00000379244,ENST00000379244,ENST00000379244,ENST000003792

T00000455263,ENST00000455263,ENST00000455263,ENST00000509690,ENST00000509690,ENST000005096

T00000320492,ENST00000320492,ENST00000320492,ENST00000357036,ENST00000357036,ENST00000357036 [illegible]
T00000524168,ENST00000524168,ENST00000524168,ENST00000524168

T00000370165,ENST00000370165,ENST00000370165,ENST00000370165

T00000541979,ENST00000541979,ENST00000541979,ENST00000541979

T00000379244,ENST00000379244,ENST00000379244,ENST00000379244,ENST00000379244,ENST00000379244 [illegible]

T00000381885,ENST00000381885,ENST00000381885,ENST00000396923,ENST00000396923,ENST000003969[illegible]

T00000371367,ENST00000371367,ENST00000371367,ENST00000371372,ENST00000371372,ENST000003713[illegible]

T00000455263,ENST00000455263,ENST00000455263,ENST00000509690,ENST00000509690,ENST000005096[illegible]

T00000541205,ENST00000541205,ENST00000541205,ENST00000544327,ENST00000544327,ENST0000054433[illegible]

T00000556134,ENST00000556134,ENST00000556134,ENST00000556134

T00000606214,ENST00000606214,ENST00000606214,ENST00000606214

T00000523676,ENST00000523676,ENST00000523676,ENST00000523676

T00000526775,ENST00000526775,ENST00000526775,ENST00000526775

52,ENST00000449052,ENST00000449052,ENST00000449052,ENST00000538115,ENST00000538115,ENST0000

14,ENST00000409914,ENST00000409914,ENST00000409914,ENST00000409914,ENST00000409914,ENST0000

52,ENST00000449052,ENST00000449052,ENST00000449052,ENST00000538115,ENST00000538115,ENST0000

94,ENST00000393494,ENST00000393494,ENST00000393494,ENST00000393494,ENST00000393494,ENST0000

73,ENST00000506773,ENST00000506773,ENST00000506773,ENST00000506773,ENST00000506773,ENST0000

33,ENST00000537033,ENST00000537033,ENST00000537033,ENST00000537033,ENST00000537033,ENST0000

94,ENST00000393494,ENST00000393494,ENST00000393494,ENST00000393494,ENST00000393494,ENST0000

73,ENST00000506773,ENST00000506773,ENST00000506773,ENST00000506773,ENST00000506773,ENST0000

07,ENST00000437307,ENST00000437307,ENST00000437307,ENST00000437307,ENST00000437307,ENST0000

94,ENST00000393494,ENST00000393494,ENST00000393494,ENST00000393494,ENST00000393494,ENST0000

21,ENST00000535621,ENST00000535621,ENST00000535621,ENST00000563679,ENST00000563679,ENST0000

73,ENST00000506773,ENST00000506773,ENST00000506773,ENST00000506773,ENST00000506773,ENST0000

52,ENST00000449052,ENST00000449052,ENST00000449052,ENST00000538115,ENST00000538115,ENST0000

82,ENST00000490882,ENST00000490882,ENST00000490882,ENST00000490882,ENST00000490882,ENST0000

44,ENST00000379244,ENST00000379246,ENST00000379246,ENST00000379246,ENST00000379246,ENST0000

37,ENST00000370337,ENST00000370337,ENST00000370337,ENST00000370337,ENST00000370337,ENST0000

90,ENST00000509690,ENST00000509690,ENST00000509690,ENST00000509690,ENST00000509690,ENST0000

17,ENST00000394617,ENST00000394617,ENST00000394617,ENST00000394617,ENST00000394617,ENST0000

00,ENST00000421100,ENST00000421100,ENST00000421100,ENST00000427578,ENST00000427578,ENST0000

52,ENST00000449052,ENST00000449052,ENST00000449052,ENST00000538115,ENST00000538115,ENST0000

44,ENST00000379244,ENST00000379246,ENST00000379246,ENST00000379246,ENST00000379246,ENST0000

90,ENST00000509690,ENST00000509690,ENST00000509690,ENST00000509690,ENST00000509690,ENST00000509690,ENST0000

44,ENST00000379244,ENST00000379246,ENST00000379246,ENST00000379246,ENST00000379246,ENST0000

90,ENST00000509690,ENST00000509690,ENST00000509690,ENST00000509690,ENST00000509690,ENST0000

36,ENST00000357036,ENST00000357036,ENST00000357036,ENST00000357036,ENST00000357036,ENST0000

44,ENST00000379244,ENST00000379246,ENST00000379246,ENST00000379246,ENST00000379246,ENST0000

23,ENST00000396923,ENST00000396923,ENST00000396923,ENST00000396923,ENST00000396923,ENST0000

72,ENST00000371372,ENST00000371372,ENST00000371372,ENST00000371372,ENST00000371372,ENST0000

90,ENST00000509690,ENST00000509690,ENST00000509690,ENST00000509690,ENST00000509690,ENST0000

27,ENST00000544327,ENST00000544327,ENST00000544327,ENST00000544327,ENST00000544327,ENST0000

0538115,ENST00000538115,ENST00000538115,ENST00000538115,ENST00000538115,ENST00000538115,ENS

0409914,ENST00000409914,ENST00000409914,ENST00000409914

0538115,ENST00000538115,ENST00000538115,ENST00000538115,ENST00000538115,ENST00000538115,ENS

0393494,ENST00000393494,ENST00000393494,ENST00000393494,ENST00000393498,ENST00000393498,ENS

0506773,ENST00000506773,ENST00000506773,ENST00000506773,ENST00000511167,ENST00000511167,ENS

0540663,ENST00000540663,ENST00000540663,ENST00000540663,ENST00000540663,ENST00000540663,ENS

0393494,ENST00000393494,ENST00000393494,ENST00000393494,ENST00000393498,ENST00000393498,ENS

0506773,ENST00000506773,ENST00000506773,ENST00000506773,ENST00000511167,ENST00000511167,ENS

0451338,ENST00000451338,ENST00000451338,ENST00000451338,ENST00000451338,ENST00000451338,ENS

0393494,ENST00000393494,ENST00000393494,ENST00000393494,ENST00000393498,ENST00000393498,ENS

0563679,ENST00000563679,ENST00000563679,ENST00000563679,ENST00000563679,ENST00000563679,ENS

0506773,ENST00000506773,ENST00000506773,ENST00000506773,ENST00000511167,ENST00000511167,ENS

)0538115,ENST00000538115,ENST00000538115,ENST00000538115,ENST00000538115,ENST00000538115,ENS

)0493452,ENST00000493452,ENST00000493452,ENST00000493452,ENST00000493452,ENST00000493452,ENS

0379246,ENST00000379246,ENST00000379246,ENST00000379246,ENST00000379246,ENST00000379246,ENS

)0370337,ENST00000370337,ENST00000370337,ENST00000370337,ENST00000370340,ENST00000370340,ENS

0514944,ENST00000514944,ENST00000514944,ENST00000514944,ENST00000514944,ENST00000514944,ENS

0489216,ENST00000489216,ENST00000489216,ENST00000489216,ENST00000489216,ENST00000489216,ENS

0427578,ENST00000427578,ENST00000427578,ENST00000427578,ENST00000427578,ENST00000427578,ENS

0538115,ENST00000538115,ENST00000538115,ENST00000538115,ENST00000538115,ENST00000538115,ENS

)0379246,ENST00000379246,ENST00000379246,ENST00000379246,ENST00000379246,ENST00000379246,ENS

0514944,ENST00000514944,ENST00000514944,ENST00000514944,ENST00000514944,ENST00000514944,ENS

0379246,ENST00000379246,ENST00000379246,ENST00000379246,ENST00000379246,ENST00000379246,ENS

0514944,ENST00000514944,ENST00000514944,ENST00000514944,ENST00000514944,ENST00000514944,ENS

0357036,ENST00000357036,ENST00000357036,ENST00000357036,ENST00000357036,ENST00000357036,ENS

0379246,ENST00000379246,ENST00000379246,ENST00000379246,ENST00000379246,ENST00000379246,ENS

0420765,ENST00000420765,ENST00000420765,ENST00000420765,ENST00000420765,ENST00000420765,ENS

0545473,ENST00000545473,ENST00000545473,ENST00000545473,ENST00000545473,ENST00000545473,ENS

0514944,ENST00000514944,ENST00000514944,ENST00000514944,ENST00000514944,ENST00000514944,ENS

0546215,ENST00000546215,ENST00000546215,ENST00000546215,ENST00000546215,ENST00000546215,ENS

ST00000538115,ENST00000542585,

ST00000538115,ENST00000542585,

[illegible]T00000393498,ENST00000393498,

[illegible]T00000511167,ENST00000511167,

[illegible]T00000540663,ENST00000540663

ST00000393498,ENST00000393498,

;T00000511167,ENST00000511167,

;T00000451338,ENST00000451338

ST00000393498,ENST00000393498,

ST00000563679,ENST00000567306,

ST00000511167,ENST00000511167,

ST00000538115,ENST00000542585,

ST00000493452,ENST00000493452

ST00000379246,ENST00000379246,

ST00000370340,ENST00000370340,

ST00000514944,ENST00000514944

ST00000489216,ENST00000489216

ST00000427578,ENST00000427578,

ST00000538115,ENST00000542585,

ST00000379246,ENST00000379246,

ST00000514944,ENST00000514944

ST00000379246,ENST00000379246,

ST00000514944,ENST00000514944

;T00000357036,ENST00000357036,

;T00000379246,ENST00000379246,

;T00000420765,ENST00000420765

;T00000545473,ENST00000545473

;T00000514944,ENST00000514944

ST00000546215,ENST00000546215
